# Harnessing Noncovalent π‑Type Interactions in Thiourea–Chloride Supramolecular Complexes: Toward the Asymmetric Dearomatization of Diazaheterocycles

**DOI:** 10.1021/acscatal.5c04438

**Published:** 2025-08-06

**Authors:** Marta Velázquez, Pilar Elías-Rodríguez, Tomás Tejero, Rosario Fernández, Pedro Merino, José M. Lassaletta, David Monge

**Affiliations:** † Departamento de Química Orgánica, Facultad de Química, 16778 Universidad de Sevilla and Centro de Innovación en Química Avanzada (ORFEO-CINQA), C/Prof. García González, 1, 41012 Sevilla, Spain; ‡ Instituto de Síntesis Química y Catálisis Homogénea (ISQCH), Universidad de Zaragoza-CSIC, 50009 Zaragoza, Spain; § Instituto de Biocomputación y Física de Sistemas Complejos (BIFI), 16765 Universidad de Zaragoza, 50009 Zaragoza, Spain; ∥ Instituto de Investigaciones Químicas (CSIC-US) and Centro de Innovación en Química Avanzada (ORFEO-CINQA), Avda. Américo Vespucio, 49, 41092 Sevilla, Spain

**Keywords:** anion-binding catalysis, mechanism, noncovalent interactions, supramolecular complex, thiourea, diazaheterocycle, dearomatization, cyclic hydrazino phosphonates

## Abstract

Enantioselective dearomatization of diazaheterocycles through anion-binding catalysis has been developed. The process involves the nucleophilic addition of phosphorus nucleophiles to *in situ* generated *N*-benzoyliminium chlorides, using a *tert*-leucine-derived thiourea as an H-bond donor catalyst, thereby providing access to appealing cyclic hydrazino phosphonates and their derivatives. Mechanistic investigations suggest that the low solubility of these salts might favor the formation of a supramolecular thiourea–chloride–iminium 2:1:1 complex, which is proposed as the catalytically relevant species accounting for the observed nonlinear effect. Experimental and computational data support a 4H-activation model via this highly ordered ion pair, in which two thiourea molecules are arranged in an antiparallel orientation around the chloride, generating a *C*_2_-symmetric groove. Hence, stereodefined insertion of the *N*-benzoyliminium is ensured by key noncovalent π-type interactions, thereby maximizing the enantioinduction event.

## Graphical abstract

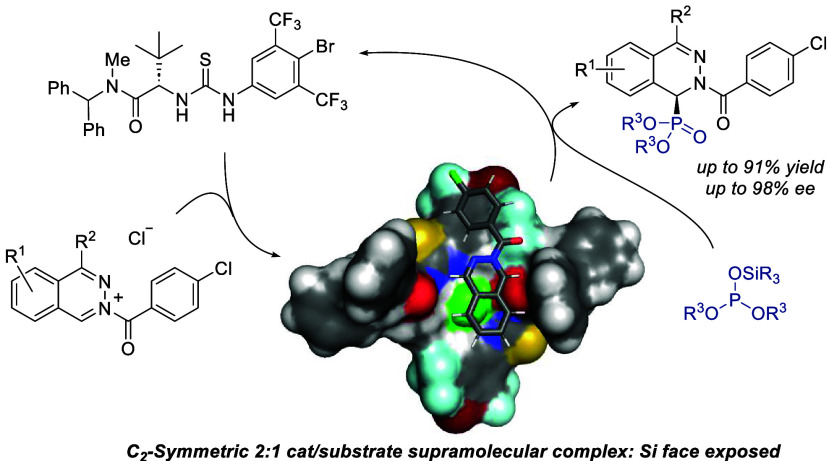

## Introduction

Charged or polarized substrates and intermediates are ubiquitous in chemical and biological systems.^1^ Inspired by the anion-recognition capabilities of biomolecules,^2^ organocatalysts, designed to mimic enzymatic active sites, have been developed to facilitate appealing organic transformations.^3^ In asymmetric anion-binding catalysis, bi- and multidentate H-bond donors have enabled high efficiency and stereocontrol.^4^ Notably, diverse scaffolds in catalysts or reagents have been enrolled in key noncovalent interactions such as secondary hydrogen bonds, π–π, cation–π, halogen–π, and London dispersion forces. These multiple interactions, operating in a cooperative manner, allow the formation of highly ordered supramolecular complexes, thereby accounting for highly selective processes.^5^ Somewhat complex activation models have been proposed depending on catalysts (2H-, 3H-, 4H-, or superior H-bond donors), substrates (neutral ionizable substrates, preformed ion pairs, and others), involved anions (chloride, fluoride, triflate, etc.), and reaction heterogeneity (Scheme 1A). In this regard, mechanistic studies have primarily focused on homogeneous reactions involving neutral starting materials, where the catalyst promotes substrate ionization through an anion abstraction process. Jacobsen and co-workers have reported 2H- and 4H-activation modes exerted by thioureas in different contexts. Notably, indicating a cooperative 4H–Cl-activation mode, detailed mechanistic studies on the addition of silyl ketene acetals to 1-chloroisochromans supported 2:1:1 catalyst–chloride–substrate complexes as the relevant enantio-determining transition states (Scheme 1B).^6^ These studies led to the development of linked bis-thiourea catalysts, expanding the repertoire of synthetic tools for organic synthesis.^7^ Anion recognition of preformed *N*-acyliminium intermediates by different types of chiral X–H-bond donor catalysts proved to be a powerful tool for asymmetric dearomatization of heterocycles employing mostly silylated nucleophiles.^8^ For example, García-Mancheño and co-workers have disclosed the unique features of tetrakistriazoles as catalysts for the asymmetric dearomatizations of quinolinium and pyridinium chlorides (Scheme 1C).^9^ ^1^H NMR and circular dichroism (CD) titration experiments supported the generation of helical supramolecular multidentate C–H/chloride complexes, enabling an efficient chirality transfer.^10^ In the search for new activation models, a few years ago, we demonstrated that specifically designed nucleophiles, such as *N*-*tert*-butylhydrazones, might also participate as bidentate H-bond donors enrolled in the molecular recognition of isoquinolinium chlorides, providing excellent stereocontrol in the formation of two contiguous stereogenic centers (Scheme 1D).^11^ Despite intense research in this field, the mechanistic aspects of those catalytic systems in some applications remain unclear, partly due to the operational challenges associated with the use of predominantly insoluble ionic substrates, leading to heterogeneous reactions, which hinder the collection of experimental evidence, such as the detection of catalytically relevant interactions by ^1^H NMR. In this work, we present a detailed mechanistic study combining experimental [nonlinear effect (NLE), ^1^H NMR, and MS studies] and computational data [molecular dynamics (MD) and density functional theory (DFT)] employing a challenging heterogeneous reaction. The catalytic system disclosed herein has allowed the development of the asymmetric dearomatization of diazaheterocycles with phosphorus nucleophiles, a virtually unknown enantioselective transformation for phthalazines^12^ or quinazolines, among others.

**Scheme 1:**
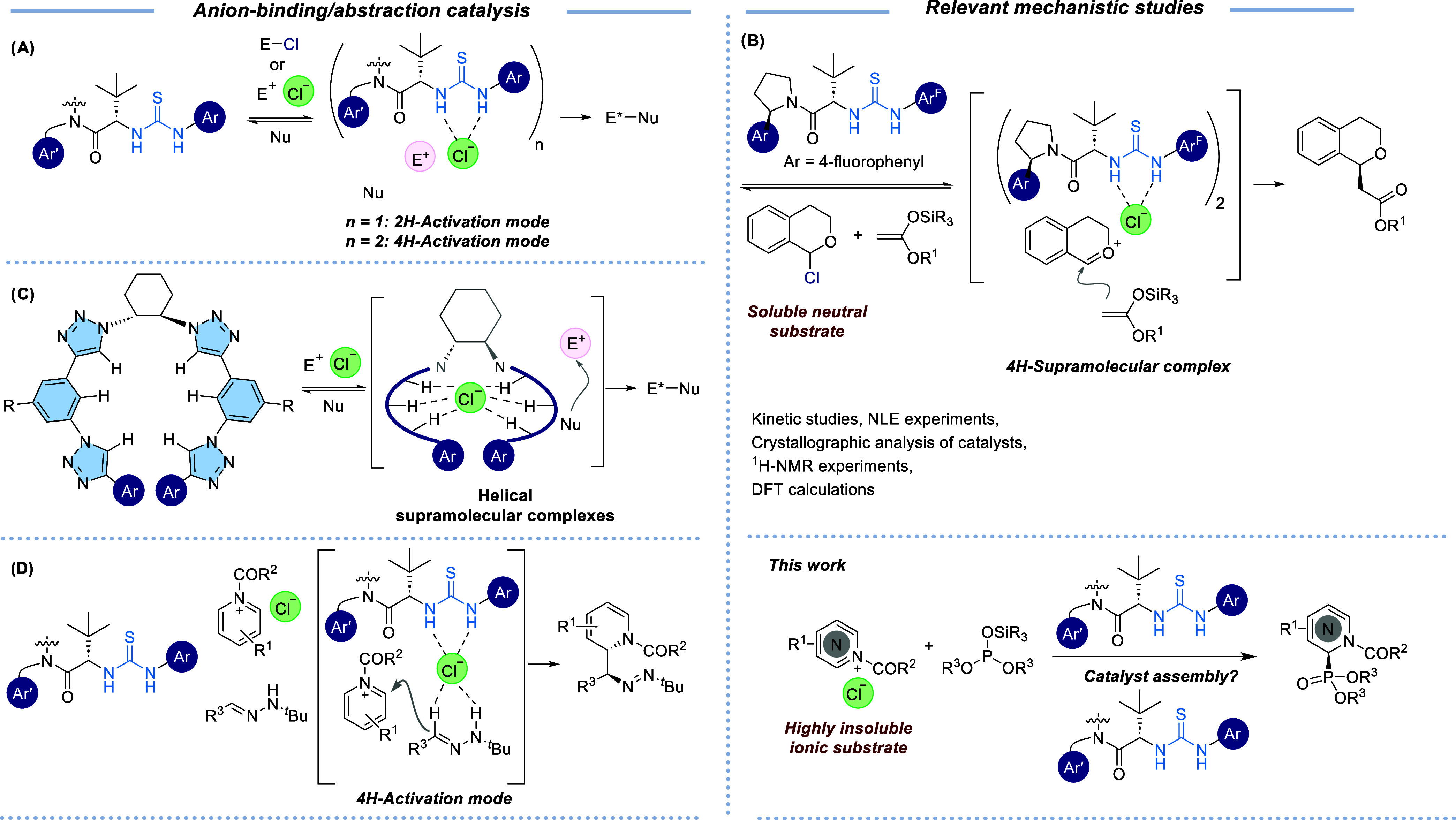
Selected Activation Modes in Anion-Binding Catalysis by H-Bond Donors

## Reaction Development

Preliminary experiments were conducted employing phthalazine (**1a**) as the model diaza-heterocyclic substrate, 2,2,2-trichloroethylchloroformate (TrocCl) or benzoyl chloride (BzCl) as acylating reagents, and *tert*-butyldimethylsilyl diethyl phosphite (**2A**) as the nucleophile (Table 1). Although quite insoluble phthalazinium salts were generated, significant background reactions were observed in methyl *tert*-butyl ether (MTBE), even at low temperatures: employing a temperature gradient (from −78 °C to room temperature over 18 h), (*rac*)-dihydrophthalazines **3aA** and **4aA** were obtained in 99 and 74% NMR yields, respectively (entries 1 and 2). From the initial screening of hydrogen-bond donor (HBD) organocatalysts (5 mol %), *tert*-leucine-derived thiourea **I** was identified as the most promising one,^13^ affording (*S*)-**3aA** in 82% NMR yield and 47% *ee* (entry 3). In accordance with previous studies,^14^ the use of benzoyl chloride as an acylating reagent had a positive impact on the enantioselectivity, presumably due to a key π-type interaction [(*S*)-**4aA**, 91%, 82% *ee* (entry 4)]. Thioureas **II**–**VI**, containing different dialkylamino groups, were additionally evaluated. The presence of a *p*-fluorophenyl ring in catalyst **II** had little impact on the reaction outcome, while catalysts **III** and **IV**, bearing pyrrolidine scaffolds with a more extended phenanthrene π-system or a benzhydryl group at position 2, provided (*S*)-**3aA** in lower enantioselectivities (40% and 7% *ee*, respectively). Remarkably, the presence of more flexible acyclic *N*-methyl, *N*-benzyl, or benzhydryl groups in organocatalysts **V** and **VI** proved to be beneficial for enantioinduction (85–87% *ee*), achieving a maximum yield of 76%. Next, the fine-tuning process of catalyst **VI** (evaluation of alternative ureas, thioureas, and squaramides bearing distinctive aryl fragments)^13^ led to thiourea **VII** as the optimal catalyst, affording the desired product **4aA** in high yield (84%) and 90% *ee* (entry 5). The better catalytic performance of **VII** might be correlated with the higher acidity of the NH group attached to the 4-bromo-3,5-bis­(trifluoromethyl)­phenyl fragment. Nevertheless, it cannot be ruled out that this improvement may result from more efficient π–π or halogen–π-type interactions involving this key aryl fragment. Additionally, other solvents, concentrations (in MTBE), and fixed temperatures (−78 °C) were unsuccessfully tested.^13^ Under optimal conditions, model reactions on a 0.2 mmol scale afforded **3aA** and **4aA** without compromising the enantioselectivities (71–86% *ee*) or chemical yields [99 and 70% of isolated yield, respectively (entries 6 and 7)].

**Table 1:**
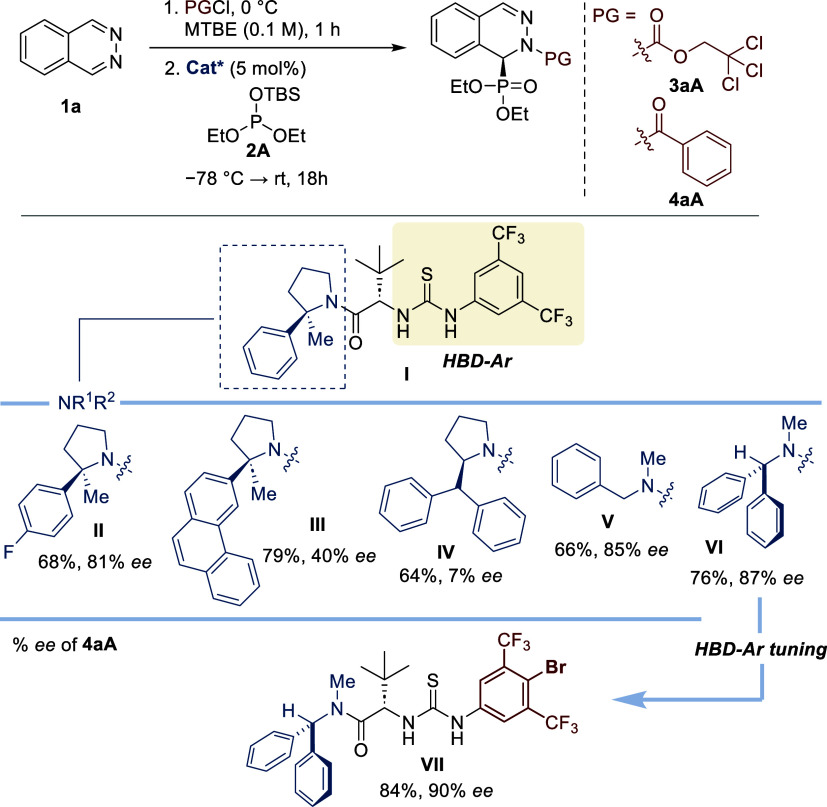
Screening of Organocatalysts and Optimization of the Reaction Parameters^a^

entry	PG	cat.	3/4	yield (%)^b^	*ee* (%)^c^
1	Troc		**3aA**	99	
2	Bz		**4aA**	74	
3	Troc	**I**	**3aA**	82	47
4	Bz	**I**	**4aA**	91	82
5	Bz	**VII**	**4aA**	84	90
6	Troc	**VII**	**3aA**	99^d^	71
7	Bz	**VII**	**4aA**	70^d^	86

a Reactions performed at the 0.1 mmol scale.

b Determined by ^1^H NMR using mesitylene as the internal standard.

c Determined by HPLC analysis.

d Isolated yields of reactions at the 0.2 mmol scale. HBD = Hydrogen-bond donor.

As expected, the nature of the halide anion modified the reaction outcome (Scheme 2). Hence, bromide binding led to a lower yield (57%) and enantioselectivity (80% *ee*), while the use of benzoyl fluoride as an acylating reagent was unproductive. The influence of the acyl protecting group was also investigated. In the carbamate series, benzyloxycarbonyl (Cbz) provided even worse enantioselectivity than Troc (**5aA**, 41% *ee*). Moreover, replacing a phenyl group with a cyclohexyl group also led to a decrease in stereochemical control (**6aA**, 75% *ee*), highlighting the suitability of an aromatic ring next to the carbonyl group as the optimal scaffold. We envisioned that this aromatic group participates in π-type interactions with the catalyst’s aromatic fragments, prompting the evaluation of aryl groups with different stereoelectronic topologies. Electron-rich aromatic groups, *p*-methyl and *p*-methoxyphenyl, afforded essentially the same enantioselectivity as the phenyl group (**7aA**: 88% *ee*, **8aA**: 87% *ee*), while electron-deficient *p*-trifluoromethylphenyl provided **9aA** in an excellent 95% *ee*, albeit in lower yield (58%). Importantly, halogenated aryls afforded the highest chemical yields and enantioselectivities: *p*-bromophenyl (**10aA**: 76%, 91% *ee*) and *p*-chlorophenyl (**11aA**: 82%, 95% *ee*). These results point toward halogen–π interactions as a significant stabilizing factor in the enantio-determining transition state. Finally, other chlorophenyl groups (*ortho*- and *meta*-) and condensed aromatic groups (2-naphthyl and 1-naphthyl) were tested without further improvement (**12aA**: 73%, 84% *ee*; **13aA**: 56%, 92% *ee*; **14aA**: 48%, 86% *ee*; **15aA**: 62%, 87% *ee*). Next, the influence of the silyl protecting group was analyzed. Neither triisopropylsilyl [**2A**-**TIPS** → **4Aa** (77%, 92% *ee*)] nor trimethylsilyl [**2A**-**TMS** → **4Aa** (58%, 92% *ee*)] improved the results obtained with *tert*-butyldimethylsilyl (TBS) in model reagent **2A**. The minimal effect of the silyl group on the enantioselectivity suggests that this fragment does not play a very important role in the enantioselectivity-determining step.

**Scheme 2:**
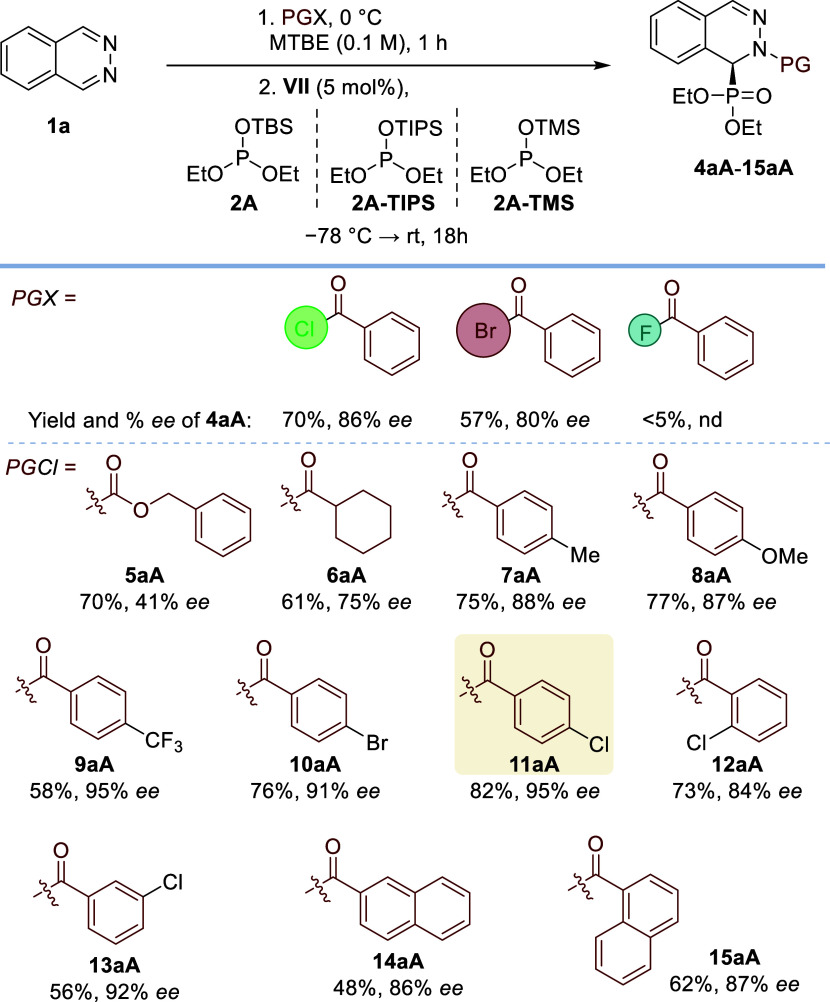
Optimization of Acylating and Silyl Protecting Groups^a^

a Reactions performed at the 0.2 mmol scale. Yields given for isolated products after chromatography. *ee*’s were determined by HPLC on chiral stationary phases.

Next, the reactivity profile of **1a** and **2A**, using *p*-chlorobenzoyl chloride as an acylating reagent, was investigated in more detail.^13^ At the beginning of the catalytic reaction (from −78 to −50 °C, 5 h), the conversion was low (24% NMR yield). A significant acceleration was observed from −50 to −25 °C, reaching 61% NMR yield after 6 h. An additional 12 h (ranging from −25 to 20 °C) was needed to reach a maximum NMR yield of 80%. Notably, the enantioselectivity (95% *ee*) remained constant throughout the process. The noncatalyzed reaction exhibited a similar profile with lower reactivity, affording *rac*-**11aA** in 35% NMR yield after 18 h.

## Mechanistic Investigations

In accordance with previous related processes involving isoquinolinium salts and *tert*-leucine-derived thioureas^10,15^ and with the collected experimental data, an anion-binding activation mechanism can be assumed for the reaction. Additionally, thiourea might also play a role as a phase-transfer catalyst, given the heterogeneous nature of the reactions.^10^ Aiming to further understand the process, we conducted experimental (NLE, ^1^H NMR, and MS studies) and computational studies. The latter include molecular dynamics simulations (to explore in detail phenomena such as aggregation, binding affinities, or conformational changes that require analysis over time) and DFT calculations (which provide more accurate geometries and energies of the various stationary points relevant to the process, thereby enabling the prediction of reactivity and selectivity). This combined synergistic methodology allows for a more comprehensive understanding of the complex molecular system under study.

## Experimental Insight for the Activation Model

Initially, experimental evidence for the proposed chloride binding was collected from mass spectrometry analysis. The negative ion ESI-MS spectrum of a representative sample, prepared from a heterogeneous solution of *p*-chlorobenzoylphthalazinium chloride with catalytic amounts of thiourea **VII** in MTBE (first step of the reaction protocol), detected (thiourea–chloride)^−^ complexes in both 1:1 (2H-activation mode) and 2:1 (4H-activation mode) stoichiometries.^13^ Similar MS spectra were recorded in other solvents such as THF and CH_2_Cl_2_.

To shed light on whether one or two molecules of the catalyst were involved as 2H/4H-bond donors, the synthesis of **11aA** was performed by employing catalyst **VII** at various enantiomeric ratios. From the collected data, a strong positive nonlinear effect (NLE) was observed (Figure 1), suggesting the involvement of at least two catalyst molecules in the enantio-determining step.

**Figure 1:**
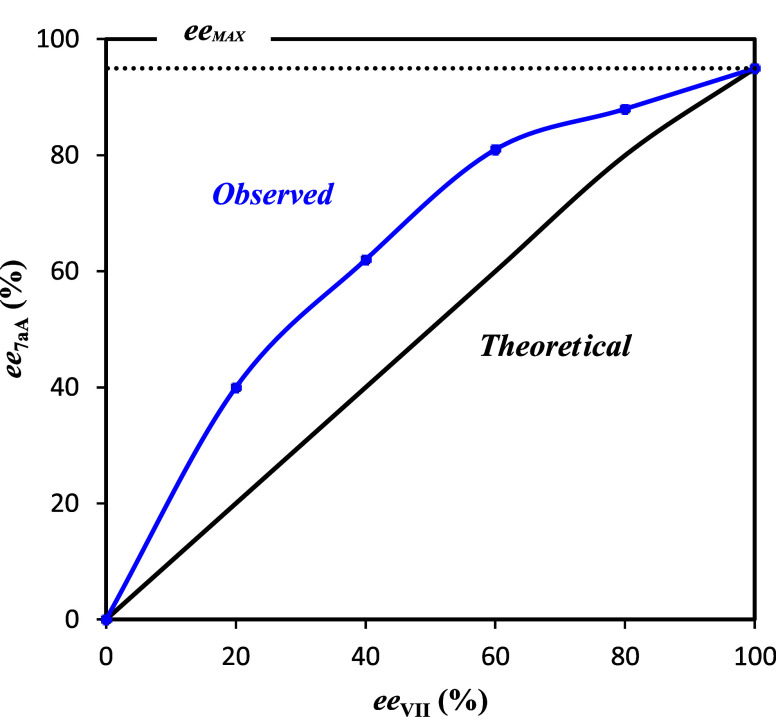
Nonlinear effect study (*ee*_**VII**_ *vs ee*_**7aA**_).

### NMR Studies of a Thiourea–Chloride Model Complex

Attempts to perform experiments with *p*-chlorobenzoylphthalazinium chloride under representative reaction conditions failed due to solubility issues. Instead, the binding of catalyst **VII** to 2-benzylphthalazin-2-ium chloride, a model substrate chosen to keep similar π-electron density, was analyzed by ^1^H NMR titration experiments in CD_2_Cl_2_ under representative reaction conditions.^16^ As shown in Figure 2, the thiourea N–H protons (H_1_ and H_2_) and the *ortho* C–H protons of the 4-bromo-3,5-bis­(trifluoromethyl)­phenyl group (H_3_)^17^ are strongly shifted downfield upon the stepwise addition of the chloride salt, supporting the molecular recognition of chloride by the thiourea. It is worth highlighting that between 0.6 and 0.8 equiv of salts added, a dynamic behavior between different species is observed, as evidenced by the line broadening of H_1_ and H_2_ signals, along with serpentine trends for some signals not directly involved in the main interaction with chloride (such as the ^*t*^Bu and *N*-Me groups of the *tert*-leucine fragment).^13^ These observations support a binding model in which an initial 2:1 thiourea/chloride complex forms and subsequently evolves into a 1:1 complex at higher substrate concentrations. Additionally, the binding constant analysis employing BindFit v0.5 software suggests a better fit for a 2:1 noncooperative binding model,^18^ which would be consistent with the observed nonlinear effect and MD simulations (vide infra). Next, complexes prepared using different thiourea/salt ratios (2:1, 1:1, and 1:2) in CH_2_Cl_2_ were comparatively analyzed by ESI-MS: A 2:1 stoichiometric model was detected for substoichiometric amounts of salt, while a 1:1 stoichiometric model was detected for salt concentrations exceeding 1 equiv. As shown in Figure 3 (left), the signals **a** and **b** for diagnostic protons in the diazaheterocycle ring shifted upfield in the supramolecular complex [**VII**:salt (2:1)]. This is a typical behavior of a π-stacking event between a guest (salt) and a host (catalyst). Additionally, signal **c** of the benzylic CH_2_ protons of phthalazinium chloride, which appears as a singlet in the ^1^H NMR spectrum of the salt, becomes two diastereotopic doublets (*J* = 13.6 Hz) in the complex, supporting the insertion of the phthalazinium ion into the stereodefined groove, which resulted in the formation of a well-defined chiral contact ion pair, as shown in the simulation by MD (see Figure 5). The antiparallel arrangement of the 2:1 complex has been confirmed to exist in solution through nuclear overhauser effect spectroscopy (NOESY) and rotating-frame nuclear overhauser effect spectroscopy (ROESY) NMR spectra.^13^ These spectra show a distinct intermolecular correlation between the *ortho* protons H_3_ and the protons of the *tert*-butyl group, as well as with the aromatic signals of the benzhydryl scaffold (Figure 3, right).

**Figure 2:**
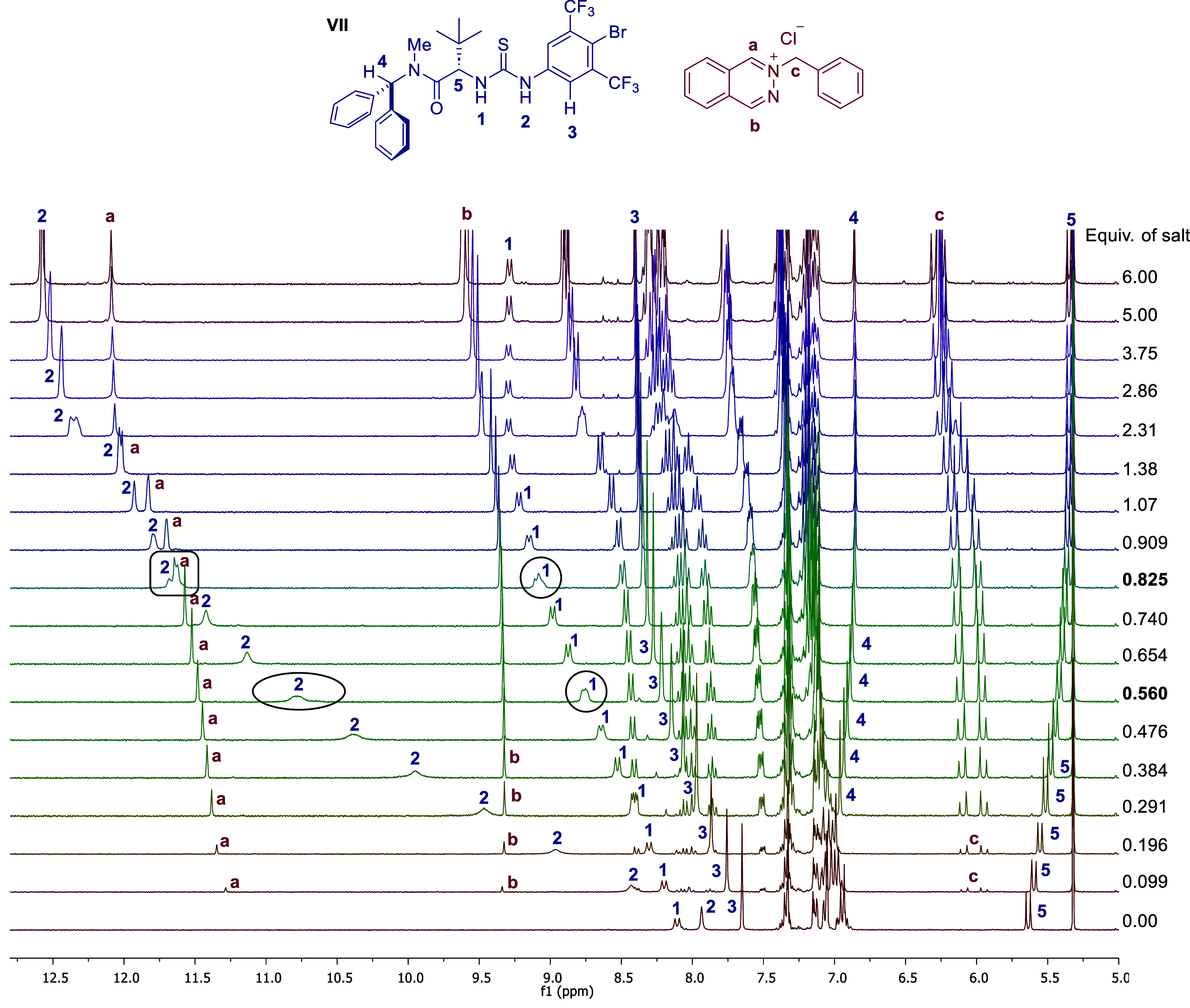
^1^H NMR titration experiment of **VII** with 2-benzylphthalazin-2-ium chloride (CD_2_Cl_2_, [**VII**] = 0.01M).

**Figure 3:**
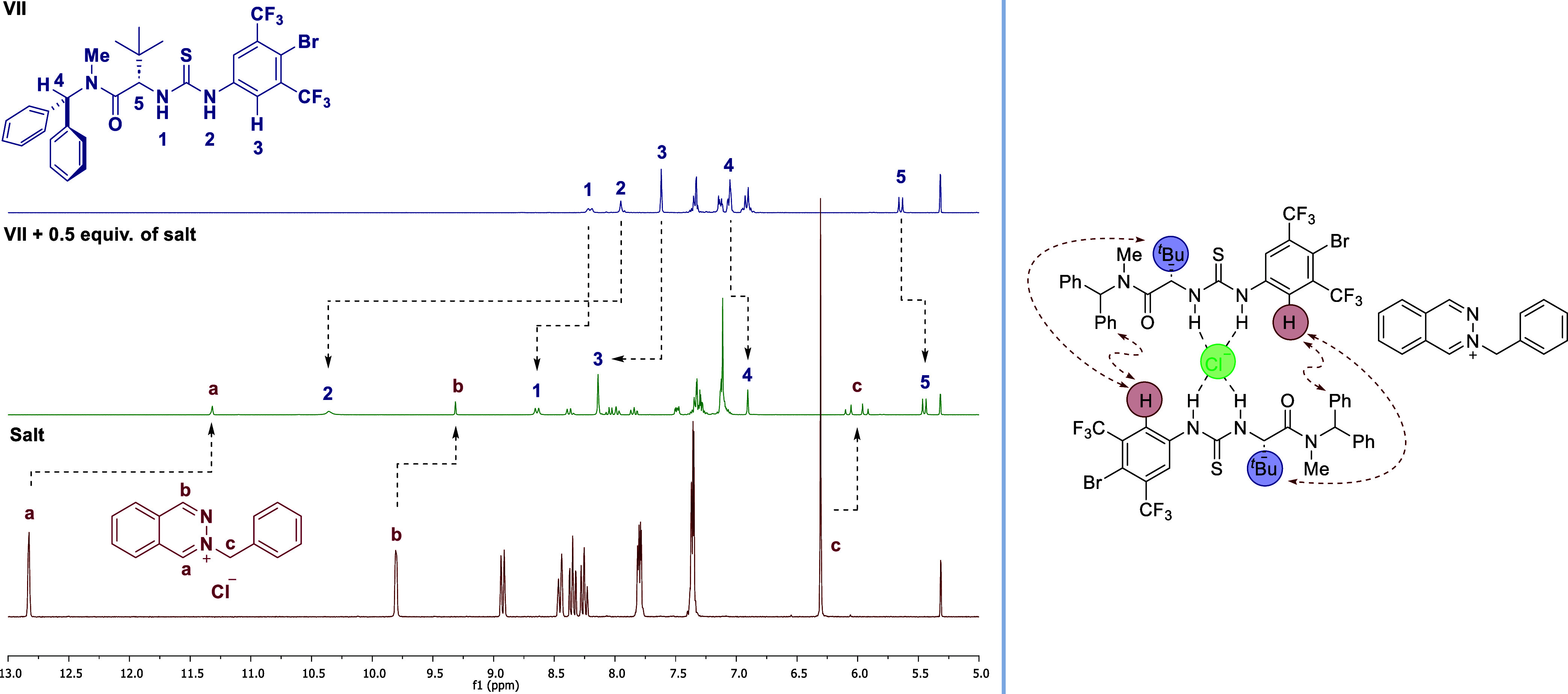
Comparison of ^1^H NMR spectra of catalyst **VII**, 2-benzylphthalazin-2-ium chloride, and complex **VII**:salt (2:1) in CD_2_Cl_2_ (left). NOE correlations support the solution-state structure of the supramolecular complex **VII**:salt (2:1) (right).

**Figure 4:**
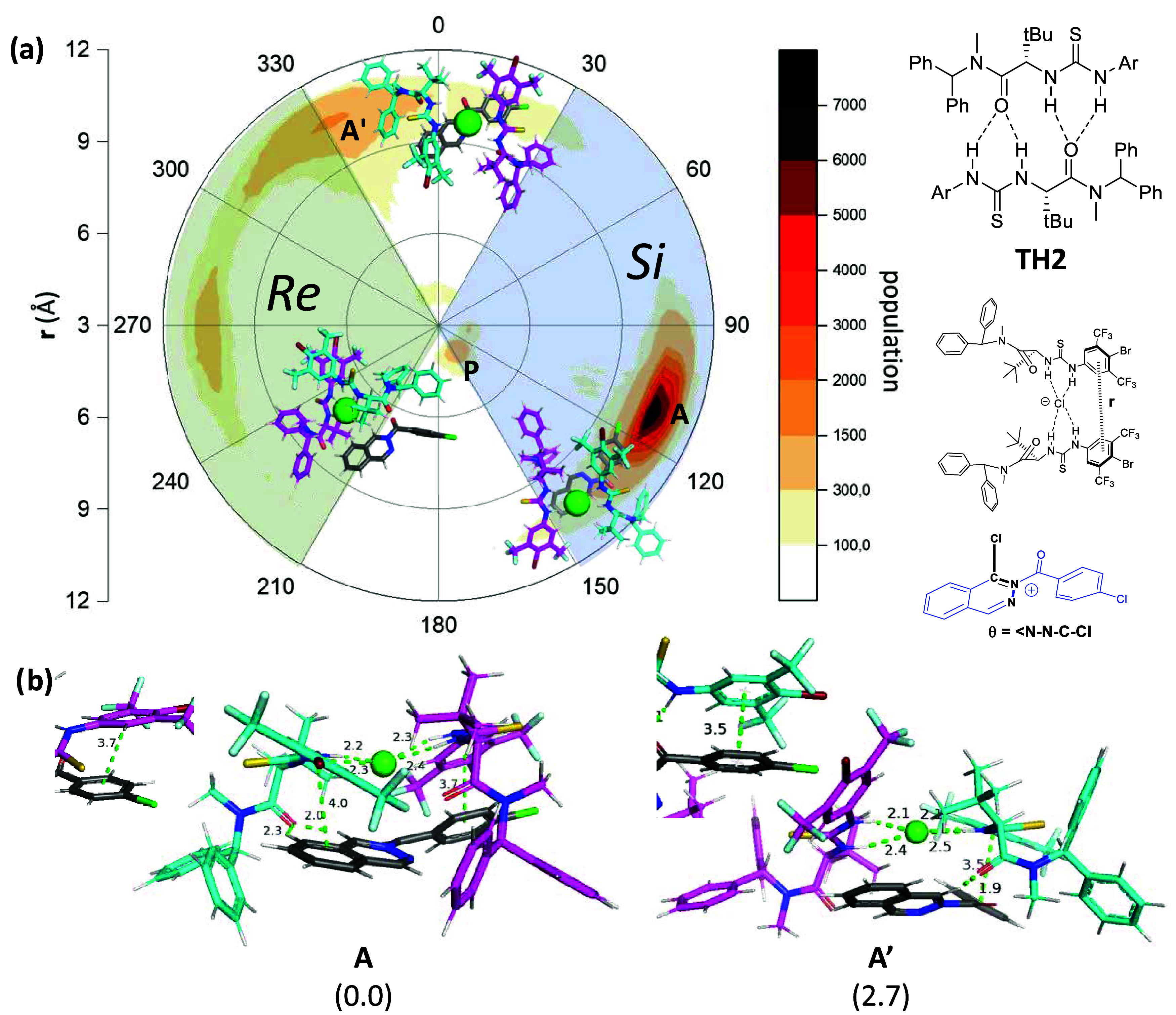
(a) Polar representation of the population of the complexes **A**, **A′**, and **P** formed between two thioureas and the 2-acylphtalazinium ion. Distance (**r**) corresponds to the distance between the two 4-bromo-3,5-bis­(trifluoromethyl)­phenyl groups. 3–4 Å corresponds to a parallel orientation of thioureas (in which a π–π stacking is present), while 10–12 Å corresponds to an antiparallel orientation. The angle (θ) corresponds to the dihedral angle defined as N–N­(COAr)–C–Cl. 30–150 degrees correspond to the complex showing the *Si* face, and 210–330 degrees correspond to the complex showing the *Re* face. (Population analysis elaborated with 365.000 points taken from seven replicas of MD simulations of the complex introduced in a THF box solvent). (b) Optimized structures of **A** and **A′** at the wb97xd/6–31+G­(d,p) level of theory. Relative energy values, given in kcal/mol, have been calculated at the wb97xd/6–311+G­(d,p)/smd = THF level of theory.

Interestingly, no NOE correlations are observed in complexes with salt concentrations exceeding 1 equiv, which supports the trend change noted in the titration experiments. In these 1:1 and 1:2 complexes, a less efficient chiral environment around the phthalazinium ion is confirmed by the tendency of benzylic proton signals to progressively coalesce. Therefore, considering that the real acyl-phthalazinium chloride at low temperatures is even a more insoluble salt than the model benzyl-phthalazinium one, the concentration of salt in solution would remain lower than the concentration of the catalyst (like below 1:1 stoichiometries) across the reaction, and hence, a preferred 4H-activation model should be acting.

## Computational Studies

When two catalyst molecules are required to interact with the reactant, the analysis of the initial interactions and subsequent orientations to predict the selectivity becomes considerably more complex, mainly because there are in these cases increased possibilities for binding orientations and configurations. Moreover, both units of the catalyst can exhibit either cooperative or competitive behavior, even though the former could be anticipated from the NLE study. Modeling these interactions accurately often requires enhanced sampling techniques to explore a wider range of configurations and transitions that occur in the system. In this context, molecular dynamics simulations offer several significant advantages. In particular, MD simulations will track the time-dependent behavior of the molecules, allowing the observation of aggregation pathways and the dynamic formation of complexes. Also, by incorporating explicit solvents, MD simulations provide a realistic depiction of molecular interactions, which is crucial for accurately modeling how molecules aggregate.

MD simulations of two units of thiourea in chloroform or THF as a solvent showed the formation of aggregate **TH2** formed by two thiourea molecules through hydrogen bonding between the NH groups of one molecule and the carbonyl group of the other (Figure 4). The presence of other complexes is negligible. The aggregate is particularly stable in chloroform, where it holds for several hundred nanoseconds.^13^ However, the stability periods are shortened in THF, and the aggregation is broken by the solvent molecules, resulting in discrete catalyst molecules that are ready to interact with the substrate. Indeed, when the aggregate is placed in the presence of phthalazinium chloride (**PC**), it breaks into three complexes with very different populations. The predominant complex **A** features an antiparallel orientation of the two thioureas, with **PC** oriented so that the *Si face* is exposed to the nucleophilic attack. A less abundant complex **A′** also shows the antiparallel orientation of the thioureas, but **PC** is oriented so that the *Re face* is exposed to the nucleophile. Finally, the very minor complex **P** shows a parallel orientation of the thioureas in which π–π stacking between the two 4-bromo-3,5-(bis)­trifluorophenyl rings is observed. In the latter, **PC** is oriented in such a way that both prochiral faces are exposed to nucleophilic attack. In order to have accurate energy values, we optimized these structures by DFT methods at the wb97xd/6–31+G­(d,p) level of theory and calculated the energy at the wb97xd/6–311+G­(d,p)/smd = THF level of theory. As expected, complex **A** proved to be the most stable, the other antiparallel complex **A′** being 2.7 kcal/mol higher in energy, while the parallel complex **P** was 10 kcal/mol higher.

A close inspection of complexes **A** and **A′** revealed a π–Cl interaction between the chloride anion and the fused aromatic ring of the substrate, as well as a π–π stacking interaction between the 4-bromo-3,5-(bis)­trifluorophenyl ring of one unit of the catalyst and the *p*-chlorophenyl group of the intermediate. In **A**, more hydrogen-bond interactions between the carbonyl group of the catalyst and phthalazine protons are observed than in **A′**. But the most notable difference between both complexes is the π–π stacking interaction observed in **A** between the benzene ring of the phthalazine and the 4-bromo-3,5-(bis)­trifluorophenyl ring of the thiourea, not involved in the first stacking, granting it greater stability. These interactions are responsible for the recognition of the cation by the formed complex. The insertion of the phthalazinium ion occurs in such a way that one face of the diazaheterocycle is completely hindered by these interactions, while the other face is fully exposed to the nucleophile. In fact, the recognition of the chloride anion by the thiourea moieties creates a groove where the substrate cation is primarily accommodated due to electrostatic attraction and the previously mentioned π–π interactions. In the case of complex **A**, both the recognition of the chloride anion and the aromatic ring of the catalyst occur more efficiently, leading to a more stable structure (Figure 5) [for a detailed discussion, see the supporting information (SI)].

**Figure 5:**
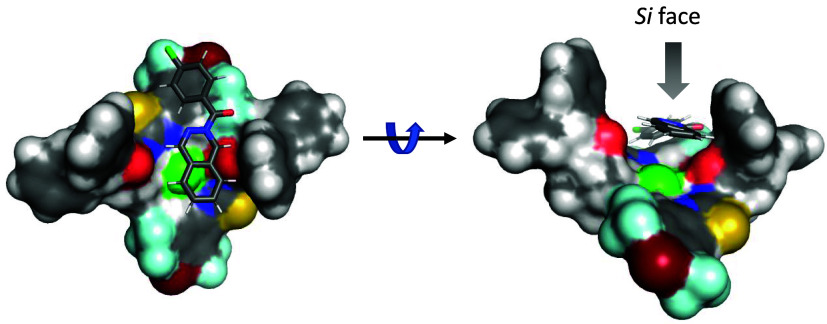
Left: Disposition of the thiourea molecules around the chloride ion shows the C_2_-symmetry in complex **A** with phthalazinium chloride. Right: Alternative view showing the groove of insertion of the ion.

Considering complexes **A**/**A′** as the starting complexes of the reaction, the catalytic cycle illustrated in Scheme 3 can be suggested.

**Scheme 3:**
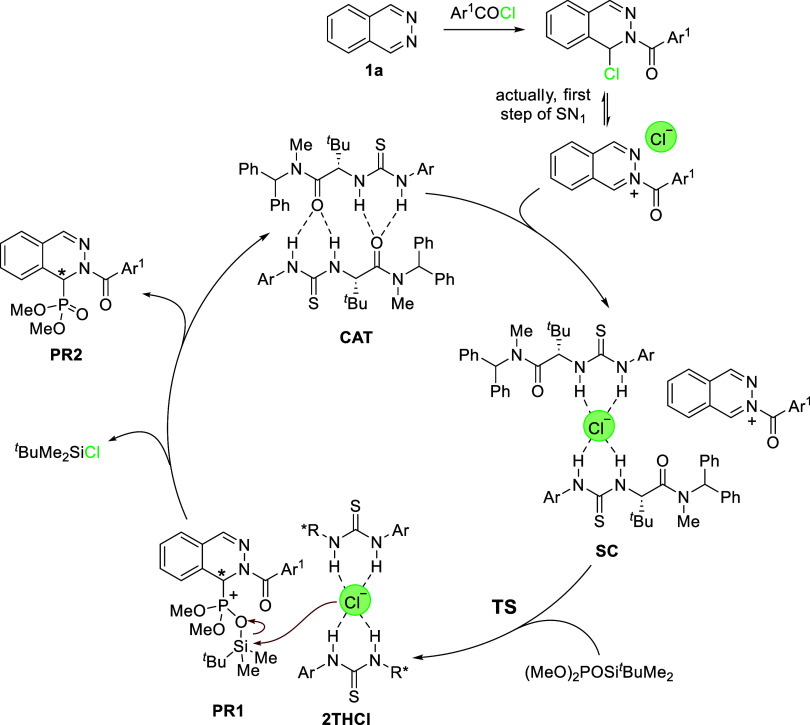
Proposed Catalytic Cycle

The process is actually a two-step process, similar to a typical SN_1_ reaction, in which acylation forms the chloroaminal-type substrate *in situ*. The first step takes place practically without a barrier to form the cationic phthalazinium intermediate, which is stable in its iminium-type form. This iminium ion enters the catalytic cycle, forming the starting complex **SC**. It might also be considered that the formation of the ionic pair **SC** is facilitated by the catalyst, which abstracts the chloride to form the *C*_2_-symmetric supramolecular complex. The nucleophilic addition of the silyl phosphite affords intermediate **PR1···2THCl**, which evolves to give **PR2** and chloro *tert*-butyldimethylsilane, while regenerating the catalyst in its aggregate form. The formation of **PR1···2THCl** is the second step in the SN_1_ reaction and represents the rate-limiting and enantiodifferentiating step of the reaction.

We located the corresponding transition structures **TS-** ***Re*** and **TS-** ***Si*** by approaching the *tert*-butyldimethylsilyl dimethyl phosphite to the phthalazinium accessible face in the corresponding complex. Aiming to explore the conformational potential energy surface of the transition structures, three staggered approaches for the nucleophile were considered.^13^ Additionally, the conformational preferences for the rest of the molecule were assumed to be those found by using MD simulations of the precursor complexes. Nevertheless, rotation of the diphenylmethyl groups of the thioureas was also considered, and no results different from those observed in MD were found. In consequence, we found the two transition structures (**TS-** ***Re*** and **TS-** ***Si***) illustrated in Figure 6. In both cases, practically the same interactions found in the starting complexes are maintained, particularly those of the π–π stacking type. As it happened in the starting complexes, the structure of the transition state, favoring attack from the *Si* face (**TS-** ***Si***) shows two interactions of the reagent, specifically between the 4-chlorophenyl and benzene ring of the phthalazine with the 4-bromo-3,5-bistrifluoromethylphenyl rings of the thioureas. In contrast, **TS-** ***Re*** shows only one π–π interaction, similar to its starting complex. On the other hand, the entry of the silyl phosphite occurs similarly, with the *tert*-butyl group oriented toward the diphenylmethyl group of the nearest thiourea, a situation that will favor the occurrence of London forces, although the mobility of the fragments does not allow for identifying a possible CH−π interaction between both groups. In any case, it seems that the additional π–π interaction with the reagent is decisive in confirming that the favored face is the *Si* face, leading to the experimentally obtained product stereochemistry.

**Figure 6:**
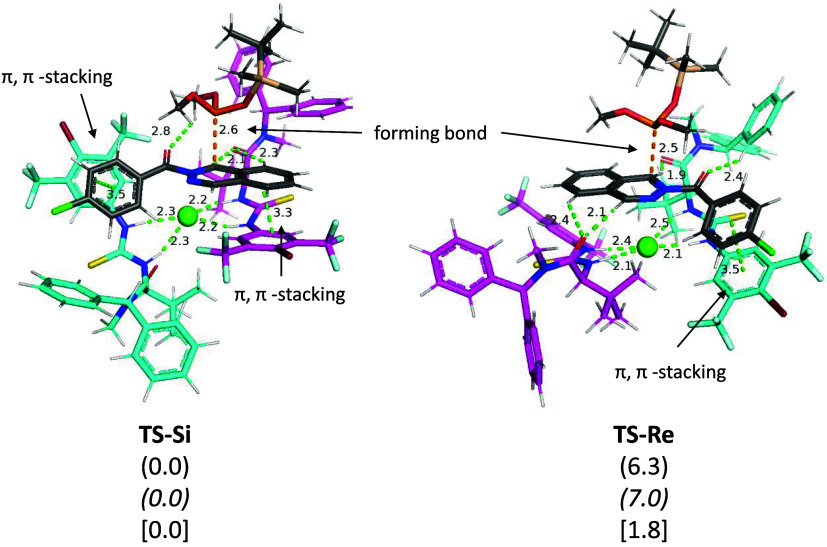
Optimized (wb97xd/def2svp) structures of **TS-** ***Re*** and **TS-** ***Si***. The two units of thiourea have been colored cyan and magenta for the sake of clarity. Relative energy values are given in kcal/mol in plain brackets for m062*x*/6–311+G­(d,p)/SMD = THF//m062*x*/6–31G­(d,p), in italic brackets for wb97xd/def2tzvp/SMD = THF// wb97xd/def2svp, and in square brackets for wb97xd/6–311+G­(d,p)/SMD = THF//wb97xd/6–31G­(d,p).

Semiquantitative NCI analysis^19^ of **TS-** ***Si*** and **TS-** ***Re*** confirmed the above-mentioned interactions (Figure 7). Additionally, in **TS-** ***Si*** London interactions are also observed between an aromatic ring of one of the diphenylmethyl groups of the thiourea and the *tert*-butyldimethylsilyl group of the phosphite, whereas similar interactions with the incoming phosphite are less evident for **TS-** ***Re***. In both cases, hydrogen interactions of the NH groups of the thiourea with the chloride ion are appreciated as blue disks, clearly indicating the strength of the bond. The semiquantitative NCI analysis confirms that there are more favorable interactions in **TS-** ***Si*** than in **TS-** ***Re***.

**Figure 7:**
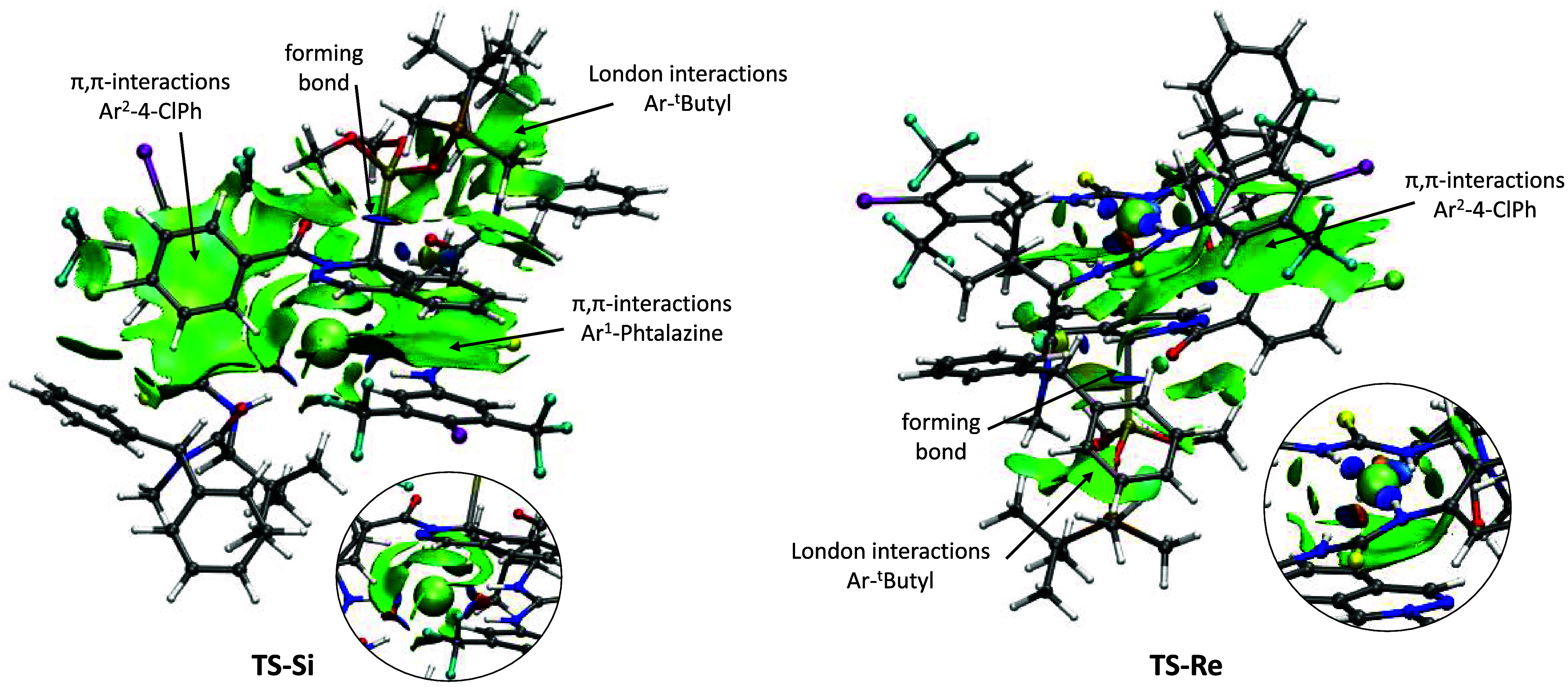
NCI analysis of **TS-** ***Si*** and **TS-** ***Re***. Circle: detail of chloride interactions. The thin, delocalized green surface indicates van der Waals interactions. Small, lenticular, and bluish surfaces indicate strong interactions such as hydrogen bonding. Steric clashes are shown as red isosurfaces.

## Substrate Scope and Product Derivatization

Next, we explored the scope and limitations of the methodology (Scheme 4). Silyl phosphites bearing diverse alkoxy groups were initially tested. Alkyl chains such as methyl, butyl, and isopropyl were well-suited, affording dihydrophthalazines (*S*)-**11aB**–**11aD** in good yields (74–83%) and good enantioselectivities (91–93% *ee*). The presence of a benzyl chain in **2E** reduces the enantioselectivity [(*S*)-**11aE**: 90%, 83% *ee*]. Finally, it was not possible to control chirality at phosphorus as nonsymmetrical silyl phosphite **2F** afforded **11aF** in excellent yield (91%) and enantioselectivity (90%), albeit as a 1.3:1 mixture of diastereoisomers.

**Scheme 4:**
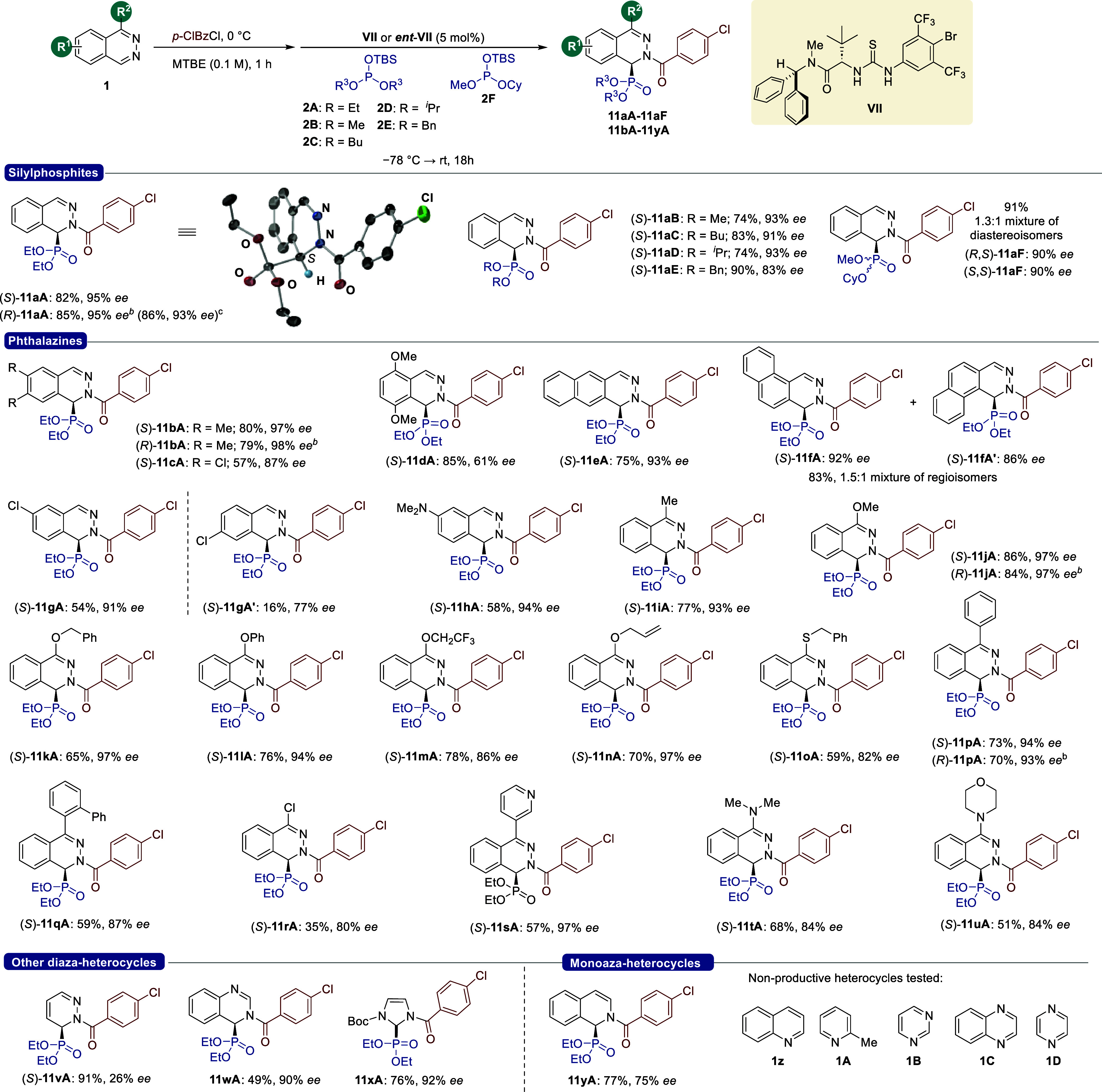
Scope^c^

b Employing catalyst *ent*-**VII**.
c Reaction performed at the 1 mmol scale.
a Reactions performed at the 0.2 mmol scale. Yields given for isolated products after chromatography. *ee*’s were determined by HPLC on chiral stationary phases.

Next, we carried out a phosphonylation reaction of other phthalazines employing **2A** as a representative silyl phosphite. The substitution in the most distant positions from the reactive center was generally well-tolerated: 6,7-dimethyl 1,2-dihydrophthalazine (*S*)-**11bA** was obtained in 80% yield and 97% *ee*, while the 6,7-dichloro derivative (*S*)-**11cA** was isolated in 57% yield and 87% *ee*. The presence of substituents near the reactive center, such as in 5,8-dimethoxyphthalazine (**1d**), had a negative impact on the enantioselectivity, affording (*S*)-**11dA** in 85% yield and 61% *ee*. Additionally, the stereochemical impact of additional fused rings on the substrate was studied. Benzo­[*g*]­phthalazine (**1e**) afforded (*S*)-**11eA** in 75% yield and 93% *ee*, while benzo­[*f*]­phthalazine (**1f**) gave an inseparable mixture of regioisomers **11fA/11fA′** (1.5:1) in good yield and *ee* values (92 and 86% *ee*, respectively). Noticeably, regioselective processes were observed when more challenging C6-monosubstituted phthalazines were employed. For example, 6-chlorophthalazine (**1g**) yielded a separable mixture of regioisomers (*S*)-**11gA** (54%, 91% *ee*) and (*S*)-**11gA′** (16%, 77% *ee*) in a 3.4:1 ratio, while phthalazine **1h**, bearing a strongly donating dimethylamino group, provided (*S*)-**11hA** (58%, 94% *ee*) as a single regioisomer. Next, the methodology was applied to C1-monosubstituted phthalazines. Adducts (*S*)-**11iA**–**11qA**, bearing alkyl, diverse alkoxy, aryl, and thioether groups, were isolated regularly in good yields (59–86%) and good-to-excellent enantioselectivities (82–97% *ee*). Unfortunately, phthalazines containing electron-withdrawing groups at this position, such as **1r**, exhibited lower reactivity, affording (*S*)-**11rA** in 35% yield and 80% *ee*. Other challenging substrates, containing nitrogen functional groups prone to be acylated, such as **1s** (pyridine fragment), **1t** (dimethylamino group), and **1u (**morpholine), were well-tolerated. To further evaluate the synthetic value of the methodology, a model reaction was performed on a 1 mmol scale to afford (*R*)-**11aA** without significant loss in yield or enantioselectivity. Finally, other diazaheterocycles were evaluated. Pyridazine (**1v**) led to the expected adduct **11vA** in lower enantioselectivity (26% *ee*), according to the stereochemical model (*vide supra*, Figures 6 and 7). Compared to phthalazine substrates, this heterocycle lacks the condensed aromatic ring and, consequently, the π–π interactions with the catalytic system, which is a key element for stereocontrol according to the calculations discussed above. Additionally, the competing background reaction facilitated by a higher solubility of pyridazinium chloride may also contribute to the lower enantioselectivity observed in this case.^20^ Interestingly, some 1,3-diazaheterocycles were also suitable substrates for the reaction. Quinazoline (**1w**) provided **11wA** as a single regioisomer in moderate yield (49%) and high 90% *ee*, while imidazole derivative **1x** afforded the desired product **11xA** in good yield (76%) and 92% *ee*. The catalytic system was also tested for the dearomatization of monoazaheterocycles. Isoquinoline (**1y**), overcoming also a background reaction (58% NMR yield in the absence of the catalyst), afforded adduct **11yA** in good yield (77%), albeit in moderate 75% *ee*. Finally, other heterocycles, such as quinoline (**1z**), 2-picoline (**1A**), pyrimidine (**1B**), quinoxaline (**1C**), and pyrazine (**1D**), were unproductive substrates for this transformation. Crystals of (*S*)-**11aA** suitable for X-ray diffraction analysis were used to assign its absolute *S* configuration, which is in agreement with the stereochemical model discussed above. Assuming a uniform reaction pathway, the absolute configuration of other products (*S*)-**11** was assigned by analogy.

The possibility of employing other nonsilylated nucleophiles was next explored (Scheme 5A). Under optimal conditions, diethyl phosphonate (**2G**) provided low reactivity (<10%) and enantioselectivity (11% *ee*). To our delight, the use of trialkylphosphites **2H** and **2I** afforded dihydrophthalazines (*S*)-**11aA** and (*S*)-**11aD**, maintaining excellent yields and without significant erosion of the enantiomeric excess (92–93% *ee*). These results highlight the practical utility of the methodology, which was adaptable to simple, commercially available phosphorus reagents. Also, it generates gaseous or volatile byproducts that are easier to remove. Finally, the synthetic usefulness of dihydrophthalazines **11** was demonstrated by accessing some selected targets (Scheme 5B). Subjecting (*S*)-**11aA** to standard hydrogenation [Pd­(C), 1 atm (H_2_)] of the C=N double bond afforded cyclic α-hydrazino phosphonate (*S*)-**16** in 71% yield and 95% *ee*. Under identical conditions, (*S*)-**11kA** was efficiently transformed into phthalazone derivative (*S*)-**17**, which was isolated in 69% yield and 96% *ee*. Finally, phosphonic acid (*R*)-**18** was easily obtained by treatment of (*R*)-**11aA** with TMSBr, also without racemization.

**Scheme 5:**
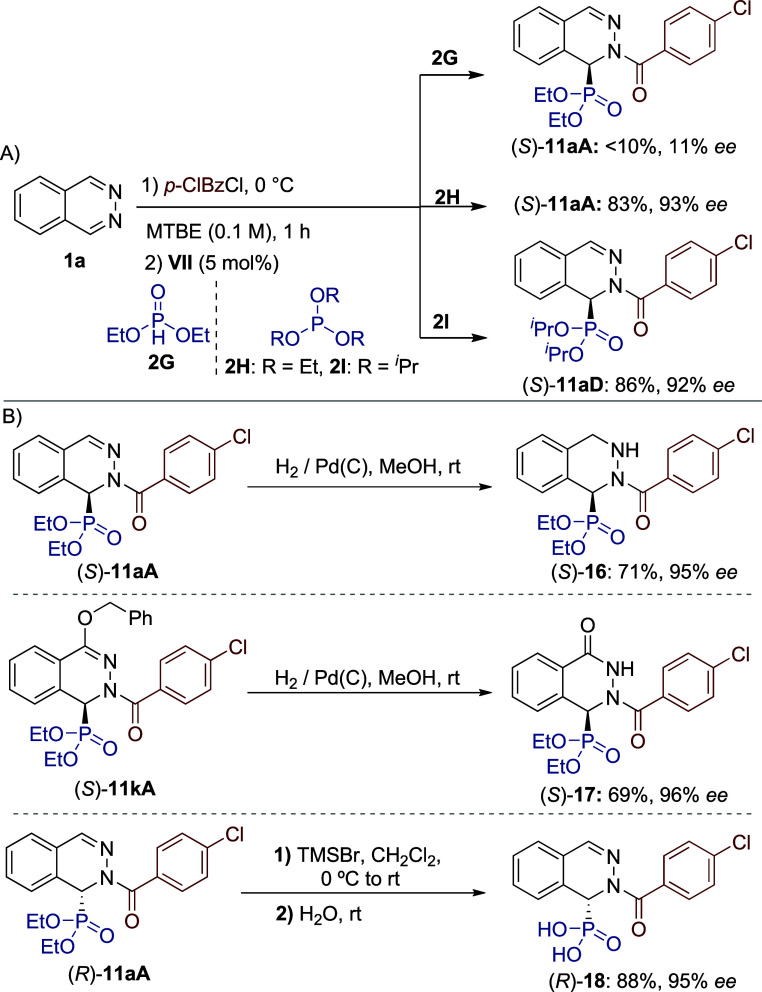
(A) Reaction with Other Phosphorus Nucleophiles. (B) Transformations of Dihydrophthalazines **11**

## Conclusions

In summary, a practical methodology for the enantioselective dearomatization of diazaheterocycles employing silylated and nonsilylated phosphorus nucleophiles has been developed. The process involves the molecular recognition of *N*-acyliminium chlorides by a *tert*-leucine-derived thiourea catalyst, yielding appealing cyclic hydrazino phosphonates in good yields with excellent enantioselectivities. NMR and MS studies suggest that the low solubility of these salts might favor the formation of a supramolecular thiourea–chloride–iminium 2:1:1 complex. Assisted by MD simulations and DFT calculations, a high-order supramolecular complex, featuring *C*_2_-symmetry around the chloride, was proposed as the catalytically relevant species, explaining the nonlinear effect observed experimentally. Additionally, a stereochemical model was proposed, further supported by a quantitative analysis of the noncovalent interactions.

## Methods

### General Information

Unless otherwise noted, commercially available reagents were used without further purification. Solvents for catalytic reactions (MTBE, Et_2_O, THF, and toluene) were distilled and dried over Na/benzophenone at 760 Torr. ^1^H NMR spectra were recorded at 300 or 500 MHz (internal reference; CDCl_3_ = 7.26 ppm; CD_2_Cl_2_ = 5.32 ppm; acetone-*d*_6_ = 2.05 ppm; DMSO-*d*_6_ = 2.50 ppm). ^13^C NMR spectra were recorded at 75.5 or 126 MHz (internal reference; CDCl_3_ = 77.16 ppm; CD_2_Cl_2_ = 54.00 ppm; acetone-*d*_6_ = 29.84 ppm; DMSO-*d*_6_ = 39.52 ppm). ^31^P NMR spectra were recorded at 122 MHz.^19^F NMR spectra were recorded at 471 MHz. Column chromatography was performed on silica gel (Merck Kieselgel 40–60). Analytical TLC was performed on aluminum-backed plates (1.5 cm × 5 cm) precoated (0.25 mm) with silica gel (Merck, Silica Gel 60 F_254_). Semipreparative TLC was performed on glass-backed plates (5 cm × 10 cm) precoated (0.25 mm) with silica gel (Merck, Silica Gel 60 F_254_). Compounds were visualized by exposure to ultraviolet (UV) light and/or by dipping the plates in solutions of ninhydrin, vanillin, or phosphomolybdic acid stains, followed by heating. The melting point of crystalline solid (*S*)-**11aA** was recorded in a metal block and is uncorrected. Optical rotations were measured on a JASCO P-2000 polarimeter. The enantiomeric excess (*ee*) of the products was determined by chiral stationary phase HPLC columns (Daicel Chiralpack IA, IB, IC, and ID). High-resolution mass spectrometry (HRMS) was performed by using a Thermo Fisher Orbitrap Elite with an Orbitrap mass analyzer.

### General Procedure for the Asymmetric Dearomatization Reaction of Phthalazines **1a**-**u**

In a flame-dried Schlenk flask, *p*-chlorobenzoyl chloride (26 μL, 0.2 mmol) was added to a solution of the corresponding diazarene derivative **1a**–**u** (0.2 mmol) in freshly distilled anhydrous MTBE (2 mL, 0.1 M) at 0 °C. The resulting suspension was stirred for 1 h at room temperature. Then, catalyst **VII** (7 mg, 0.01 mmol, 5 mol %) was added, and the reaction was cooled to −78 °C (dry ice/acetone bath). *Tert*-butyldimethylsilyl phosphite **2A**–**F** (0.22 mmol) was added, and the reaction mixture was stirred for 18 h and allowed to warm slowly to room temperature during that time (−78 → −75 °C, reaction time: 4 h; −75 → −50 °C, 5 h; −50 → −25 °C, 6 h; −25 → 0 °C, 8 h; 0 → 10 °C, 10 h; 10 → 20 °C, 18 h). Then, the solvent was removed under reduced pressure, and the residue was purified by flash chromatography to afford the corresponding products (*S*)-**11aA**–**11uA**. Enantiomeric ratios were determined by HPLC analysis. *Racemic samples* were prepared without a catalyst following the general procedure described above.

## Supplementary Material





## Notes

Experimental procedures; material and methods; catalyst screening; characterization data for new compounds; HPLC traces; NMR titration studies, molecular dynamics (MD); and detailed DFT calculations (PDF)
Crystallographic data for (*S*)-**11aA** (CIF)
