# Supplementary material for: Harnessing Noncovalent π‑Type Interactions in Thiourea–Chloride Supramolecular Complexes: Toward the Asymmetric Dearomatization of Diazaheterocycles
Source: ACS Catal. 2025 Aug 6;15(16):14443–55. doi: 10.1021/acscatal.5c04438 (PMC12363210; doi:10.1021/acscatal.5c04438)
Supplement: Supplementary file 1 [file cs5c04438_si_001.pdf]

Supporting Information

Harnessing Noncovalent π-Type Interactions in Thiourea-Chloride Supramolecular Complexes: Toward the Asymmetric Dearomatization of Diazaheterocycles

Marta Velázquez,[a] Pilar Elías-Rodríguez,[a] Tomás Tejero,[b] Rosario Fernández,[a,*] Pedro Merino,[c,*] José M. Lassaletta[d,*] and David Monge[a,*]

[a]Departamento de Química Orgánica, Facultad de Química, Universidad de Sevilla and Centro de Innovación en Química Avanzada (ORFEO-CINQA), C/ Prof. García González, 1, 41012 Sevilla, Spain. Email: ffernan@us.es, dmonge@us.es

[b]Instituto de Síntesis Química y Catálisis Homogénea (ISQCH), Universidad de Zaragoza-CSIC, 50009 Zaragoza, Spain.

[c]Instituto de Biocomputación y Física de Sistemas Complejos (BIFI), Universidad de Zaragoza, 50009 Zaragoza, Spain. Email: pmerino@unizar.es

[d]Instituto de Investigaciones Químicas (CSIC-US) and Centro de Innovación en Química Avanzada (ORFEO-CINQA), Avda. Américo Vespucio, 49, 41092 Sevilla, Spain. Email: jmlassa@iiq.csic.es

Table of content

1. General information S3
2. General procedure for the synthesis of phthalazines **1m-o** S4
3. Synthesis of 1-([1,1'-biphenyl]-2-yl)phthalazine **1q** S5
4. Synthesis of 2-benzylphthalazin-2-ium chloride (***Pht*-1**) S5
5. Synthesis of organocatalysts. S6
6. Synthesis of silylphosphites **2A-F** S10
7. Preliminary experiments S12
8. Preliminary screening of chiral organocatalysts. S13
9. Solvent optimization S15
10. Optimization of the acylating reagent S16
11. General procedure for the optimization of the acylating reagents S17
12. Kinetic profile S28
13. General procedure for the asymmetric dearomatization reaction of benzodiazines derivatives **1** S29
14. Derivatizations S52

14.1. Synthesis of diethyl (*S*)-[2-(4-chlorobenzoyl)-1,2,3,4-tetrahydrophthalazin-1-yl]phosphonate [(*S*)-**16**] S52

14.2. Synthesis of diethyl (*S*)-[2-(4-chlorobenzoyl)-4-oxo-1,2,3,4-tetrahydrophthalazin-1-yl]phosphonate [(*S*)-**17**]. S53

14.3. Synthesis of (*R*)-[2-(4-chlorobenzoyl)-1,2-dihydrophthalazin-1-yl]phosphonic acid [(*R*)-**18**]. S54

15. Analysis of the reaction mixture by mass spectrometry S55
16. Non-linear effect experiment S56
17. ^{1}H-NMR titration experiments S58
18. Data analysis for binding model determination S60
19. Characterization of complexes S62

19.1. Complex **VII:*Pht*-1 (2:1)** S62

19.1.1. Bidimensional NOESY and ROESY experiments S64
19.2. Complex **VII:*Pht*-1 (1:1)** S67
19.2.1. Bidimensional ROESY experiments S69
20. Computational studies. S72
20.1 . Computational methods. S72
20.1.1. Molecular dynamics methods S72
20.1.2. QM methods S72
20.2. Molecular dynamics studies on aggregation of catalyst and formation of complexes with the phthalazinium chloride S73
20.3. DFT Studies S76
20.3.1. Achiral reaction S76
20.3.2. Catalyzed reaction S76
20.3.3. Energies S78
20.4. Cartesian coordinates S80
21. NMR spectra of new compounds S99
22. References S168

1. General information.

^{1}H-NMR spectra were recorded at 300 MHz or 500 MHz (internal reference; $CDCl_3$ = 7.26 ppm; CD_2Cl_2 = 5.32 ppm; acetone-d_6 = 2.05 ppm; DMSO-d_6 = 2.50 ppm); ^{13}C-NMR spectra were recorded at 75.5 MHz or 126 MHz (internal reference; $CDCl_3$ = 77.16 ppm; CD_2Cl_2 = 54.00 ppm; acetone-d_6 = 29.84 ppm; DMSO-d_6 = 39.52 ppm). ^{31}P-NMR spectra were recorded at 122 MHz. ^{19}F-NMR spectra were recorded at 471 MHz. Multiplicities were given as: s (singlet), br s (broad singlet), d (doublet), t (triplet), q (quartet), dd (doublet of doublets), and m (multiplet). Column chromatography was performed on silica gel (Merck Kieselgel 40-60). Analytical TLC was performed on aluminum-backed plates (1.5 × 5 cm) pre-coated (0.25 mm) with silica gel (Merck, Silica Gel 60 F_{254}). Semipreparative TLC was performed on glass-backed plates (5 x 10 cm) pre-coated (0.25 mm) with silica gel (Merck, Silica Gel 60 F_{254}). Compounds were visualized by exposure to UV light and/or by dipping the plates in solutions of ninhydrin, vanillin, or phosphomolybdic acid stains, followed by heating. The melting point of crystalline solid (*S*)-**11aA** was recorded in a metal block and is uncorrected. Optical rotations were measured on a JASCO P-2000 polarimeter. The enantiomeric excess (ee) of the products was determined by chiral stationary phase HPLC (Daicel Chiralpack IA, IB, IC, ID). High-resolution mass spectrometry (HRMS) was performed using a Thermo Fisher Orbitrap Elite with an orbitrap mass analyzer. Unless otherwise noted, commercially available reagents were used without further purification. Solvents for catalytic reactions (MTBE, Et_2O, THF, and toluene) were distilled and dried over Na/benzophenone at 760 Torr. Phthalazine derivatives **1b**,[1] **1c**[1], **1d**,[2] **1e**,[1] **1f**,[2] **1g**,[2] **1h**,[2] **1i**,[3] **1j**,[4] **1k**,[4] **1l**,[5] **1p**,[2] **1t**,[6] **1r**,[6] **1s**,[7] and **1u**[6]; silyl phosphites **2A**,[8] **2D**,[8] and organocatalysts **O1**,[9] **O2**,[10] **O3**,[11] **I**,[12] **II**,[12] **III**,[4] **IV**,[13] **V**,[14] **VI**,[15] **VII**,[13] **VIII**,[15] **IX**,[16] and **X**[13] were synthesized according to literature procedures.

2. General procedure for the synthesis of phthalazines 1m-o.

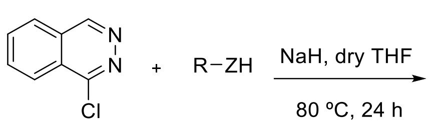

P1 **S1-3** **1m-o**

In a flame-dried Schlenk flask, the corresponding alcohol (**S1-2**) or thiol (**S3**) (15 mmol) was added dropwise to a mixture of NaH (480 mg, 6 mmol) in dry THF (18 mL). The mixture was stirred for 30 min at room temperature. Then, 1-chlorophthalazine (**P1**) (500 mg, 3 mmol) was added, and the corresponding mixture was stirred at 80 °C for 24 h. After that time, the reaction was cooled to room temperature, and EtOAc (20 mL) and H_2O (20 mL) were added. The organic layer was washed with NaCl (3 x 20 mL), dried over $MgSO_4$, and the solvent was removed under reduced pressure. The resulting residue was purified by flash chromatography to afford **1m**, **1n**, and **1o**.

1-(2,2,2-Trifluoroethoxy)phthalazine (1m): Following the general procedure **2**, using 2,2,-trifluoroethanol (**S1**) (1.1 mL, 15 mmol), **1m** was obtained after purification by flash chromatography (cyhex/Acetone 1/1) as a yellow solid (220 mg, 32%). ^{1}**H-NMR** (300 MHz, $CDCl_3$): δ 9.23 (s, 1H), 8.28 – 8.23 (m, 1H), 7.94 – 7.89 (m, 3H), 5.08 (q, J = 8.4 Hz, 2H). ^{13}**C-NMR** (75.5 MHz, $CDCl_3$): δ 159.0, 149.4, 133.0, 132.8, 129.2, 126.1, 123.6 (q, $J_{C,F}$ = 277.1 Hz), 122.9, 119.4, 63.6 (q, $J_{C,F}$ = 36.5 Hz). ^{19}**F-NMR** (471 MHz, $CDCl_3$): δ −73.71 (s, 3F). **HRMS** (ESI): *m/z* calcd for $C_{10}H_8ON_2F_3$ [M^++H] 229.0583, found 229.0579.

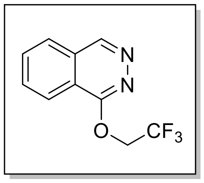

1-(Allyloxy)phthalazine (1n): Following the general procedure **2**, using allylic alcohol (**S2**) (1 mL, 15 mmol), **1n** was obtained after purification by flash chromatography (cyhex/Acetone 3/1) as a yellow solid (270 mg, 48%). ^{1}**H-NMR** (300 MHz, $CDCl_3$): δ 9.14 (s, 1H), 8.24 – 8.18 (m, 1H), 7.85 – 7.79 (m, 3H), 6.28 – 6.15 (m, 1H), 5.53 – 5.46 (m, 1H), 5.34 – 5.29 (m, 1H), 5.18 – 5.15 (m, 2H). ^{13}**C-NMR** (75.5 MHz, $CDCl_3$): δ 160.0, 148.1, 133.0, 132.3, 132.1, 128.9, 125.8, 123.0, 119.9, 118.2, 68.0. **HRMS** (ESI): *m/z* calcd for $C_{11}H_{11}ON_2$ [M^++H] 187.0866, found 187.0862.

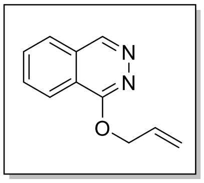

1-(Benzylthio)phthalazine (1o): Following the general procedure **2**, using benzyl mercaptan (**S3**) (1.7 mL, 15 mmol), **1o** was obtained after purification by flash chromatography (cyhex/Acetone 2/1) as a yellow solid (418 mg, 55%). ^{1}**H-NMR** (300 MHz, $CDCl_3$): δ 9.24 (s, 1H), 8.07 – 8.03 (m, 1H), 7.84 – 7.78 (m, 3H), 7.54 – 7.51 (m, 2H), 7.35 – 7.25 (m, 3H), 4.77 (s, 2H). ^{13}**C-NMR** (75.5 MHz, $CDCl_3$): δ 160.1, 149.0, 137.1, 132.52, 132.46, 129.5, 128.7, 127.5, 126.8, 125.5, 123.5, 34.4. **HRMS** (ESI): *m/z* calcd for $C_{15}H_{13}N_2S$ [M^++H] 253.0794, found 253.0790.

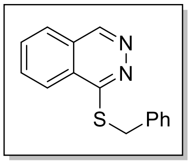

3. Synthesis of 1-([1,1'-biphenyl]-2-yl)phthalazine 1q.

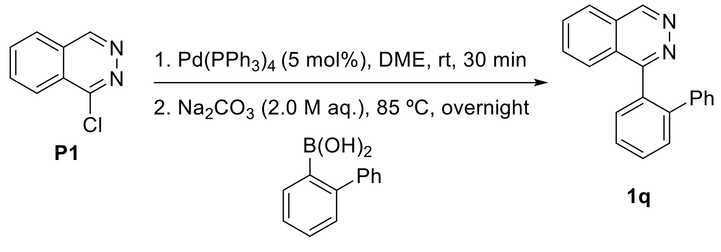

A flame-dried Schlenk flask was charged with $Pd(PPh_3)_4$ (117 mg, 0.1 mmol) and 1-chlorophthalazine (**P1**) (329 mg, 2 mmol). After three cycles of vacuum-argon, dry DME (3 mL) was added, and the reaction mixture was stirred for 30 min at room temperature. Then, [1,1'-biphenyl]-2-ylboronic acid (**S4**) (500 mg, 2.4 mmol) and Na_2CO_3 (2.0 M, 2.4 mL) were added, and the reaction mixture was stirred at 85 °C in an oil bath overnight. After that time, the reaction was cooled to room temperature, and H_2O (5 mL) was added. The aqueous layer was extracted with DCM (3 x 10 mL). The combined organic layers were washed with NaCl (1 x 10 mL), dried over $MgSO_4$, and the solvent was removed under reduced pressure. The resulting residue was purified by flash chromatography (cyhex/Acetone 2/1) to afford **1q** as a brown solid (264 mg, 47%). **^{1}H-NMR** (300 MHz, $CDCl_3$): δ 9.43 (s, 1H), 7.84 – 7.81 (m, 1H), 7.72 – 7.66 (m, 1H), 7.65 – 7.51 (m, 5H), 7.47 – 7.44 (m, 1H), 7.13 – 7.10 (m, 2H), 7.02 – 6.93 (m, 3H). **^{13}C-NMR** (75.5 MHz, $CDCl_3$): δ 161.1, 150.7, 141.9, 140.6, 135.0, 132.1, 132.0, 131.0, 130.1, 129.7, 129.3, 128.0, 127.7, 126.9, 126.3, 126.2, 125.8. **HRMS** (ESI): *m/z* calcd for $C_{20}H_{15}N_2$ [M^++H] 283.1230, found 283.1228.

4. Synthesis of 2-benzylphthalazin-2-ium chloride (*Pht*-1).

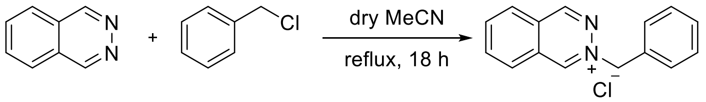

In a flame-dried Schlenk flask, benzyl chloride (**S5**) (140 µL, 1.2 mmol) was added to a solution of phthalazine (**1a**) (133 mg, 1 mmol) in dry MeCN (1.5 mL). The mixture was refluxed for 18 h. After that time, the reaction was cooled to room temperature, and the precipitate was filtered, washed with Et_2O, and dried in *vacuo* to give ***Pht*-1** as a pale orange solid (231 mg, 90%). **^{1}H-NMR** (300 MHz, CD_2Cl_2): δ 12.83 (s, 1H), 9.81 (s, 1H), 8.93 (d, *J* = 8.1 Hz, 1H), 8.47 – 8.44 (m, 1H), 8.38 – 8.32 (m, 1H), 8.28 – 8.23 (m, 1H), 7.83 – 7.77 (m, 2H), 7.40 – 7.33 (m, 3H), 6.31 (m, 2H). **^{13}C-NMR** (75.5 MHz, CD_2Cl_2): δ 155.0, 153.1, 139.8, 136.7, 133.6, 132.2, 130.6, 130.2, 129.6, 128.9, 128.5, 128.2, 67.4. **HRMS** (ESI): *m/z* calcd for $C_{15}H_{13}N_2$ [M^+] 221.1073, found 221.1070.

5. Synthesis of organocatalysts.

Synthesis of organocatalyst *ent*-VII

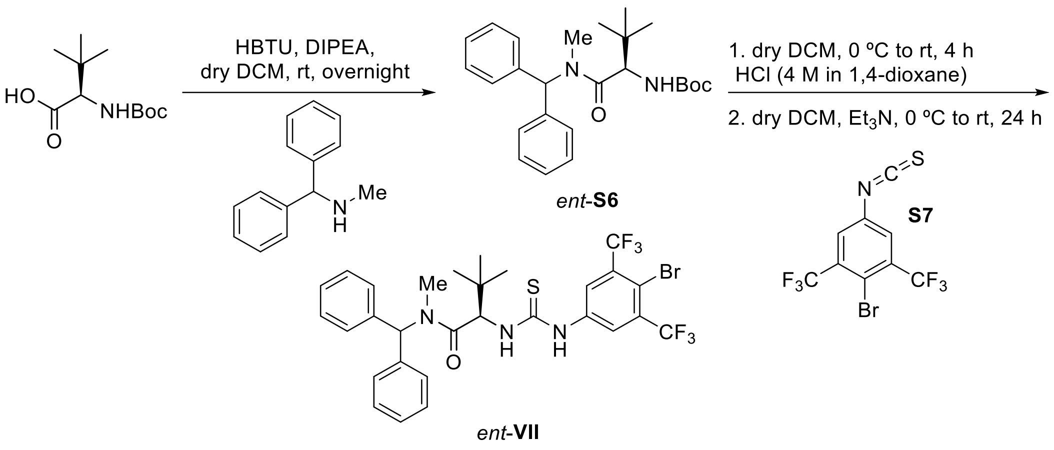

Synthesis of *ent*-S6. DIPEA (1.1 mL, 5.94 mmol) and *N*-methyl-1,1-diphenylmethanamine (1.1 g, 5.45 mmol) were subsequently added to a suspension of HBTU (2.1 g, 5.45 mmol) and *N*-Boc-D-*tert*-Leucine (1.1 g, 4.95 mmol) in dry DCM (40 mL) at room temperature. The reaction was stirred overnight. After this time, the reaction was diluted with Et_2O (100 mL) and washed with aq. HCl (1.0 M, 2 x 100 mL), saturated aqueous $NaHCO_3$ (2 x 100 mL), and NaCl (2 x 100 mL). The organic layer was dried over anhydrous $MgSO_4$, filtered and concentrated under reduced pressure. The resulting residue was purified by flash chromatography (*n*-hexane/EtOAc 4/1) to afford *tert*-butyl (*R*)-{1-[benzhydryl(methyl)amino]-3,3-dimethyl-1-oxobutan-2-yl}carbamate (*ent*-**S6**) as a white solid (1.1 g, 53%). The spectroscopic data match those reported in the literature.[17] **^{1}H-NMR** (300 MHz, $CDCl_3$): The compound exists as a ~5:1 mixture of carbamate rotamers. *Signals corresponding to the major rotamer:* δ 7.37 – 7.27 (m, 6H), 7.24 – 7.14 (m, 5H), 5.27 (d, *J* = 9.9 Hz, 1H), 4.57 (d, *J* = 10.1 Hz, 1H), 2.97 (s, 3H), 1.46 (s, 9H), 1.03 (s, 9H). *Representative signals corresponding to the minor rotamer:* δ 4.71 (d, *J* = 10.1 Hz, 1H), 2.73 (s, 3H), 1.44 (s, 9H), 0.93 (s, 9H).

Synthesis of *ent*-VII. **Step 1**: HCl (4.0 M in 1,4-dioxane, 2.6 mL, 10 mmol) was dropwise added to a solution of *tert*-butyl (*R*)-{1-[benzhydryl(methyl)amino]-3,3-dimethyl-1-oxobutan-2-yl}carbamate (*ent*-**S6**) (411 mg, 1 mmol) in dry DCM (2.6 mL) under argon atmosphere at 0 °C. The reaction mixture was allowed to warm to room temperature and stirred for 4 h. After this time, the solvent was removed under reduced pressure to afford (*R*)-2-amino-*N*-benzhydryl-*N*,3,3-trimethylbutanamide hydrochloride as a white solid, which was used in the next step without further purification.

Step 2: Et_3N (282 µL, 2 mmol) was added to a solution of (*R*)-2-amino-*N*-benzhydryl-*N*,3,3-trimethylbutanamide hydrochloride (~1 mmol) in dry DCM (5.1 mL) at 0 °C. After 15 min, 2-bromo-5-isothiocyanato-1,3-bis(trifluoromethyl)benzene (**S7**)[18] (385 mg, 1.1 mmol) was added, and the reaction mixture was allowed to warm to room temperature and stirred for 24 h. Then, the solvent was removed under reduced pressure, and the residue was purified by flash chromatography (*n*-hexane/EtOAc 7/1) to afford *ent*-**VII** as a white solid (601 mg, 91% in 2 steps). The spectroscopic data match those reported in the literature.[13] **^{1}H-NMR** (300 MHz, CD_2Cl_2): δ 8.21 (d, *J* = 9.1 Hz, 1H), 8.03 (s, 1H), 7.64 (d, 2H), 7.38 – 7.31 (m, 3H), 7.15 – 7.05 (m, 5H), 6.97 – 6.87 (m, 3H), 5.64 (d, *J* = 9.1 Hz, 1H), 3.13 (s, 3H), 1.16 (s, 9H). **^{13}C-NMR** (126 MHz, CD_2Cl_2): δ 183.1, 175.1, 139.4, 138.9, 138.0, 132.7 (q, *J* = 31.3 Hz), 130.2, 129.5 – 129.4 (m), 129.2, 128.9, 128.6, 128.0, 127.6, 123.0 (q, *J* = 274.4 Hz), 114.8, 63.2, 62.9, 36.9, 34.6, 27.9. **$[\alpha]_D^{23}$ =** +81.1 (*c* 1, $CHCl_3$).

Synthesis of organocatalyst XI

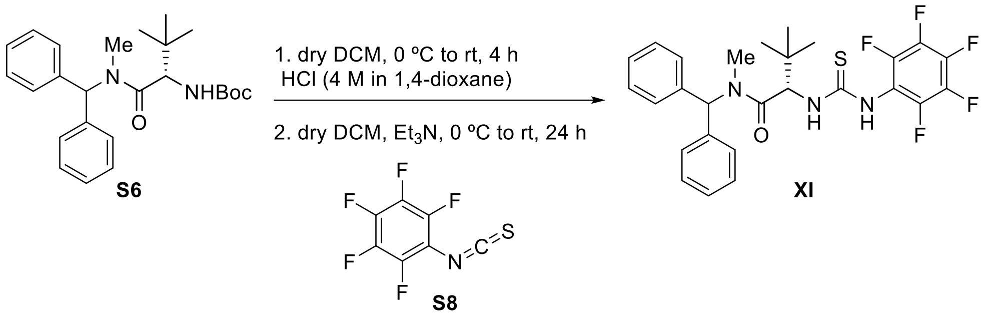

Synthesis of XI. **Step 1**: HCl (4.0 M in 1,4-dioxane, 1.3 mL, 5 mmol) was dropwise added to a solution of *tert*-butyl (*S*)-{1-[benzhydryl(methyl)amino]-3,3-dimethyl-1-oxobutan-2-yl}carbamate (**S6**)[17] (205 mg, 0.5 mmol) in dry DCM (1.3 mL) under argon atmosphere at 0 °C. The reaction mixture was allowed to warm to room temperature and stirred for 4 h. After this time, the solvent was removed under reduced pressure to afford (*S*)-2-amino-*N*-benzhydryl-*N*,3,3-trimethylbutanamide hydrochloride as a white solid, which was used in the next step without further purification.

Step 2: Et_3N (141 µL, 1 mmol) was added to a solution of (*S*)-2-amino-*N*-benzhydryl-*N*,3,3-trimethylbutanamide hydrochloride (~0.5 mmol) in dry DCM (2.55 mL) at 0 °C. After 15 min, 1,2,3,4,5-pentafluoro-6-isothiocyanatobenzene (**S8**)[19] (124 mg, ~0.55 mmol) was added, and the reaction mixture was allowed to warm to room temperature and stirred for 24 h. Then, the solvent was removed under reduced pressure, and the residue was purified by flash chromatography (*n*-hexane/EtOAc 6/1) to afford **XI** as a beige solid (161 mg, 60% in 2 steps). **$[\alpha]_D^{23}$ =** –96.5 (*c* 1, $CHCl_3$). **^{1}H-NMR** (300 MHz, $CDCl_3$): The compound exists as a ~9.6:1 mixture of carbamate rotamers. *Signals corresponding to the major rotamer:* δ 8.21 (s, 1H), 8.14 (d, *J* = 9.7 Hz, 1H), 7.39 – 7.33 (m, 3H), 7.23 – 7.20 (m, 2H), 7.14 – 7.04 (m, 4H), 6.95 (d, *J* = 7.6 Hz, 1H), 5.87 (d, *J* = 9.7 Hz, 1H), 3.02 (s, 3H), 1.12 (s, 9H). *Signals corresponding to the minor rotamer:* δ 8.47 (s, 1H), 8.04 (d, *J* = 9.5 Hz, 1H), 6.09 (d, *J* = 9.5 Hz, 1H), 2.60 (s, 3H), 0.97 (s, 9H). **^{13}C-NMR** (126 MHz, $CDCl_3$): *Signals corresponding to both rotamers*: δ 185.0, 174.7, 145.5 – 145.4 (m), 143.5 – 143.4 (m), 141.8 – 141.6 (m), 139.8 – 139.6 (m), 138.4, 136.7 – 136.5 (m), 136.0, 130.2, 129.6, 128.9, 128.7, 128.6, 128.4, 127.94, 127.88, 127.1, 126.9, 114.3 – 114.1 (m), 62.3, 61.7, 37.04, 36.97, 33.9, 27.43, 27.35. **^{19}F-NMR** (471 MHz, $CDCl_3$): δ −163.51 (s, 2F), −156.78 (s, 1F), −144.67 (s, 2F). **HRMS** (ESI): *m/z* calcd for $C_{27}H_{26}ON_3F_5NaS$ $[M^++Na]$ 558.1609, found 558.1596.

Synthesis of organocatalyst XII

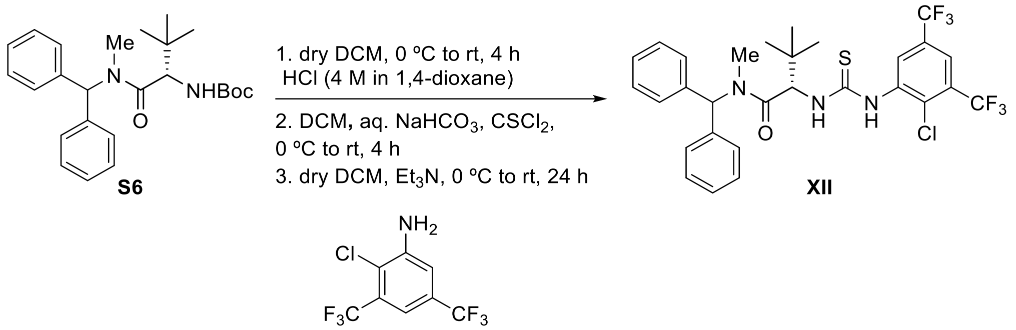

Synthesis of XII. **Step 1**: HCl (4.0 M in 1,4-dioxane, 1.3 mL, 5 mmol) was dropwise added to a solution of *tert*-butyl (*S*)-{1-[benzhydryl(methyl)amino]-3,3-dimethyl-1-oxobutan-2-yl}carbamate (**S6**)[17] (205 mg, 0.5 mmol) in dry DCM (1.3 mL) under argon atmosphere at 0 °C. The reaction mixture was allowed to warm to room temperature and stirred for 4 h. After this time, the solvent was removed under reduced pressure to afford (*S*)-2-amino-*N*-benzhydryl-*N*,3,3-trimethylbutanamide hydrochloride as a white solid, which was used in the next step without further purification.

Step 2: Saturated aqueous $NaHCO_3$ (4.2 mL) was added to a solution of 3,5-bis(trifluoromethyl)-2-chloroaniline (~0.55 mmol) in DCM (3.8 mL) and EtOAc (0.4 mL) at 0 ºC. The biphasic mixture was stirred for 15 min, and thiophosgene (150 µL, 1.65 mmol) was added to the organic layer. The reaction mixture was allowed to warm to room temperature and stirred for 24 h. After this time, the mixture was diluted with H_2O (10 mL) and extracted with DCM (3 x 15 mL). The combined organic layers were washed with H_2O (1 x 30 mL) and NaCl (1 x 30 mL), dried over $MgSO_4$, and concentrated under reduced pressure to afford 2-chloro-1-isothiocyanato-3,5-bis(trifluoromethyl)benzene as a pale orange oil, which was used in the next step without further purification.

Step 3: Et_3N (141 µL, 1 mmol) was added to a solution of (*S*)-2-amino-*N*-benzhydryl-*N*,3,3-trimethylbutanamide hydrochloride (~0.5 mmol) in dry DCM (2.55 mL) at 0 °C. After 15 min, 2-chloro-1-isothiocyanato-3,5-bis(trifluoromethyl)benzene (168 mg, ~0.55 mmol) was added, and the reaction mixture was allowed to warm to room temperature and stirred for 24 h. Then, the solvent was removed under reduced pressure, and the residue was purified by flash chromatography (*n*-hexane/EtOAc 6/1) to afford **XII** as a beige solid (203 mg, 66% in 2 steps). $[\alpha]_D^{28}$ **=** –5.3 (*c* 1, $CHCl_3$). **^{1}H-NMR** (300 MHz, $CDCl_3$): δ 8.40 (d, *J* = 9.5 Hz, 1H), 8.15 (s, 1H), 7.77 (s, 1H), 7.72 (s, 1H), 7.36 – 7.33 (m, 3H), 7.17 – 7.13 (m, 3H), 6.95 – 6.77 (m, 5H), 5.88 (d, *J* = 9.6 Hz, 1H), 3.04 (s, 3H), 1.15 (s, 9H). **^{13}C-NMR** (75.5 MHz, $CDCl_3$): δ 184.2, 174.5, 139.1, 138.8, 136.3, 133.3, 131.5, 130.1, 129.3 (q, *J* = 33.2 Hz), 129.0, 128.4, 128.4, 127.1, 127.0, 122.6 (q, *J* = 273.2 Hz), 122.3 – 122.1 (m), 62.1, 61.9, 37.1, 34.1, 27.6. **^{19}F-NMR** (471 MHz, $CDCl_3$): δ −62.90 (s, 3F), −62.88 (s, 3F). **HRMS** (ESI): *m/z* calcd for $C_{29}H_{28}ON_3ClF_6NaS$ [M^++Na] 638.1438, found 638.1427.

Synthesis of organocatalyst XIII

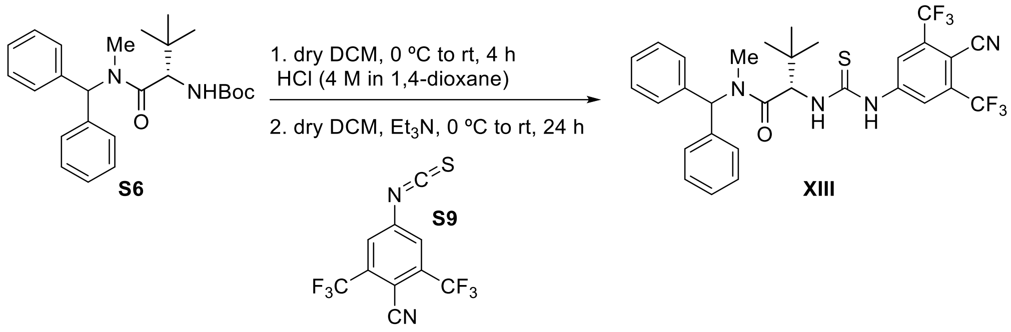

Synthesis of XIII. **Step 1**: HCl (4.0 M in 1,4-dioxane, 1.3 mL, 5 mmol) was dropwise added to a solution of *tert*-butyl (*S*)-{1-[benzhydryl(methyl)amino]-3,3-dimethyl-1-oxobutan-2-yl}carbamate (**S6**)[17] (205 mg, 0.5 mmol) in dry DCM (1.3 mL) under argon atmosphere at 0 °C. The reaction mixture was allowed to warm to room temperature and stirred for 4 h. After this time, the solvent was removed under reduced pressure to afford (*S*)-2-amino-*N*-benzhydryl-*N*,3,3-trimethylbutanamide hydrochloride as a white solid, which was used in the next step without further purification.

Step 2: Et_3N (141 µL, 1 mmol) was added to a solution of (*S*)-2-amino-*N*-benzhydryl-*N*,3,3-trimethylbutanamide hydrochloride (~0.5 mmol) in dry DCM (2.55 mL) at 0 °C. After 15 min, 4-isothiocyanato-2,6-bis(trifluoromethyl)benzonitrile (**S9**)[20] (163 mg, 0.55 mmol) was added, and the reaction mixture was allowed to warm to room temperature and stirred for 24 h. Then, the solvent was removed under reduced pressure, and the residue was purified by flash chromatography (cyhex/Et_2O 2/1) to afford **XIII** as a white solid (224 mg, 74% in 2 steps). $[\alpha]_D^{23}$ = –75.4 (*c* 1, $CHCl_3$). **^{1}H-NMR** (300 MHz, $CDCl_3$): δ 8.38 – 8.34 (m, 2H), 7.80 (s, 2H), 7.38 – 7.34 (m, 3H), 7.13 – 7.08 (m, 4H), 7.00 – 6.88 (m, 4H), 5.62 (d, *J* = 9.0 Hz, 1H), 3.16 (s, 3H), 1.19 (s, 9H). **^{13}C-NMR** (75 MHz, $CDCl_3$): δ 181.8, 174.6, 143.2, 138.5, 137.4, 135.3 (q, *J* = 33.1 Hz), 129.6, 128.9, 128.6, 128.4, 127.7, 127.5, 125.2 – 125.1 (m), 121.7 (q, *J* = 274.8 Hz), 112.0, 102.8, 62.82, 62.79, 36.6, 34.4, 27.7. **^{19}F-NMR** (471 MHz, $CDCl_3$): δ −61.92 (s, 6F). **HRMS** (ESI): *m/z* calcd for $C_{30}H_{28}ON_4F_6SNa$ $[M^+ + Na]$ 629.1780, found 629.1772.

6. Synthesis of silylphosphites 2A-F.

Diethyl (triisopropylsilyl) phosphite (2A-TIPS): To a solution of diethylphosphite (1.2 mL, 10 mmol) and triisopropylsilyl chloride (2.4 mL, 11 mmol) in dry DCM (10 mL) was added Et_3N (3 mL, 22 mmol) dropwise at 0 ºC under argon atmosphere. The reaction mixture was allowed to warm to room temperature and stirred for 24 h. Then, the solvent was removed under reduced pressure, and Et_2O was added to the residue. The resulting triethylamine hydrochloride precipitate was filtered off, and the solvent was removed under reduced pressure. This operation was performed twice. The residue was then diluted with Et_2O (12 mL) and washed with H_2O (2 x 10 mL). The organic layer was dried over $MgSO_4$, and the solvent was removed under reduced pressure to afford **2A-TIPS** as a colorless oil (2.0 g, 69%). **^{1}H-NMR** (300 MHz, CD_2Cl_2): δ 3.93 – 3.75 (m, 4H), 1.22 (t, *J* = 7.0 Hz, 6H), 1.12 – 1.06 (m, 21H). **^{13}C-NMR** (75.5 MHz, CD_2Cl_2): δ 56.9 (d, *J* = 8.8 Hz), 18.0 (d, *J* = 1.8 Hz), 17.3 (d, *J* = 4.8 Hz), 13.2. **^{31}P-NMR** (122 MHz, CD_2Cl_2): δ 124.79. **HRMS** (ESI): *m/z* calcd for $C_{13}H_{32}O_3PSi$ [M^++H] 295.3758, found 295.3763.

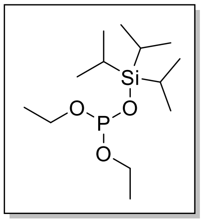

***tert*-Butyldimethylsilyl dimethyl phosphite (2B):** To a solution of dimethylphosphite (0.9 mL, 10 mmol) and *tert*-butyldimethyl silyl chloride (1.7 g, 11 mmol) in dry DCM (10 mL) was added Et_3N (3 mL, 22 mmol) dropwise at 0 ºC under argon atmosphere. The reaction mixture was allowed to warm to room temperature and stirred for 24 h. Then, the solvent was removed under reduced pressure, and Et_2O was added to the residue. The resulting triethylamine hydrochloride precipitate was filtered off, and the solvent was removed under reduced pressure. This operation was performed twice. The residue was then diluted with Et_2O (12 mL) and washed with H_2O (2 x 10 mL). The organic layer was dried over $MgSO_4$, and the solvent was removed under reduced pressure to afford **2B** as a colorless oil (1.8 g, 82%). **^{1}H-NMR** (300 MHz, CD_2Cl_2) δ 3.43 (d, *J* = 10.4 Hz, 6H), 0.93 (s, 9H), 0.18 (s, 6H). **^{13}C-NMR** (75.5 MHz, CD_2Cl_2): δ 48.0 (d, *J* = 7.5 Hz), 25.81 – 25.80 (m), 18.6, -3.2 (d, *J* = 2.8 Hz). **^{31}P-NMR** (122 MHz, CD_2Cl_2): δ 127.19. **HRMS** (ESI): *m/z* calcd for $C_8H_{22}O_3PSi$ [M^++H] 225.1070, found 225.1068.

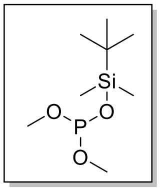

Dibutyl (*tert*-butyldimethylsilyl) phosphite (2C): To a solution of dibutylphosphite (2.0 mL, 10 mmol) and *tert*-butyldimethyl silyl chloride (1.7 g, 11 mmol) in dry DCM (10 mL) was added Et_3N (3 mL, 22 mmol) dropwise at 0 ºC under argon atmosphere. The reaction mixture was allowed to warm to room temperature and stirred for 24 h. Then, the solvent was removed under reduced pressure, and Et_2O was added to the residue. The resulting triethylamine hydrochloride precipitate was filtered off, and the solvent was removed under reduced pressure. This operation was performed twice. The residue was then diluted with Et_2O (12 mL) and washed with H_2O (2 x 10 mL). The organic layer was dried over $MgSO_4$, and the solvent was removed under reduced pressure to afford **2C** as a colorless oil (2.6 g, 85%). **^{1}H-NMR** (300 MHz, CD_2Cl_2): δ 3.78 – 3.71 (m, 4H), 1.60 – 1.51 (m, 4H), 1.44 – 1.32 (m, 4H), 0.94 – 0.90 (m, 15H), 0.17 (s, 6H). **^{13}C-NMR** (75.5 MHz, CD_2Cl_2): δ 61.9 (d, *J* = 8.1 Hz), 33.8 (d, *J* = 4.7 Hz), 25.9 (d, *J* = 1.0 Hz), 19.7, 18.6, 14.1, -3.1 (d, *J* = 2.8 Hz). **^{31}P-NMR** (122 MHz, CD_2Cl_2): δ 126.88. **HRMS** (ESI): *m/z* calcd for $C_{14}H_{34}O_3PSi$ [M^++H] 309.2009, found 309.2008.

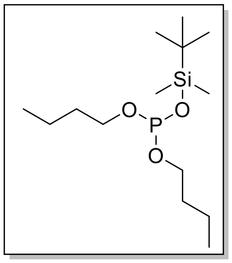

Dibenzyl (*tert*-butyldimethylsilyl) phosphite (2E): To a solution of dibenzylphosphite (2.2 mL, 10 mmol) and *tert*-butyldimethyl silyl chloride (1.7 g, 11 mmol) in dry DCM (10 mL) was added Et_3N (3 mL, 22 mmol) dropwise at 0 ºC under argon atmosphere. The reaction mixture was allowed to warm to room temperature and stirred for 24 h. Then, the solvent was removed under reduced pressure, and Et_2O was added to the residue. The resulting triethylamine hydrochloride precipitate was filtered off, and the solvent was removed under reduced pressure. This operation was performed twice. The residue was then diluted with Et_2O (12 mL) and washed with H_2O (2 x 10 mL). The organic layer was dried over $MgSO_4$, and the solvent was removed under reduced pressure to afford **2E** as a colorless oil (2.0 g, 76%). **^{1}H-NMR** (300 MHz, CD_2Cl_2): δ 7.37 – 7.25 (m, 10H), 4.90 – 4.78 (m, 4H), 0.94 (s, 9H), 0.19 (s, 6H). **^{13}C-NMR** (75.5 MHz, CD_2Cl_2): δ 139.4 (d, J = 4.9 Hz), 128.9, 128.05, 127.96, 63.3 (d, J = 8.4 Hz), 25.8, 18.6, -3.1 (d, J = 2.7 Hz). **^{31}P-NMR** (122 MHz, CD_2Cl_2): δ 126.71. **HRMS** (ESI): *m/z* calcd for $C_{20}H_{30}O_3PSi$ [M^++H] 377.1696, found 377.1697.

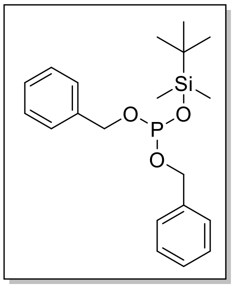

***tert*-Butyldimethylsilyl cyclohexyl methyl phosphite (2F):** To a solution of cyclohexyl methyl phosphite[21] (1.8 g, 10 mmol) and *tert*-butyldimethyl silyl chloride (1.7 g, 11 mmol) in dry DCM (10 mL) was added Et_3N (3 mL, 22 mmol) dropwise at 0 ºC under argon atmosphere. The reaction mixture was allowed to warm to room temperature and stirred for 24 h. Then, the solvent was removed under reduced pressure, and Et_2O was added to the residue. The resulting triethylamine hydrochloride precipitate was filtered off, and the solvent was removed under reduced pressure. This operation was performed twice. The residue was then diluted with Et_2O (12 mL) and washed with H_2O (2 x 10 mL). The organic layer was dried over $MgSO_4$, and the solvent was removed under reduced pressure to afford **2F** as a colorless oil (2.0 g, 55%). **^{1}H-NMR** (300 MHz, CD_2Cl_2): δ 4.09 – 3.97 (m, 1H), 3.42 (d, J = 9.6 Hz, 3H), 1.86 – 1.68 (m, 4H), 1.49 – 1.18 (m, 6H), 0.93 (s, 9H), 0.17 (s, 6H). **^{13}C-NMR** (75.5 MHz, CD_2Cl_2): δ 71.3 (d, J = 13.4 Hz), 47.3 (d, J = 3.7 Hz), 35.2 – 35.1 (m), 26.1, 25.9 – 25.8 (m), 24.6 (d, J = 1.3 Hz), 18.6, -3.1 (t, J = 2.6 Hz). **^{31}P-NMR** (122 MHz, CD_2Cl_2): δ 126.77. **HRMS** (ESI): *m/z* calcd for $C_{13}H_{30}O_3PSi$ [M^++H] 293.1696, found 293.1695.

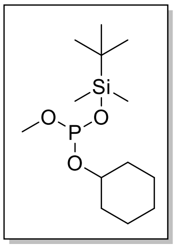

7. Preliminary experiments.

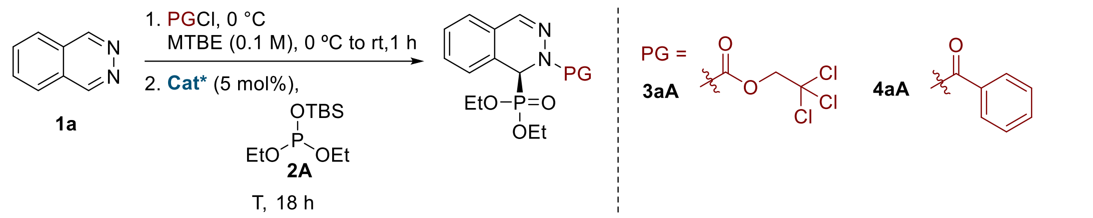

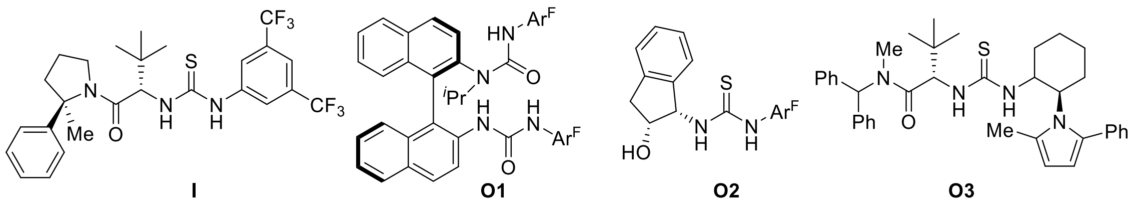

In a flame-dried Schlenk flask, the acylating reagent (0.1 mmol) was added to a solution of phthalazine (**1a**) (13 mg, 0.1 mmol) in freshly distilled anhydrous MTBE (1 mL, 0.1 M) at 0 ºC. The resulting suspension was stirred for 1 h at room temperature. Then, the corresponding organocatalyst (0.005 mmol, 5 mol%) was added, and the reaction was cooled to −78 ºC (dry ice/acetone bath). *Tert*-butyldimethylsilyl diethyl phosphite (**2A**) (30 μL, 0.11 mmol) was added, and the reaction mixture was stirred for 18 h and allowed to warm slowly to room temperature during that time. Then, the solvent was removed under reduced pressure. NMR yield was determined by ^{1}H-NMR analysis of the crude reaction mixture and enantiomeric ratios were determined by HPLC analysis.

Table S1

Entry	PG	Cat*	T (ºC)	3/4	Yield (%)[a]	ee (%)[b]
1[c]	Troc	-	-78 °C to rt	**3aA**	99	--
2[c]	Troc	**I**	-78 °C to rt	**3aA**	82	47
3[c]	Bz	-	-78 °C to rt	**4aA**	74	--
4[c]	Bz	**I**	-78 °C to rt	**4aA**	91	82
5[c]	Troc	**I**	-78 °C	**3aA**	22	29
6[c]	Bz	**I**	-78 °C	**4aA**	26	45
7	Bz	**I**	-78 °C to rt	**4aA**	85	84
8	Bz	**O1**	-78 °C to rt	**4aA**	66	10
9	Bz	**O2**	-78 °C to rt	**4aA**	58	17
10	Bz	**O3**	-78 °C to rt	**4aA**	54	*rac*

[a]Determined by ^{1}H-NMR using mesitylene as internal standard. [b]Determined by HPLC analysis after isolation of the product by semipreparative TLC (*n*-hexane/EtOAc 1/2). [c]**2A**: 0.2 mmol.

8. Preliminary screening of chiral organocatalysts.

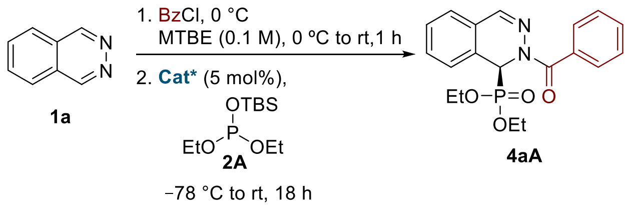

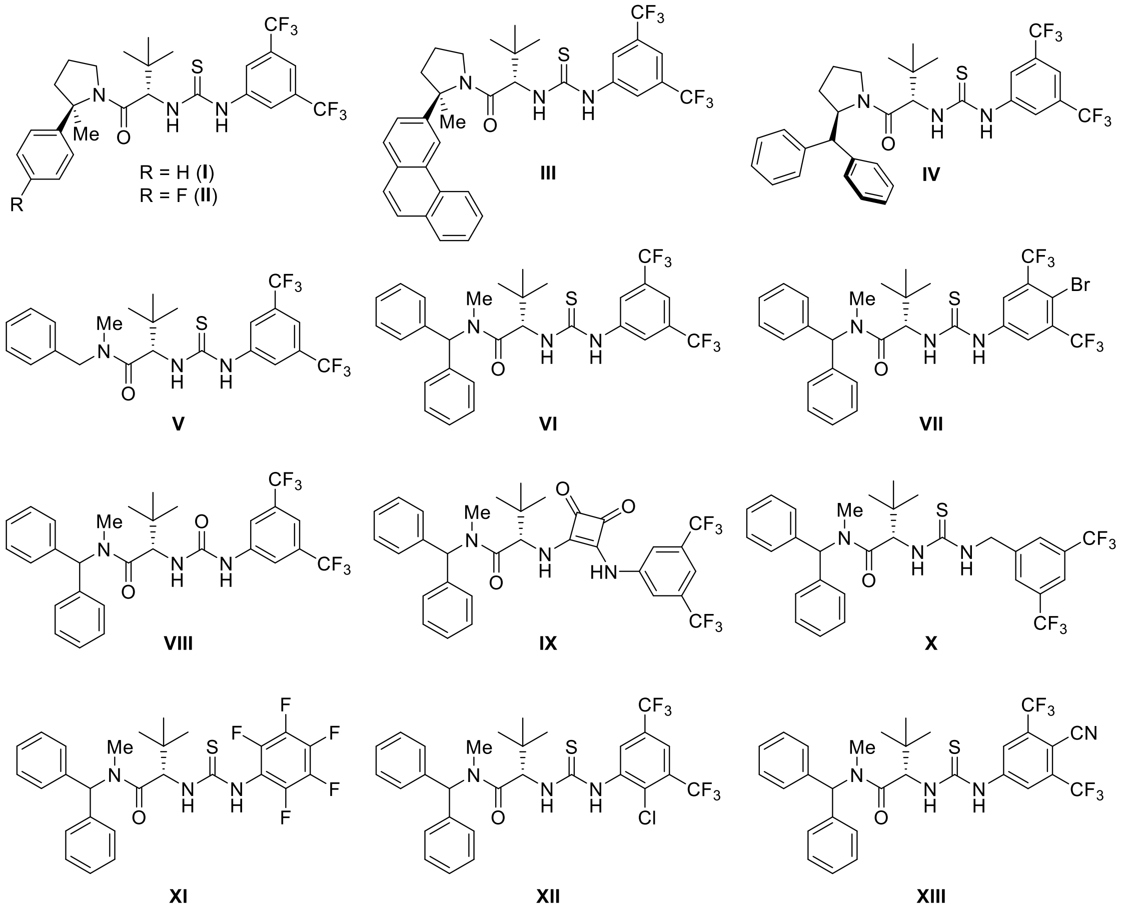

In a flame-dried Schlenk flask, benzoyl chloride (12 µL, 0.1 mmol) was added to a solution of phthalazine (**1a**) (13 mg, 0.1 mmol) in freshly distilled anhydrous MTBE (1 mL, 0.1 M) at 0 ºC. The resulting suspension was stirred for 1 h at room temperature. Then, the corresponding organocatalyst (0.005 mmol, 5 mol%) was added, and the reaction was cooled to −78 ºC (dry ice/acetone bath). *Tert*-butyldimethylsilyl diethyl phosphite (**2A**) (30 µL, 0.11 mmol) was added, and the reaction mixture was stirred for 18 h and allowed to warm slowly to room temperature during that time. Then, the solvent was removed under reduced pressure. NMR yield was determined by ^{1}H-NMR analysis of the crude reaction mixture and enantiomeric ratios were determined by HPLC analysis.

Table S2

Entry	Cat*	Yield (%)[a]	ee (%)[b]
1	**I**	91	82
2	**II**	68	81
3	**III**	79	40
4	**IV**	64	7
5	**V**	66	85
6	**VI**	76	87
7	**VII**	84	90
8	**VIII**	74	77
9	**IX**	49	32
10	**X**	76	45
11	**XI**	84	18
12	**XII**	77	37
13	**XIII**	77	90
14[c]	**VII**	87	87
15[d]	**VII**	88	87

[a]Determined by ^{1}H-NMR using mesitylene as internal standard. [b]Determined by HPLC analysis after isolation of the product by semipreparative TLC (*n*-hexane/EtOAc 1/2). [c]**2A**: 0.15 mmol. [d]**2A**: 0.2 mmol.

9. Solvent optimization.

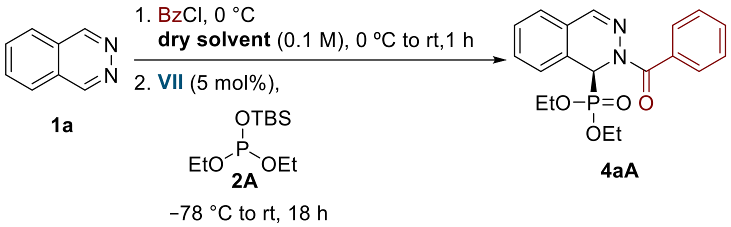

In a flame-dried Schlenk flask, benzoyl chloride (12 µL, 0.1 mmol) was added to a solution of phthalazine (**1a**) (13 mg, 0.1 mmol) in the freshly distilled corresponding solvent (1 mL, 0.1 M) at 0 ºC. The resulting suspension was stirred for 1 h at room temperature. Then, catalyst **VII** (3 mg, 0.005 mmol, 5 mol%) was added and the reaction was cooled to −78 ºC (dry ice/acetone bath). *Tert*-butyldimethylsilyl diethyl phosphite (**2A**) (30 µL, 0.11 mmol) was added, and the reaction mixture was stirred for 18 h and allowed to warm slowly to room temperature during that time. Then, the solvent was removed under reduced pressure. NMR yield was determined by ^{1}H-NMR analysis of the crude reaction mixture and enantiomeric ratios were determined by HPLC analysis.

Table S3

Entry	Solvent	Yield (%)[a]	ee (%)[b]
1	MTBE	84	90
2	Et_2O	77	85
3	CPME	58	92
4	THF	88	87
5	2-MeTHF	75	89
6	CH_2Cl_2	76	56
7	Toluene	57	80
8[c]	C_6F_6	88	87
9[d]	MTBE	82	88
10	MTBE (0.2M)	65	87
11	MTBE (0.05M)	60	85
12[e]	MTBE	83	87

[a]Determined by ^{1}H-NMR using mesitylene as internal standard. [b]Determined by HPLC analysis after isolation of the product by semipreparative TLC (*n*-hexane/EtOAc 1/2). [c]The reaction was performed at 4 °C. [d]The reaction was performed using MS 4Å. [e]Cat **VII** loading: 2.5 mol%.

10. Optimization of the acylating reagent.

Scheme S1[a]

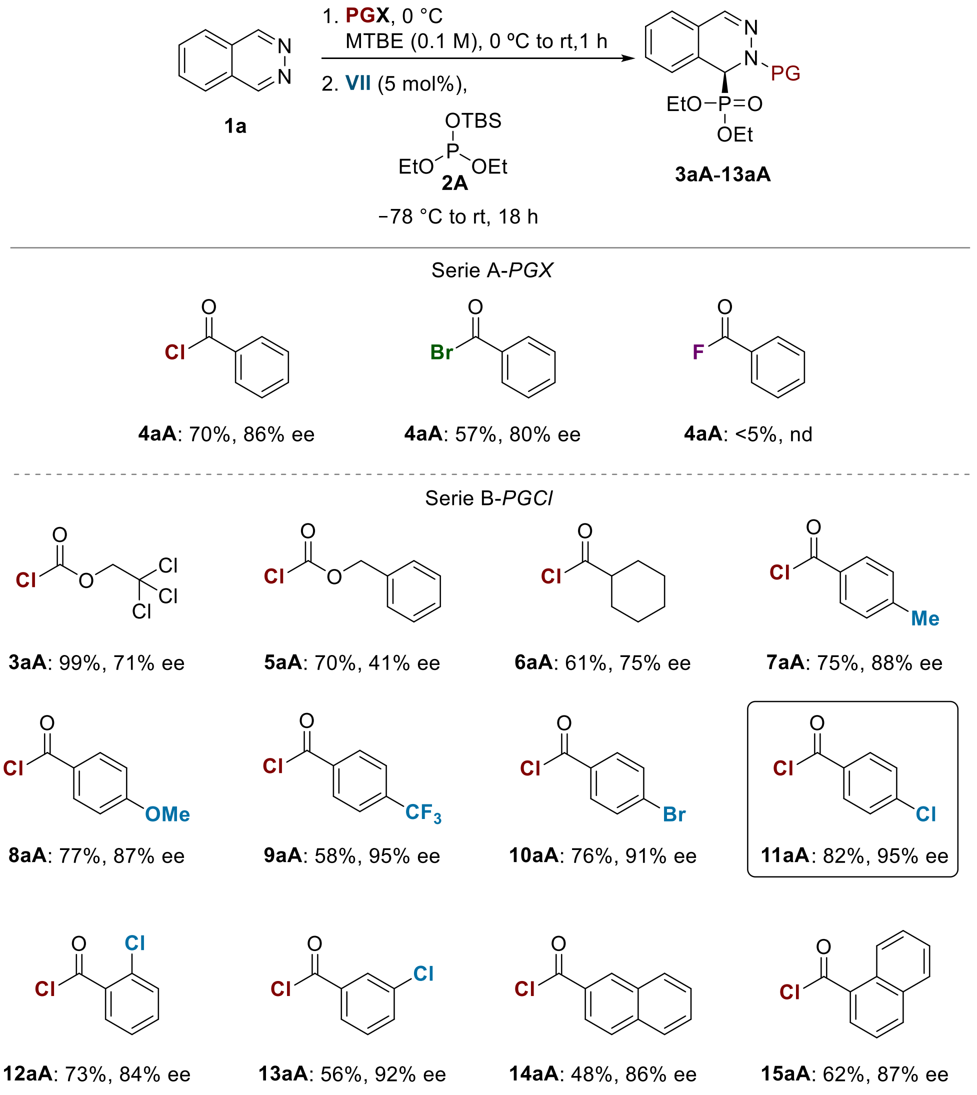

[a]Reactions performed at 0.2 mmol scale of **1a.** Yields given for isolated products after chromatography. Enantiomeric excesses were determined by HPLC on chiral stationary phases.

11. General procedure for the optimization of the acylating reagents.

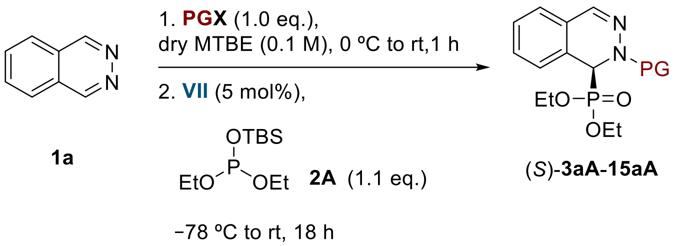

In a flame-dried Schlenk flask, the acylating reagent (0.2 mmol) was added to a solution of phthalazine (**1a**) (27 mg, 0.2 mmol) in freshly distilled anhydrous MTBE (2 mL, 0.1 M) at 0 ºC. The resulting suspension was stirred for 1 h at room temperature. Then, catalyst **VII** (7 mg, 0.01 mmol, 5 mol%) was added, and the reaction was cooled to −78 ºC (dry ice/acetone bath). *Tert*-butyldimethylsilyl diethyl phosphite (**2A**) (60 µL, 0.22 mmol) was added, and the reaction mixture was stirred for 18 h and allowed to warm slowly to room temperature during that time. Then, the solvent was removed under reduced pressure, and the residue was purified by flash chromatography to afford the corresponding products (*S*)-**3aA-15aA**. Enantiomeric ratios were determined by HPLC analysis.

Racemic products were prepared without catalyst following the general procedure described above.

2,2,2-trichloroethyl (*S*)-1-(diethoxyphosphoryl)phthalazine-2(1*H*)-carboxylate [(*S*)-3aA]: Following the general procedure **11**, employing 2,2,2-trichloroethyl chloroformate (28 µL, 0.2 mmol) as acylating reagent, (*S*)-**3aA** was obtained after purification by flash chromatography (*n*-hexane/EtOAc 1/1) as a colorless oil (88 mg, 99%, 71% ee). **$[\alpha]_D^{29}$ =** +200.0 (*c* 1, $CHCl_3$). **^{1}H-NMR** (300 MHz, $CDCl_3$): The compound exists as a ~2.5:1 mixture of carbamate rotamers. *Signals corresponding to the major rotamer:* δ 7.72 (s, 1H), 7.52 – 7.29 (m, 4H), 5.98 (d, *J* = 15.3 Hz, 1H), 4.96 (dd, *J* = 34.9, 11.9 Hz, 2H), 4.18 – 4.03 (m, 2H), 4.00 – 3.78 (m, 2H), 1.26 (t, *J* = 7.0 Hz, 3H), 1.13 (t, *J* = 7.1 Hz, 3H). *Signals corresponding to the minor rotamer:* δ 7.83 (s, 1H), 5.87 (d, *J* = 14.9 Hz, 1H), 5.12 (d, *J* = 11.8 Hz, 1H), 4.71 (d, *J* = 11.8 Hz, 1H). **^{13}C-NMR** (75.5 MHz, $CDCl_3$): *Signals corresponding to both rotamers*: δ 152.5, 151.6, 146.4, 145.3, 132.2 (d, *J* = 2.7 Hz), 129.5 (d, *J* = 3.0 Hz), 129.3 (d, *J* = 3.3 Hz), 127.6 – 127.5 (m), 127.4 – 127.3 (m), 126.5 (d, *J* = 2.9 Hz), 126.3 (d, *J* = 3.0 Hz), 124.4 (d, *J* = 3.8 Hz), 124.3 (d, *J* = 3.9 Hz), 95.1, 94.7, 75.9, 75.8, 63.7 – 63.6 (m), 63.4 – 63.1 (m), 52.8 (d, *J* = 152.3 Hz), 51.6 (d, *J* = 152.9 Hz), 16.6 – 16.1 (m). **^{31}P-NMR** (122 MHz, $CDCl_3$): *Signal corresponding to the major rotamer:* δ 16.99. *Signal corresponding to the minor rotamer:* δ 16.68. **HRMS** (ESI): *m/z* calcd for $C_{15}H_{18}O_5N_2Cl_3NaP$ $[M^++Na]$ 464.9911, found 464.9905. **HPLC** (Chiralpak IB, *n*-hexane/propan-2-ol 95:5, flow 1 mL/min) t_R 19.9 min (minor) and 21.7 min (major).

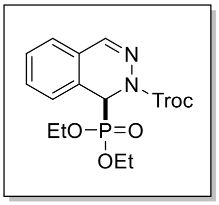

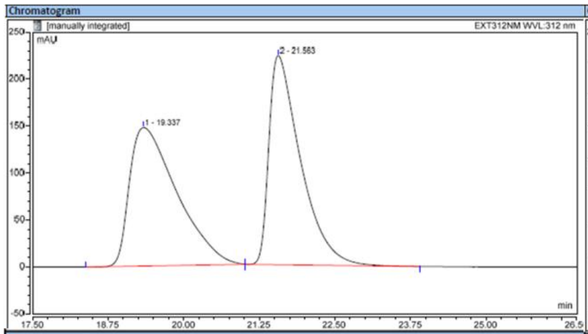

Integration Results			
No.	Retention Time min	Area mAU*min	Relative Area %
1	19.337	133.516	49.76
2	21.563	134.799	50.24

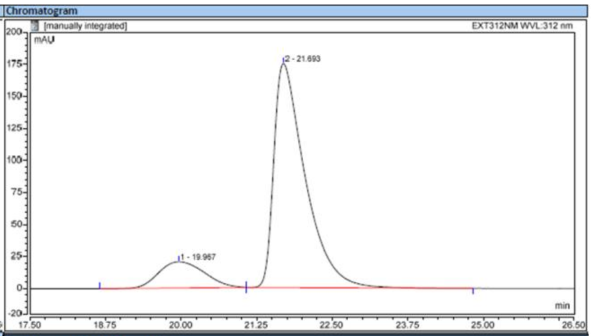

Integration Results			
No.	Retention Time min	Area mAU*min	Relative Area %
1	19.967	17.790	14.50
2	21.693	104.894	85.50

Diethyl (*S*)-(2-benzoyl-1,2-dihydrophthalazin-1-yl)phosphonate [(*S*)-4aA]: Following the general procedure **11**, employing benzoyl chloride (24 μL, 0.2 mmol) as acylating reagent, (*S*)-**4aA** was obtained after purification by flash chromatography (*n*-hexane/EtOAc 1/2) as a colorless oil (52 mg, 70%, 86% ee). **[α]$_D$^{28} =** +570.7 (*c* 1, $CHCl_3$). **^{1}H-NMR** (300 MHz, $CDCl_3$): δ 7.58 – 7.56 (m, 2H), 7.47 (s, 1H), 7.41 – 7.26 (m, 6H), 7.16 (d, *J* = 7.0 Hz, 1H), 6.39 (d, *J* = 16.5 Hz, 1H), 4.09 – 4.00 (m, 2H), 3.93 – 3.67 (m, 2H), 1.16 (t, *J* = 7.0 Hz, 3H), 1.01 (t, *J* = 7.0 Hz, 3H). **^{13}C-NMR** (75.5 MHz, $CDCl_3$): δ 169.7, 143.8, 134.4, 132.1 (d, *J* = 2.8 Hz), 130.6, 129.4, 129.2 (d, *J* = 3.4 Hz), 127.8, 127.7, 127.6 (d, *J* = 2.7 Hz), 126.1 (d, *J* = 3.0 Hz), 124.7 (d, *J* = 4.0 Hz), 63.4 (d, *J* = 6.3 Hz), 63.1 (d, *J* = 7.4 Hz), 49.6 (d, *J* = 152.0 Hz), 16.5 – 16.3 (m). **^{31}P-NMR** (122 MHz, $CDCl_3$): δ 17.43. **HRMS** (ESI): *m/z* calcd for $C_{19}H_{21}O_4N_2NaP$ [M^++Na] 395.1131, found 395.1125. **HPLC** (Chiralpak IB, *n*-hexane/propan-2-ol 90:10, flow 1 mL/min) t_R 12.4 min (minor) and 14.7 min (major).

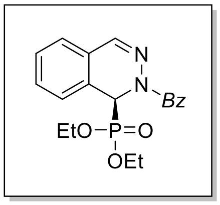

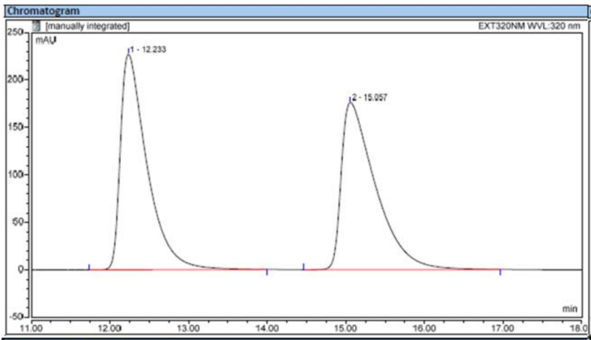

Integration Results			
No.	Retention Time min	Area mAU*min	Relative Area %
1	12.233	88.854	50.04
2	15.057	88.707	49.96

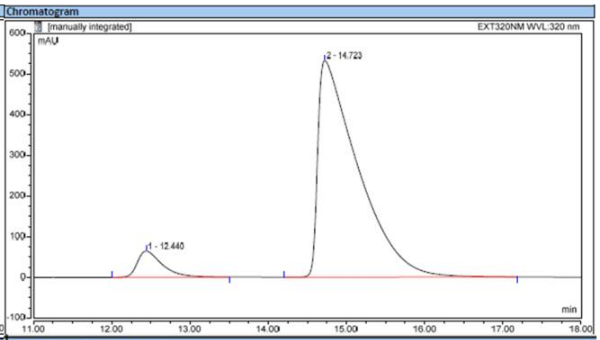

Integration Results			
No.	Retention Time min	Area mAU*min	Relative Area %
1	12.440	23.723	6.96
2	14.723	317.255	93.04

Benzyl (*S*)-1-(diethoxyphosphoryl)phthalazine-2(1*H*)-carboxylate [(*S*)-5aA]: Following the general procedure **11**, employing benzyl chloroformate (29 μL, 0.2 mmol) as acylating reagent, (*S*)-**5aA** was obtained after purification by flash chromatography (*n*-hexane/EtOAc 1/1) as a colorless oil (56 mg, 70%, 41% ee). **[α]$_D^{28}$** = +192.3 (*c* 1, $CHCl_3$). **^{1}H-NMR** (300 MHz, $CDCl_3$): The compound exists as a ~3.4:1 mixture of carbamate rotamers. *Signals corresponding to the major rotamer:* δ 7.64 (s, 1H), 7.48 – 7.44 (m, 3H), 7.40 – 7.25 (m, 6H), 6.00 (d, *J* = 15.1 Hz, 1H), 5.35 (s, 2H), 4.14 – 3.64 (m, 4H), 1.19 (t, *J* = 7.1 Hz, 3H), 1.08 (t, *J* = 7.0 Hz, 3H). *Signals corresponding to the minor rotamer* δ 7.76 (br s, 1H), 5.85 (d, *J* = 14.8 Hz, 1H), 5.32 (s, 2H). **^{13}C-NMR** (75.5 MHz, $CDCl_3$): *Signals corresponding to both rotamers*: δ 153.8, 145.1, 144.3, 136.1, 132.10 – 132.07 (m), 132.0 – 131.9 (m), 129.4 – 129.3 (m), 129.16 (d, *J* = 3.1 Hz), 128.8 – 128.7 (m), 128.6 (d, *J* = 4.3 Hz), 128.4, 127.5 (d, *J* = 4.9 Hz), 126.1 (d, *J* = 2.9 Hz), 124.6 (d, *J* = 3.7 Hz), 68.7, 63.6 – 63.4 (m), 63.3 – 63.1 (m), 52.7 (d, *J* = 153.0 Hz), 51.3 (d, *J* = 152.8 Hz), 16.4 (d, *J* = 5.6 Hz). **^{31}P-NMR** (122 MHz, $CDCl_3$): *Signal corresponding to the major rotamer:* δ 17.43. *Signal corresponding to the minor rotamer:* δ 17.20. **HRMS** (ESI): *m/z* calcd for $C_{20}H_{23}O_5N_2NaP$ [M^++Na] 425.1237, found 425.1233. **HPLC** (Chiralpak IA *n*-hexane/propan-2-ol 85:15, flow 1 mL/min) t_R 20.2 (minor) and 25.7 min (major).

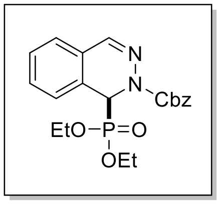

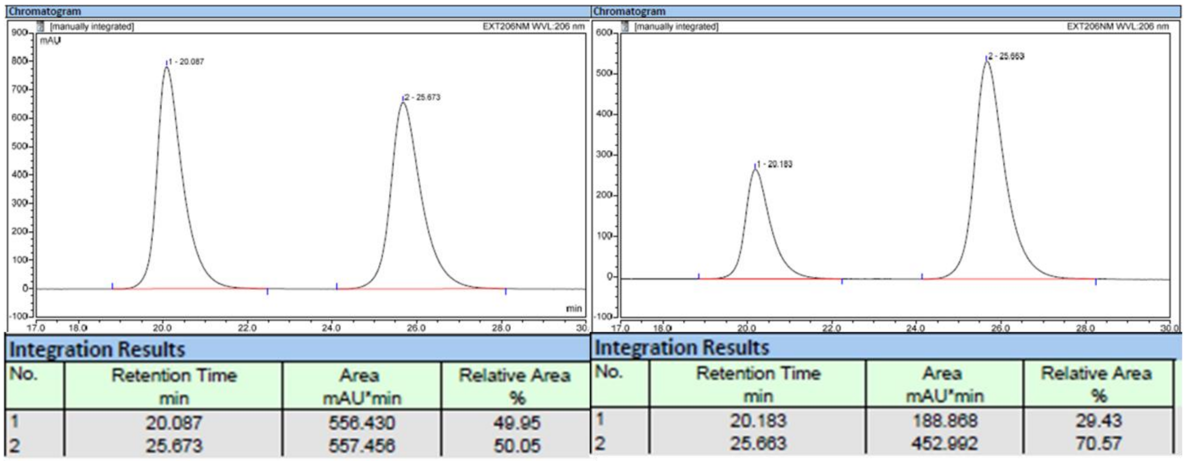

Integration Results

No.	Retention Time min	Area mAU*min	Relative Area %
1	20.087	556.430	49.95
2	25.673	557.456	50.05

Integration Results

No.	Retention Time min	Area mAU*min	Relative Area %
1	20.183	188.868	29.43
2	25.663	452.992	70.57

Diethyl (*S*)-[2-(cyclohexanecarbonyl)-1,2-dihydrophthalazin-1-yl]phosphonate [(*S*)-6aA]: Following the general procedure **11**, employing cyclohexanecarbonyl chloride (27 μL, 0.2 mmol) as acylating reagent, (*S*)-**6aA** was obtained after purification by flash chromatography (*n*-hexane/EtOAc 1/1) as a colorless oil (46 mg, 61%, 75% ee). **[α]$_D^{28}$** = +362.7 (*c* 1, $CHCl_3$). **^{1}H-NMR** (300 MHz, $CDCl_3$): δ 7.57 (s, 1H), 7.47 – 7.41 (m, 1H), 7.39 – 7.31 (m, 2H), 7.26 – 7.24 (m, 1H), 6.32 (d, *J* = 16.2 Hz, 1H), 4.14 – 4.01 (m, 2H), 3.98 – 3.73 (m, 2H), 3.35 – 3.25 (m, 1H), 1.98 – 1.68 (m, 5H), 1.58 – 1.21 (m, 8H), 1.10 (t, *J* = 7.1 Hz, 3H). **^{13}C-NMR** (75.5 MHz, $CDCl_3$): δ 176.7, 143.0, 131.9 (d, *J* = 2.8 Hz), 129.0 (d, *J* = 3.4 Hz), 127.9 (d, *J* = 5.1 Hz), 127.7 (d, *J* = 2.1 Hz), 125.9 (d, *J* = 3.1 Hz), 125.0 (d, *J* = 4.1 Hz), 63.2 (d, *J* = 6.1 Hz), 63.0 (d, *J* = 7.5 Hz), 48.6 (d, *J* = 152.3 Hz), 40.0, 29.2, 28.9, 26.1, 25.88, 25.85, 16.5 – 16.4 (m). **^{31}P-NMR** (122 MHz, $CDCl_3$): δ 17.67. **HRMS** (ESI): *m/z* calcd for $C_{19}H_{27}O_4N_2NaP$ [M^++Na] 401.1601, found 401.1589. **HPLC** (Chiralpak IB, *n*-hexane/propan-2-ol 90:10, flow 1 mL/min) t_R 6.7 min (minor) and 8.1 min (major).

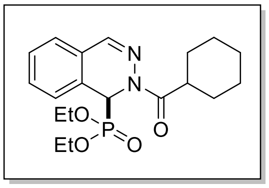

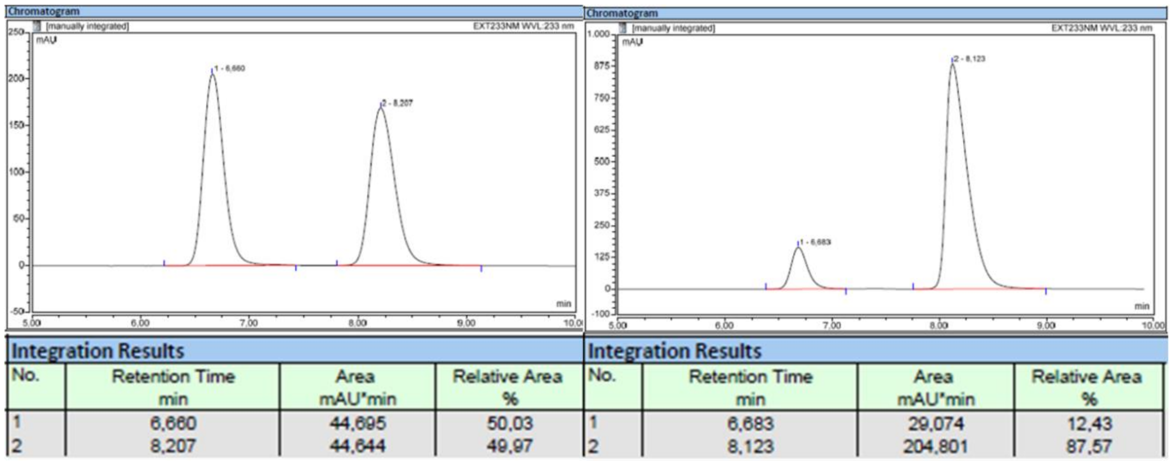

Integration Results

No.	Retention Time min	Area mAU*min	Relative Area %
1	6,660	44,695	50,03
2	8,207	44,644	49,97

Integration Results

No.	Retention Time min	Area mAU*min	Relative Area %
1	6,683	29,074	12,43
2	8,123	204,801	87,57

Diethyl (*S*)-[2-(4-methylbenzoyl)-1,2-dihydrophthalazin-1-yl]phosphonate [(*S*)-7aA]: Following the general procedure **11**, employing *p*-toluoyl chloride (27 μL, 0.2 mmol) as acylating reagent, (*S*)-**7aA** was obtained after purification by flash chromatography (*n*-hexane/EtOAc 1/2) as a colorless oil (58 mg, 75%, 88% ee). **$[\alpha]_D^{28}$ =** +624.9 (*c* 1, $CHCl_3$). **^{1}H-NMR** (300 MHz, $CDCl_3$): δ 7.63 – 7.59 (m, 3H), 7.52 – 7.47 (m, 1H), 7.43 – 7.37 (m, 2H), 7.29 – 7.26 (m, 1H), 7.22 – 7.20 (m, 2H), 6.51 (d, *J* = 16.5 Hz, 1H), 4.21 – 4.10 (m, 2H), 4.05 – 3.78 (m, 2H), 2.39 (s, 3H), 1.27 (t, *J* = 7.2 Hz, 3H), 1.12 (t, *J* = 7.1 Hz, 3H). **^{13}C-NMR** (75.5 MHz, $CDCl_3$): δ 169.7, 143.7, 141.1, 132.1 (d, *J* = 2.8 Hz), 131.4, 129.6, 129.2 (d, *J* = 3.4 Hz), 128.5, 127.8 – 127.7 (m), 126.0 (d, *J* = 3.1 Hz), 124.9 (d, *J* = 4.0 Hz), 63.4 (d, *J* = 6.3 Hz), 63.2 (d, *J* = 7.5 Hz), 49.7 (d, *J* = 152.0 Hz), 21.6, 16.5 – 16.4 (m). **^{31}P-NMR** (122 MHz, $CDCl_3$): δ 17.52. **HRMS** (ESI): *m/z* calcd for $C_{20}H_{23}O_4N_2NaP$ $[M^+ + Na]$ 409.1288, found 409.1281. **HPLC** (Chiralpak IB, *n*-hexane/propan-2-ol 90:10, flow 1 mL/min) t_R 12.8 min (minor) and 14.9 min (major).

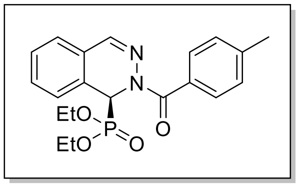

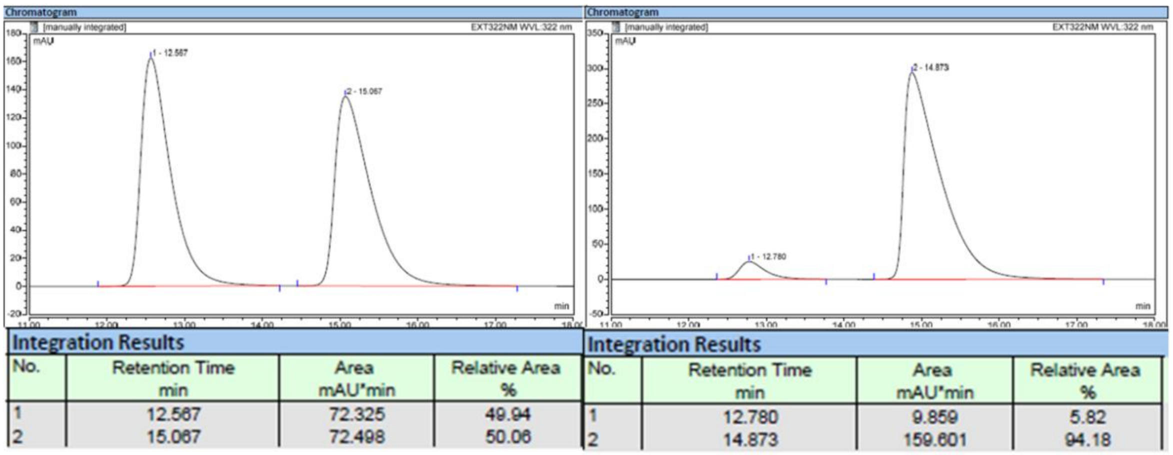

Integration Results

No.	Retention Time min	Area mAU*min	Relative Area %
1	12.567	72.325	49.94
2	15.067	72.498	50.06

Integration Results

No.	Retention Time min	Area mAU*min	Relative Area %
1	12.780	9.859	5.82
2	14.873	159.601	94.18

Diethyl (*S*)-[2-(4-methoxybenzoyl)-1,2-dihydrophthalazin-1-yl]phosphonate [(*S*)-8aA]: Following the general procedure **11**, employing *p*-methoxybenzoyl chloride (27 µL, 0.2 mmol) as acylating reagent, (*S*)-**8aA** was obtained after purification by flash chromatography (*n*-hexane/EtOAc 1/2) as a colorless oil (62 mg, 77%, 87% ee). **[α]$_D^{28}$ =** +648.3 (*c* 1, CHCl$_3$). **^{1}H-NMR** (300 MHz, CDCl$_3$): δ 7.77 – 7.72 (m, 2H), 7.60 (s, 1H), 7.52 – 7.37 (m, 3H), 7.30 – 7.27 (m, 1H), 6.93 – 6.88 (m, 2H), 6.50 (d, *J* = 16.5 Hz, 1H), 4.19 – 4.10 (m, 2H), 4.04 – 3.79 (m, 5H), 1.26 (t, *J* = 7.1 Hz, 3H), 1.12 (t, *J* = 7.1 Hz, 3H). **^{13}C-NMR** (75.5 MHz, CDCl$_3$): δ 169.0, 161.7, 143.6, 132.1 (d, *J* = 2.8 Hz), 132.0, 129.1 (d, *J* = 3.5 Hz), 127.8, 127.7 (d, *J* = 3.0 Hz), 126.3, 126.0 (d, *J* = 3.1 Hz), 124.9 (d, *J* = 4.0 Hz), 113.1, 63.3 (d, *J* = 6.3 Hz), 63.1 (d, *J* = 7.4 Hz), 55.4, 49.8 (d, *J* = 151.9 Hz), 16.5 – 16.4 (m). **^{31}P-NMR** (122 MHz, CDCl$_3$): δ 17.63. **HRMS** (ESI): *m/z* calcd for $C_{20}H_{23}O_5N_2NaP$ [M$^+$+Na] 425.1237, found 425.1227. **HPLC** (Chiralpak IB, *n*-hexane/propan-2-ol 90:10, flow 1 mL/min) t_R 19.8 min (minor) and 23.4 min (major).

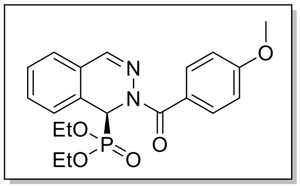

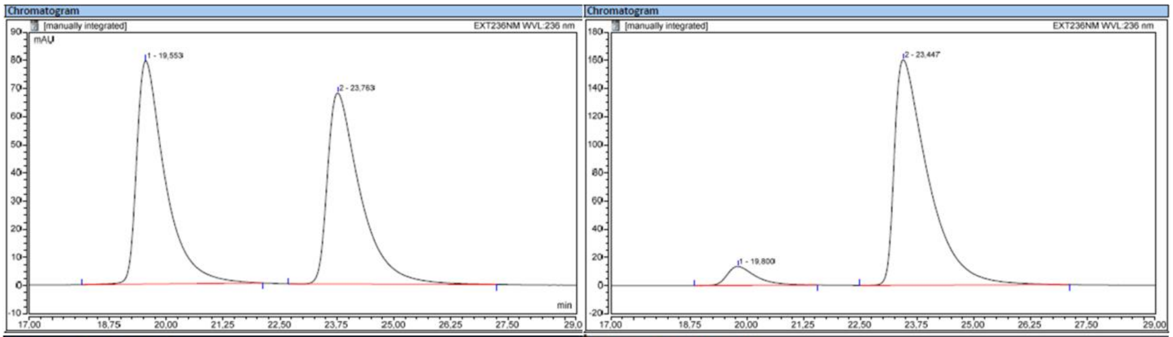

Integration Results

No.	Retention Time min	Area mAU*min	Relative Area %
1	19,553	57,560	50,41
2	23,763	56,626	49,59

Integration Results

No.	Retention Time min	Area mAU*min	Relative Area %
1	19,800	9,750	6,61
2	23,447	137,657	93,39

Diethyl (*S*)-{2-[4-(trifluoromethyl)benzoyl]-1,2-dihydrophthalazin-1-yl)}phosphonate [(*S*)-9aA]: Following the general procedure **11**, employing 4-(trifluoromethyl)benzoyl chloride (30 µL, 0.2 mmol) as acylating reagent, (*S*)-**9aA** was obtained after purification by flash chromatography (*n*-hexane/EtOAc 1/1) as a colorless oil (51 mg, 58%, 95% ee). **[α]$_D^{28}$ =** +597.6 (*c* 1, CHCl$_3$). **^{1}H-NMR** (300 MHz, CDCl$_3$): δ 7.79 (d, *J* = 8.1 Hz, 2H), 7.67 (d, *J* = 8.2 Hz, 2H), 7.58 (s, 1H), 7.55 – 7.50 (m, 1H), 7.45 – 7.39 (m, 2H), 7.31 – 7.28 (m, 1H), 6.48 (d, *J* = 16.5 Hz, 1H), 4.24 – 4.09 (m, 2H), 4.05 – 3.80 (m, 2H), 1.27 (t, *J* = 7.1 Hz, 3H), 1.14 (t, *J* = 7.0 Hz, 3H). **^{13}C-NMR** (75.5 MHz, CDCl$_3$): δ 168.6, 144.3, 138.1, 132.9, 132.4 (d, *J* = 2.8 Hz), 132.0, 131.6, 129.7, 129.4 (d, *J* = 3.5 Hz), 127.9 (d, *J* = 3.5 Hz), 127.9 (d, *J* = 5.0 Hz), 127.5 (d, *J* = 2.6 Hz), 126.4 (d, *J* = 3.1 Hz), 125.7, 124.9 – 124.8 (m), 124.6 (d, *J* = 4.2 Hz), 122.1, 118.5, 63.5 (d, *J* = 6.3 Hz), 63.3 (d, *J* = 7.5 Hz), 49.7 (d, *J* = 152.2 Hz), 16.5 – 16.4 (m). **^{31}P-NMR** (122 MHz, CDCl$_3$): δ 17.09. **^{19}F NMR** (471 MHz, CDCl$_3$): δ −62.96 (s, 3F). **HRMS** (ESI): *m/z* calcd for $C_{20}H_{20}O_4N_2F_3NaP$ [M$^+$+Na] 463.1005, found 463.0993. **HPLC** (Chiralpak IB, *n*-hexane/propan-2-ol 90:10, flow 1 mL/min) t_R 10.3 min (minor) and 12.5 min (major).

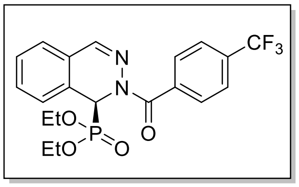

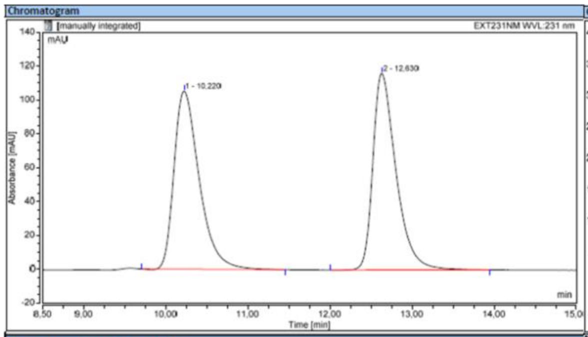

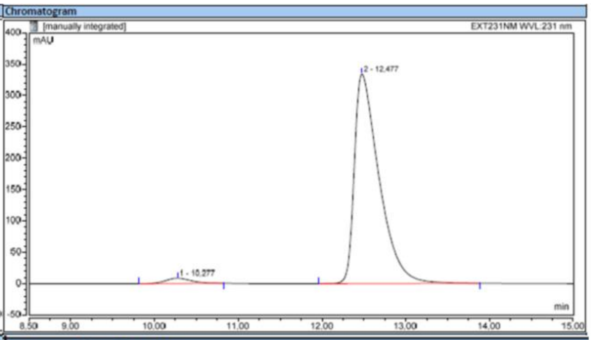

Integration Results

No.	Retention Time min	Area mAU*min	Relative Area %
1	10,220	37,416	49,71
2	12,630	37,850	50,29

Integration Results

No.	Retention Time min	Area mAU*min	Relative Area %
1	10,277	2,924	2,50
2	12,477	113,915	97,50

Diethyl (*S*)-[2-(4-bromobenzoyl)-1,2-dihydrophthalazin-1-yl]phosphonate [(*S*)-10aA]: Following the general procedure **11**, employing *p*-bromobenzoyl chloride (45 mg, 0.2 mmol) as acylating reagent, (*S*)-**10aA** was obtained after purification by flash chromatography (*n*-hexane/EtOAc 1/1) as a colorless oil (69 mg, 76%, 91% ee). $[\alpha]_D^{28}$ **=** +607.3 (*c* 1, $CHCl_3$). ^{1}**H-NMR** (300 MHz, $CDCl_3$): δ 7.61 – 7.48 (m, 6H), 7.44 – 7.38 (m, 2H), 7.30 – 7.27 (m, 1H), 6.47 (d, *J* = 16.5 Hz, 1H), 4.20 – 4.07 (m, 2H), 4.04 – 3.78 (m, 2H), 1.26 (t, *J* = 7.1 Hz, 3H), 1.12 (t, *J* = 7.0 Hz, 3H). ^{13}**C-NMR** (75.5 MHz, $CDCl_3$): δ 168.8, 144.1, 133.2, 132.3 (d, *J* = 2.9 Hz), 131.2, 1310, 129.3 (d, *J* = 3.5 Hz), 127.8 (d, *J* = 5.1 Hz), 127.6 (d, *J* = 2.5 Hz), 126.2 (d, *J* = 3.1 Hz), 125.3, 126.7 (d, *J* = 4.0 Hz), 63.5 (d, *J* = 6.3 Hz), 63.2 (d, *J* = 7.5 Hz), 49.7 (d, *J* = 152.1 Hz), 16.5 – 16.4 (m). ^{31}**P-NMR** (122 MHz, $CDCl_3$): δ 17.24. **HRMS** (ESI): *m/z* calcd for $C_{19}H_{20}O_4N_2BrNaP$ $[M^+ + Na]$ 473.0236, found 473.0226. **HPLC** (Chiralpak IB, *n*-hexane/propan-2-ol 90:10, flow 1 mL/min) t_R 12.2 min (minor) and 14.2 min (major).

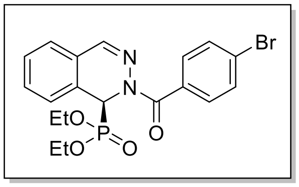

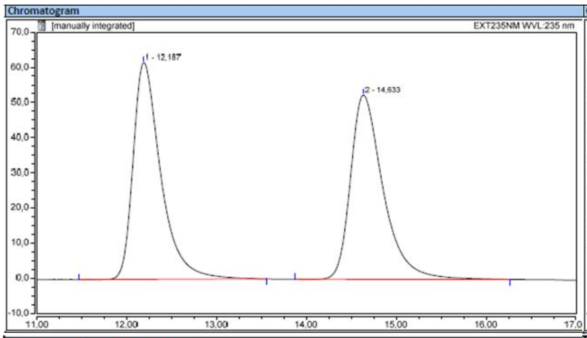

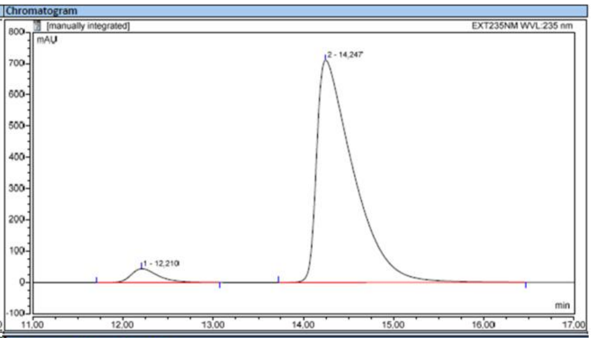

Integration Results

No.	Retention Time min	Area mAU*min	Relative Area %
1	12,187	22,039	50,01
2	14,633	22,030	49,99

Integration Results

No.	Retention Time min	Area mAU*min	Relative Area %
1	12,210	15,452	4,42
2	14,247	333,989	95,58

Diethyl (*S*)-[2-(4-chlorobenzoyl)-1,2-dihydrophthalazin-1-yl]phosphonate [(*S*)-11aA]: Following the general procedure **11**, employing *p*-chlorobenzoyl chloride (26 μL, 0.2 mmol) as acylating reagent, (*S*)-**11aA** was obtained after purification by flash chromatography (*n*-hexane/EtOAc 1/1) as a white solid (67 mg, 82%, 95% ee). **$[\alpha]_D^{27}$ =** +672.8 (*c* 1, $CHCl_3$). **^{1}H-NMR** (300 MHz, $CDCl_3$): δ 7.68 – 7.63 (m, 2H), 7.58 (s, 1H), 7.52 – 7.47 (m, 1H), 7.43 – 7.34 (m, 4H), 7.29 – 7.27 (m, 1H), 6.47 (d, *J* = 16.5 Hz, 1H), 4.19 – 4.07 (m, 2H), 4.04 – 3.78 (m, 2H), 1.25 (t, *J* = 7.0 Hz, 3H), 1.12 (t, *J* = 7.1 Hz, 3H). **^{13}C-NMR** (75.5 MHz, $CDCl_3$): δ 168.6, 144.0, 136.8, 132.7, 132.2 (d, *J* = 2.9 Hz), 131.1, 129.2 (d, *J* = 3.4 Hz), 128.0, 127.8 (d, *J* = 5.1 Hz), 127.5 (d, *J* = 2.4 Hz), 126.2 (d, *J* = 3.1 Hz), 124.6 (d, *J* = 4.1 Hz), 63.4 (d, *J* = 6.2 Hz), 63.2 (d, *J* = 7.4 Hz), 49.7 (d, *J* = 152.0 Hz), 16.5 – 16.4 (m). **^{31}P-NMR** (122 MHz, $CDCl_3$): δ 17.28. **HRMS** (ESI): *m/z* calcd for $C_{19}H_{20}O_4N_2ClNaP$ $[M^++Na]$ 429.0741, found 429.0735. **HPLC** (Chiralpak IB, *n*-hexane/propan-2-ol 90:10, flow 1 mL/min) t_R 11.4 min (minor) and 13.1 min (major).

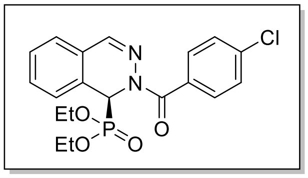

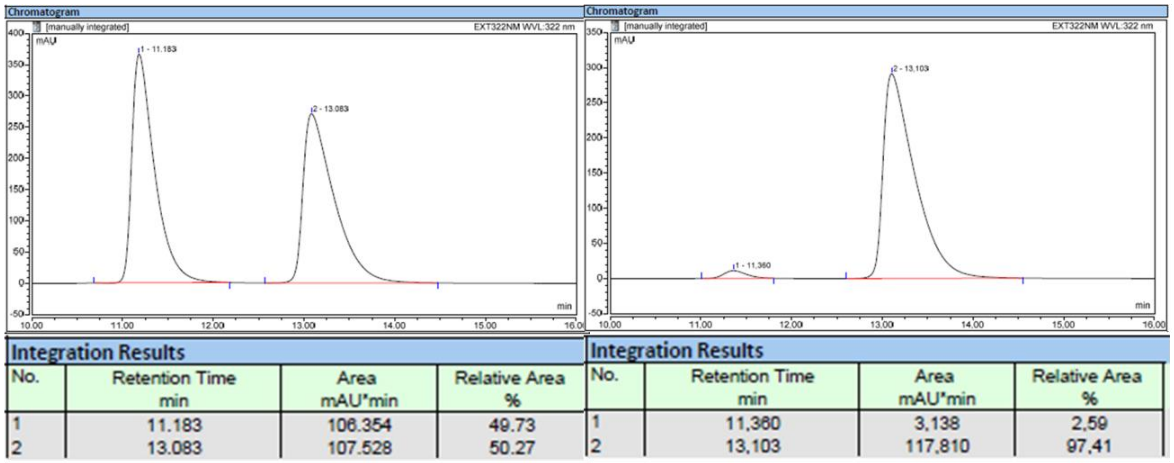

Integration Results

No.	Retention Time min	Area mAU*min	Relative Area %
1	11.183	106.354	49.73
2	13.083	107.528	50.27

Integration Results

No.	Retention Time min	Area mAU*min	Relative Area %
1	11,360	3,138	2,59
2	13,103	117,810	97,41

Crystallization of (*S*)-**11aA** by slow diffusion of *n*-pentane in a solution of (*S*)-**11aA** in DCM afforded crystals which are suitable for X-ray analysis. Mp = 93-95 °C.

Crystal Data for (*S*)-11Aa

Low-temperature diffraction data were collected on a Bruker D8 Quest APEX-III single crystal diffractometer with a Photon III detector and a IμS 3.0 microfocus X-ray source at the Instituto de Investigaciones Químicas, Sevilla. Data were collected by means of ω and φ scans using monochromatic radiation λ(Mo Kα1) = 0.71073 Å. The diffraction images collected were processed and scaled using APEX-4 v2021.4-0 software. The structures were solved with SHELXT and was refined against F2 on all data by full-matrix least squares with SHELXL [1], using Olex2 [2] as graphical interface. All non-hydrogen atoms were refined anisotropically. Hydrogen atoms were included in the model at geometrically calculated positions and refined using a riding model, unless otherwise noted. The isotropic displacement parameters of all hydrogen atoms were fixed to 1.2 times the U value of the atoms to which they are linked (1.5 times for methyl groups).

$C_{19}H_{20}ClN_2O_4P$ (*M* =406.79 g/mol):
Space group: C 1 2 1
Hall group: C 2y
a = 23.3542(19) Å,
b = 7.3837(6) Å,
c = 12.1678(9) Å,
α = 90°,
β = 1114.438(3)°,
γ = 90°,
V = 1910.2(3) Å^3,
Z = 1, *T* = 193.00 K,
μ(MoKα) = 0.312 mm^{-1},
Dcalc = 1.414 g/cm^3,
Nref = 4729,
R (reflections) = 0.536 (4163), s = 1.013, Npar = 246
wR2 (reflections) = 0.1306 (4729).

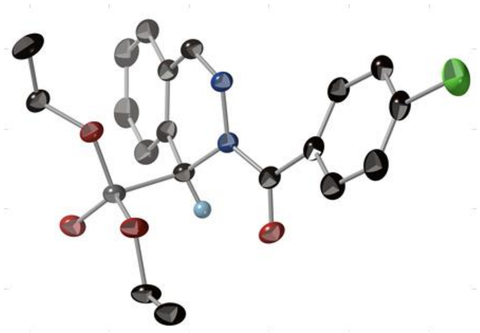

Figure S1. ORTEP plot of (*S*)-**11aA** with thermal ellipsoids set at the 50% probability level. Hydrogen atoms are omitted for clarity (except stereogenic centers).

Diethyl (*R*)-[2-(4-chlorobenzoyl)-1,2-dihydrophthalazin-1-yl]phosphonate [(*R*)-11aA]: Following the general procedure **11**, employing *p*-chlorobenzoyl chloride (26 µL, 0.2 mmol) as acylating reagent, and *ent*-**VII** (7 mg, 0.01 mmol, 5 mol%), (*R*)-**11aA** was obtained after purification by flash chromatography (*n*-hexane/EtOAc 1/1) as a white solid (72 mg, 88%, 95% ee). **[α]D[26] =** –677.2 (*c* 1, $CHCl_3$).

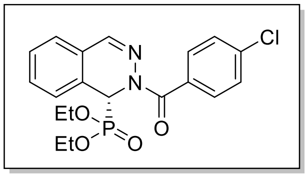

1 mmol scale: In a flame-dried Schlenk flask, *p*-chlorobenzoyl chloride (128 µL, 1 mmol) was added to a solution of phthalazine (**1a**) (133 mg, 1 mmol) in freshly distilled anhydrous MTBE (10 mL, 0.1 M) at 0 ºC. The resulting suspension was stirred for 1 h at room temperature. Then, catalyst *ent*-**VII** (33 mg, 0.05 mmol, 5 mol%) was added, and the reaction was cooled to −78 ºC (dry ice/acetone bath). *Tert*-butyldimethylsilyl diethyl phosphite (**2A**) (300 µL, 1.1 mmol) was added, and the reaction mixture was stirred for 18 h and allowed to warm slowly to room temperature during that time. Then, the solvent was removed under reduced pressure, and the residue was purified by flash chromatography (*n*-hexane/EtOAc 1/1) to afford (*R*)-**11aA** as a white solid (348 mg, 86%, 93% ee). **HPLC** (Chiralpak IB, *n*-hexane/propan-2-ol 90:10, flow 1 mL/min) t_R 11.1 min (major) and 13.5 min (minor).

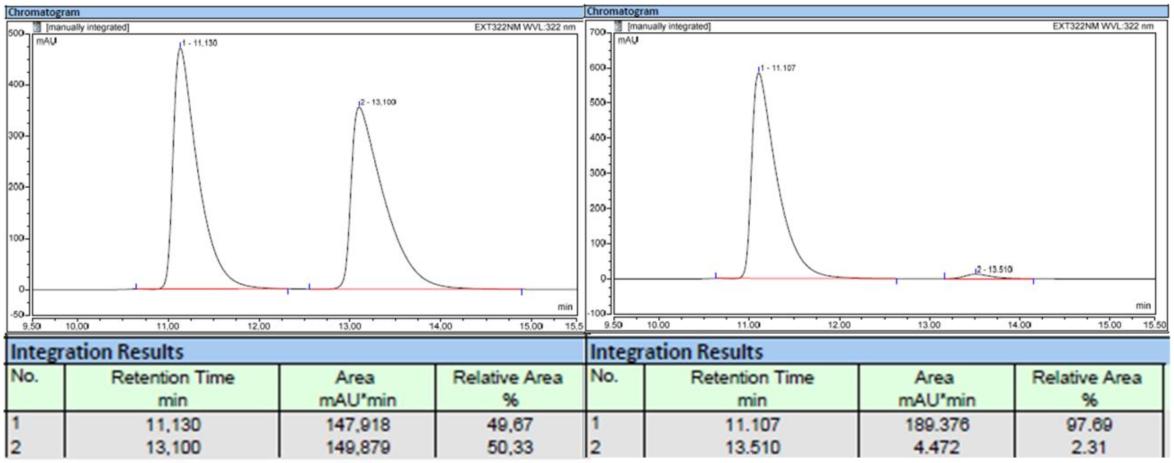

Integration Results

No.	Retention Time min	Area mAU*min	Relative Area %
1	11.130	147.918	49.67
2	13.100	149.879	50.33

Integration Results

No.	Retention Time min	Area mAU*min	Relative Area %
1	11.107	189.376	97.69
2	13.510	4.472	2.31

Diethyl (*S*)-[2-(2-chlorobenzoyl)-1,2-dihydrophthalazin-1-yl]phosphonate [(*S*)-12aA]: Following the general procedure **11**, employing *o*-chlorobenzoyl chloride (27 µL, 0.2 mmol) as acylating reagent, (*S*)-**12aA** was obtained after purification by flash chromatography (*n*-hexane/EtOAc 1/1) as a colorless oil (59 mg, 73%, 84% ee). **[α]D[29] =** +512.1 (*c* 1, $CHCl_3$). **^{1}H-NMR** (300 MHz, $CDCl_3$): δ 7.53 – 7.48 (m, 2H), 7.45 – 7.29 (m, 6H), 7.28 – 7.25 (m, 1H), 6.44 (d, *J* = 16.3 Hz, 1H), 4.25 – 4.16 (m, 2H), 4.06 – 3.81 (m, 2H), 1.32 (t, *J* = 7.2 Hz, 3H), 1.13 (t, *J* = 7.1 Hz, 3H). **^{13}C-NMR** (75.5 MHz, $CDCl_3$): δ 168.2, 144.7, 135.8, 132.3 (d, *J* = 2.9 Hz), 131.2, 130.4, 129.5, 129.2 (d, *J* = 3.5 Hz), 128.5, 128.0 (d, *J* = 4.9 Hz), 127.3 (d, *J* = 2.8 Hz), 126.6, 126.4 (d, *J* = 3.1 Hz), 124.6 (d, *J* = 4.2 Hz), 63.6 (d, *J* = 6.0 Hz), 63.2 (d, *J* = 7.6 Hz), 49.4 (d, *J* = 152.5 Hz), 16.6 – 16.4 (m). **^{31}P-NMR** (122 MHz, $CDCl_3$): δ 16.76. **HRMS** (ESI): *m/z* calcd for $C_{19}H_{20}O_4N_2ClNaP$ $[M^+ + Na]$ 429.0746, found 429.0741. **HPLC** (Chiralpak IB, *n*-hexane/propan-2-ol 90:10, flow 1 mL/min) t_R 13.1 min (minor) and 16.0 min (major).

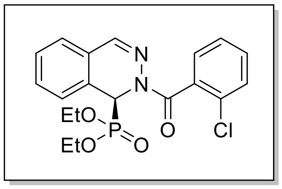

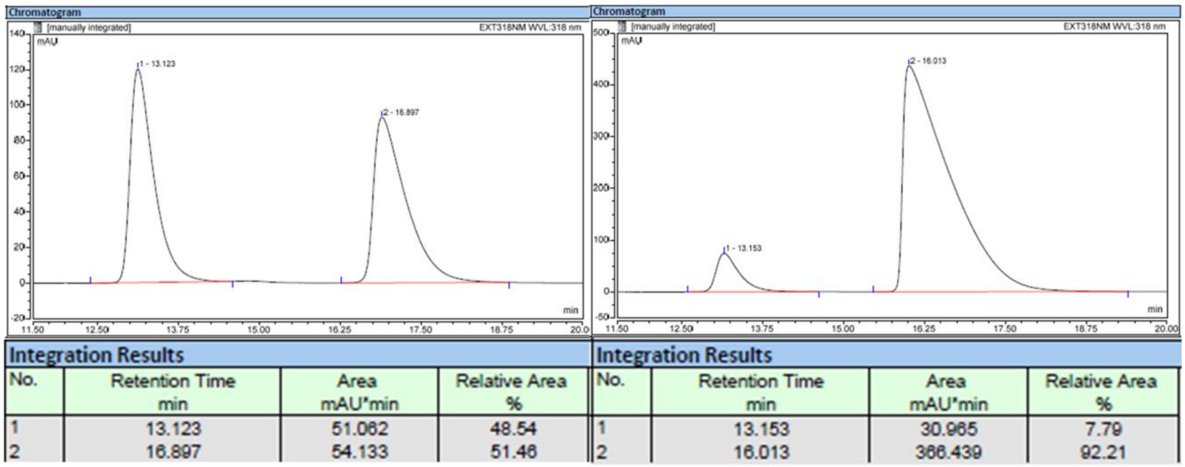

Integration Results

No.	Retention Time min	Area mAU*min	Relative Area %
1	13.123	51.062	48.54
2	16.897	54.133	51.46

Integration Results

No.	Retention Time min	Area mAU*min	Relative Area %
1	13.153	30.965	7.79
2	16.013	366.439	92.21

Diethyl (*S*)-[2-(3-chlorobenzoyl)-1,2-dihydrophthalazin-1-yl]phosphonate [(*S*)-13aA]: Following the general procedure **11**, employing *m*-chlorobenzoyl chloride (26 µL, 0.2 mmol) as acylating reagent, (*S*)-**13aA** was obtained after purification by flash chromatography (*n*-hexane/EtOAc 1/1) as a colorless oil (46 mg, 56%, 92% ee). **$[\alpha]_D^{29}$ =** +621.8 (*c* 1, $CHCl_3$). **^{1}H-NMR** (300 MHz, $CDCl_3$): δ 7.67 – 7.66 (m, 1H), 7.59 – 7.55 (m, 2H), 7.53 – 7.48 (m, 1H), 7.43 – 7.38 (m, 3H), 7.36 – 7.27 (m, 2H), 6.45 (d, *J* = 16.5 Hz, 1H), 4.23 – 4.05 (m, 2H), 4.04 – 3.78 (m, 2H), 1.27 (t, *J* = 7.0 Hz, 3H), 1.13 (t, *J* = 7.1 Hz, 3H). **^{13}C-NMR** (75.5 MHz, $CDCl_3$): δ 168.3, 144.2, 136.1, 133.8, 132.3 (d, *J* = 2.8 Hz), 130.6, 129.5, 129.3 (d, *J* = 3.4 Hz), 129.1, 127.8 (d, *J* = 5.0 Hz), 127.6, 127.5 (d, *J* = 2.4 Hz), 126.3 (d, *J* = 3.0 Hz), 124.6 (d, *J* = 4.1 Hz), 63.5 (d, *J* = 6.3 Hz), 63.2 (d, *J* = 7.5 Hz), 49.7 (d, *J* = 152.1 Hz), 16.5 – 16.4 (m). **^{31}P-NMR** (122 MHz, $CDCl_3$): δ 17.17. **HRMS** (ESI): *m/z* calcd for $C_{19}H_{20}O_4N_2ClNaP$ $[M^++Na]$ 429.0741, found 429.0745. **HPLC** (Chiralpak IB, *n*-hexane/propan-2-ol 90:10, flow 1 mL/min) t_R 10.8 min (minor) and 12.9 min (major).

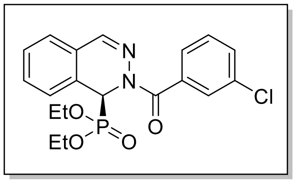

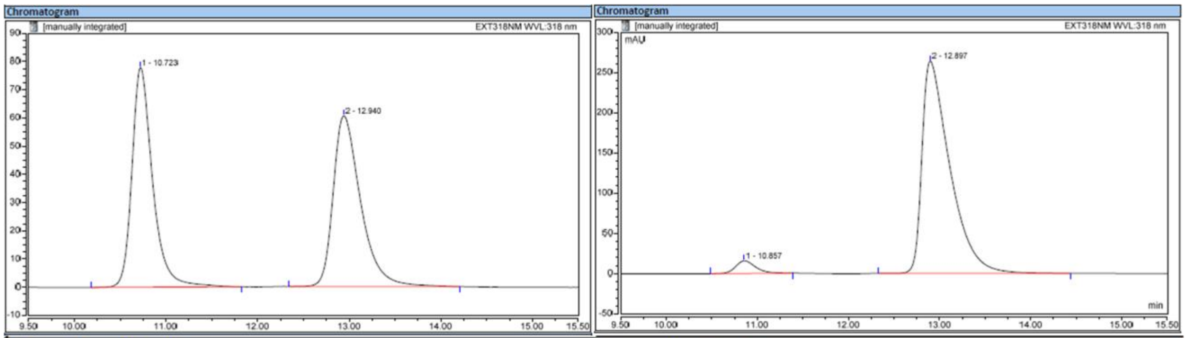

Integration Results

No.	Retention Time min	Area mAU*min	Relative Area %
1	10.723	20.380	49.19
2	12.940	21.053	50.81

Integration Results

No.	Retention Time min	Area mAU*min	Relative Area %
1	10.857	3.963	4.07
2	12.897	93.520	95.93

Diethyl (*S*)-[2-(2-naphthoyl)-1,2-dihydrophthalazin-1-yl]phosphonate [(*S*)-14aA]: Following the general procedure **11**, employing 2-naphthoyl chloride (38 mg, 0.2 mmol) as acylating reagent, (*S*)-**14aA** was obtained after purification by flash chromatography (*n*-hexane/EtOAc 1/1) as a colorless oil (43 mg, 48%, 86% ee). **$[\alpha]_D^{28}$ =** +497.1 (*c* 1, $CHCl_3$). **^{1}H-NMR** (300 MHz, $CDCl_3$): δ 8.24 (s, 1H), 7.92 – 7.76 (m, 4H), 7.61 (s, 1H), 7.57 – 7.39 (m, 5H), 7.31 – 7.29 (m, 1H), 6.58 (d, *J* = 16.5 Hz,

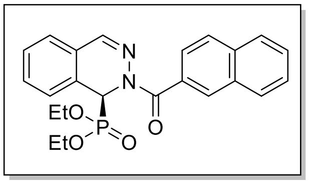

1H), 4.24 – 4.15 (m, 2H), 4.06 – 3.83 (m, 2H), 1.30 (t, *J* = 7.0 Hz, 3H), 1.15 (t, *J* = 7.0 Hz, 3H). **^{13}C-NMR** (75.5 MHz, $CDCl_3$): δ 169.8, 143.9, 134.4, 132.5, 132.2 (d, *J* = 2.8 Hz), 131.8, 130.1, 129.2 (d, *J* = 3.4 Hz), 129.0, 127.9, 127.8, 127.7 (d, *J* = 2.3 Hz), 127.5, 127.3, 126.5, 126.21, 126.16, 124.8 (d, *J* = 4.1 Hz), 63.5 (d, *J* = 6.3 Hz), 63.3 (d, *J* = 7.5 Hz), 49.8 (d, *J* = 152.2 Hz), 16.6 – 16.4 (m). **^{31}P-NMR** (122 MHz, $CDCl_3$): δ 17.47. **HRMS** (ESI): *m/z* calcd for $C_{23}H_{23}O_4N_2NaP$ [M^++Na] 445.1288, found 445.1280. **HPLC** (Chiralpak IA, *n*-hexane/propan-2-ol 85:15, flow 1 mL/min) t_R 15.4 min (minor) and 24.3 min (major).

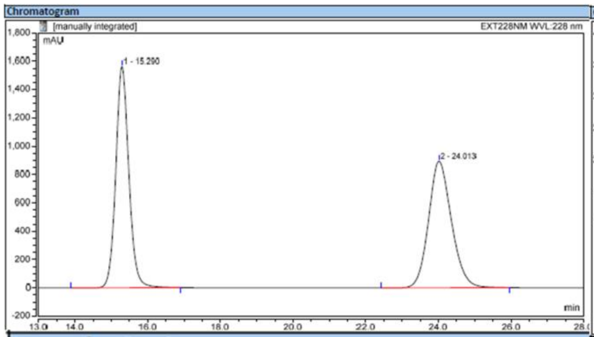

Integration Results

No.	Retention Time min	Area mAU*min	Relative Area %
1	15.290	651.004	49.92
2	24.013	653.187	50.08

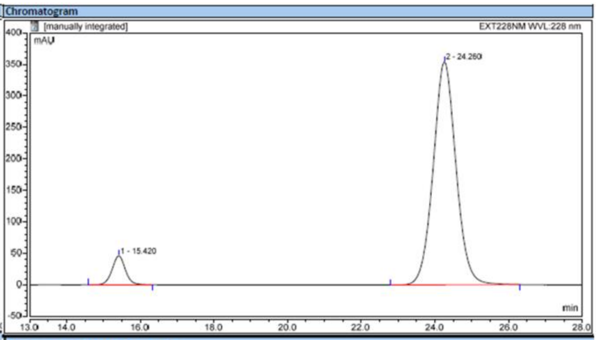

Integration Results

No.	Retention Time min	Area mAU*min	Relative Area %
1	15.420	19.000	6.97
2	24.260	253.675	93.03

Diethyl (*S*)-[2-(1-naphthoyl)-1,2-dihydrophthalazin-1-yl]phosphonate [(*S*)-15aA]: Following the general procedure **11**, employing 1-naphthoyl chloride (30 µL, 0.2 mmol) as acylating reagent, (*S*)-**15aA** was obtained after purification by flash chromatography (*n*-hexane/EtOAc 1/1) as a colorless oil (55 mg, 62%, 87% ee). **$[\alpha]_D^{28}$ =** +432.4 (*c* 1, $CHCl_3$). **^{1}H-NMR** (300 MHz, $CDCl_3$): δ 8.01 – 7.85 (m, 3H), 7.56 – 7.47 (m, 6H), 7.42 – 7.38 (m, 2H), 7.25 – 7.22 (m, 1H), 6.65 (d, *J* = 16.3 Hz, 1H), 4.32 – 4.23 (m, 2H), 4.15 – 3.87 (m, 2H), 1.35 (t, *J* = 7.1 Hz, 3H), 1.18 (t, *J* = 7.1 Hz, 3H). **^{13}C-NMR** (75.5 MHz, $CDCl_3$): δ 170.7, 144.1, 133.7, 133.4, 132.3 (d, *J* = 2.8 Hz), 130.2, 129.7, 129.3 (d, *J* = 3.4 Hz), 128.4, 128.0 (d, *J* = 4.9 Hz), 127.4 (d, *J* = 2.7 Hz), 126.8, 126.3 (d, *J* = 3.1 Hz), 126.2, 125.6, 125.3, 124.9, 124.7 (d, *J* = 4.1 Hz), 63.7 (d, *J* = 6.0 Hz), 63.2 (d, *J* = 7.6 Hz), 49.5 (d, *J* = 152.2 Hz), 16.6 – 16.4 (m). **^{31}P-NMR** (122 MHz, $CDCl_3$): δ 17.30. **HRMS** (ESI): *m/z* calcd for $C_{23}H_{23}O_4N_2NaP$ [M^++Na] 445.1288, found 445.1281. **HPLC** (Chiralpak IA, *n*-hexane/propan-2-ol 85:15, flow 1 mL/min) t_R 12.9 min (minor) and 14.8 min (major).

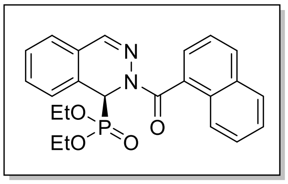

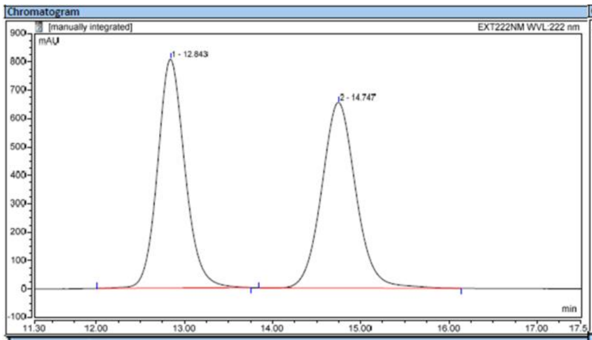

Integration Results

No.	Retention Time min	Area mAU*min	Relative Area %
1	12.843	290.629	49.74
2	14.747	293.631	50.26

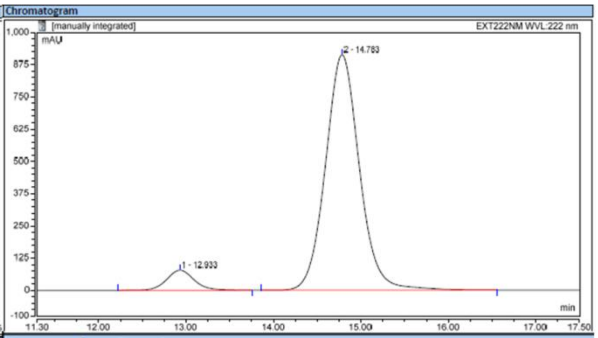

Integration Results

No.	Retention Time min	Area mAU*min	Relative Area %
1	12.933	28.376	6.46
2	14.783	410.576	93.54

12. Kinetic profile.

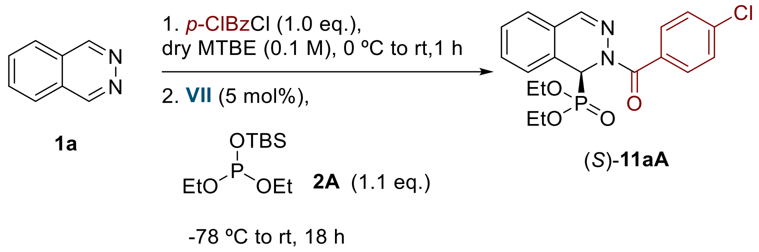

In a flame-dried Schlenk flask, *p*-chlorobenzoyl chloride (13 µL, 0.1 mmol) was added to a solution of phthalazine (**1a**) (13 mg, 0.1 mmol) in freshly distilled MTBE (1 mL, 0.1 M) at 0 ºC. The resulting suspension[1] was stirred for 1 h at room temperature. Then, for enantioselective reactions, catalyst **VII** (3 mg, 0.005 mmol, 5 mol%) was added, and the reaction was cooled to −78 ºC (dry ice/acetone bath). *Tert*-butyldimethylsilyl diethyl phosphite (**2A**) (30 µL, 0.11 mmol) was added, and the reaction mixture was stirred for the indicated time and allowed to warm slowly to room temperature. The reaction was quenched by the addition of H_2O (0.5 mL), extracted with DCM (2 x 1 mL), dried over $MgSO_4$, and the solvent was removed under reduced pressure. Data are collected in **Table S4**.

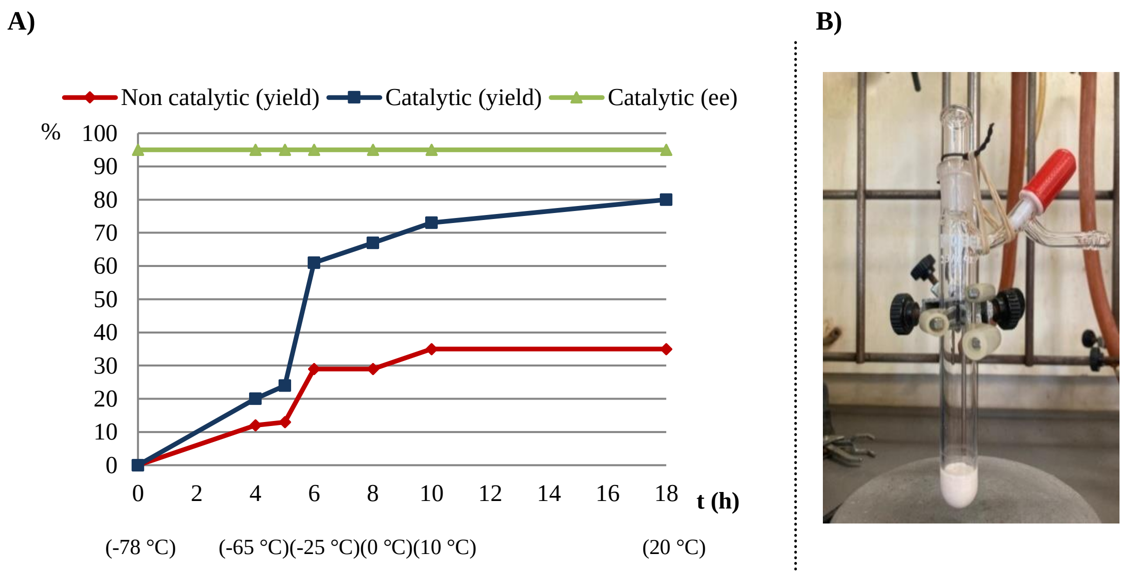

Figure S2. (A) Kinetic profile of model reaction. **(B)** Picture of model reaction after the acylation step.

[1]After the acylation step, a highly insoluble *N*-acyl phthalazinium chloride was observed, which became solubilized as the reaction progressed.

Table S4. Kinetic profile of model reaction.[a]

Entry	Time (h)	Temperature (ºC)	Non-catalyzed yield (%)[b]	Catalyzed yield (%)[b]	ee (%)[c]
**1**	4	−75 ºC	12	20	95
**2**	5	−50 ºC	13	24	95
**3**	6	−25 ºC	29	61	95
**4**	8	0 ºC	29	67	95
**5**	10	+10 ºC	35	73	95
**6**	18	+20 ºC	35	80	95

[a]Reactions were stopped after 4, 5, 6, 8, 10 and 18 h. [b]NMR yields were determined by ^{1}H-NMR analysis of the crude reaction mixture using mesitylene (0.1 mmol, 14 µL) as the internal standard. [c]Enantiomeric ratios were determined by HPLC analysis after the product was isolated by semipreparative TLC (*n*-hexane/EtOAc 1/1) **[**(Chiralpak IB, *n*-hexane/propan-2-ol 90:10, flow 1 mL/min) t_R 11.4 min (minor) and 13.1 min (major)].

13. General procedure for the asymmetric dearomatization reaction of benzodiazines derivatives 1.

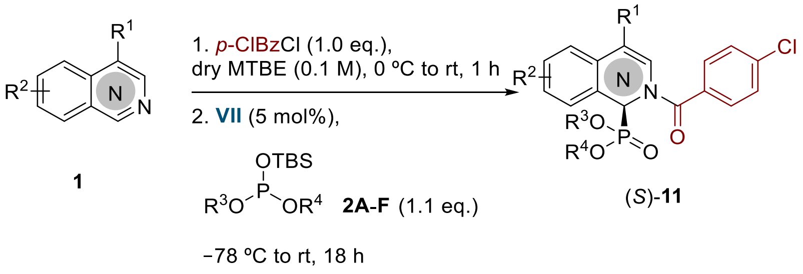

In a flame-dried Schlenk flask, *p*-chlorobenzoyl chloride (26 µL, 0.2 mmol) was added to a solution of the corresponding diazarene derivative **1** (0.2 mmol) in freshly distilled anhydrous MTBE (2 mL, 0.1 M) at 0 ºC. The resulting suspension was stirred for 1 h at room temperature. Then, catalyst **VII** (7 mg, 0.01 mmol, 5 mol%) was added, and the reaction was cooled to −78 ºC (dry ice/acetone bath). *Tert*-butyldimethylsilyl phosphite **2A-F** (0.22 mmol) was added, and the reaction mixture was stirred for 18 h and allowed to warm slowly to room temperature during that time. Then, the solvent was removed under reduced pressure, and the residue was purified by flash chromatography to afford the corresponding products (*S*)-**11**. Enantiomeric ratios were determined by HPLC analysis.

Racemic products were prepared without catalyst following the general procedure described above.

Dimethyl (*S*)-[2-(4-chlorobenzoyl)-1,2-dihydrophthalazin-1-yl]phosphonate [(*S*)-11aB]: Following the general procedure **13**, starting from phthalazine (**1a**) (27 mg, 0.2 mmol) and *tert*-butyldimethylsilyl dimethyl phosphite (**2B**) (49 µL, 0.22 mmol), (*S*)-**11aB** was obtained after purification by flash chromatography (*n*-hexane/EtOAc 1/2) as a white solid (56 mg, 74%, 93% ee). **[α]$_D$^{28} =** +627.5 (*c* 1, $CHCl_3$). **^{1}H-NMR** (300 MHz, $CDCl_3$): δ 7.68 – 7.65 (m, 2H), 7.61 (s, 1H), 7.54 – 7.50 (m, 1H), 7.45 – 7.36 (m, 4H), 7.31 – 7.29 (m, 1H), 6.48 (d, *J* = 16.4 Hz, 1H), 3.77 (d, *J* = 10.8 Hz, 3H), 3.57 (d, *J* = 10.8 Hz, 3H). **^{13}C-NMR** (75.5 MHz, $CDCl_3$): δ 168.7, 144.0, 137.0, 132.6, 132.4 (d, *J* = 2.8 Hz), 131.1, 129.4 (d, *J* = 3.4 Hz), 128.1, 127.8 (d, *J* = 5.1 Hz), 127.2 (d, *J* = 2.6 Hz), 126.4 (d, *J* = 3.1 Hz), 124.5 (d, *J* = 4.1 Hz), 53.9 (d, *J* = 6.0 Hz), 53.5 (d, *J* = 7.5 Hz), 49.3 (d, *J* = 151.9 Hz). **^{31}P-NMR** (122 MHz, $CDCl_3$): δ 18.06. **HRMS** (ESI): *m/z* calcd for $C_{17}H_{16}O_4N_2ClNaP$ [M^++Na] 401.0428, found 401.0431. **HPLC** (Chiralpak IB, *n*-hexane/propan-2-ol85:15, flow 1 mL/min) t_R 12.2 min (minor) and 14.6 min (major).

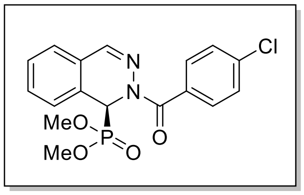

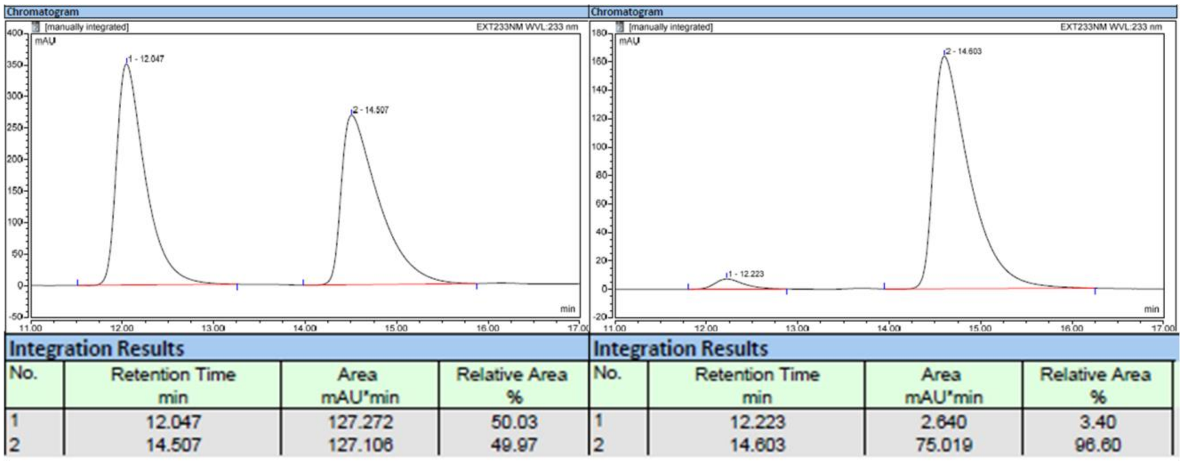

Integration Results

No.	Retention Time min	Area mAU*min	Relative Area %
1	12.047	127.272	50.03
2	14.507	127.106	49.97

Integration Results

No.	Retention Time min	Area mAU*min	Relative Area %
1	12.223	2.640	3.40
2	14.603	75.019	96.60

Dibutyl (*S*)-[2-(4-chlorobenzoyl)-1,2-dihydrophthalazin-1-yl]phosphonate [(*S*)-11aC]: Following the general procedure **13**, starting from phthalazine (**1a**) (27 mg, 0.2 mmol) and *tert*-butyldimethylsilyl dibutyl phosphite (**2C**) (73 µL, 0.22 mmol), (*S*)-**11aC** was obtained after purification by flash chromatography (*n*-hexane/EtOAc 1/1) as a colorless oil (77 mg, 83%, 91% ee). **[α]$_D$^{29} =** +551.2 (*c* 1, $CHCl_3$). **^{1}H-NMR** (300 MHz, $CDCl_3$): δ 7.67 – 7.63 (m, 2H), 7.56 (s, 1H), 7.51 – 7.46 (m, 1H), 7.41 – 7.35 (m, 4H), 7.28 – 7.25 (m, 1H), 6.46 (d, *J* = 16.6 Hz, 1H), 4.13 – 3.99 (m, 2H), 3.98 – 3.86 (m, 1H), 3.81 – 3.70 (m, 1H), 1.61 – 1.52 (m, 2H), 1.48 – 1.39 (m, 2H), 1.37 – 1.27 (m, 2H), 1.25 – 1.15 (m, 2H), 0.89 – 0.78 (m, 6H). **^{13}C-NMR** (75.5 MHz, $CDCl_3$): δ 168.5, 143.9, 136.8, 132.7, 132.2 (d, *J* = 2.8 Hz), 131.1, 129.2 (d, *J* = 3.4 Hz), 128.0, 127.8 (d, *J* = 5.1 Hz), 127.6 (d, *J* = 2.5 Hz), 126.1 (d, *J* = 3.1 Hz), 124.6 (d, *J* = 4.1 Hz), 66.9 (d, *J* = 6.6 Hz), 66.6 (d, *J* = 7.7 Hz), 49.7 (d, *J* = 152.1 Hz), 32.6 – 32.4 (m), 18.6 (d, *J* = 7.8 Hz), 13.6 (d, *J* = 5.8 Hz). **^{31}P-NMR** (122 MHz, $CDCl_3$): δ 17.46. **HRMS** (ESI): *m/z* calcd for $C_{23}H_{28}O_4N_2ClNaP$ [M^++Na] 485.1367, found 485.1373. **HPLC** (Chiralpak IB, *n*-hexane/propan-2-ol 90:10, flow 1 mL/min) t_R 7.3 min (minor) and 8.9 min (major).

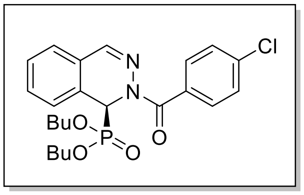

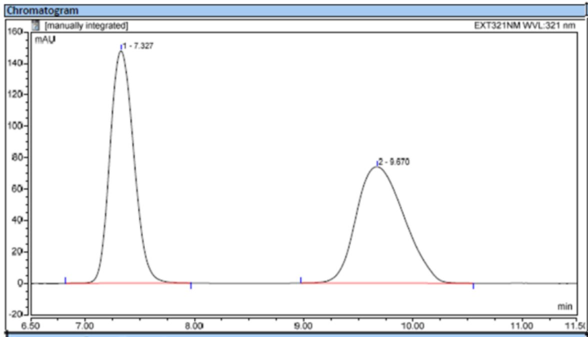

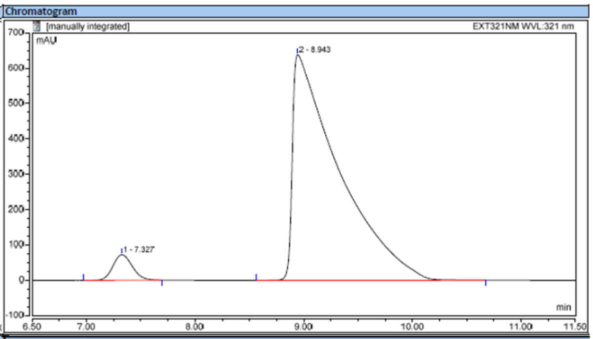

Integration Results

No.	Retention Time min	Area mAU*min	Relative Area %
1	7.327	37.342	49.88
2	9.670	37.528	50.12

Integration Results

No.	Retention Time min	Area mAU*min	Relative Area %
1	7.327	14.812	4.49
2	8.943	315.291	95.51

Diisopropyl (*S*)-[2-(4-chlorobenzoyl)-1,2-dihydrophthalazin-1-yl]phosphonate [(*S*)-11aD]: Following the general procedure **13**, starting from phthalazine (**1a**) (27 mg, 0.2 mmol) and *tert*-butyldimethylsilyl diisopropyl phosphite (**2D**) (70 μL, 0.22 mmol), (*S*)-**11aD** was obtained after purification by flash chromatography (*n*-hexane/EtOAc 1/1) as a white solid (64 mg, 74%, 93% ee). **$[\alpha]_D^{28}$ =** +551.4 (*c* 1, $CHCl_3$). **^{1}H-NMR** (300 MHz, $CDCl_3$): δ 7.68 – 7.65 (m, 2H), 7.56 (s, 1H), 7.50 – 7.46 (m, 1H), 7.41 – 7.34 (m, 4H), 7.28 – 7.25 (m, 1H), 6.45 (d, *J* = 17.1 Hz, 1H), 4.74 – 4.60 (m, 1H), 4.58 – 4.43 (m, 1H), 1.29 – 1.20 (m, 9H), 1.01 (d, *J* = 6.2 Hz, 3H). **^{13}C-NMR** (75.5 MHz, $CDCl_3$): δ 168.4, 144.2, 136.7, 132.9, 132.0 (d, *J* = 2.7 Hz), 131.1, 129.1 (d, *J* = 3.4 Hz), 128.0, 127.9 (d, *J* = 2.1 Hz), 127.7 (d, *J* = 5.1 Hz), 126.0 (d, *J* = 3.1 Hz), 124.8 (d, *J* = 4.1 Hz), 72.4 (d, *J* = 7.3 Hz), 72.2 (d, *J* = 7.9 Hz), 50.4 (d, *J* = 155.2 Hz), 29.33 – 29.28 (m), 23.9 (d, *J* = 5.8 Hz), 23.6 (d, *J* = 5.5 Hz). **^{31}P-NMR** (122 MHz, $CDCl_3$): δ 15.17. **HRMS** (ESI): *m/z* calcd for $C_{21}H_{24}O_4N_2ClNaP$ $[M^+ + Na]$ 457.1054, found 457.1060. **HPLC** (Chiralpak IB, *n*-hexane/propan-2-ol 90:10, flow 1 mL/min) t_R 7.2 min (minor) and 9.1 min (major).

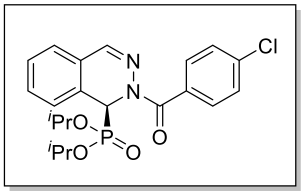

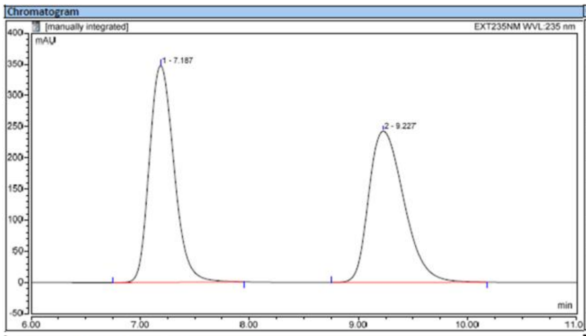

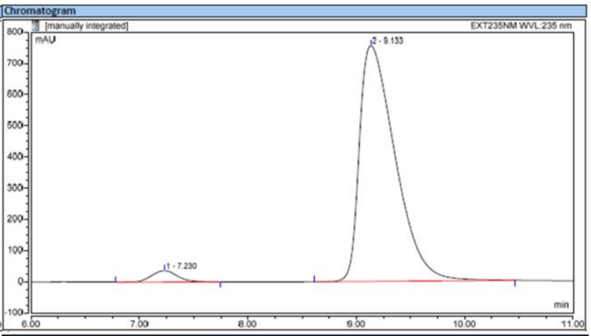

Integration Results

No.	Retention Time min	Area mAU*min	Relative Area %
1	7.187	90.838	50.09
2	9.227	90.509	49.91

Integration Results

No.	Retention Time min	Area mAU*min	Relative Area %
1	7.230	9.997	3.35
2	9.133	288.016	96.65

Dibenzyl (*S*)-[2-(4-chlorobenzoyl)-1,2-dihydrophthalazin-1-yl]phosphonate [(*S*)-11aE]: Following the general procedure **13**, starting from phthalazine (**1a**) (27 mg, 0.2 mmol) and *tert*-butyldimethylsilyl dibenzyl phosphite (**2E**) (82 µL, 0.22 mmol), (*S*)-**11aE** was obtained after purification by flash chromatography (*n*-hexane/EtOAc 1/1) as a white solid (96 mg, 90%, 83% ee). **[α]D**^{24} = +420.8 (*c* 1, $CHCl_3$). **^{1}H-NMR** (300 MHz, $CDCl_3$): δ 7.54 – 7.48 (m, 4H), 7.45 – 7.39 (m, 2H), 7.36 – 7.26 (m, 10H), 7.24 – 7.21 (m, 1H), 7.18 – 7.13 (m, 2H), 6.62 (d, *J* = 16.3 Hz, 1H), 5.09 (d, *J* = 7.3 Hz, 2H), 4.94 – 4.77 (m, 2H). **^{13}C-NMR** (75.5 MHz, $CDCl_3$): δ 168.6, 144.1, 136.8, 136.0 (d, *J* = 6.8 Hz), 135.9 (d, *J* = 6.1 Hz), 132.6, 132.3 (d, *J* = 2.8 Hz), 131.1, 129.3 (d, *J* = 3.5 Hz), 128.6, 128.51, 128.49, 128.4, 128.2, 128.0, 127.9, 127.8 (d, *J* = 5.1 Hz), 127.2 (d, *J* = 2.4 Hz), 126.3 (d, *J* = 3.2 Hz), 124.6 (d, *J* = 4.1 Hz), 68.6 (d, *J* = 6.2 Hz), 68.2 (d, *J* = 7.5 Hz), 50.0 (d, *J* = 151.3 Hz). **^{31}P-NMR** (122 MHz, $CDCl_3$): δ 18.19. **HRMS** (ESI): *m/z* calcd for $C_{29}H_{24}O_4N_2ClNaP$ [M^++Na] 553.1054, found 553.1064. **HPLC** (Chiralpak IB, *n*-hexane/propan-2-ol 90:10, flow 1 mL/min) t_R 15.0 min (minor) and 23.7 min (major).

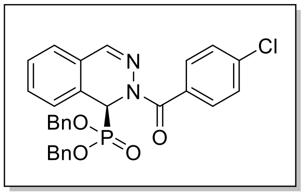

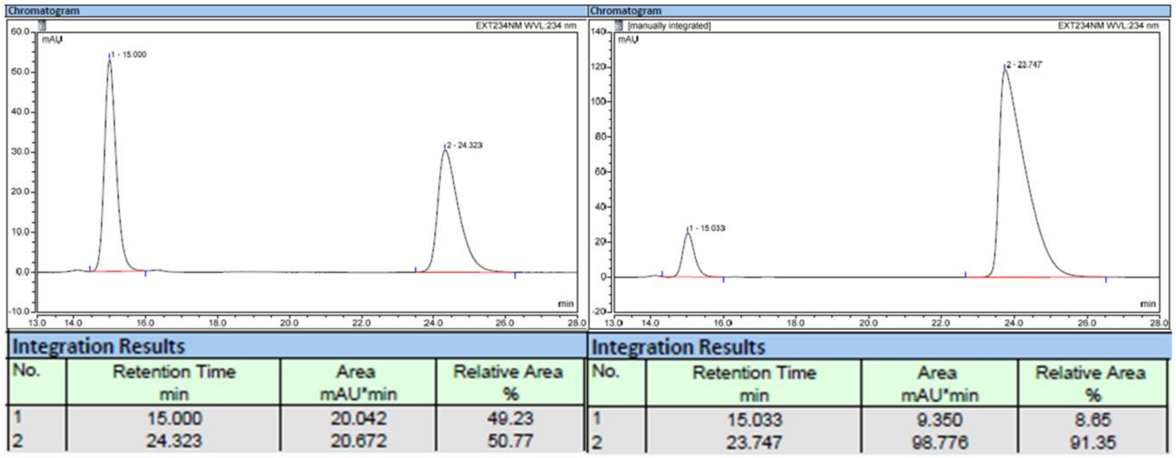

Integration Results

No.	Retention Time min	Area mAU*min	Relative Area %
1	15.000	20.042	49.23
2	24.323	20.672	50.77

Integration Results

No.	Retention Time min	Area mAU*min	Relative Area %
1	15.033	9.350	8.65
2	23.747	98.776	91.35

Cyclohexyl methyl (*R or S*)-[(*S*)-2-(4-chlorobenzoyl)-1,2-dihydrophthalazin-1-yl]phosphonate [(*R/S,S*)-11aF]: Following the general procedure **13**, starting from phthalazine (**1a**) (27 mg, 0.2 mmol) and *tert*-butyldimethylsilyl cyclohexyl methyl phosphite (**2F**) (65 µL, 0.22 mmol), (*R/S,S*)-**11aF** was obtained after purification by flash chromatography (*n*-hexane/EtOAc 1/1) as a colorless oil (66 mg, 91%; combined yield of the mixture of diastereoisomers in a ~1.3:1 ratio, 90% ee, 90% ee). **[α]D**^{23} = +385.1 (*c* 1, $CHCl_3$). **^{1}H-NMR** (300 MHz, $CDCl_3$): The compound exists as a ~1.3:1 mixture of diastereoisomers. *Signals corresponding to both diastereoisomers:* δ 7.69 – 7.27 (m, 9H), 6.53 – 6.42 (m, 1H), 4.49 – 4.26 (m, 1H), 3.78 – 3.55 (m, 3H), 1.91 – 1.15 (m, 10 H). **^{13}C-NMR** (75.5 MHz, $CDCl_3$): *Signals corresponding to both diastereoisomers:* δ 168.7, 168.6, 144.2, 144.0, 136.9, 132.82, 132.75, 132.3 (d, *J* = 2.8 Hz), 132.2 (d, *J* = 2.8 Hz), 131.2, 131.1, 129.3 (d, *J* = 3.4 Hz), 129.2 (d, *J* = 3.5 Hz), 128.09, 128.08, 127.9 – 127.8 (m), 127.6 (d, *J* = 2.0 Hz), 126.3 (d, *J* = 3.1 Hz), 126.2 (d, *J* = 3.0 Hz), 124.7 (d, *J* = 4.0 Hz), 53.8 (d, *J* = 5.8 Hz), 53.6 (d, *J* = 7.2 Hz), 49.9 (d, *J* = 154.3 Hz), 49.8 (d, *J* = 152.6 Hz), 33.9 (d, *J* = 2.6 Hz), 33.7 (d, *J* = 3.1 Hz), 33.4 (d, *J* = 5.3 Hz), 33.1 (d, *J* = 5.1 Hz), 25.16, 25.12, 23.5, 23.23, 23.17. **^{31}P-NMR** (122 MHz, $CDCl_3$): *Signal corresponding to the major diastereoisomer:* δ 16.98. *Signal corresponding to the minor diastereoisomer:* δ 18.19. **HRMS** (ESI): *m/z* calcd for $C_{22}H_{24}O_4N_2ClNaP$ [M^++Na] 469.1054, found 469.1047. **HPLC** (Chiralpak IB, *n*-hexane/propan-2-ol 90:10, flow 1 mL/min) t_R 9.7 min (minor) and 13.8 min (major); 10.7 min (minor) and 12.3 min (major).

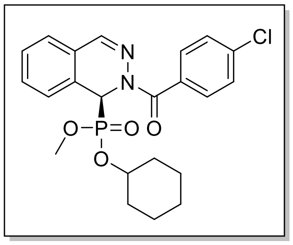

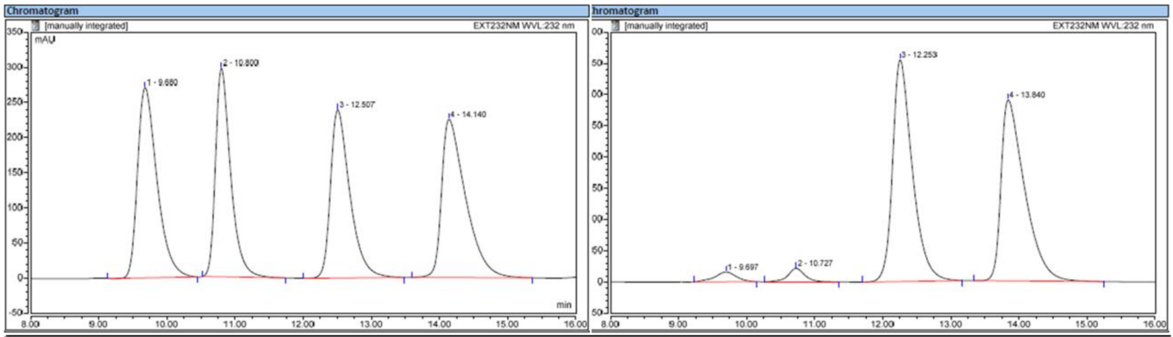

Integration Results

No.	Retention Time min	Area mAU*min	Relative Area %
1	9.680	89.438	26.58
2	10.800	76.654	22.78
3	12.507	78.873	23.44
4	14.140	91.585	27.21

Integration Results

No.	Retention Time min	Area mAU*min	Relative Area %
1	9.697	5.714	2.31
2	10.727	6.177	2.50
3	12.253	119.326	48.22
4	13.840	116.233	46.97

Diethyl (*S*)-[2-(4-chlorobenzoyl)-6,7-dimethyl-1,2-dihydrophthalazin-1-yl]phosphonate [(*S*)-11bA]:

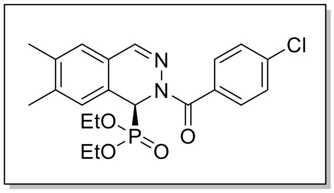

Following the general procedure **13**, starting from 6,7-dimethylphthalazine (**1b**) (32 mg, 0.2 mmol) and *tert*-butyldimethylsilyl diethyl phosphite (**2A**) (60 µL, 0.22 mmol), (*S*)-**11bA** was obtained after purification by flash chromatography (*n*-hexane/EtOAc 1/1) as a white solid (70 mg, 80%, 97% ee). **$[\alpha]_D^{23}$ =** +609.8 (*c* 1, $CHCl_3$). **^{1}H-NMR** (300 MHz, $CDCl_3$): δ 7.66 – 7.62 (m, 2H), 7.52 (s, 1H), 7.39 – 7.34 (m, 2H), 7.18 – 7.17 (m, 1H), 7.05 (s, 1H), 6.40 (d, *J* = 16.0 Hz, 1H), 4.21 – 4.09 (m, 2H), 4.04 – 3.78 (m, 2H), 2.31 – 2.27 (m, 6H), 1.27 (t, *J* = 7.1 Hz, 3H), 1.13 (t, *J* = 7.1 Hz, 3H). **^{13}C-NMR** (75.5 MHz, $CDCl_3$): δ 168.6, 144.4, 141.8 (d, *J* = 2.8 Hz), 137.9 (d, *J* = 3.7 Hz), 136.7, 133.0, 131.0, 128.8 (d, *J* = 4.9 Hz), 128.0, 127.3 (d, *J* = 3.2 Hz), 124.9 (d, *J* = 2.4 Hz), 122.5 (d, *J* = 4.2 Hz), 63.3 (d, *J* = 6.2 Hz), 63.1 (d, *J* = 7.5 Hz), 49.4 (d, *J* = 152.1 Hz), 20.2, 19.6, 16.5 – 16.4 (m). **^{31}P-NMR** (122 MHz, $CDCl_3$): δ 17.62. **HRMS** (ESI): *m/z* calcd for $C_{21}H_{24}O_4N_2ClNaP$ $[M^+ + Na]$ 457.1054, found 457.1047. **HPLC** (Chiralpak IA, *n*-hexane/propan-2-ol 80:20, flow 1 mL/min) t_R 13.9 min (minor) and 22.8 min (major).

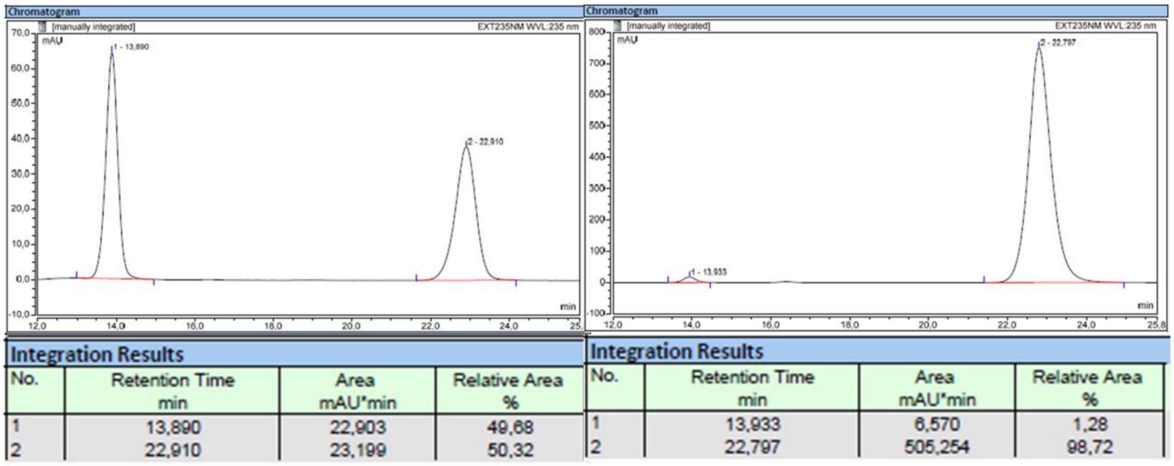

Integration Results

No.	Retention Time min	Area mAU*min	Relative Area %
1	13,890	22,903	49,68
2	22,910	23,199	50,32

Integration Results

No.	Retention Time min	Area mAU*min	Relative Area %
1	13,933	6,570	1,28
2	22,797	505,254	98,72

Diethyl (*R*)-[2-(4-chlorobenzoyl)-6,7-dimethyl-1,2-dihydrophthalazin-1-yl]phosphonate [(*R*)-11bA]:

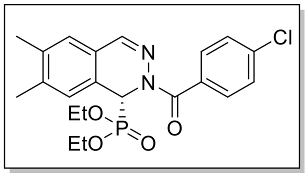

Following the general procedure **13**, starting from 6,7-dimethylphthalazine (**1b**) (32 mg, 0.2 mmol), *tert*-butyldimethylsilyl diethyl phosphite (**2A**) (60 µL, 0.22 mmol) and *ent*-**VII** (7 mg, 0.01 mmol, 5 mol%), (*R*)-**11bA** was obtained after purification by flash chromatography (*n*-hexane/EtOAc 1/1) as a white solid (69 mg, 79%, 98% ee). **$[\alpha]_D^{23}$ =** –604.3 (*c* 1, $CHCl_3$). **HPLC** (Chiralpak IA, *n*-hexane/propan-2-ol 80:20, flow 1 mL/min) t_R 13.9 min (major) and 22.9 min (minor).

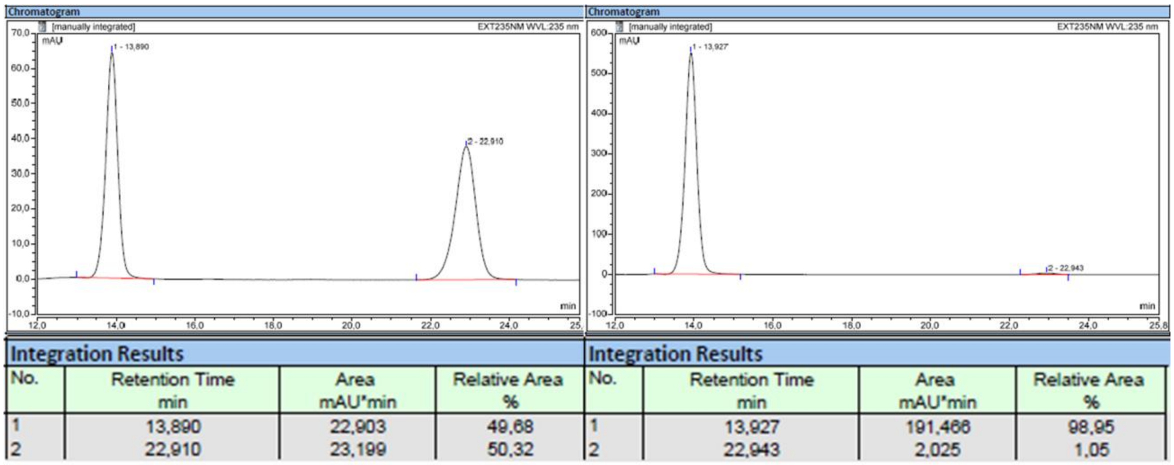

Integration Results

No.	Retention Time min	Area mAU*min	Relative Area %
1	13,890	22,903	49,68
2	22,910	23,199	50,32

Integration Results

No.	Retention Time min	Area mAU*min	Relative Area %
1	13,927	191,466	98,95
2	22,943	2,025	1,05

Diethyl (*S*)-[6,7-dichloro-2-(4-chlorobenzoyl)-1,2-dihydrophthalazin-1-yl]phosphonate [(*S*)-11cA]:

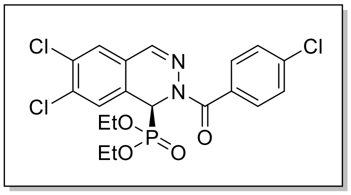

Following the general procedure **13**, starting from 6,7-dichlorophthalazine (**1c**) (39 mg, 0.2 mmol) and *tert*-butyldimethylsilyl diethyl phosphite (**2A**) (60 µL, 0.22 mmol), (*S*)-**11cA** was obtained after purification by flash chromatography (*n*-hexane/EtOAc 1/1) as a white solid (54 mg, 57%, 87% ee). **$[\alpha]_D^{27}$ =** +460.1 (*c* 1, $CHCl_3$). **^{1}H-NMR** (300 MHz, $CDCl_3$): δ 7.66 – 7.62 (m, 2H), 7.50 – 7.49 (m, 2H), 7.40 – 7.36 (m, 3H), 6.40 (d, *J* = 16.7 Hz, 1H), 4.21 – 3.89 (m, 4H), 1.27 (t, *J* = 7.3 Hz, 3H), 1.19 (t, *J* = 7.1 Hz, 3H). **^{13}C-NMR** (75.5 MHz, $CDCl_3$): δ 168.6, 141.5, 137.2, 136.3 (d, *J* = 3.2 Hz), 133.6 (d, *J* = 4.0 Hz), 132.2, 131.1, 129.7 (d, *J* = 5.3 Hz), 128.1, 127.6 (d, *J* = 3.0 Hz), 127.2 (d, *J* = 2.3 Hz), 124.1 (d, *J* = 3.8 Hz), 63.7 (d, *J* = 6.3 Hz), 63.4 (d, *J* = 7.5 Hz), 48.9 (d, *J* = 153.3 Hz), 16.5 – 16.4 (m). **^{31}P-NMR** (122 MHz, $CDCl_3$): δ 16.23. **HRMS** (ESI): *m/z* calcd for $C_{19}H_{18}O_4N_2Cl_3NaP$ $[M^++Na]$ 496.9962, found 496.9955. **HPLC** (Chiralpak IA, *n*-hexane/propan-2-ol 80:20, flow 1 mL/min) t_R 11.4 min (minor) and 17.0 min (major).

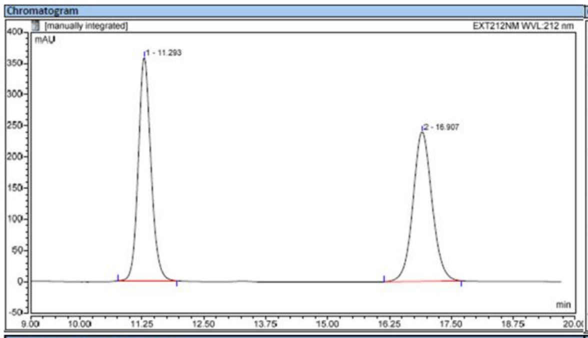

No.	Retention Time min	Area mAU*min	Relative Area %
1	11.293	105.891	49.71
2	16.907	107.126	50.29

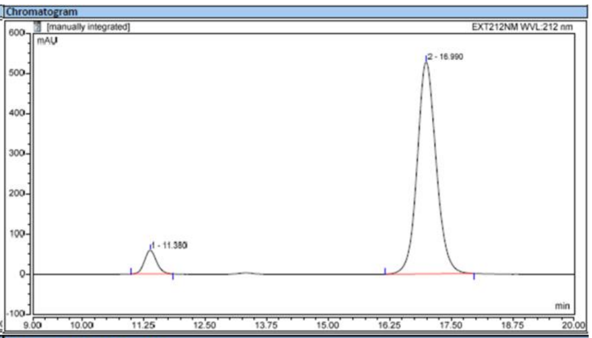

No.	Retention Time min	Area mAU*min	Relative Area %
1	11.380	16.807	6.65
2	16.990	236.037	93.35

Diethyl (*S*)-[2-(4-chlorobenzoyl)-5,8-dimethoxy-1,2-dihydrophthalazin-1-yl]phosphonate [(*S*)-11dA]:

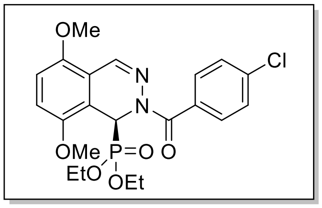

Following the general procedure **13**, starting from 5,8-dimethoxyphthalazine (**1d**) (38 mg, 0.2 mmol) and *tert*-butyldimethylsilyl diethyl phosphite (**2A**) (60 µL, 0.22 mmol), (*S*)-**11dA** was obtained after purification by flash chromatography (*n*-hexane/EtOAc 1/1) as a white solid (80 mg, 85%, 61% ee). **$[\alpha]_D^{28}$ =** +320.3 (*c* 2, $CHCl_3$). **^{1}H-NMR** (300 MHz, $CDCl_3$): δ 7.99 (s, 1H), 7.68 – 7.64 (m, 2H), 7.37 – 7.33 (m, 2H), 6.99 – 6.96 (m, 1H), 6.84 – 6.80 (m, 1H), 6.70 (d, *J* = 16.3 Hz, 1H), 4.15 – 3.97 (m, 4H), 3.86 (s, 3H), 3.82 (s, 3H), 1.25 – 1.19 (m, 6H). **^{13}C-NMR** (75.5 MHz, $CDCl_3$): δ 168.6, 149.9 (d, *J* = 3.3 Hz), 149.1 (d, *J* = 4.8 Hz), 140.2, 136.6, 132.8, 131.2, 127.9, 117.3 (d, *J* = 1.8 Hz), 115.3 (d, *J* = 2.7 Hz), 114.3 (d, *J* = 4.2 Hz), 111.3 (d, *J* = 3.3 Hz), 63.0 – 62.9 (m), 56.2, 56.0, 44.9 (d, *J* = 152.0 Hz), 16.5 – 16.3 (m). **^{31}P-NMR** (122 MHz, $CDCl_3$): δ 17.71. **HRMS** (ESI): *m/z* calcd for $C_{21}H_{24}O_6N_2ClNaP$ $[M^++Na]$ 489.0953, found 489.0944. **HPLC** (Chiralpak IB, *n*-hexane/propan-2-ol 90:10, flow 0.8 mL/min) t_R 31.0 min (major) and 36.2 min (minor).

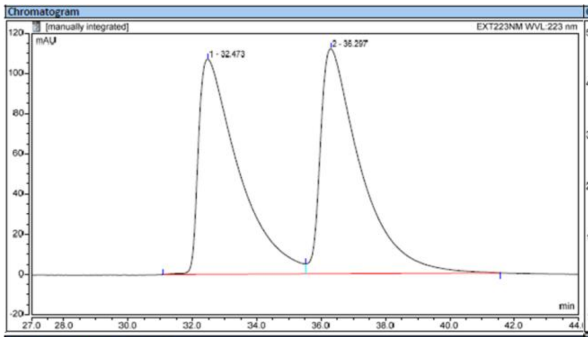

No.	Retention Time min	Area mAU*min	Relative Area %
1	32.473	154.416	48.81
2	36.297	161.928	51.19

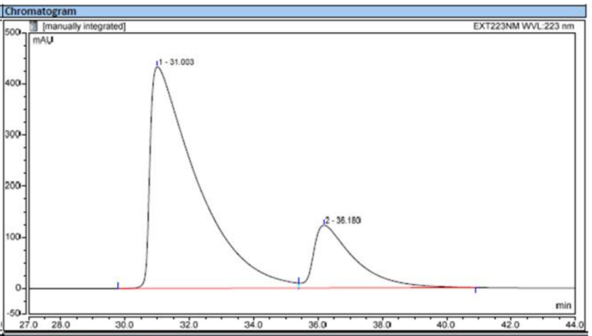

No.	Retention Time min	Area mAU*min	Relative Area %
1	31.003	730.790	80.29
2	36.180	179.348	19.71

Diethyl (*S*)-[(2-(4-chlorobenzoyl)-1,2-dihydrobenzo[*g*]phthalazin-1-yl]phosphonate [(*S*)-11eA]:

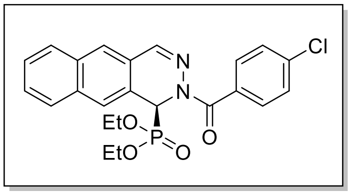

Following the general procedure **13**, starting from benzo[*g*]phthalazine (**1e**) (36 mg, 0.2 mmol) and *tert*-butyldimethylsilyl diethyl phosphite (**2A**) (60 µL, 0.22 mmol), (*S*)-**11eA** was obtained after purification by flash chromatography (*n*-hexane/EtOAc 1/1) as a white solid (68 mg, 75%, 93% ee). **[α]$_D^{26}$ =** +370.2 (*c* 1, $CHCl_3$). **^{1}H-NMR** (300 MHz, $CDCl_3$): δ 7.88 – 7.84 (m, 2H), 7.77 – 7.67 (m, 4H), 7.59 – 7.50 (m, 2H), 7.41 – 7.37 (m, 2H), 6.63 (d, *J* = 17.0 Hz, 1H), 4.22 – 4.07 (m, 2H), 4.03 – 3.75 (m, 2H), 1.26 (t, *J* = 7.0 Hz, 3H), 1.10 (t, *J* = 7.1 Hz, 3H). **^{13}C-NMR** (75.5 MHz, $CDCl_3$): δ 168.6, 143.9, 136.8, 134.9 (d, *J* = 2.9 Hz), 133.2 (d, *J* = 2.3 Hz), 132.8, 131.1, 128.9 (d, *J* = 1.3 Hz), 128.3, 128.1, 127.4 (d, *J* = 1.3 Hz), 127.1 (d, *J* = 6.6 Hz), 126.6 (d, *J* = 2.2 Hz), 124.0 (d, *J* = 3.2 Hz), 122.2 (d, *J* = 2.7 Hz), 63.6 (d, *J* = 6.2 Hz), 63.2 (d, *J* = 7.5 Hz), 50.1 (d, *J* = 151.7 Hz), 16.5 – 16.3 (m). **^{31}P-NMR** (122 MHz, $CDCl_3$): δ 17.65. **HRMS** (ESI): *m/z* calcd for $C_{23}H_{22}O_4N_2ClNaP$ [M^++Na] 479.0898, found 479.0890. **HPLC** (Chiralpak IA, *n*-hexane/propan-2-ol 80:20, flow 1 mL/min) t_R 24.8 min (minor) and 37.4 min (major).

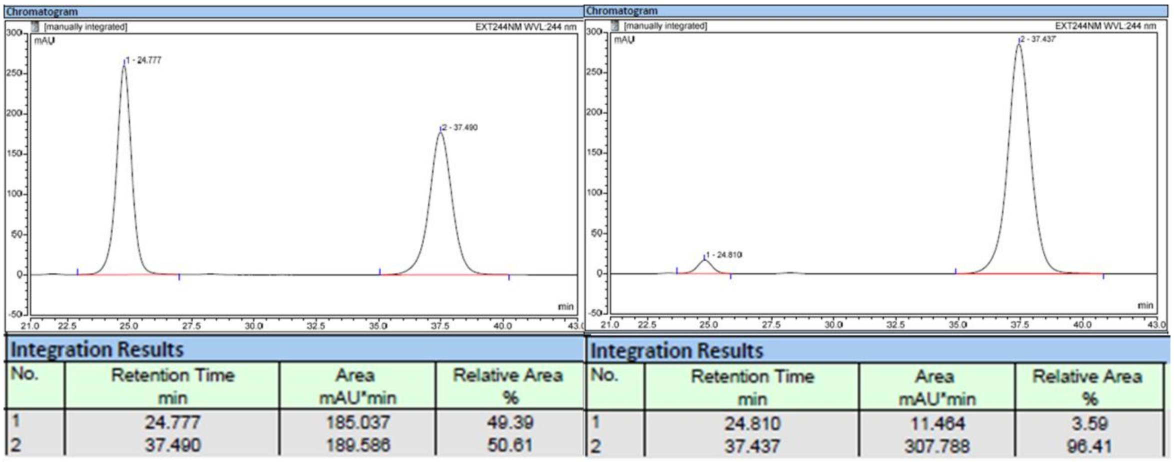

Integration Results

No.	Retention Time min	Area mAU*min	Relative Area %
1	24.777	185.037	49.39
2	37.490	189.586	50.61

Integration Results

No.	Retention Time min	Area mAU*min	Relative Area %
1	24.810	11.464	3.59
2	37.437	307.788	96.41

Diethyl (*S*)-[3-(4-chlorobenzoyl)-3,4-dihydrobenzo[*f*]phthalazin-4-yl]phosphonate/Diethyl (*S*)-(2-[4-chlorobenzoyl)-1,2-dihydrobenzo[*f*]phthalazin-1-yl]phosphonate [(*S*)-11fA/11fA’]:

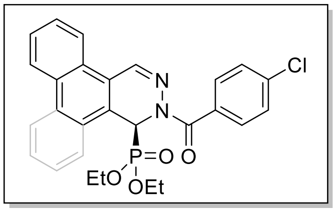

Following the general procedure **13**, starting benzo[*f*]phthalazine (**1f**) (36 mg, 0.2 mmol) and *tert*-butyldimethylsilyl diethyl phosphite (**2A**) (60 µL, 0.22 mmol), (*S*)-**11fA/11fA’** was obtained after purification by flash chromatography (*n*-hexane/EtOAc 1/1) as a white solid (76 mg, 83%, 92% ee major, 86% ee minor, *ratio regioisomer* 1.5/1). **[α]$_D^{28}$ =** +684.9 (*c* 2, $CHCl_3$). **^{1}H-NMR** (300 MHz, $CDCl_3$): The compound exists as a ~1.5:1 mixture of regioisomers. *Signals corresponding to both regioisomers:* δ 8.41 (s, 1H), 8.30 (d, *J* = 8.4 Hz, 1H), 8.20 (d, *J* = 8.4 Hz, 1H), 8.02 – 7.87 (m, 2H), 7.87 (s, 1H), 7.73 – 7.38 (m, 7H), 7.24 (d, *J* = 16.2 Hz, 1H), 6.61 (d, *J* = 16.6 Hz, 1H), 4.24 – 3.79 (m, 4H), 1.29 – 1.08 (m, 6H). **^{13}C-NMR** (75.5 MHz, $CDCl_3$): *Signals corresponding to both regioisomers:* δ 168.8, 168.6, 145.2, 141.0, 136.98 – 136.96 (m), 135.4 (d, *J* = 2.3 Hz), 133.7 (d, *J* = 2.5 Hz), 132.8 (d, *J* = 2.3 Hz), 132.6 (d, *J* = 3.9 Hz), 131.22, 131.18, 129.6 (d, *J* = 3.7 Hz), 129.3 (d, *J* = 4.0 Hz), 129.21 – 121.19 (m), 128.7, 128.4, 128.3, 128.13, 128.10, 127.7, 127.0 – 126.9 (m), 63.5 – 63.1 (m), 50.4 (d, *J* = 151.2 Hz), 47.1 (d, *J* = 152.9 Hz), 16.5 – 16.4 (m). **^{31}P-NMR** (122 MHz, $CDCl_3$): *Signal corresponding to the major regioisomer:* δ 17.09. *Signal corresponding to the minor regioisomer:* δ 17.51. **HRMS** (ESI): *m/z* calcd for $C_{23}H_{22}O_4N_2ClNaP$ [M^++Na] 479.0898, found 479.0890. **HPLC** (Chiralpak IA, *n*-hexane/propan-2-ol 90:10, flow 1 mL/min) *Major regioisomer:* t_R 42.2 min (minor) and 52.5 min (major). *Minor regioisomer:* t_R 24.6 min (minor) and 35.9 min (major).

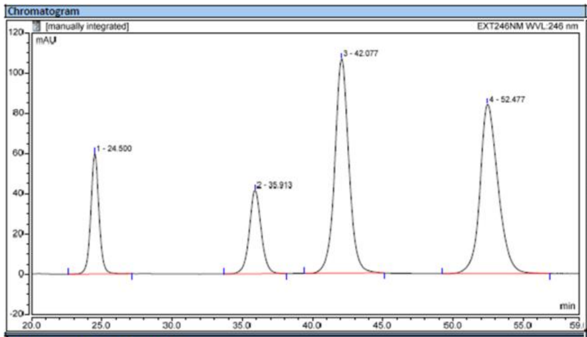

No.	Retention Time min	Area mAU*min	Relative Area %
1	24.500	40.205	12.16
2	35.913	40.642	12.29
3	42.077	123.793	37.44
4	52.477	126.047	38.12

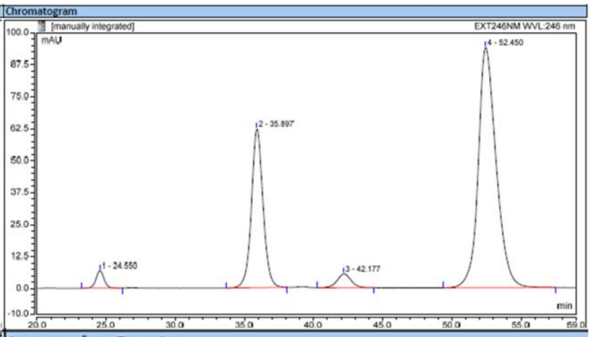

No.	Retention Time min	Area mAU*min	Relative Area %
1	24.550	4.633	2.18
2	35.897	60.588	28.47
3	42.177	6.419	3.02
4	52.450	141.147	66.33

Diethyl (*S*)-[6-chloro-2-(4-chlorobenzoyl)-1,2-dihydrophthalazin-1-yl]phosphonate [(*S*)-11gA]:

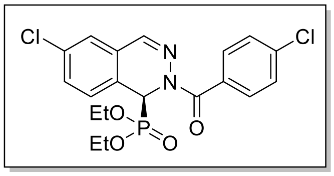

Following the general procedure **13**, starting from 6-chlorophthalazine (**1g**) (82 mg, 0.5 mmol) and *tert*-butyldimethylsilyl diethyl phosphite (**2A**) (150 µL, 0.55 mmol), (*S*)-**11gA** was obtained after purification by flash chromatography (*n*-hexane/EtOAc 1/1) as a colorless oil (120 mg, 54%, 91% ee). **$[\alpha]_D^{23}$ =** +516.3 (*c* 1, $CHCl_3$). **^{1}H-NMR** (300 MHz, $CDCl_3$) δ 7.68 – 7.63 (m, 2H), 7.52 (s, 1H), 7.49 – 7.46 (m, 1H), 7.41 – 7.33 (m, 3H), 7.29 – 7.28 (m, 1H), 6.44 (d, *J* = 16.4 Hz, 1H), 4.21 – 4.08 (m, 2H), 4.08 – 3.84 (m, 2H), 1.27 (t, *J* = 7.0 Hz, 3H), 1.16 (t, *J* = 7.1 Hz, 3H). **^{13}C-NMR** (75.5 MHz, $CDCl_3$): δ 168.7, 142.4, 137.1, 135.3 (d, *J* = 4.2 Hz), 132.4, 132.1 (d, *J* = 2.8 Hz), 131.1, 129.2 (d, *J* = 5.1 Hz), 128.2, 126.1 (d, *J* = 3.1 Hz), 126.0 – 125.9 (m), 63.6 (d, *J* = 6.3 Hz), 63.3 (d, *J* = 7.6 Hz), 49.4 (d, *J* = 152.9 Hz), 16.55 – 16.47 (m). **^{31}P-NMR** (122 MHz, $CDCl_3$): δ 16.77. **HRMS** (ESI): *m/z* calcd for $C_{19}H_{19}O_4N_2Cl_2NaP$ $[M^{+}+Na]$ 463.0352, found 463.0340. **HPLC** (Chiralpak IA, *n*-hexane/propan-2-ol 80:20, flow 1 mL/min) t_R 13.8 min (minor) and 19.4 min (major).

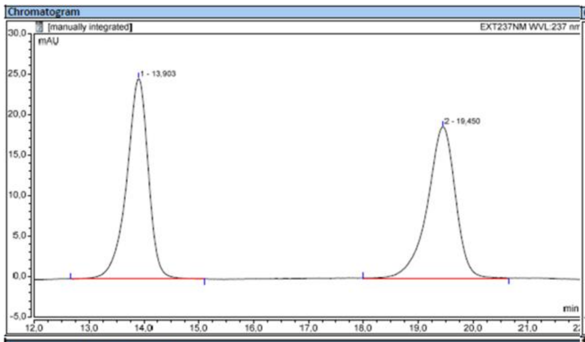

No.	Retention Time min	Area mAU*min	Relative Area %
1	13,903	11,317	50,60
2	19,450	11,050	49,40

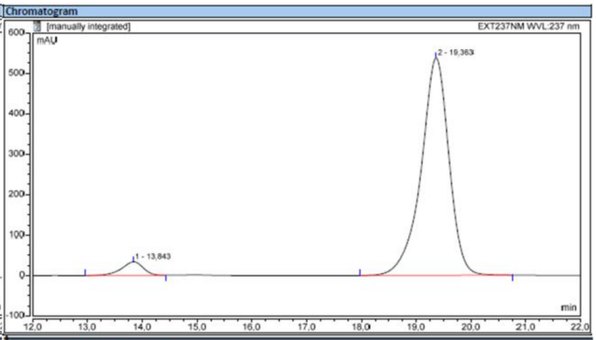

No.	Retention Time min	Area mAU*min	Relative Area %
1	13,843	15,299	4,61
2	19,363	316,402	95,39

Diethyl (*S*)-[7-chloro-2-(4-chlorobenzoyl)-1,2-dihydrophthalazin-1-yl]phosphonate [(*S*)-11gA']:

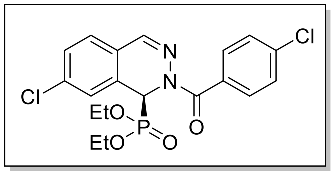

Following the general procedure **13**, starting from 6-chlorophthalazine (**1g**) (82 mg, 0.5 mmol) and *tert*-butyldimethylsilyl diethyl phosphite (**2A**) (150 µL, 0.55 mmol), (*S*)-**11gA'** was obtained after purification by flash chromatography (*n*-hexane/EtOAc 2/1) as a white solid (36 mg, 16%, 77% ee). $[\alpha]_D^{22}$ **=** +491.4 (*c* 1, $CHCl_3$). **^{1}H-NMR** (300 MHz, $CDCl_3$): δ 7.68 – 7.63 (m, 2H), 7.56 (s, 1H), 7.40 – 7.36 (m, 4H), 7.23 (d, *J* = 7.8 Hz, 1H), 6.43 (d, *J* = 16.9 Hz, 1H), 4.21 – 3.87 (m, 4H), 1.27 (t, *J* = 7.1 Hz, 3H), 1.17 (t, *J* = 7.1 Hz, 3H). **^{13}C-NMR** (75.5 MHz, $CDCl_3$): δ 168.6, 142.9, 138.2 (d, *J* = 3.2 Hz), 137.1, 132.5, 131.2, 129.6 (d, *J* = 3.4 Hz), 129.4 (d, *J* = 2.3 Hz), 128.1, 128.0 (d, *J* = 5.1 Hz), 127.3 (d, *J* = 3.0 Hz), 123.1 (d, *J* = 4.0 Hz), 63.7 (d, *J* = 6.3 Hz), 63.4 (d, *J* = 7.5 Hz), 49.5 (d, *J* = 152.6 Hz), 16.5 – 16.4 (m). **^{31}P-NMR** (122 MHz, $CDCl_3$): δ 16.60. **HRMS** (ESI): *m/z* calcd for $C_{19}H_{19}O_4N_2Cl_2NaP$ [M^++Na] 463.0352, found 463.0345. **HPLC** (Chiralpak IB, *n*-hexane/propan-2-ol 90:10, flow 1 mL/min) t_R 9.1 min (minor) and 10.6 min (major).

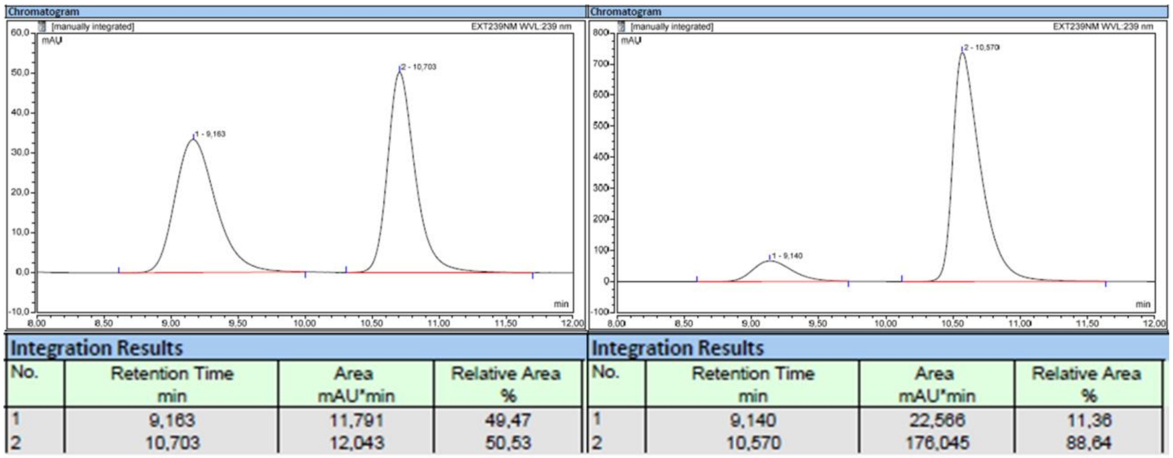

Integration Results

No.	Retention Time min	Area mAU*min	Relative Area %
1	9,163	11,791	49,47
2	10,703	12,043	50,53

Integration Results

No.	Retention Time min	Area mAU*min	Relative Area %
1	9,140	22,566	11,36
2	10,570	176,045	88,64

Diethyl (*S*)-[2-(4-chlorobenzoyl)-6-(dimethylamino)-1,2-dihydrophthalazin-1-yl]phosphonate [(*S*)-11hA]:

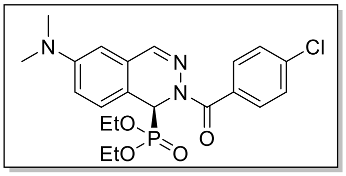

Following the general procedure **13**, starting from *N*,*N*-dimethylphthalazin-6-amine (**1h**) (35 mg, 0.2 mmol) and *tert*-butyldimethylsilyl diethyl phosphite (**2A**) (60 µL, 0.22 mmol), (*S*)-**11hA** was obtained after purification by flash chromatography (*n*-hexane/EtOAc 1/2) as a yellow solid (52 mg, 58%, 94% ee). $[\alpha]_D^{23}$ **=** +469.5 (*c* 1, $CHCl_3$). **^{1}H-NMR** (300 MHz, $CDCl_3$): δ 7.66 – 7.63 (m, 2H), 7.52 (s, 1H), 7.38 – 7.35 (m, 2H), 7.26 – 7.23 (m, 2H), 6.81 (dd, *J* = 8.5, 2.6 Hz, 1H), 6.56 (d, *J* = 2.6 Hz, 1H), 6.37 (d, *J* = 14.7 Hz, 1H), 4.17 – 4.07 (m, 2H), 4.01 – 3.78 (m, 2H), 2.98 (s, 6H), 1.26 (t, *J* = 7.0 Hz, 3H), 1.14 (t, *J* = 7.1 Hz, 3H). **^{13}C-NMR** (75.5 MHz, $CDCl_3$): δ 168.6, 151.0 (d, *J* = 2.5 Hz), 144.9, 136.6, 133.1, 131.0, 128.4 (d, *J* = 4.7 Hz), 128.0, 125.1 (d, *J* = 4.0 Hz), 115.6 (d, *J* = 2.1 Hz), 114.2 (d, *J* = 2.9 Hz), 109.6 (d, *J* = 2.9 Hz), 63.2 – 63.1 (m), 49.2 (d, *J* = 153.8 Hz), 40.5, 16.6 – 16.5 (m). **^{31}P-NMR** (122 MHz, $CDCl_3$): δ 18.04. **HRMS** (ESI): *m/z* calcd for $C_{21}H_{25}O_4N_3ClNaP$ [M^++Na] 472.1163, found 472.1152. **HPLC** (Chiralpak IB, *n*-hexane/propan-2-ol 90:10, flow 1 mL/min) t_R 19.5 min (minor) and 24.9 min (major).

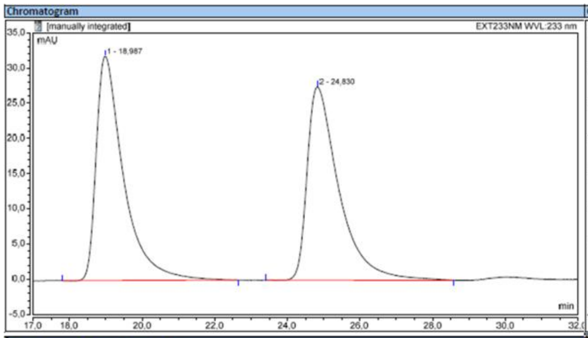

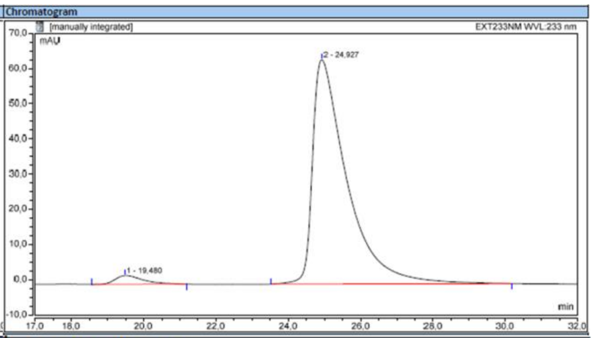

Integration Results

No.	Retention Time min	Area mAU*min	Relative Area %
1	18,987	27,721	50,00
2	24,830	27,718	50,00

Integration Results

No.	Retention Time min	Area mAU*min	Relative Area %
1	19,480	2,160	2,99
2	24,927	70,090	97,01

Diethyl (*S*)-[2-(4-chlorobenzoyl)-4-methoxy-1,2-dihydrophthalazin-1-yl]phosphonate [(*S*)-11iA]:

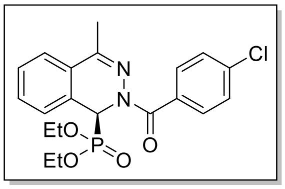

Following the general procedure **13**, starting from 1-methylphthalazine (**1i**) (29 mg, 0.2 mmol) and *tert*-butyldimethylsilyl diethyl phosphite (**2A**) (60 µL, 0.22 mmol), (*S*)-**11iA** was obtained after purification by flash chromatography (*n*-hexane/EtOAc 1/1) as a colorless oil (65 mg, 77%, 93% ee). **$[\alpha]_D^{25}$ =** +597.1 (*c* 1, $CHCl_3$). **^{1}H-NMR** (300 MHz, $CDCl_3$): δ 7.71 – 7.68 (m, 2H), 7.54 – 7.40 (m, 4H), 7.38 – 7.35 (m, 2H), 6.45 (d, *J* = 16.7 Hz, 1H), 4.19 – 4.10 (m, 2H), 4.02 – 3.77 (m, 2H), 2.33 (s, 3H), 1.26 (t, *J* = 7.0 Hz, 3H), 1.10 (t, *J* = 7.0 Hz, 3H). **^{13}C-NMR** (75.5 MHz, $CDCl_3$): δ 167.8, 150.9, 136.7, 132.8, 132.0 (d, *J* = 2.8 Hz), 131.5, 129.1 (d, *J* = 3.4 Hz), 128.7 (d, *J* = 1.9 Hz), 127.9, 127.7 (d, *J* = 5.3 Hz), 126.1 (d, *J* = 4.1 Hz), 124.6 (d, *J* = 3.1 Hz), 63.4 (d, *J* = 6.3 Hz), 63.0 (d, *J* = 7.5 Hz), 49.9 (d, *J* = 150.4 Hz), 19.1, 16.5 – 16.3 (m). **^{31}P-NMR** (122 MHz, $CDCl_3$): δ 17.61. **HRMS** (ESI): *m/z* calcd for $C_{20}H_{22}O_4N_2ClNaP$ [M^++Na] 443.0898, found 443.0887. **HPLC** (Chiralpak IA, *n*-hexane/propan-2-ol 90:10, flow 1 mL/min) t_R 23.9 min (minor) and 29.5 min (major).

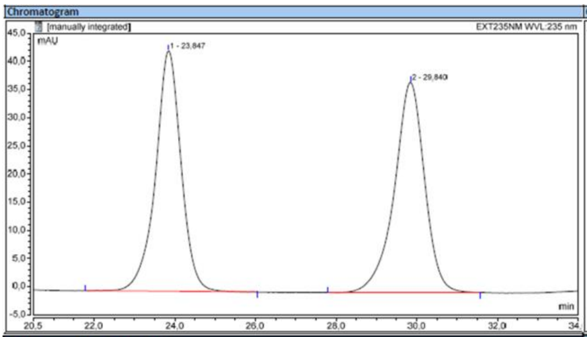

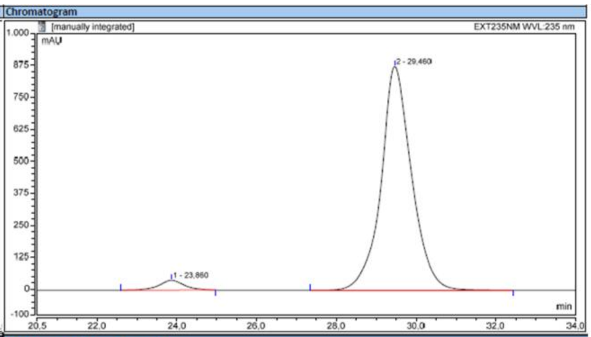

Integration Results

No.	Retention Time min	Area mAU*min	Relative Area %
1	23,847	31,330	49,22
2	29,840	32,319	50,78

Integration Results

No.	Retention Time min	Area mAU*min	Relative Area %
1	23,860	28,134	3,50
2	29,460	776,168	96,50

Diethyl (*S*)-[2-(4-chlorobenzoyl)-4-methoxy-1,2-dihydrophthalazin-1-yl]phosphonate [(*S*)-11jA]:

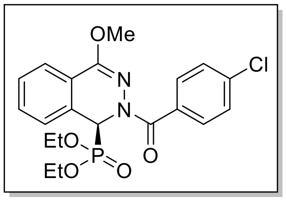

Following the general procedure **13**, starting from 1-methoxyphthalazine (**1j**) (32 mg, 0.2 mmol) and *tert*-butyldimethylsilyl diethyl phosphite (**2A**) (60 µL, 0.22 mmol), (*S*)-**11jA** was obtained after purification by flash chromatography (*n*-hexane/EtOAc 1/1) as a colorless oil (75 mg, 86%, 97% ee). **[α]$_D$^{28} =** +599.1 (*c* 1, $CHCl_3$). **^{1}H-NMR** (300 MHz, $CDCl_3$): δ 7.74 – 7.69 (m, 2H), 7.64 (d, *J* = 7.7 Hz, 1H), 7.54 – 7.32 (m, 5H), 6.49 (d, *J* = 17.4 Hz, 1H), 4.19 – 4.10 (m, 2H), 4.02 – 3.77 (m, 2H), 3.76 (s, 3H), 1.25 (t, *J* = 7.0 Hz, 3H), 1.09 (t, *J* = 7.1 Hz, 3H). **^{13}C-NMR** (75.5 MHz, $CDCl_3$): δ 167.0 (d, *J* = 1.5 Hz), 156.0 (d, *J* = 1.5 Hz), 136.3, 133.1, 132.4 (d, *J* = 2.8 Hz), 131.0, 130.6 (d, *J* = 1.6 Hz), 128.8 (d, *J* = 3.5 Hz), 127.7, 127.2 (d, *J* = 5.2 Hz), 124.0 (d, *J* = 3.1 Hz), 122.0 (d, *J* = 4.3 Hz), 63.4 (d, *J* = 6.1 Hz), 63.1 (d, *J* = 7.4 Hz), 54.4, 50.2 (d, *J* = 149.2 Hz), 16.4 (d, *J* = 6.3 Hz), 16.2 (d, *J* = 6.1 Hz). **^{31}P-NMR** (122 MHz, $CDCl_3$): δ 17.50. **HRMS** (ESI): *m/z* calcd for $C_{20}H_{22}O_5N_2ClNaP$ [M^++Na] 459.0847, found 459.0839. **HPLC** (Chiralpak IC, *n*-hexane/propan-2-ol 50:50, flow 1 mL/min) t_R 17.7 min (major) and 22.2 min (minor).

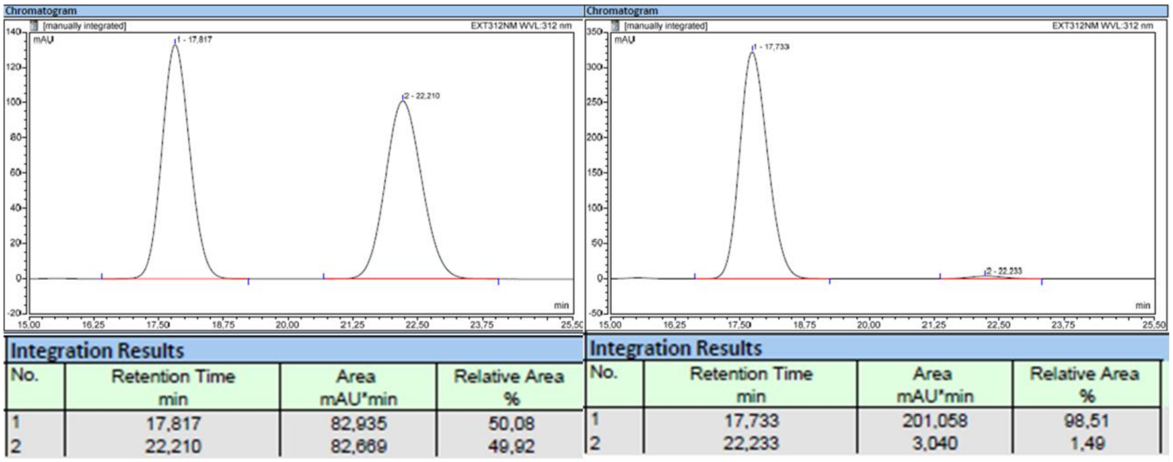

Integration Results

No.	Retention Time min	Area mAU*min	Relative Area %
1	17,817	82,935	50,08
2	22,210	82,669	49,92

Integration Results

No.	Retention Time min	Area mAU*min	Relative Area %
1	17,733	201,058	98,51
2	22,233	3,040	1,49

Diethyl (*R*)-[2-(4-chlorobenzoyl)-4-methoxy-1,2-dihydrophthalazin-1-yl]phosphonate [(*R*)-11jA]:

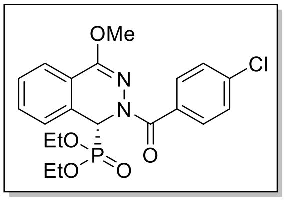

Following the general procedure **13**, starting from 1-methoxyphthalazine (**1j**) (32 mg, 0.2 mmol), *tert*-butyldimethylsilyl diethyl phosphite (**2A**) (60 µL, 0.22 mmol) and *ent*-**VII** (7 mg, 0.01 mmol, 5 mol%), (*R*)-**11jA** was obtained after purification by flash chromatography (*n*-hexane/EtOAc 1/1) as a colorless oil (73 mg, 84%, 97% ee). **[α]$_D$^{28} =** –595.3 (*c* 1, $CHCl_3$). **HPLC** (Chiralpak IC, *n*-hexane/propan-2-ol 50:50, flow 1 mL/min) t_R 17.9 min (minor) and 22.1 min (major).

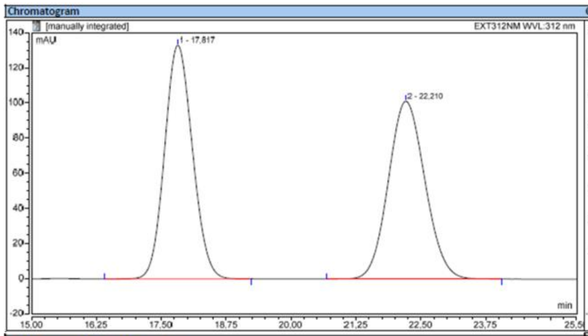

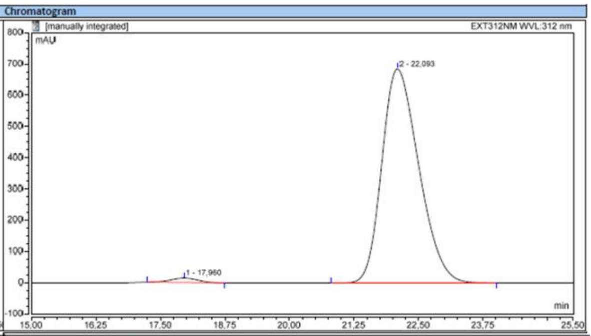

Integration Results

No.	Retention Time min	Area mAU*min	Relative Area %
1	17,817	82,935	50,08
2	22,210	82,669	49,92

Integration Results

No.	Retention Time min	Area mAU*min	Relative Area %
1	17,960	8,504	1,48
2	22,093	567,549	98,52

Diethyl (*S*)-[4-(benzyloxy)-2-(4-chlorobenzoyl)-1,2-dihydrophthalazin-1-yl]phosphonate [(*S*)-11kA]:

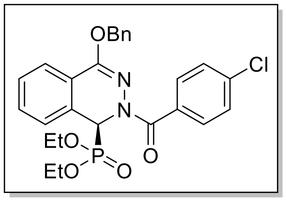

Following the general procedure **13**, starting from 1-(benzyloxy)phthalazine (**1k**) (47 mg, 0.2 mmol) and *tert*-butyldimethylsilyl diethyl phosphite (**2A**) (60 μL, 0.22 mmol), (*S*)-**11kA** was obtained after purification by flash chromatography (*n*-hexane/EtOAc 1/1) as a white solid (70 mg, 65%, 97% ee). **$[\alpha]_D^{25}$ =** +426.7 (*c* 1, $CHCl_3$). **^{1}H-NMR** (300 MHz, $CDCl_3$): δ 7.75 – 7.72 (m, 1H), 7.67 – 7.62 (m, 2H), 7.57 – 7.26 (m, 10H), 6.52 (d, *J* = 17.4 Hz, 1H), 5.19 – 5.09 (m, 2H), 4.20 – 4.11 (m, 2H), 4.01 – 3.75 (m, 2H), 1.27 (t, *J* = 7.1 Hz, 3H), 1.09 (t, *J* = 7.1 Hz, 3H). **^{13}C-NMR** (75.5 MHz, $CDCl_3$): δ 167.3 (d, *J* = 1.3 Hz), 155.2, 136.3, 136.2, 133.2, 132.5 (d, *J* = 2.8 Hz), 130.8, 130.7 (d, *J* = 1.6 Hz), 128.9 (d, *J* = 3.5 Hz), 128.6, 128.2, 127.82, 127.76, 127.3 (d, *J* = 5.2 Hz), 124.2 (d, *J* = 3.1 Hz), 122.1 (d, *J* = 4.3 Hz), 68.5, 63.5 (d, *J* = 6.1 Hz), 63.2 (d, *J* = 7.4 Hz), 50.2 (d, *J* = 149.2 Hz), 16.5 (d, *J* = 6.3 Hz), 16.3 (d, *J* = 6.0 Hz). **^{31}P-NMR** (122 MHz, $CDCl_3$): δ 17.49. **HRMS** (ESI): *m/z* calcd for $C_{26}H_{26}O_5N_2ClNaP$ $[M^+ +Na]$ 535.1160, found 535.1147. **HPLC** (Chiralpak IA, *n*-hexane/propan-2-ol 80:20, flow 1 mL/min) t_R 9.6 min (major) and 12.7 min (minor).

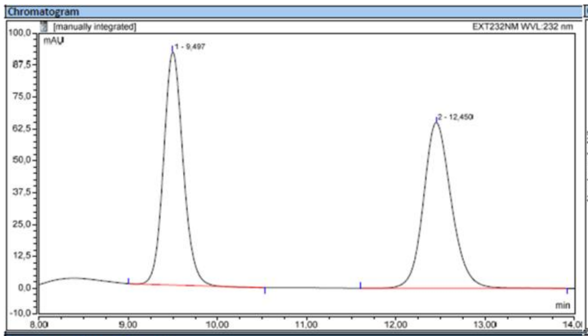

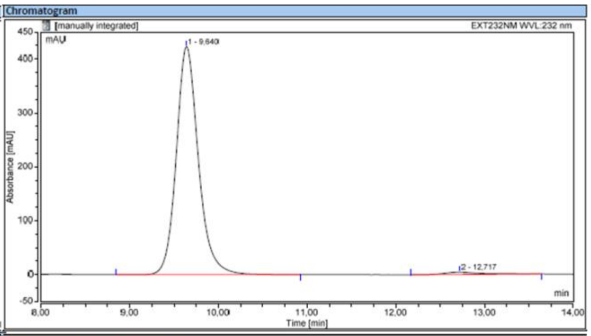

Integration Results

No.	Retention Time min	Area mAU*min	Relative Area %
1	9,497	23,174	49,62
2	12,450	23,526	50,38

Integration Results

No.	Retention Time min	Area mAU*min	Relative Area %
1	9,640	122,081	98,28
2	12,717	2,136	1,72

Diethyl (*S*)-[2-(4-chlorobenzoyl)-4-phenoxy-1,2-dihydrophthalazin-1-yl]phosphonate [(*S*)-11lA]:

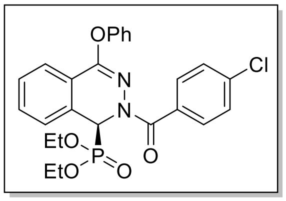

Following the general procedure **13**, starting from 1-phenoxyphthalazine (**1l**) (44 mg, 0.2 mmol) and *tert*-butyldimethylsilyl diethyl phosphite (**2A**) (60 µL, 0.22 mmol), (*S*)-**11lA** was obtained after purification by flash chromatography (*n*-hexane/EtOAc 1/1) as a colorless oil (76 mg, 76%, 94% ee). **$[\alpha]_D^{28}$ =** +432.6 (*c* 2, $CHCl_3$). **^{1}H-NMR** (300 MHz, $CDCl_3$): δ 7.85 (d, *J* = 7.1 Hz, 1H), 7.61 – 7.56 (m, 1H), 7.52 – 7.45 (m, 4H), 7.34 – 7.28 (m, 2H), 7.24 – 7.18 (m, 1H), 7.12 – 7.07 (m, 4H), 6.54 (d, *J* = 17.2 Hz, 1H), 4.21 – 4.09 (m, 2H), 4.07 – 3.81 (m, 2H), 1.24 (t, *J* = 7.1 Hz, 3H), 1.15 (t, *J* = 7.1 Hz, 3H). **^{13}C-NMR** (75.5 MHz, $CDCl_3$): δ 166.7 (d, *J* = 1.2 Hz), 155.5 (d, *J* = 1.4 Hz), 153.0, 136.2, 132.8 (d, *J* = 2.9 Hz), 132.1, 131.3, 130.5 (d, *J* = 1.9 Hz), 129.4, 129.1 (d, *J* = 3.5 Hz), 127.6 (d, *J* = 5.3 Hz), 127.5, 125.3, 124.1 (d, *J* = 3.1 Hz), 121.7, 121.6, 63.5 (d, *J* = 6.2 Hz), 63.3 (d, *J* = 7.4 Hz), 50.2 (d, *J* = 150.2 Hz), 16.5 – 16.3 (m). **^{31}P-NMR** (122 MHz, $CDCl_3$): δ 17.31. **HRMS** (ESI): *m/z* calcd for $C_{25}H_{24}O_5N_2ClNaP$ [M^++Na] 521.1004, found 521.0989. **HPLC** (Chiralpak IA, *n*-hexane/propan-2-ol 90:10, flow 1 mL/min) t_R 22.3 min (major) and 24.1 min (minor).

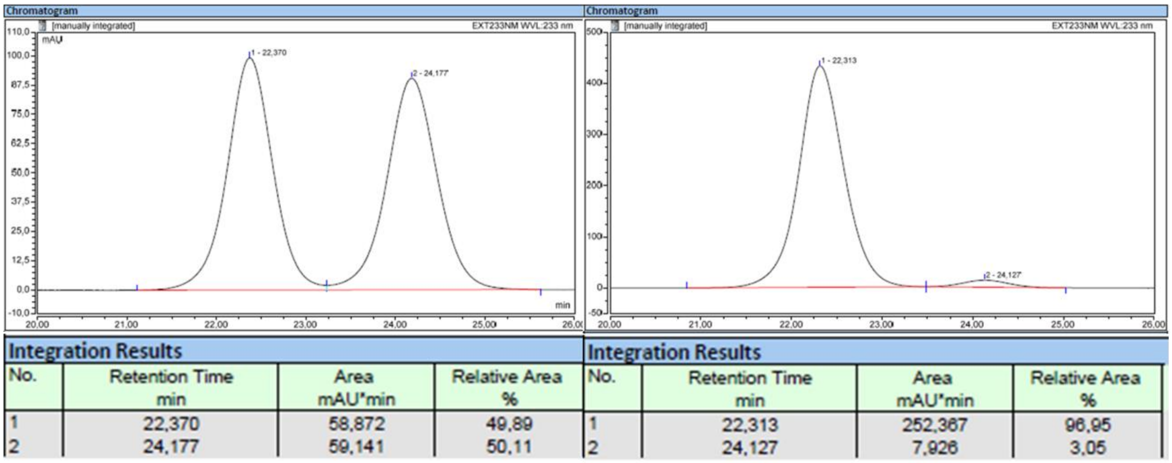

Integration Results

No.	Retention Time min	Area mAU*min	Relative Area %
1	22,370	58,872	49,89
2	24,177	59,141	50,11

Integration Results

No.	Retention Time min	Area mAU*min	Relative Area %
1	22,313	252,367	96,95
2	24,127	7,926	3,05

Diethyl (*S*)-[2-(4-chlorobenzoyl)-4-(2,2,2-trifluoroethoxy)-1,2-dihydrophthalazin-1-yl]phosphonate [(*S*)-11mA]:

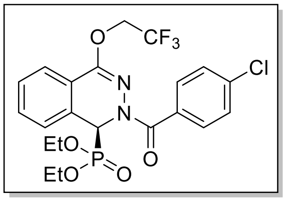

Following the general procedure **13**, starting from 1-(2,2,2-trifluoroethoxy)phthalazine (**1m**) (46 mg, 0.2 mmol) and *tert*-butyldimethylsilyl diethyl phosphite (**2A**) (60 µL, 0.22 mmol), (*S*)-**11mA** was obtained after purification by flash chromatography (*n*-hexane/EtOAc 1/1) as a colorless oil (79 mg, 78%, 86% ee). **$[\alpha]_D^{25}$ =** +405.5 (*c* 1, $CHCl_3$). **^{1}H-NMR** (300 MHz, $CDCl_3$): δ 7.70 – 7.55 (m, 4H), 7.49 – 7.42 (m, 2H), 7.39 – 7.34 (m, 2H), 6.48 (d, *J* = 17.1 Hz, 1H), 4.55 – 4.32 (m, 2H), 4.19 – 4.10 (m, 2H), 4.02 – 3.75 (m, 2H), 1.26 (t, *J* = 7.0 Hz, 3H), 1.08 (t, *J* = 7.1 Hz, 3H). **^{13}C-NMR** (75.5 MHz, $CDCl_3$): δ 167.3 (d, *J* = 1.0 Hz), 153.8 (d, *J* = 1.4 Hz), 136.7, 133.1 (d, *J* = 2.9 Hz), 132.7, 130.7, 130.6 (d, *J* = 1.8 Hz), 129.1 (d, *J* = 3.4 Hz), 127.9, 127.5 (d, *J* = 5.2 Hz), 124.0 (d, *J* = 3.0 Hz), 123.1 (q, *J* = 277.4 Hz), 121.0 (d, *J* = 4.3 Hz), 63.6 (d, *J* = 6.2 Hz), 63.2 (d, *J* = 7.3 Hz), 63.0 (q, *J* = 36.7 Hz), 50.4 (d, *J* = 149.6 Hz), 16.4 (d, *J* = 6.4 Hz), 16.2 (d, *J* = 6.1 Hz). **^{31}P-NMR** (122 MHz, $CDCl_3$): δ 17.09. **^{19}F NMR** (471 MHz, $CDCl_3$): δ −73.69 (s, 3F). **HRMS** (ESI): *m/z* calcd for $C_{21}H_{21}O_5N_2ClF_3NaP$ [M^++Na] 527.0721, found 527.0711. **HPLC** (Chiralpak ID, *n*-hexane/propan-2-ol 80:20, flow 1 mL/min) t_R 14.4 min (major) and 20.6 min (minor).

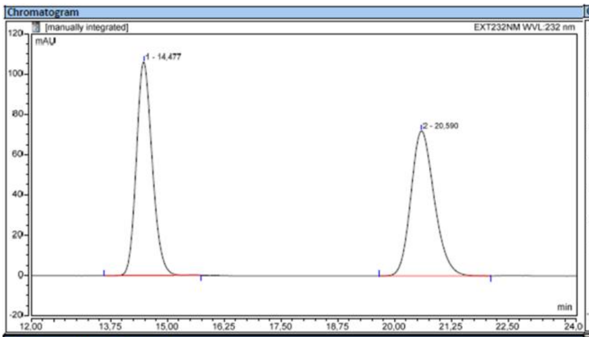

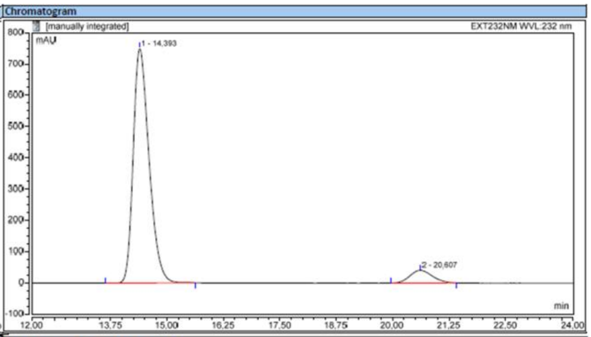

Integration Results

No.	Retention Time min	Area mAU*min	Relative Area %
1	14,477	42,903	49,93
2	20,590	43,018	50,07

Integration Results

No.	Retention Time min	Area mAU*min	Relative Area %
1	14,393	304,195	92,86
2	20,607	23,380	7,14

Diethyl (*S*)-[4-(allyloxy)-2-(4-chlorobenzoyl)-1,2-dihydrophthalazin-1-yl]phosphonate [(*S*)-11nA]:

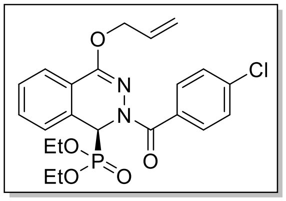

Following the general procedure **13**, starting from 1-(allyloxy)phthalazine (**1n**) (37 mg, 0.2 mmol) and *tert*-butyldimethylsilyl diethyl phosphite (**2A**) (60 µL, 0.22 mmol), (*S*)-**11nA** was obtained after purification by flash chromatography (*n*-hexane/EtOAc 1/1) as a yellow oil (65 mg, 70%, 97% ee). **$[\alpha]_D^{28}$ =** +510.8 (*c* 1, $CHCl_3$). **^{1}H-NMR** (300 MHz, $CDCl_3$): δ 7.71 – 7.67 (m, 3H), 7.56 – 7.39 (m, 3H), 7.37 – 7.33 (m, 2H), 6.50 (d, *J* = 17.4 Hz, 1H), 6.03 – 5.90 (m, 1H), 5.35 – 5.21 (m, 2H), 4.66 – 4.51 (m, 2H), 4.20 – 4.11 (m, 2H), 4.03 – 3.76 (m, 2H), 1.26 (t, *J* = 7.2 Hz, 3H), 1.10 (t, *J* = 7.2 Hz, 3H). **^{13}C-NMR** (75.5 MHz, $CDCl_3$): δ 167.1 (d, *J* = 1.4 Hz), 155.2 (d, *J* = 1.4 Hz), 136.3, 133.1, 132.5, 132.4, 131.0, 130.1 (d, *J* = 1.6 Hz), 128.9 (d, *J* = 3.5 Hz), 127.8, 127.3 (d, *J* = 5.3 Hz), 124.1 (d, *J* = 3.1 Hz), 122.1 (d, *J* = 4.3 Hz), 118.1, 67.5, 63.5 (d, *J* = 6.1 Hz), 63.2 (d, *J* = 7.4 Hz), 50.2 (d, *J* = 149.2 Hz), 16.5 (d, *J* = 6.4 Hz), 16.3 (d, *J* = 6.1 Hz). **^{31}P-NMR** (122 MHz, $CDCl_3$): δ 17.51. **HRMS** (ESI): *m/z* calcd for $C_{22}H_{24}O_5N_2ClNaP$ [M^++Na] 485.1004, found 485.0991. **HPLC** (Chiralpak IA, *n*-hexane/propan-2-ol 95:5, flow 1 mL/min) t_R 36.4 min (major) and 40.2 min (minor).

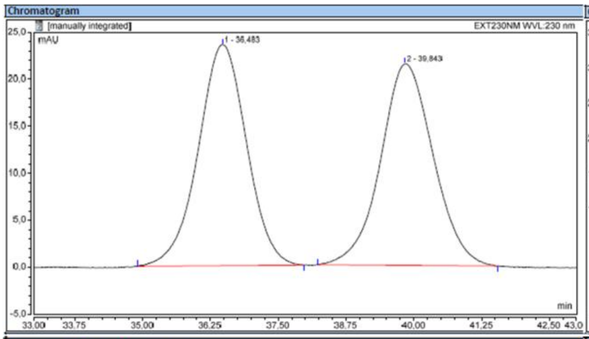

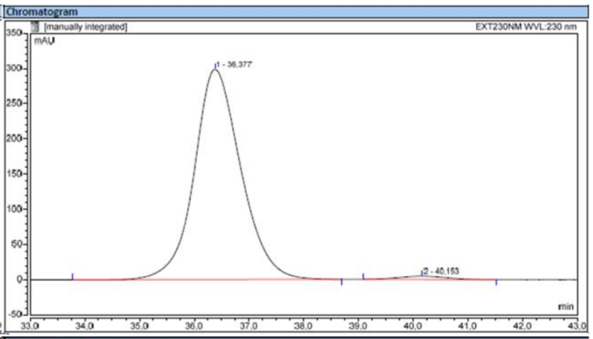

Integration Results

No.	Retention Time min	Area mAU*min	Relative Area %
1	36,483	24,290	50,16
2	39,843	24,133	49,84

Integration Results

No.	Retention Time min	Area mAU*min	Relative Area %
1	36,377	294,960	98,47
2	40,153	4,573	1,53

Diethyl (*S*)-[4-(benzylthio)-2-(4-chlorobenzoyl)-1,2-dihydrophthalazin-1-yl]phosphonate [(*S*)-11oA]:

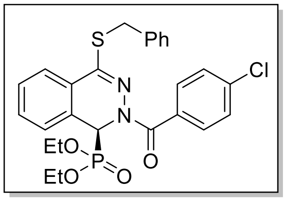

Following the general procedure **13**, starting from starting from 1-(benzylthio)phthalazine (**1o**) (50 mg, 0.2 mmol) and *tert*-butyldimethylsilyl diethyl phosphite (**2A**) (60 µL, 0.22 mmol), (*S*)-**11oA** was obtained after purification by flash chromatography (*n*-hexane/EtOAc 1/2) as a yellow oil (62 mg, 59%, 82% ee). **[α]$_D$^{27} =** +137.7 (*c* 1, $CHCl_3$). **^{1}H-NMR** (300 MHz, $CDCl_3$): δ 7.65 – 7.37 (m, 6H), 7.33 – 7.29 (m, 2H), 7.25 – 7.22 (m, 3H), 7.14 – 7.11 (m, 2H), 6.46 (d, *J* = 16.8 Hz, 1H), 4.18 – 4.01 (m, 4H), 3.94 – 3.72 (m, 2H), 1.26 (t, *J* = 7.1 Hz, 3H), 1.09 (t, *J* = 7.1 Hz, 3H). **^{13}C-NMR** (75.5 MHz, $CDCl_3$): δ 167.9, 152.6, 136.6 (d, *J* = 2.9 Hz), 133.2, 132.5 (d, *J* = 2.8 Hz), 130.8, 129.1 (d, *J* = 3.4 Hz), 128.9, 128.6, 128.5, 127.9, 127.8 (d, *J* = 5.3 Hz), 127.5, 125.3 (d, *J* = 4.1 Hz), 124.5 (d, *J* = 3.1 Hz), 63.5 (d, *J* = 6.1 Hz), 63.3 (d, *J* = 7.5 Hz), 50.2 (d, *J* = 149.5 Hz), 33.9, 16.5 (d, *J* = 6.3 Hz), 16.3 (d, *J* = 6.0 Hz). **^{31}P-NMR** (122 MHz, $CDCl_3$): δ 17.21. **HRMS** (ESI): *m/z* calcd for $C_{26}H_{26}O_4N_2ClNaPS$ [M^++Na] 551.0932 found 551.0931. **HPLC** (Chiralpak IA, *n*-hexane/propan-2-ol 80:20, flow 1 mL/min) t_R 9.9 min (major) and 24.5 min (minor).

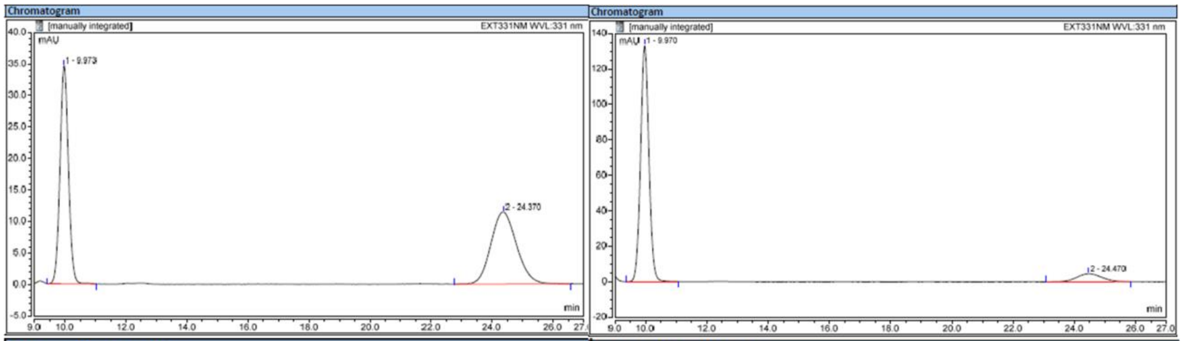

Integration Results

No.	Retention Time min	Area mAU*min	Relative Area %
1	9.973	11.101	49.94
2	24.370	11.127	50.06

Integration Results

No.	Retention Time min	Area mAU*min	Relative Area %
1	9.970	42.089	90.75
2	24.470	4.291	9.25

Diethyl (*S*)-[2-(4-chlorobenzoyl)-4-phenyl-1,2-dihydrophthalazin-1-yl]phosphonate [(*S*)-11pA]:

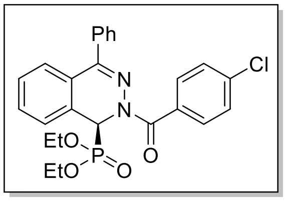

Following the general procedure **13**, starting from 1-phenylphthalazine (**1p**) (41 mg, 0.2 mmol) and *tert*-butyldimethylsilyl diethyl phosphite (**2A**) (60 µL, 0.22 mmol), (*S*)-**11pA** was obtained after purification by flash chromatography (*n*-hexane/EtOAc 1/1) as a white solid (70 mg, 73%, 94% ee). **[α]$_D$^{28} =** +671.9 (*c* 1, $CHCl_3$). **^{1}H-NMR** (300 MHz, $CDCl_3$): δ 7.78 – 7.73 (m, 2H), 7.58 – 7.50 (m, 4H), 7.45 – 7.34 (m, 7H), 6.51 (d, *J* = 16.7 Hz, 1H), 4.24 – 4.14 (m, 2H), 4.01 – 3.88 (m, 2H), 1.27 (t, *J* = 7.0 Hz, 3H), 1.09 (t, *J* = 7.1 Hz, 3H). **^{13}C-NMR** (75.5 MHz, $CDCl_3$): δ 168.2, 154.3, 136.9, 134.7, 132.6, 132.1 (d, *J* = 2.4 Hz), 131.5, 129.9 (d, *J* = 1.3 Hz), 129.7, 128.9, 128.8 (d, *J* = 3.2 Hz), 128.6, 128.0, 127.9, 127.1 (d, *J* = 3.0 Hz), 125.6 (d, *J* = 3.8 Hz), 63.4 (d, *J* = 6.2 Hz), 62.9 (d, *J* = 7.6 Hz), 50.2 (d, *J* = 150.3 Hz), 16.5 – 16.3 (m). **^{31}P-NMR** (122 MHz, $CDCl_3$): δ 17.54. **HRMS** (ESI): *m/z* calcd for $C_{25}H_{24}O_4N_2ClNaP$ [M^++Na] 505.1054, found 505.1047. **HPLC** (Chiralpak IC, *n*-hexane/propan-2-ol 50:50, flow 1 mL/min) t_R 15.6 min (major) and 18.0 min (minor).

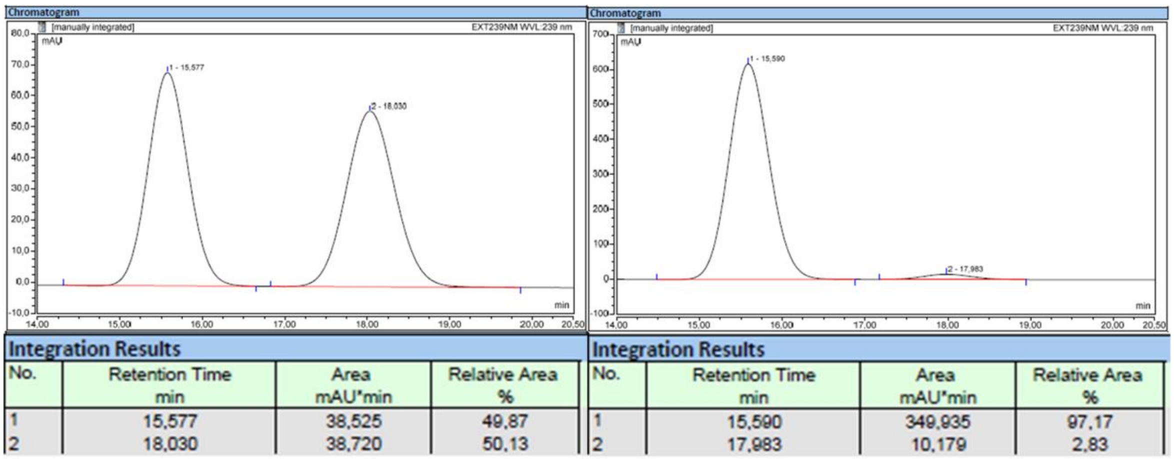

Integration Results

No.	Retention Time min	Area mAU*min	Relative Area %
1	15,577	38,525	49,87
2	18,030	38,720	50,13

Integration Results

No.	Retention Time min	Area mAU*min	Relative Area %
1	15,590	349,935	97,17
2	17,983	10,179	2,83

Diethyl (*R*)-[2-(4-chlorobenzoyl)-4-phenyl-1,2-dihydrophthalazin-1-yl]phosphonate [(*R*)-11pA]:

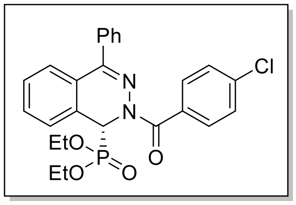

Following the general procedure **13**, starting from 1-phenylphthalazine (**1p**) (41 mg, 0.2 mmol), *tert*-butyldimethylsilyl diethyl phosphite (**2A**) (60 μL, 0.22 mmol), and *ent*-**VII** (7 mg, 0.01 mmol, 5 mol%), (*R*)-**11pA** was obtained after purification by flash chromatography (*n*-hexane/EtOAc 1/1) as a white solid (68 mg, 70%, 93% ee). **[α]$_D^{28}$ =** –677.1 (*c* 1, $CHCl_3$). **HPLC** (Chiralpak IC, *n*-hexane/propan-2-ol 50:50, flow 1 mL/min) t_R 15.6 min (minor) and 18.0 min (major).

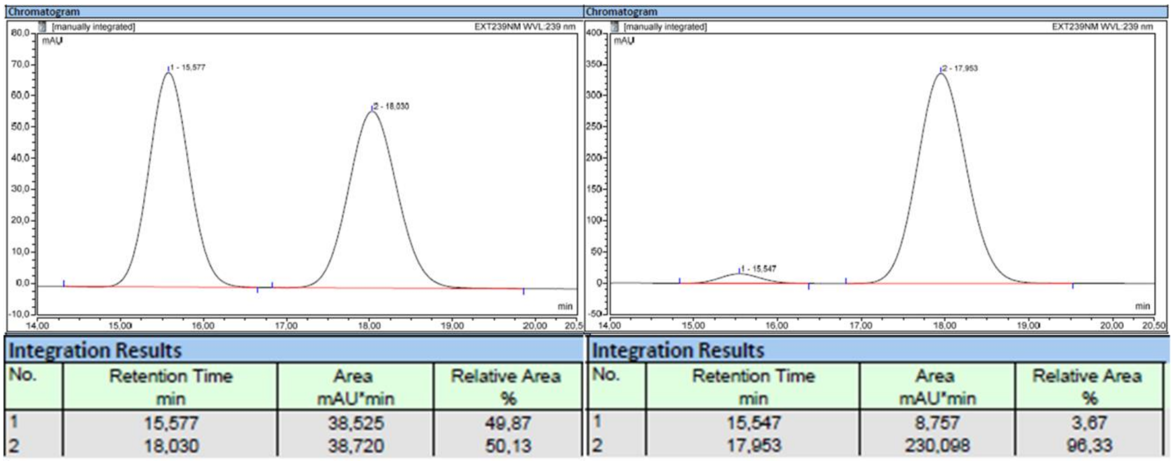

Integration Results

No.	Retention Time min	Area mAU*min	Relative Area %
1	15,577	38,525	49,87
2	18,030	38,720	50,13

Integration Results

No.	Retention Time min	Area mAU*min	Relative Area %
1	15,547	8,757	3,67
2	17,953	230,098	96,33

Diethyl (*S*)-[4-([1,1'-biphenyl]-2-yl)-2-(4-chlorobenzoyl)-1,2-dihydrophthalazin-1-yl]phosphonate [(*S*)-11qA]:

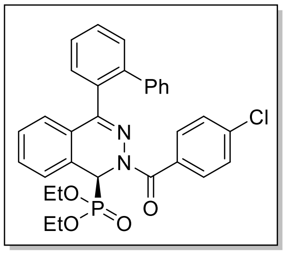

Following the general procedure **13**, starting from 1-([1,1'-biphenyl]-2-yl)phthalazine (**1q**) (57 mg, 0.2 mmol) and *tert*-butyldimethylsilyl diethyl phosphite (**2A**) (60 μL, 0.22 mmol), (*S*)-**11qA** was obtained after purification by flash chromatography (*n*-hexane/EtOAc 1/1) as a pale yellow solid (66 mg, 59%, 87% ee). **[α]$_D^{23}$ =** +432.8 (*c* 1, $CHCl_3$). **^{1}H-NMR** (300 MHz, $CDCl_3$): δ 7.56 – 7.35 (m, 7H), 7.28 – 7.18 (m, 6H), 7.11 – 6.96 (m, 4H), 6.51 (d, *J* = 16.9 Hz, 1H), 4.21 – 3.89 (m, 4H), 1.27 (t, *J* = 7.1 Hz, 3H), 1.18 (t, *J* = 7.1 Hz, 3H). **^{13}C-NMR** (75.5 MHz, $CDCl_3$): δ 168.3, 153.9, 141.6, 140.8, 136.4, 133.4, 132.05, 132.01, 131.2, 130.9 (d, *J* = 6.3 Hz), 129.5, 129.1 (d, *J* = 3.3 Hz), 129.0, 128.7, 128.6, 127.8, 127.6 (d, *J* = 5.4 Hz), 127.23, 127.16, 63.3 (d, *J* = 6.5 Hz), 63.1 (d, *J* = 7.5 Hz), 50.1 (d, *J* = 150.4 Hz), 16.6 – 16.5 (m). **^{31}P-NMR** (122 MHz, $CDCl_3$): δ 17.88. **HRMS** (ESI): *m/z* calcd for $C_{31}H_{28}O_4N_2ClNaP$ [M$^+$+Na]

581.1367, found 581.1355. **HPLC** (Chiralpak IA, *n*-hexane/propan-2-ol 90:10, flow 1 mL/min) t_R 15.8 min (major) and 20.3 min (minor).

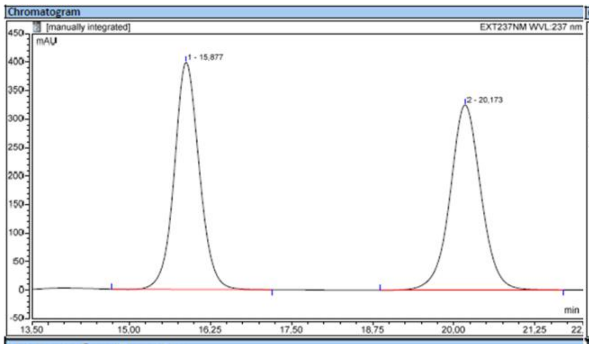

No.	Retention Time min	Area mAU*min	Relative Area %
1	15,877	177,745	49,82
2	20,173	179,025	50,18

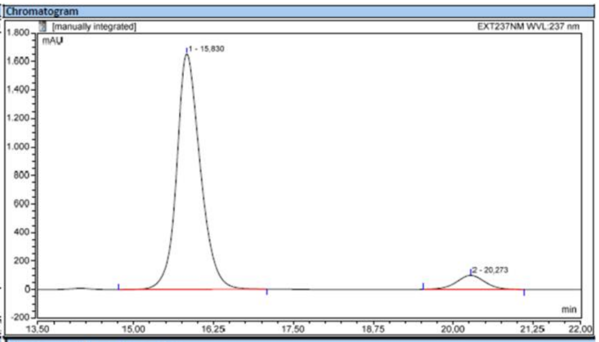

No.	Retention Time min	Area mAU*min	Relative Area %
1	15,830	709,065	93,34
2	20,273	50,591	6,66

Diethyl (*S*)-[4-chloro-2-(4-chlorobenzoyl)-1,2-dihydrophthalazin-1-yl]phosphonate [(*S*)-11rA]:

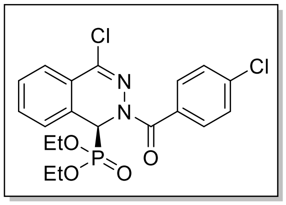

Following the general procedure **13**, starting from starting from 1-chlorophthalazine (**1r**) (33 mg, 0.2 mmol) and *tert*-butyldimethylsilyl diethyl phosphite (**2A**) (60 µL, 0.22 mmol), (*S*)-**11rA** was obtained after purification by flash chromatography (*n*-hexane/EtOAc 1/1) as a colorless oil (31 mg, 35%, 80% ee). **[α]$_D^{27}$ =** +314.2 (*c* 1, $CHCl_3$). **^{1}H-NMR** (300 MHz, $CDCl_3$): δ 7.73 – 7.68 (m, 3H), 7.62 – 7.57 (m, 1H), 7.52 – 7.45 (m, 2H), 7.42 – 7.38 (m, 2H), 6.47 (d, *J* = 16.3 Hz, 1H), 4.23 – 4.13 (m, 2H), 4.03 – 3.78 (m, 2H), 1.28 (t, *J* = 7.1 Hz, 3H), 1.12 (t, *J* = 7.1 Hz, 3H). **^{13}C-NMR** (126 MHz, $CDCl_3$): δ 167.6, 144.8, 137.4, 133.4 (d, *J* = 2.7 Hz), 131.7, 131.5, 129.5 (d, *J* = 3.2 Hz), 129.1 (d, *J* = 2.0 Hz), 128.2, 127.7 (d, *J* = 5.3 Hz), 125.8 (d, *J* = 2.8 Hz), 124.7 (d, *J* = 4.1 Hz), 63.8 (d, *J* = 6.2 Hz), 63.3 (d, *J* = 7.7 Hz), 50.6 (d, *J* = 150.9 Hz), 16.5 (d, *J* = 6.4 Hz), 16.3 (d, *J* = 6.1 Hz). **^{31}P-NMR** (122 MHz, $CDCl_3$): δ 16.44. **HRMS** (ESI): *m/z* calcd for $C_{19}H_{19}O_4N_2Cl_2NaP$ [M^++Na] 463.0352, found 463.0347. **HPLC** (Chiralpak IA, *n*-hexane/propan-2-ol 85:15, flow 1 mL/min) t_R 10.4 min (major) and 11.4 min (minor).

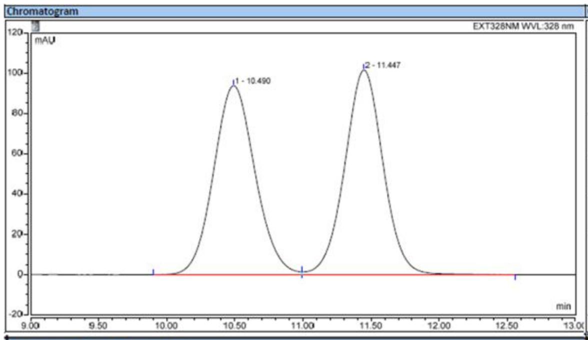

No.	Retention Time min	Area mAU*min	Relative Area %
1	10.490	33.056	49.91
2	11.447	33.169	50.09

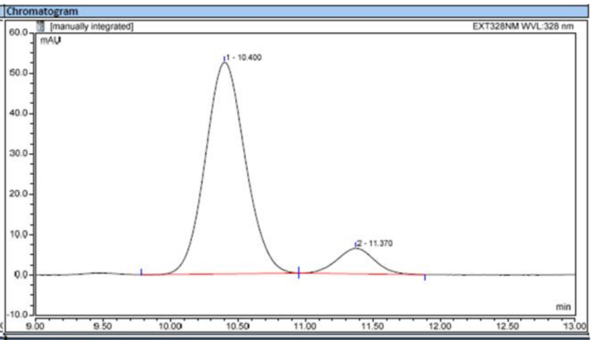

No.	Retention Time min	Area mAU*min	Relative Area %
1	10.400	18.000	89.85
2	11.370	2.034	10.15

Diethyl (*S*)-[2-(4-chlorobenzoyl)-4-(pyridin-3-yl)-1,2-dihydrophthalazin-1-yl]phosphonate [(*S*)-11sA]:

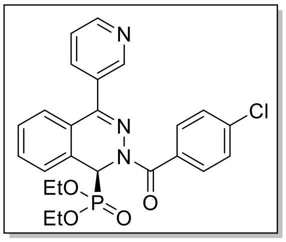

Following the general procedure **13**, starting from starting from 1-(pyridin-3-yl)phthalazine (**1s**) (41 mg, 0.2 mmol) and *tert*-butyldimethylsilyl diethyl phosphite (**2A**) (60 µL, 0.22 mmol), (*S*)-**11sA** was obtained after purification by flash chromatography (EtOAc) as a colorless oil (55 mg, 57%, 97% ee). **$[\alpha]_D^{27}$ =** +607.4 (*c* 1, $CHCl_3$). **^{1}H-NMR** (300 MHz, $CDCl_3$): δ 8.80 (s, 1H), 8.69 – 8.67 (m, 1H), 7.86 – 7.82 (m, 1H), 7.74 – 7.69 (m, 2H), 7.61 – 7.51 (m, 2H), 7.46 – 7.30 (m, 5H), 6.50 (d, *J* = 16.6 Hz, 1H), 4.23 – 4.11 (m, 2H), 4.02 – 3.87 (m, 2H), 1.27 (t, *J* = 7.1 Hz, 3H), 1.10 (t, *J* = 7.1 Hz, 3H). **^{13}C-NMR** (75.5 MHz, $CDCl_3$): δ 168.4, 151.5, 150.6, 149.9, 137.2, 136.2, 132.5 (d, *J* = 2.4 Hz), 132.4, 131.4, 130.7, 129.8 (d, *J* = 1.2 Hz), 129.1 (d, *J* = 3.2 Hz), 128.2 (d, *J* = 5.5 Hz), 128.1, 126.4 (d, *J* = 2.9 Hz), 125.1 (d, *J* = 3.8 Hz), 123.5, 63.6 (d, *J* = 6.3 Hz), 63.0 (d, *J* = 7.6 Hz), 50.3 (d, *J* = 150.7 Hz), 16.6 – 16.4 (m). **^{31}P-NMR** (122 MHz, $CDCl_3$): δ 17.32. **HRMS** (ESI): *m/z* calcd for $C_{24}H_{24}O_4N_3ClP$ [M^++H] 484.1187, found 484.1182. **HPLC** (Chiralpak IA, *n*-hexane/propan-2-ol 90:10, flow 1 mL/min) t_R 22.9 min (major) and 27.8 min (minor).

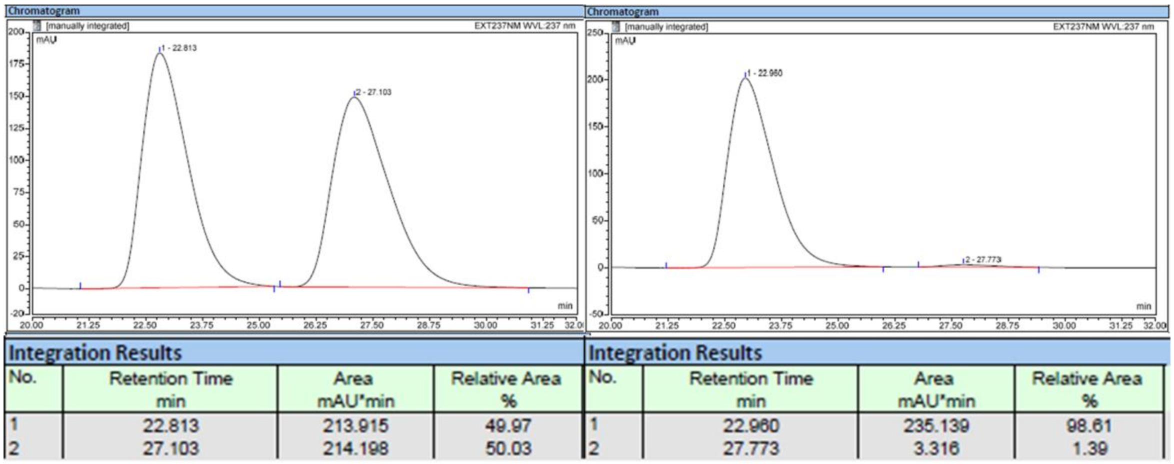

Integration Results

No.	Retention Time min	Area mAU*min	Relative Area %
1	22.813	213.915	49.97
2	27.103	214.198	50.03

Integration Results

No.	Retention Time min	Area mAU*min	Relative Area %
1	22.960	235.139	98.61
2	27.773	3.316	1.39

Diethyl (*S*)-[2-(4-chlorobenzoyl)-4-(dimethylamino)-1,2-dihydrophthalazin-1-yl]phosphonate [(*S*)-11tA]:

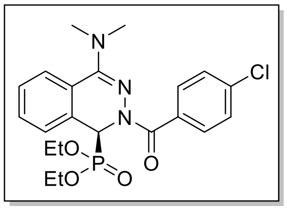

Following the general procedure **13**, starting from *N*,*N*-dimethylphthalazin-1-amine (**1t**) (35 mg, 0.2 mmol) and *tert*-butyldimethylsilyl diethyl phosphite (**2A**) (60 µL, 0.22 mmol), (*S*)-**11tA** was obtained after purification by flash chromatography (*n*-hexane/EtOAc 1/2) as a yellow oil (61 mg, 68%, 84% ee). **$[\alpha]_D^{23}$ =** +503.7 (*c* 1, $CHCl_3$). **^{1}H-NMR** (300 MHz, $CDCl_3$): δ 7.74 – 7.70 (m, 2H), 7.54 – 7.39 (m, 4H), 7.34 – 7.30 (m, 2H), 6.39 (d, *J* = 17.7 Hz, 1H), 4.20 – 3.96 (m, 4H), 2.79 (s, 6H), 1.29 (t, *J* = 7.1 Hz, 3H), 1.19 (t, *J* = 7.1 Hz, 3H). **^{13}C-NMR** (75.5 MHz, $CDCl_3$): δ 166.8 (d, *J* = 1.4 Hz), 158.0, 136.3, 133.1, 131.8, 131.3, 128.5 (d, *J* = 3.2 Hz), 127.6, 127.5, 126.3 (d, *J* = 3.0 Hz), 123.4 (d, *J* = 3.9 Hz), 63.0 – 62.9 (m), 50.3 (d, *J* = 149.2 Hz), 40.9, 16.7 – 16.5 (m). **^{31}P-NMR** (122 MHz, $CDCl_3$): δ 18.79. **HRMS** (ESI): *m/z* calcd for $C_{21}H_{26}O_4N_3ClP$ [M^++H] 450.1344, found 450.1334. **HPLC** (Chiralpak IA, *n*-hexane/propan-2-ol 80:20, flow 1 mL/min) t_R 9.7 min (minor) and 19.0 min (major).

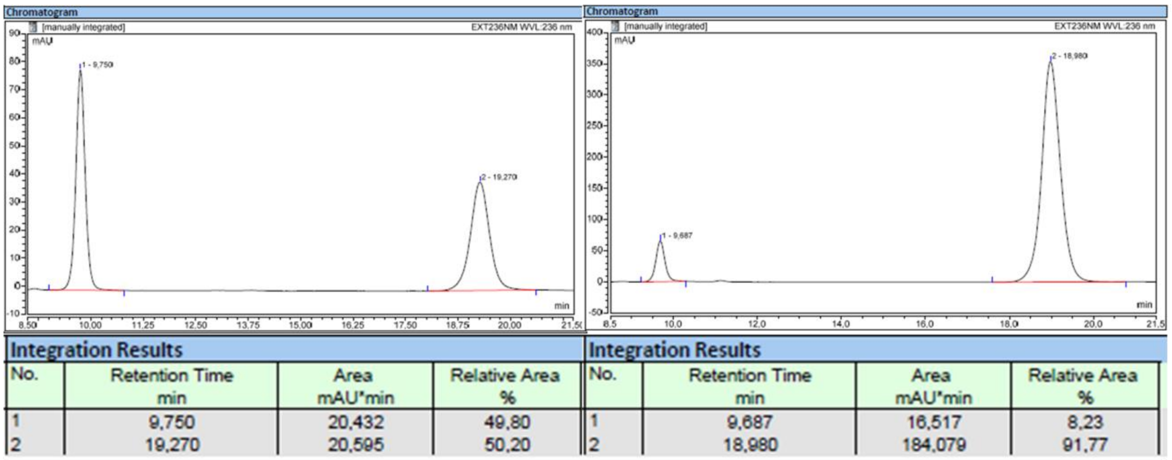

Integration Results

No.	Retention Time min	Area mAU*min	Relative Area %
1	9.750	20.432	49.80
2	19.270	20.595	50.20

Integration Results

No.	Retention Time min	Area mAU*min	Relative Area %
1	9.687	16.517	8.23
2	18.980	184.079	91.77

Diethyl (*S*)-[2-(4-chlorobenzoyl)-4-morpholino-1,2-dihydrophthalazin-1-yl]phosphonate [(*S*)-11uA]:

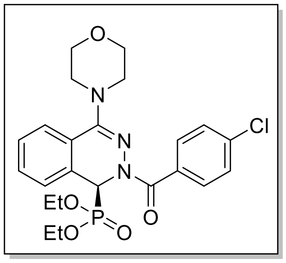

Following the general procedure **13**, starting from 4-(phthalazin-1-yl)morpholine (**1u**) (43 mg, 0.2 mmol) and *tert*-butyldimethylsilyl diethyl phosphite (**2A**) (60 µL, 0.22 mmol), (*S*)-**11uA** was obtained after purification by flash chromatography (*n*-hexane/EtOAc 1/2) as a colorless oil (50 mg, 51%, 84% ee). **$[\alpha]_D^{27}$ =** +498.3 (*c* 1, $CHCl_3$). **^{1}H-NMR** (300 MHz, $CDCl_3$): δ 7.70 – 7.65 (m, 2H), 7.54 – 7.40 (m, 4H), 7.36 – 7.31 (m, 2H), 6.39 (d, *J* = 17.7 Hz, 1H), 4.20 – 3.97 (m, 4H), 3.90 – 3.73 (m, 4H), 3.35 – 3.28 (m, 2H), 3.02 – 2.95 (m, 2H), 1.32 – 1.20 (m, 9H). **^{13}C-NMR** (75.5 MHz, $CDCl_3$): δ 167.1, 157.1, 136.5, 133.0, 131.9, 131.7 (d, *J* = 2.3 Hz), 131.2, 128.7 (d, *J* = 3.2 Hz), 127.8, 127.7, 125.8 (d, *J* = 2.8 Hz), 122.9 (d, *J* = 3.9 Hz), 66.5, 63.2 (d, *J* = 7.2 Hz), 62.9 (d, *J* = 6.7 Hz), 50.2 (d, *J* = 149.7 Hz), 49.7, 16.7 (d, *J* = 5.3 Hz), 16.6 (d, *J* = 6.2 Hz). **^{31}P-NMR** (122 MHz, $CDCl_3$): δ 18.57. **HRMS** (ESI): *m/z* calcd for $C_{23}H_{27}O_5N_3ClNaP$ $[M^++Na]$ 514.1269, found 514.1266. **HPLC** (Chiralpak IA, *n*-hexane/propan-2-ol 80:20, flow 1 mL/min) t_R 8.8 min (minor) and 10.8 min (major).

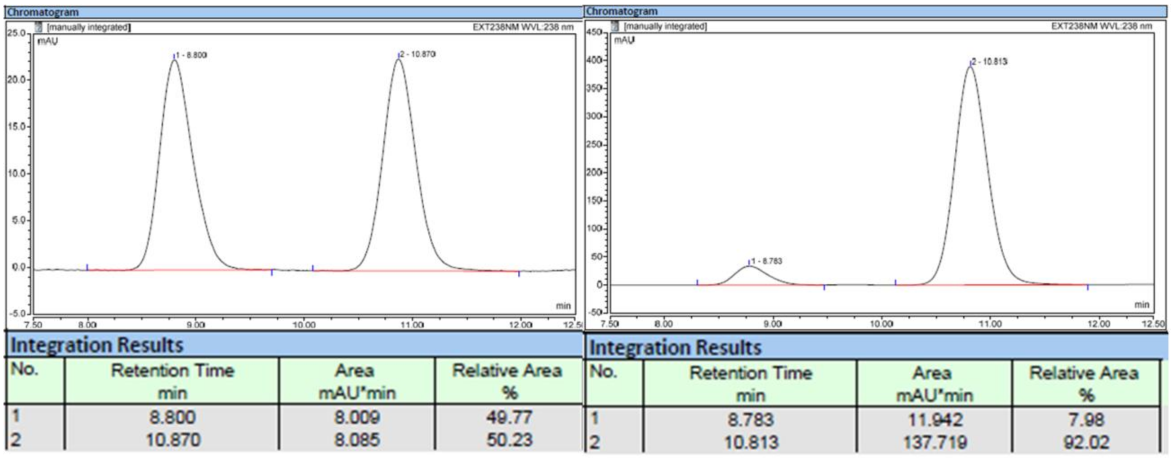

Integration Results

No.	Retention Time min	Area mAU*min	Relative Area %
1	8.800	8.009	49.77
2	10.870	8.085	50.23

Integration Results

No.	Retention Time min	Area mAU*min	Relative Area %
1	8.783	11.942	7.98
2	10.813	137.719	92.02

Diethyl (*S*)-[2-(4-chlorobenzoyl)-2,3-dihydropyridazin-3-yl]phosphonate [(*S*)-11vA]: Following the general procedure **13**, starting from pyridazine (**1v**) (15 µL, 0.2 mmol) and *tert*-butyldimethylsilyl diethyl phosphite (**2A**) (60 µL, 0.22 mmol), (*S*)-**11vA** was obtained after purification by flash chromatography (*n*-hexane/EtOAc 1/2) as a yellow oil (65 mg, 91%, 26% ee, *the product contain traces of the C4 addition adduct*). **[α]$_D$^{26} =** +157.7 (*c* 1, $CHCl_3$). **^{1}H-NMR** (300 MHz, $CDCl_3$): δ 7.65 – 7.61 (m, 2H), 7.38 – 7.34 (m, 2H), 7.08 – 7.06 (m, 1H), 6.37 – 6.30 (m, 1H), 6.06 – 5.92 (m, 2H), 4.25 – 4.09 (m, 4H), 1.35 – 1.24 (m, 6H). **^{13}C-NMR** (75.5 MHz, $CDCl_3$): δ 168.9, 141.4 (d, *J* = 4.4 Hz), 136.9, 132.4, 131.0, 128.0, 127.2 (d, *J* = 5.6 Hz), 118.9 (d, *J* = 10.0 Hz), 63.4 – 63.2 (m), 47.5 (d, *J* = 154.0 Hz), 16.6 – 16.4 (m). **^{31}P-NMR** (122 MHz, $CDCl_3$): δ 16.22. **HRMS** (ESI): *m/z* calcd for $C_{15}H_{18}O_4N_2ClNaP$ [M$^+$+Na] 379.0585, found 379.0578. **HPLC** (Chiralpak IB, *n*-hexane/propan-2-ol 90:10, flow 1 mL/min) t_R 11.9 min (minor) and 13.8 min (major).

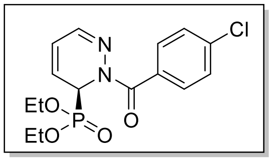

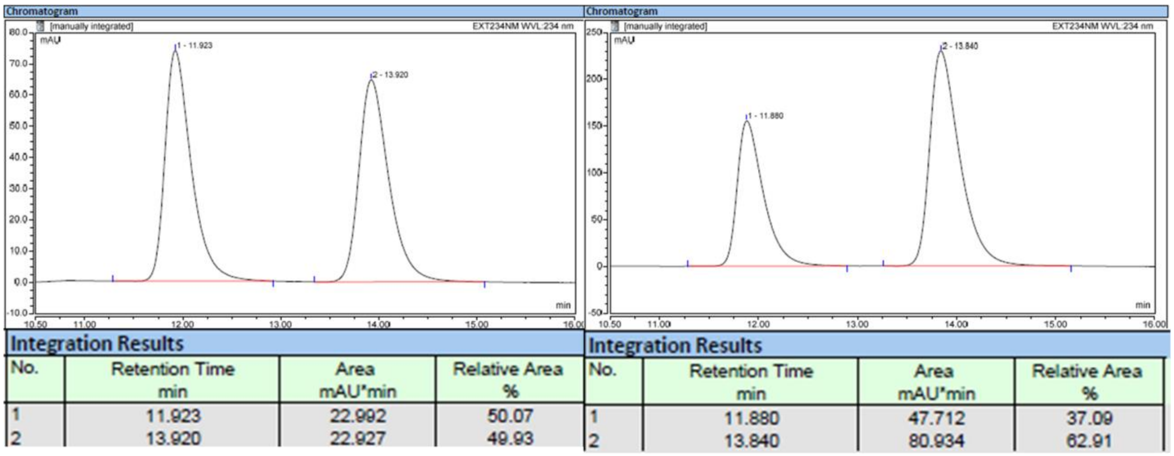

Integration Results

No.	Retention Time min	Area mAU*min	Relative Area %
1	11.923	22.992	50.07
2	13.920	22.927	49.93

Integration Results

No.	Retention Time min	Area mAU*min	Relative Area %
1	11.880	47.712	37.09
2	13.840	80.934	62.91

Diethyl (*S*)-(3-(4-chlorobenzoyl)-3,4-dihydroquinazolin-4-yl)phosphonate [(*S*)-11wA]: Following the general procedure **13**, starting from quinazoline (**1w**) (26 mg, 0.2 mmol) and *tert*-butyldimethylsilyl diethyl phosphite (**2A**) (60 µL, 0.22 mmol), (*S*)-**11wA** was obtained after purification by flash chromatography (*n*-hexane/EtOAc 1/2) as a colorless oil (40 mg, 49%, 90% ee). **[α]$_D$^{22} =** +33.2 (*c* 0.5, Acetone). **^{1}H-NMR** (300 MHz, Acetone-*d$_6$*): δ 7.73 (s, 1H), 7.69 – 7.61 (m, 4H), 7.40 – 7.25 (m, 4H), 6.03 (d, *J* = 14.3 Hz, 1H), 4.12 – 3.75 (m, 4H), 1.21 – 1.10 (m, 6H). **^{13}C-NMR** (75.5 MHz, Acetone-*d$_6$*): δ 168.7, 143.3, 141.5 (d, *J* = 3.9 Hz), 138.0, 133.1, 131.2, 130.1 (d, *J* = 3.3 Hz), 130.0, 128.7 (d, *J* = 4.6 Hz), 128.1 (d, *J* = 2.7 Hz), 126.7 (d, *J* = 2.9 Hz), 121.9 (d, *J* = 3.5 Hz), 63.7 – 63.6 (m), 51.3 (d, *J* = 152.5 Hz), 16.7 – 16.6 (m). **^{31}P-NMR** (122 MHz, Acetone-*d$_6$*): δ 17.24. **HRMS** (ESI): *m/z* calcd for $C_{19}H_{20}O_4N_2ClNaP$ [M$^+$+Na] 429.0741, found 429.0737. **HPLC** (Chiralpak IC, *n*-hexane/propan-2-ol 70:30, flow 1 mL/min) t_R 20.1 min (major) and 22.9 min (minor).

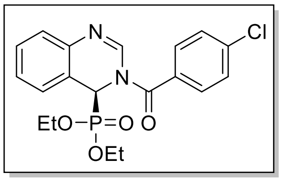

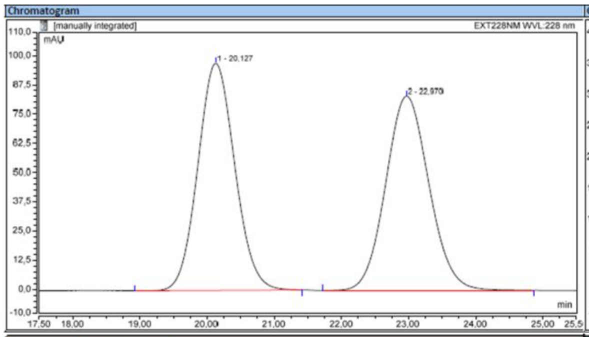

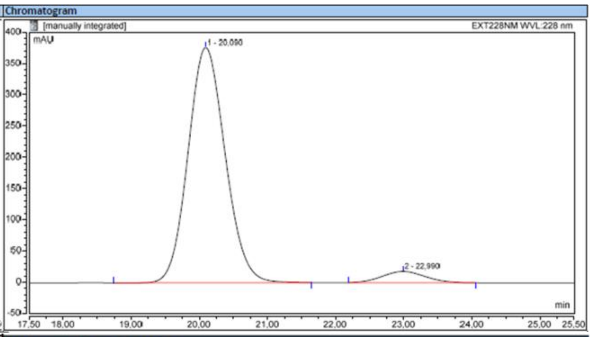

Integration Results

No.	Retention Time min	Area mAU*min	Relative Area %
1	20,127	61,875	49,97
2	22,970	61,962	50,03

Integration Results

No.	Retention Time min	Area mAU*min	Relative Area %
1	20,090	237,723	94,80
2	22,990	13,028	5,20

***tert*-Butyl (*S*)-3-(4-chlorobenzoyl)-2-(diethoxyphosphoryl)-2,3-dihydro-1*H*-imidazole-1-carboxylate [(*S*)-11xA]:** Following the general procedure **13**, starting from *tert*-butyl 1*H*-imidazole-1-carboxylate (**1x**) (34 mg, 0.2 mmol) and *tert*-butyldimethylsilyl diethyl phosphite (**2A**) (60 μL, 0.22 mmol), (*S*)-**11xA** was obtained after purification by flash chromatography (*n*-hexane/EtOAc 1/2) as a colorless oil (68 mg, 76%, 92% ee). **$[\alpha]_D^{23}$** = +84.4 (*c* 1, $CHCl_3$). **^{1}H-NMR** (300 MHz, $CDCl_3$): δ 7.55 – 7.52 (m, 2H), 7.42 – 7.38 (m, 2H), 6.51 – 6.07 (m, 3H), 4.27 – 4.09 (m, 4H), 1.51 (s, 9H), 1.35 – 1.29 (m, 6H). **^{13}C-NMR** (75.5 MHz, $CDCl_3$): δ 164.2, 149.3, 137.3, 132.2, 129.3, 129.0, 115.8, 113.1, 82.8 – 82.3 (m), 71.8 – 68.8 (m), 63.2, 28.1, 16.6 – 16.5 (m). **^{31}P-NMR** (122 MHz, $CDCl_3$): The compound exists as a ~1.5:1 mixture of carbamate rotamers. *Signal corresponding to the major rotamer:* δ 15.86. *Signal corresponding to the minor rotamer:* δ 14.39. **HRMS** (ESI): *m/z* calcd for $C_{19}H_{26}O_6N_2ClNaP$ [M^++Na] 467.1109, found 467.1104. **HPLC** (Chiralpak IA, *n*-hexane/propan-2-ol 90:10, flow 1 mL/min) t_R 14.7 min (minor) and 16.5 min (major).

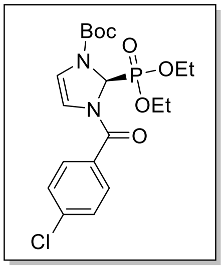

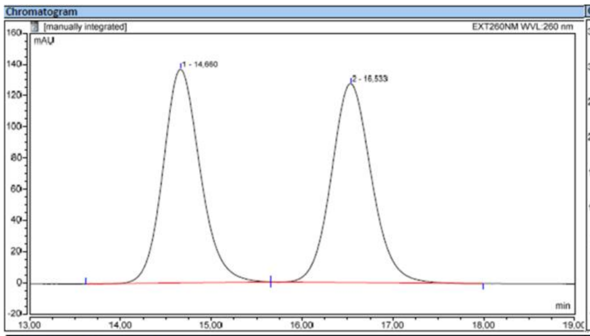

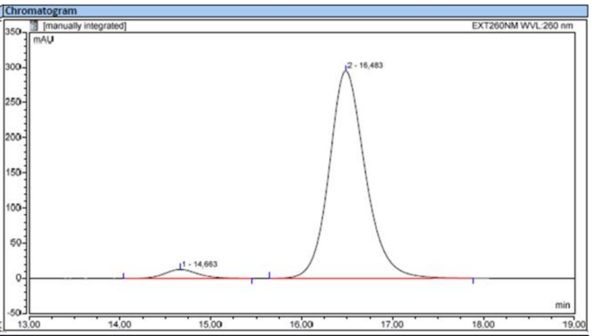

Integration Results

No.	Retention Time min	Area mAU*min	Relative Area %
1	14,660	64,624	49,82
2	16,533	65,089	50,18

Integration Results

No.	Retention Time min	Area mAU*min	Relative Area %
1	14,663	5,538	3,91
2	16,483	136,200	96,09

Diethyl (*S*)-[2-(4-chlorobenzoyl)-1,2-dihydroisoquinolin-1-yl]phosphonate [(*S*)-11yA]: Following the general procedure **13**, starting from isoquinoline (**1y**) (25 µL, 0.2 mmol) and *tert*-butyldimethylsilyl diethyl phosphite (**2A**) (60 µL, 0.22 mmol), (*S*)-**11yA** was obtained after purification by flash chromatography (*n*-hexane/EtOAc 1/1) as a colorless oil (63 mg, 77%, 75% ee). **$[\alpha]_D^{23}$** = +408.8 (*c* 1, $CHCl_3$). **^{1}H-NMR** (300 MHz, $CDCl_3$): δ 7.48 – 7.38 (m, 4H), 7.33 – 7.23 (m, 3H), 7.09 – 7.07 (m, 1H), 6.50 (d, *J* = 7.7 Hz, 1H), 6.30 (d, *J* = 17.6 Hz, 1H), 5.86 (d, *J* = 7.7 Hz, 1H), 4.10 – 3.82 (m, 4H), 1.22 – 1.14 (m, 6H). **^{13}C-NMR** (75.5 MHz, $CDCl_3$): δ 167.5, 137.0, 132.5, 131.1 (d, *J* = 4.0 Hz), 129.8, 128.9, 128.8 (d, *J* = 3.3 Hz), 127.9 – 127.7 (m), 126.6, 126.5 (d, *J* = 2.6 Hz), 125.1 (d, *J* = 2.9 Hz), 111.1, 63.2 – 63.1 (m), 52.9 (d, *J* = 149.2 Hz), 16.5 – 16.4 (m). **^{31}P-NMR** (122 MHz, $CDCl_3$): δ 18.97. **HRMS** (ESI): *m/z* calcd for $C_{20}H_{21}O_4NClNaP$ $[M^+ + Na]$ 428.0789, found 428.0779. **HPLC** (Chiralpak IA, *n*-hexane/propan-2-ol 90:10, flow 1 mL/min) t_R 17.9 min (minor) and 30.1 min (major).

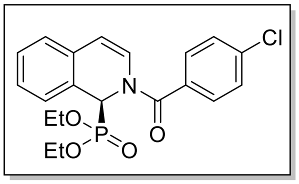

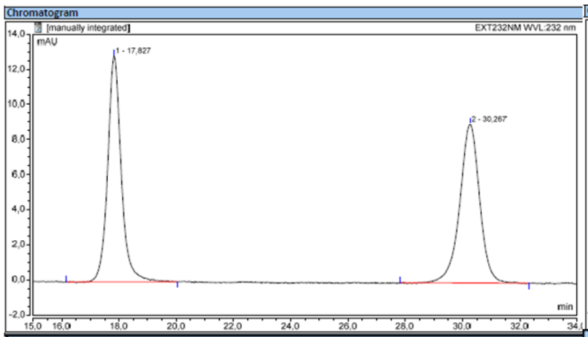

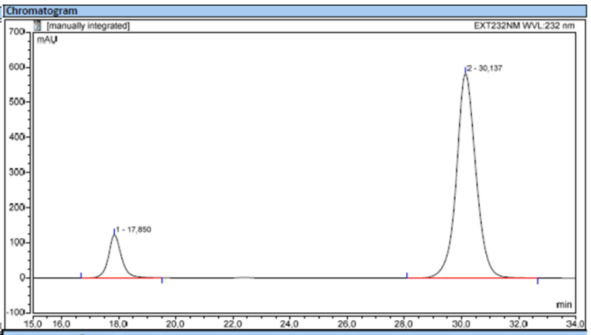

Integration Results

No.	Retention Time min	Area mAU*min	Relative Area %
1	17,827	7,057	49,28
2	30,267	7,263	50,72

Integration Results

No.	Retention Time min	Area mAU*min	Relative Area %
1	17,850	65,043	12,35
2	30,137	461,690	87,65

14. Derivatizations.

14.1. Synthesis of diethyl (*S*)-[2-(4-chlorobenzoyl)-1,2,3,4-tetrahydrophthalazin-1-yl]phosphonate [(*S*)-16].

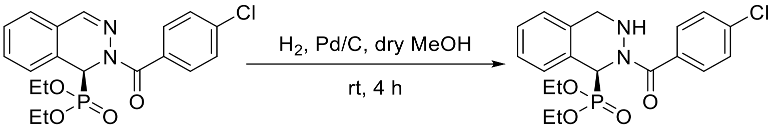

(*S*)-**11aA** (*S*)-**16**

A 25-mL round-bottomed flask was charged with Pd/C (10% w/w) (43 mg, 0.04 mmol), dry MeOH (2 mL), (*S*)-**11aA** (81 mg, 0.2 mmol, 95% ee) and sealed with a rubber septum. The headspace was evacuated and back-filled with hydrogen three times and then stirred under a balloon of hydrogen for 4 h at room temperature. After this time, the mixture was filtered through a Celite pad, and the solvent was removed under reduced pressure. The residue was purified by flash chromatography (*n*-hexane/EtOAc 1/1) to afford (*S*)-**16** as a colorless oil (58 mg, 71%, 95% ee). **$[\alpha]_D^{27}$ =** +107.9 (*c* 1, Acetone). **^{1}H-NMR** (300 MHz, Acetone-d_6): δ 7.74 – 7.70 (m, 2H), 7.52 – 7.44 (m, 3H), 7.32 – 7.26 (m, 2H), 7.18 – 7.13 (m, 1H), 6.07 (d, *J* = 19.8 Hz, 1H), 5.54 – 5.49 (m, 1H), 4.25 – 3.97 (m, 6H), 1.30 – 1.21 (m, 6H). **^{13}C-NMR** (75.5 MHz, Acetone-d_6): δ 170.0, 136.1, 135.1, 134.6 (d, *J* = 5.5 Hz), 131.3, 129.7, 128.6, 128.5, 127.20, 127.18, 127.1, 63.9 (d, *J* = 7.0 Hz), 63.8 (d, *J* = 7.1 Hz), 49.3 (d, *J* = 152.6 Hz), 49.2, 16.8 (d, *J* = 5.4 Hz), 16.7 (d, *J* = 5.6 Hz). **^{31}P-NMR** (122 MHz, Acetone-d_6): δ 19.76. **HRMS** (ESI) *m/z:* calcd for $C_{19}H_{22}O_4N_2ClNaP$ $[M^+ + Na]$ 431.0898, found 431.0888. **HPLC** (Chiralpak IB, *n*-hexane/propan-2-ol 95:5, flow 1 mL/min) t_R 15.7 min (minor) and 17.0 min (major).

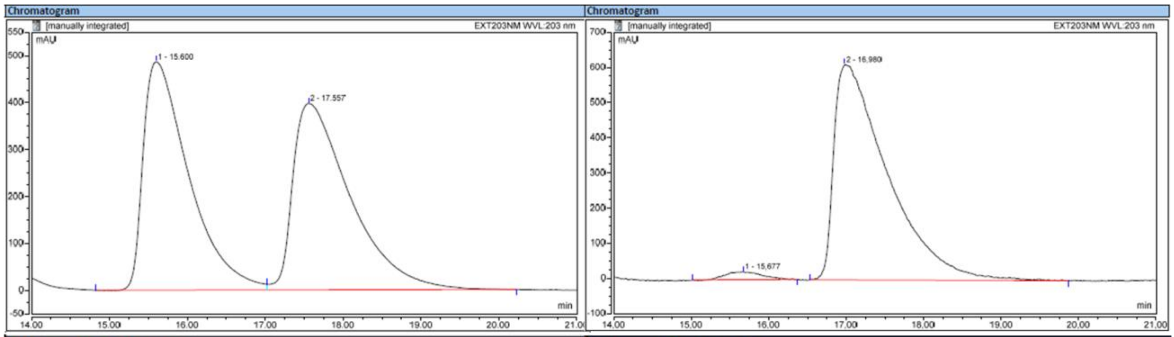

Integration Results

No.	Retention Time min	Area mAU*min	Relative Area %
1	15.600	329.593	49.37
2	17.557	337.980	50.63

Integration Results

No.	Retention Time min	Area mAU*min	Relative Area %
1	15,677	12,048	2,40
2	16,980	490,781	97,60

14.2. Synthesis of diethyl (*S*)-[2-(4-chlorobenzoyl)-4-oxo-1,2,3,4-tetrahydrophthalazin-1-yl]phosphonate [(*S*)-17].

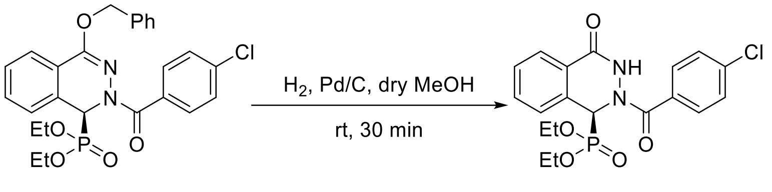

(*S*)-**11kA** (*S*)-**17**

A 25-mL round-bottomed flask was charged with Pd/C (10% w/w) (43 mg, 0.04 mmol), dry MeOH (2 mL), (*S*)-**11kA** (103 mg, 0.2 mmol, 97% ee), and sealed with a rubber septum. The headspace was evacuated and back-filled with hydrogen three times and then stirred under a balloon of hydrogen for 4 h at room temperature. After this time, the mixture was filtered through a Celite pad, and the solvent was removed under reduced pressure. The residue was purified by flash chromatography (*n*-hexane/EtOAc 1/1) to afford (*S*)-**17** as a white foam (58 mg, 69%, 96% ee). **$[\alpha]_D^{25}$ =** +378.5 (*c* 1, $CHCl_3$). **^{1}H-NMR** (300 MHz, Acetone-d_6): δ 9.78 (br s, 1H), 7.99 (d, *J* = 7.5 Hz, 1H), 7.70 – 7.61 (m, 3H), 7.57 – 7.47 (m, 4H), 6.12 (d, *J* = 17.6 Hz, 1H), 4.16 – 4.00 (m, 4H), 1.22 (q, *J* = 7.1 Hz, 6H). **^{13}C-NMR** (75.5 MHz, Acetone-d_6): δ 169.7, 163.8, 137.4, 135.8, 133.5 (d, *J* = 2.4 Hz), 133.1, 131.0, 129.6 (d, *J* = 3.1 Hz), 129.4, 129.1, 128.8 (d, *J* = 2.1 Hz), 128.4 (d, *J* = 3.8 Hz), 127.7 (d, *J* = 4.5 Hz), 63.9 (d, *J* = 7.1 Hz), 63.8 (d, *J* = 6.8 Hz), 16.7 – 16.6 (m). **^{31}P-NMR** (122 MHz, Acetone-d_6): δ 16.59. **HRMS** (ESI) *m/z* calcd for $C_{19}H_{20}O_5N_2ClNaP$ $[M^+ + Na]$ 445.0691, found 445.0677. **HPLC** (Chiralpak IA, *n*-hexane/propan-2-ol 80:20, flow 1 mL/min) t_R 11.0 min (major) and 17.4 min (minor).

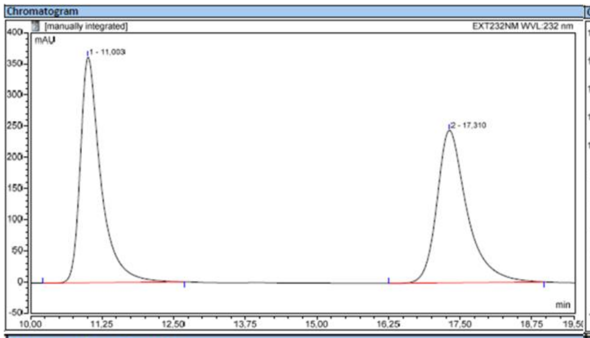

Integration Results

No.	Retention Time min	Area mAU*min	Relative Area %
1	11,003	150,211	50,25
2	17,310	148,733	49,75

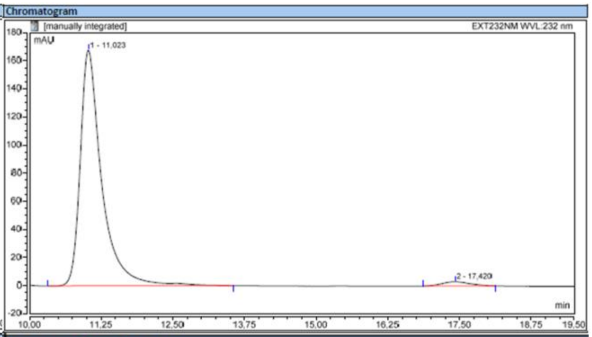

Integration Results

No.	Retention Time min	Area mAU*min	Relative Area %
1	11,023	72,722	97,76
2	17,420	1,667	2,24

14.3. Synthesis of (*R*)-[2-(4-chlorobenzoyl)-1,2-dihydrophthalazin-1-yl]phosphonic acid [(*R*)-18].

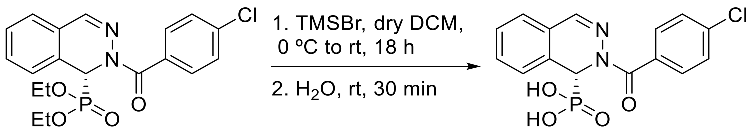

(*R*)-**11aA** (*R*)-**18**

TMSBr (79 µL, 0.6 mmol) was added to a solution of (*R*)-**11aA** (81 mg, 0.2 mmol, 95% ee) in dry DCM (3.5 mL) under argon atmosphere at 0 °C. The reaction mixture was allowed to warm to room temperature and stirred for 18 h. After this time, H_2O (3.5 mL) was added, and the mixture was stirred for 30 min. Then, the aqueous layer was extracted with DCM (3 x 10 mL). The combined organic layers were washed with NaCl (1 x 10 mL), dried over $MgSO_4$, and the solvent was removed under reduced pressure. The solid was washed with pentane and dried in *vacuo* to give (*R*)-**18** as white solid (62 mg, 88%, 95% ee). **$[\alpha]_D^{22}$ =** –536.1 (*c* 0.5, DMSO). **^{1}H-NMR** (300 MHz, DMSO-d_6) δ 7.73 (s, 1H), 7.63 – 7.60 (m, 2H), 7.54 – 7.48 (m, 3H), 7.42 – 7.35 (m, 3H), 6.18 (d, *J* = 17.2 Hz, 1H). **^{13}C-NMR** (126 MHz, DMSO-d_6): δ 167.6, 144.3, 134.5, 134.1, 131.5, 130.8, 129.2, 128.3, 127.6, 127.4 (d, *J* = 4.4 Hz), 125.9, 124.5 (d, *J* = 3.6 Hz), 50.7 (d, *J* = 146.8 Hz). **^{31}P-NMR** (122 MHz, DMSO-d_6): δ 12.89. **HRMS** (ESI): *m/z* calcd for $C_{15}H_{12}O_4N_2ClNaP$ [M^++Na] 373.0115, found 373.0109.

*The enantiomeric excess was determined after esterification of the phosphonic acid (*R*)-**18** (10 mg, 0.03 mmol, 95% ee) with $CH(OEt)_3$ (0.3 mL) at 145 ºC for 1 h. The product (*R*)-**11aA** was purified by preparative TLC (*n*-hexane/EtOAc 1/1), and the enantiomeric excess (ee) was determined by HPLC analysis [(Chiralpak IB, *n*-hexane/propan-2-ol 90:10, flow 1 mL/min) t_R 11.1 min (major) and 13.5 min (minor)].

15. Analysis of the reaction mixture by mass spectrometry.

A representative sample containing *p*-chlorobenzoyl chloride (26 μL, 0.2 mmol) and catalyst **VII** (7 mg, 0.01 mmol, 5 mol%) in MTBE (2 mL, 0.1 M) was analyzed by high-resolution mass spectrometry. Complexes with both 1:1 (2H-activation mode) and 2:1 (4H-activation mode) binding stoichiometries were detected.

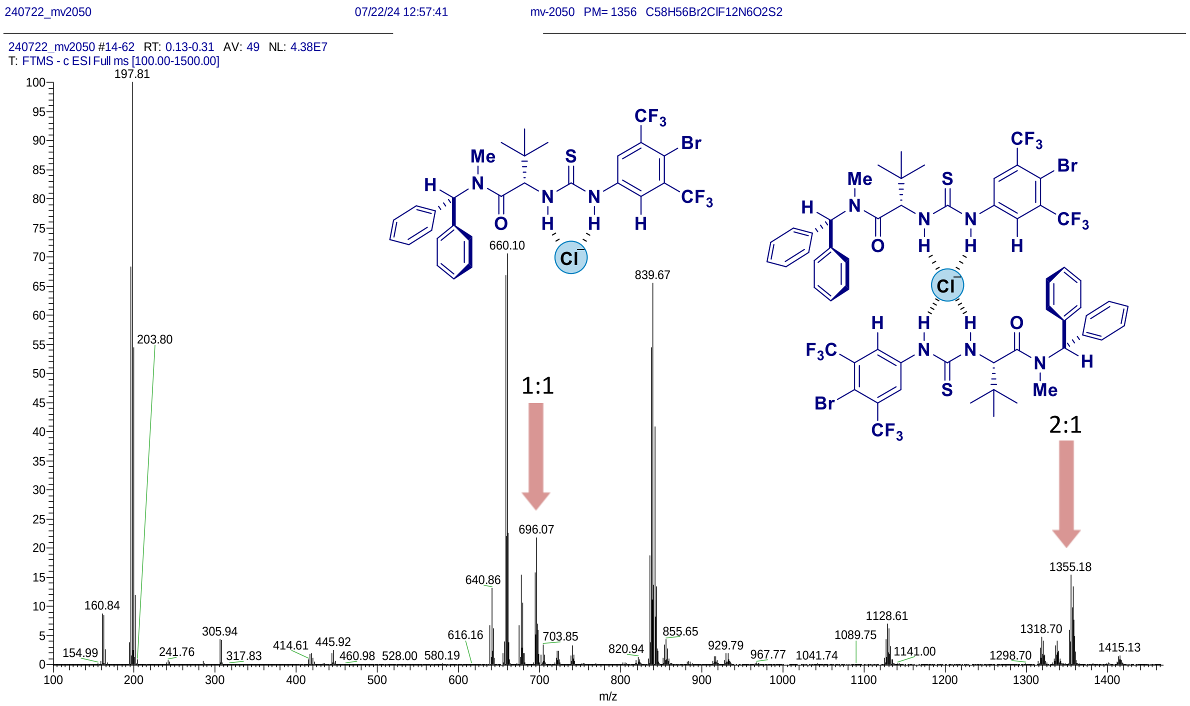

Figure S3. Negative ion ESI-MS spectrum of the first reaction step (ionization performed using MeCN).

16. Non-linear effect experiment.

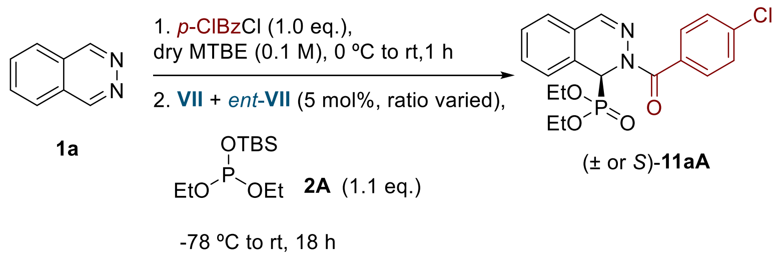

Two stock solutions of catalysts **VII** (*sol.* A) and *ent*-**VII** (*sol.* B) were prepared in dichloromethane using a round-bottom flask equipped with magnetic stir bars (0.015 M, 10 mg/mL; the solutions were prepared by dissolving 50 mg of **VII** or *ent*-**VII** to 5 mL total volume). Once the catalysts were fully dissolved, mixtures of the stock solutions were prepared in vials according to **Table S5**. The solvents were then removed under reduced pressure, resulting in catalyst **VII** with varying enantiomeric excesses.

Subsequently, in a flame-dried Schlenk flask, *p*-chlorobenzoyl chloride (13 µL, 0.1 mmol) was added to a solution of phthalazine (**1a**) (13 mg, 0.1 mmol) in freshly distilled MTBE (1 mL, 0.1 M) at 0 ºC. The resulting suspension was stirred for 1 h at room temperature. Then, catalyst **VII** (3 mg, 0.005 mmol, 5 mol%) was added, and the reaction was cooled to −78 ºC (dry ice/acetone bath). *Tert*-butyldimethylsilyl diethyl phosphite (**2A**) (30 µL, 0.11 mmol) was added, and the reaction mixture was stirred for 18 h and allowed to warm slowly to room temperature. Then, the solvent was removed under reduced pressure. NMR yield was determined by ^{1}H-NMR analysis, and enantiomeric excesses were determined by HPLC analysis.

Table S5. Non-linear effect experiment. Data used to generate **Figure S4**.

Entry	L_{ent} (µL) (*sol.* A)	D_{ent} (µL) (*sol.* B)	ee_{VII} (%)[a]	Yield (%)[b]	ee_{11aA} (%)[c]	ee_{Theor}(%)
**1**	1000	0	100	79	95	100
**2**	900	100	80	82	88	80
**3**	800	200	60	80	81	60
**4**	700	300	40	74	62	40
**5**	600	400	20	79	40	20
**6**	500	500	0 (*rac*)	73	0	0

[a]The preparations of **VII** and *ent*-**VII** are assumed to be enantiomerically pure. [b]Yields were determined by ^{1}H-NMR analysis of the crude reaction mixture using mesitylene as internal standard. [c]Determined by HPLC analysis after isolation of the product by semipreparative TLC (*n*-hexane/EtOAc 1/1).

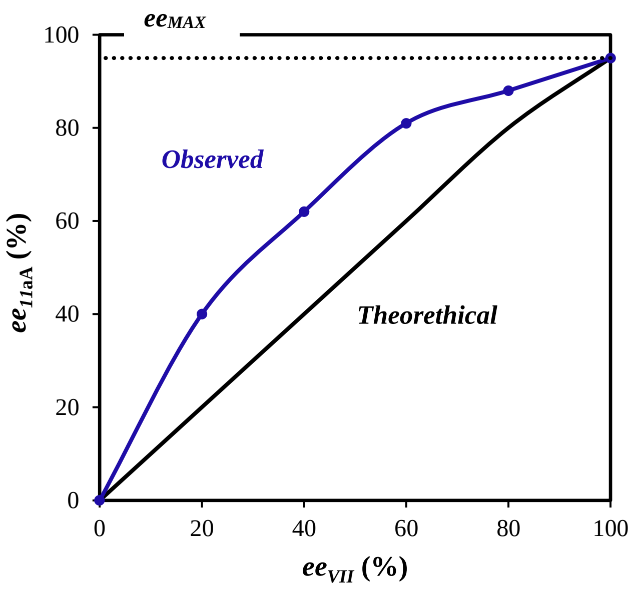

Figure S4. Relationship between catalyst and product enantiomeric excess. The solid blue line represents the regression of the data obtained with catalyst **VII** (**blue circles**). The solid black line represents the linear regression of the reaction. Data is summarized in **Table S5**.

17. ^{1}H-NMR titration experiments.

Following a similar procedure reported by Wezenberg,[22] two solutions containing catalyst **VII** (host) (0.01 M) were prepared in CD_2Cl_2. *N*-Benzylphthalazinium chloride (***Pht*-1**) (guest) was dissolved in one of these solutions and was present at 0.1 M concentration. The solution containing the chloride salt (***Pht*-1**) was then added stepwise to 0.5 mL of the other catalyst stock solution. After each addition, a ^{1}H-NMR spectrum was recorded. The titration was carried out with 21 data points, from 0 to 6 equiv. of ***Pht*-1**.

The ^{1}H-NMR spectra were recorded on a Bruker AV300 spectrometer and were calibrated to the residual CD_2Cl_2 solvent peak. All compounds were dried under high vacuum prior to use.

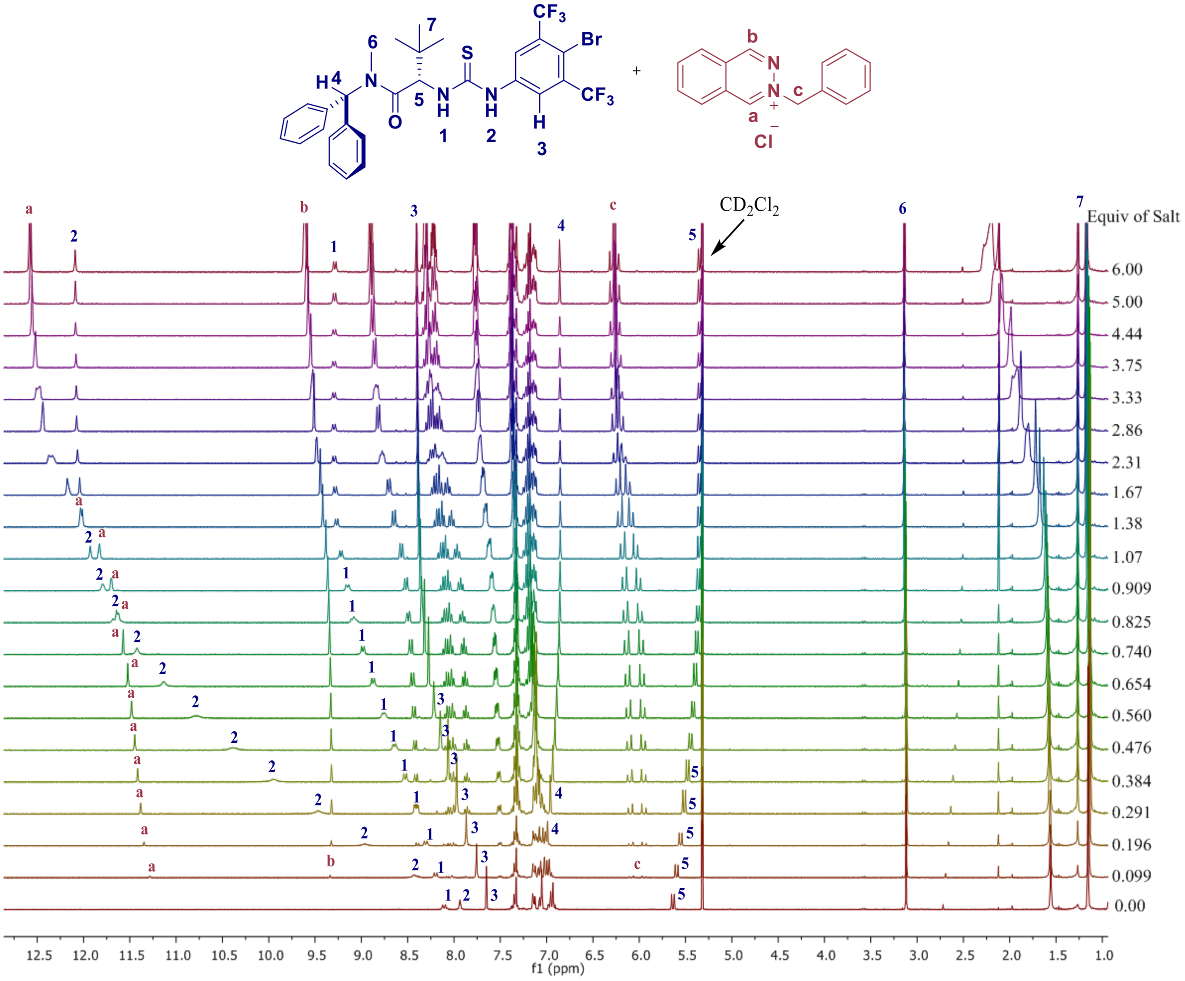

Figure S5. ^{1}H-NMR (300 MHz) chemical shifts (δ) of **VII** (0.01M) in CD_2Cl_2 + 0.0-6.0 equiv. of ***Pht*-1**.

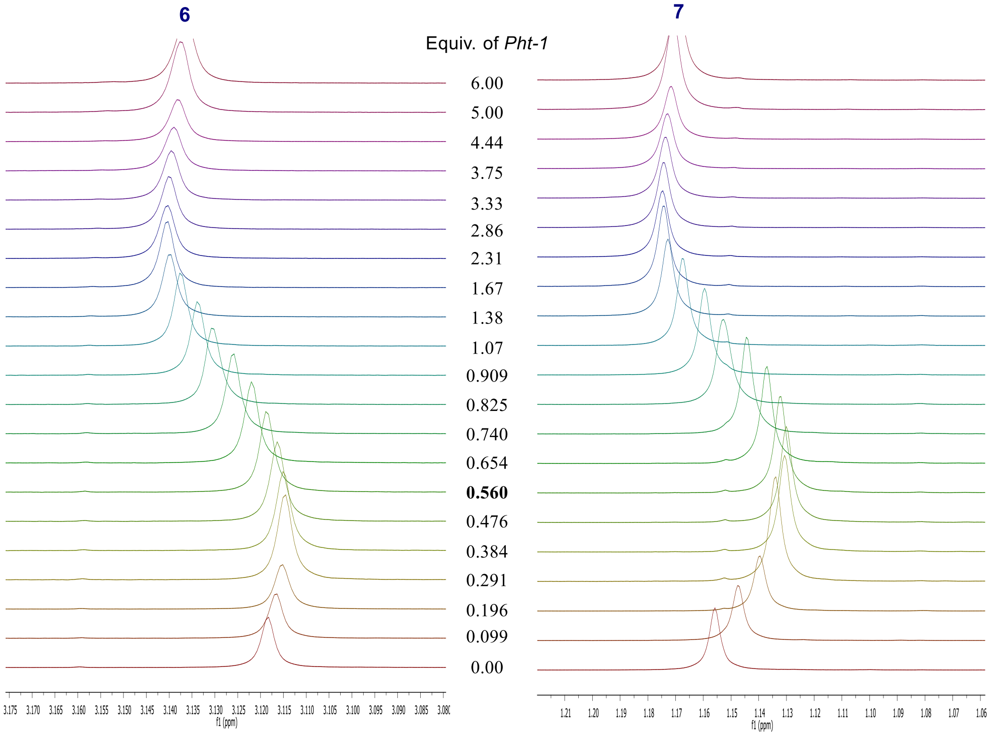

Figure S6. Partially stacked ^{1}H-NMR (300 MHz, CD_2Cl_2) spectra from the titration of **VII** with ***Pht*-1**, highlighting signals 6 and 7. An inflection point around 0.5 equiv. of *N*-benzylphthalazinium salt is observed, suggesting the formation or consumption of different species.

.

18. Data analysis for binding model determination.

Three different proton resonances were recorded from the ^{1}H-NMR titration studies, providing three sets of data from which the association constants can be determined by nonlinear regression using BindFit v0.5 software.[23] Binding isotherms for both 1:1 and 2:1 host-guest complexes were modeled, and the optimal fit was selected based on the criteria established by Thordarson.[24]

Table S6. Data values from the titration of **VII** (host) with ***Pht*-1** (guest).

[VII] M	[*Pht-1*] M	δ_{CH} (ppm)	δ_{CH} (ppm)	δ_{CH} (ppm)
0,01	0	7,963	8,111	7,651
0,01	0,00099	8,432	8,202	7,754
0,01	0,00196	8,963	8,301	7,867
0,01	0,00291	9,467	8,406	7,972
0,01	0,00384	9,96	8,531	8,064
0,01	0,00476	10,407	8,639	8,148
0,01	0,0056	10,792	8,762	8,216
0,01	0,00654	11,146	8,869	8,276
0,01	0,0074	11,422	8,988	8,319
0,01	0,00825	11,679	9,084	8,351
0,01	0,00909	11,794	9,151	8,377
0,01	0,0107	11,927	9,221	8,385
0,01	0,0138	12,013	9,265	8,389
0,01	0,0167	12,042	9,284	8,391
0,01	0,0231	12,065	9,293	8,394
0,01	0,0286	12,074	9,296	8,396
0,01	0,0333	12,097	9,296	8,398
0,01	0,0375	12,081	9,293	8,4
0,01	0,0444	12,085	9,293	8,402
0,01	0,05	12,088	9,289	8,404
0,01	0,06	12,091	9,289	8,404

Table S7. Association constants of host **VII** towards ***Pht*-1** obtained from ^{1}H-NMR titrations (300 MHz) of **VII** with ***Pht*-1** (from 0 to 6.0 equiv.) in CD_2Cl_2; and comparison of binding models.

**Binding model**	***cov*$_{fit}$**	***cov*$_{fit}$ factor**[a]	**K_{11} (M^{-1})**	**K_{21} (M^{-1})**	**β_{21} (M^{-1})**[b]	**α**[c]
**1:1**	1.024×10^{-2}	1	1.8×10^{6} (±763%)	-	-	-
**Full 2:1**	3.84×10^{-4}	26.6	363 (±5%)	–49[d] (±1%)	-	-
**Non-cooperative 2:1**	6.208×10^{-3}	1.6	129 (±3%)	32	4.12×10^{3}	1
**Additive 2:1**	1.6×10^{-3}	6.4	3.1×10^{5} (±126%)	17 (±14%)	5.33×10^{6}	0.0022
**Statistical 2:1**	7.71×10^{-3}	1.3	296 (±6%)	74	2.2×10^{5}	1

[a]The relative quality of fit; Cov_{fit} factor = Cov_{fit} 1:1/Cov_{fit} of the binding model under study. To select the best fit, it is important to significantly improve the Cov_{fit} factor (>2 fold) when comparing higher-complexity models to the simplest 1:1 model. [b]The overall association constant $\beta_{21} = K_1 \times K_2$. [c]The interaction parameter $\alpha = 4K_2/K_1$ with $\alpha > 1$ indicating positive cooperativity, $\alpha < 1$ negative cooperativity, and $\alpha = 1$ no cooperativity. [d]A negative association constant was obtained, hence calculation for β_{21} and α are excluded.

The 2:1 full binding model provides the best fit; however, the fitting process yielded a nonrealistic negative value for K_2, which suggests that different complexes may be formed. Additionally, a value of K much larger than 10^5 M^{-1} should be considered unreliable for NMR titration experiments. Therefore, despite the small difference in Cov_{fit}, the non-cooperative 2:1 binding model appears to be the most appropriate for describing the system.

19. Characterization of complexes.

19.1. Complex VII:*Pht*-1 (2:1).

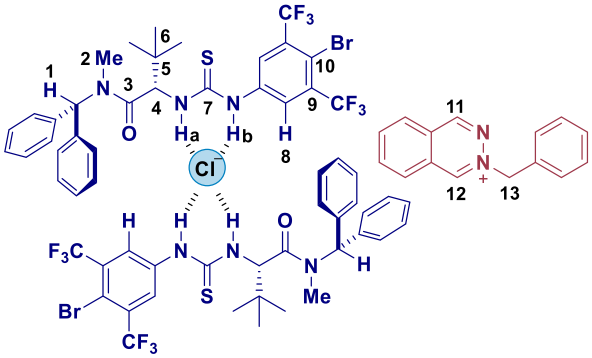

The complex **VII:*Pht*-1** (2:1) was fully characterized using a sample prepared by dissolving **VII** (13 mg, 0.02 mmol) and ***Pht*-1** (3 mg, 0.01 mmol) in CD_2Cl_2 (0.5 mL).

Characterization data:

^{1}H-NMR (500 MHz, CD_2Cl_2): δ 11.29 (s, 1H, H_{12}), 10.41 (br s, 2H, N<u>H</u>$_b$), 9.31 (s, 1H, H_{11}), 8.65 (d, J = 8.6 Hz, 2H, N<u>H</u>$_a$), 8.36 (d, J = 7.9 Hz, 1H, H_{Ar}), 8.15 (s, 4H, H_8), 8.06 – 8.02 (m, 1H, H_{Ar}), 7.97 (d, J = 7.7 Hz, 1H, H_{Ar}), 7.85 – 7.82 (m, 1H, H_{Ar}), 7.49 – 7.47 (m, 2H, H_{Ar}), 7.36 – 7.27 (m, 9H, H_{Ar}), 7.15 – 7.06 (m, 14H, H_{Ar}), 6.90 (s, 2H, H_1), 6.07 (d, J = 13.6 Hz, 1H, H_{13}), 5.94 (d, J = 13.6 Hz, 1H, $H_{13'}$), 5.45 (d, J = 8.6 Hz, 2H, H_4), 3.12 (s, 6H, H_2), 1.12 (s, 18H, H_6).

^{13}C-NMR (126 MHz, CD_2Cl_2): δ 182.1 (C_7), 173.1 (C_4), 154.3 (C_{11}), 152.1 (C_{12}), 140.3 (C_{Ar}), 139.9 (C_{Ar}), 139.2 (C_{Ar}), 138.8 (C_{Ar}), 136.2 (C_{Ar}), 133.3 (C_{Ar}), 132.3 (q, $J_{C,F}$ = 31.0 Hz) (C_9), 131.8 (C_{Ar}), 130.3 (C_{Ar}), 130.2 (C_{Ar}), 130.1 (C_{Ar}), 129.6 (C_{Ar}), 129.1 (C_{Ar}), 128.9 (C_{Ar}), 128.7 (C_{Ar}), 128.4 (C_{Ar}), 128.3 (C_{Ar}), 127.78 (C_{Ar}), 127.76 (C_{Ar}), 127.5 (C_{Ar}), 125.5 (C_{Ar}), 123.1 (q, $J_{C,F}$ = 274.2 Hz) (<u>C</u>F_3), 111.7 (C_{10}), 68.4 (C_{13}), 62.3 (C_4), 62.2 (C_1), 36.5 (C_5), 34.2 (C_2), 27.6 (C_6).

^{19}F-NMR (471 MHz, CD_2Cl_2): δ −62.23 (s, 12F).

HRMS (ESI): *m/z* calcd for $C_{58}H_{56}O_2N_6Br_2ClF_{12}S_2$ [M^-] 1355.1755, found 1355.1772.

[1]**H-NMR** (CD_2Cl_2, 500 MHz) of the **VII:*Pht*-1 (2:1)** complex.

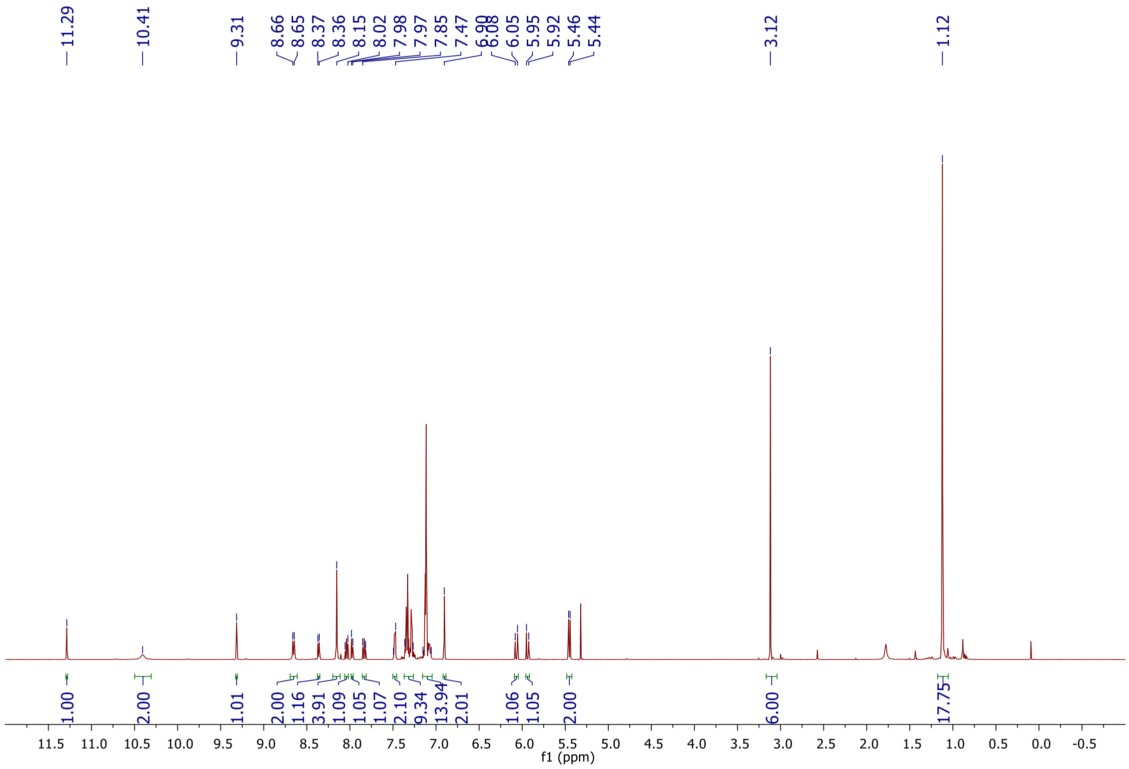

[13]**C-NMR** (CD_2Cl_2, 126 MHz) of the **VII:*Pht*-1 (2:1)** complex.

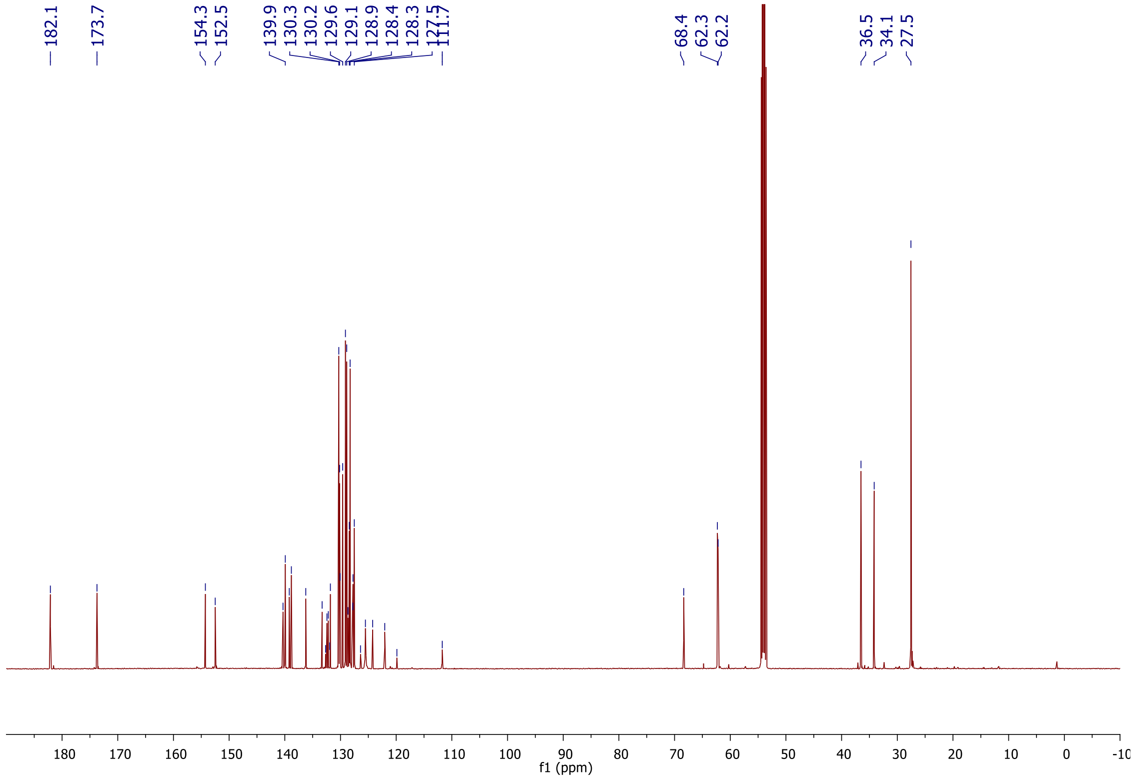

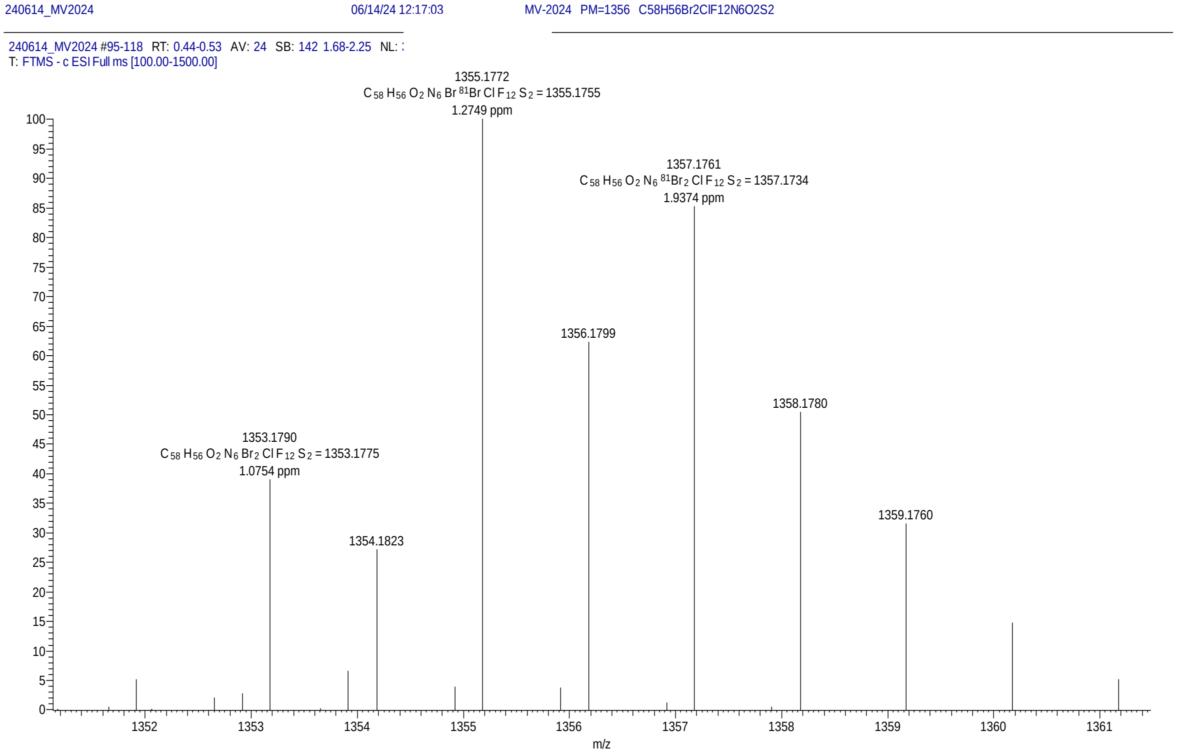

Figure S7. Negative ion ESI-MS spectrum of the **VII:*Pht*-1 (2:1)** complex in DCM, with ionization performed using MeCN.

19.1.1. Bidimensional NOESY and ROESY experiments.

Two sets of NOESY cross-peaks between the *ortho* protons (H_8) and the protons of the *tert*-butyl group (H_6), as well as the aromatic signals of the benzhydryl scaffold, are observed in the NOESY and ROESY experiments in CD_2Cl_2, supporting the antiparallel arrangement of the 2:1 complex.

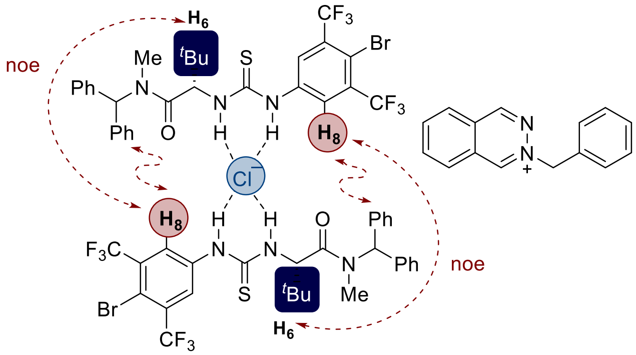

Figure S8. Summary of diagnostic noe correlations.

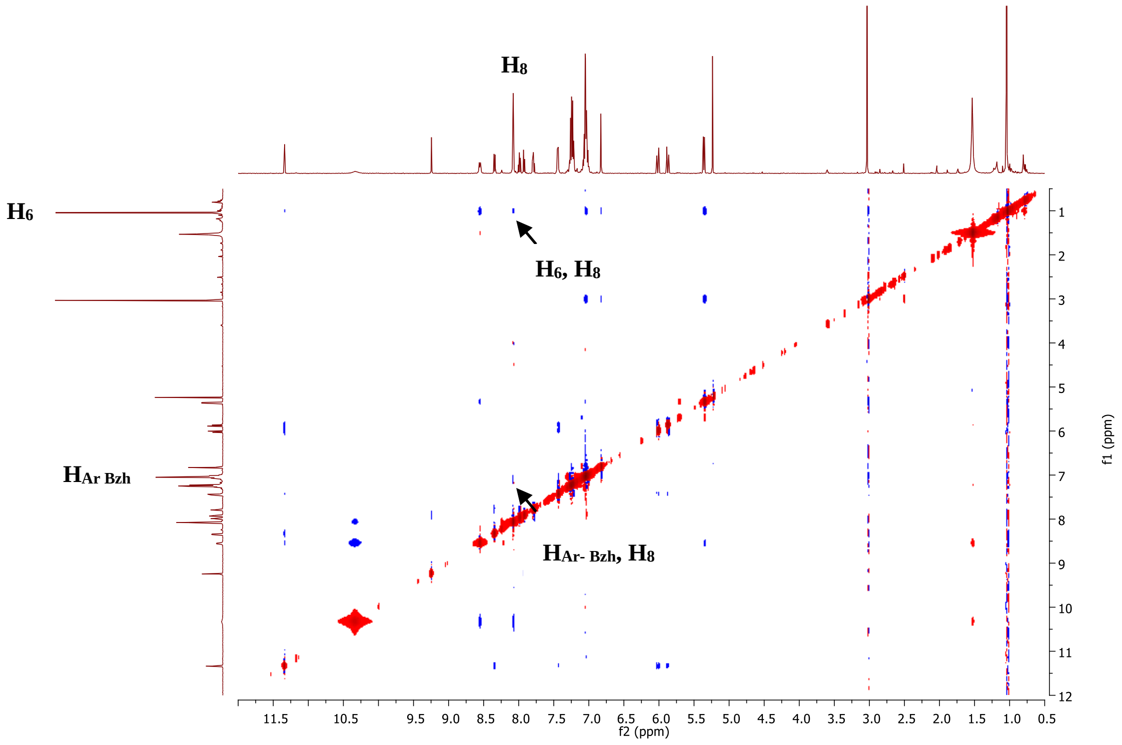

Figure S9. NOESY spectrum of the **VII:*Pht*-1 (2:1)** complex in CD_2Cl_2.

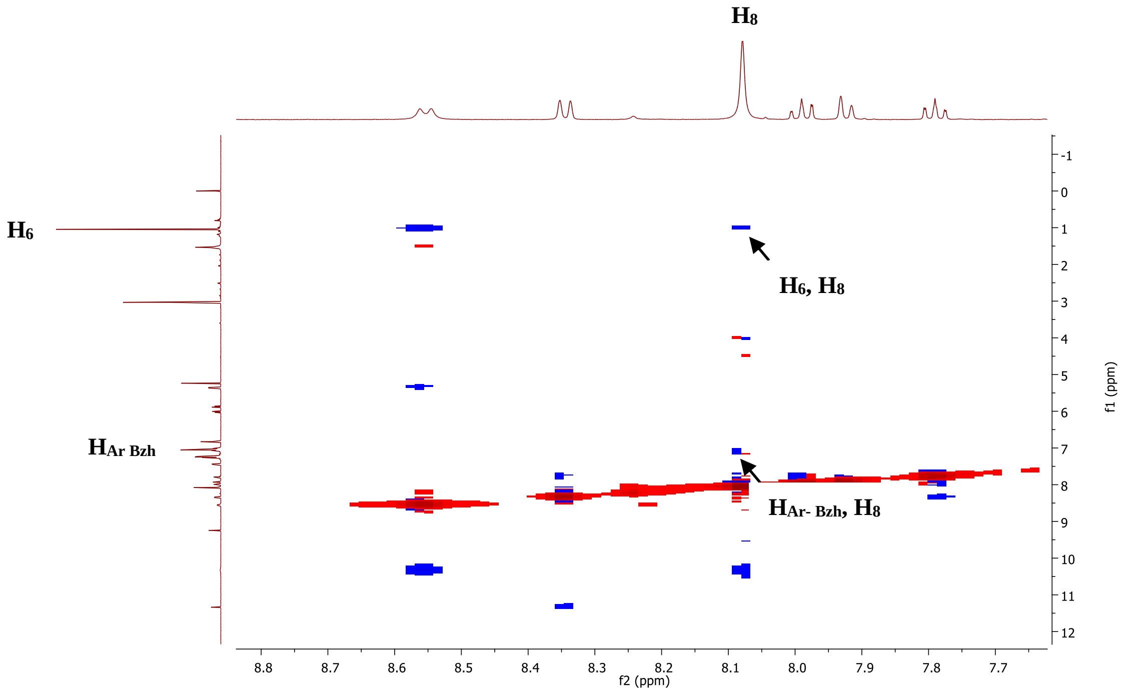

Figure S10. Zoomed-in region of **Figure S9** showing the NOESY spectrum of the **VII:*Pht*-1 (2:1)** complex, highlighting NOE cross-peaks between $H_6 \leftrightarrow H_8$ and $H_{Ar\text{-}Bzh} \leftrightarrow H_8$.

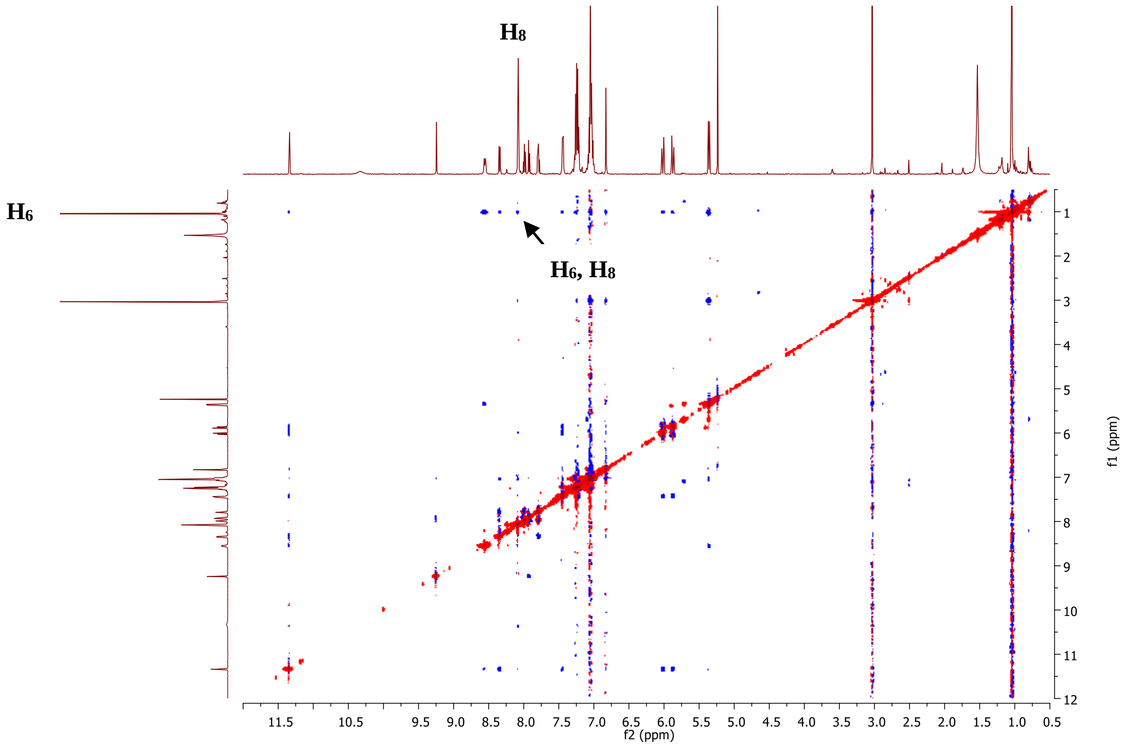

Figure S11. ROESY spectrum of the **VII:*Pht*-1 (2:1)** complex in CD_2Cl_2.

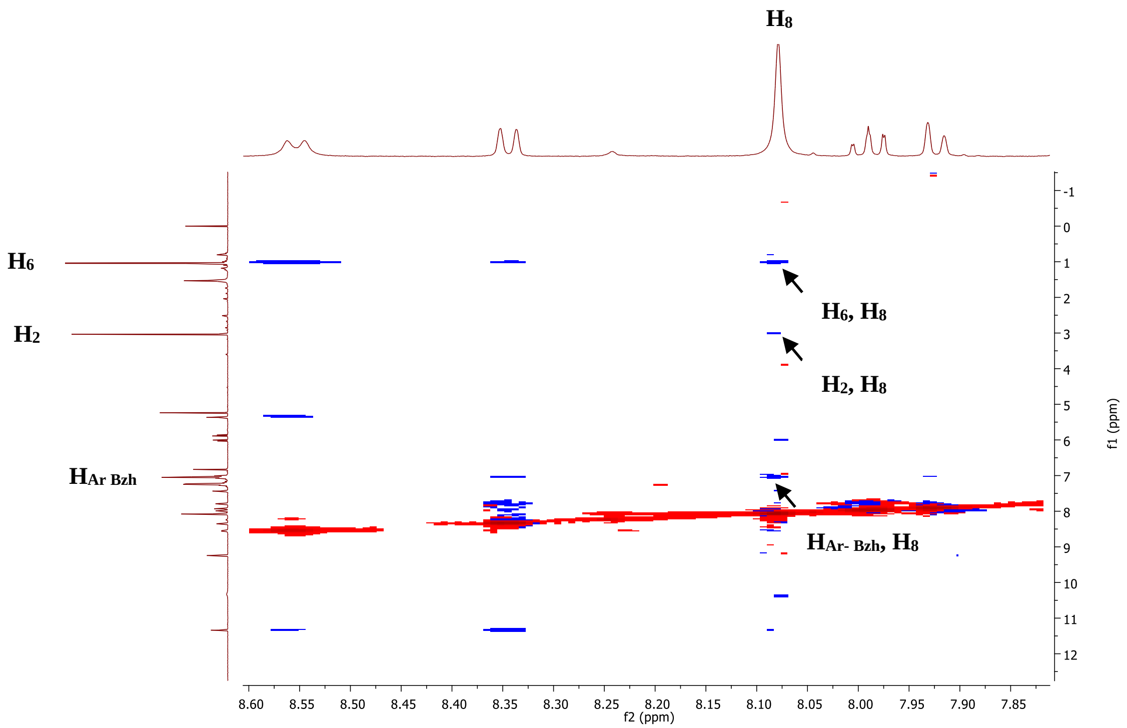

Figure S12. Zoomed-in region of **Figure S11** showing the ROESY spectrum of the **VII:*Pht*-1 (2:1)** complex, highlighting NOE cross-peaks between H_2↔ H_8, H_6↔ H_8, and $H_{Ar\text{-}Bzh}$↔ H_8.

19.2.Complex VII:*Pht*-1 (1:1).

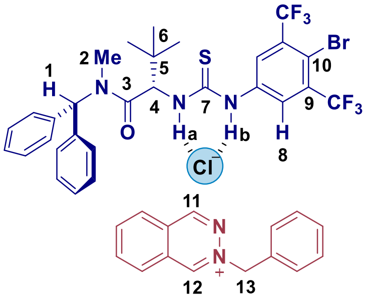

The complex **VII:*Pht*-1** (1:1) was fully characterized using a sample prepared by dissolving **VII** (7 mg, 0.01 mmol) and ***Pht*-1** (3 mg, 0.01 mmol) in CD_2Cl_2 (0.5 mL).

Characterization data:

^{1}H-NMR (500 MHz, CD_2Cl_2): δ 11.77 (br s, 1H, N<u>H</u>$_b$), 11.58 (s, 1H, H_{12}), 9.38 (s, 1H, H_{11}), 9.11 (d, J = 8.6 Hz, 1H, N<u>H</u>$_a$), 8.49 (d, J = 8.2 Hz, 1H, H_{Ar}), 8.32 (s, 2H, H_8), 8.11 (t, J = 7.5 Hz, 1H, H_{Ar}), 8.06 (d, J = 7.8 Hz, 1H, H_{Ar}), 7.91 (t, J = 7.6 Hz, 1H, H_{Ar}), 7.58 – 7.56 (m, 2H, H_{Ar}), 7.37 – 7.29 (m, 6H, H_{Ar}), 7.24 – 7.12 (m, 7H, H_{Ar}), 6.86 (s, 1H, H_1), 6.14 (d, J = 13.6 Hz, 1H, H_{13}), 5.99 (d, J = 13.6 Hz, 1H, $H_{13'}$), 5.37 (d, J = 8.3 Hz, 1H, H_4), 3.13 (s, 3H, H_2), 1.15 (s, 9H, H_6).

^{13}C-NMR (126 MHz, CD_2Cl_2): δ 181.7 (C_7), 173.4 (C_4), 154.2 (C_{11}), 152.7 (C_{12}), 141.0 (C_{Ar}), 140.1 (C_{Ar}), 139.3 (C_{Ar}), 139.2 (C_{Ar}), 136.4 (C_{Ar}), 133.4 (C_{Ar}), 132.1 (q, $J_{C,F}$ = 30.8 Hz) (C_9), 132.0 (C_{Ar}), 130.4 (C_{Ar}), 130.3 (C_{Ar}), 130.1 (C_{Ar}), 129.6 (C_{Ar}), 129.1 (C_{Ar}), 128.9 (C_{Ar}), 128.7 (C_{Ar}), 128.38 (C_{Ar}), 128.35 (C_{Ar}), 128.7 (C_{Ar}), 127.7 (C_{Ar}), 127.5 (C_{Ar}), 123.7 – 123.5 (m) (C_8), 123.2 (q, $J_{C,F}$ = 274.1 Hz) (<u>C</u>F_3), 110.2 (C_{10}), 68.2 (C_{13}), 62.3 (C_4), 62.2 (C_1), 36.5 (C_5), 34.2 (C_2), 27.6 (C_6).

^{19}F-NMR (471 MHz, CD_2Cl_2): δ −62.77 (s, 6F).

HRMS (ESI): *m/z* calcd for $C_{29}H_{28}ON_3BrClF_6S$ [M^-] 696.0714, found 696.0704.

^{1}H-NMR (CD_2Cl_2, 500 MHz) of the **VII:*Pht*-1 (1:1)** complex.

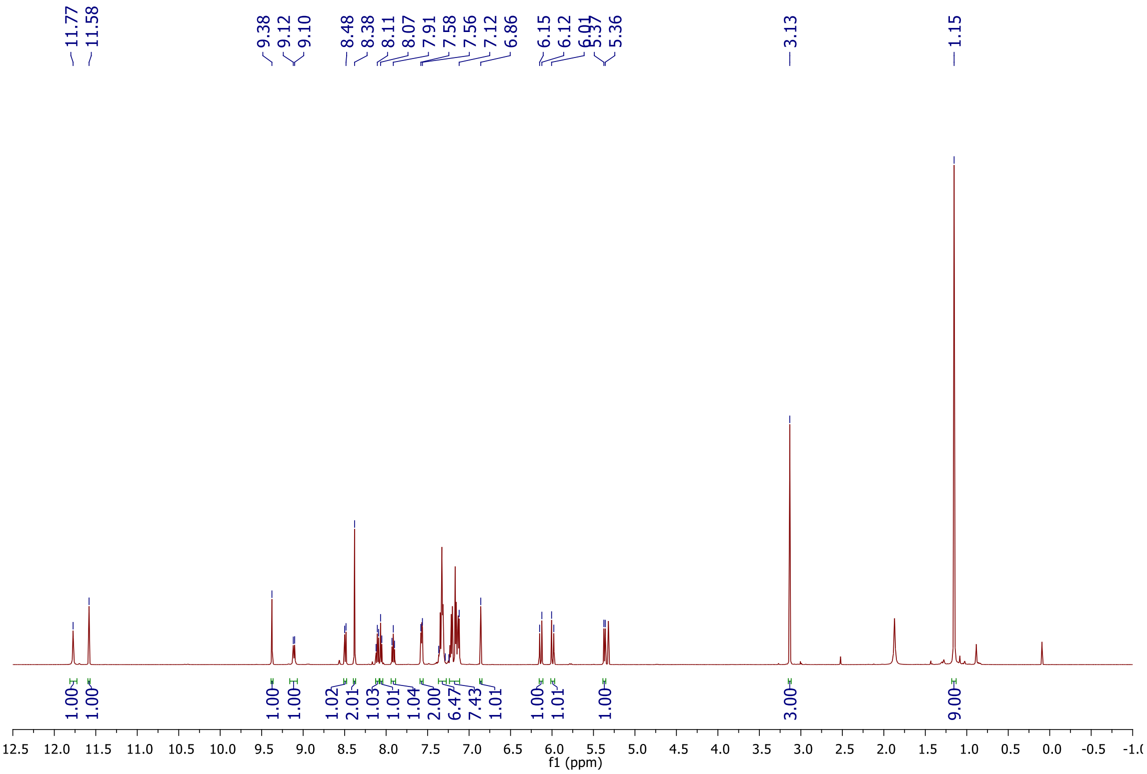

^{13}C-NMR (CD_2Cl_2, 126 MHz) of the **VII:*Pht*-1 (1:1)** complex.

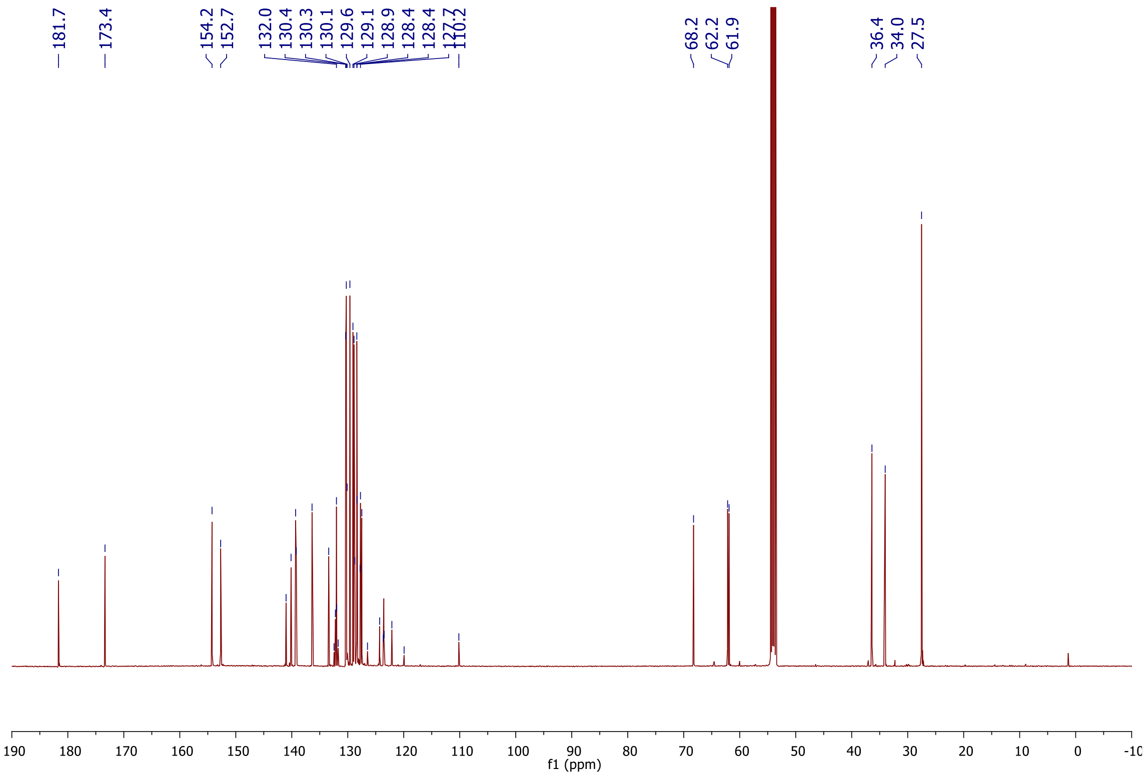

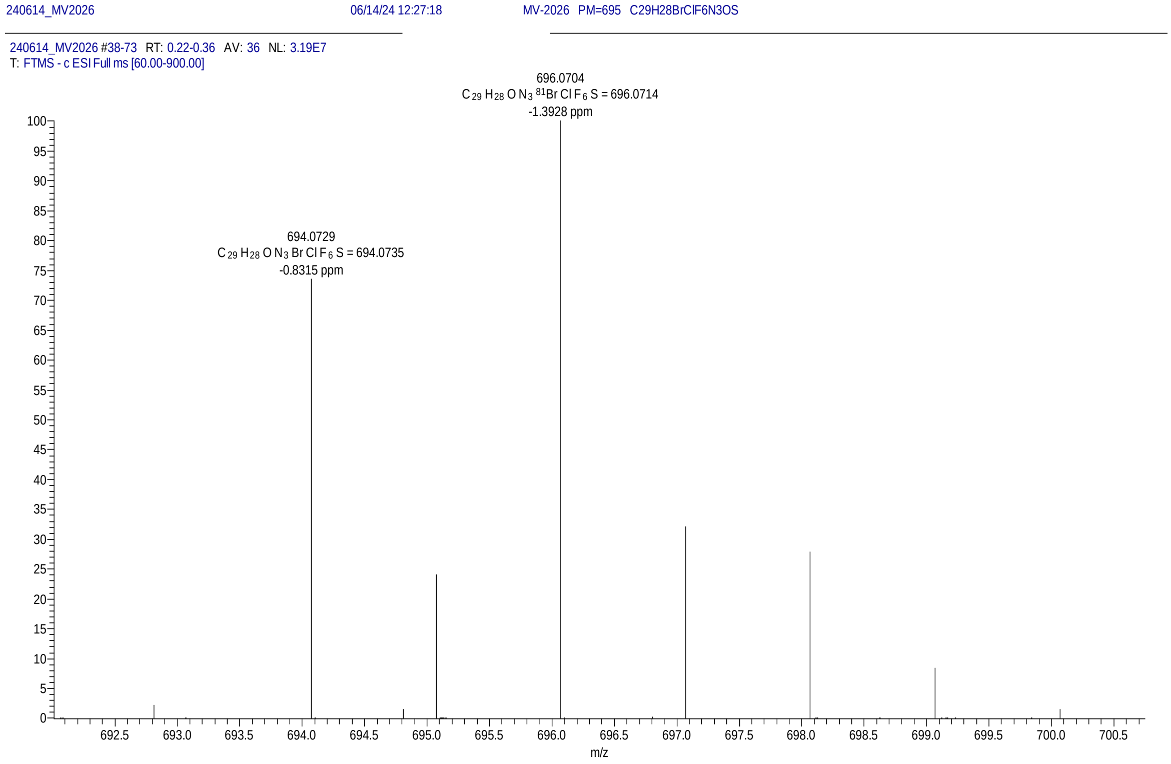

Figure S13. Negative ion ESI-MS spectrum of the **VII:*Pht*-1 (1:1)** complex in DCM, with ionization performed using MeCN.

19.2.1. Bidimensional ROESY experiments.

A very weak NOE cross-interaction between the *ortho* protons (H_8) and the *tert*-butyl group protons (H_6) is observed in the zoomed-in region of the ROESY (**Figure S14**). This evidence suggests that, although the equilibrium is shifting towards the 1:1 complex, the 2:1 complex remains detectable. However, this interaction disappears as the salt concentration increases and is no longer observable in samples prepared in a 1:2 stoichiometry (**Figure S16**).

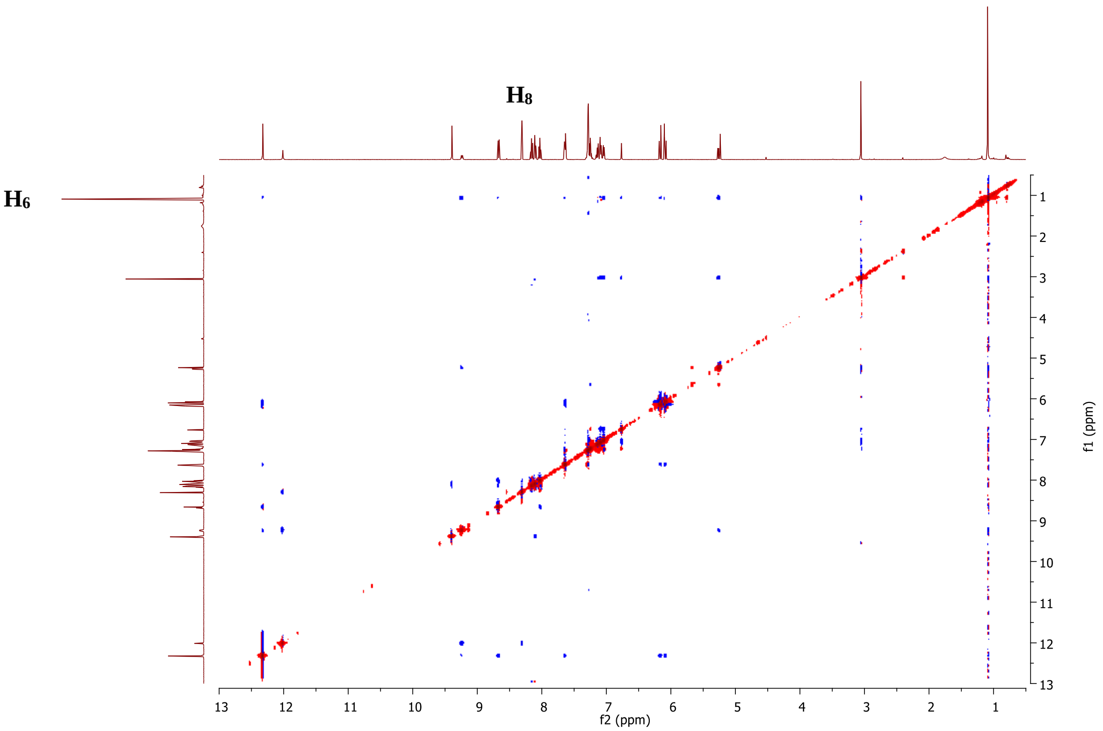

Figure S14. ROESY spectrum of the **VII:*Pht*-1 (1:1)** complex in CD_2Cl_2.

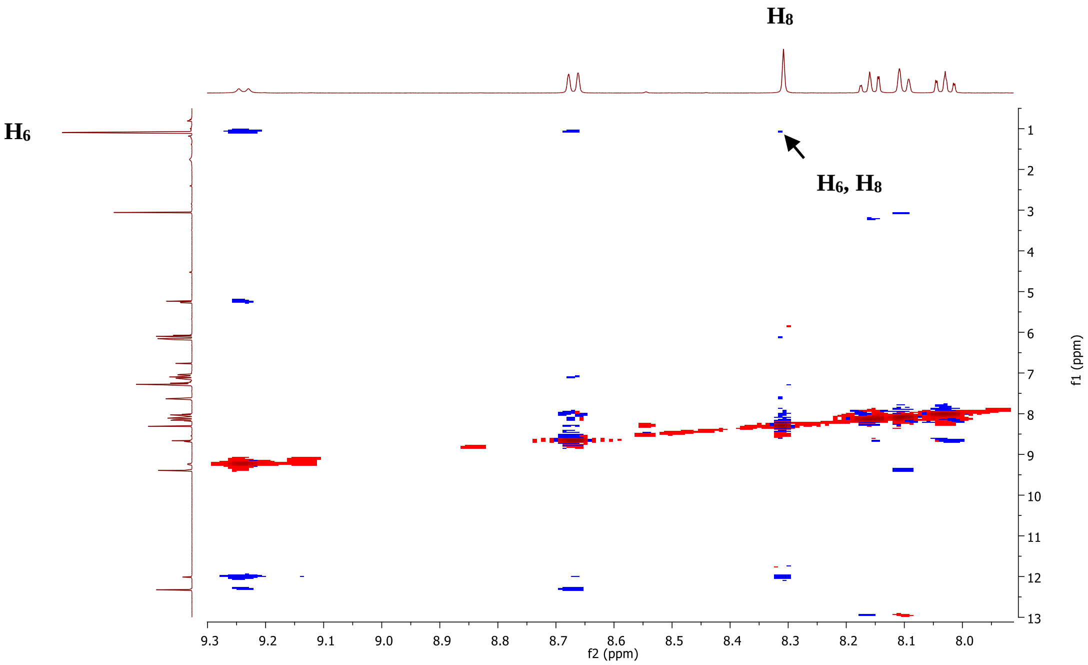

Figure S15. Zoomed-in region of **Figure S14** showing the ROESY spectrum of the **VII:*Pht*-1 (1:1)** complex, highlighting very weak NOE cross peaks between H_6 and H_8.

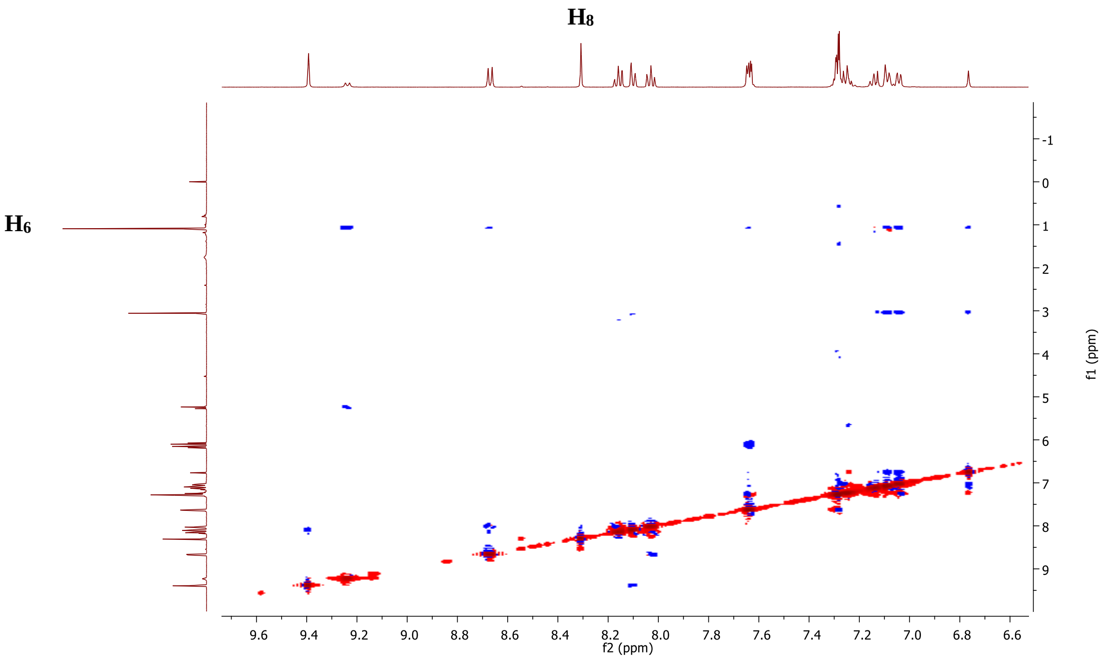

Figure S16. Zoomed-in view of the ROESY spectrum of the **VII:*Pht*-1 (1:2)** complex in CD_2Cl_2, focusing on the H_8 region.

20. Computational studies.
20.1. Computational methods.
20.1.1. Molecular dynamics methods.

MD simulations were carried out with AMBER24 suite of programs.[25] Parameters for the studied compounds were generated with the antechamber module using the general Amber force field (GAFF2),[26] with partial charges calculated using AM1-BCC method. The ff14SB force field[27] was employed in all simulations. The corresponding model was immersed in a solvent box of 12 Å. We used default parameters for chloroform and a solvent box of THF was constructed in house.[2] A two-stage geometry optimization approach was carried out: i) minimization of only the positions of solvent molecules (peptides restrained: k = 500 kcal·mol^{-1}·Å^{-2}) executed by 500 cycles of steepest descent minimization followed by 500 cycles of conjugate gradient minimization and (ii) unrestrained minimization of all the atoms in the simulation cell executed by 2500 cycles of steepest descent minimization followed by 2500 cycles of conjugate gradient minimization. After system optimization, the running of MD simulations was started on the systems by gradually heating each system in the NVT ensemble from 0 to 300 K for 100 ps using a Langevin thermostat with a coupling coefficient of 1.0/ps. Harmonic restraints (k = 10 kcal·mol^{-1}·Å^{-2}) were applied to the solute, and the Langevin temperature coupling scheme[28] was used to control and equalize the temperature. The time step was kept at 2 fs during the heating stages, allowing potential in homogeneities to self-adjust. Water molecules are treated with the SHAKE algorithm such that the angle between the hydrogen atoms is kept fixed. Long-range electrostatic effects are modeled using the particle-mesh-Ewald method.[29] Then 100 ps of density equilibration with a force constant of 2.0 kcal/mol·Å2 on the complex was performed by releasing all the restraints. All simulations used AMBER24 PMEMD CUDA on GPU cards. Production trajectories were then run for the indicated time under the same simulation conditions. Pymol2.0 was used for structural alignments and visualizations.[30] During all MD simulations, replicated 3-5 times to ensure feasibility.

20.1.2. QM methods.

All of the calculations were performed using the Gaussian16program.[31] Computations were done using wb97xd functional[32] in conjunction with standard basis sets def2SVP and def2TZVP.[33] Geometry full optimizations were made at wb97xd/def2SVP level. Single point calculations using def2TZVP basis set were carried out over-optimized geometries to obtain the energy values. Solvent effects (toluene) were considered using the SMD model.[34] The nature of stationary points was defined on the basis of calculations of normal vibrational frequencies (force constant Hessian matrix). The optimizations were carried out using the Berny analytical gradient optimization method.[35] Minimum energy pathways for the reactions studied were found by gradient descent of transition states in the forward and backward direction of the transition vector (IRC analysis).[36] Analytical second derivatives of the energy were calculated to classify the nature of every stationary point, to determine the harmonic vibrational frequencies, and to provide zero-point vibrational energy corrections. The thermal and entropic contributions to the free energies were also obtained from the vibrational frequency calculations using the unscaled frequencies. Correction to free energy was made by substracingS_{trans} contribution and considering a 1M concentration.[37] Structural representations were generated using CYLView.[38]

NCI (non-covalent interactions) were computed using the methodology previously described.[39] Quantitative data were obtained with the NCIPLOT4 program.[40] A density cutoff of ρ=0.5 a.u. was applied and isosurfaces of s(r) =0.5 were colored by $sign(\lambda_2)\rho$ in the [-0.03,0.03] a.u. range using VMD software.[41] s(r) against $sign(\lambda_2)\rho(r)$ plots were generated with gnuplot software.[42]

---

[2]The box solvent was constructed.

20.2. Molecular dynamics studies on aggregation of catalyst and formation of complexes with the phthalazinium chloride.

Molecular dynamics simulations of two thiourea units in chloroform and THF as solvents showed the formation of an aggregate of two thiourea molecules through hydrogen bonding between the amino groups of one molecule and the carbonyl group of the other (referred to as complex **B**). The presence of other complexes, such as complex **A**, is negligible. The **B** arrangement is particularly stable in chloroform, remaining intact for several hundred nanoseconds. However, when introduced to a THF solvent environment, the stability decreases, leading to the disintegration of the complex and resulting in discrete thiourea catalyst molecules that are ready to interact with the substrate (**Figure S17**).

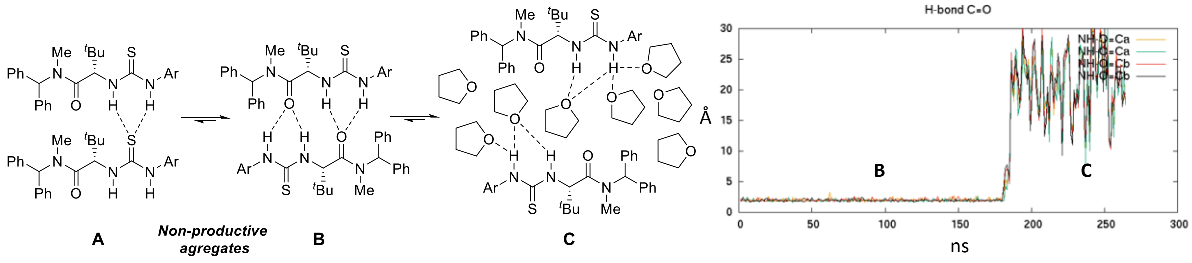

Figure S17. *Left*: Two thioureas aggregate in chloroform mainly with the disposition showed in **B**. In THF, the aggregation is broken by the solvent molecules to form **C**. *Right*: monitoring of H-bond distances in **B** and **C** of an MD simulation.

In fact, when the aggregate of two thioureas is exposed to the reagent (in the form of iminium chloride) in THF as a solvent, the aggregate breaks to form a complex of the two thiourea units with the chloride anion (**Figure S18**).

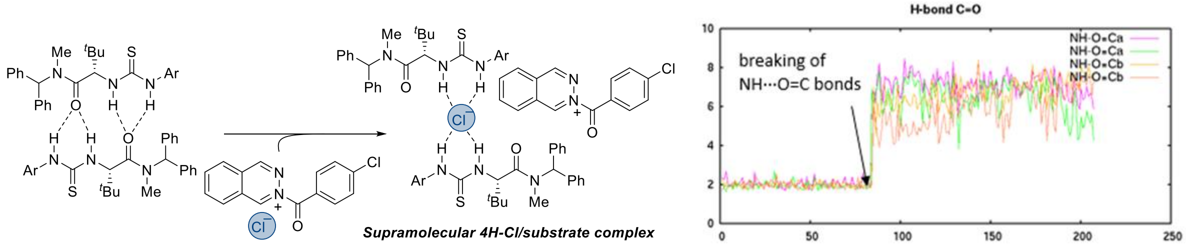

Figure S18. MD simulations of the formation of a complex between two molecules of catalyst and the substrate in THF as solvent.

The structure of the formed complex was analyzed using various MD simulations (up to 10 replicas conducted). These simulations clearly demonstrated the association of the two catalyst molecules with the chloride ion, leading to the formation of an ion pair with 2-acylphthalazinium. The arrangement of the catalyst molecules with respect to the chloride ion occurs in an antiparallel fashion, resulting in an anionic complex with C_2-symmetry (**Figure S19**).

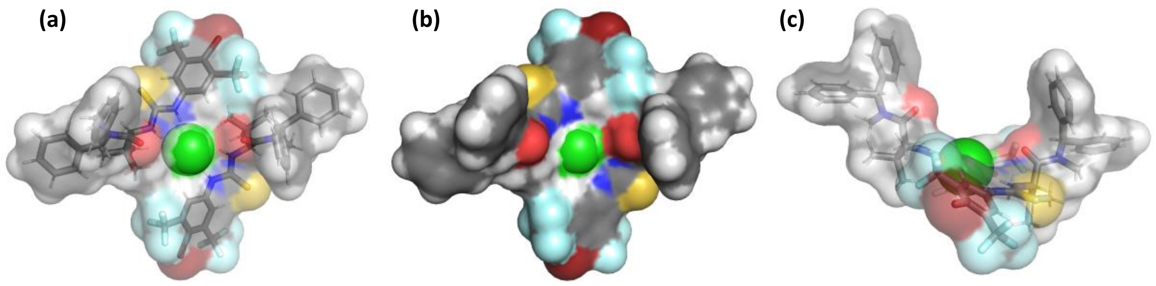

Figure S19. (a) Disposition of the thiourea molecules around de chloride ion. **(b)** View showing the groove of insertion of the phthalazinium ion. **(c)** Surface of the complex showing the C_2-symmetry.

This association features a groove in which the phthalazinium ion is inserted perpendicular to the chloride ion, capable of doing so in principle with two orientations, each showing a different enantiotopic face. There are four distinct approaches, but two are equivalent due to the C_2-symmetry of the complex (**Figures S20** and **S21**).

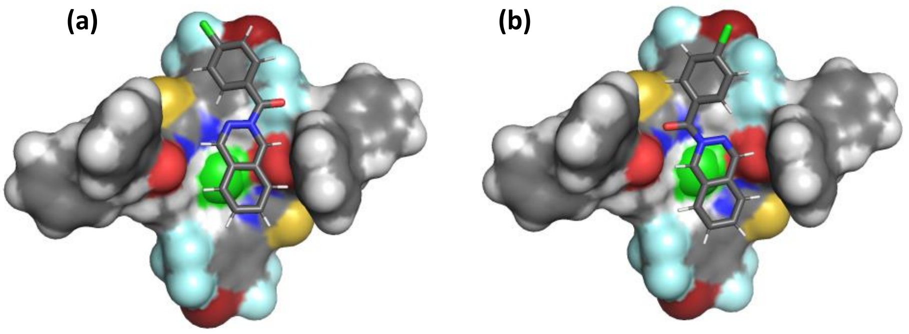

Figure S20. Insertion of the phthalazinium ion into the groove of the anionic complex showing the *Si* face **(a)** and the *Re* face **(b)**.

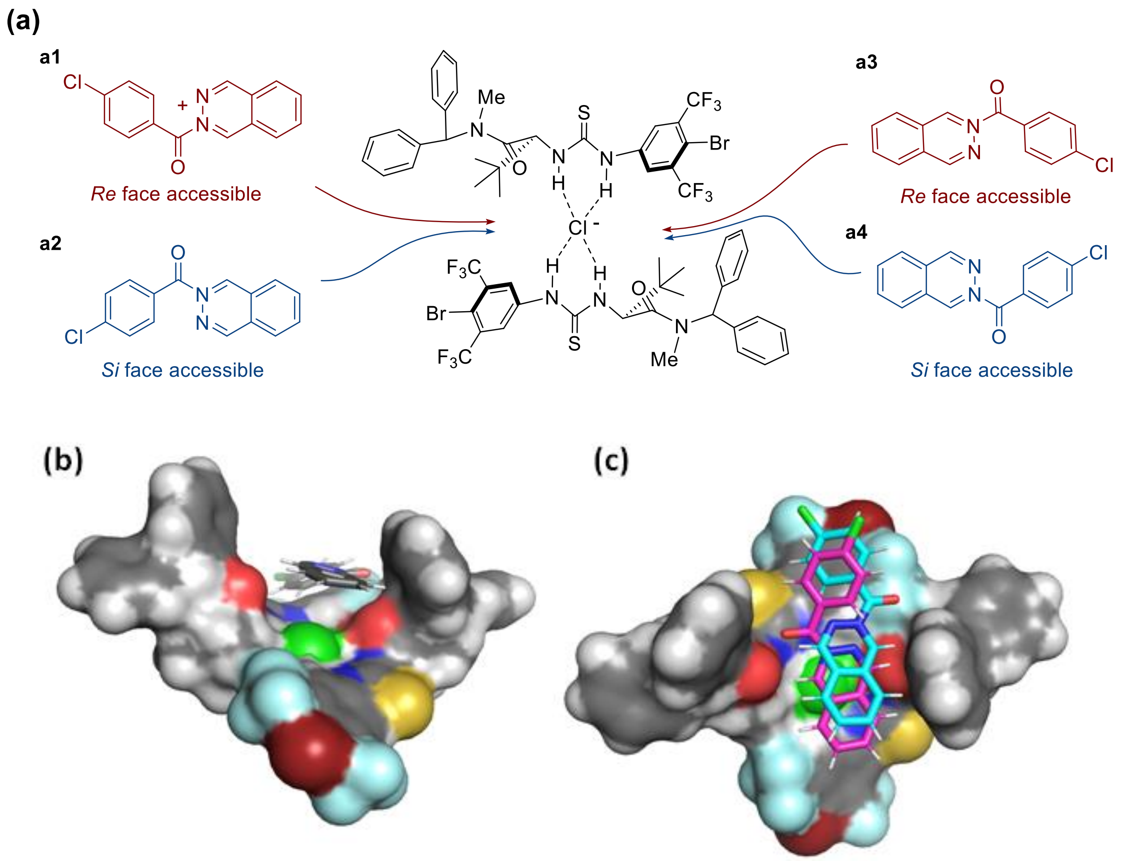

Figure S21. Insertion of the phthalazinium ion into the groove of the anionic complex. **(a)** The four possible approaches of the phthalazinium ion. Due to the C_2-symmetry, **a1** and **a3**, and **a2** and **a4** are the same. **(b)** View of the complex showing the *Si* face inserted in the groove. **(c)** Overlap of the two insertion modes of the phthalazinium cation showing *Re* (magenta) and *Si* (cyan) faces.

20.3. DFT Studies.

20.3.1. Achiral reaction.

First, we examined the addition reaction of the phosphite to the *N*-acylated phthalazine. The uncatalyzed reaction exhibited an energy barrier of 27 kcal/mol, ruling out any unwanted background reactions. In this reaction, the IRC analysis reveals an asynchronous process with a hidden intermediate (carbocation/imine), as presumed by the shoulder after the transition state (TS) (**Figure S22**).

If we consider the presence of the hidden intermediate **B** as the carbocation, the energy profile indicated by the IRC corresponds to a pseudo-S_N1 reaction, and it cannot be considered that there exists an equilibrium between **A** and **B** since the rate-limiting step is the formation of carbocation **B**, which as soon as it forms, reacts practically barrierless to give the reaction product **C**. This is because the calculation is performed in the gas phase or in the presence of a continuous solvent model that does not consider the presence of solvent molecules explicitly, unlike molecular dynamics. Under these conditions, **B** is never located as a minimum since any attempt at optimization collapses the charges, resulting in **A**.

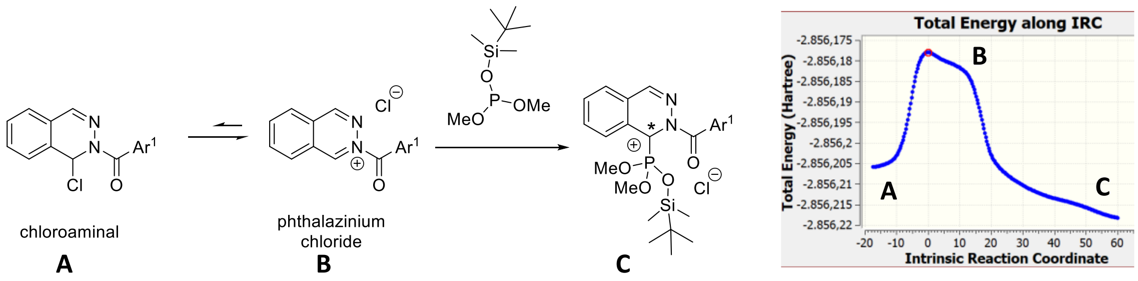

Figure S22. *Left:* Uncatalyzed reaction. *Right*: Intrinsic reaction coordinate (IRC) for the reaction in gas phase.

20.3.2. Catalyzed reaction.

On the contrary, the situation in the presence of the catalyst is completely different, whether in the gas phase or within a continuous solvent model. The stabilization of the carbocation, exerted by the two catalyst molecules through the abstraction of the chloride ion, makes the ion pair **SC** much more stable than chloroaminal-type **A**, as the former forms without a barrier, with the second step being the rate-limiting one, as mentioned in the main text.

We optimized the two starting complexes obtained from MD simulations showing the *Re* (**SC-*Re***) and *Si* (**SC-*Si***) faces, with the thioureas in an antiparallel disposition. For comparison, we also optimized the structure of a very minor starting complex in which the thioureas are oriented in a parallel way, forcing the phthalazinium to stand more on edge but still preferably showing the *Si* face (**Figure S23**).

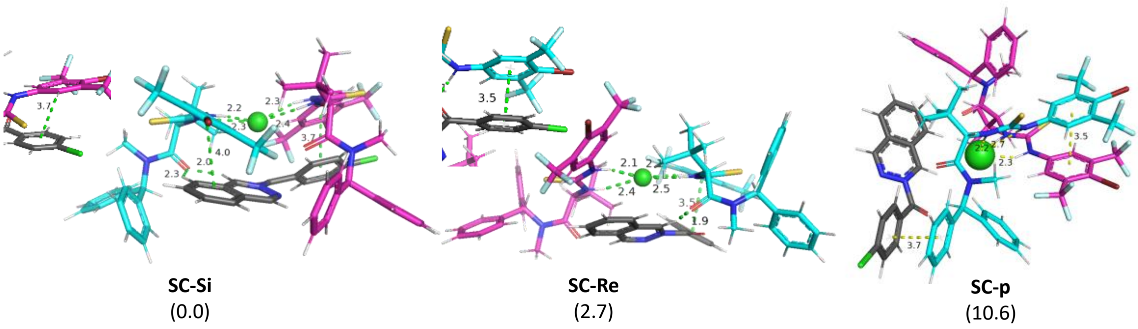

Figure S23. Optimized (wb97xd/6-31G(d,p)) starting complexes showing *Si* (**SC-*Si***) and *Re* (**SC-*Re***) faces and the very minor complex **SC-p** with the ligand on edge. Thioureas are colored in cyan and magenta for clarity. For **SC-*Si*** and **SC-*Re***, details of π–π interactions are given. Relative energy (wb97xd/def2tzvp/smd=THF) values are given in kcal/mol in brackets.

Both **SC-*Si*** and **SC-*Re*** exhibit π–π interactions between the 4-chlorophenyl ring of the substrate and the 4-bromo-3,5-bistrifluoromethylphenyl ring of one of the thioureas (*see* **Figure S23**). Additionally, a similar binding of the thiourea moieties with the chloride anion is observed in both complexes. In **SC-*Si***, more hydrogen bond interactions between the carbonyl group of the catalyst and phthalazine protons are observed than for **SC-*Re***. However, the most significant difference between both complexes is the π–π stacking between the benzene ring of the phthalazine and the 4-bromo-bis-trifluoromethylphenyl ring of the thiourea which is not involved in the first stacking observed in **SC-*Si***. This additional interaction grants **SC-*Si*** greater stability. Nevertheless, the difference of less than three kcal/mol between both complexes prevents us from confidently determining that the more stable complex is the most reactive. According to the Curtin-Hammett principle, it is necessary to analyze the two corresponding transition states to determine which one presents a lower activation barrier.

Therefore, we located the corresponding transition structures **TS-*Si*** and **TS-*Rec*** by approaching the *tert*-butyldimethylsilyl dimethyl phosphite by the accessible face. To explore the conformational potential energy surface of the transition structures, we considered three staggered approaches for the nucleophile (**Figure S24**, a and b). In both cases, the three staggered approaches converged to only one structure for each approach. Additionally, the conformational preferences for the rest of the molecule were assumed to be those found using MD simulations of the precursor complexes. Nevertheless, rotation of the diphenylmethyl groups of the thioureas was also considered, and no different results from those observed in MD were found.

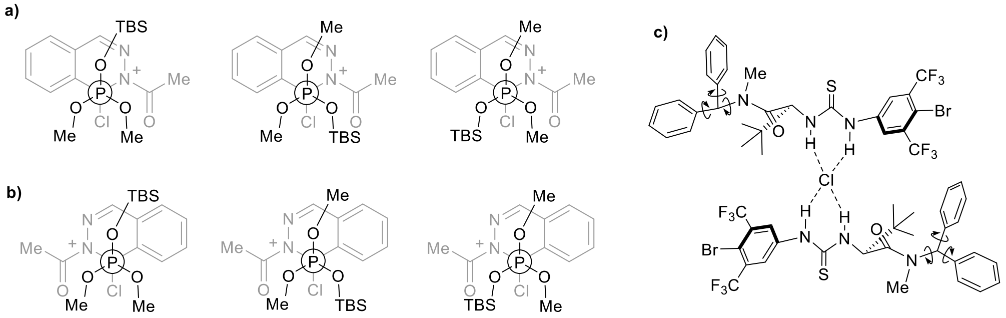

Figure S24. Starting approaches for exploring the conformational PES of the transition structures coming from **SC-*Si*** **(a)** and **SC-*Re*** **(b)** complexes; the structure of the ionic pair formed by the reagent and the two thioureas was taken from the previously optimized structure. **(c)** Additional exploration of the potential energy surface (PES).

As a result, we found two transition structures (**TS-*Si*** and **TS-*Re***). In both cases, practically the same interactions found in the starting complexes are maintained, particularly those of the π−π stacking type. The intermolecular interactions were corroborated with an NCI analysis, as indicated in the main text.

20.3.3. Energies.

We optimized the structures and calculated the energies at three different levels of theory in order to verify which level best described the experimental results:

Level 1: m062x/6-311+G(d,p)/SMD=THF//m062x/6-31G(d,p)
Level 2: wb97xd/def2tzvp/SMD=THF//wb97xd/def2svp
Level 3: wb97xd/6-311+G(d,p)/SMD=THF//wb97xd/6-31G(d,p)

Table S8. Absolute (hartree) and relative (kcal/mol) energies corresponding to the uncatalyzed reaction calculated at level 1.

	E_0	ΔE_0[a]	G	ΔG[b]	im. freq
**RE**	-1682.426412		-1682.484241		
**PHOS**	-1173.747782		-1173.809382		
**TS**	-2856.165880	5.2	-2856.261547	20.1	-93.5

[a]Referred to the sum of the reagents (-2856.174194). [b]Referred to the sum of the reagents (-2856.29362299).

Table S9. Absolute (hartree) and relative (kcal/mol) energies corresponding to the catalyzed reaction calculated at level 1.

	E_0	ΔE_0	G	ΔG	im. freq
**TH**	-4932.201606		-4932.304150		
**TH-dimer**	-9864.436338	-20.8[a]	-9864.599930	5.3[b]	
**PHTA**	-1682.426412		-1682.484241		
**PHOS**	-1173.747782		-1173.809382		
**SC-Re**	-11546.889545	-37.6[c]	-11547.084496	5.0[d]	
**SC-Si**	-11546.892895	-39.7[c]	-11547.090967	1.0[d]	
**TS-Re**	-12720.634491	-35.8[e]	-12720.863133	24.3[f]	-106.9
**TS-Si**	-12720.651491	-46.5[e]	-12720.814504	13.5[f]	-75.4

[a]Referred to two molecules of catalyst **TH** (-9864.403211148). [b]Referred to two molecules of catalyst **TH** (-9864.60829985). [c]Referred to two molecules of catalyst **TH** and phthalazine **PHTA** (-11546.82962380). [d]Referred to two molecules of catalyst **TH** phthalazine **PHTA** (-11547.09254094). [e]Referred to two molecules of catalyst **TH**, phthalazine **PHTA** and phosphite **PHOS** (-12720.57740580). [f]Referred to two molecules of catalyst **TH**, phthalazine **PHTA** and phosphite **PHOS** (-12720.901992284).

Table S10. Absolute (hartree) and relative (kcal/mol) energies corresponding to the uncatalyzed reaction calculated at level 2.

	E_0	ΔE_0[a]	G	ΔG[b]	im. freq
**RE**	-1682.572075		-1682.629904		
**PHOS**	-1173.946374		-1174.007974		
**TS**	-2856.509383	5.7	-2856.605051	20.6	-93.5

[a]Referred to the sum of the reagents (-2856.518450). [b]Referred to the sum of the reagents (-2856.63787836).

Table S11. Absolute (hartree) and relative (kcal/mol) energies corresponding to the catalyzed reaction calculated at level 2.

	E_0	ΔE_0	G	ΔG	im. freq
**TH**	-4932.618102		-4932.722200		
**TH-dimer**	-9865.277009	-25.6[a]	-9865.444408	0.0[b]	
**PHTA**	-1682.572075		-1682.629904		
**PHOS**	-1173.946374		-1174.007974		
**SC-Re**	-11547.863646	-34.7[c]	-11548.068683	3.5[d]	
**SC-Si**	-11547.867782	-37.3[c]	-11548.068897	3.4[d]	
**TS-Re**	-12721.813176	-36.7[e]	-12722.046607	22.4[f]	-138.5
**TS-Si**	-12721.826525	-45.1[e]	-12722.057704	15.4[f]	-106.9

[a]Referred to two molecules of catalyst **TH** (-9865.23620340). [b]Referred to two molecules of catalyst **TH** (-9865.44440084). [c]Referred to two molecules of catalyst **TH** and phthalazine **PHTA** (-11547.80827864). [d]Referred to two molecules of catalyst **TH** phthalazine **PHTA** (-11548.07430485). [e]Referred to two molecules of catalyst **TH**, phthalazine **PHTA** and phosphite **PHOS** (-12721.75465309). [f]Referred to two molecules of catalyst **TH**, phthalazine **PHTA** and phosphite **PHOS** (-12721.08337920).

Table S12. Absolute (hartree) and relative (kcal/mol) energies corresponding to the uncatalyzed reaction calculated at level 3.

	E_0	ΔE_0[a]	G	ΔG[b]	im. freq
**RE**	-1682.483452		-1682.541281		
**PHOS**	-1173.857625		-1173.919225		
**TS**	-2856.335715	3.4	-2856.431383	18.3	-93.5

[a]Referred to the sum of the reagents (-2856.341077). [b]Referred to the sum of the reagents (-2856.46050567).

Table S13. Absolute (hartree) and relative (kcal/mol) energies corresponding to the catalyzed reaction calculated at level 3.

	E_0	ΔE_0	G	ΔG	im. freq
**TH**	-4932.346938		-4932.447585		
**TH-dimer**	-9864.742182	-30.3[a]	-9864.906577	-7.2[b]	
**PHTA**	-1682.483452		-1682.541281		
**PHOS**	-1173.857625		-1173.919225		
**SC-Re**	-11547.247288	-43.9[c]	-11547.453081	-10.4[d]	
**SC-Si**	-11547.256517	-49.7[c]	-11547.460051	-14.8[d]	
**TS-Re**	-12721.111627	-48.1[e]	-12721.346830	5.6[f]	-137.6
**TS-Si**	-12721.123399	-55.5[e]	-12721.349691	3.8[f]	-96.1

[a]Referred to two molecules of catalyst **TH** (-9864.69387586). [b]Referred to two molecules of catalyst **TH** (-9864.89516997). [c]Referred to two molecules of catalyst **TH** and phthalazine **PHTA** (-11547.17732798). [d]Referred to two molecules of catalyst **TH** phthalazine **PHTA** (-11547.43645085). [e]Referred to two molecules of catalyst **TH**, phthalazine **PHTA** and phosphite **PHOS** (-12721.03495286). [f]Referred to two molecules of catalyst **TH**, phthalazine **PHTA** and phosphite **PHOS** (-12721.35567563).

20.4. Cartesian coordinates.

catalyst-dimer

0 1

C	0	-5.1026939228	2.7701901612	6.1600887748
C	0	-4.2390393331	1.6777682485	6.0924335341
C	0	-3.9122777388	1.1204870762	4.8577386335
H	0	-3.2166383795	0.2785192738	4.8039696490
H	0	-3.8088399450	1.2603725858	7.0052253508
H	0	-5.3550005089	3.2121528282	7.1261780595
C	0	-5.6348610311	3.3013710538	4.9860974340
H	0	-6.3065154425	4.1614938172	5.0293312180
C	0	-5.3027717277	2.7458475021	3.7521347692
H	0	-5.7016881621	3.1976809577	2.8417288675
C	0	-4.4398400708	1.6489258983	3.6755096841
C	0	-4.0244517971	1.0189968393	2.3596063782
H	0	-3.0536075432	0.5360476242	2.5246836864
C	0	-4.9290052518	-0.1183405704	1.9080570575
C	0	-6.3173114043	-0.0907161002	2.0697266703
C	0	-7.1028235653	-1.1492340110	1.6183760744
C	0	-6.5058961415	-2.2591196030	1.0225321868
C	0	-5.1202255009	-2.3085333649	0.8877455648
C	0	-4.3375755335	-1.2403118570	1.3194912890
H	0	-3.2489587602	-1.2867646593	1.2221134333
H	0	-4.6419276934	-3.1861184789	0.4521253371
H	0	-7.1184939813	-3.0913289139	0.6706646781
H	0	-8.1869077539	-1.1106921873	1.7443142553
H	0	-6.7913523286	0.7606697118	2.5640223255
N	0	-3.8239985515	2.0182045020	1.2857823427
C	0	-4.8984751293	2.2550934220	0.3333962764
H	0	-4.7174899573	3.2091509942	-0.1732453945

H	0	-4.9577490505	1.4479842512	-0.4129556510
H	0	-5.8637471837	2.2946076421	0.8514186810
C	0	-2.6508428016	2.6610376636	1.0951872394
O	0	-2.4559156017	3.3598342746	0.0899741681
C	0	-1.5135277531	2.4788940351	2.0984288943
H	0	-1.7982585038	1.7871459432	2.8941101048
C	0	-1.0690463895	3.7892756282	2.8204400650
C	0	-2.2807045529	4.4048026425	3.5289411494
H	0	-2.7035849878	3.7190700931	4.2774578154
H	0	-1.9809223494	5.3276561173	4.0481368673
H	0	-3.0791978863	4.6705340549	2.8185072568
C	0	-0.0190390752	3.3937742625	3.8688486730
H	0	-0.4112214691	2.6411974390	4.5696375740
H	0	0.8823900986	2.9750159757	3.3968486392
H	0	0.2866575952	4.2782505814	4.4483713453
C	0	-0.4589873864	4.8136598394	1.8601709744
H	0	0.3978610646	4.3941691807	1.3122075026
H	0	-1.1958003693	5.1679033509	1.1281065270
H	0	-0.0884986534	5.6806780504	2.4284713058
N	0	-0.4272156065	1.8415511226	1.3879125916
H	0	0.0522338031	2.3905585945	0.6720941438
C	0	0.1276434021	0.6540866239	1.7319864639
S	0	-0.5246314183	-0.4068827643	2.8588097371
N	0	1.2935700092	0.4199872276	1.0622968712
H	0	1.6146418299	1.1920643131	0.4681069845
C	0	2.2782009383	-0.5269715005	1.3976750922
C	0	2.0391305660	-1.8974605875	1.4025821041
H	0	1.0441322576	-2.2690775167	1.1733553649
C	0	3.5568064417	-0.0619135366	1.6858872338
H	0	3.7419255150	1.0119260436	1.7018577663
C	0	4.6054618821	-0.9395420107	1.9471780742
C	0	5.9819191078	-0.3407925107	2.1727141696
F	0	6.8538350705	-0.7477406808	1.2442977770
F	0	5.9428758687	0.9955663202	2.1005381779
F	0	6.4836311402	-0.6504835055	3.3658832162
C	0	4.3628201757	-2.3189640530	1.9545897492
Br	0	5.7753871184	-3.5295823751	2.2891138185
C	0	3.0677837666	-2.7914349231	1.6848616260
C	0	2.7342686798	-4.2740077887	1.6527535373
F	0	3.4553973671	-4.9286384447	0.7365101235
F	0	2.9565104702	-4.8551853173	2.8317390839
F	0	1.4514362386	-4.4749835331	1.3493298193
C	0	3.1517892563	-3.0256015385	-1.9226613672
C	0	4.3546620778	-2.5132349715	-1.4382321953
C	0	4.5600245538	-1.1368370077	-1.3797601105
H	0	5.5036859869	-0.7546672557	-0.9862634524
H	0	5.1368407395	-3.1899371794	-1.0877312167
H	0	2.9906564333	-4.1043446651	-1.9608142672
C	0	2.1558044183	-2.1490475550	-2.3440667388
H	0	1.2090743349	-2.5350190393	-2.7261947865
C	0	2.3563661302	-0.7710228596	-2.2757511986
H	0	1.5579725675	-0.1056723591	-2.6128108757
C	0	3.5624882510	-0.2483274955	-1.7982329578

C	0	3.7401683736	1.2559568733	-1.6190049730
H	0	3.4520440153	1.4872768830	-0.5872864924
C	0	5.1507356049	1.8009505904	-1.7853996336
C	0	5.5499619457	2.8619030496	-0.9655983218
C	0	6.8076224099	3.4426940241	-1.1108448511
C	0	7.6854664728	2.9664337072	-2.0832010988
C	0	7.2990315864	1.9070905430	-2.9031466799
C	0	6.0402453975	1.3276624801	-2.7546206977
H	0	5.7545865308	0.4859589591	-3.3897862887
H	0	7.9851695535	1.5245223477	-3.6617597505
H	0	8.6738008129	3.4165524760	-2.1977208707
H	0	7.1059152399	4.2644963590	-0.4565502897
H	0	4.8628245362	3.2349734653	-0.2008431211
N	0	2.8017974178	2.0198589382	-2.4551838799
C	0	3.0443889747	1.9789935654	-3.8908449667
H	0	2.0999066327	1.9570791229	-4.4458826332
H	0	3.6760195051	2.8168513747	-4.2194662392
H	0	3.5622205334	1.0406510061	-4.1233065147
C	0	1.7836320957	2.6770948329	-1.8666115516
O	0	1.6022392311	2.6486455491	-0.6396360421
C	0	0.7813935149	3.4667781815	-2.7244964965
H	0	0.8661462520	3.1562838494	-3.7693327030
C	0	1.0052558445	5.0051084517	-2.7000084833
C	0	0.8127501200	5.6074407458	-1.3054203192
H	0	-0.2028110401	5.4350652511	-0.9201425548
H	0	1.5264371464	5.1937801071	-0.5800444530
H	0	0.9601394754	6.6972947953	-1.3497690311
C	0	2.4304860496	5.2997257130	-3.1872097649
H	0	2.6019465557	6.3861336997	-3.2140949607
H	0	3.1947351308	4.8610586851	-2.5258875033
H	0	2.5932287029	4.9139448457	-4.2052477254
C	0	-0.0028820497	5.6327818148	-3.6727162192
H	0	-1.0372957360	5.4421306826	-3.3529298207
H	0	0.1447615685	6.7221753792	-3.7248421903
H	0	0.1163899710	5.2239027758	-4.6880398159
N	0	-0.5475746652	3.0829157589	-2.3046888278
H	0	-0.9028773692	3.4738294018	-1.4315106963
C	0	-1.1154455624	1.9230273894	-2.7209179198
S	0	-0.5051040264	0.9708684724	-3.9638299174
N	0	-2.2589062148	1.5978008125	-2.0596763576
H	0	-2.5970404613	2.2632082605	-1.3541303656
C	0	-2.8017202713	0.2945530633	-2.0750715940
C	0	-4.0812470236	0.0616781661	-2.5569845443
H	0	-4.6686759603	0.8978877189	-2.9337233571
C	0	-2.0541677403	-0.7759858957	-1.5878314084
H	0	-1.0613509548	-0.5889293756	-1.1793483121
C	0	-2.5483178315	-2.0735121874	-1.6377660489
C	0	-1.6651318685	-3.1826504775	-1.0925467760
F	0	-2.2766965289	-3.8848213063	-0.1367110436
F	0	-1.2821362195	-4.0315688164	-2.0454984270
F	0	-0.5523921536	-2.6799185465	-0.5473990905
C	0	-3.8242408809	-2.3127600964	-2.1779546964
Br	0	-4.4626692026	-4.0824137896	-2.3588816931

C	0	-4.6085352847	-1.2306401553	-2.5974157579
C	0	-6.0188558709	-1.3995871444	-3.1385621142
F	0	-6.8164264875	-2.0192760967	-2.2687562796
F	0	-6.5759909077	-0.2104052740	-3.3941358405
F	0	-6.0363228523	-2.0921130542	-4.2761756210

catalyst

0 1

C	0	7.2057954755	-1.5108100118	2.1137730894
C	0	7.4109139471	-0.8391572142	0.9093788582
C	0	6.3711670986	-0.1209876171	0.3213244533
H	0	6.5367672668	0.3761220037	-0.6363021752
H	0	8.3854911182	-0.8811118508	0.4182322514
H	0	8.0183016203	-2.0800053301	2.5704150855
C	0	5.9550462295	-1.4580429313	2.7278923358
H	0	5.7817058350	-1.9884142514	3.6666308703
C	0	4.9185577765	-0.7357708073	2.1398007123
H	0	3.9350476323	-0.7043146464	2.6171381054
C	0	5.1155400884	-0.0604429236	0.9313764291
C	0	3.9501385849	0.6968308769	0.3228888609
H	0	3.0321120391	0.1880192446	0.6432884740
C	0	3.7841574955	2.1179959909	0.8510000427
C	0	4.8251412404	2.8333866662	1.4452841146
C	0	4.6220876021	4.1422995879	1.8844949254
C	0	3.3757362030	4.7461920512	1.7366310468
C	0	2.3270874112	4.0324851588	1.1545716147
C	0	2.5304976190	2.7269831642	0.7182885275
H	0	1.7040593509	2.1590938494	0.2814617211
H	0	1.3421965468	4.4926958453	1.0497279844
H	0	3.2178780019	5.7702792086	2.0817088043
H	0	5.4445201307	4.6910817233	2.3484838136
H	0	5.8035493081	2.3642689942	1.5712925946
N	0	3.9573844425	0.6851545645	-1.1474782409
C	0	4.5650765063	1.7915902920	-1.8729834331
H	0	3.8194933113	2.5568040741	-2.1413374315
H	0	5.3327144137	2.2658580656	-1.2513713233
H	0	5.0153951860	1.4152453741	-2.7997746671
C	0	3.2784824068	-0.2211800901	-1.9015751371
O	0	3.1342372034	-0.0720269371	-3.1076667831
C	0	2.6436733266	-1.4530380322	-1.2384695915
H	0	2.7009579764	-1.4126937550	-0.1480002853
C	0	3.2931514912	-2.8042814374	-1.6637812575
C	0	3.0695334076	-3.1215604911	-3.1483191442
H	0	3.5421784160	-4.0845486397	-3.3953102225
H	0	1.9974249719	-3.2229072617	-3.3815376579
H	0	3.4901919800	-2.3428501735	-3.7975765040
C	0	4.7963596321	-2.7443123108	-1.3706846184
H	0	5.2998507186	-1.9702960228	-1.9702331761
H	0	4.9931015064	-2.5339930207	-0.3088316452
H	0	5.2638685991	-3.7101934916	-1.6149318997
C	0	2.6531967934	-3.9044737678	-0.8055775338

H	0	1.5651879792	-3.9496153519	-0.9633032868
H	0	3.0746571978	-4.8871924254	-1.0669853844
H	0	2.8318852175	-3.7311496595	0.2667645033
N	0	1.2330944820	-1.4005817176	-1.5754446184
H	0	1.0670167266	-1.3264116351	-2.5755371932
C	0	0.2432096199	-1.0431399504	-0.7270121305
S	0	0.4665017124	-0.8028493306	0.9151582656
N	0	-0.9657119483	-0.8954727217	-1.3633732328
H	0	-0.9421405861	-0.9361275309	-2.3761355499
C	0	-2.2033520671	-0.4900777033	-0.8356464230
C	0	-3.0048764104	0.3443146982	-1.6113247012
H	0	-2.6441919960	0.6949778892	-2.5788961639
C	0	-2.6857414276	-0.9260645666	0.3982436388
H	0	-2.0802219050	-1.5890907142	1.0100948178
C	0	-3.9314127523	-0.5099023472	0.8594182560
C	0	-4.3794101398	-1.0254448911	2.2196874244
F	0	-3.4618527358	-1.8340787554	2.7540174735
F	0	-4.5754255883	-0.0302240278	3.0832946177
F	0	-5.5115616476	-1.7245950797	2.1326387724
C	0	-4.7329299414	0.3400270466	0.0796275662
Br	0	-6.4161882885	0.9214878950	0.7076881734
C	0	-4.2622536332	0.7546212704	-1.1746603532
C	0	-5.0639282363	1.6648055906	-2.0918469527
F	0	-4.4039797457	1.8934886079	-3.2341682066
F	0	-6.2365221515	1.1278047908	-2.4236239783
F	0	-5.2953116456	2.8512148997	-1.5335213219

SC-Re

0 1

C	0	4.4965120000	5.5540420000	2.1170940000
C	0	4.0303520000	5.2803830000	0.8358260000
C	0	2.6640140000	5.3096550000	0.5627830000
H	0	2.3210360000	5.0733760000	-0.4401620000
H	0	4.7257920000	5.0264290000	0.0422350000
H	0	5.5612700000	5.5381870000	2.3279510000
C	0	3.5841230000	5.8466840000	3.1304980000
H	0	3.9350740000	6.0563900000	4.1361510000
C	0	2.2206470000	5.8777500000	2.8558480000
H	0	1.5172930000	6.1204930000	3.6477740000
C	0	1.7446860000	5.6223340000	1.5643610000
C	0	0.2419230000	5.6161530000	1.3047670000
H	0	-0.1612660000	4.7279540000	1.7977450000
C	0	-0.5294170000	6.8187550000	1.8323220000
C	0	0.0461720000	8.0736590000	2.0234680000
C	0	-0.7318550000	9.1504000000	2.4426790000
C	0	-2.0932480000	8.9838940000	2.6709030000
C	0	-2.6775190000	7.7337960000	2.4789100000
C	0	-1.8984810000	6.6611430000	2.0635310000
H	0	-2.3538220000	5.6858180000	1.9084400000
H	0	-3.7390700000	7.5942330000	2.6558610000
H	0	-2.6982850000	9.8234450000	2.9976590000

H	0	-0.2701680000	10.1217990000	2.5878840000
H	0	1.1088180000	8.2125460000	1.8491980000
N	0	-0.0742380000	5.4154180000	-0.1114850000
C	0	0.0960770000	6.5528400000	-1.0091750000
H	0	0.8071330000	7.2452460000	-0.5567320000
H	0	0.5224530000	6.2308260000	-1.9596240000
H	0	-0.8447490000	7.0857880000	-1.1706050000
C	0	-0.4640850000	4.1710930000	-0.4921280000
O	0	-0.5133800000	3.2351390000	0.3096700000
C	0	-0.8342190000	3.9055420000	-1.9628600000
H	0	-0.4403630000	4.6816360000	-2.6157700000
C	0	-2.3685280000	3.8458990000	-2.2072740000
C	0	-2.9341210000	5.2618360000	-2.0195980000
H	0	-2.4821570000	5.9698990000	-2.7224010000
H	0	-4.0135180000	5.2569080000	-2.1975580000
H	0	-2.7687770000	5.6331640000	-1.0015720000
C	0	-2.6089670000	3.3867770000	-3.6516180000
H	0	-2.0759890000	4.0253390000	-4.3638220000
H	0	-2.2732020000	2.3576960000	-3.8016840000
H	0	-3.6776530000	3.4297100000	-3.8810310000
C	0	-3.0855580000	2.9017230000	-1.2397160000
H	0	-4.1521460000	2.8727030000	-1.4724160000
H	0	-2.7049780000	1.8773350000	-1.3021680000
H	0	-2.9657030000	3.2464520000	-0.2099110000
N	0	-0.1545170000	2.6791730000	-2.3297680000
H	0	-0.5365890000	1.8175540000	-1.9360670000
C	0	1.1513260000	2.6653010000	-2.6700660000
S	0	1.9915800000	4.0624210000	-3.1148980000
N	0	1.6830680000	1.4005160000	-2.6568640000
H	0	1.0016180000	0.6568040000	-2.4754520000
C	0	3.0095970000	0.9820740000	-2.5550350000
C	0	4.0931310000	1.8357700000	-2.3419550000
H	0	3.9351240000	2.9028490000	-2.3434000000
C	0	3.2454990000	-0.3968440000	-2.5825820000
H	0	2.4179640000	-1.0819610000	-2.7341310000
C	0	4.5166930000	-0.9092290000	-2.3504410000
C	0	4.7000880000	-2.4104920000	-2.3306690000
F	0	4.9526300000	-2.8507670000	-1.0766390000
F	0	3.6090980000	-3.0522410000	-2.7607370000
F	0	5.7156460000	-2.8110560000	-3.1038600000
C	0	5.5817220000	-0.0481600000	-2.0751270000
Br	0	7.2580060000	-0.7317750000	-1.5357840000
C	0	5.3603140000	1.3291910000	-2.0969280000
C	0	6.4594640000	2.3217420000	-1.7801790000
F	0	6.8840890000	2.1960760000	-0.5036120000
F	0	7.5243040000	2.1727170000	-2.5732890000
F	0	6.0378000000	3.5888460000	-1.9157630000
C	0	2.4786520000	-0.7808330000	4.6963620000
C	0	1.4066340000	-1.5748520000	4.3087590000
C	0	1.5887690000	-2.5977950000	3.3771120000
H	0	0.7329990000	-3.1787530000	3.0549170000
H	0	0.4183200000	-1.3981770000	4.7223240000
H	0	2.3359780000	0.0186430000	5.4164780000

C	0	3.7413350000	-1.0211800000	4.1557960000
H	0	4.5849470000	-0.4030860000	4.4459180000
C	0	3.9209510000	-2.0438490000	3.2337960000
H	0	4.9045450000	-2.2215630000	2.8073030000
C	0	2.8442910000	-2.8369630000	2.8248440000
C	0	3.0818500000	-3.8887550000	1.7506130000
H	0	3.5185060000	-3.3712000000	0.8932760000
C	0	4.0613330000	-4.9838230000	2.1524270000
C	0	4.2961860000	-5.3310410000	3.4820640000
C	0	5.1737830000	-6.3654570000	3.7964530000
C	0	5.8253070000	-7.0611320000	2.7837820000
C	0	5.5983100000	-6.7175330000	1.4529880000
C	0	4.7234110000	-5.6841010000	1.1413700000
H	0	4.5544980000	-5.4085560000	0.1045470000
H	0	6.1091170000	-7.2497070000	0.6569680000
H	0	6.5124790000	-7.8642700000	3.0296880000
H	0	5.3490660000	-6.6238420000	4.8358410000
H	0	3.7970210000	-4.7860030000	4.2777370000
N	0	1.8409630000	-4.4788330000	1.2315690000
C	0	1.2216230000	-5.5810590000	1.9648920000
H	0	1.4202310000	-6.5486540000	1.4943220000
H	0	1.6438290000	-5.6060040000	2.9688690000
H	0	0.1436190000	-5.4322450000	2.0536790000
C	0	1.3202350000	-3.9527690000	0.0971760000
O	0	1.7723130000	-2.9184360000	-0.4112530000
C	0	0.1720780000	-4.6883570000	-0.5925150000
H	0	-0.2688740000	-5.4369920000	0.0626470000
C	0	0.6581070000	-5.4186320000	-1.8933940000
C	0	2.0353490000	-6.0540660000	-1.6537510000
H	0	2.0466450000	-6.6870620000	-0.7592340000
H	0	2.3049410000	-6.6846180000	-2.5063380000
H	0	2.8101910000	-5.2885940000	-1.5513170000
C	0	-0.3652530000	-6.5132380000	-2.2218510000
H	0	-0.3959280000	-7.2789690000	-1.4401350000
H	0	-1.3714490000	-6.0952240000	-2.3162410000
H	0	-0.1062680000	-6.9987870000	-3.1685410000
C	0	0.7457640000	-4.4388900000	-3.0713620000
H	0	1.2084960000	-4.9336990000	-3.9309430000
H	0	-0.2481680000	-4.0999280000	-3.3756060000
H	0	1.3454620000	-3.5642870000	-2.8153270000
N	0	-0.8624820000	-3.7216320000	-0.8709500000
H	0	-0.5412340000	-2.8468870000	-1.2830590000
C	0	-2.1592510000	-3.8165060000	-0.4999280000
S	0	-2.7827420000	-5.1272960000	0.3551390000
N	0	-2.8606410000	-2.7170200000	-0.9106220000
H	0	-2.2821380000	-1.9809550000	-1.3220680000
C	0	-4.2090750000	-2.3705300000	-0.8677530000
C	0	-5.2258000000	-3.0957050000	-0.2494190000
H	0	-4.9909890000	-4.0288790000	0.2407820000
C	0	-4.5363070000	-1.1683140000	-1.5041540000
H	0	-3.7562410000	-0.6002210000	-1.9996520000
C	0	-5.8361810000	-0.6907930000	-1.5166640000
C	0	-6.1091690000	0.6010370000	-2.2552280000

F	0	-6.4667850000	1.5900970000	-1.4171130000
F	0	-5.0164000000	1.0253880000	-2.9152790000
F	0	-7.0796510000	0.4702650000	-3.1672950000
C	0	-6.8477690000	-1.3990320000	-0.8598210000
Br	0	-8.6044170000	-0.7166320000	-0.7982800000
C	0	-6.5279160000	-2.6056090000	-0.2362480000
C	0	-7.5657000000	-3.3966890000	0.5316180000
F	0	-8.6098710000	-3.7456170000	-0.2296890000
F	0	-7.0514220000	-4.5264400000	1.0378680000
F	0	-8.0398250000	-2.6851760000	1.5733770000
Cl	0	-0.7378090000	-0.4409230000	-1.6195360000
N	0	-0.3114150000	1.0332890000	2.4964660000
C	0	0.7350890000	1.7981740000	2.5713610000
H	0	0.6040800000	2.7024710000	3.1568500000
C	0	2.0021300000	1.4978610000	1.9871810000
C	0	3.1455060000	2.2919390000	2.1538520000
H	0	3.0824950000	3.2339890000	2.6879510000
C	0	4.3422970000	1.8543550000	1.6249640000
H	0	5.2296230000	2.4675830000	1.7366640000
C	0	4.4396940000	0.6267990000	0.9404330000
H	0	5.4017150000	0.2927200000	0.5710680000
C	0	3.3213340000	-0.1456180000	0.7307260000
H	0	3.3589780000	-1.0919190000	0.2044340000
C	0	2.0903630000	0.2952890000	1.2528590000
C	0	0.9216050000	-0.4730070000	1.1335400000
H	0	0.8913480000	-1.3976010000	0.5651900000
N	0	-0.1811950000	-0.0858490000	1.7476310000
C	0	-1.3297360000	-1.0214130000	1.7398770000
O	0	-1.0507340000	-2.1906710000	1.7091000000
C	0	-2.6828910000	-0.4655180000	1.8032930000
C	0	-3.0010470000	0.8374320000	1.4063250000
H	0	-2.2311130000	1.5117680000	1.0478100000
C	0	-4.3205130000	1.2605070000	1.4497050000
H	0	-4.5900860000	2.2587300000	1.1282820000
C	0	-5.3060860000	0.3894410000	1.9075700000
Cl	0	-6.9501420000	0.9414010000	2.0013910000
C	0	-5.0060630000	-0.9170190000	2.2837340000
H	0	-5.7969370000	-1.5888460000	2.5936580000
C	0	-3.6912050000	-1.3457650000	2.2152490000
H	0	-3.4358010000	-2.3716340000	2.4603870000

SC-Si

0 1

C	0	7.0001948352	-2.3199244423	-3.1595090469
C	0	6.1795156120	-3.1141673508	-3.9624649856
C	0	5.3170230652	-4.0389788358	-3.3809645261
H	0	4.6774028538	-4.6590327982	-4.0152717272
H	0	6.2105017409	-3.0091484621	-5.0492467695
H	0	7.6791517160	-1.5951997199	-3.6143210933
C	0	6.9447680026	-2.4592034136	-1.7751856811
H	0	7.5785521014	-1.8438151120	-1.1320206872

C	0	6.0759643731	-3.3848100126	-1.1945700299
H	0	6.0178383726	-3.4515641060	-0.1079733387
C	0	5.2561066397	-4.1862474078	-1.9901457115
C	0	4.2949254356	-5.2080908141	-1.3997132394
H	0	3.3437895017	-5.0733997933	-1.9331667957
C	0	4.7182341026	-6.6576975862	-1.5917137501
C	0	6.0291263402	-7.0286770059	-1.8978954809
C	0	6.3701773815	-8.3754447686	-2.0319794269
C	0	5.4047587236	-9.3643423406	-1.8612212413
C	0	4.0912636337	-9.0022933323	-1.5575481345
C	0	3.7534734603	-7.6587756692	-1.4262449797
H	0	2.7225364867	-7.3765618604	-1.1931836271
H	0	3.3261962261	-9.7710251714	-1.4288381250
H	0	5.6715709207	-10.4177725399	-1.9692209410
H	0	7.3998723406	-8.6501170052	-2.2709846397
H	0	6.7914585463	-6.2585183601	-2.0353714862
N	0	3.9789668838	-4.9457424424	0.0105055559
C	0	4.8175883122	-5.5165029210	1.0546322611
H	0	5.0524677014	-4.7558750698	1.8113322408
H	0	4.3531115266	-6.3930375851	1.5291567186
H	0	5.7636711026	-5.8441738674	0.6086282765
C	0	2.9992700490	-4.0374186182	0.2371395778
O	0	2.4656346893	-3.4442305038	-0.6975564264
C	0	2.5058594092	-3.7637192851	1.6678120579
H	0	3.2816103009	-4.0287086378	2.3973286370
C	0	1.2270544622	-4.5861162670	2.0220625489
C	0	0.7217599751	-4.1195421425	3.3967048649
H	0	-0.0913750958	-4.7789369286	3.7363522178
H	0	1.5223407102	-4.1489645605	4.1523530063
H	0	0.3228567091	-3.0956183565	3.3580272436
C	0	0.1076510129	-4.4118896203	0.9871730456
H	0	0.3862089088	-4.8042765097	-0.0004329942
H	0	-0.7878438750	-4.9533360358	1.3252603724
H	0	-0.1802330538	-3.3585499099	0.8565032253
C	0	1.6049806585	-6.0707359353	2.1062586435
H	0	2.3604159735	-6.2517227366	2.8871013176
H	0	0.7157196800	-6.6680552386	2.3566720376
H	0	1.9980438089	-6.4486600877	1.1497424530
N	0	2.2614828798	-2.3470872013	1.7935527138
H	0	1.3060369336	-2.0087669258	1.6380847731
C	0	3.2400769240	-1.4262648788	1.8470886322
S	0	4.8713461508	-1.8443177941	1.9067727216
N	0	2.7632445540	-0.1422173214	1.8194024896
H	0	1.7476980123	-0.0620107621	1.6762638596
C	0	3.4654086124	1.0600097938	1.7158599769
C	0	4.7118094546	1.2873349018	2.3042417450
H	0	5.1885017799	0.4933793914	2.8718059462
C	0	2.8663439391	2.1001143600	1.0029148484
H	0	1.8891704285	1.9371293878	0.5437809902
C	0	3.4938281072	3.3360646036	0.8627019762
C	0	2.7780190869	4.4104908838	0.0642959797
F	0	3.5216458948	4.8573194666	-0.9566768260
F	0	1.6467551805	3.9445382178	-0.4631267715

F	0	2.4540412095	5.4630569555	0.8184241086
C	0	4.7544349546	3.5530721832	1.4373733278
Br	0	5.6185559205	5.2268799635	1.2574939360
C	0	5.3574752305	2.5116794652	2.1585568015
C	0	6.7197102307	2.6642179556	2.8165388303
F	0	7.6683264608	2.9449669572	1.9195104396
F	0	6.7242668104	3.6325062097	3.7331436182
F	0	7.0887435185	1.5406634728	3.4337755211
C	0	-5.6171473497	5.2487548131	-4.2817050162
C	0	-5.4739717288	4.0082150323	-3.6600576794
C	0	-4.3242487512	3.7269657738	-2.9280538535
H	0	-4.2139910419	2.7533288218	-2.4487619978
H	0	-6.2578939729	3.2523850599	-3.7413505238
H	0	-6.5177210891	5.4736713456	-4.8571500178
C	0	-4.6038347361	6.1984266042	-4.1665204585
H	0	-4.7068775700	7.1700494451	-4.6544932213
C	0	-3.4552909995	5.9152580908	-3.4268745703
H	0	-2.6667265377	6.6664598440	-3.3420965460
C	0	-3.3073197634	4.6778885029	-2.7938247462
C	0	-2.0961173680	4.3381954555	-1.9350188168
H	0	-1.8403069010	3.2874684133	-2.1404870027
C	0	-0.8613830299	5.1556041404	-2.2828340584
C	0	-0.2048709209	4.9104751840	-3.4949098570
C	0	0.9504018880	5.6102542419	-3.8346625960
C	0	1.4610673624	6.5784803232	-2.9684482240
C	0	0.7965519603	6.8502143803	-1.7760337730
C	0	-0.3573134052	6.1430401160	-1.4382186054
H	0	-0.8568877386	6.3486345118	-0.4904063612
H	0	1.1870204950	7.6045315191	-1.0902200106
H	0	2.3736036858	7.1200661587	-3.2249875395
H	0	1.4529254028	5.4030303182	-4.7824773335
H	0	-0.6146540969	4.1673965387	-4.1864202202
N	0	-2.4188054220	4.3726996735	-0.4992608563
C	0	-3.4474138450	5.2704503130	0.0128229417
H	0	-4.3504150494	4.7202773777	0.3199652338
H	0	-3.0753583900	5.8527555222	0.8647193394
H	0	-3.7276660242	5.9790158152	-0.7728196511
C	0	-1.7321382549	3.5045960475	0.2920022264
O	0	-0.8580364553	2.7753390646	-0.1646056287
C	0	-2.1344721873	3.3620336749	1.7718122743
H	0	-3.1020390300	3.8423629497	1.9523189165
C	0	-1.1171956196	3.9456238963	2.7966455495
C	0	-0.8528286601	5.4218078488	2.4803529943
H	0	-0.3932348324	5.5463745422	1.4879863098
H	0	-1.7756075005	6.0207716945	2.5276165765
H	0	-0.1504993553	5.8437558633	3.2148672250
C	0	-1.7462303444	3.8197302811	4.1899997278
H	0	-1.0646901750	4.2240561006	4.9540946889
H	0	-2.6994081463	4.3668726286	4.2528614929
H	0	-1.9472898840	2.7668794164	4.4356863147
C	0	0.2134049463	3.1896606978	2.7603051319
H	0	0.0878390445	2.1166686984	2.9711220201
H	0	0.6771428024	3.2887817125	1.7744563551

H	0	0.9056056479	3.5998794980	3.5117569570
N	0	-2.3462360615	1.9477611141	1.9792107464
H	0	-1.5520970580	1.3452428520	1.7385427244
C	0	-3.5596403150	1.3581308616	2.0774602279
S	0	-4.9973161131	2.2258538007	2.1523835456
N	0	-3.4547566703	-0.0090054666	2.0914016870
H	0	-2.4915773465	-0.3471639660	1.9889561217
C	0	-4.4027226855	-0.9793696199	1.7536298574
C	0	-5.7750162531	-0.8646197469	1.9756511876
H	0	-6.1654962039	0.0255354724	2.4602421565
C	0	-3.9202053487	-2.1387861589	1.1375304440
H	0	-2.8489512749	-2.2345632166	0.9525787516
C	0	-4.7817968349	-3.1433182635	0.7136895911
C	0	-4.1729262147	-4.3436020493	0.0134727530
F	0	-4.3715284538	-5.4743626370	0.6908614284
F	0	-4.6731877837	-4.5155538717	-1.2165133229
F	0	-2.8527101226	-4.2019308549	-0.1326203323
C	0	-6.1653966360	-3.0109630546	0.9100775162
Br	0	-7.3498968247	-4.3641355136	0.3224354015
C	0	-6.6496668031	-1.8623932128	1.5495825142
C	0	-8.1313633804	-1.6453288722	1.8122923260
F	0	-8.6334713184	-2.5919199840	2.6092216321
F	0	-8.3528004078	-0.4745910276	2.4120898932
F	0	-8.8420704775	-1.6451711324	0.6835493441
Cl	0	-0.3715328401	-0.5963492531	1.2272752845
N	0	-0.2453107601	0.6979900815	-2.4928932954
C	0	0.7447099773	1.5314553694	-2.5696048887
H	0	0.4980425105	2.5371649336	-2.9176330691
C	0	2.0935035747	1.1980367668	-2.2359802992
C	0	3.1704969154	2.0882622279	-2.3863621622
H	0	2.9916346554	3.0970866513	-2.7613949782
C	0	4.4351877642	1.6767496205	-2.0119438853
H	0	5.2708632188	2.3746721177	-2.0942745041
C	0	4.6642050980	0.3775436977	-1.5075449504
H	0	5.6697379735	0.0743504401	-1.2134102671
C	0	3.6282847938	-0.5215904517	-1.3788801229
H	0	3.7938757190	-1.5363303215	-1.0132869629
C	0	2.3234326195	-0.1080782482	-1.7351281895
C	0	1.2038346067	-0.9588214804	-1.6249763421
H	0	1.2895904830	-1.9853140730	-1.2422243940
N	0	0.0180092297	-0.5293534858	-2.0221434401
C	0	-1.1204349202	-1.4905068251	-1.9647676949
O	0	-0.8377161215	-2.6454786763	-2.0629262664
C	0	-2.4832314718	-0.9423572890	-1.8379067010
C	0	-2.7840093597	0.2711174217	-1.2045935123
H	0	-1.9924736521	0.8982444050	-0.7927059966
C	0	-4.1087648572	0.6597739662	-1.0608559419
H	0	-4.3676374703	1.5761973142	-0.5279879171
C	0	-5.1275609869	-0.1530329450	-1.5590326933
Cl	0	-6.7750062524	0.3604043883	-1.3946547451
C	0	-4.8442552623	-1.3691089225	-2.1796831030
H	0	-5.6531512141	-2.0068028256	-2.5364105927
C	0	-3.5191200349	-1.7620091928	-2.3067486057

H	0	-3.2768829419	-2.7222849339	-2.7634841238

TS-Re

0 1

C	0	-8.3390660000	-4.2281540000	-3.6042230000
C	0	-8.5518260000	-2.8533740000	-3.4859510000
C	0	-7.7835910000	-2.1012720000	-2.6036070000
H	0	-7.9505950000	-1.0244800000	-2.5154150000
H	0	-9.3179940000	-2.3633340000	-4.0906330000
H	0	-8.9407950000	-4.8202370000	-4.2969530000
C	0	-7.3493560000	-4.8373990000	-2.8383420000
H	0	-7.1671890000	-5.9102150000	-2.9315050000
C	0	-6.5793010000	-4.0801670000	-1.9533730000
H	0	-5.7915900000	-4.5657310000	-1.3749860000
C	0	-6.7921420000	-2.7074790000	-1.8210280000
C	0	-5.9721410000	-1.8307450000	-0.8924410000
H	0	-5.5866470000	-0.9970350000	-1.4952700000
C	0	-6.7798500000	-1.1467260000	0.2118450000
C	0	-8.0207100000	-1.6157320000	0.6492980000
C	0	-8.7115470000	-0.9530370000	1.6647470000
C	0	-8.1688700000	0.1880110000	2.2511710000
C	0	-6.9248880000	0.6557300000	1.8250740000
C	0	-6.2334350000	-0.0078380000	0.8152200000
H	0	-5.2636960000	0.3693380000	0.4829680000
H	0	-6.4929320000	1.5524690000	2.2754620000
H	0	-8.7138040000	0.7122980000	3.0394660000
H	0	-9.6824380000	-1.3300560000	1.9937250000
H	0	-8.4611070000	-2.5006430000	0.1837100000
N	0	-4.8157560000	-2.5158490000	-0.3100640000
C	0	-5.0215710000	-3.3253450000	0.8809820000
H	0	-4.7386280000	-2.7721040000	1.7869100000
H	0	-6.0769830000	-3.6150400000	0.9431880000
H	0	-4.3960540000	-4.2267520000	0.8288280000
C	0	-3.5252350000	-2.2574600000	-0.6244550000
O	0	-2.6232720000	-2.6285560000	0.1283580000
C	0	-3.1733840000	-1.5618560000	-1.9452150000
H	0	-4.0808610000	-1.2881070000	-2.4926280000
C	0	-2.3655510000	-2.4944080000	-2.9003920000
C	0	-2.0923190000	-1.7067830000	-4.1904880000
H	0	-3.0203720000	-1.2960270000	-4.6174890000
H	0	-1.4020270000	-0.8695210000	-4.0086700000
H	0	-1.6254670000	-2.3628190000	-4.9390220000
C	0	-1.0292000000	-2.9683500000	-2.3131080000
H	0	-0.3776380000	-2.1322160000	-2.0099880000
H	0	-1.1785860000	-3.6188250000	-1.4436130000
H	0	-0.4908160000	-3.5432440000	-3.0771580000
C	0	-3.2335460000	-3.7175940000	-3.2216550000
H	0	-4.2000510000	-3.4263390000	-3.6619450000
H	0	-2.7126560000	-4.3691660000	-3.9390540000
H	0	-3.4387590000	-4.3165110000	-2.3204570000
N	0	-2.4721380000	-0.3248970000	-1.6821340000

H	0	-1.4687090000	-0.3881960000	-1.4981930000
C	0	-2.9710380000	0.9142260000	-1.9218380000
S	0	-4.5636450000	1.2095970000	-2.3601830000
N	0	-2.0118630000	1.8765300000	-1.7877720000
H	0	-1.0527240000	1.5186140000	-1.6772560000
C	0	-2.0620480000	3.2632740000	-1.9106560000
C	0	-3.1951020000	4.0455640000	-1.6902610000
H	0	-4.1417520000	3.5627970000	-1.4651130000
C	0	-0.8513560000	3.9041690000	-2.1896390000
H	0	0.0451210000	3.2992530000	-2.3335850000
C	0	-0.7615190000	5.2895580000	-2.2455200000
C	0	0.6141950000	5.8895160000	-2.4688580000
F	0	0.6630220000	6.6988810000	-3.5220370000
F	0	1.0202470000	6.5868440000	-1.3976410000
F	0	1.5311800000	4.9351610000	-2.6684070000
C	0	-1.9059250000	6.0759750000	-2.0374520000
C	0	-3.1226250000	5.4348500000	-1.7608950000
C	0	-4.4099750000	6.2031600000	-1.5077140000
F	0	-4.7692220000	6.9317070000	-2.5638630000
F	0	-5.4238170000	5.3760410000	-1.2384210000
F	0	-4.3030550000	7.0270540000	-0.4615770000
N	0	0.2613280000	1.2024020000	2.3165270000
C	0	1.3411310000	0.5265630000	2.5095000000
H	0	2.1771480000	1.0574760000	2.9714090000
C	0	1.5143690000	-0.8496070000	2.1043200000
C	0	2.7292370000	-1.5365940000	2.2416730000
H	0	3.5930130000	-1.0140800000	2.6560290000
C	0	2.8065380000	-2.8538950000	1.8192210000
H	0	3.7514810000	-3.3930140000	1.9120610000
C	0	1.6912410000	-3.5001530000	1.2544460000
H	0	1.7847180000	-4.5332680000	0.9136180000
C	0	0.4848880000	-2.8343010000	1.1185680000
H	0	-0.4011960000	-3.3093170000	0.6947770000
C	0	0.4012130000	-1.4990130000	1.5463080000
C	0	-0.8207530000	-0.7471960000	1.4473110000
N	0	-0.7709790000	0.5798810000	1.6906890000
C	0	-1.9725180000	1.3393340000	1.4706490000
O	0	-2.9987830000	0.7166960000	1.3454650000
C	0	-1.8913840000	2.8146470000	1.4772440000
C	0	-3.0535370000	3.5056570000	1.8459370000
H	0	-3.9490200000	2.9393110000	2.1077660000
C	0	-3.0694970000	4.8933750000	1.8869800000
H	0	-3.9668350000	5.4371590000	2.1823570000
C	0	-1.9140270000	5.5935420000	1.5351820000
C	0	-0.7688960000	4.9226140000	1.1065500000
H	0	0.1035720000	5.4910570000	0.7844440000
C	0	-0.7580600000	3.5353760000	1.0798610000
H	0	0.1277760000	3.0144490000	0.7215130000
C	0	9.3783340000	4.7474770000	-0.1239170000
C	0	9.2075690000	3.6292200000	-0.9378660000
C	0	8.6550680000	2.4619320000	-0.4121460000
H	0	8.5097330000	1.5890310000	-1.0550010000
H	0	9.4965090000	3.6674890000	-1.9903740000

H	0	9.8037400000	5.6655500000	-0.5345980000
C	0	8.9998360000	4.6884330000	1.2175920000
H	0	9.1284350000	5.5616850000	1.8607880000
C	0	8.4470900000	3.5212400000	1.7387120000
H	0	8.1350480000	3.4966610000	2.7847910000
C	0	8.2634470000	2.3971050000	0.9276960000
C	0	7.6348130000	1.1187940000	1.4443940000
H	0	7.2713050000	0.5501260000	0.5775640000
C	0	8.6134260000	0.1671710000	2.1265550000
C	0	9.9372500000	0.5109610000	2.4049520000
C	0	10.7917350000	-0.4043350000	3.0233960000
C	0	10.3318830000	-1.6716790000	3.3686130000
C	0	9.0110160000	-2.0254260000	3.0855910000
C	0	8.1611280000	-1.1149220000	2.4675450000
H	0	7.1318860000	-1.3950860000	2.2279740000
H	0	8.6441980000	-3.0223420000	3.3400380000
H	0	11.0014730000	-2.3872260000	3.8507480000
H	0	11.8257790000	-0.1212980000	3.2321890000
H	0	10.3118970000	1.4999190000	2.1341480000
N	0	6.4589490000	1.3530350000	2.2979130000
C	0	6.6180700000	1.4048290000	3.7444690000
H	0	6.3024060000	0.4645110000	4.2226960000
H	0	7.6719250000	1.5760040000	3.9890260000
H	0	6.0027550000	2.2158800000	4.1564990000
C	0	5.1751720000	1.3624950000	1.8495150000
O	0	4.2463070000	1.2989320000	2.6502800000
C	0	4.8741600000	1.5296190000	0.3497200000
H	0	5.7940300000	1.4142940000	-0.2361540000
C	0	4.3082700000	2.9497280000	0.0039140000
C	0	5.2949260000	4.0252310000	0.4697040000
H	0	5.4558860000	3.9964640000	1.5581540000
H	0	6.2718600000	3.9201300000	-0.0225660000
H	0	4.8972470000	5.0203150000	0.2202570000
C	0	4.1690570000	3.0308560000	-1.5251290000
H	0	3.4179490000	2.3227870000	-1.9051610000
H	0	3.8474430000	4.0401140000	-1.8193430000
H	0	5.1292830000	2.8203330000	-2.0216740000
C	0	2.9394810000	3.2178180000	0.6472030000
H	0	2.9939110000	3.2097900000	1.7436290000
H	0	2.5801710000	4.2064040000	0.3204150000
H	0	2.1811490000	2.4860130000	0.3302580000
N	0	3.9435340000	0.5151600000	-0.0981000000
H	0	2.9702200000	0.7962270000	-0.2255550000
C	0	4.2841370000	-0.7019460000	-0.5692150000
S	0	5.8289010000	-1.3503200000	-0.4254330000
N	0	3.2432950000	-1.3332380000	-1.1853440000
H	0	2.3964520000	-0.7640500000	-1.3254360000
C	0	3.2124640000	-2.5712720000	-1.8288160000
C	0	3.8312200000	-3.7156220000	-1.3276150000
H	0	4.4321800000	-3.6433070000	-0.4265160000
C	0	2.4640980000	-2.6766770000	-3.0011270000
H	0	1.9792830000	-1.7861330000	-3.4016300000
C	0	2.3337460000	-3.8924620000	-3.6686690000

C	0	1.5538660000	-3.9011280000	-4.9729290000
F	0	1.0673690000	-2.6876500000	-5.2531690000
F	0	2.3154940000	-4.2643240000	-6.0024770000
F	0	0.5065650000	-4.7327400000	-4.9296080000
C	0	2.9387370000	-5.0460130000	-3.1480550000
C	0	3.6851360000	-4.9442060000	-1.9626990000
C	0	4.3183080000	-6.1497020000	-1.2893880000
F	0	4.9318840000	-5.8010390000	-0.1514470000
F	0	3.3964150000	-7.0599960000	-0.9592930000
F	0	5.2282660000	-6.7452270000	-2.0550940000
Br	0	2.7538320000	-6.7093290000	-4.0272050000
Cl	0	0.7745140000	0.5949660000	-1.2313280000
Cl	0	-1.8941500000	7.3216280000	1.6312880000
Br	0	-1.8043200000	7.9607640000	-2.1326460000
H	0	-1.6458000000	-1.1195040000	0.8371640000
P	0	-2.0024850000	-1.5884530000	3.5088980000
O	0	-1.1764630000	-2.8284610000	4.1575900000
O	0	-1.7298920000	-0.4859330000	4.6936610000
O	0	-3.5020810000	-2.0674620000	3.7052960000
C	0	0.1374780000	-2.7446230000	4.6912020000
C	0	-2.2830800000	0.8200140000	4.6747940000
Si	0	-4.6009900000	-2.6034720000	4.9279280000
H	0	0.4002160000	-1.7086630000	4.9481430000
H	0	0.1623660000	-3.3520680000	5.6064300000
H	0	0.8625210000	-3.1477850000	3.9701150000
H	0	-1.5604430000	1.5382530000	4.2565870000
H	0	-3.2193420000	0.8584800000	4.0948040000
H	0	-2.5040770000	1.1028040000	5.7125050000
C	0	-6.2841090000	-2.3006220000	4.1880210000
C	0	-4.3102500000	-1.5392970000	6.4385670000
C	0	-4.2879770000	-4.4453300000	5.2659710000
H	0	-6.3233740000	-1.3133760000	3.7036520000
H	0	-6.5442320000	-3.0492330000	3.4257060000
H	0	-7.0606360000	-2.3300600000	4.9681640000
H	0	-3.2526110000	-1.5571210000	6.7405690000
H	0	-4.5946340000	-0.4942720000	6.2409010000
H	0	-4.9188620000	-1.8981540000	7.2836690000
C	0	-4.0238240000	-5.1714930000	3.9376300000
C	0	-3.0887570000	-4.6367450000	6.2071990000
C	0	-5.5502000000	-5.0247090000	5.9306530000
H	0	-3.1193610000	-4.7880450000	3.4422420000
H	0	-3.8811380000	-6.2512440000	4.1152390000
H	0	-4.8663970000	-5.0643510000	3.2355650000
H	0	-2.9153220000	-5.7118460000	6.3865920000
H	0	-2.1707840000	-4.2158260000	5.7733270000
H	0	-3.2562590000	-4.1635720000	7.1878860000
H	0	-5.3925590000	-6.0894510000	6.1738890000
H	0	-5.7976700000	-4.5103850000	6.8743790000
H	0	-6.4314560000	-4.9657190000	5.2728830000

TS-Si

0 1

C	0	-8.3911190000	2.6664940000	-0.2469400000
C	0	-7.1387910000	2.4373220000	0.3196000000
C	0	-6.4673090000	1.2405930000	0.0746710000
H	0	-5.5011660000	1.0508580000	0.5474660000
H	0	-6.6824720000	3.1947630000	0.9609060000
H	0	-8.9169020000	3.6045620000	-0.0571200000
C	0	-8.9701990000	1.6877040000	-1.0530720000
H	0	-9.9559320000	1.8526490000	-1.4933350000
C	0	-8.2910510000	0.4977610000	-1.3057870000
H	0	-8.7582910000	-0.2636180000	-1.9344590000
C	0	-7.0257860000	0.2645480000	-0.7557330000
C	0	-6.2037960000	-0.9705020000	-1.0946580000
H	0	-5.4272130000	-1.0644690000	-0.3236950000
C	0	-6.9596320000	-2.2924710000	-1.0884980000
C	0	-8.0347810000	-2.4992170000	-0.2173300000
C	0	-8.6460470000	-3.7476420000	-0.1213750000
C	0	-8.1976080000	-4.8132270000	-0.8999360000
C	0	-7.1297680000	-4.6183020000	-1.7738570000
C	0	-6.5164180000	-3.3698120000	-1.8625390000
H	0	-5.6685250000	-3.2313560000	-2.5352620000
H	0	-6.7656560000	-5.4438360000	-2.3893470000
H	0	-8.6778760000	-5.7910170000	-0.8251370000
H	0	-9.4794110000	-3.8867800000	0.5708080000
H	0	-8.3967190000	-1.6759450000	0.4002900000
N	0	-5.4652790000	-0.7612520000	-2.3503360000
C	0	-6.2523710000	-0.4155480000	-3.5219060000
H	0	-6.5118150000	0.6542860000	-3.5237090000
H	0	-5.7216110000	-0.6530810000	-4.4457770000
H	0	-7.1739280000	-1.0133200000	-3.5180640000
C	0	-4.1192320000	-0.6186100000	-2.2760180000
O	0	-3.5151590000	-0.7289430000	-1.2103600000
C	0	-3.2967270000	-0.3323940000	-3.5537460000
H	0	-3.9079660000	0.2105820000	-4.2860580000
C	0	-2.7338990000	-1.6278290000	-4.2287370000
C	0	-1.6709660000	-1.2196510000	-5.2624050000
H	0	-1.3836330000	-2.0975910000	-5.8610760000
H	0	-2.0532500000	-0.4491760000	-5.9501150000
H	0	-0.7589980000	-0.8329290000	-4.7866130000
C	0	-2.1083990000	-2.5808940000	-3.2020670000
H	0	-2.8663350000	-3.0091570000	-2.5305260000
H	0	-1.6183490000	-3.4175360000	-3.7222590000
H	0	-1.3507100000	-2.0859980000	-2.5773690000
C	0	-3.8608690000	-2.3647920000	-4.9631920000
H	0	-4.2905820000	-1.7504040000	-5.7701930000
H	0	-3.4643090000	-3.2826050000	-5.4221330000
H	0	-4.6711980000	-2.6647340000	-4.2834950000
N	0	-2.2148880000	0.5408700000	-3.1799390000
H	0	-1.3429780000	0.0944520000	-2.8774960000
C	0	-2.3764870000	1.8377140000	-2.8542960000
N	0	-1.2071130000	2.3853410000	-2.3871020000

H	0	-0.4447680000	1.7024870000	-2.2974680000
C	0	-0.9572990000	3.5707580000	-1.7040190000
C	0	-1.7112750000	4.7398950000	-1.8185880000
H	0	-2.5605720000	4.7619850000	-2.4965380000
C	0	0.1374850000	3.5649360000	-0.8311050000
H	0	0.7347490000	2.6558720000	-0.7374250000
C	0	0.4592420000	4.6784790000	-0.0661710000
C	0	1.6465540000	4.5648960000	0.8719480000
F	0	1.2975090000	4.7432110000	2.1554210000
F	0	2.2110260000	3.3596220000	0.8016370000
F	0	2.5934960000	5.4600410000	0.5861270000
C	0	-0.3290840000	5.8368760000	-0.1473160000
Br	0	0.0520290000	7.3319040000	0.9481400000
C	0	-1.4130990000	5.8537380000	-1.0354700000
C	0	-2.3449220000	7.0480240000	-1.1477840000
F	0	-2.9792310000	7.2719600000	0.0124200000
F	0	-1.7036370000	8.1655930000	-1.4820310000
F	0	-3.2881450000	6.8466500000	-2.0681320000
C	0	9.0317510000	-0.2500300000	3.4056690000
C	0	8.4275200000	-0.8246670000	2.2899540000
C	0	7.6547440000	-0.0469480000	1.4289380000
H	0	7.1813200000	-0.5149880000	0.5611720000
H	0	8.5502970000	-1.8896370000	2.0849400000
H	0	9.6387960000	-0.8601560000	4.0779640000
C	0	8.8523440000	1.1092710000	3.6582290000
H	0	9.3142710000	1.5698590000	4.5344600000
C	0	8.0845190000	1.8879210000	2.7951910000
H	0	7.9504590000	2.9518230000	3.0027760000
C	0	7.4828870000	1.3215150000	1.6650270000
C	0	6.5734450000	2.1570300000	0.7770370000
H	0	5.5748020000	2.1228410000	1.2333940000
C	0	6.9441740000	3.6235180000	0.6107670000
C	0	5.9110480000	4.5641970000	0.5348990000
C	0	6.1840600000	5.9056290000	0.2765220000
C	0	7.5007160000	6.3251190000	0.0894990000
C	0	8.5379010000	5.3961770000	0.1656710000
C	0	8.2615590000	4.0544350000	0.4242970000
H	0	9.0818800000	3.3358910000	0.4921280000
H	0	9.5720020000	5.7178090000	0.0229170000
H	0	7.7187720000	7.3763440000	-0.1112600000
H	0	5.3628170000	6.6234910000	0.2245880000
H	0	4.8788530000	4.2357740000	0.6733900000
N	0	6.3789540000	1.5675500000	-0.5505990000
C	0	7.5406740000	1.4254760000	-1.4102330000
H	0	8.4320410000	1.3280180000	-0.7776900000
H	0	7.4674280000	0.5027050000	-2.0003430000
H	0	7.6847270000	2.2936960000	-2.0701970000
C	0	5.1008200000	1.2995590000	-0.9308040000
O	0	4.1605210000	1.3726070000	-0.1487310000
C	0	4.8077160000	0.9257560000	-2.3924800000
H	0	5.7144110000	0.5634560000	-2.8901360000
C	0	4.2761460000	2.1385580000	-3.2210600000
C	0	5.3582720000	3.2251780000	-3.2730390000

H	0	5.6159790000	3.6024570000	-2.2706780000
H	0	6.2764360000	2.8541200000	-3.7536340000
H	0	4.9984850000	4.0811800000	-3.8634170000
C	0	3.9885790000	1.6436000000	-4.6457520000
H	0	3.6756240000	2.4846290000	-5.2834830000
H	0	4.8833960000	1.1879450000	-5.0978910000
H	0	3.1858650000	0.8926450000	-4.6545020000
C	0	2.9963930000	2.7444710000	-2.6282340000
H	0	2.1794870000	2.0093010000	-2.5799970000
H	0	3.1616170000	3.1339640000	-1.6152610000
H	0	2.6540620000	3.5736240000	-3.2672140000
N	0	3.8615110000	-0.1625470000	-2.3931460000
H	0	2.8779840000	0.0884240000	-2.2644600000
C	0	4.1984280000	-1.4420610000	-2.1171720000
S	0	5.7909460000	-1.9273330000	-1.8944130000
N	0	3.0949810000	-2.2547600000	-2.0667600000
H	0	2.2142800000	-1.7525310000	-2.2131840000
C	0	2.8984250000	-3.5419830000	-1.5743590000
C	0	3.9072540000	-4.4403080000	-1.2200880000
H	0	4.9467350000	-4.1546910000	-1.3598360000
C	0	1.5655650000	-3.9447230000	-1.4113810000
H	0	0.7639440000	-3.2576280000	-1.6865330000
C	0	1.2411400000	-5.1770480000	-0.8582730000
C	0	-0.2288460000	-5.5135180000	-0.6858930000
F	0	-0.5488890000	-6.6741750000	-1.2596370000
F	0	-0.5743210000	-5.5957450000	0.6067850000
F	0	-1.0161450000	-4.5879020000	-1.2373990000
C	0	2.2605550000	-6.0485740000	-0.4428780000
Br	0	1.8389590000	-7.6752940000	0.4292750000
C	0	3.5938490000	-5.6727750000	-0.6510840000
C	0	4.7573390000	-6.5610410000	-0.2414440000
F	0	4.7064370000	-7.7539560000	-0.8371080000
F	0	5.9286900000	-6.0153080000	-0.5633030000
F	0	4.7733390000	-6.7698400000	1.0807600000
Cl	0	0.6315700000	-0.1899150000	-1.9080500000
N	0	0.4607720000	0.4057040000	2.2566490000
C	0	0.0169300000	1.5663710000	2.6028780000
H	0	0.5921890000	2.0776180000	3.3795560000
C	0	-1.1386570000	2.1936840000	2.0161830000
C	0	-1.5642490000	3.4911610000	2.3410090000
H	0	-1.0106840000	4.0767630000	3.0762130000
C	0	-2.6535030000	4.0343270000	1.6795350000
H	0	-2.9609430000	5.0597200000	1.8936080000
C	0	-3.3473340000	3.2901210000	0.7090530000
H	0	-4.1899130000	3.7313260000	0.1735100000
C	0	-2.9572250000	2.0006260000	0.4018520000
H	0	-3.4885260000	1.4125110000	-0.3455250000
C	0	-1.8365450000	1.4513000000	1.0424010000
C	0	-1.3640880000	0.1332980000	0.7397300000
H	0	-1.7272790000	-0.4154170000	-0.1326750000
N	0	-0.1969430000	-0.2600040000	1.2817700000
C	0	0.2464830000	-1.6177230000	1.0181980000
O	0	-0.5853570000	-2.3929660000	0.6256370000

C	0	1.6439580000	-1.9645930000	1.3260160000
C	0	2.7150900000	-1.0930510000	1.0964620000
H	0	2.5673330000	-0.0848280000	0.7071120000
C	0	4.0171480000	-1.5307190000	1.3070050000
H	0	4.8534000000	-0.8624450000	1.1060840000
C	0	4.2376020000	-2.8330190000	1.7525040000
Cl	0	5.8595430000	-3.4068800000	1.9673960000
C	0	3.1787550000	-3.7088610000	1.9992850000
H	0	3.3776870000	-4.7297510000	2.3258280000
C	0	1.8830650000	-3.2728370000	1.7691450000
H	0	1.0453490000	-3.9590170000	1.9060040000
P	0	-3.1643330000	-1.2480910000	2.0445570000
O	0	-4.2483670000	-2.2690840000	1.3990090000
O	0	-2.7571180000	-2.1003420000	3.3895790000
O	0	-4.1533470000	-0.1205650000	2.5919210000
C	0	-3.7986950000	-3.3833020000	0.6308810000
C	0	-1.4426040000	-2.1368480000	3.9123890000
Si	0	-5.0223500000	0.3042280000	4.0088500000
H	0	-3.1781230000	-4.0568610000	1.2404830000
H	0	-4.6992500000	-3.9127140000	0.2968960000
H	0	-3.2219940000	-3.0419050000	-0.2396440000
H	0	-0.8372720000	-2.8989360000	3.3984830000
H	0	-0.9323630000	-1.1626340000	3.8391900000
H	0	-1.5175010000	-2.4041160000	4.9744340000
C	0	-5.6048180000	2.0454530000	3.6732380000
C	0	-3.8100650000	0.2856800000	5.4353330000
C	0	-6.4568430000	-0.9130370000	4.2307350000
H	0	-4.7724210000	2.6521320000	3.2835360000
H	0	-6.4160940000	2.0561180000	2.9318700000
H	0	-5.9761420000	2.5196310000	4.5948300000
H	0	-3.4124850000	-0.7240310000	5.6096400000
H	0	-2.9656360000	0.9619220000	5.2267020000
H	0	-4.2942400000	0.6308390000	6.3623040000
C	0	-7.1731410000	-1.1023390000	2.8838020000
C	0	-5.9380780000	-2.2732360000	4.7251960000
C	0	-7.4369170000	-0.3293110000	5.2636080000
H	0	-6.5027470000	-1.5487630000	2.1345530000
H	0	-8.0347890000	-1.7815970000	3.0076730000
H	0	-7.5570260000	-0.1537100000	2.4728600000
H	0	-6.7757210000	-2.9861550000	4.8162750000
H	0	-5.2026150000	-2.7093300000	4.0329720000
H	0	-5.4631470000	-2.1952860000	5.7161290000
H	0	-8.2657750000	-1.0365870000	5.4390280000
H	0	-6.9561750000	-0.1463600000	6.2392130000
H	0	-7.8826380000	0.6183520000	4.9215500000
S	0	-3.8543540000	2.6268870000	-2.9954690000

21. NMR spectra of new compounds.

^{1}H-NMR ($CDCl_3$, 300 MHz) of **1m**

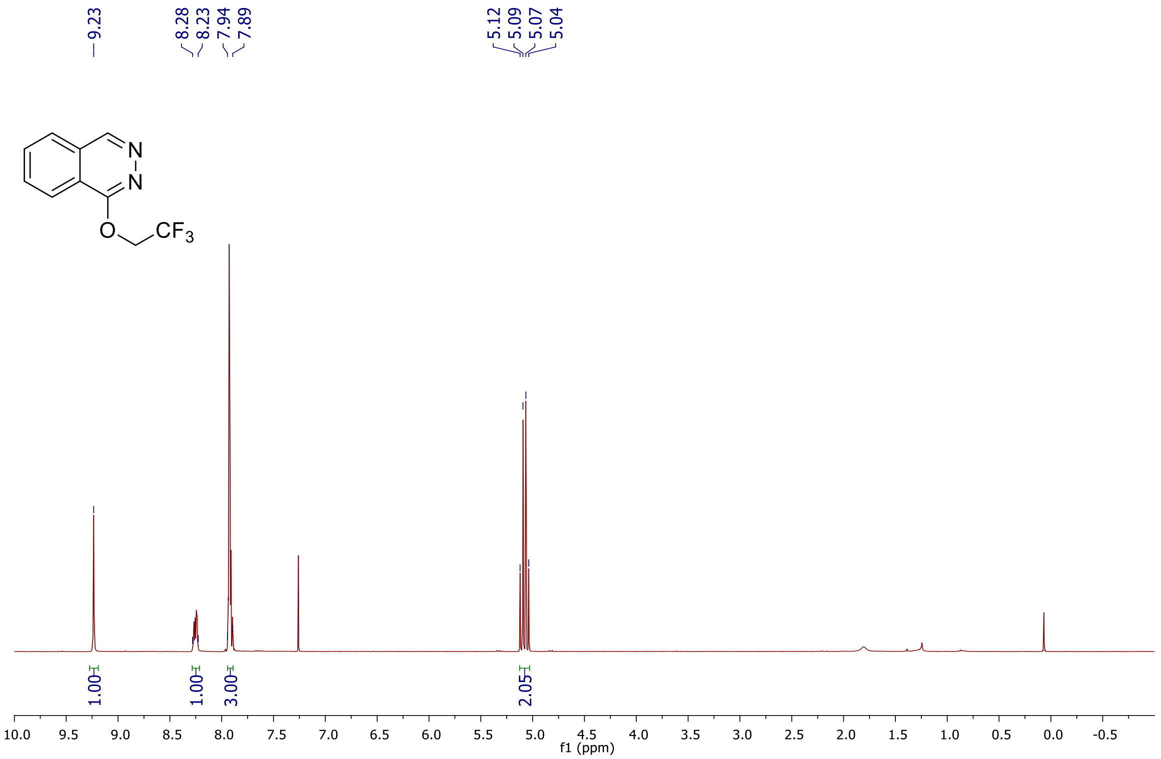

^{13}C-NMR ($CDCl_3$, 75.5 MHz) of **1m**

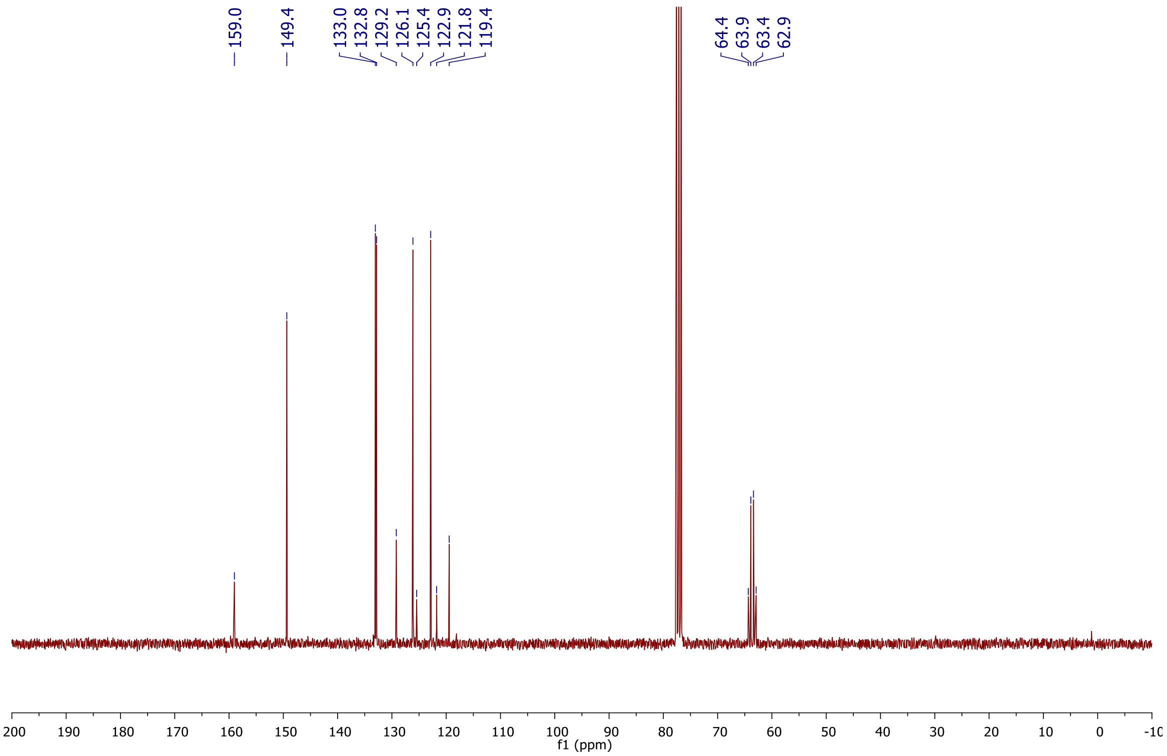

^{1}**H-NMR** ($CDCl_3$, 300 MHz) of **1n**

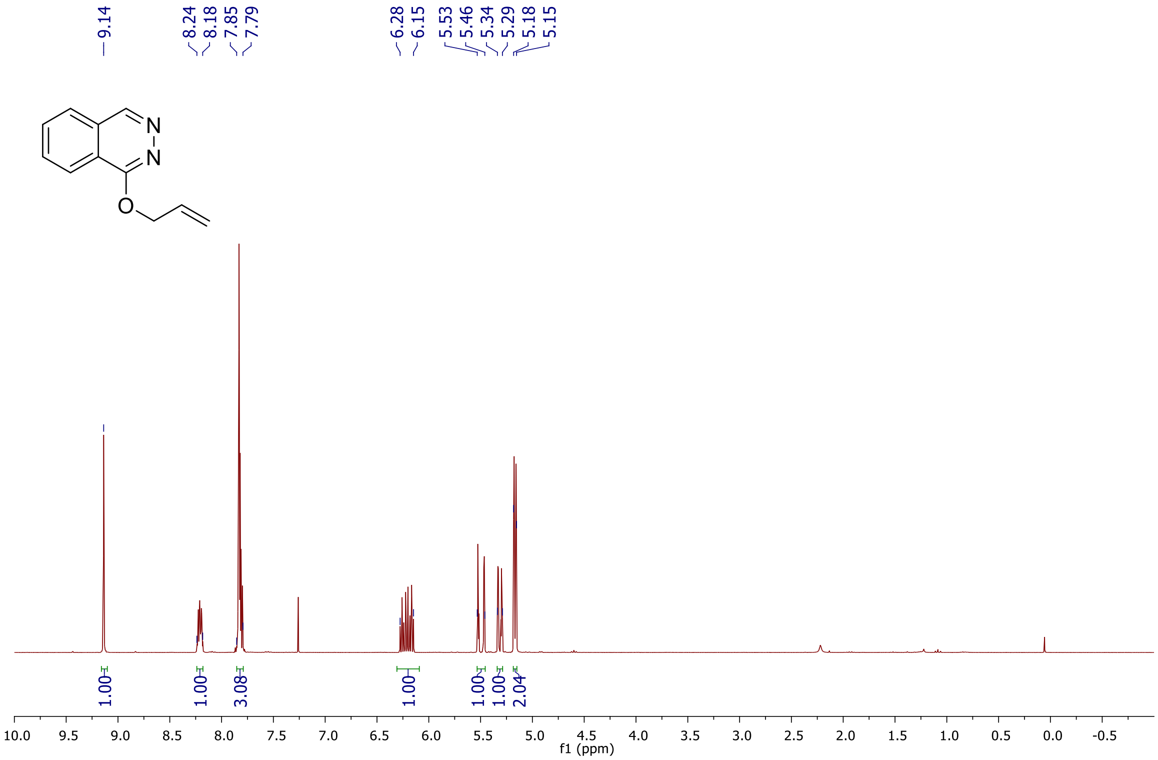

^{13}**C-NMR** ($CDCl_3$, 75.5 MHz) of **1n**

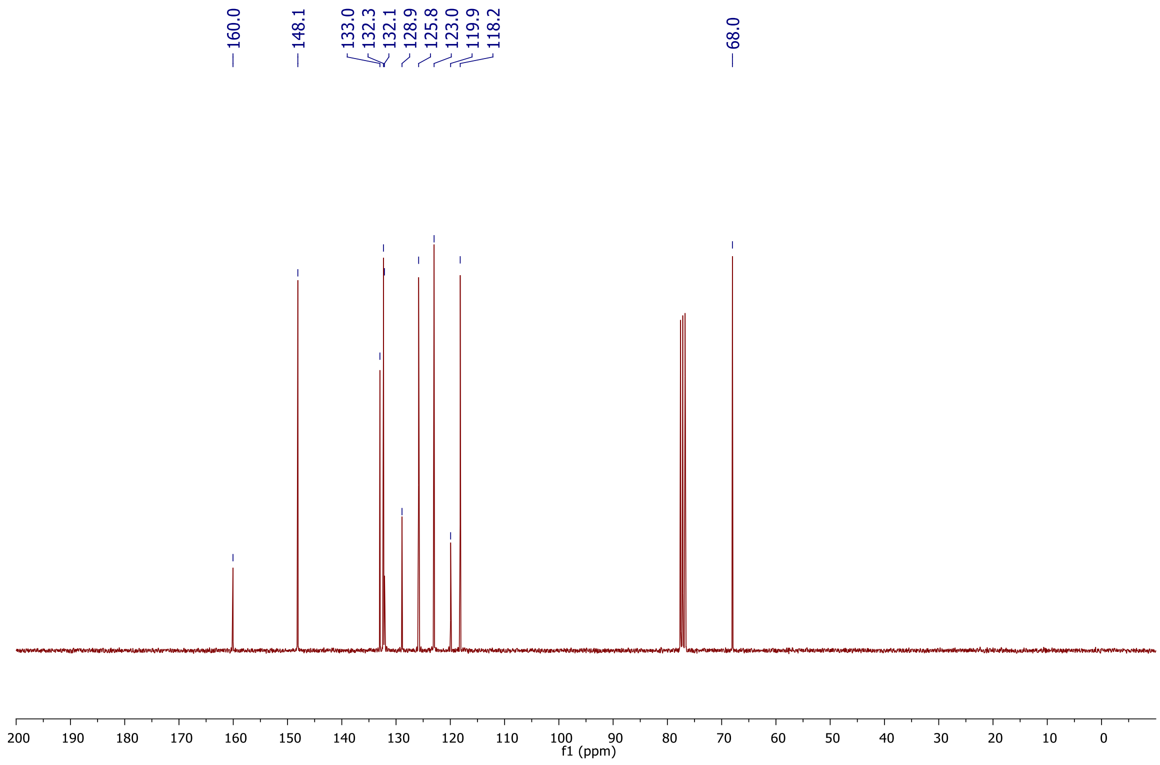

^{1}H-NMR (CDCl$_3$, 300 MHz) of **1o**

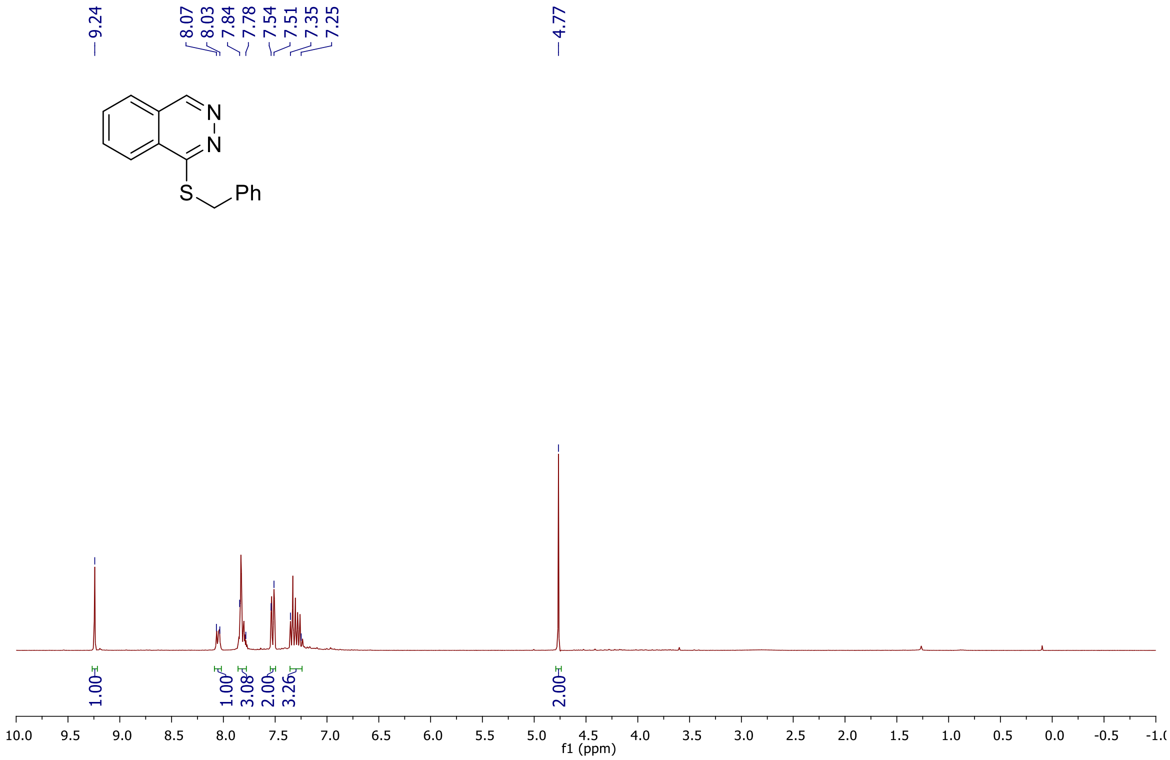

^{13}C-NMR (CDCl$_3$, 75.5 MHz) of **1o**

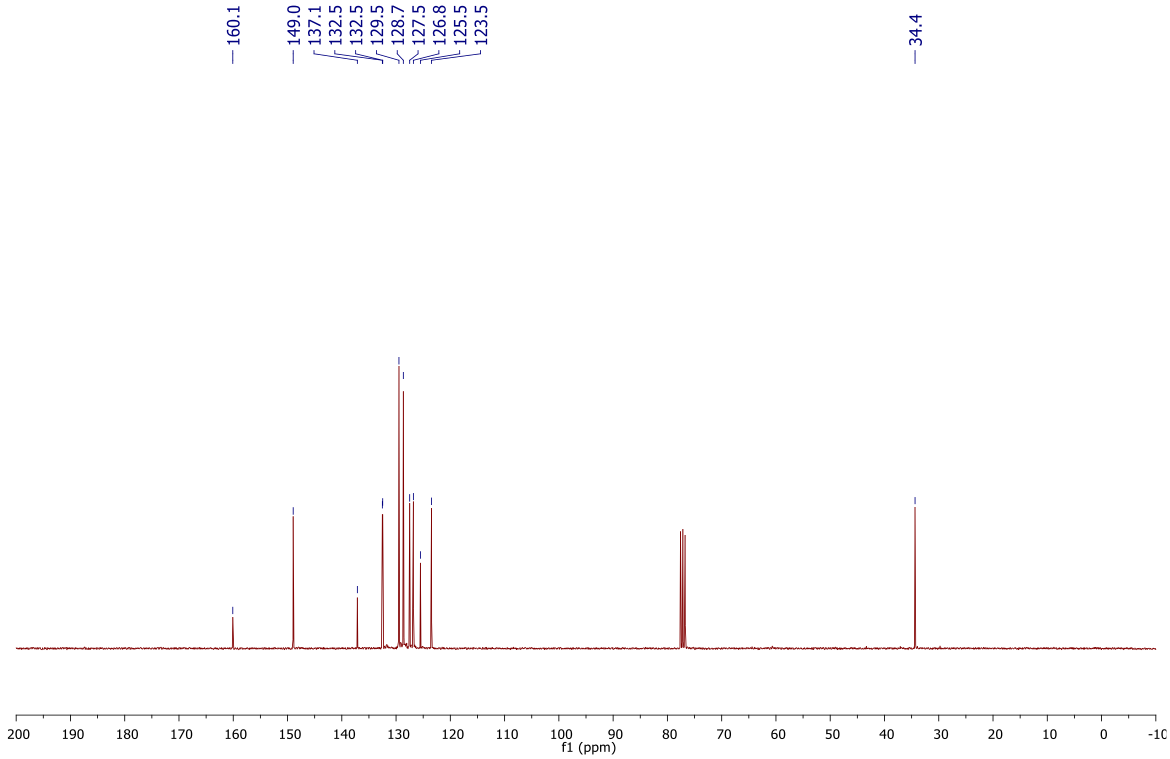

^{1}H-NMR (CDCl$_3$, 300 MHz) of **1q**

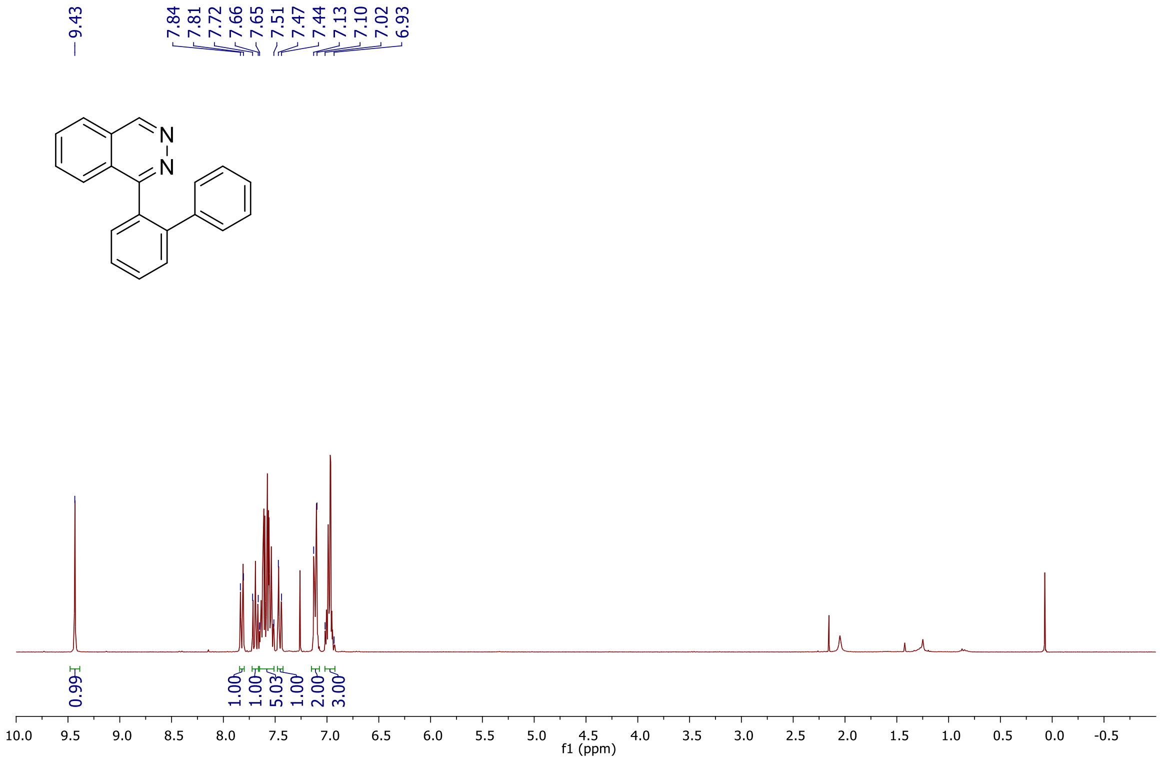

^{13}C-NMR (CDCl$_3$, 75.5 MHz) of **1q**

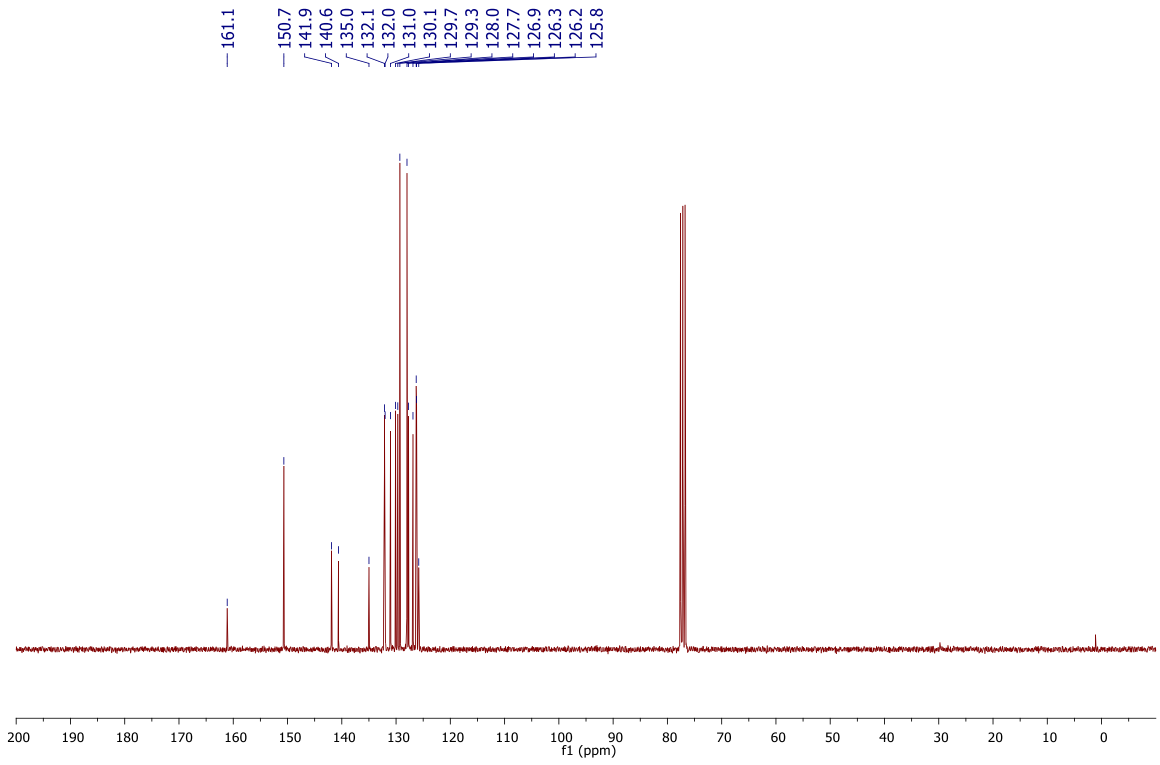

[1]**H-NMR** (CD_2Cl_2, 300 MHz) of ***Pht*-1**

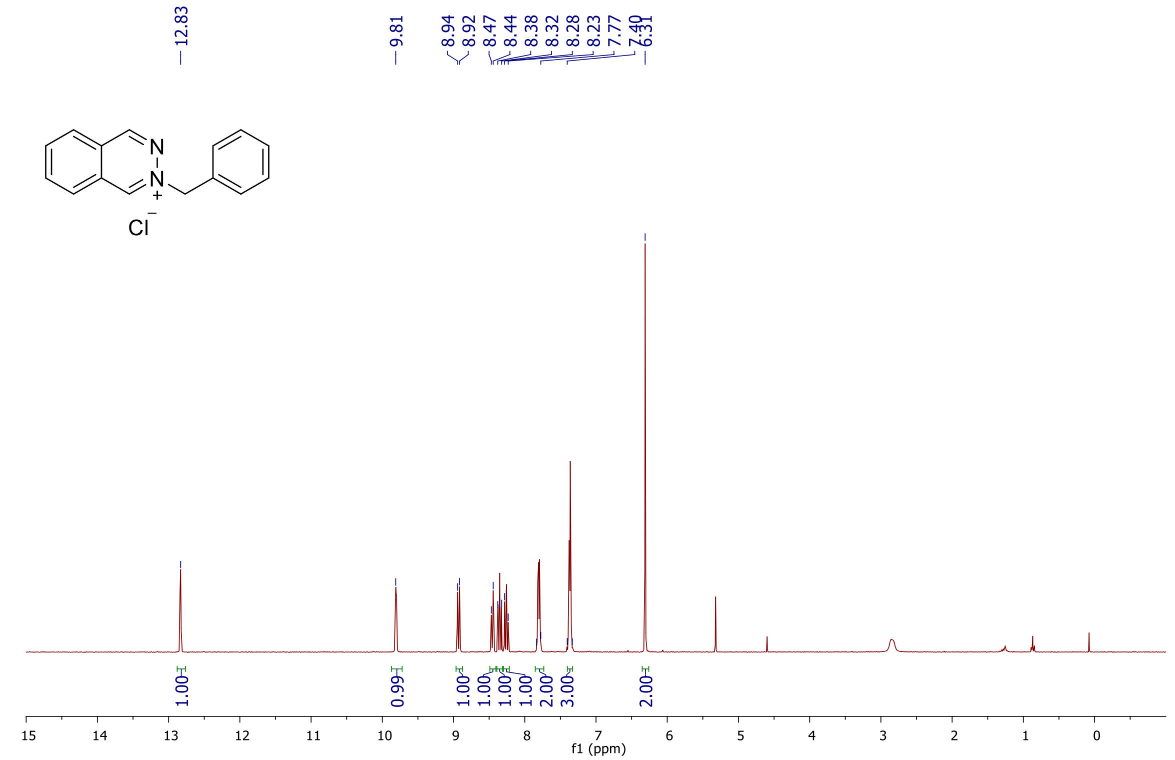

[13]**C-NMR** (CD_2Cl_2, 75.5 MHz) of ***Pht*-1**

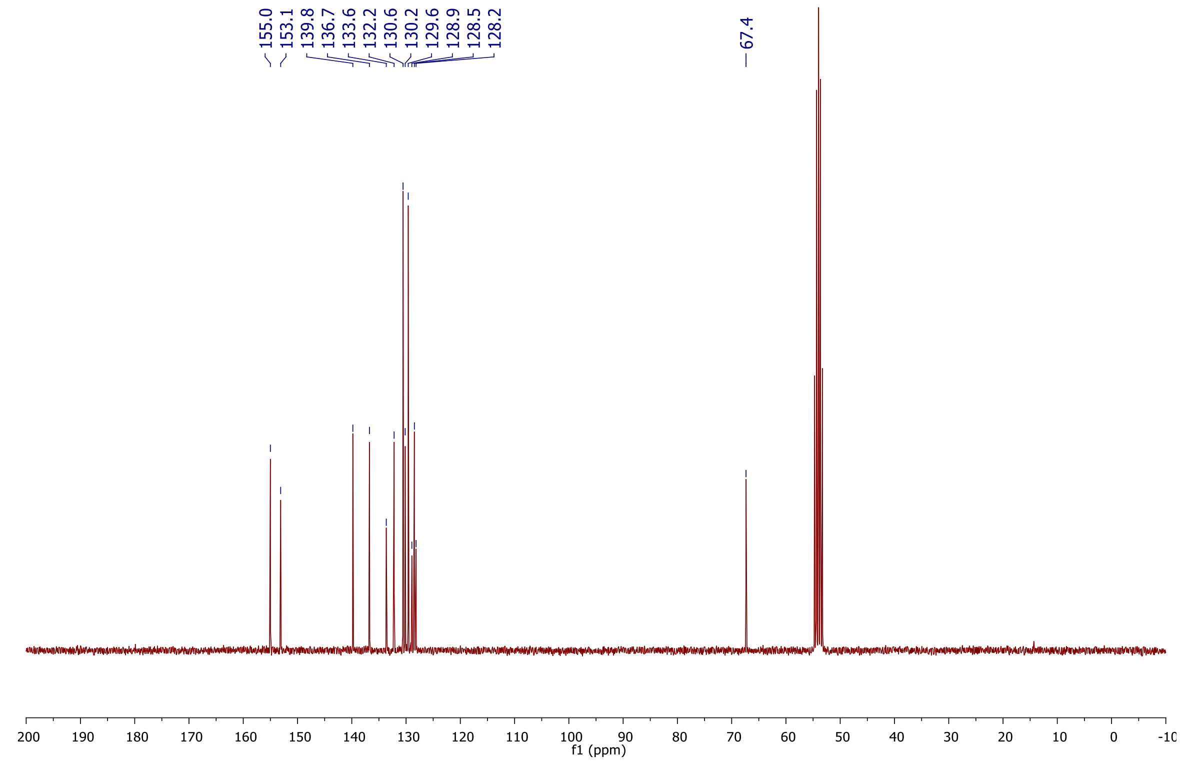

^{1}H NMR (CD_2Cl_2, 300 MHz) of **VII**

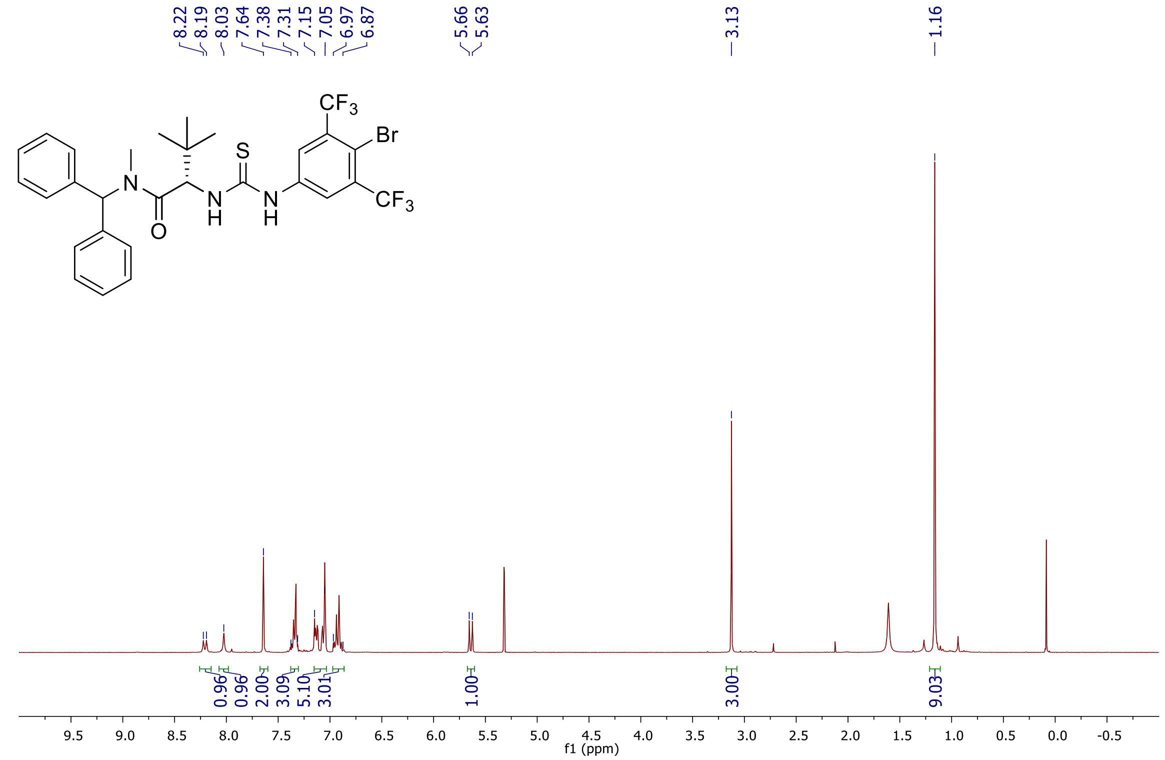

^{13}C NMR (CD_2Cl_2, 126 MHz) of **VII**

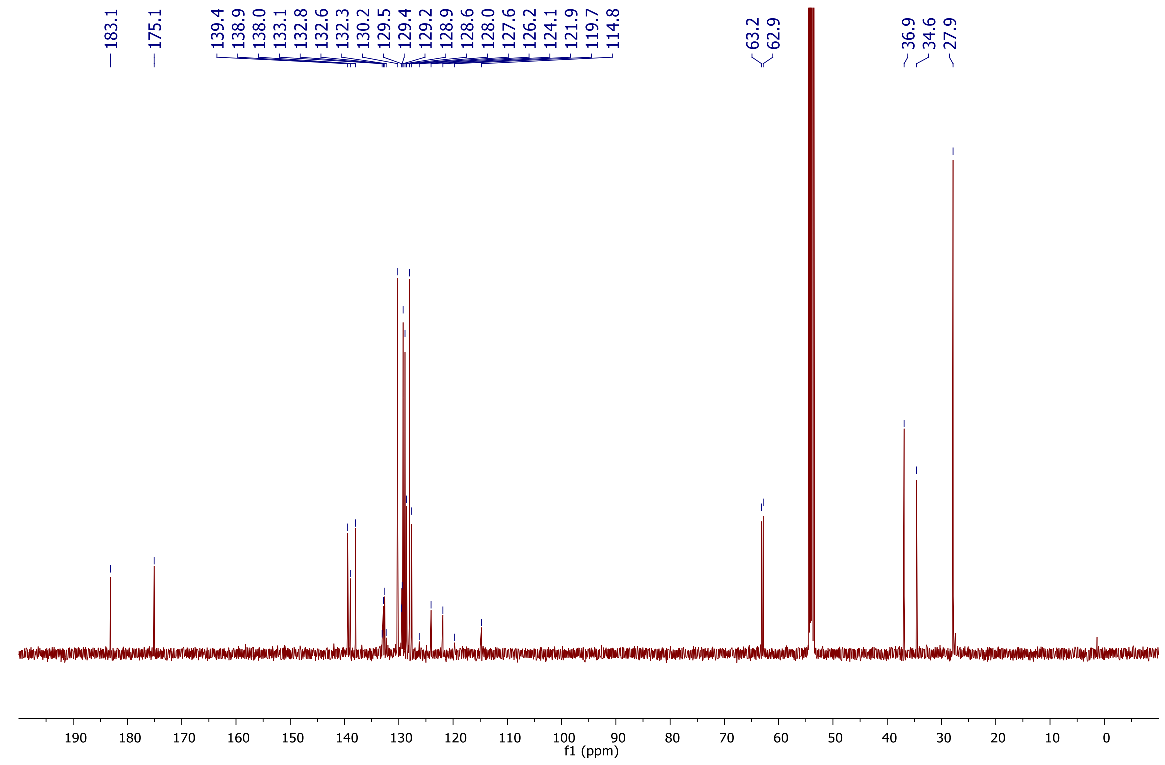

^{1}H-NMR (CDCl$_3$, 300 MHz) of **XI**

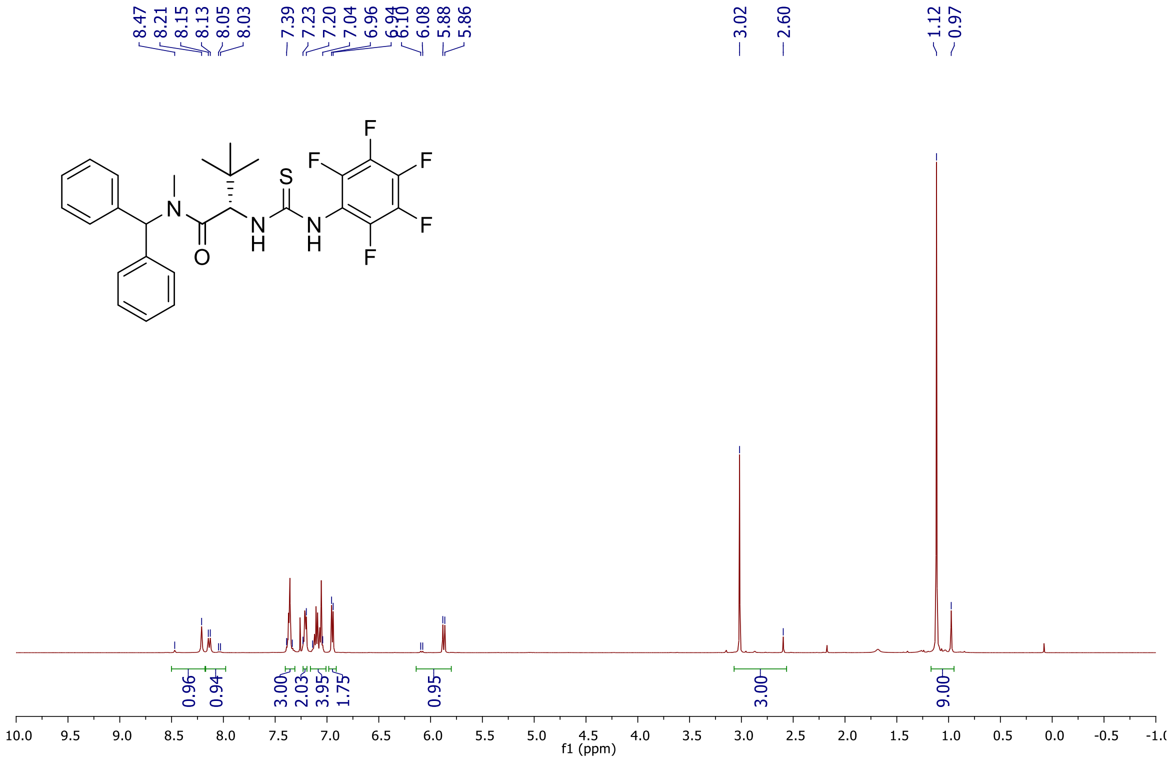

^{13}C-NMR (CDCl$_3$, 126 MHz) of **XI**

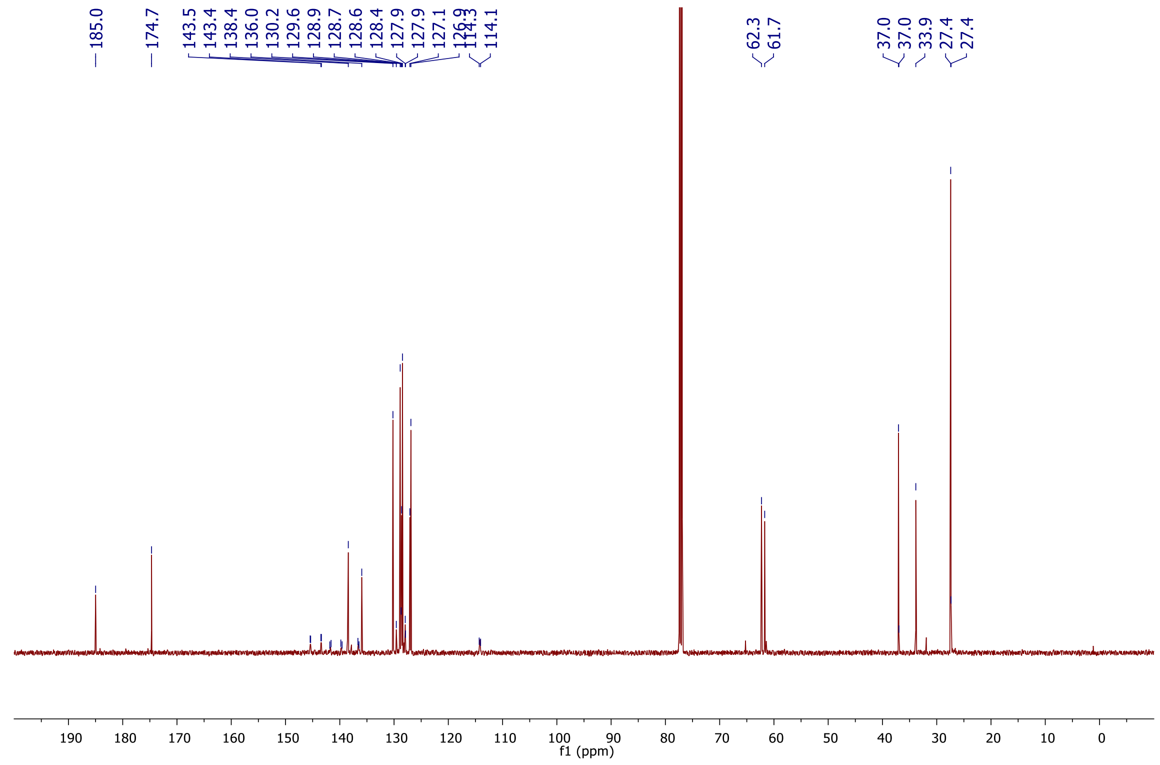

^{1}H-NMR ($CDCl_3$, 300 MHz) of **XII**

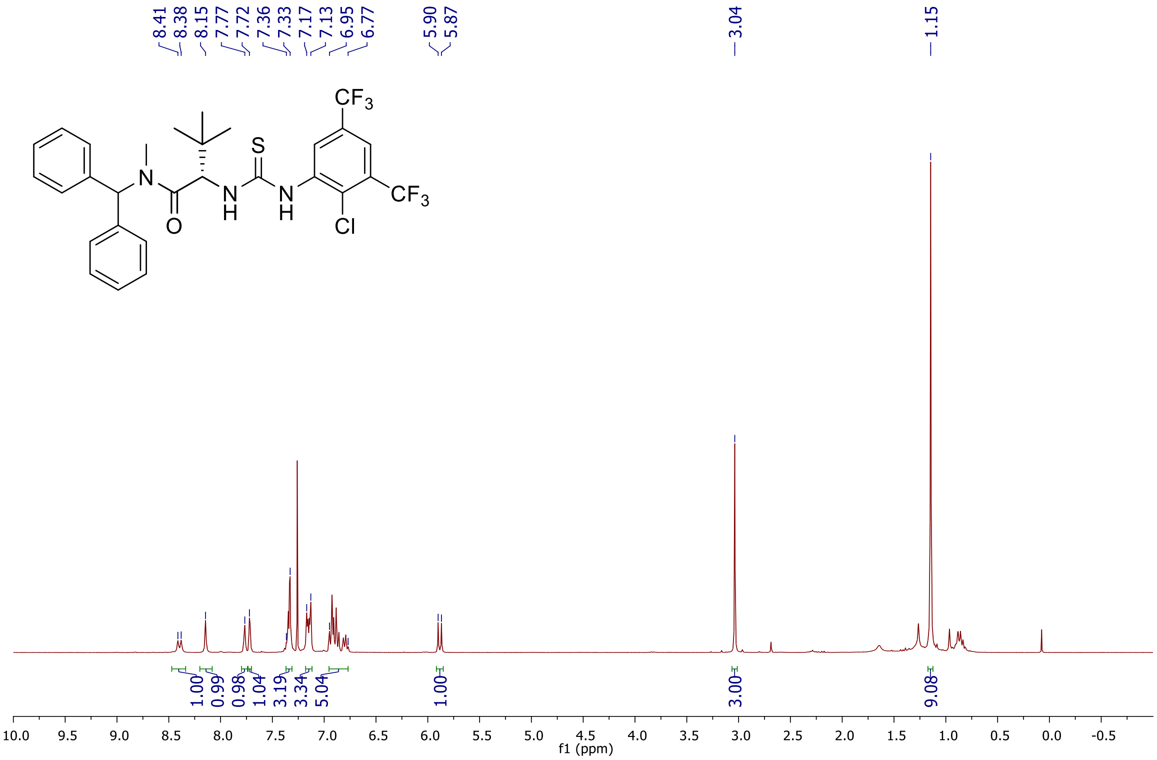

^{13}C-NMR ($CDCl_3$, 75.5 MHz) of **XII**

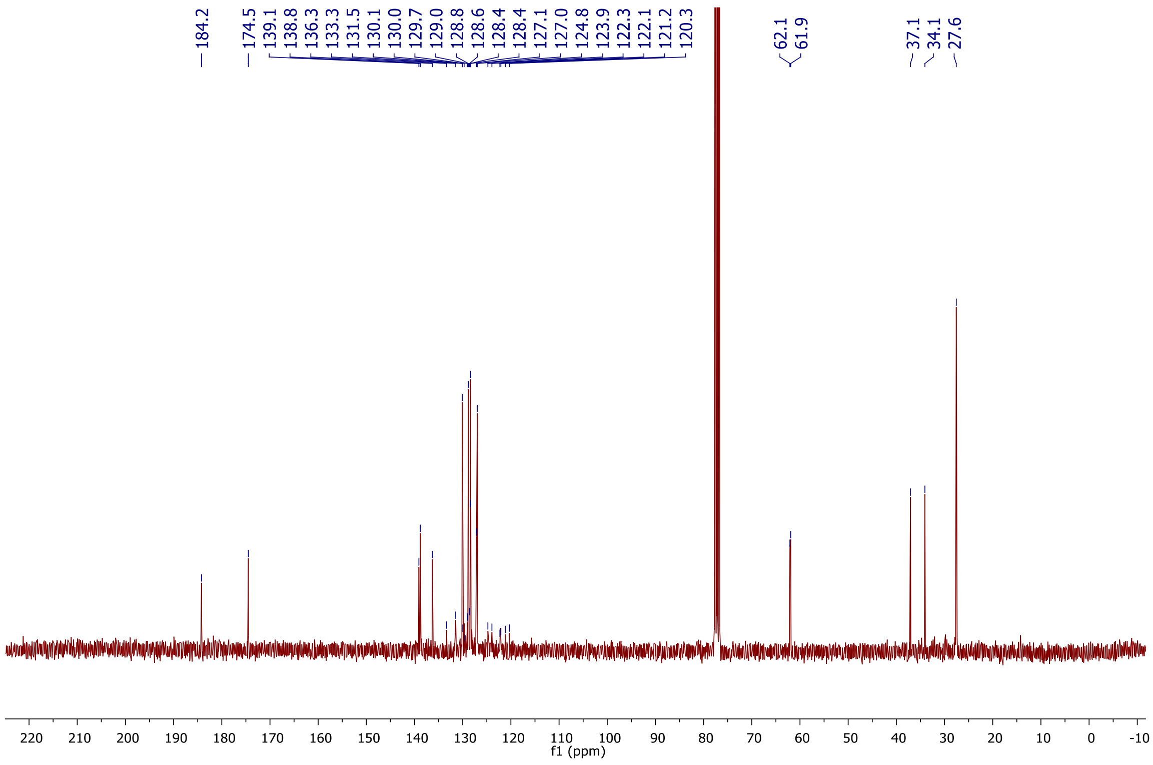

^{1}H-NMR (CDCl$_3$, 300 MHz) of **XIII**

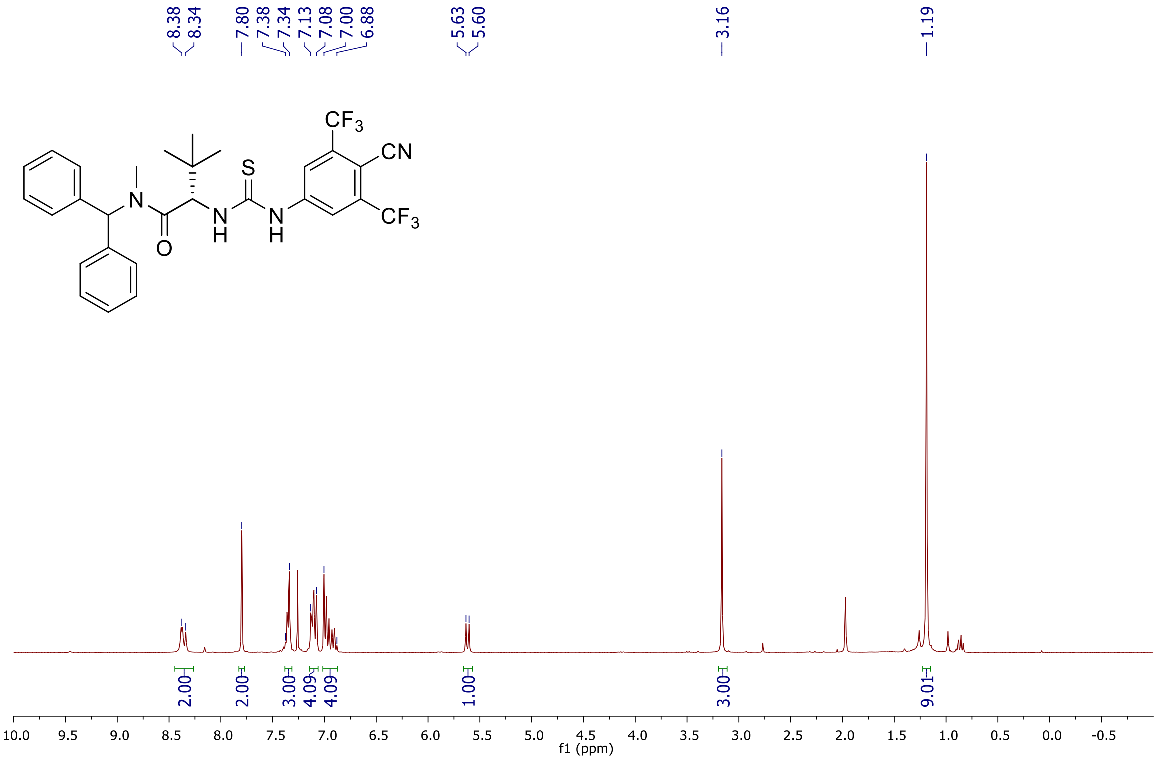

^{13}C-NMR (CDCl$_3$, 75.5 MHz) of **XIII**

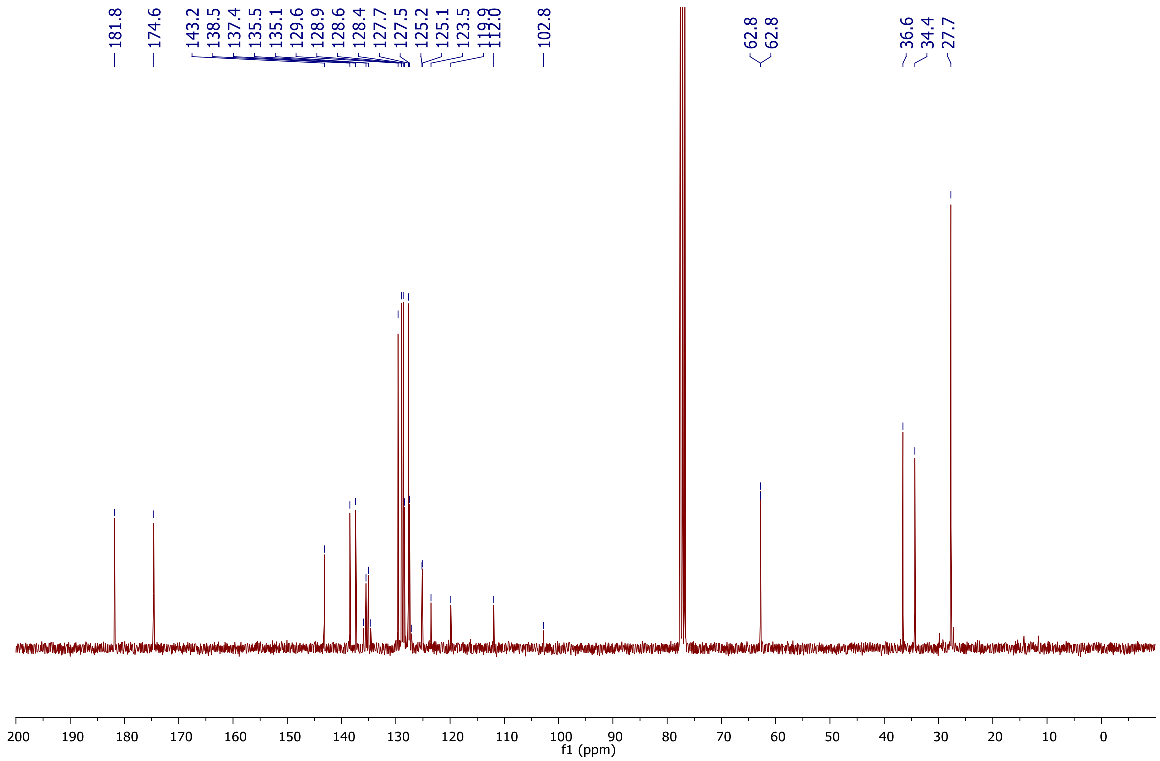

^{1}H-NMR (CD_2Cl_2, 300 MHz) of **2A-TIPS**

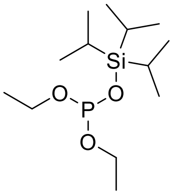

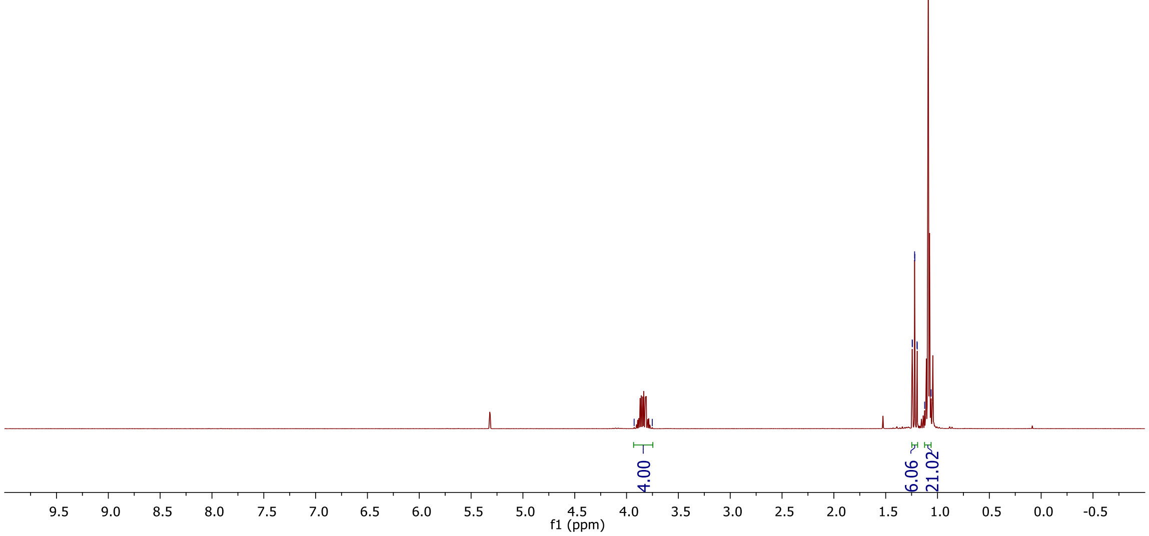

^{13}C-NMR (CD_2Cl_2, 75.5 MHz) of **2A-TIPS**

^{31}P-NMR (122 MHz):

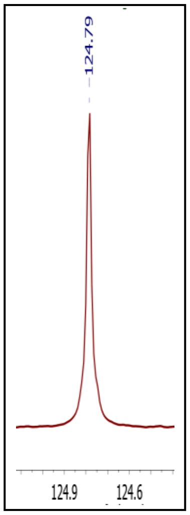

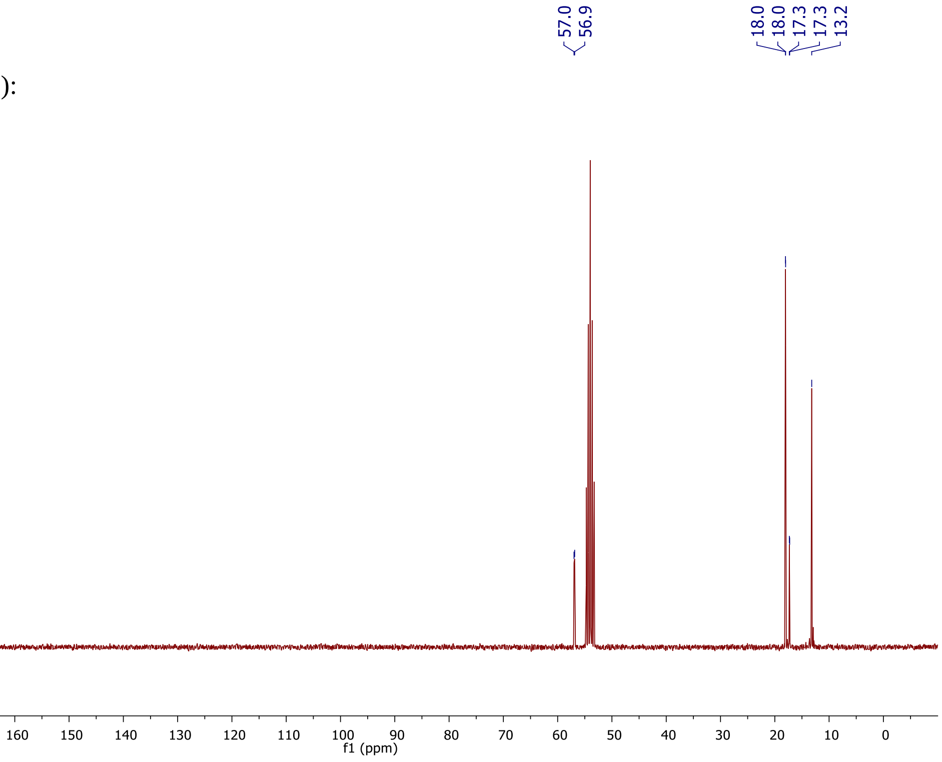

^{1}H-NMR (CD_2Cl_2, 300 MHz) of **2B**

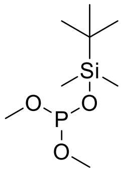

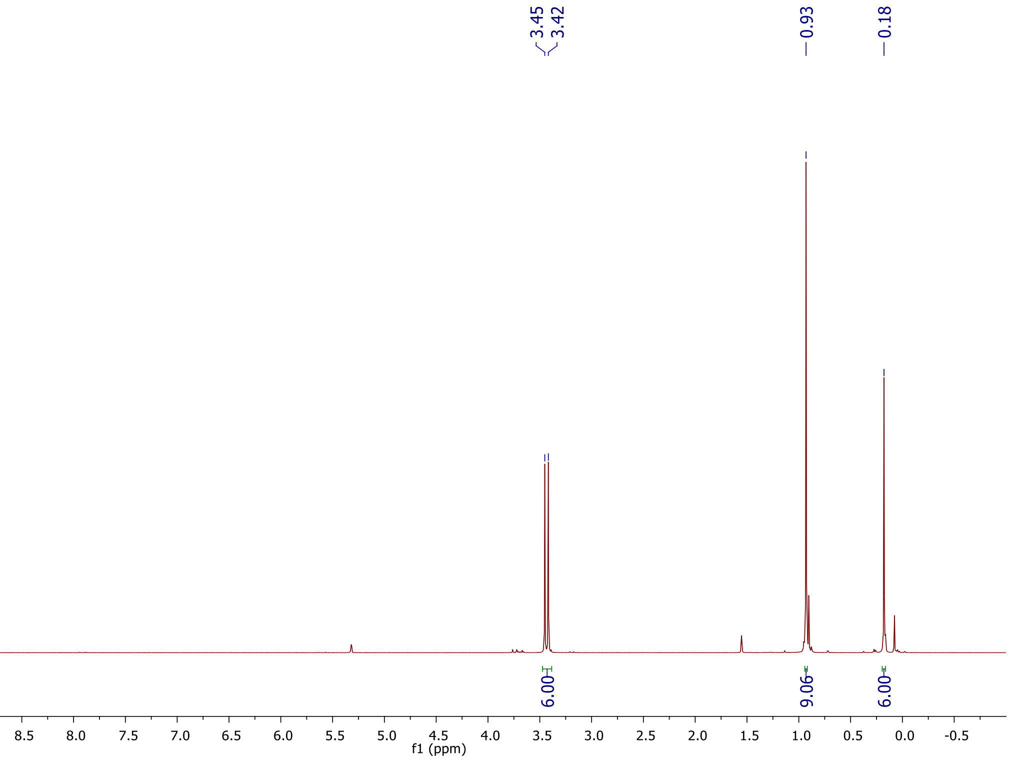

^{13}C-NMR (CD_2Cl_2, 75.5 MHz) of **2B**

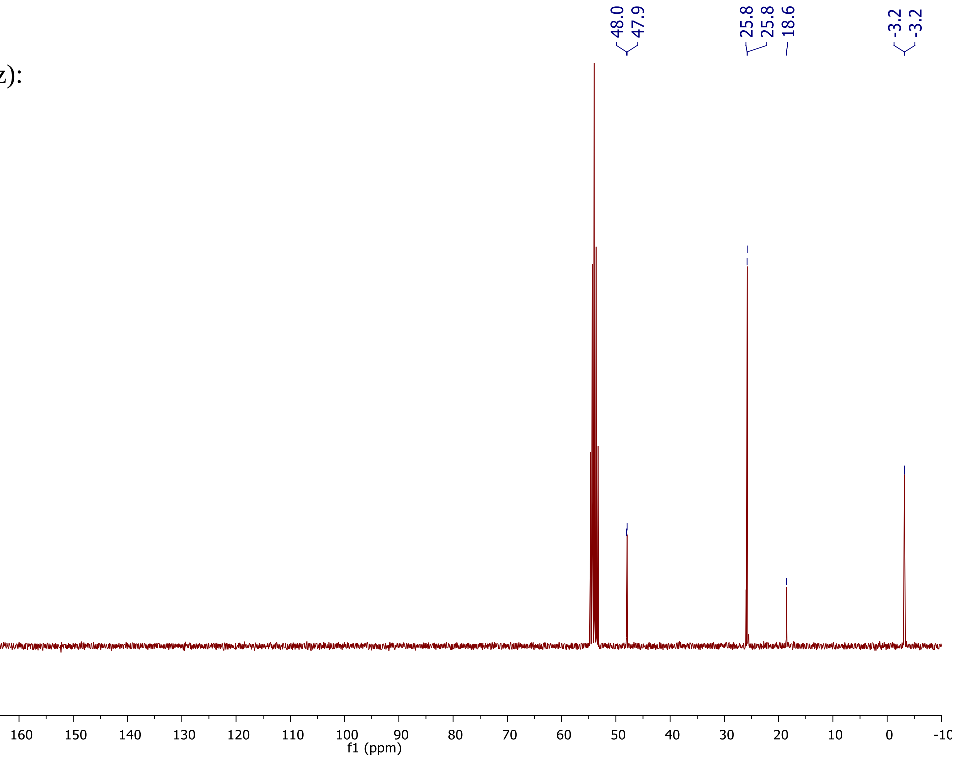

^{31}P-NMR (122 MHz):

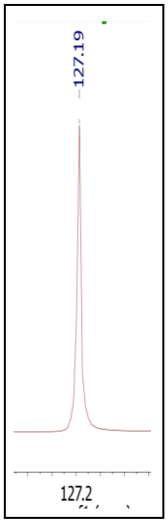

^{1}H-NMR (CD_2Cl_2, 300 MHz) of **2C**

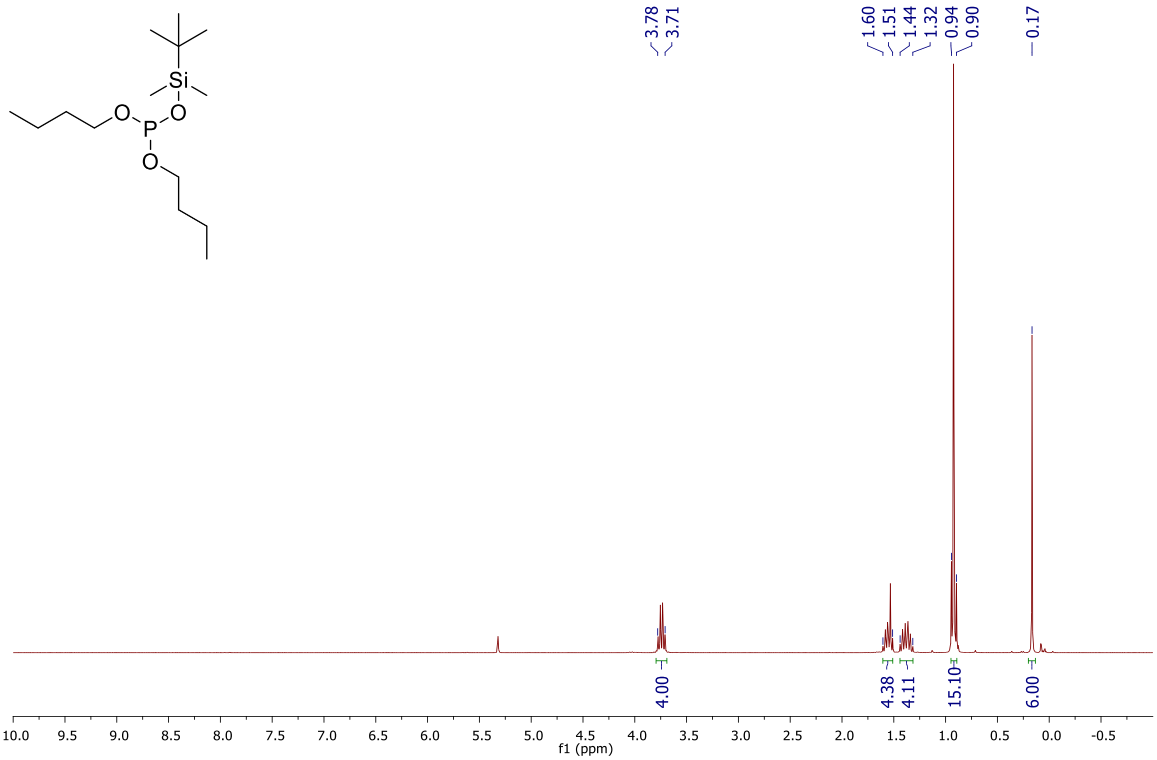

^{13}C-NMR (CD_2Cl_2, 75.5 MHz) of **2C**

^{31}P-NMR (122 MHz):

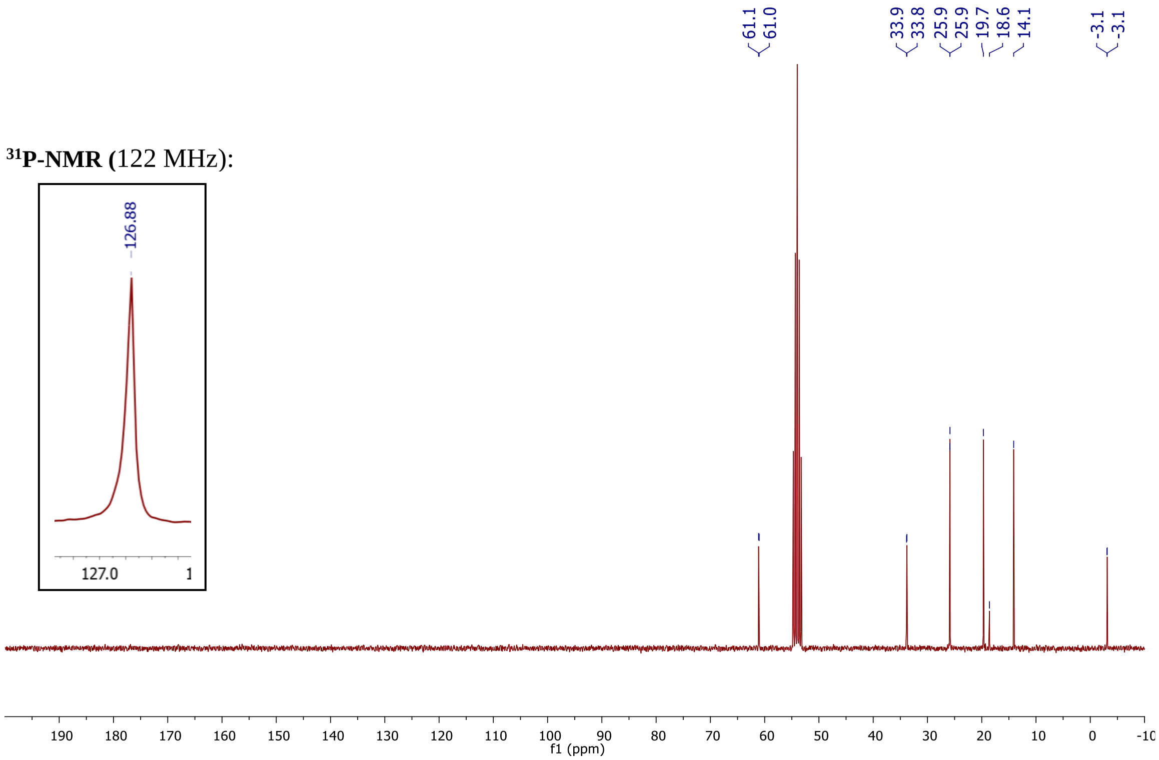

^{1}H-NMR (CD_2Cl_2, 300 MHz) of **2E**

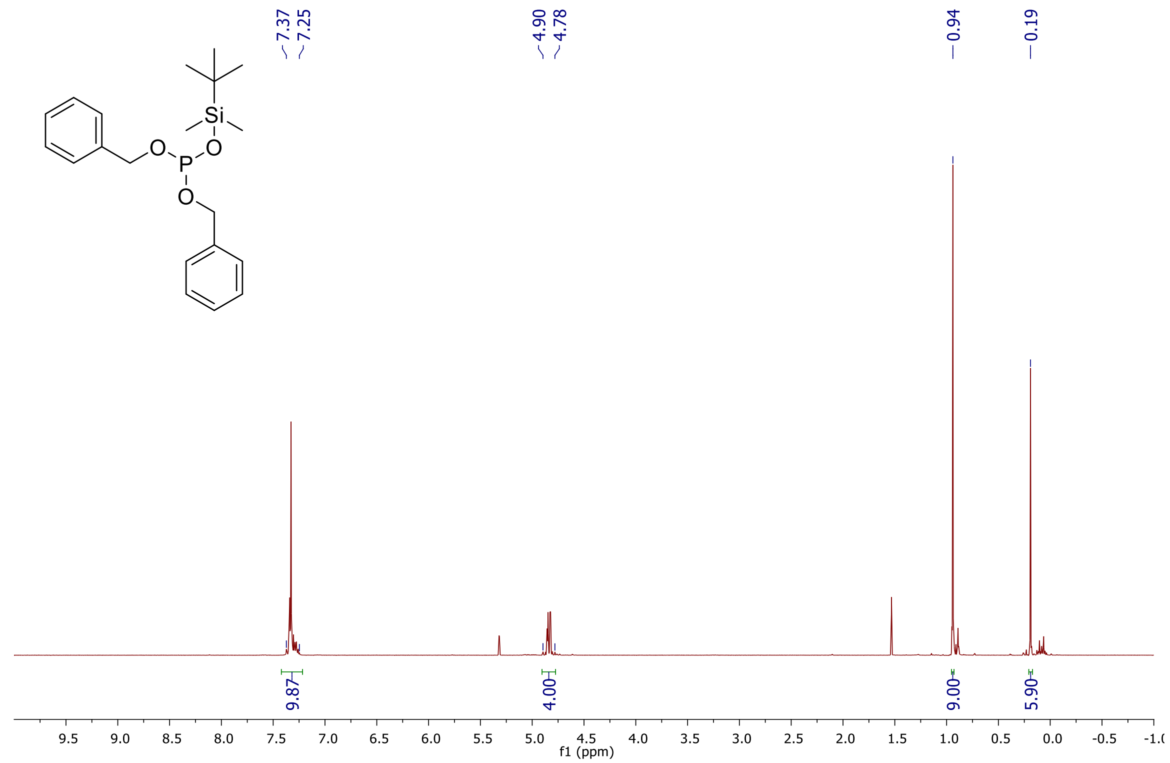

^{13}C-NMR (CD_2Cl_2, 75.5 MHz) of **2E**

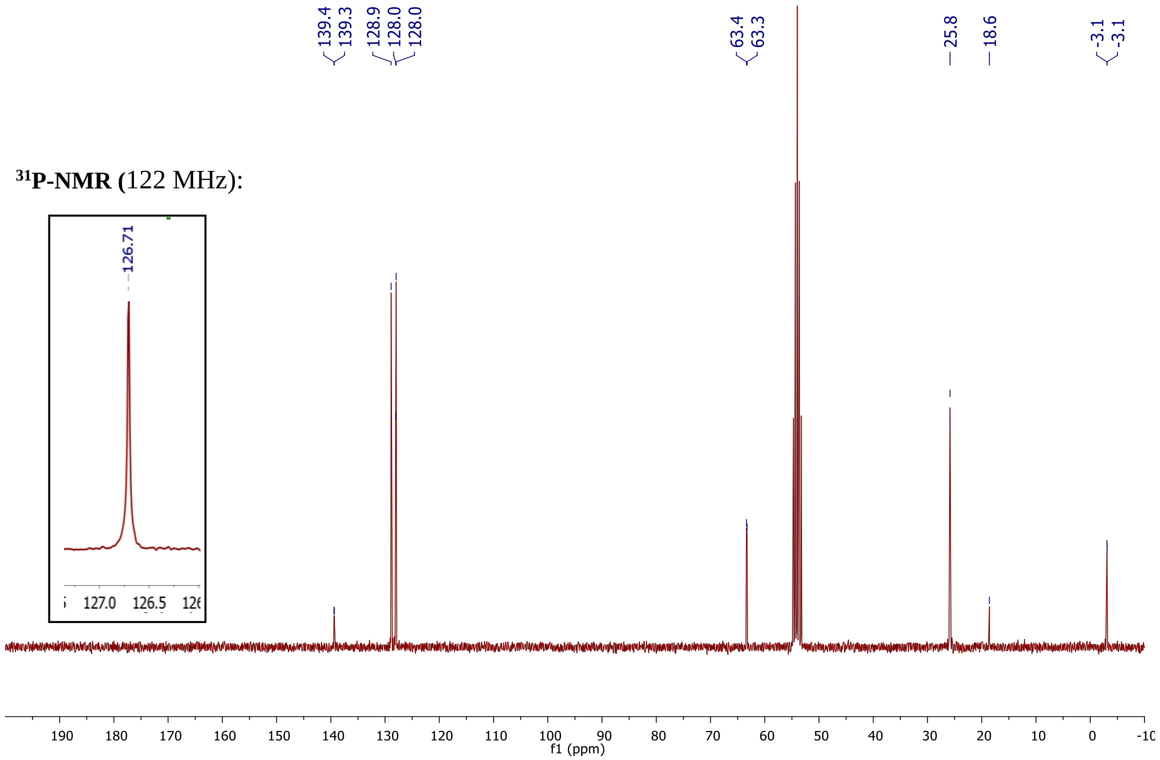

^{1}H-NMR (CD_2Cl_2, 300 MHz) of **2F**

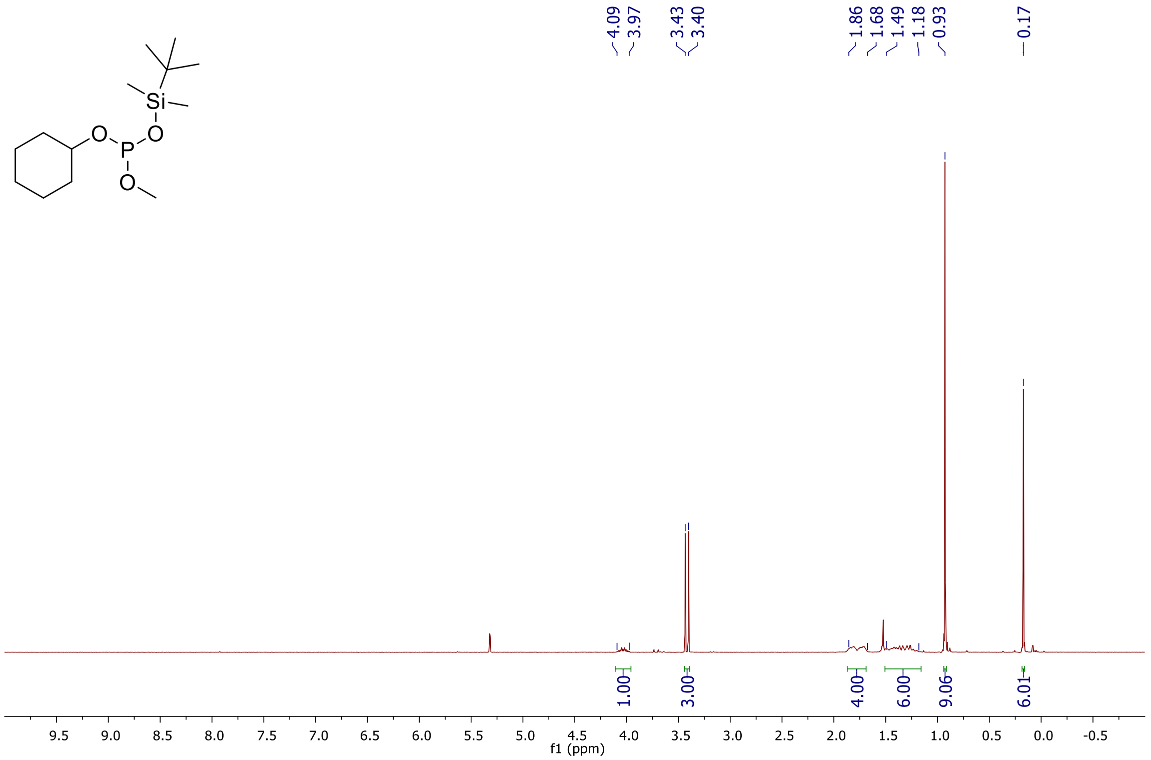

^{13}C-NMR (CD_2Cl_2, 75.5 MHz) of **2F**

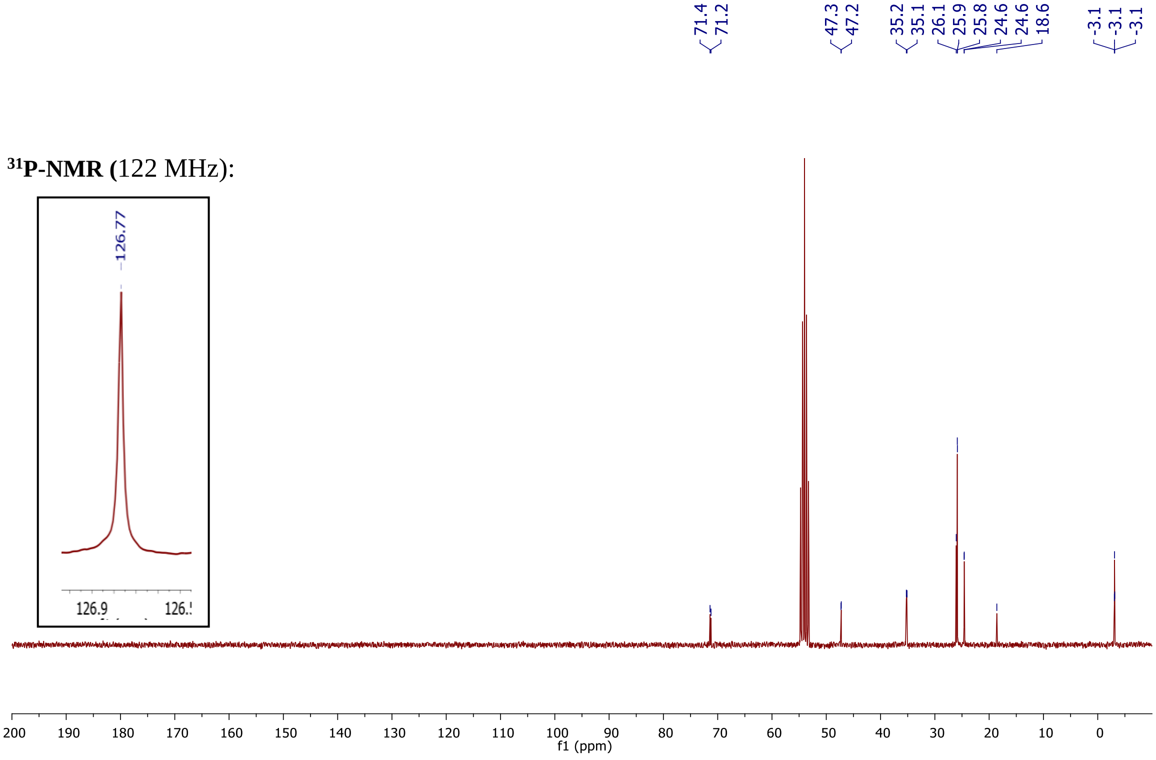

^{1}H-NMR (CDCl$_3$, 300 MHz) of (*S*)-**3aA**

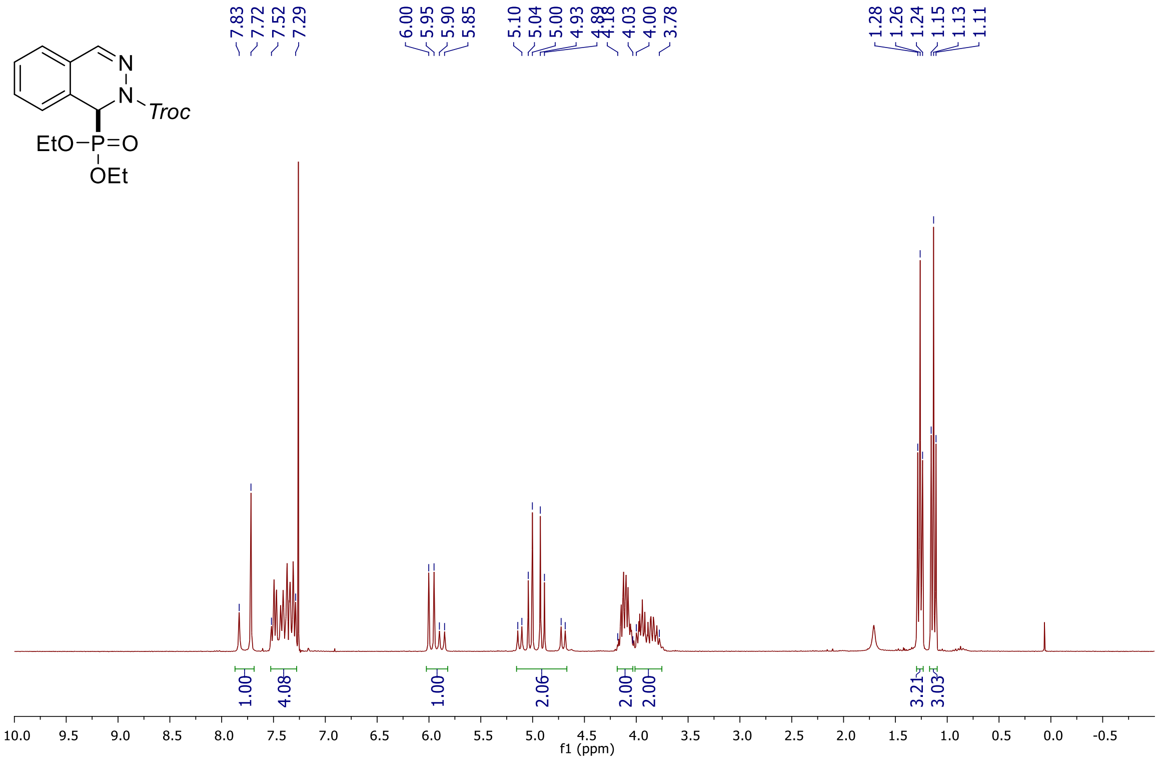

^{13}C-NMR (CDCl$_3$, 75.5 MHz) of (*S*)-**3aA**

^{31}P-NMR (122 MHz):

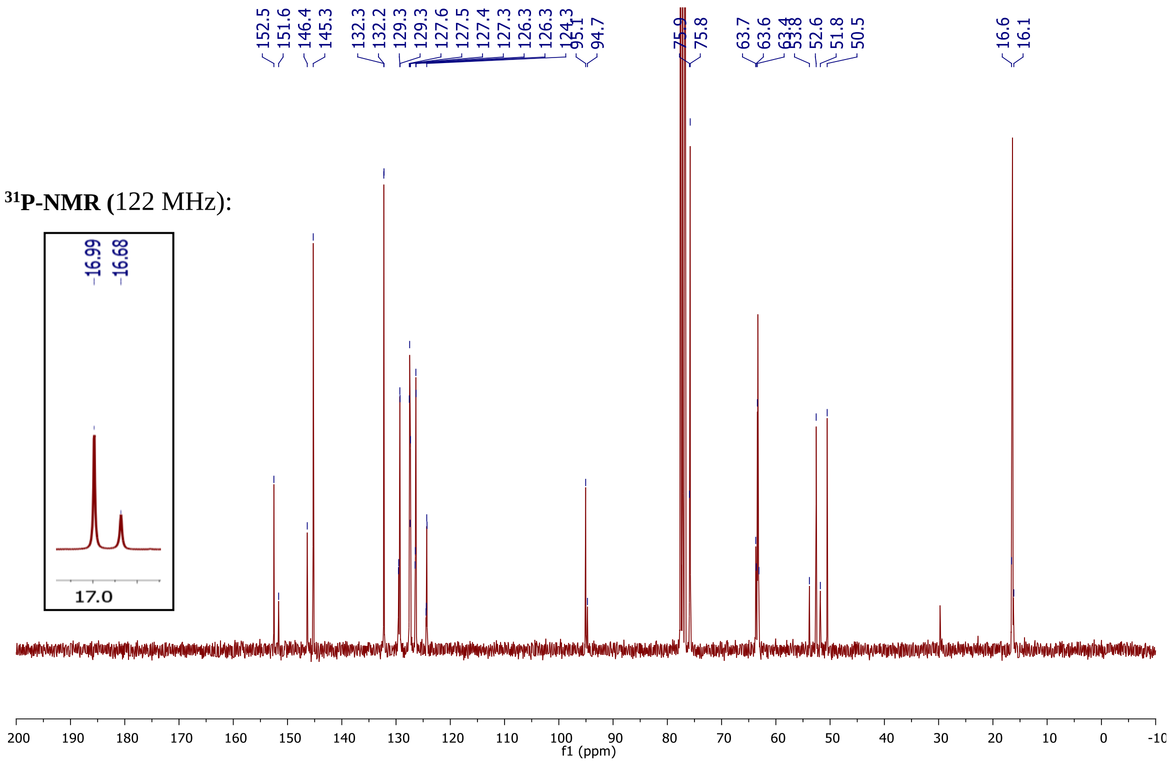

^{1}H-NMR ($CDCl_3$, 300 MHz) of (*S*)-**4aA**

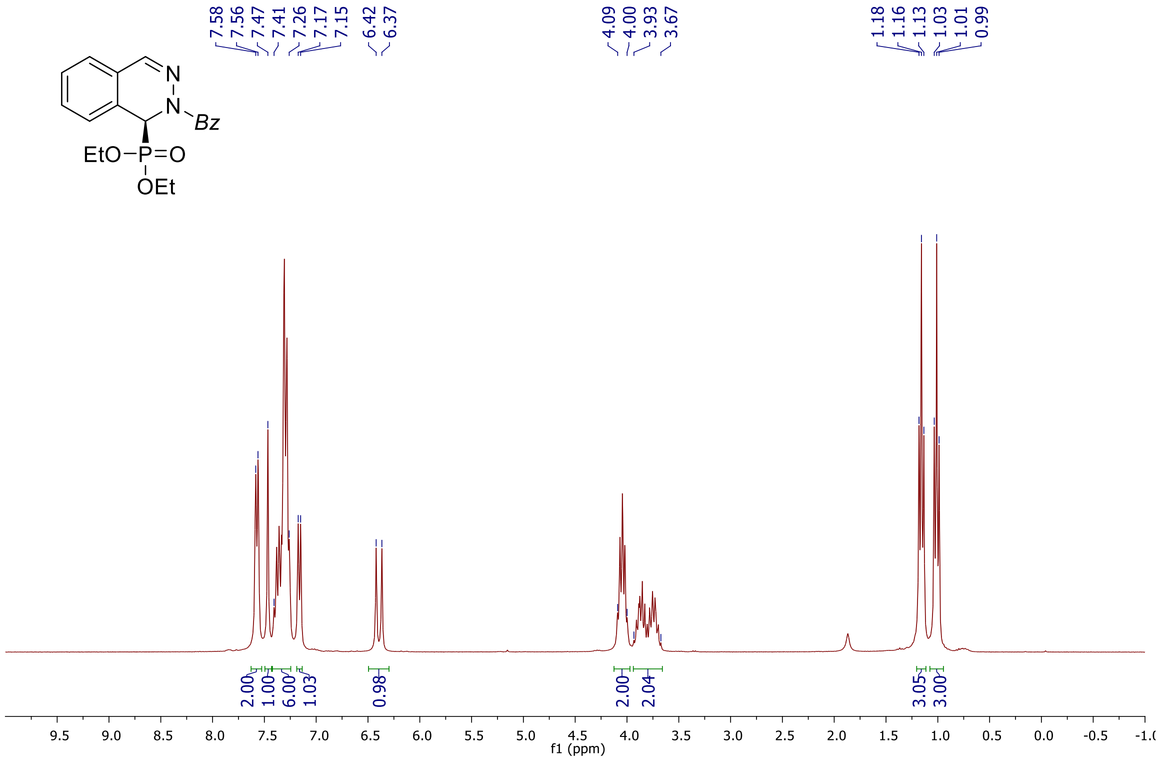

^{13}C-NMR ($CDCl_3$, 75.5 MHz) of (*S*)-**4aA**

^{31}P-NMR (122 MHz):

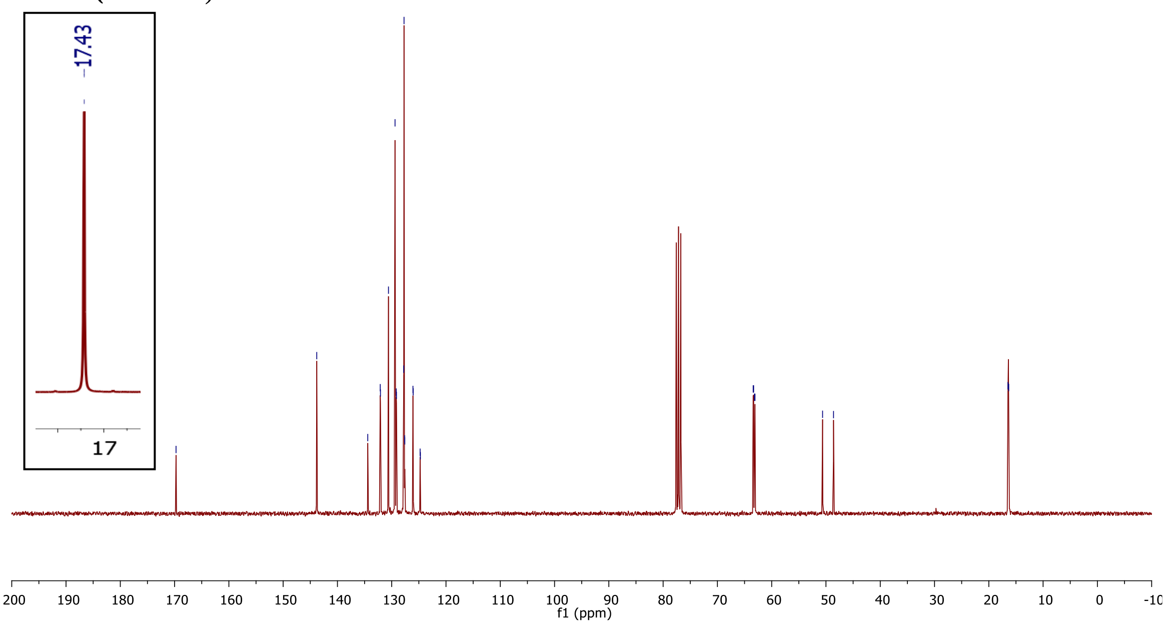

^{1}H-NMR (CDCl$_3$, 300 MHz) of (*S*)-**5aA**

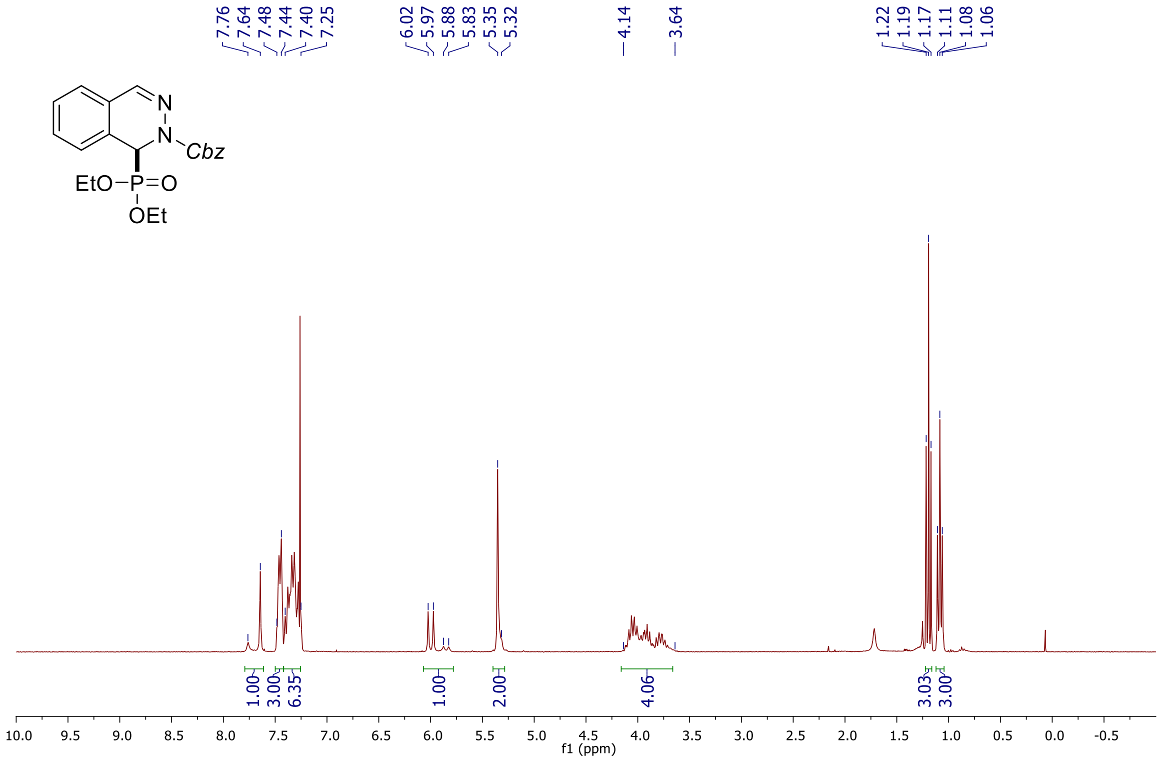

^{13}C-NMR (CDCl$_3$, 75.5 MHz) of (*S*)-**5aA**

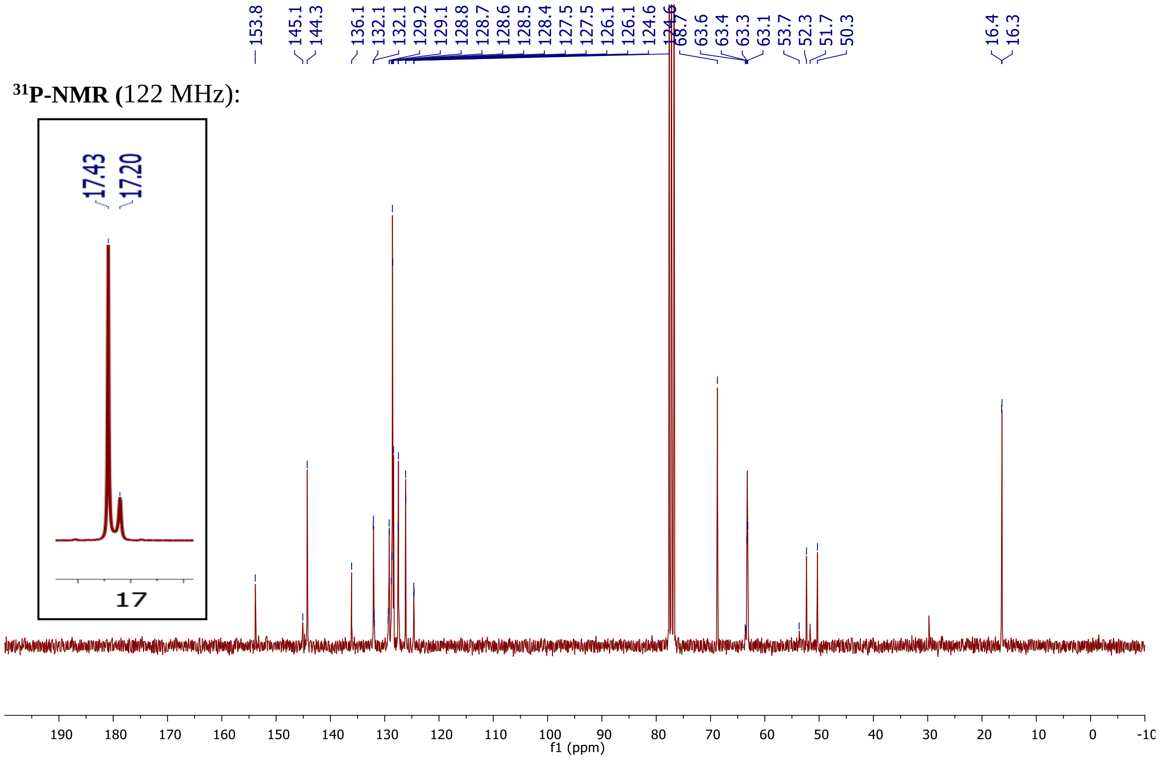

^{1}H-NMR (CDCl$_3$, 300 MHz) of (*S*)-**6aA**

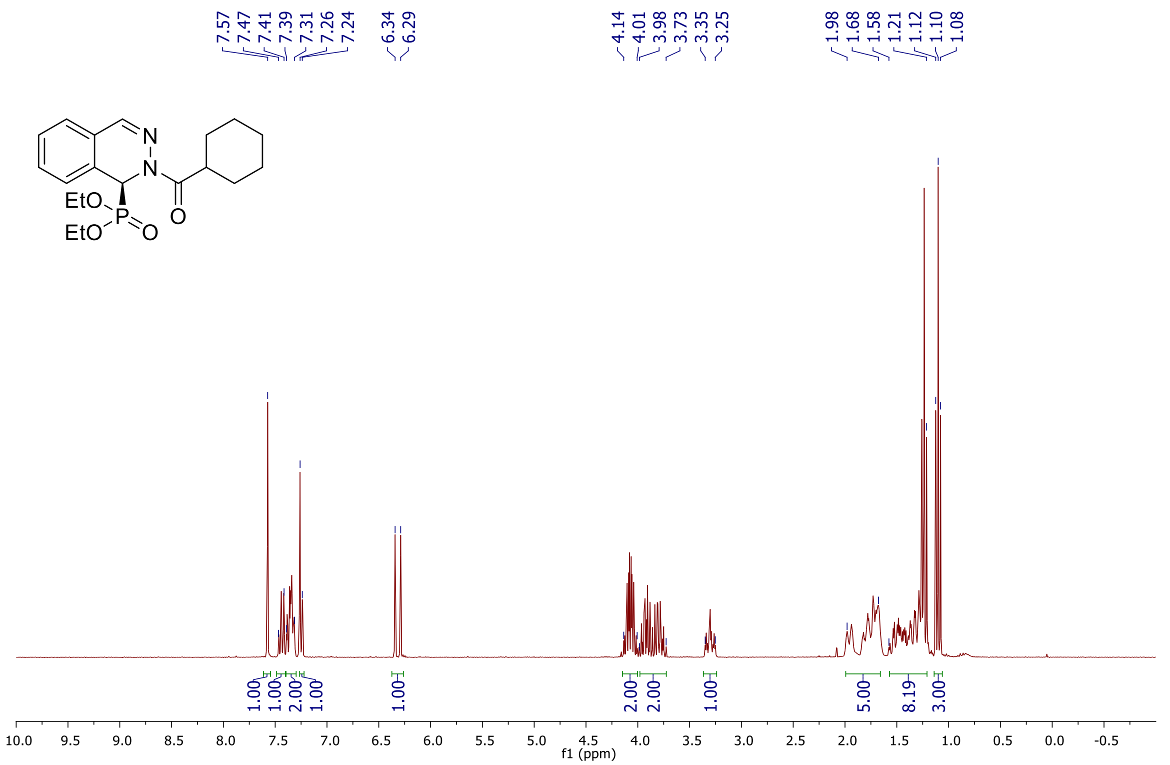

^{13}C-NMR (CDCl$_3$, 75.5 MHz) of (*S*)-**6aA**

^{31}P-NMR (122 MHz):

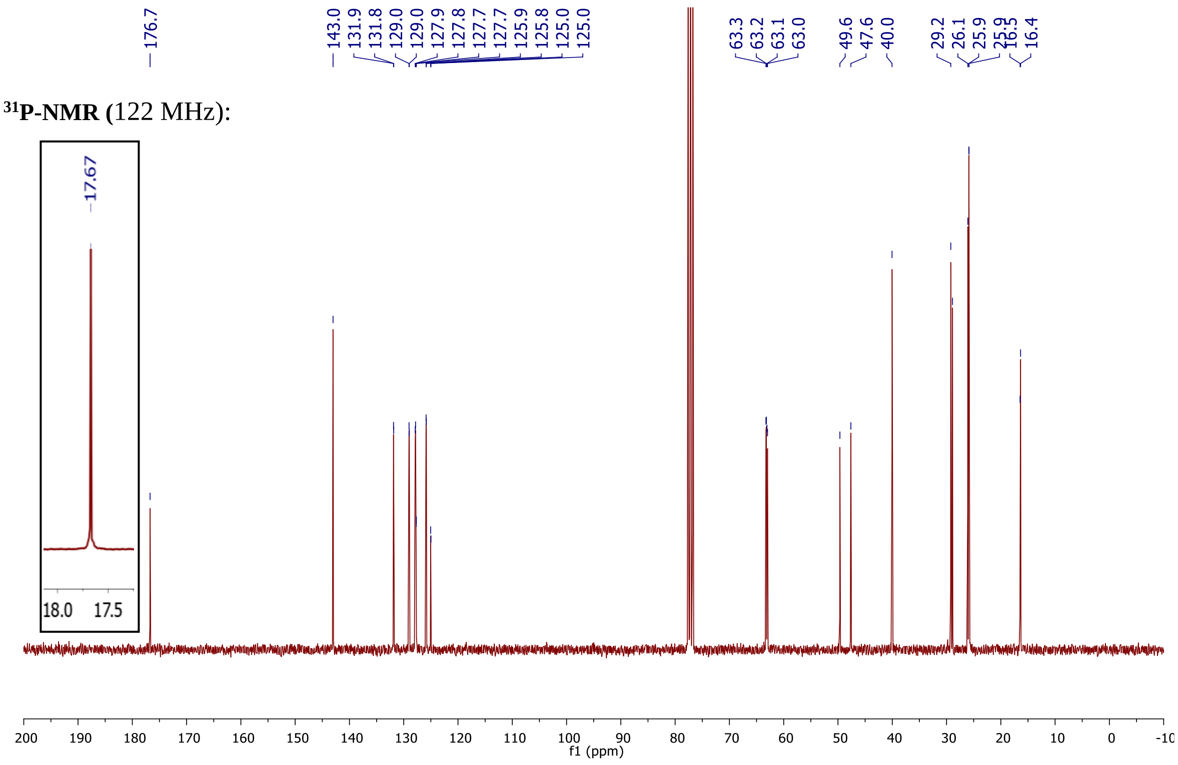

^{1}H-NMR (CDCl$_3$, 300 MHz) of (*S*)-**7aA**

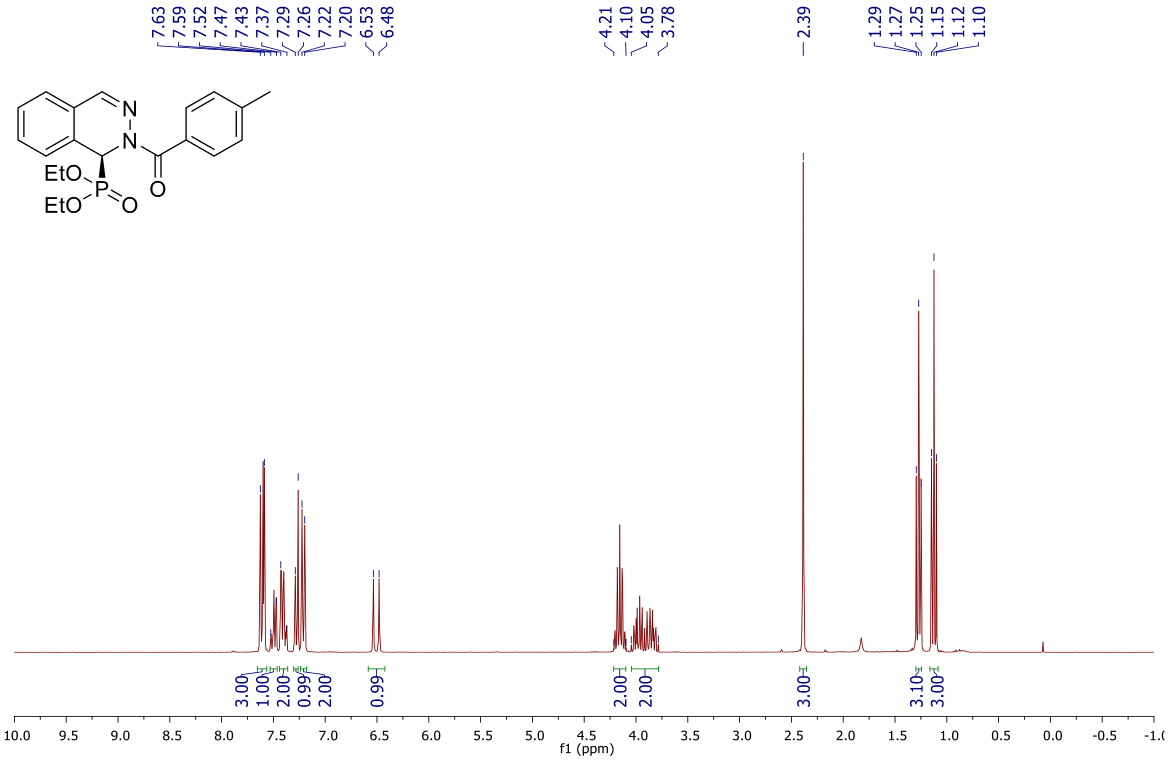

^{13}C-NMR (CDCl$_3$, 75.5 MHz) of (*S*)-**7aA**

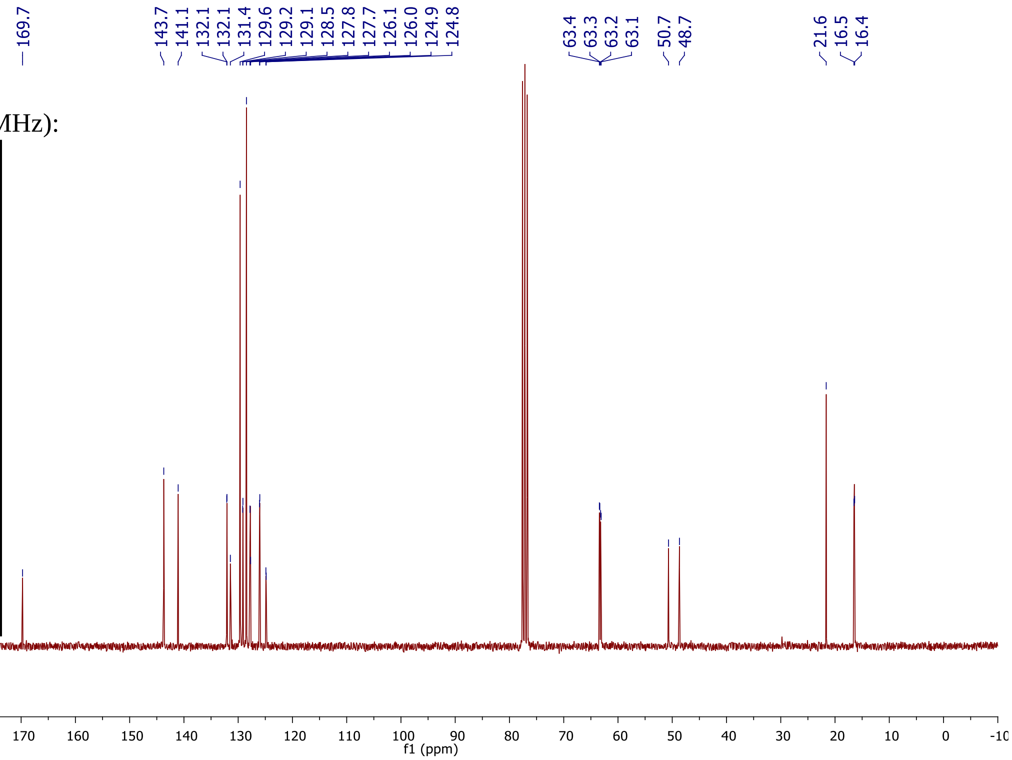

^{31}P-NMR (122 MHz):

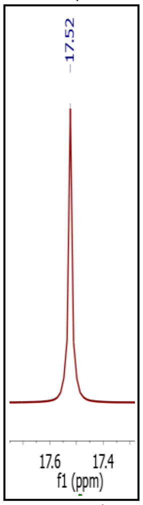

[1]**H-NMR** (CDCl$_3$, 300 MHz) of (*S*)-**8aA**

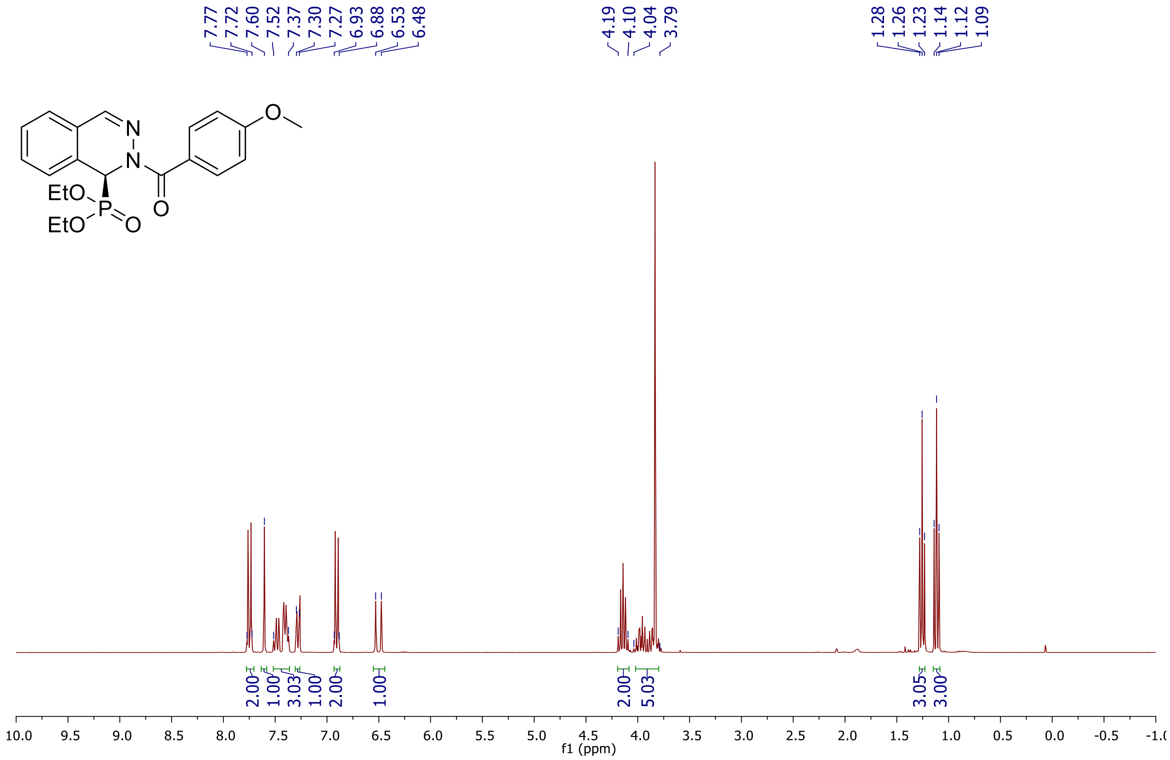

[13]**C-NMR** (CDCl$_3$, 75.5 MHz) of (*S*)-**8aA**

[31]**P-NMR (**122 MHz):

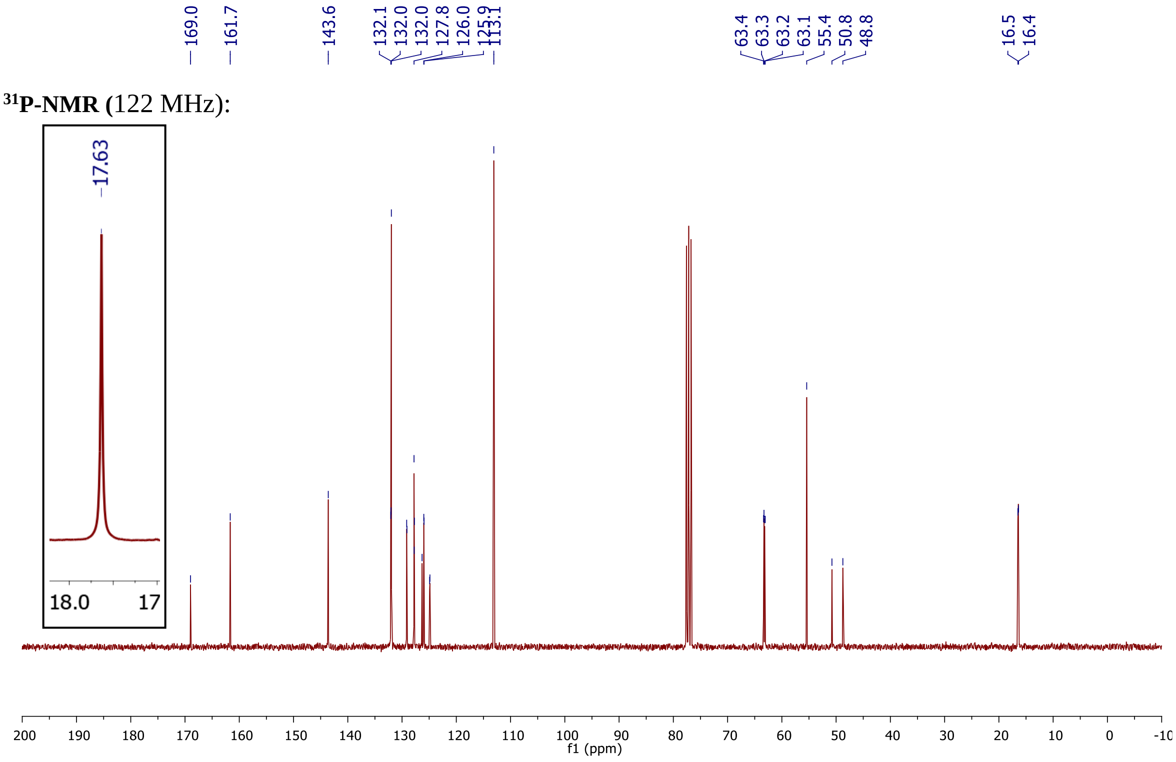

^{1}H-NMR (CDCl$_3$, 300 MHz) of (*S*)-**9aA**

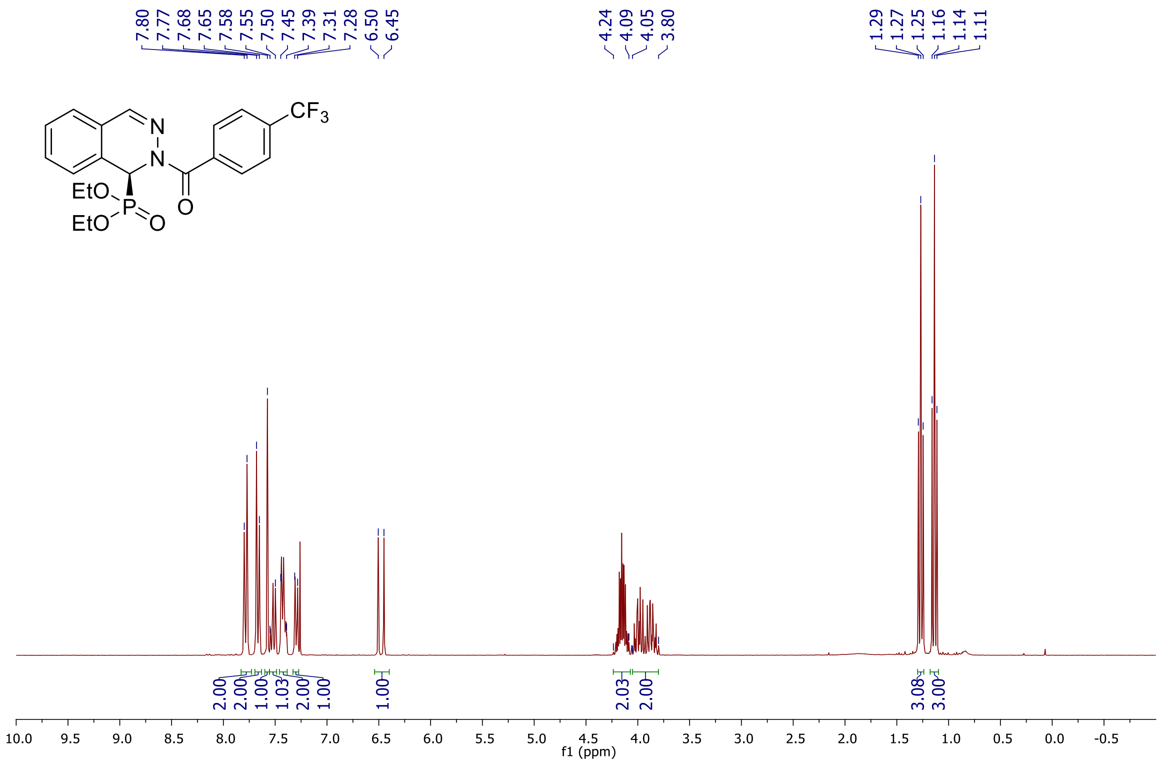

^{13}C-NMR (CDCl$_3$, 75.5 MHz) of (*S*)-**9aA**

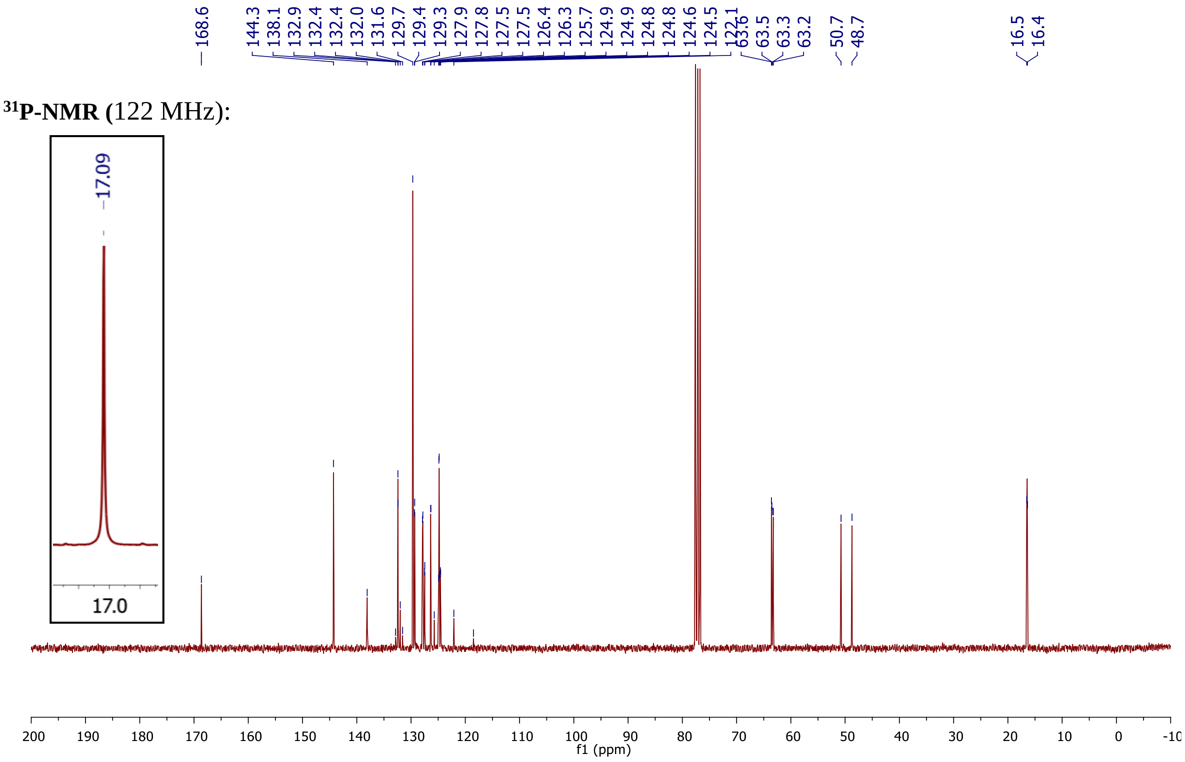

^{1}H-NMR (CDCl$_3$, 300 MHz) of (*S*)-**10aA**

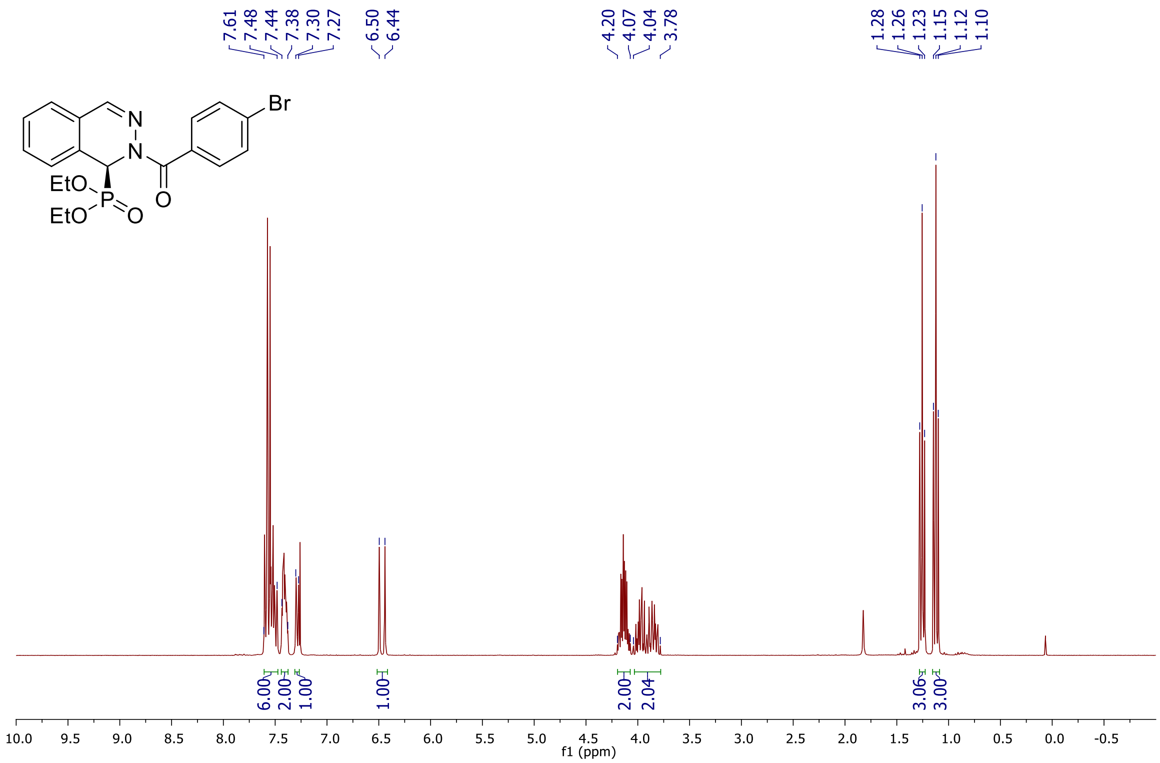

^{13}C-NMR (CDCl$_3$, 75.5 MHz) of (*S*)-**10aA**

^{31}P-NMR (122 MHz):

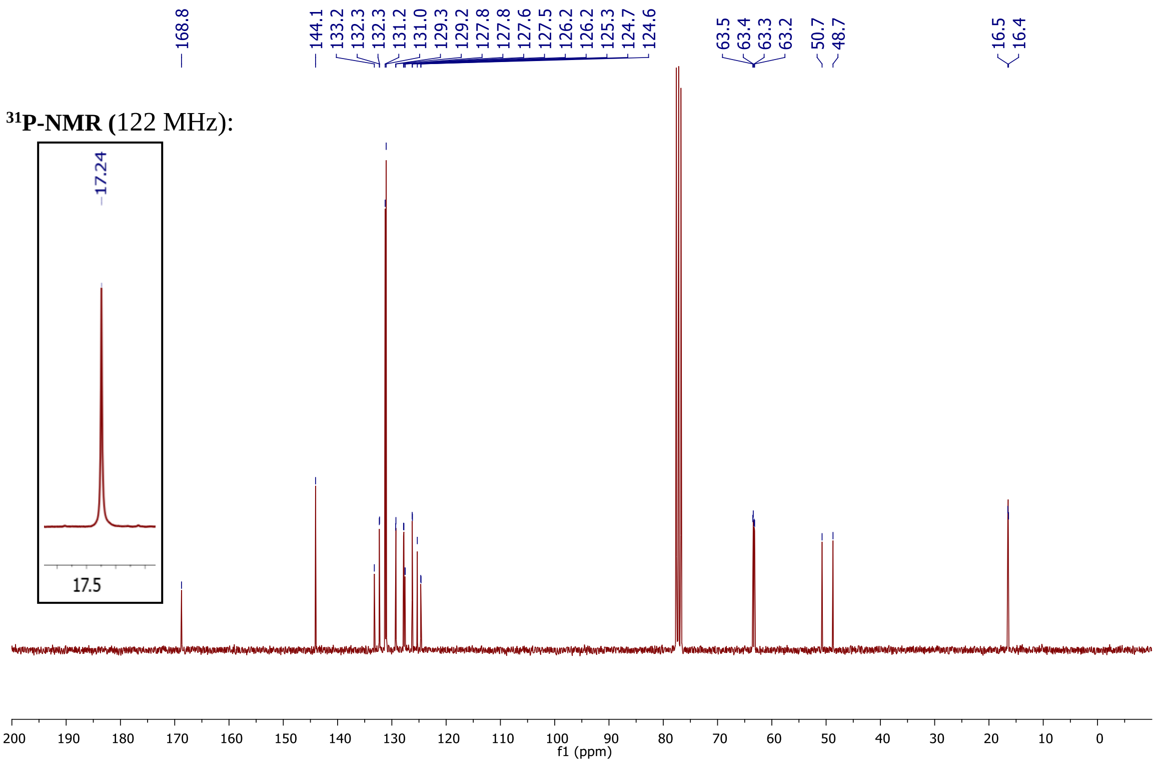

^{1}H-NMR (CDCl$_3$, 300 MHz) of (*S*)-**11aA**

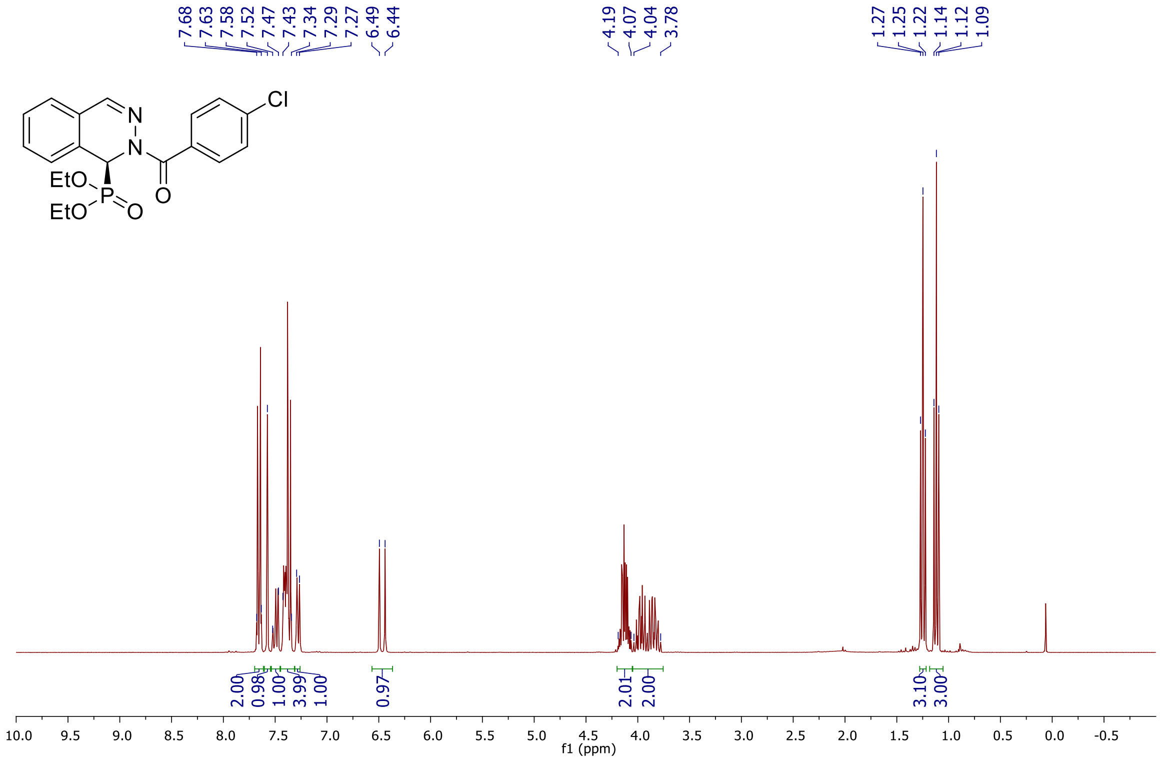

^{13}C-NMR (CDCl$_3$, 75.5 MHz) of (*S*)-**11aA**

^{31}P-NMR (122 MHz):

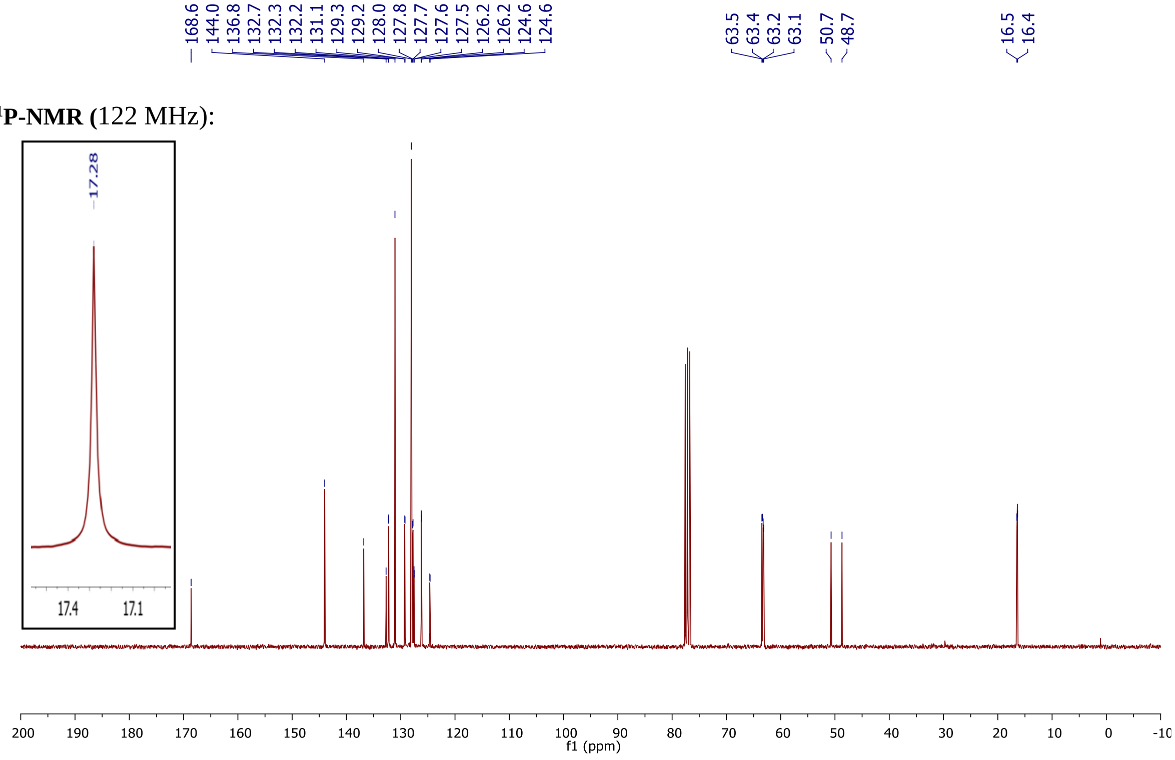

^{1}H-NMR (CDCl$_3$, 300 MHz) of (*S*)-**12aA**

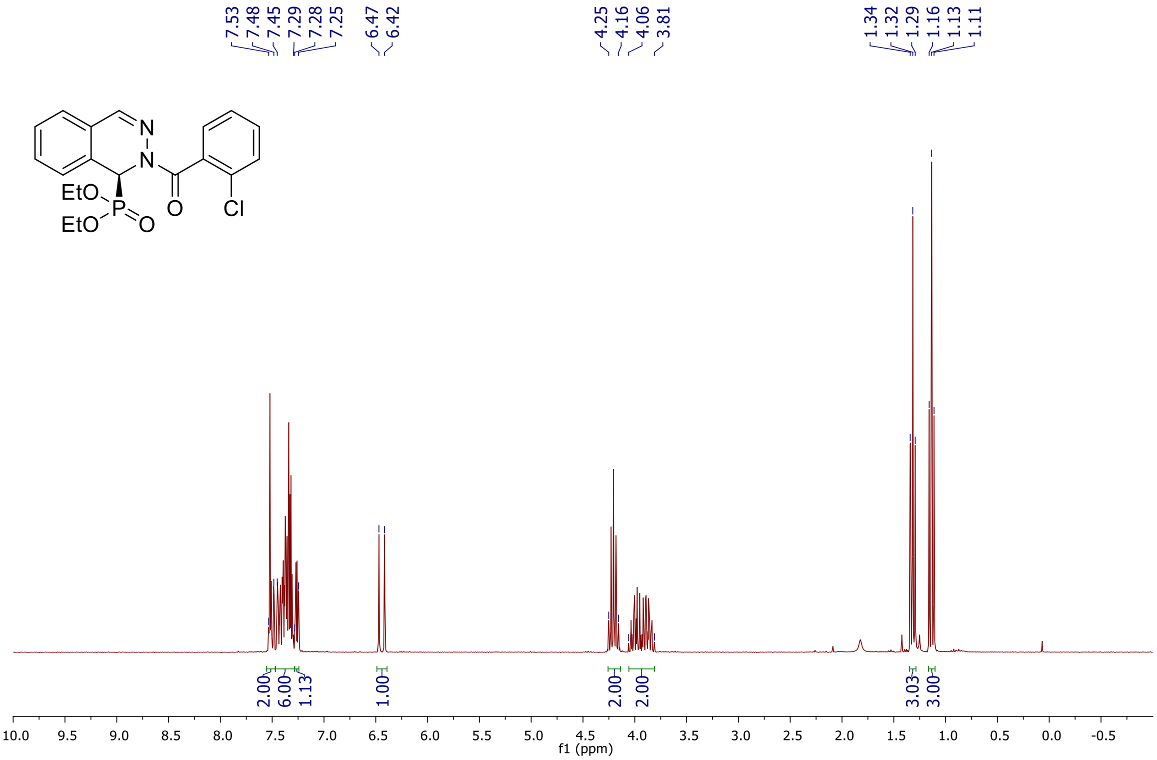

^{13}C-NMR (CDCl$_3$, 75.5 MHz) of (*S*)-**12aA**

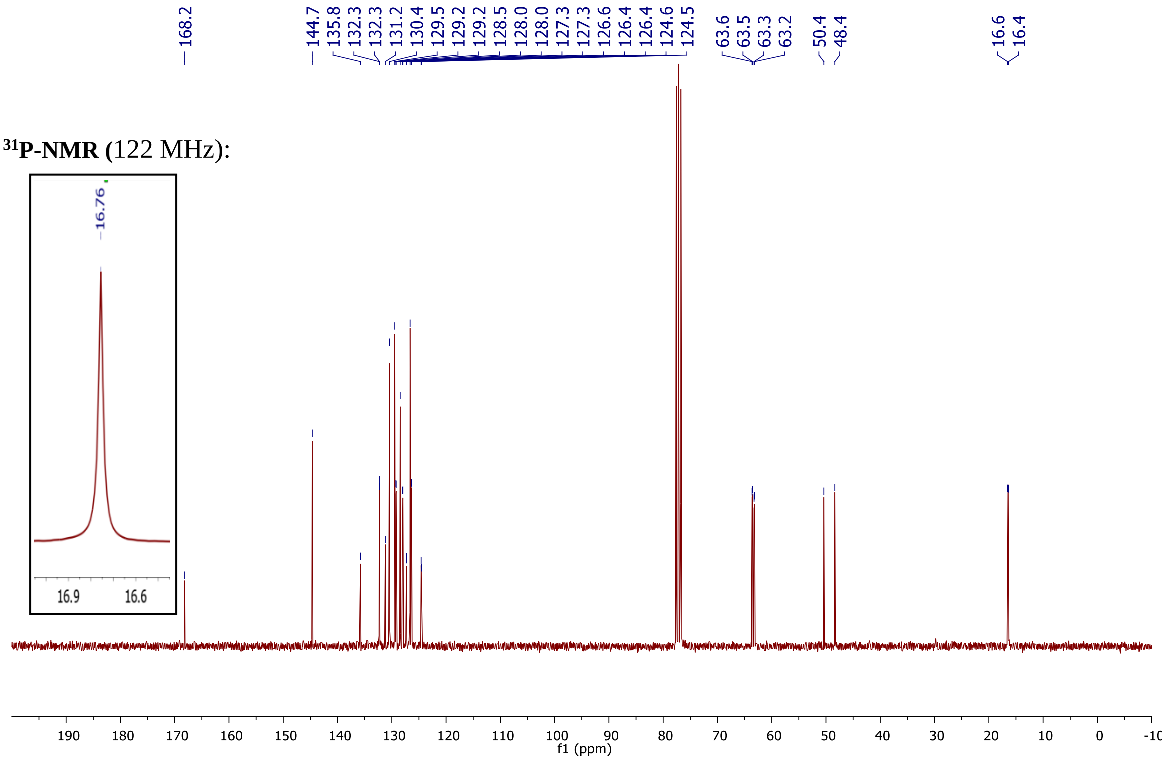

^{1}H-NMR ($CDCl_3$, 300 MHz) of (*S*)-**13aA**

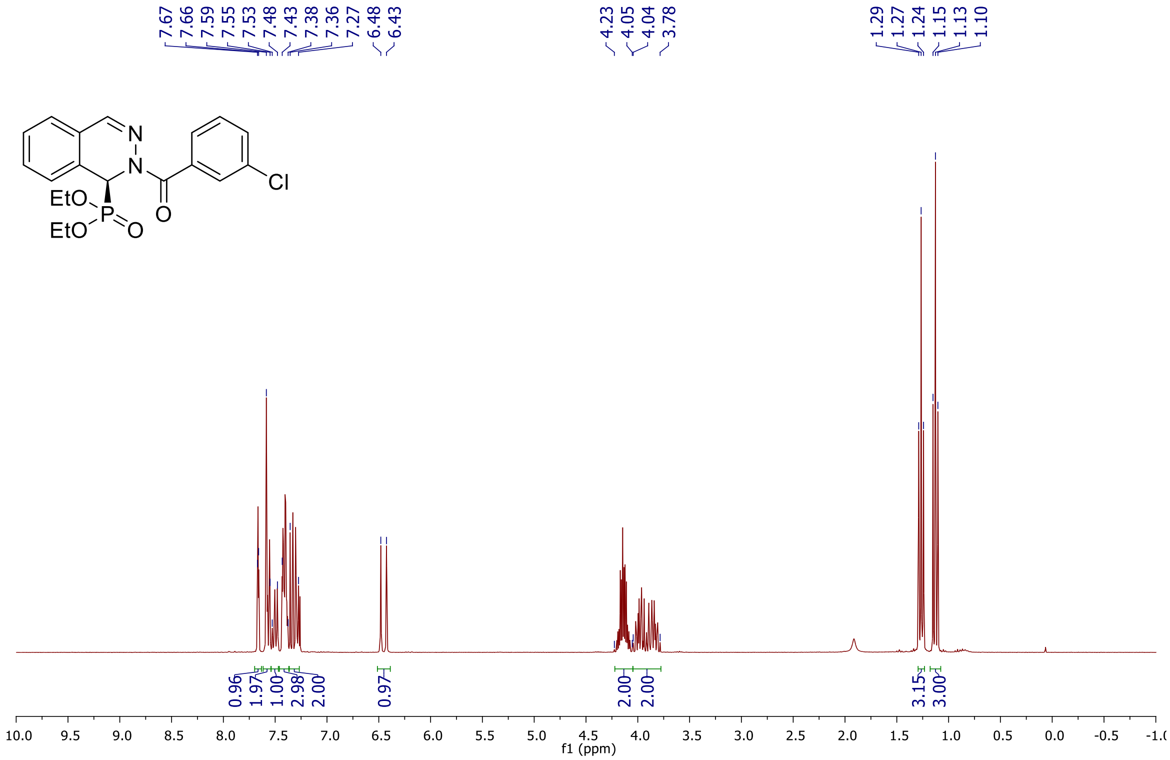

^{13}C-NMR ($CDCl_3$, 75.5 MHz) of (*S*)-**13aA**

^{31}P-NMR (122 MHz):

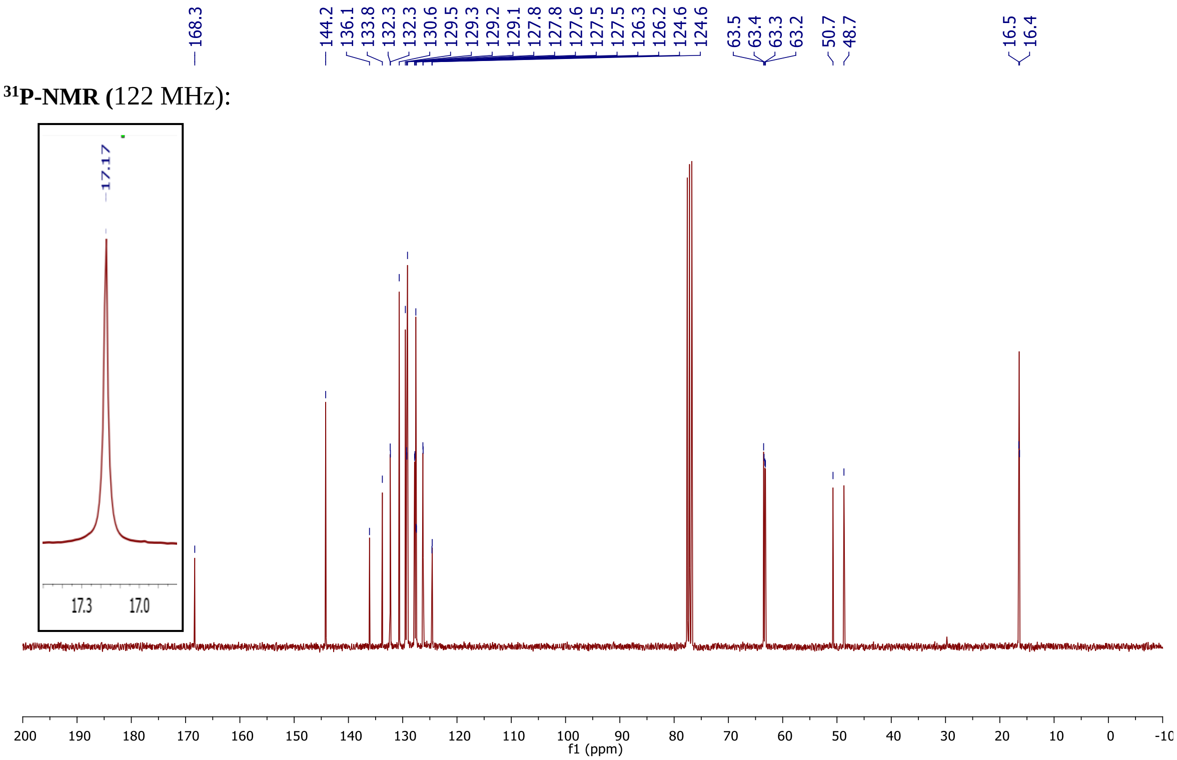

^{1}H-NMR (CDCl$_3$, 300 MHz) of (*S*)-**14aA**

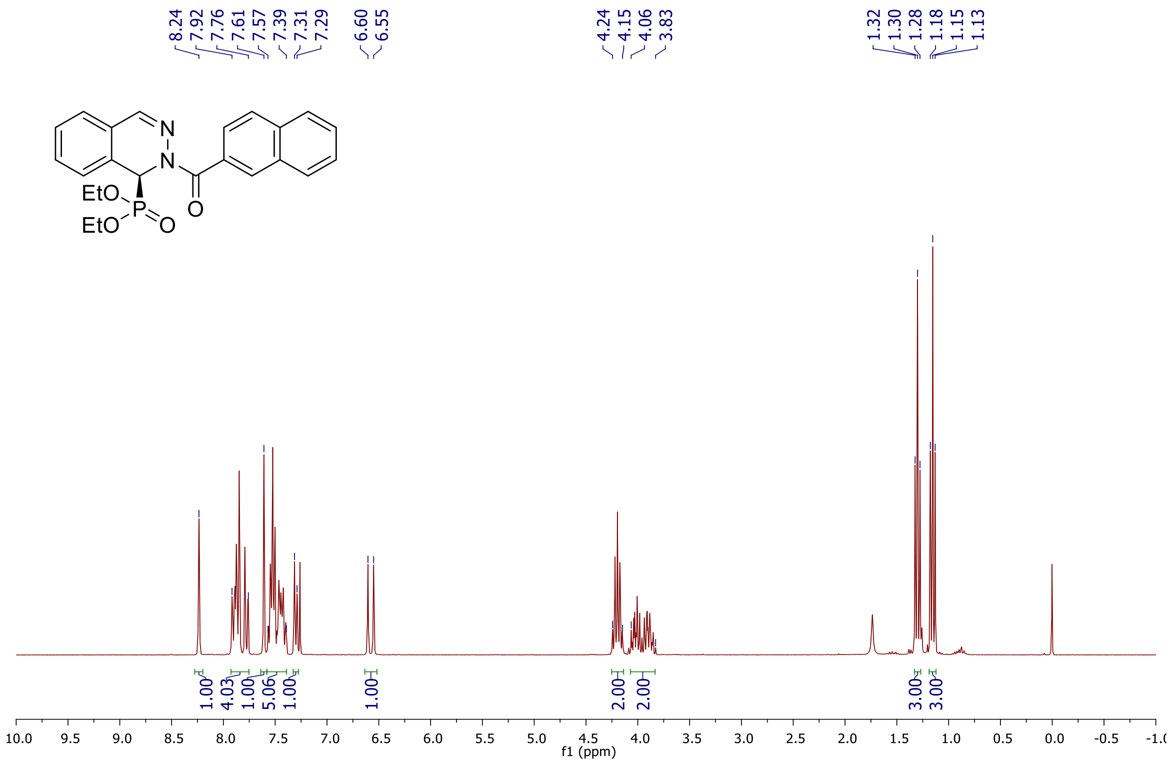

^{13}C-NMR (CDCl$_3$, 75.5 MHz) of (*S*)-**14aA**

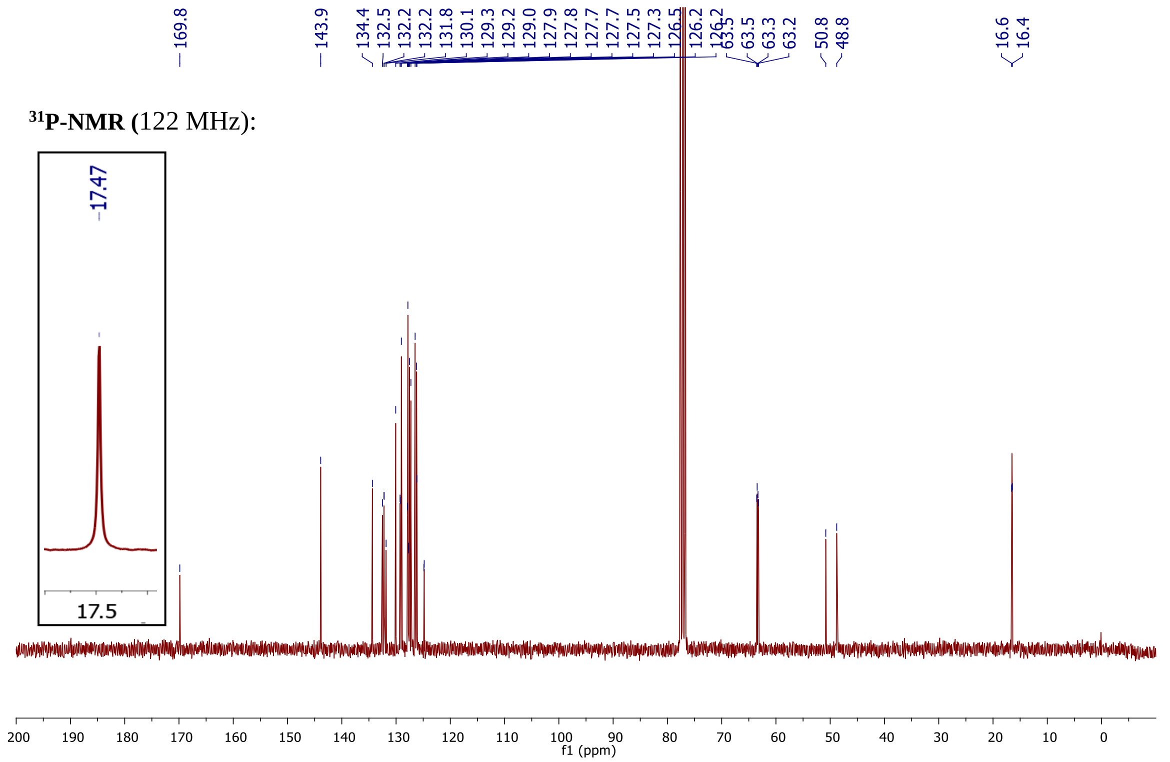

^{1}H-NMR (CDCl$_3$, 300 MHz) of (*S*)-**15aA**

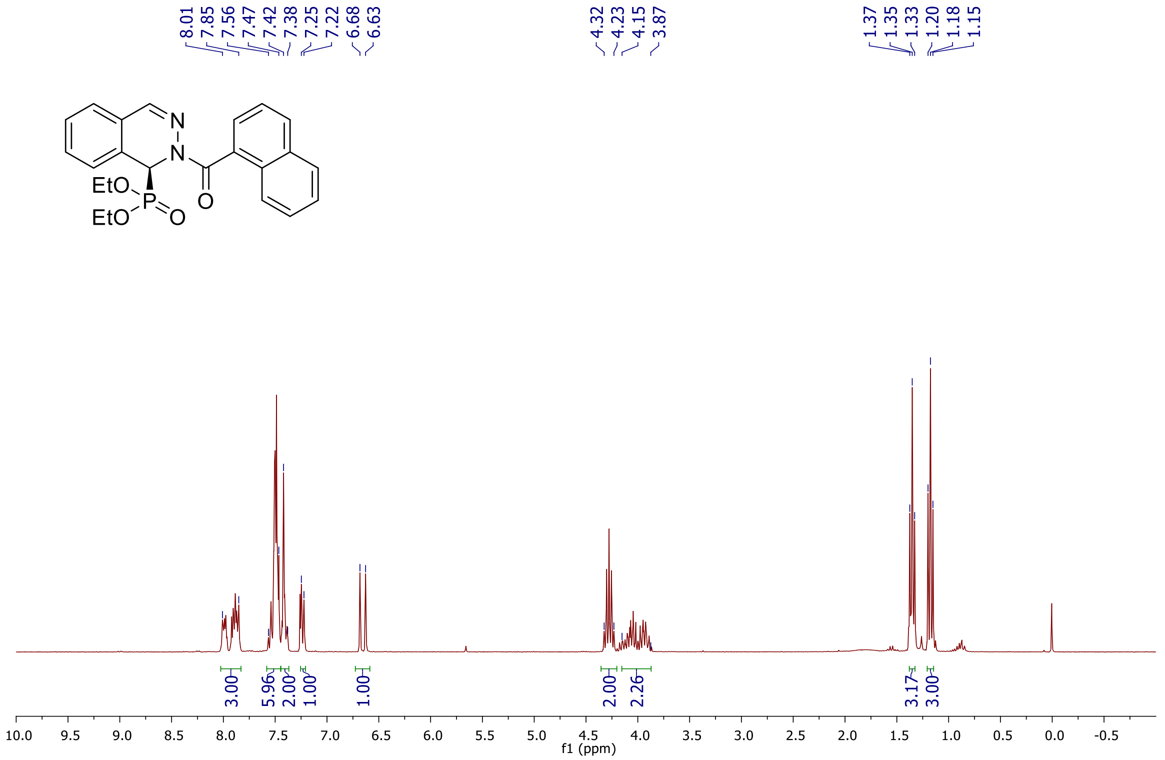

^{13}C-NMR (CDCl$_3$, 75.5 MHz) of (*S*)-**15aA**

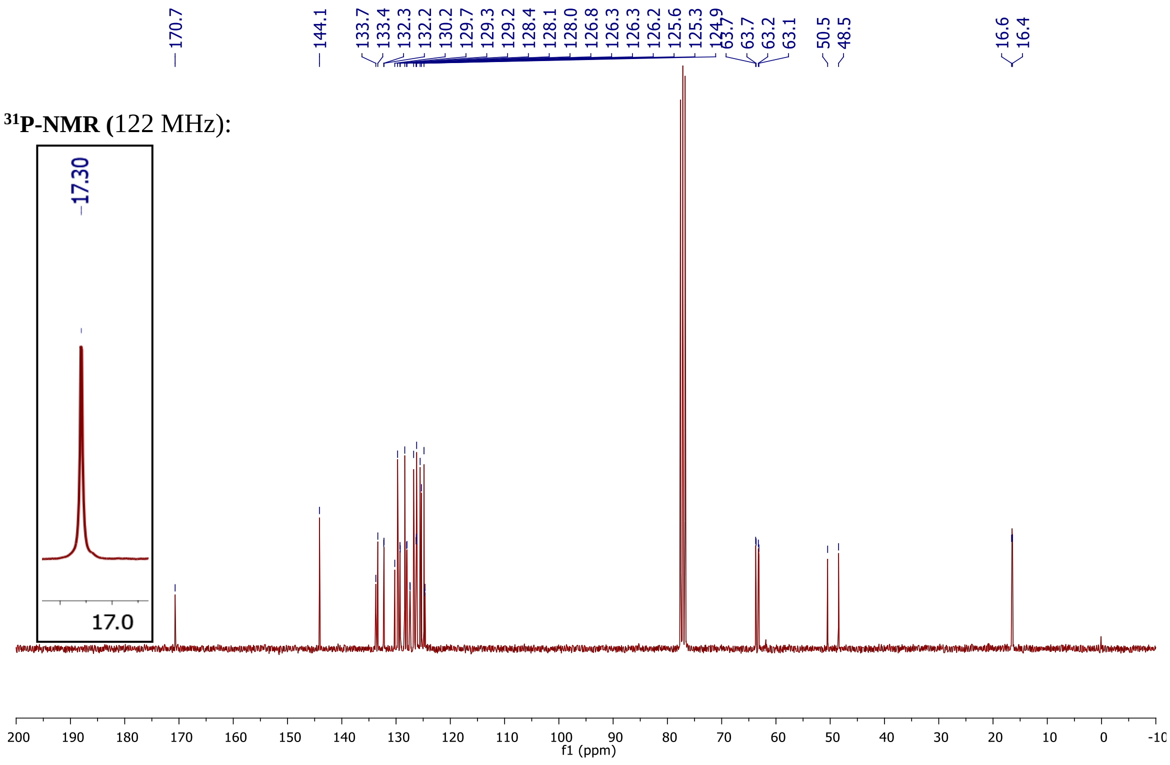

^{1}H-NMR (CDCl$_3$, 300 MHz) of (*S*)-**11aB**

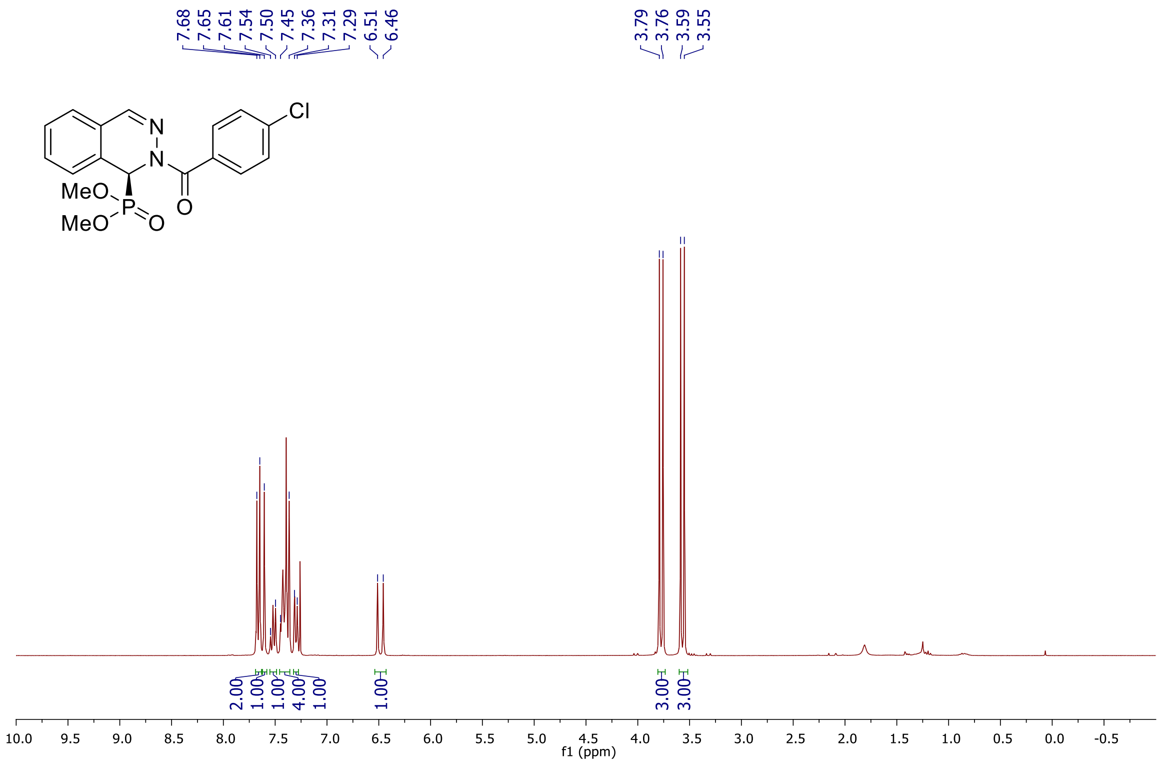

^{13}C-NMR (CDCl$_3$, 75.5 MHz) of (*S*)-**11aB**

^{31}P-NMR (122 MHz):

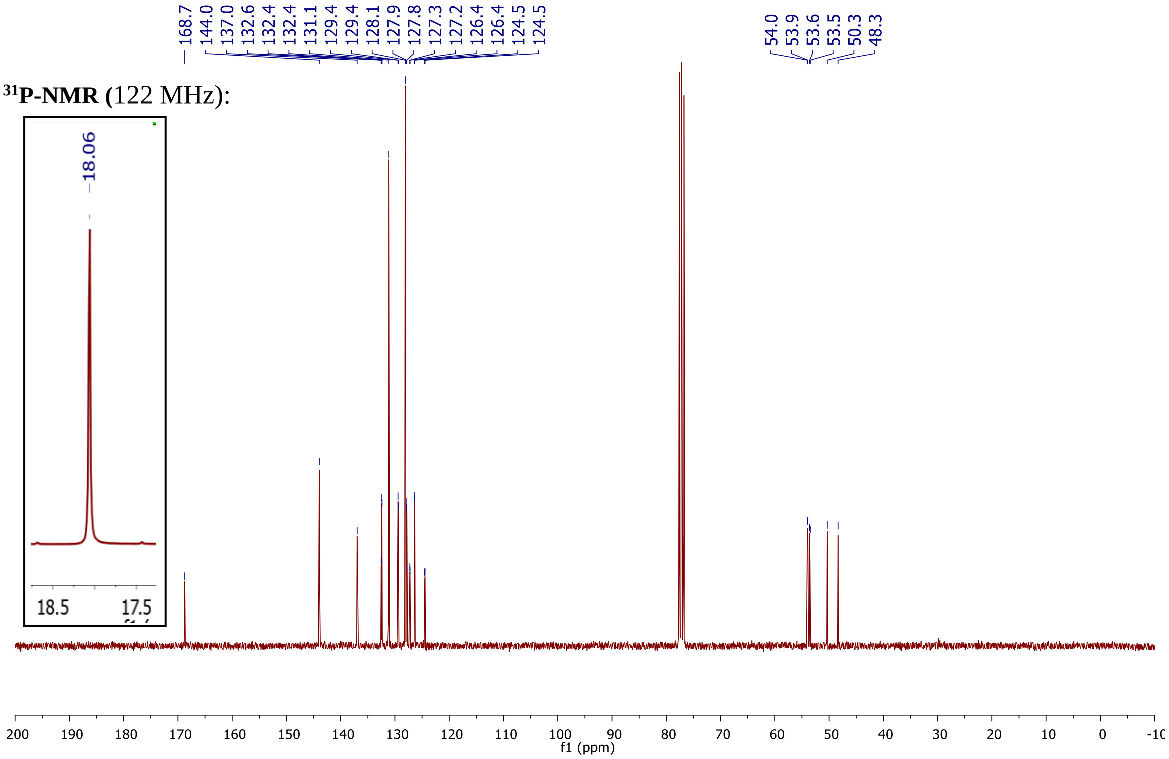

[1]**H-NMR** ($CDCl_3$, 300 MHz) of (*S*)-**11aC**

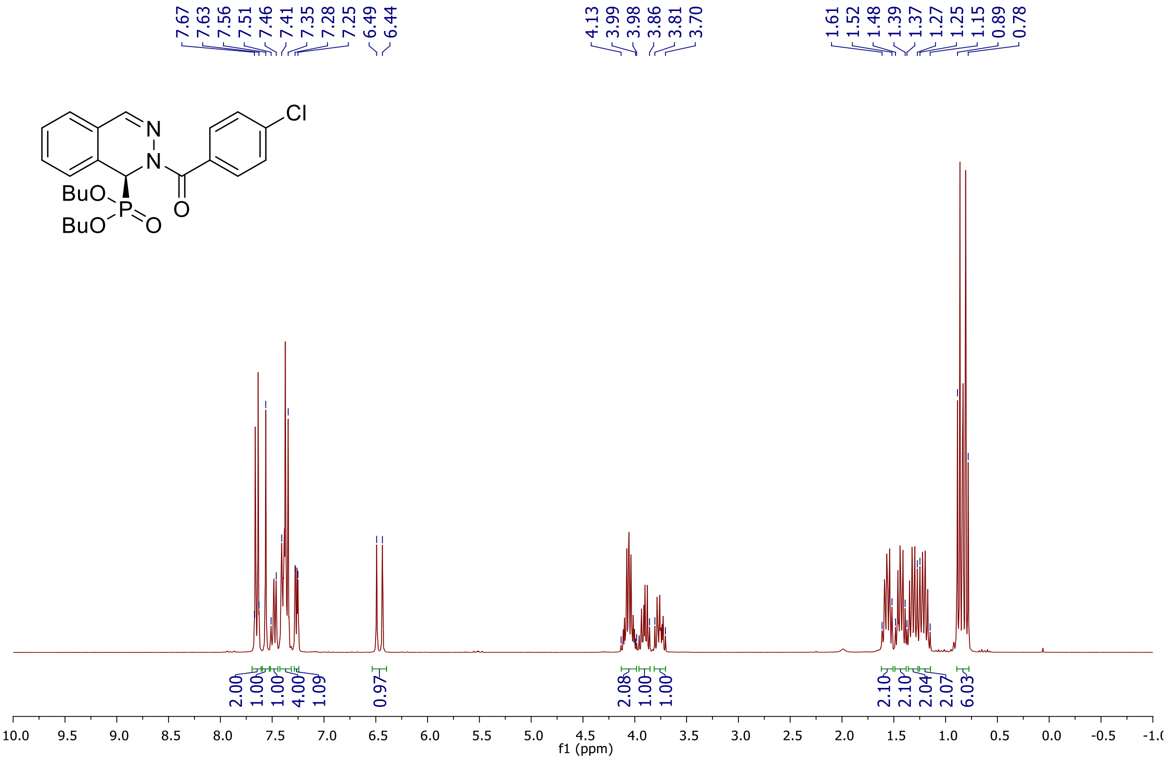

[13]**C-NMR** ($CDCl_3$, 75.5 MHz) of (*S*)-**11aC**

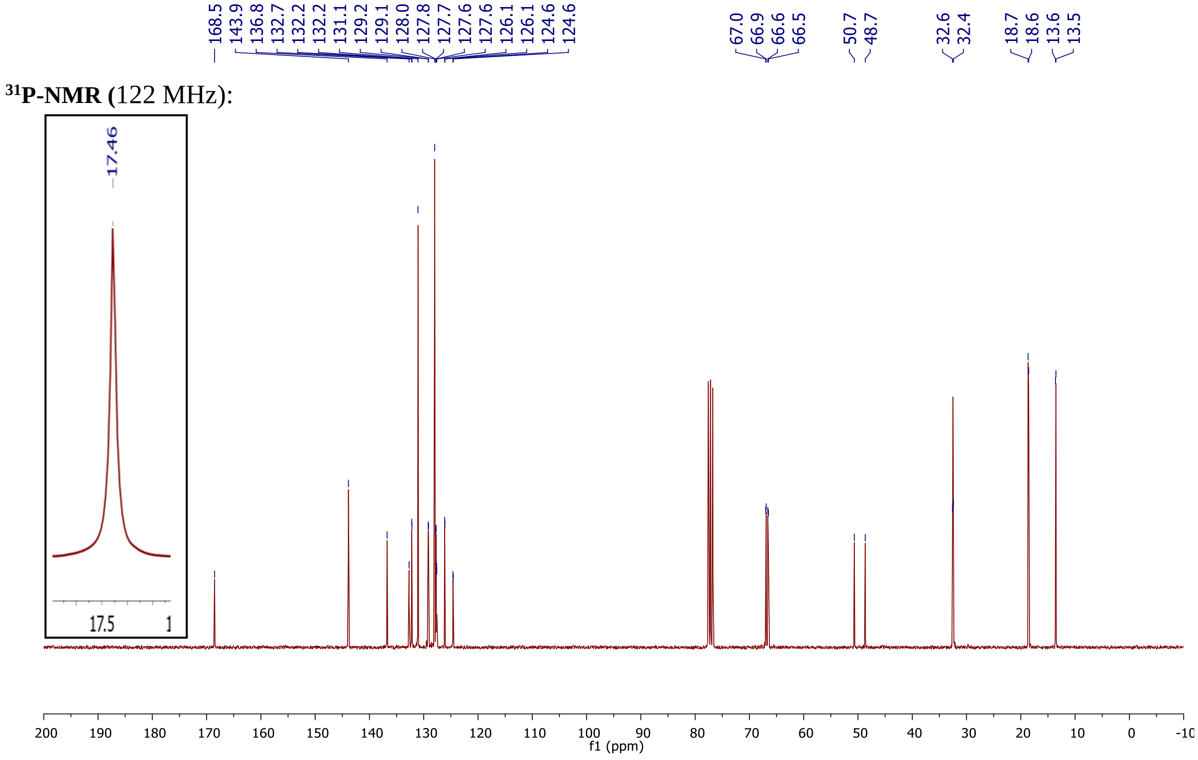

[1]**H-NMR** ($CDCl_3$, 300 MHz) of (*S*)-**11aD**

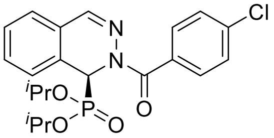

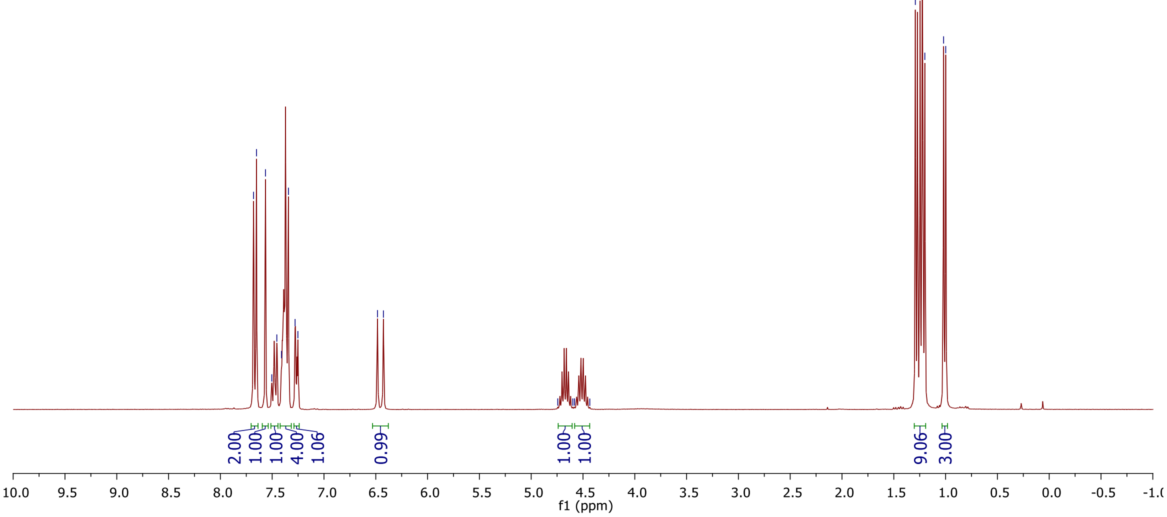

[13]**C-NMR** ($CDCl_3$, 75.5 MHz) of (*S*)-**11aD**

[31]**P-NMR (**122 MHz):

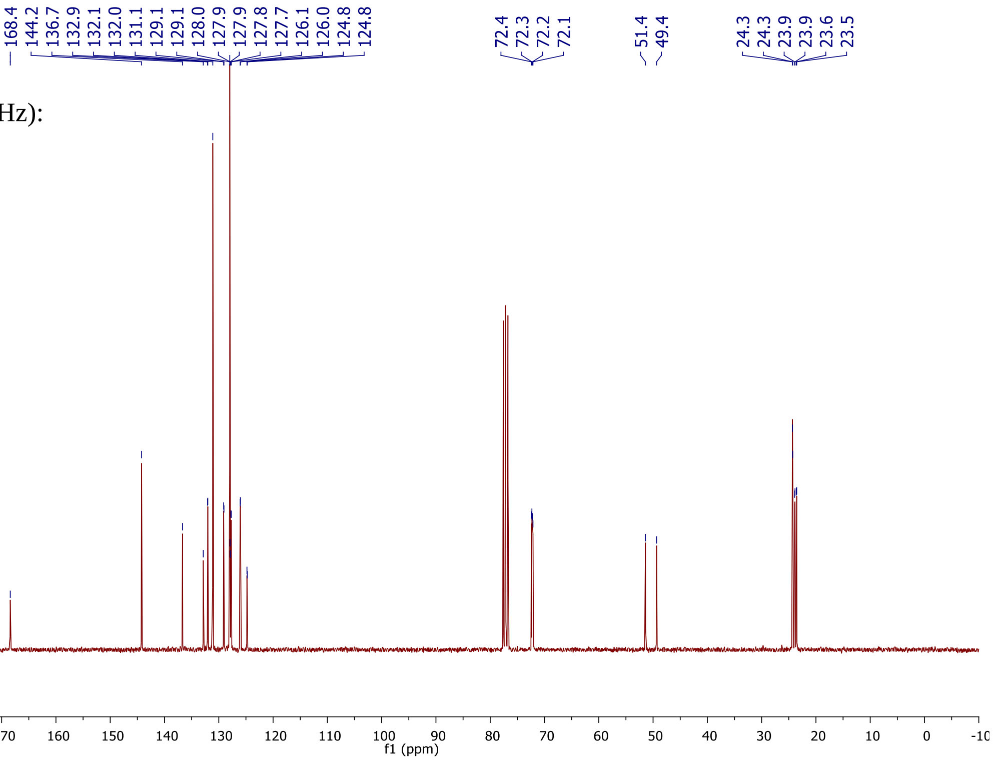

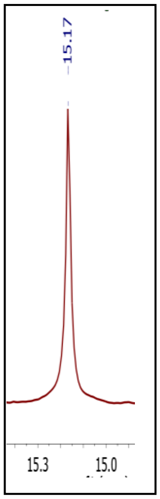

^{1}H-NMR (CDCl$_3$, 300 MHz) of (*S*)-**11aE**

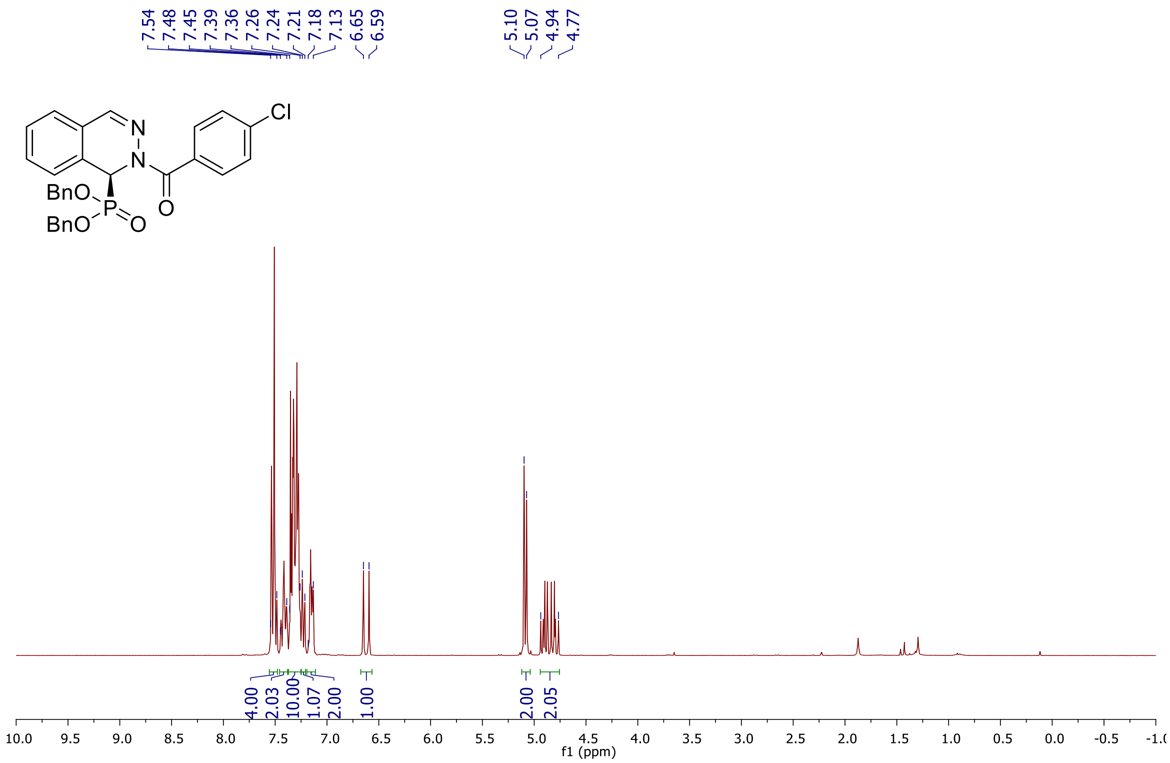

^{13}C-NMR (CDCl$_3$, 75.5 MHz) of (*S*)-**11aE**

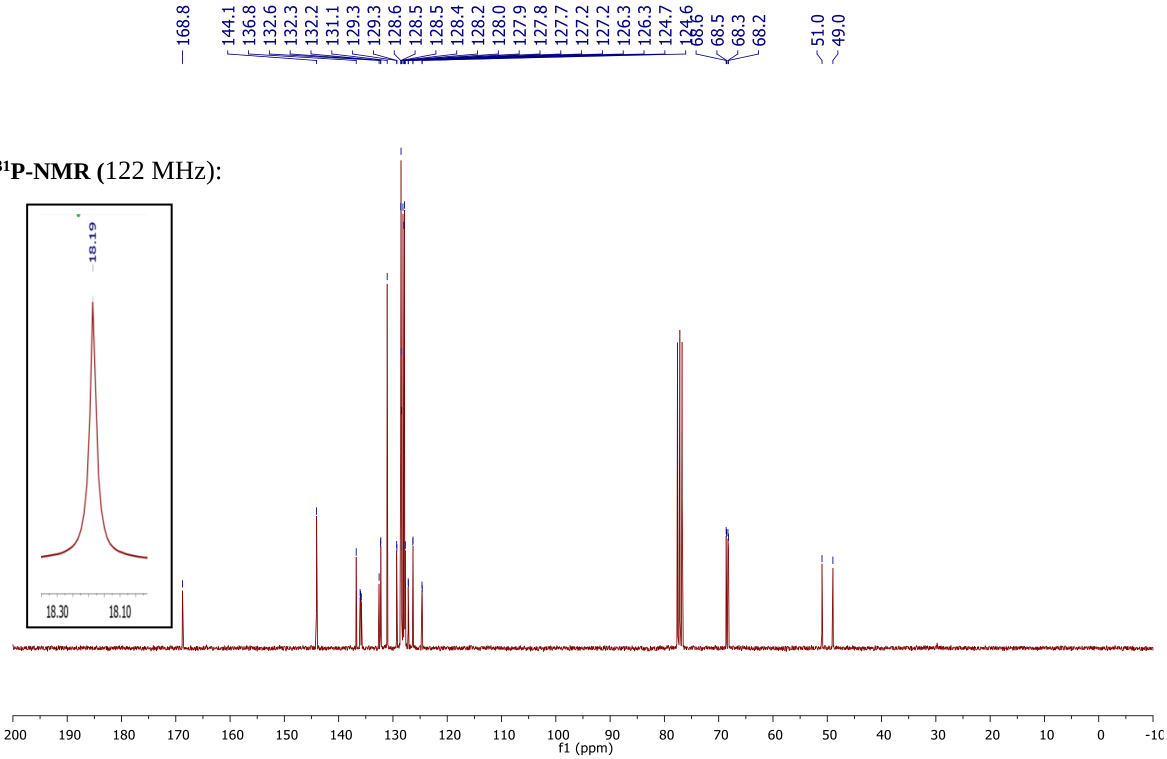

^{31}P-NMR (122 MHz):

^{1}H-NMR (CDCl$_3$, 300 MHz) of (*R/S*,*S*)-**11aF**

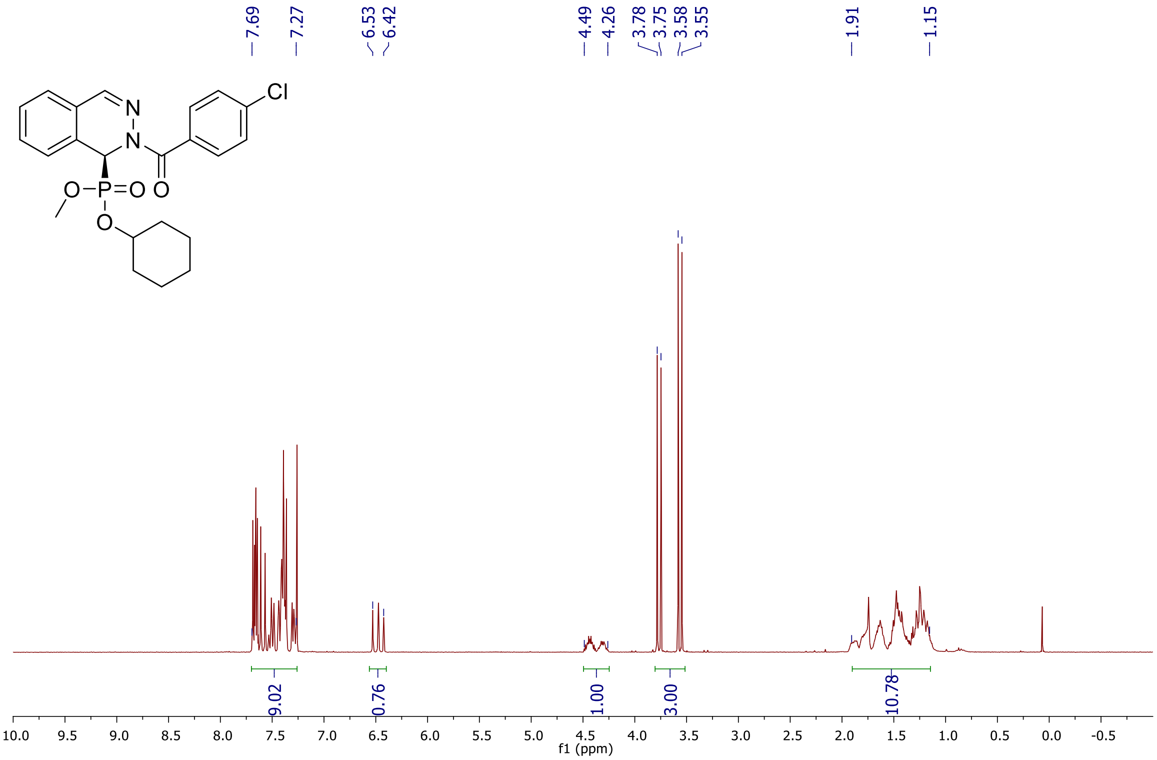

^{13}C-NMR (CDCl$_3$, 75.5 MHz) of (*R/S*,*S*)-**11aF**

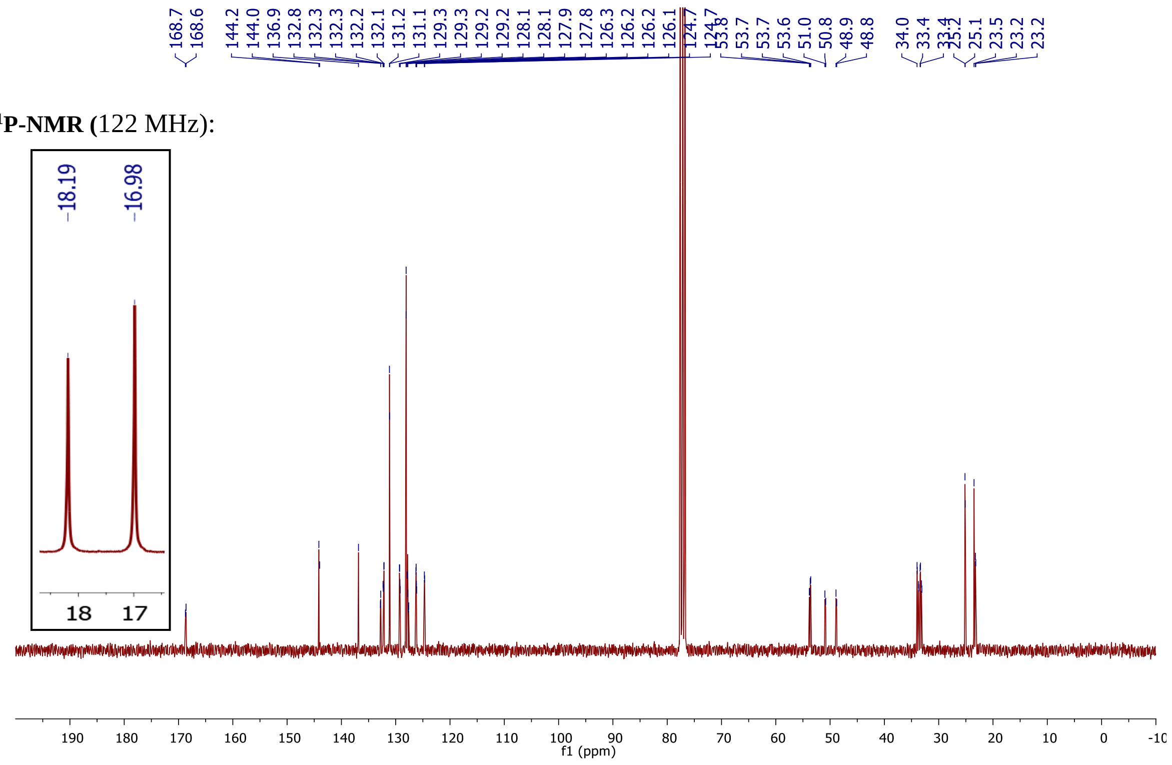

^{31}P-NMR (122 MHz):

^{1}H-NMR (CDCl$_3$, 300 MHz) of (*S*)-**11bA**

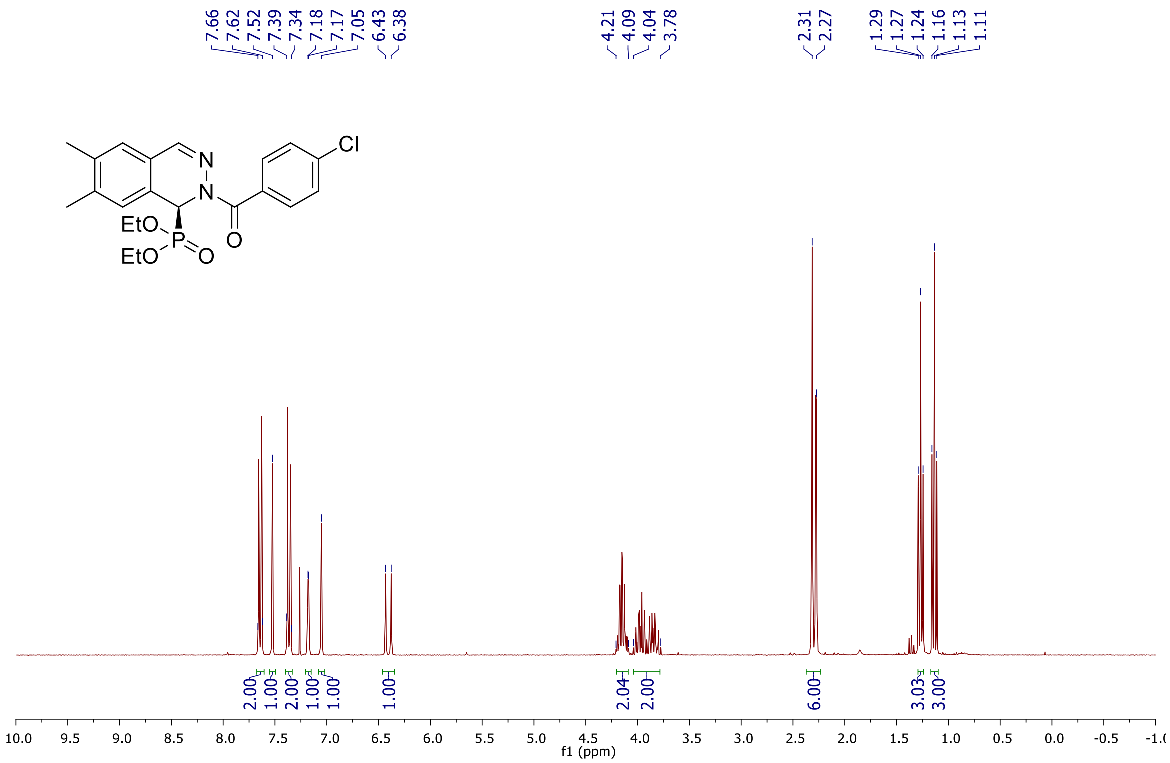

^{13}C-NMR (CDCl$_3$, 75.5 MHz) of (*S*)-**11bA**

^{31}P-NMR (122 MHz):

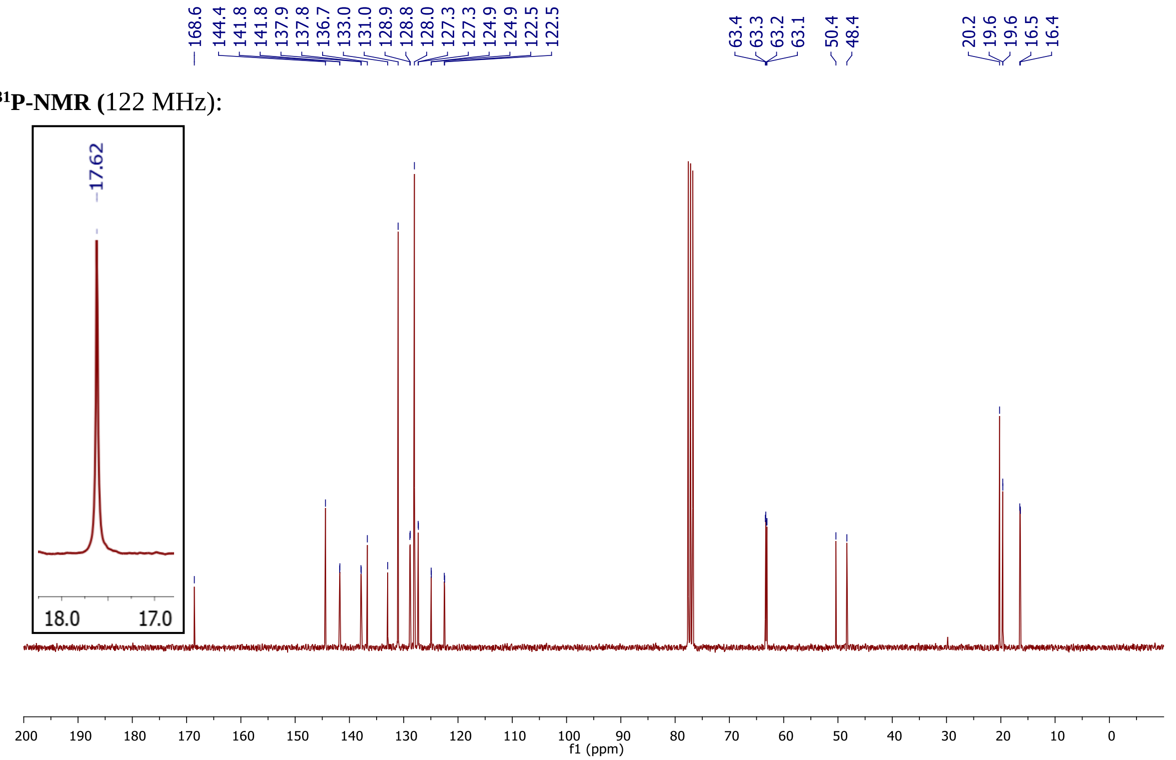

^{1}**H-NMR** (CDCl$_3$, 300 MHz) of (*S*)-**11cA**

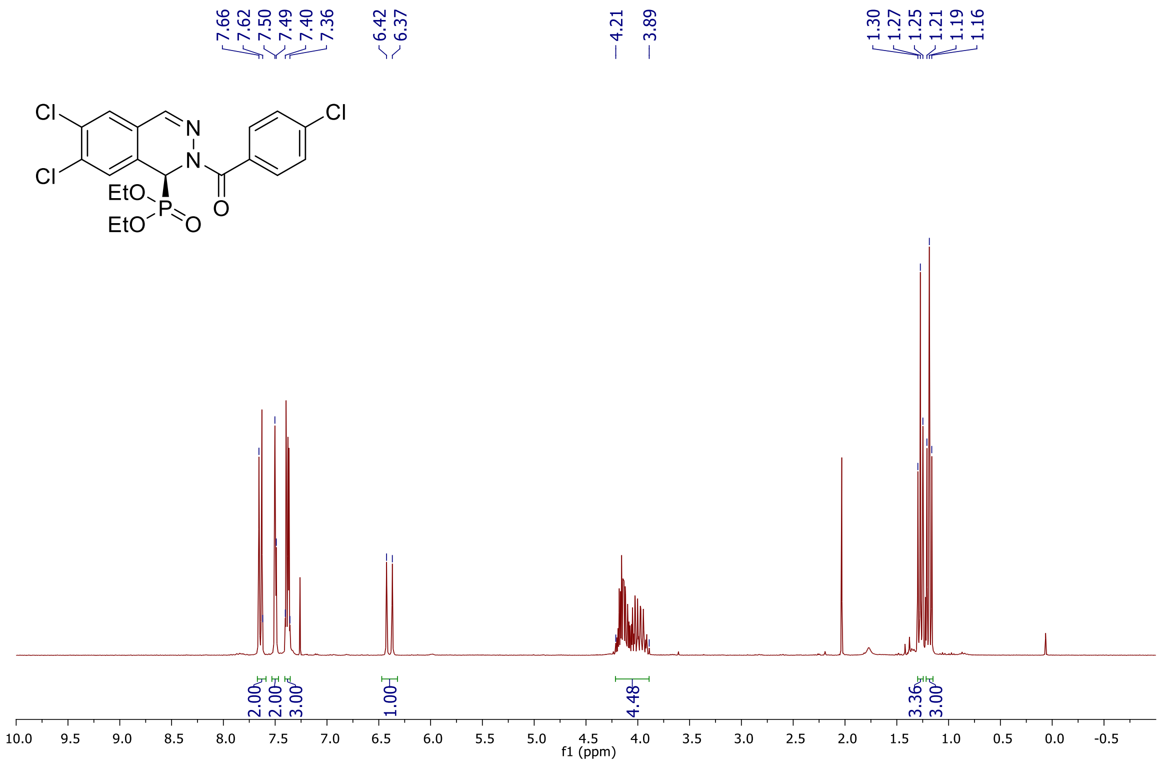

^{13}**C-NMR** (CDCl$_3$, 75.5 MHz) of (*S*)-**11cA**

^{31}**P-NMR (**122 MHz):

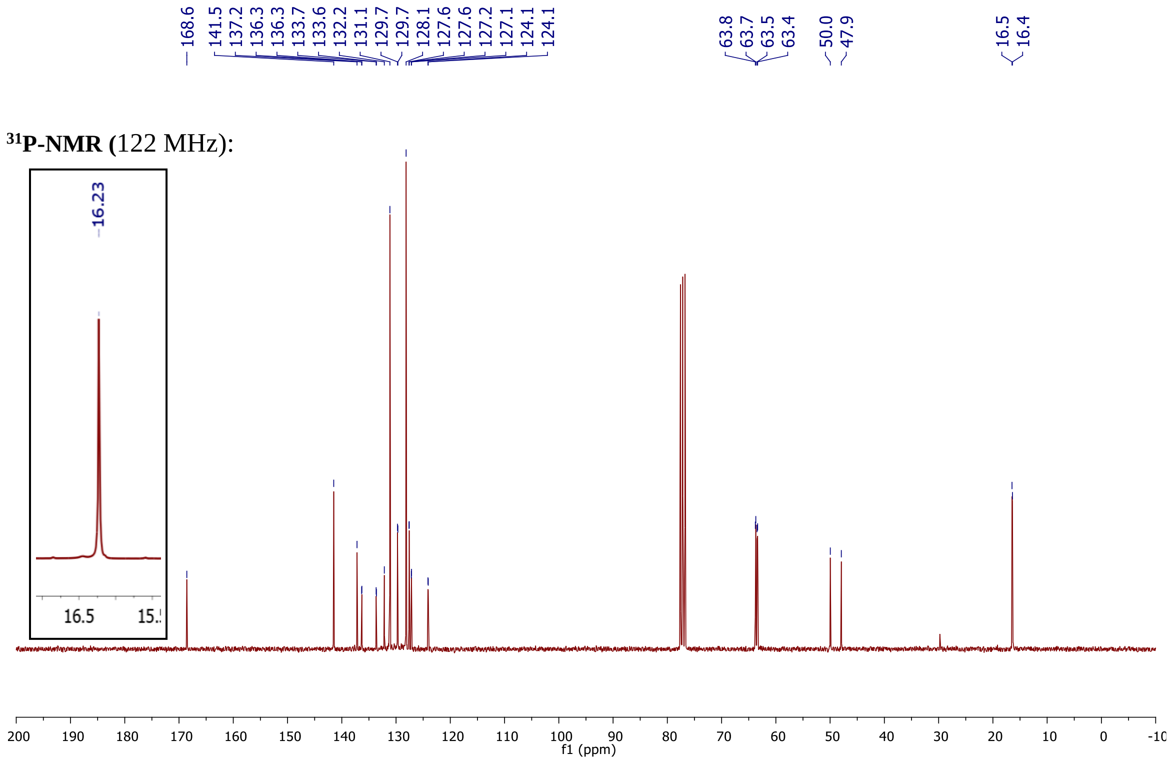

[1]**H-NMR** (CDCl$_3$, 300 MHz) of (*S*)-**11dA**

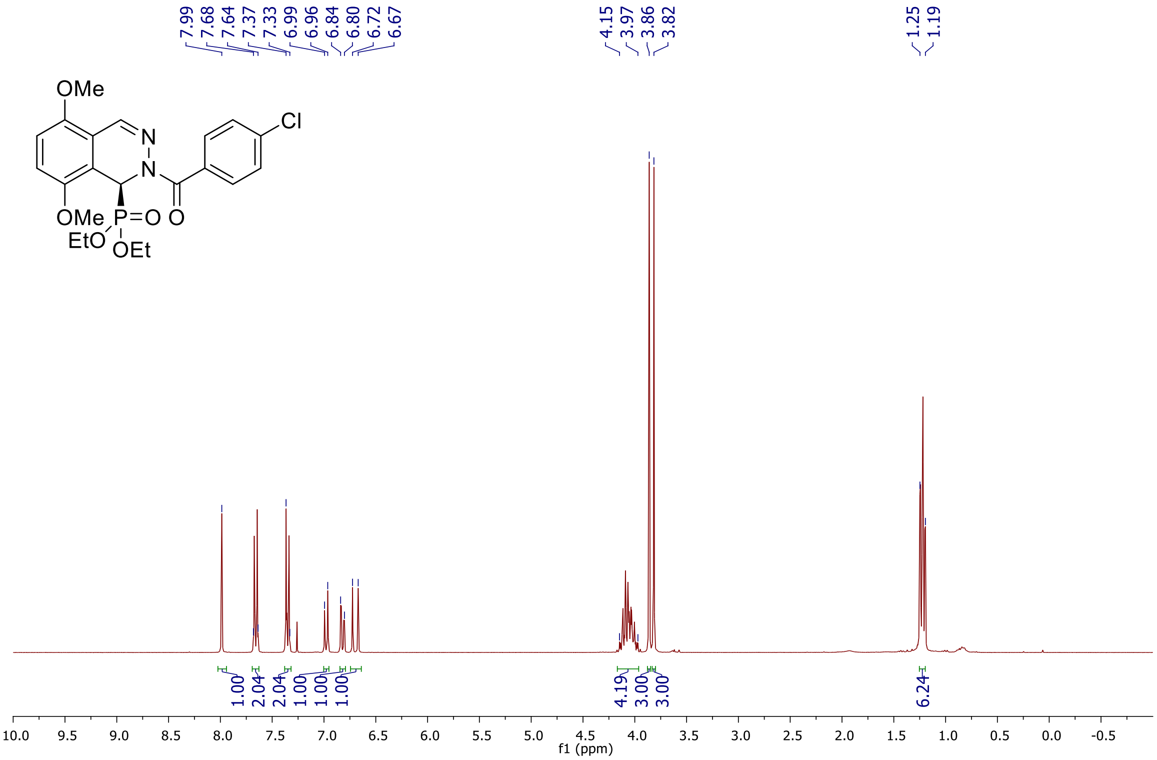

[13]**C-NMR** (CDCl$_3$, 75.5 MHz) of (*S*)-**11dA**

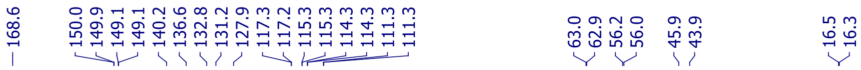

[31]**P-NMR (**122 MHz):

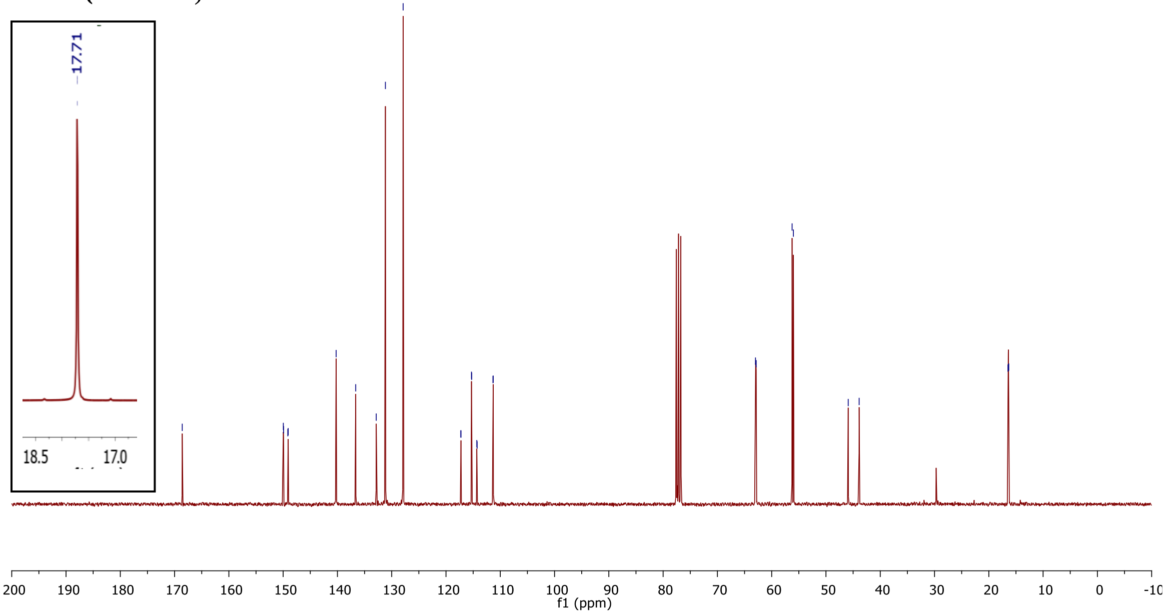

^{1}H-NMR (CDCl$_3$, 300 MHz) of (*S*)-**11eA**

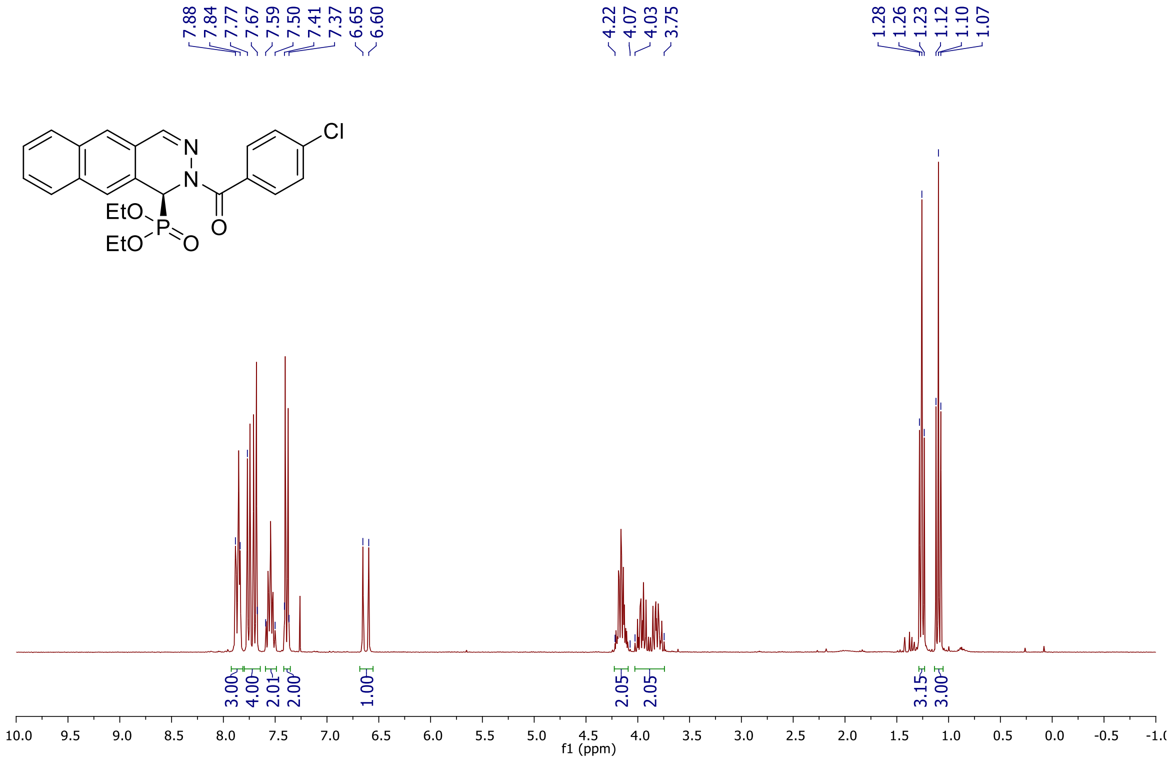

^{13}C-NMR (CDCl$_3$, 75.5 MHz) of (*S*)-**11eA**

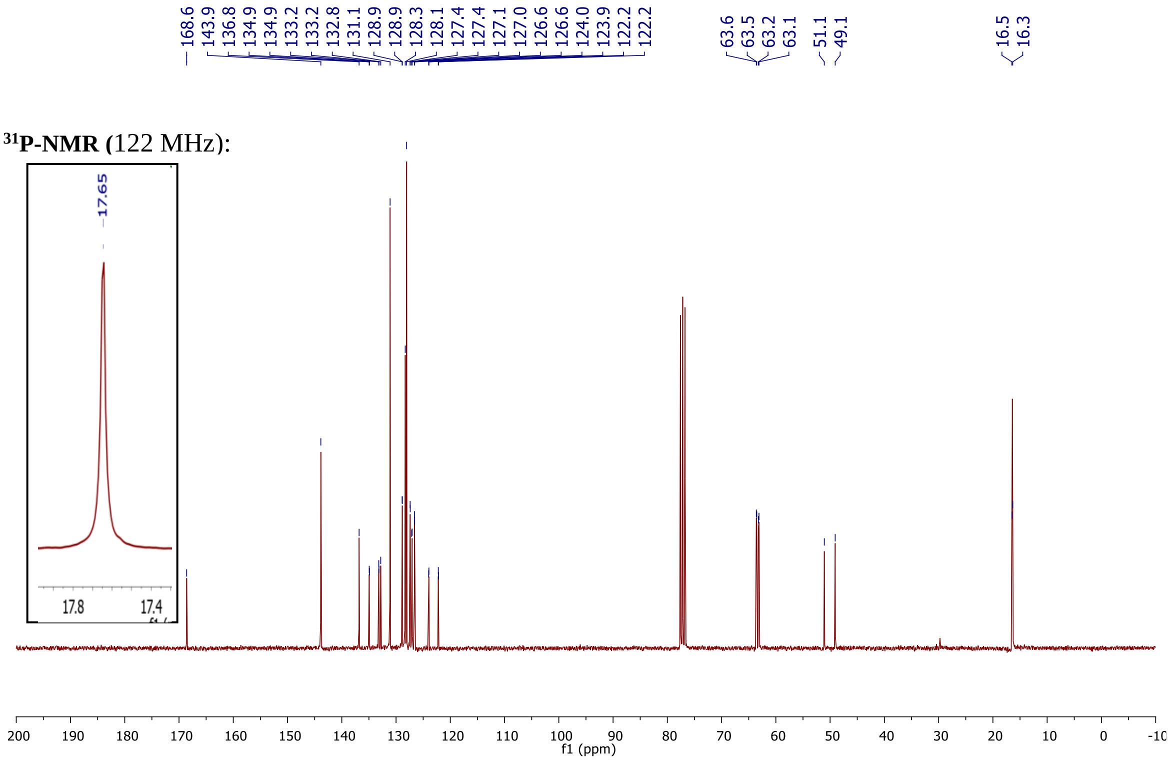

^{1}H-NMR (CDCl$_3$, 300 MHz) of (*S*)-**11fA/11fA'**

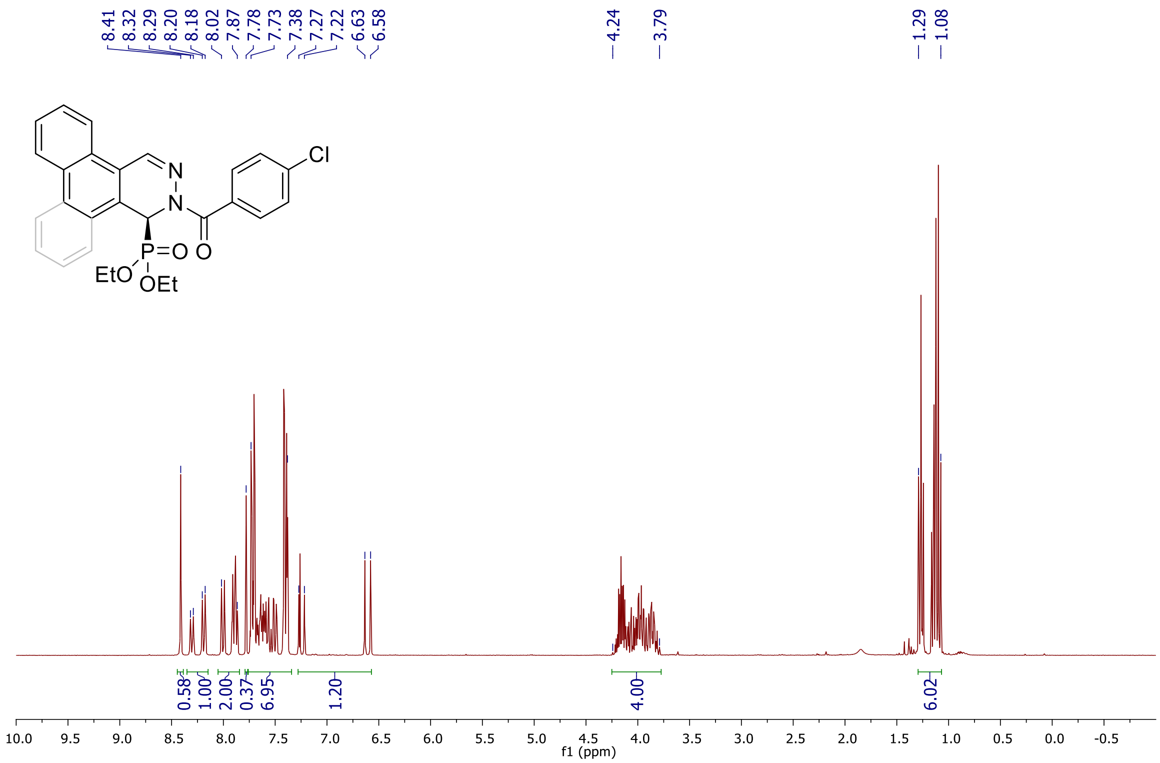

^{13}C-NMR (CDCl$_3$, 75.5 MHz) of (*S*)-**11fA/11fA'**

^{31}P-NMR (122 MHz):

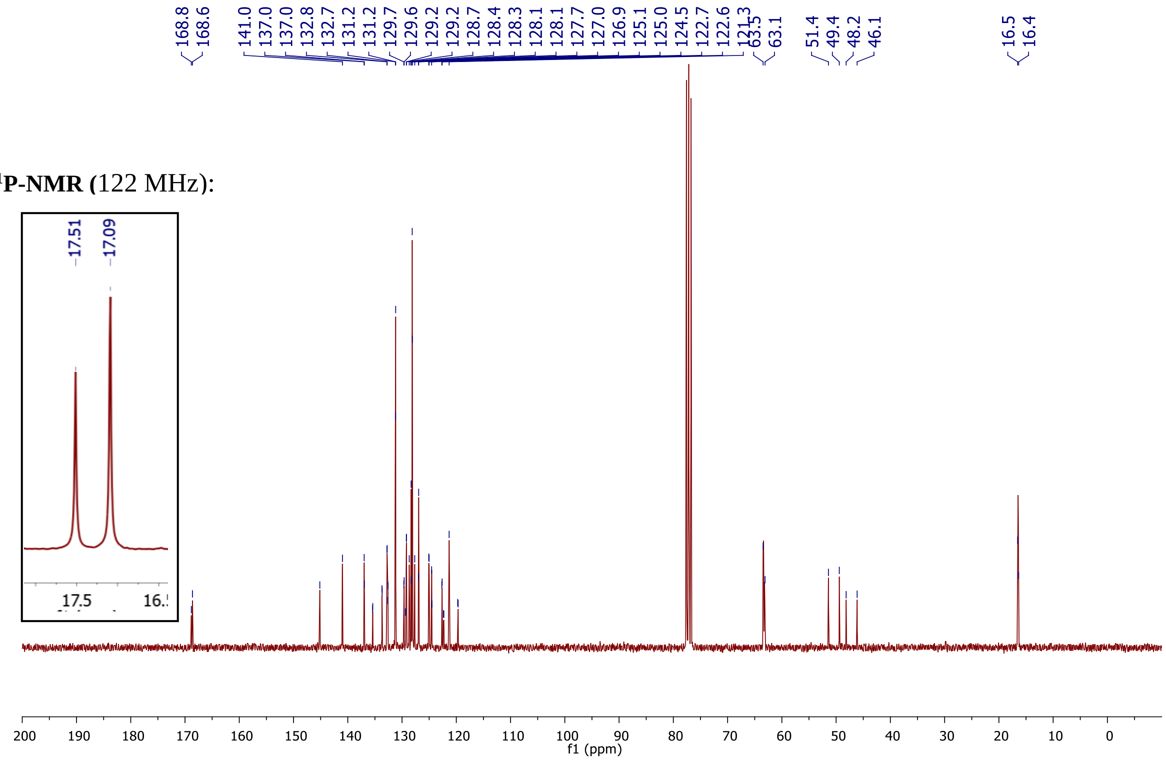

[1]**H-NMR** (CDCl$_3$, 300 MHz) of (*S*)-**11gA**

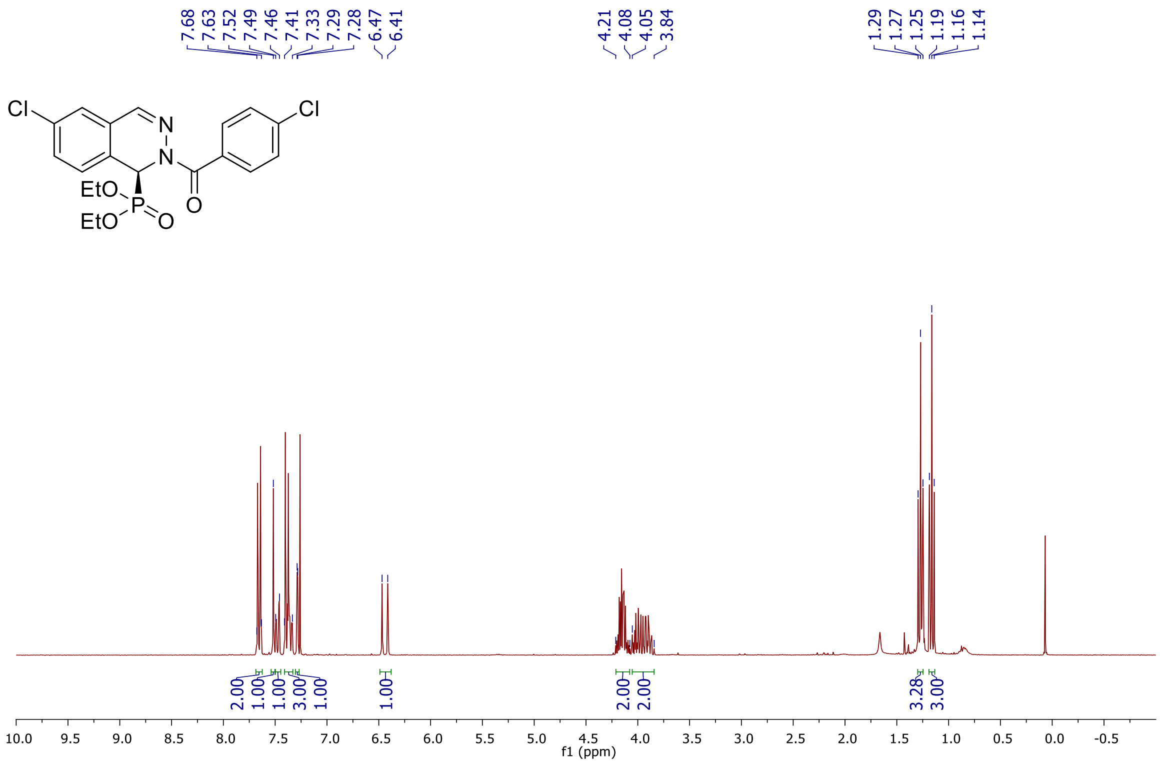

[13]**C-NMR** (CDCl$_3$, 75.5 MHz) of (*S*)-**11gA**

[31]**P-NMR (**122 MHz):

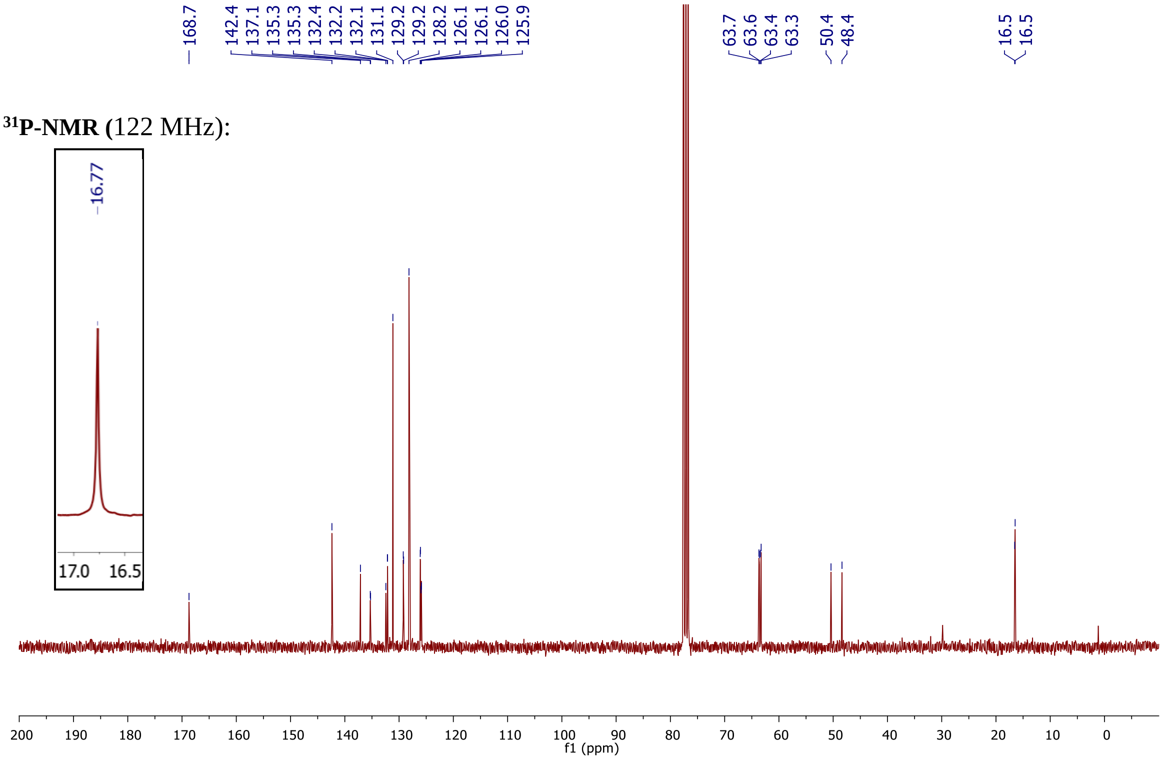

COSY (CDCl$_3$, 300 MHz) of (*S*)-**11gA**

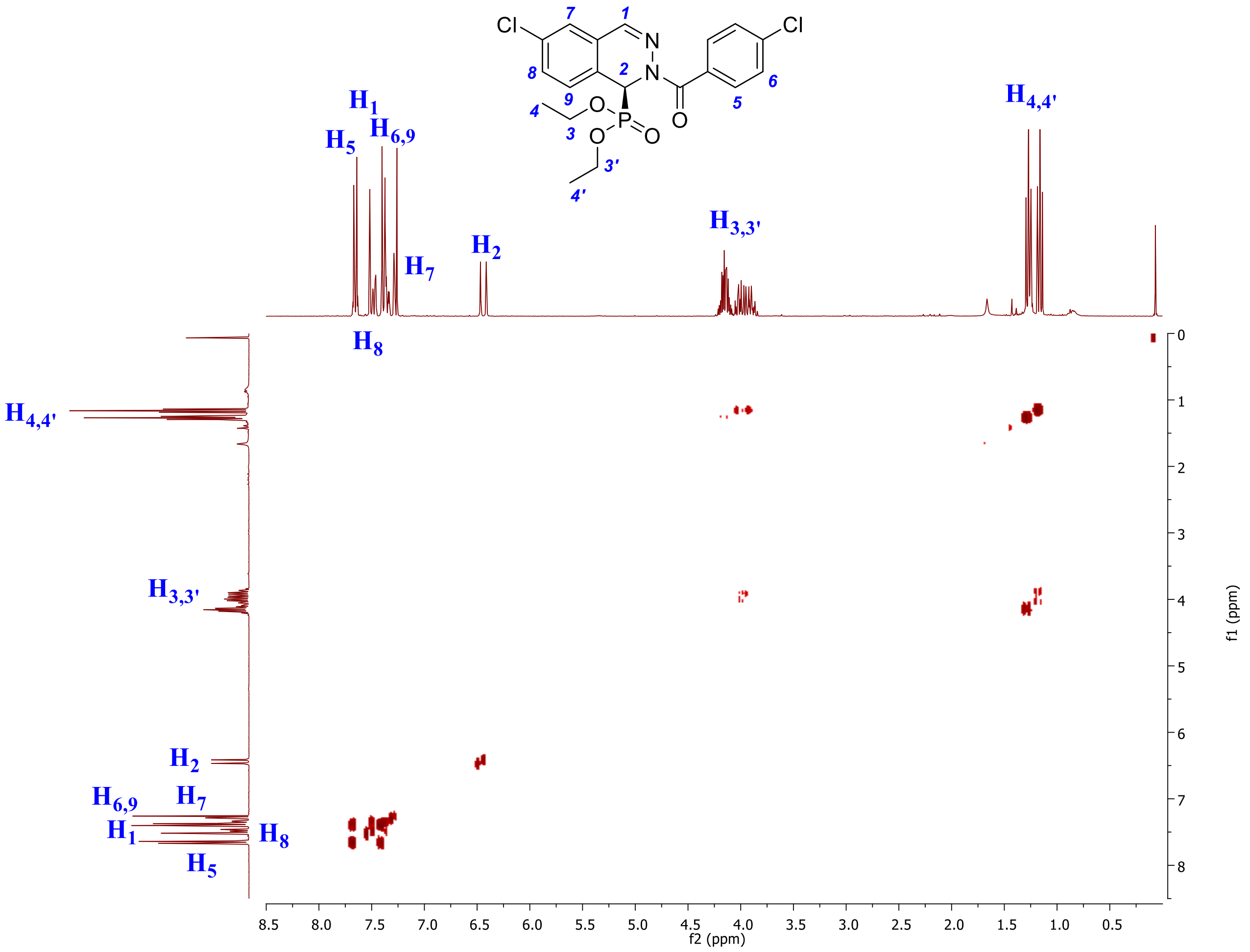

NOESY (CDCl$_3$, 300 MHz) of (*S*)-**11gA**

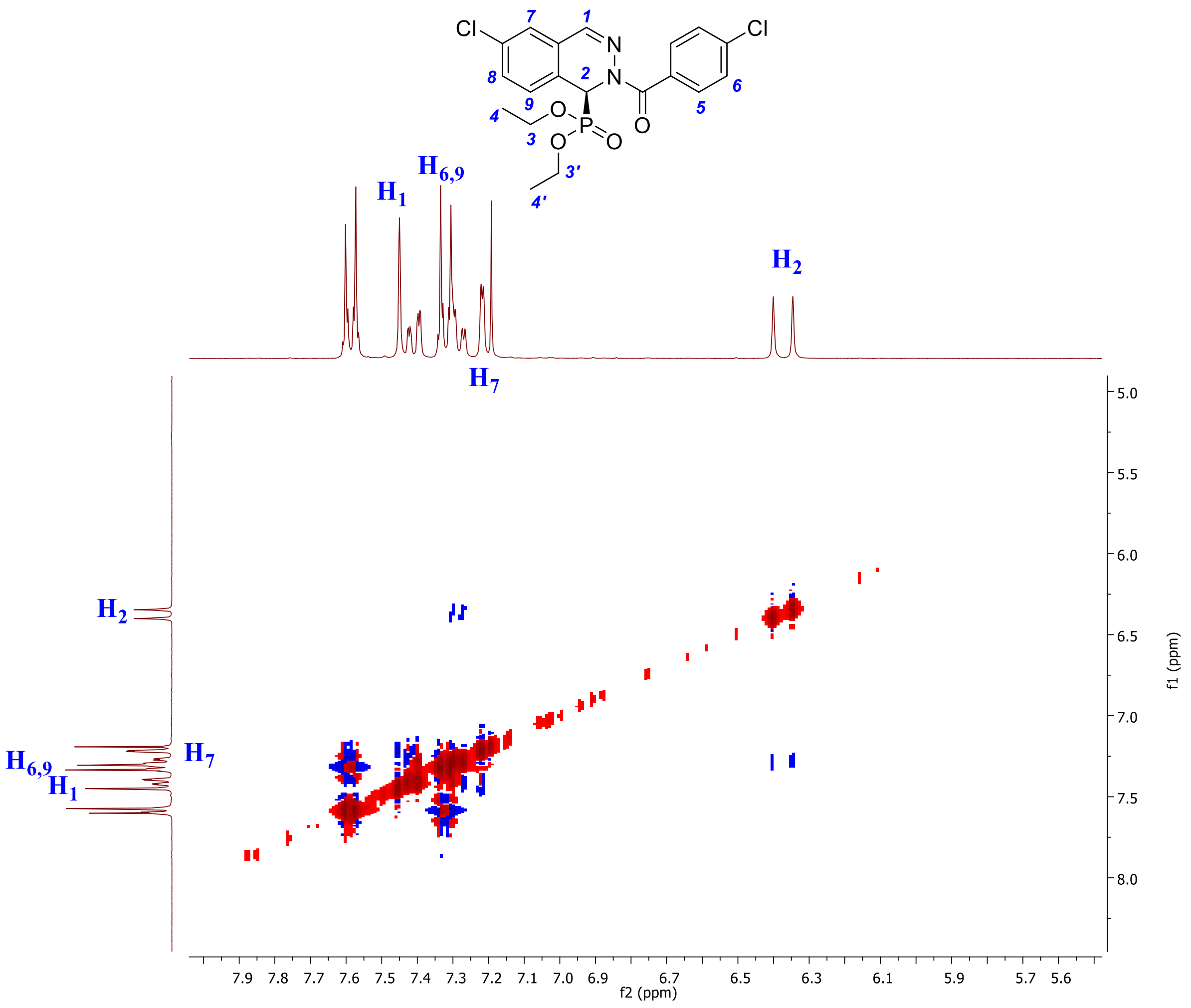

^{1}H-NMR (CDCl$_3$, 300 MHz) of (*S*)-**11gA'**

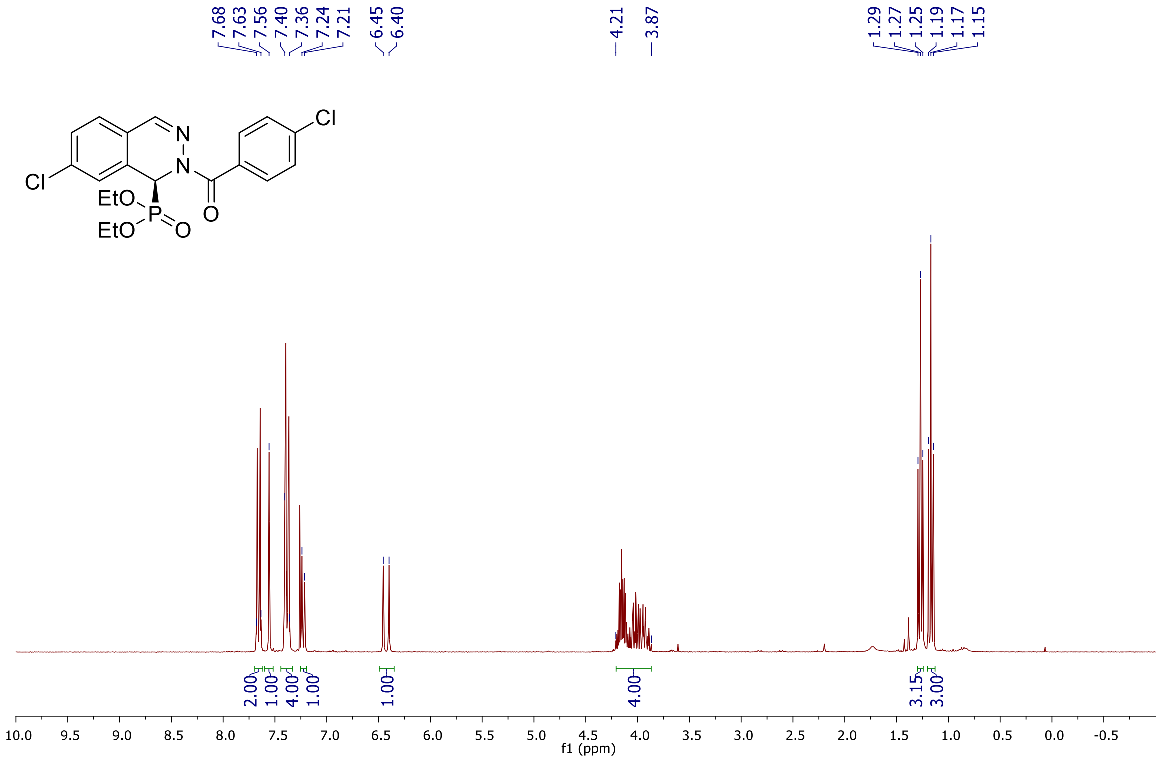

^{13}C-NMR (CDCl$_3$, 75.5 MHz) of (*S*)-**11gA'**

^{31}P-NMR (122 MHz):

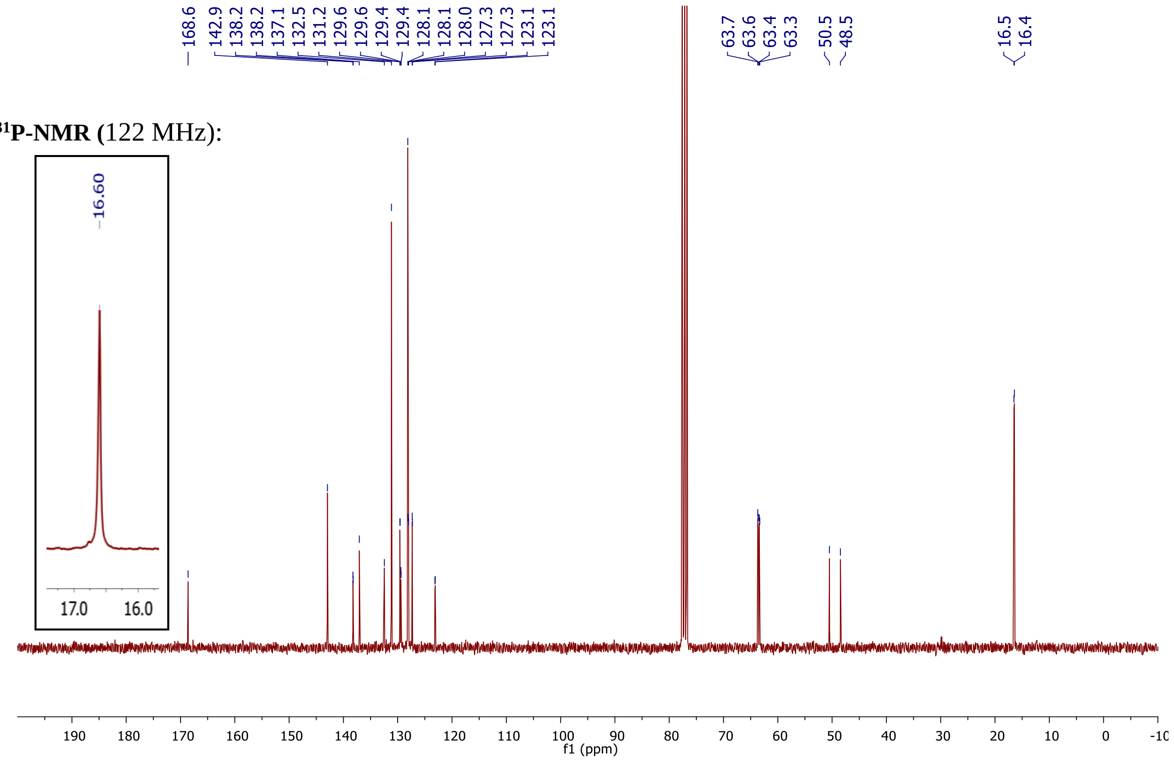

COSY ($CDCl_3$, 300 MHz) of (*S*)-**11gA'**

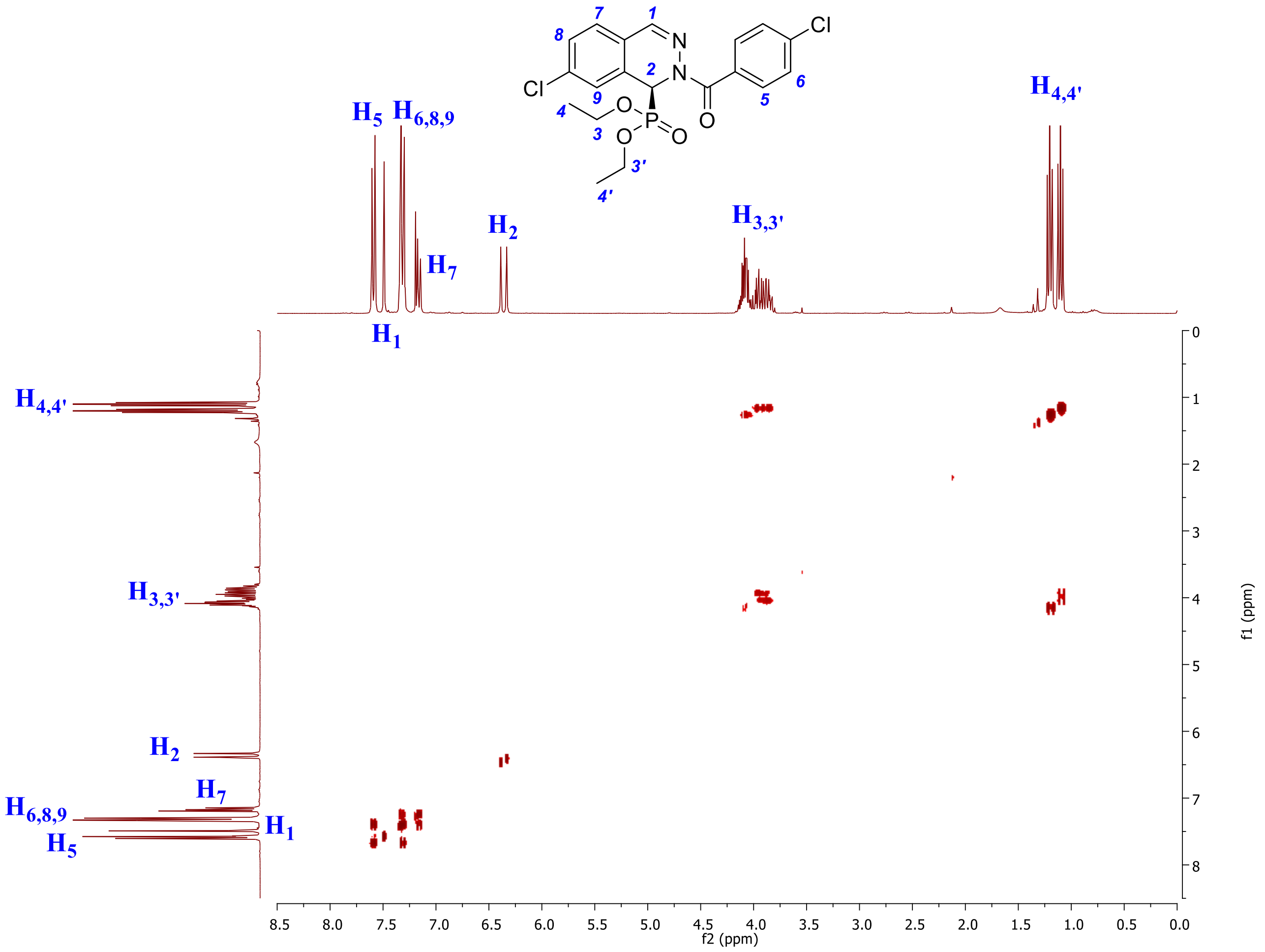

NOESY (CDCl$_3$, 300 MHz) of (*S*)-**11gA'**

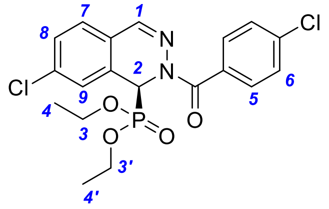

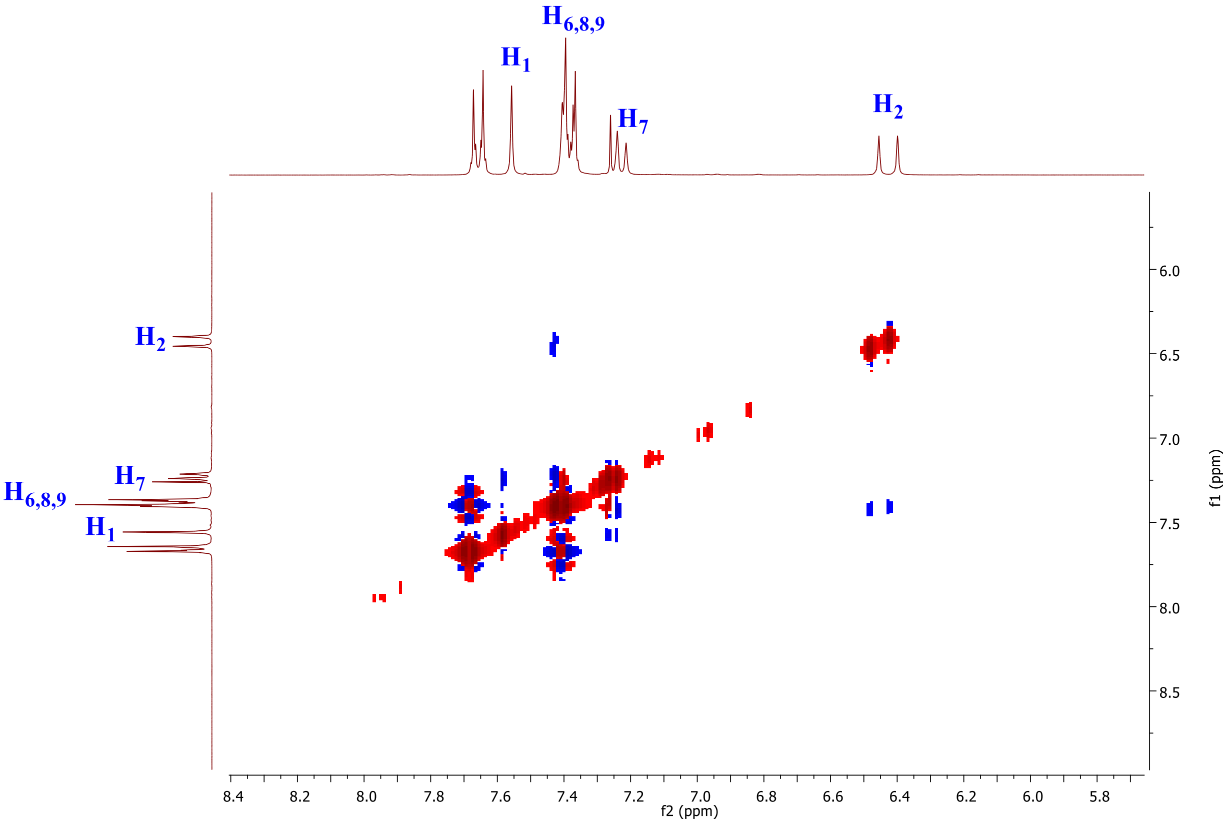

^{1}H-NMR (CDCl$_3$, 300 MHz) of (*S*)-**11hA**

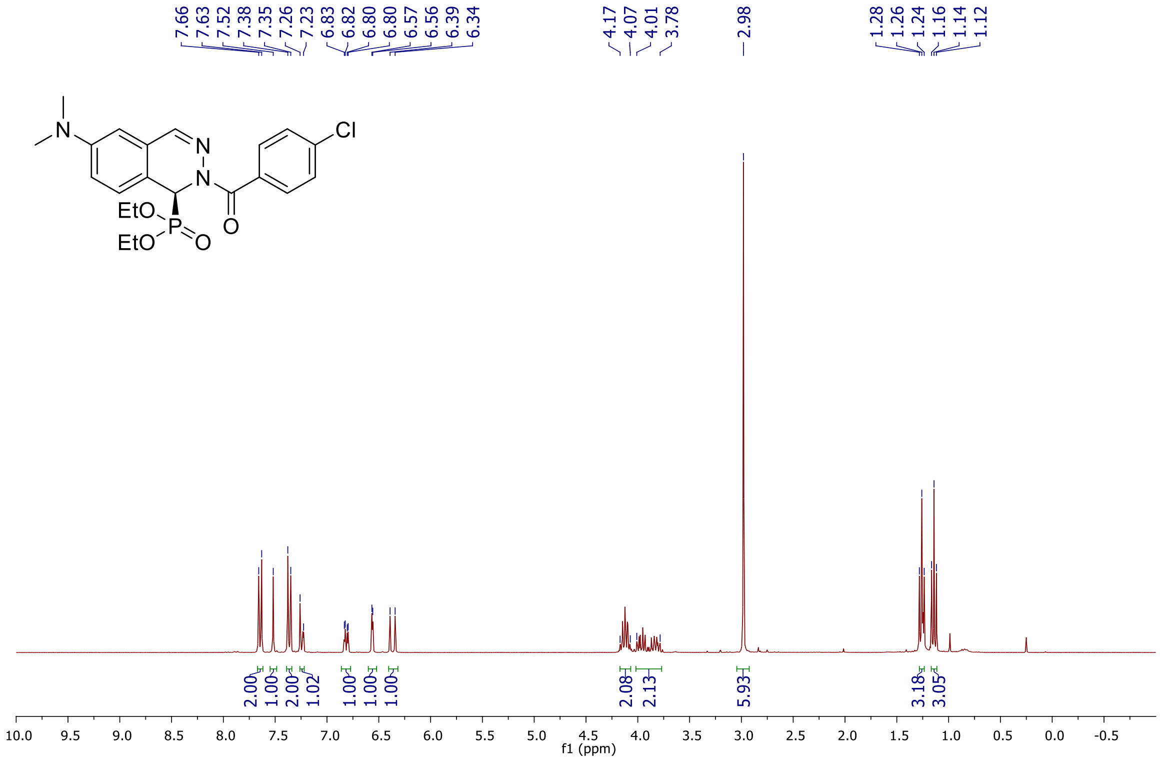

^{13}C-NMR (CDCl$_3$, 75.5 MHz) of (*S*)-**11hA**

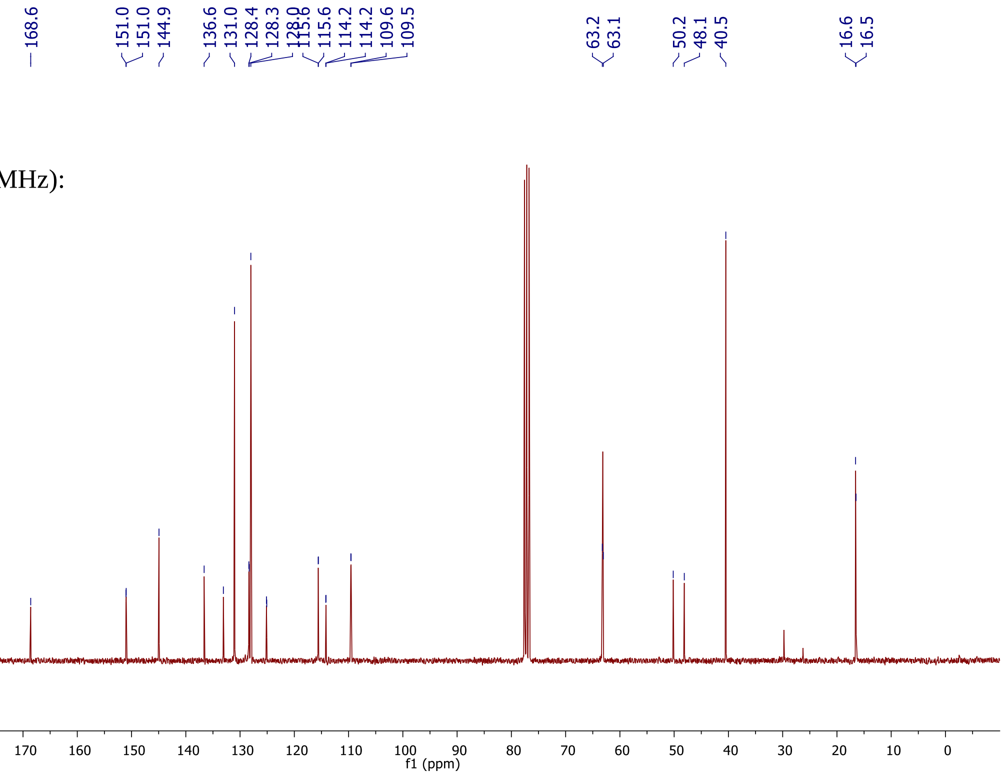

^{31}P-NMR (122 MHz):

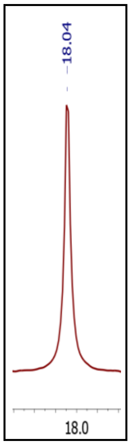

COSY (CDCl$_3$, 300 MHz) of (*S*)-**11hA**

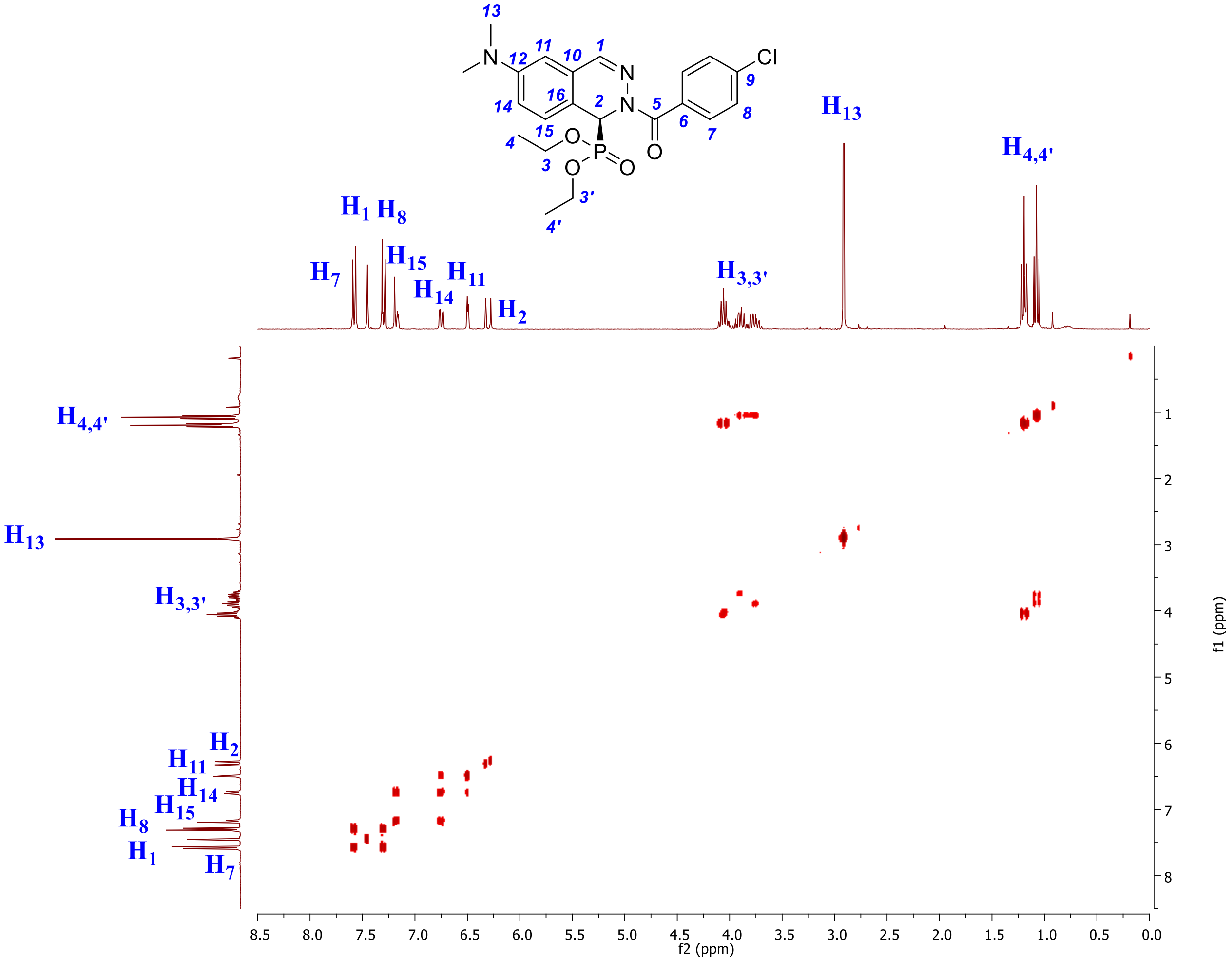

HSQC (CDCl$_3$, 300 MHz) of (*S*)-**11hA**

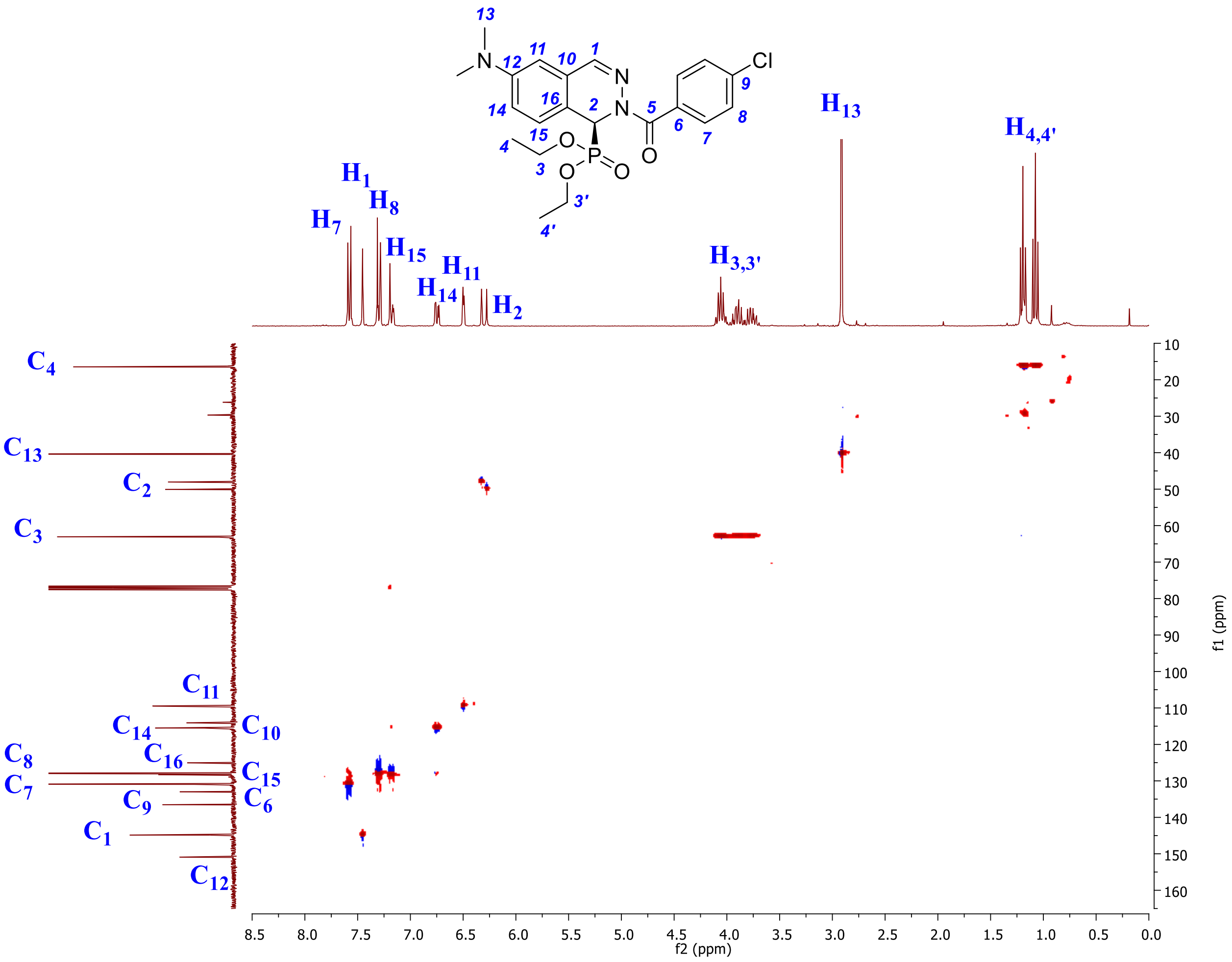

HMBC ($CDCl_3$, 300 MHz) of (*S*)-**11hA**

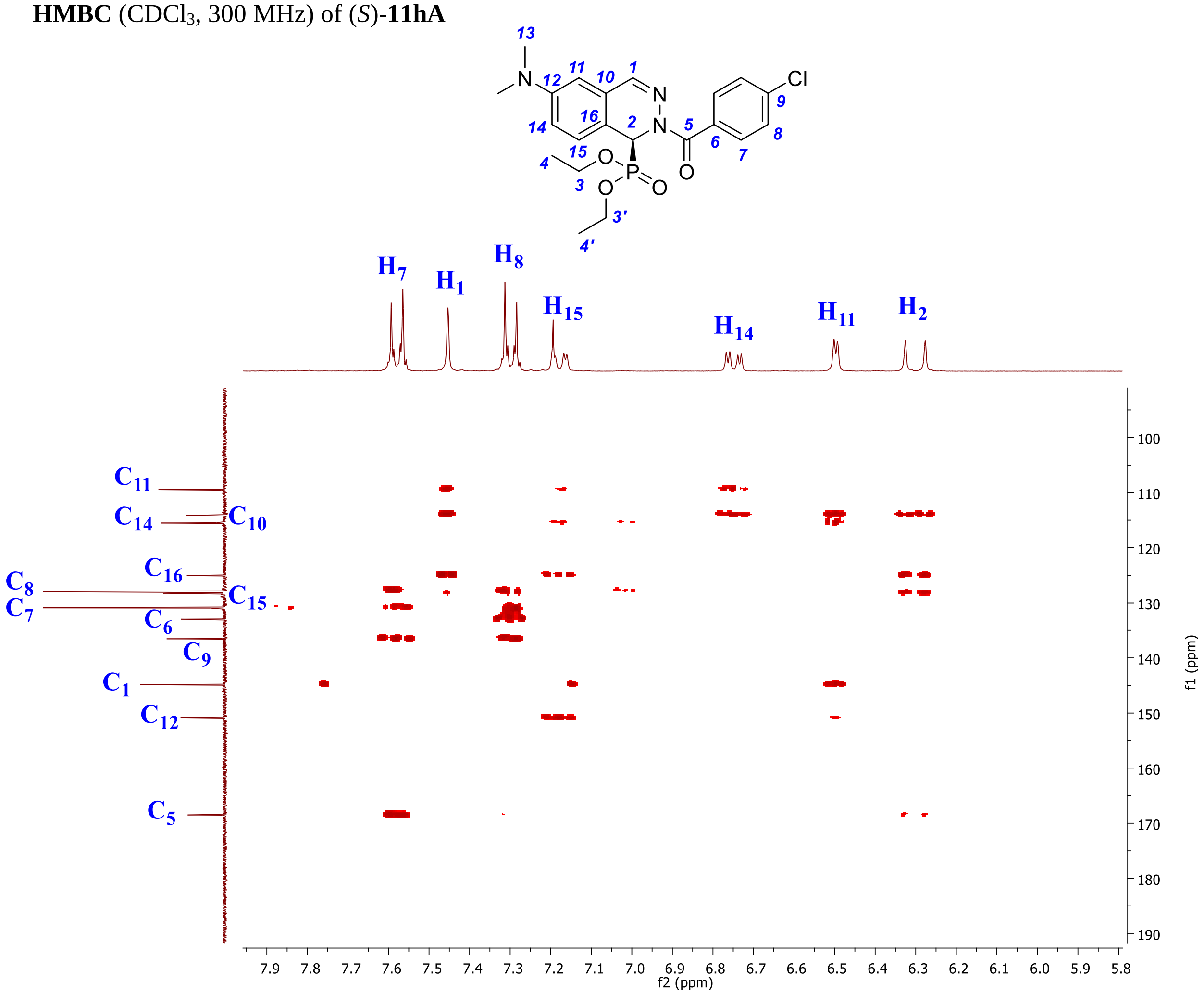

NOESY ($CDCl_3$, 300 MHz) of (*S*)-**11hA**

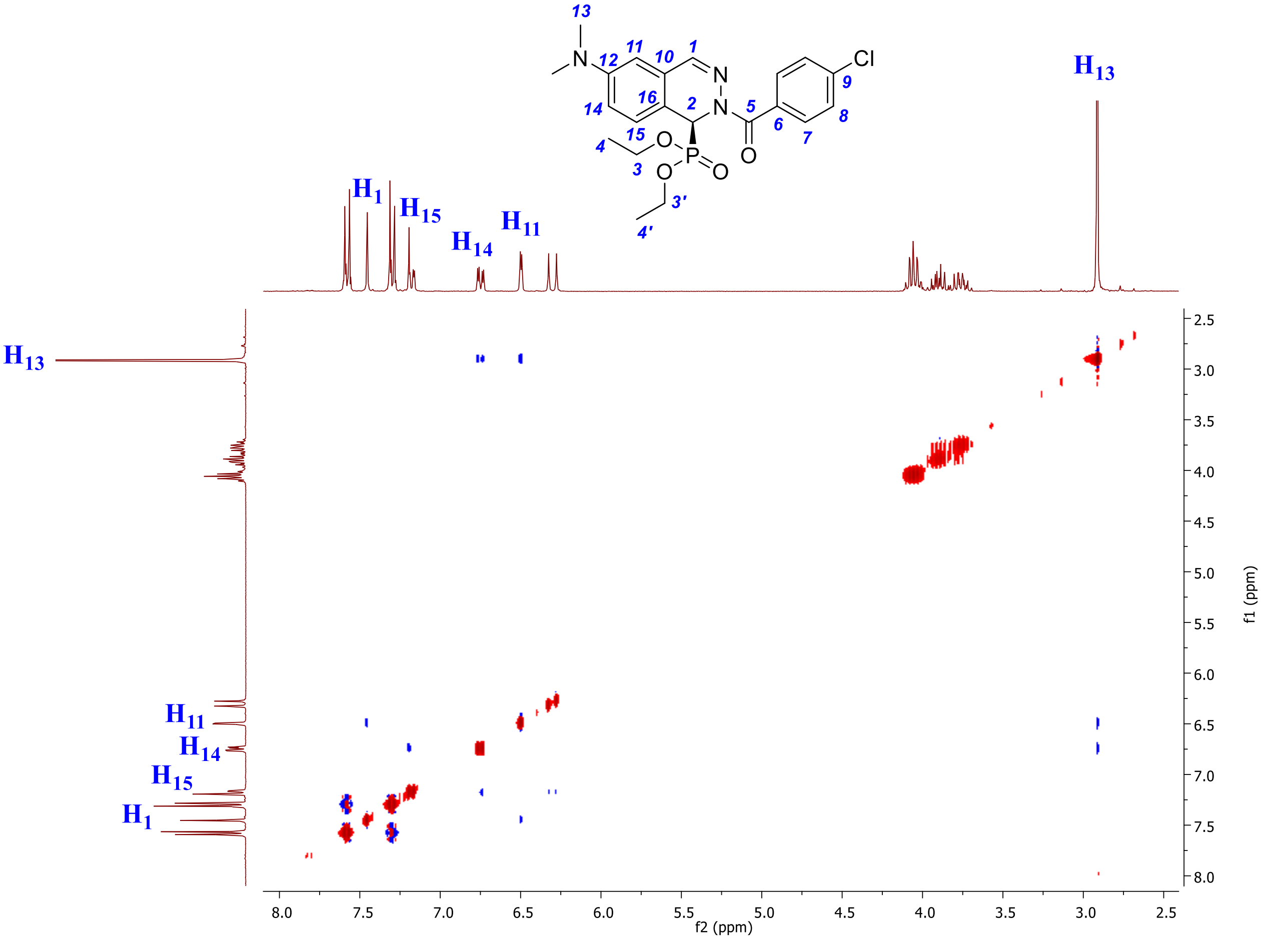

^{1}H-NMR (CDCl$_3$, 300 MHz) of (*S*)-**11iA**

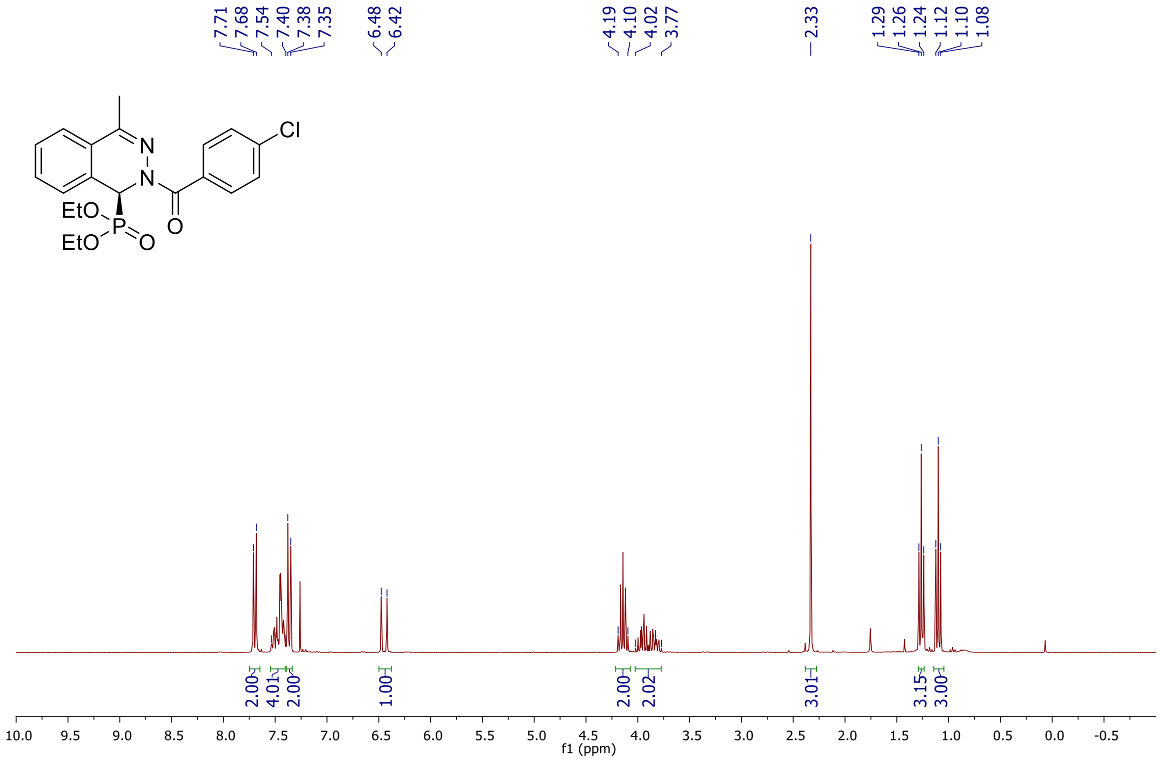

^{13}C-NMR (CDCl$_3$, 75.5 MHz) of (*S*)-**11iA**

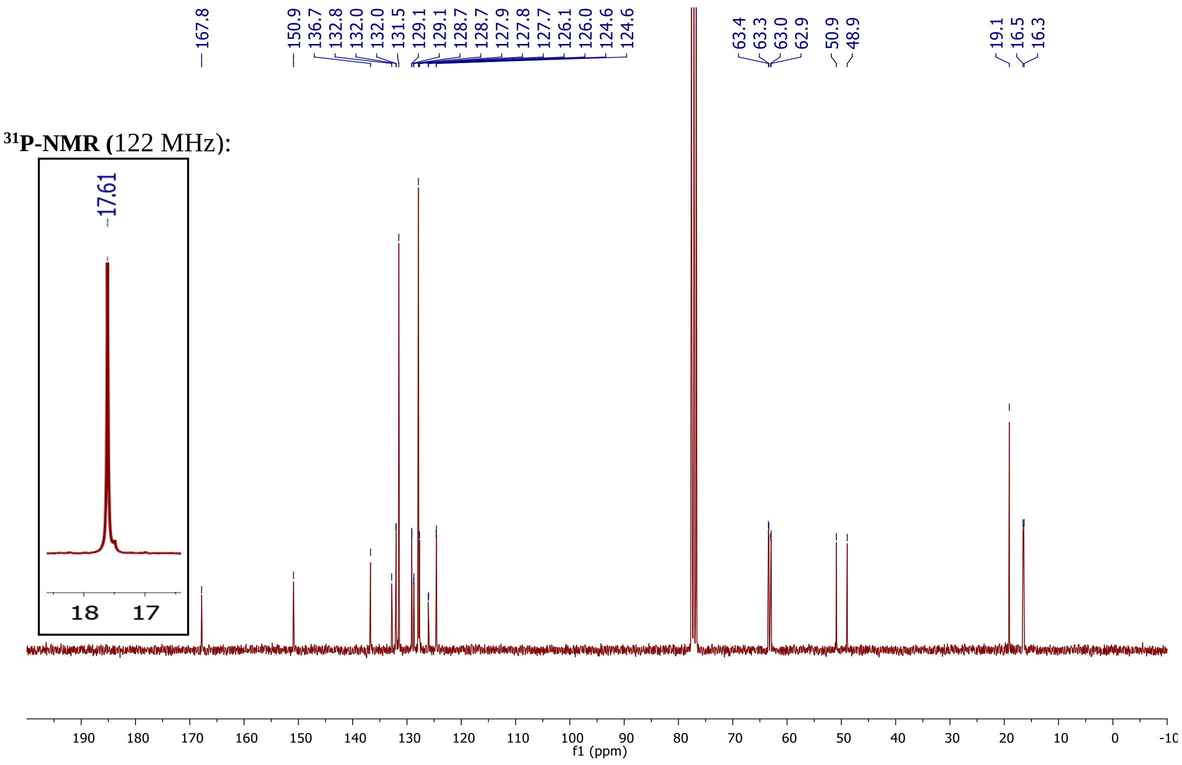

^{1}H-NMR (CDCl$_3$, 300 MHz) of (*S*)-**11jA**

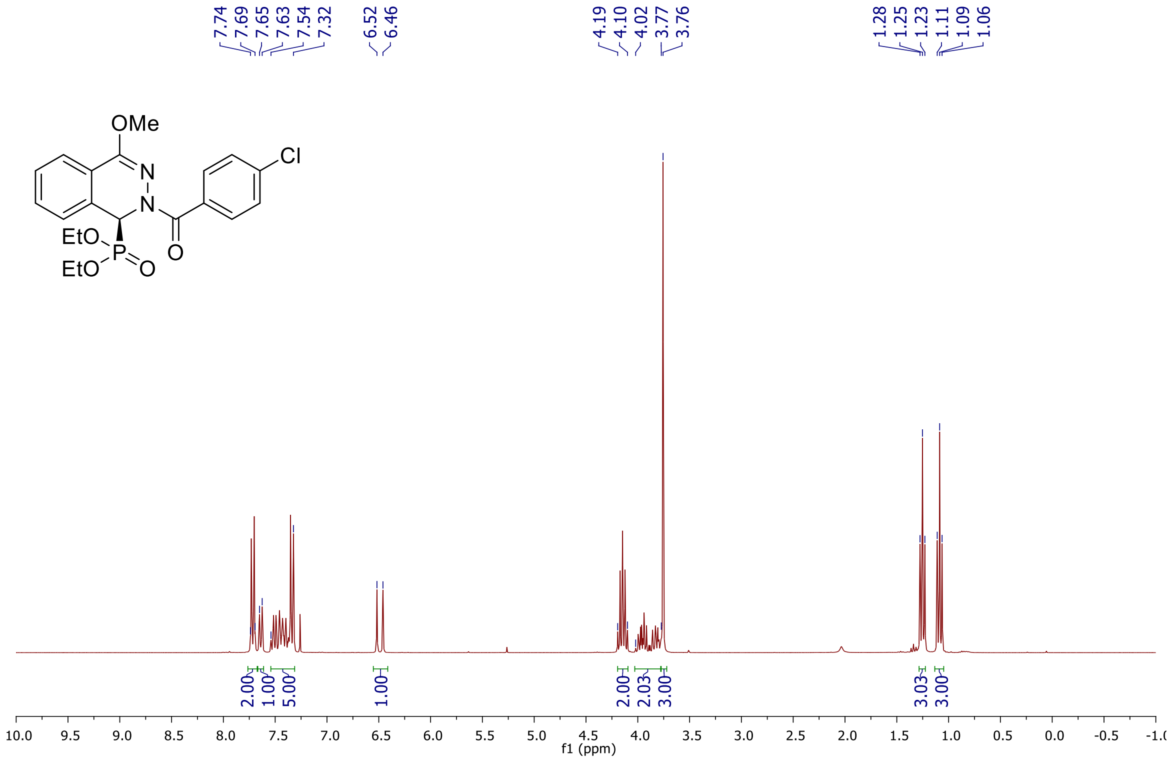

^{13}C-NMR (CDCl$_3$, 75.5 MHz) of (*S*)-**11jA**

^{31}P-NMR (122 MHz):

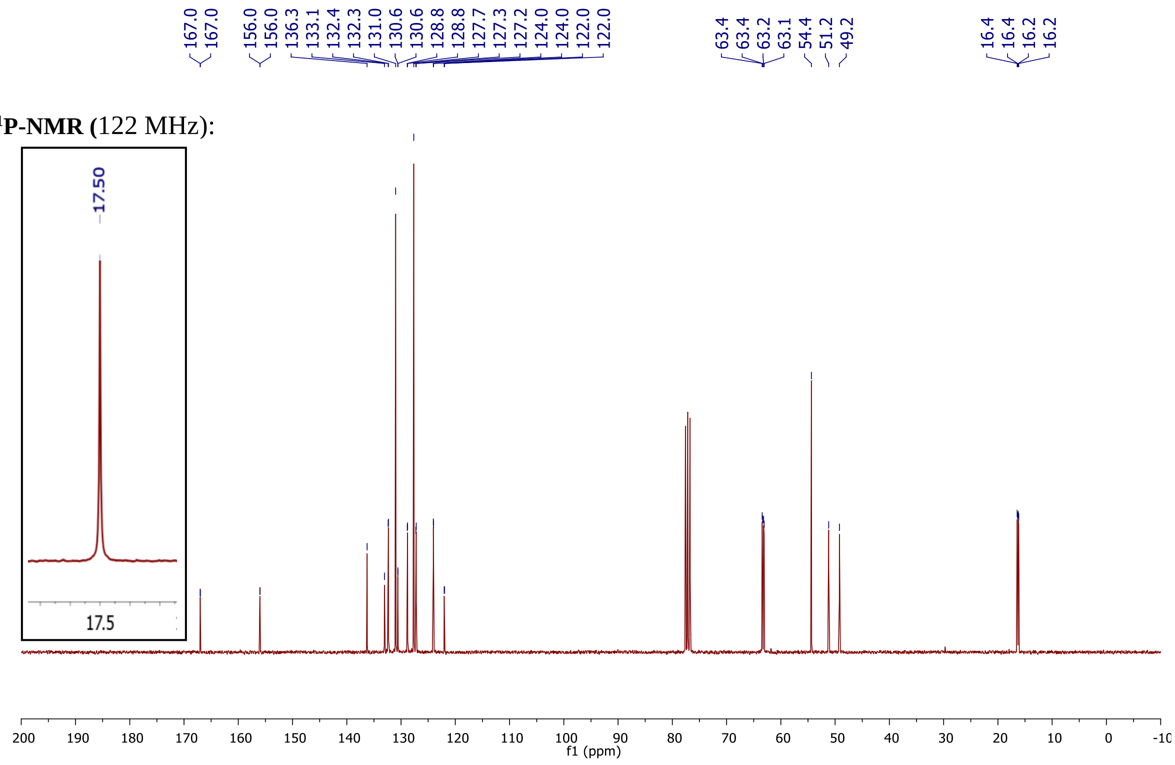

^{1}H-NMR (CDCl$_3$, 300 MHz) of (*S*)-**11kA**

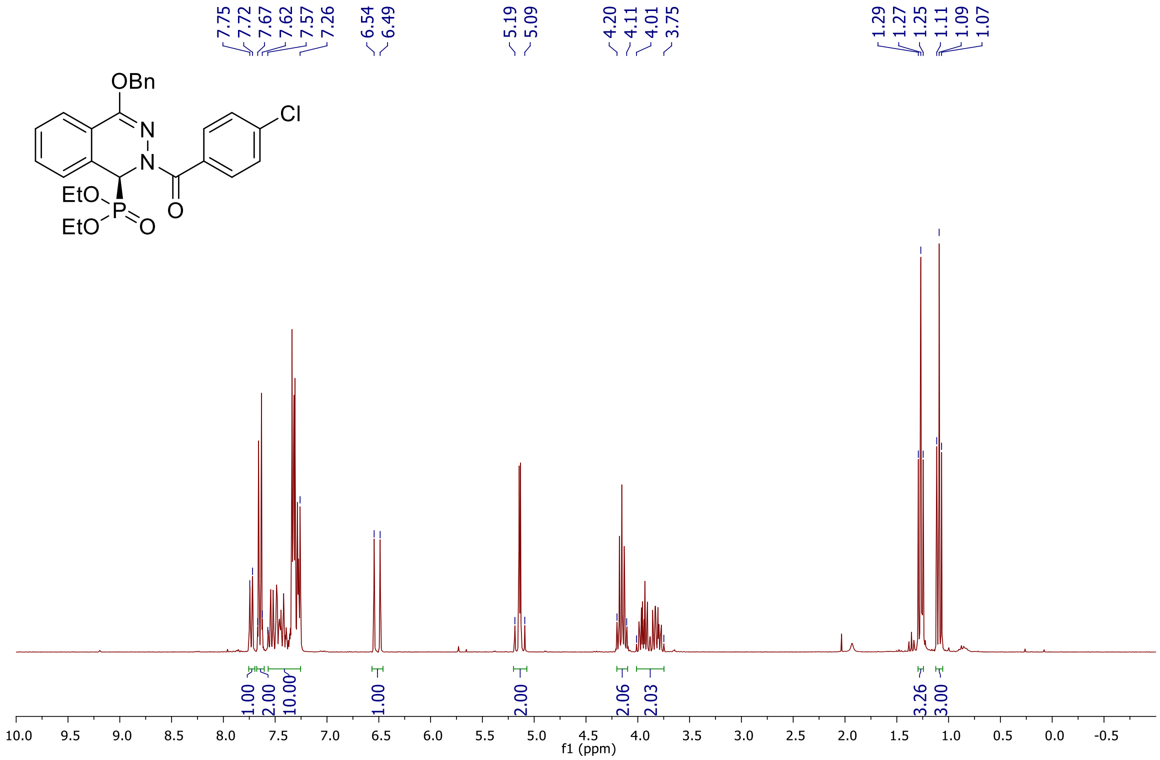

^{13}C-NMR (CDCl$_3$, 75.5 MHz) of (*S*)-**11kA**

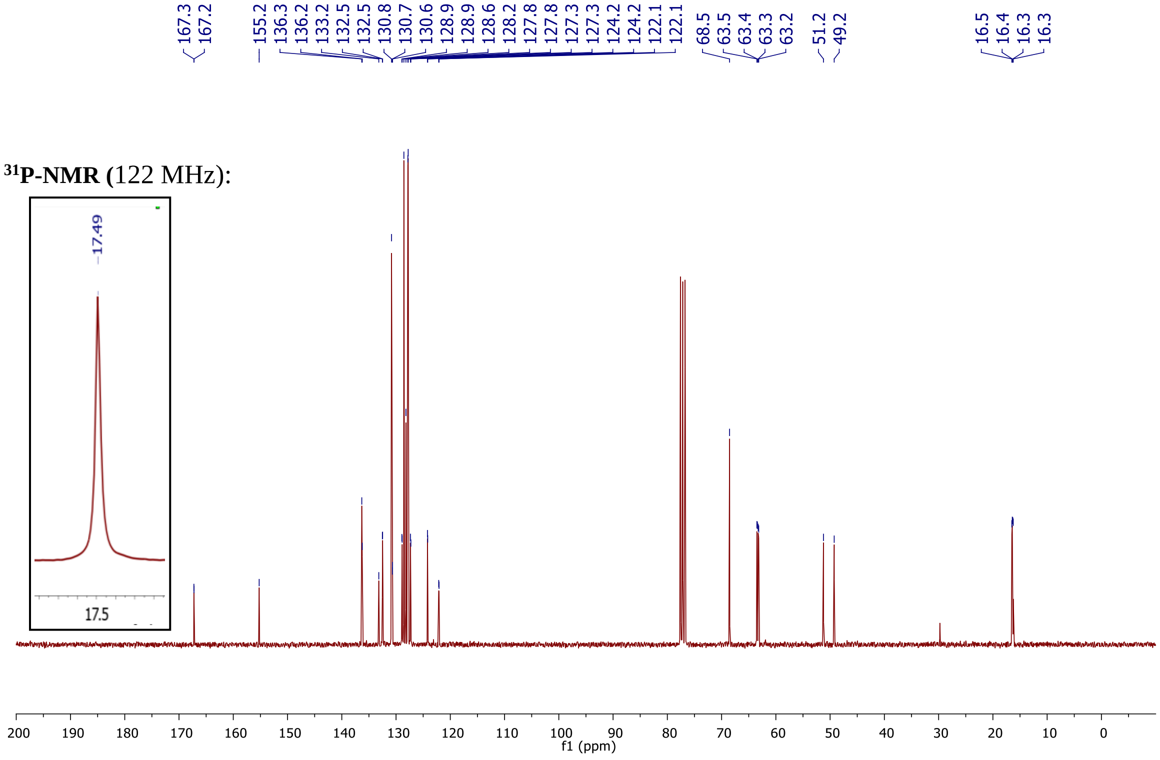

[1]**H-NMR** (CDCl$_3$, 300 MHz) of (*S*)-**11lA**

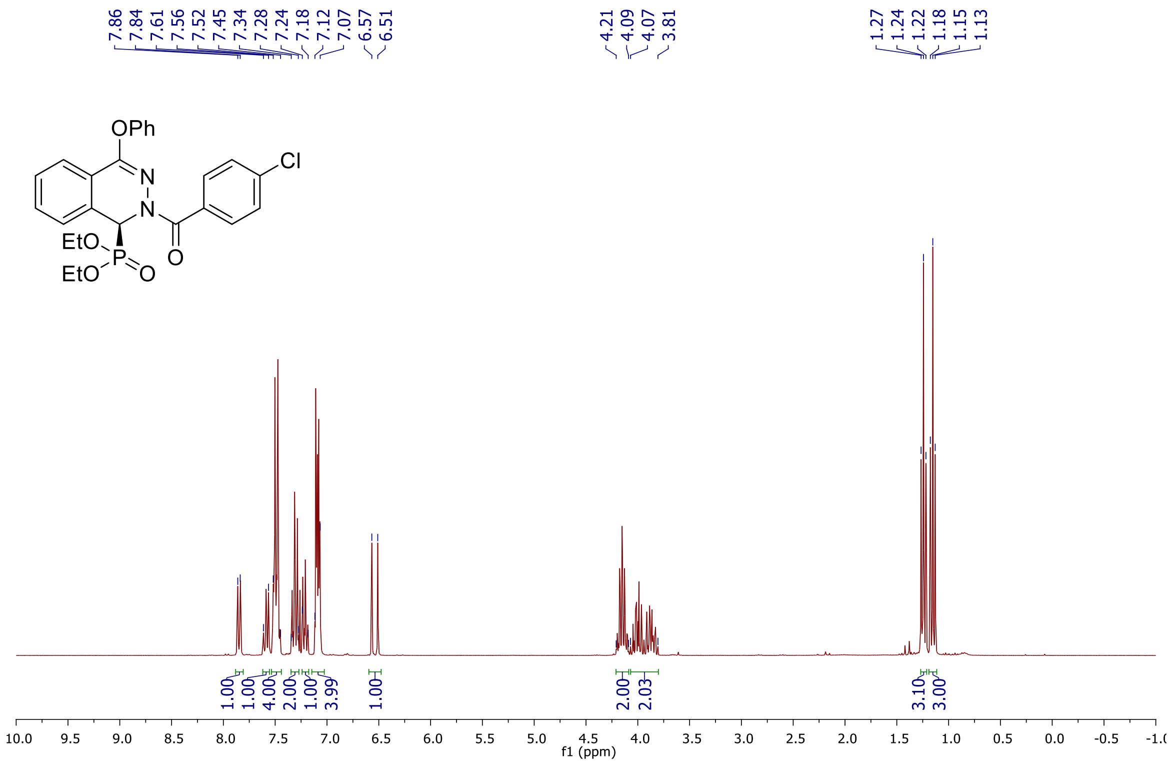

[13]**C-NMR** (CDCl$_3$, 75.5 MHz) of (*S*)-**11lA**

[31]**P-NMR (**122 MHz):

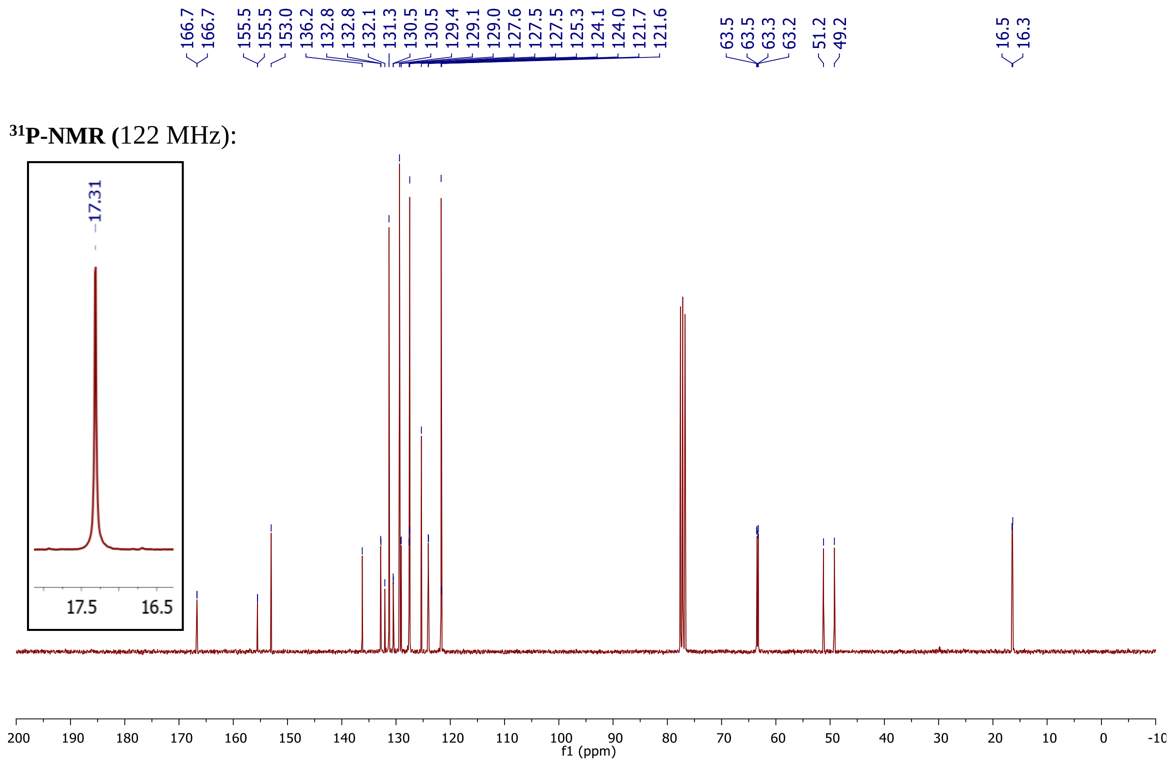

^{1}H-NMR (CDCl$_3$, 300 MHz) of (*S*)-**11mA**

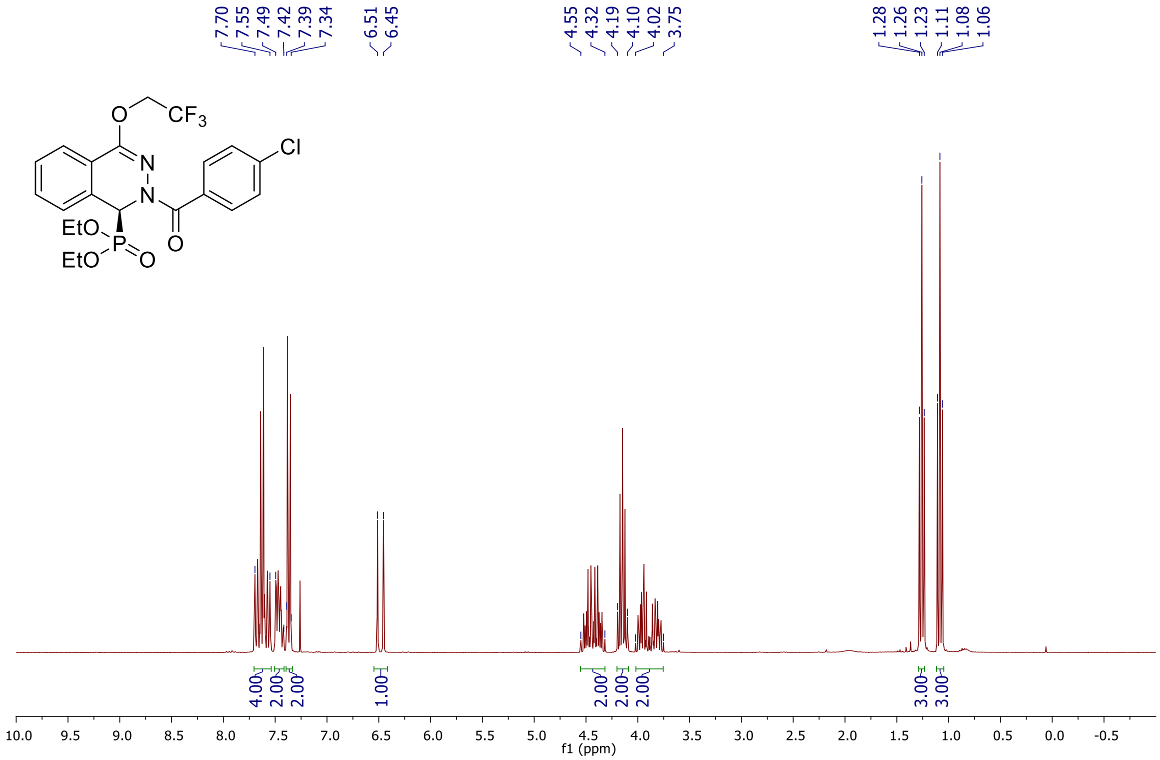

^{13}C-NMR (CDCl$_3$, 75.5 MHz) of (*S*)-**11mA**

^{31}P-NMR (122 MHz):

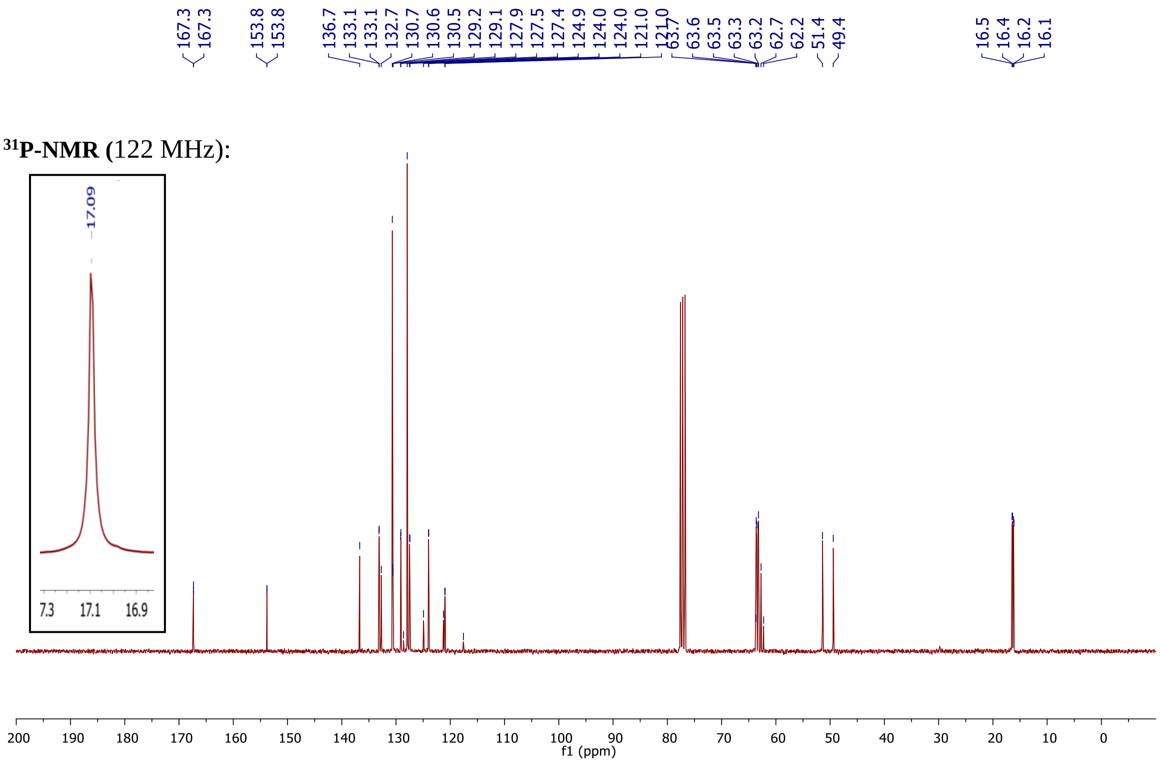

^{1}H-NMR (CDCl$_3$, 300 MHz) of (*S*)-**11nA**

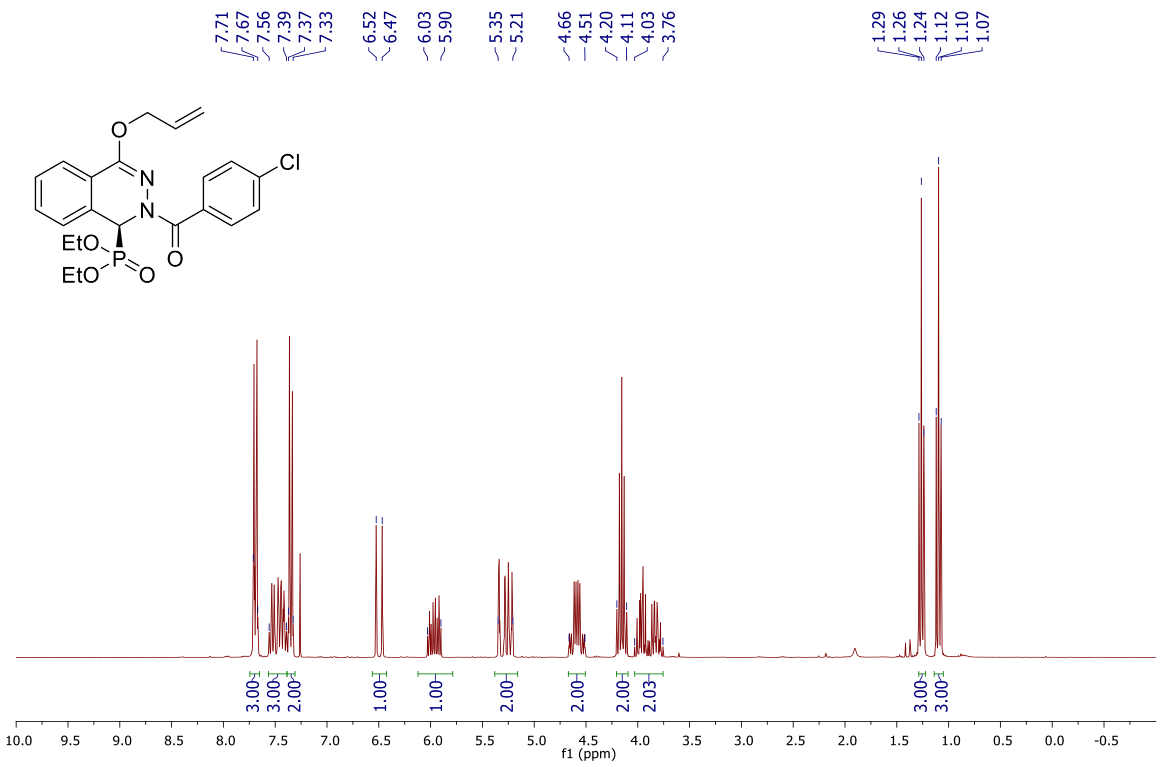

^{13}C-NMR (CDCl$_3$, 75.5 MHz) of (*S*)-**11nA**

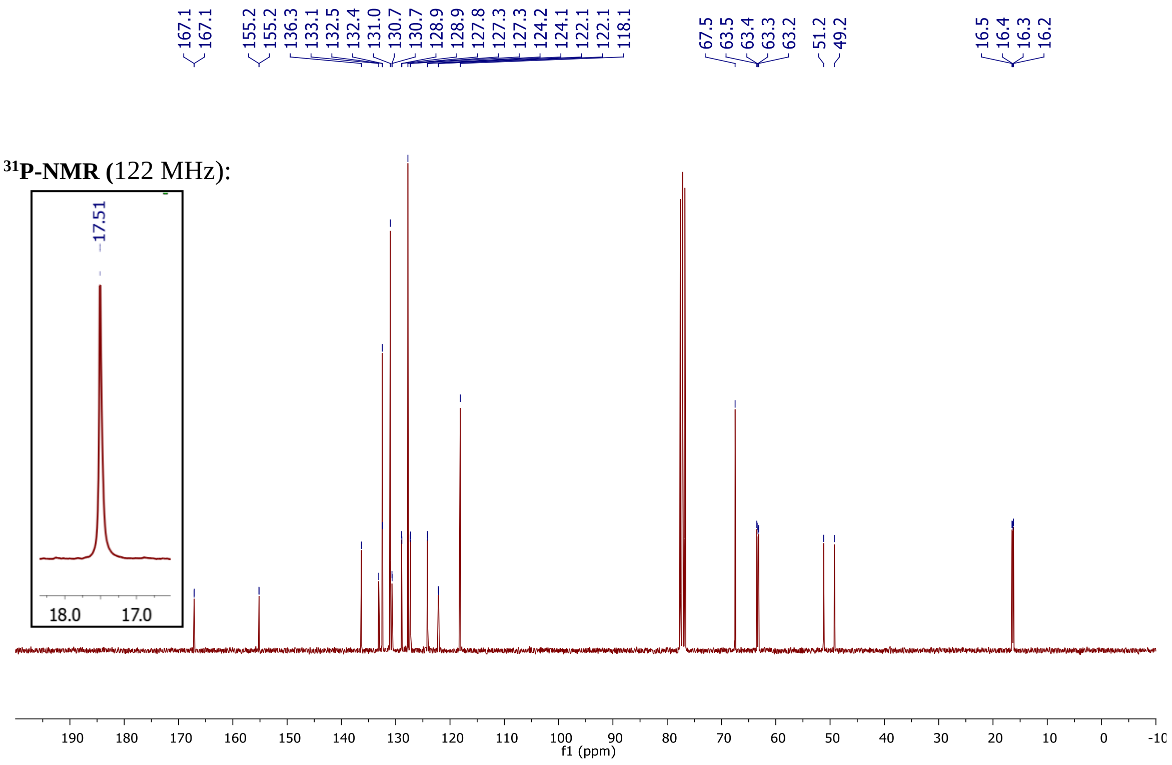

^{1}H NMR (CDCl$_3$, 300 MHz) of (*S*)-**11oA**

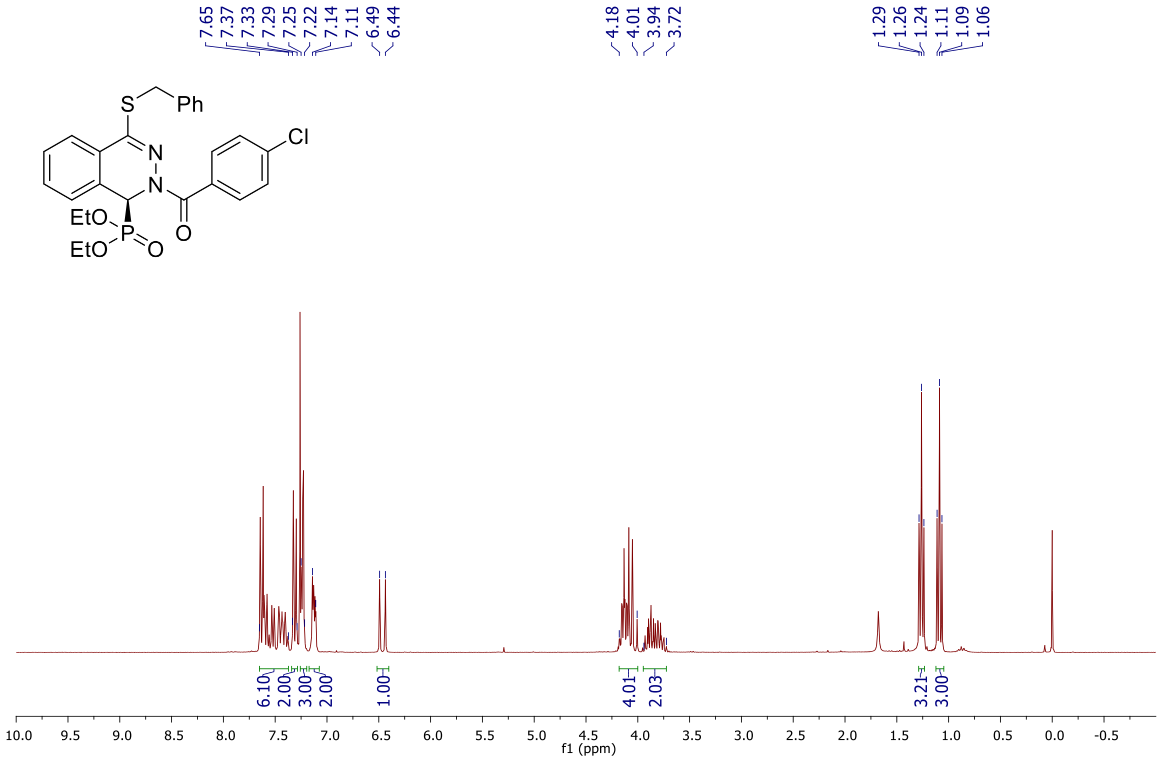

^{13}C NMR (CDCl$_3$, 75.5 MHz) of (*S*)-**11oA**

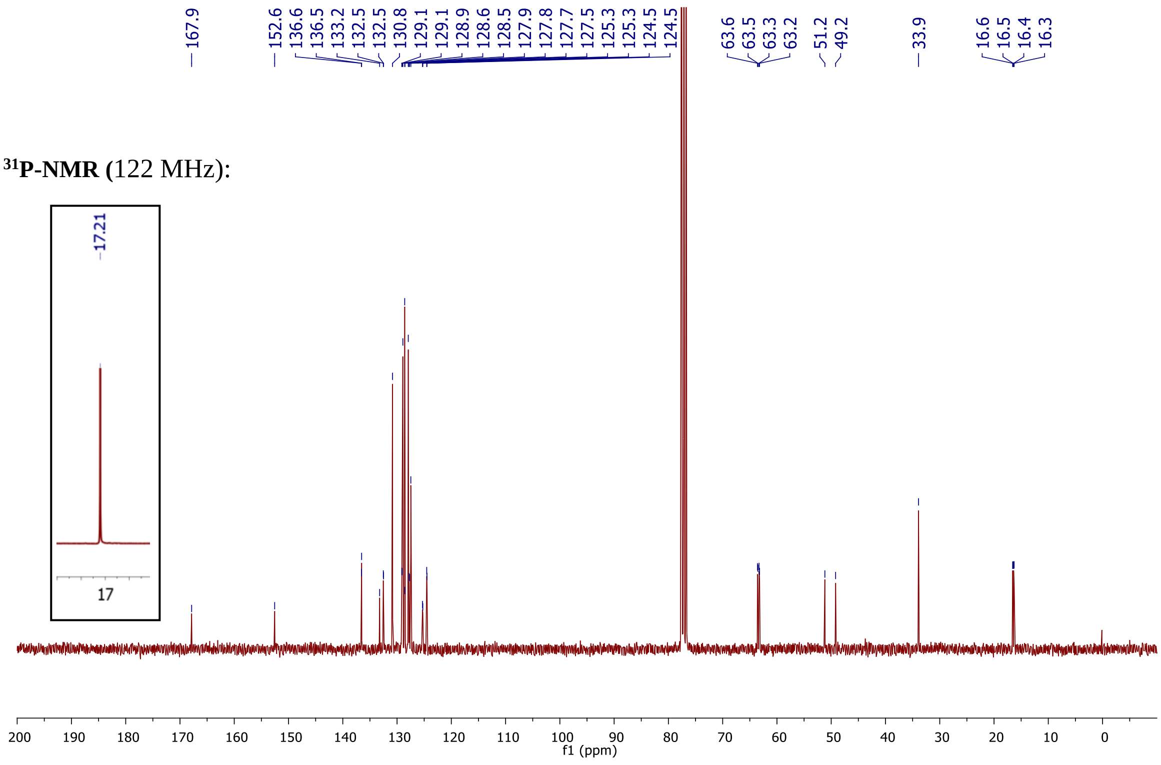

^{31}P-NMR (122 MHz):

[1]**H-NMR** (CDCl$_3$, 300 MHz) of (*S*)-**11pA**

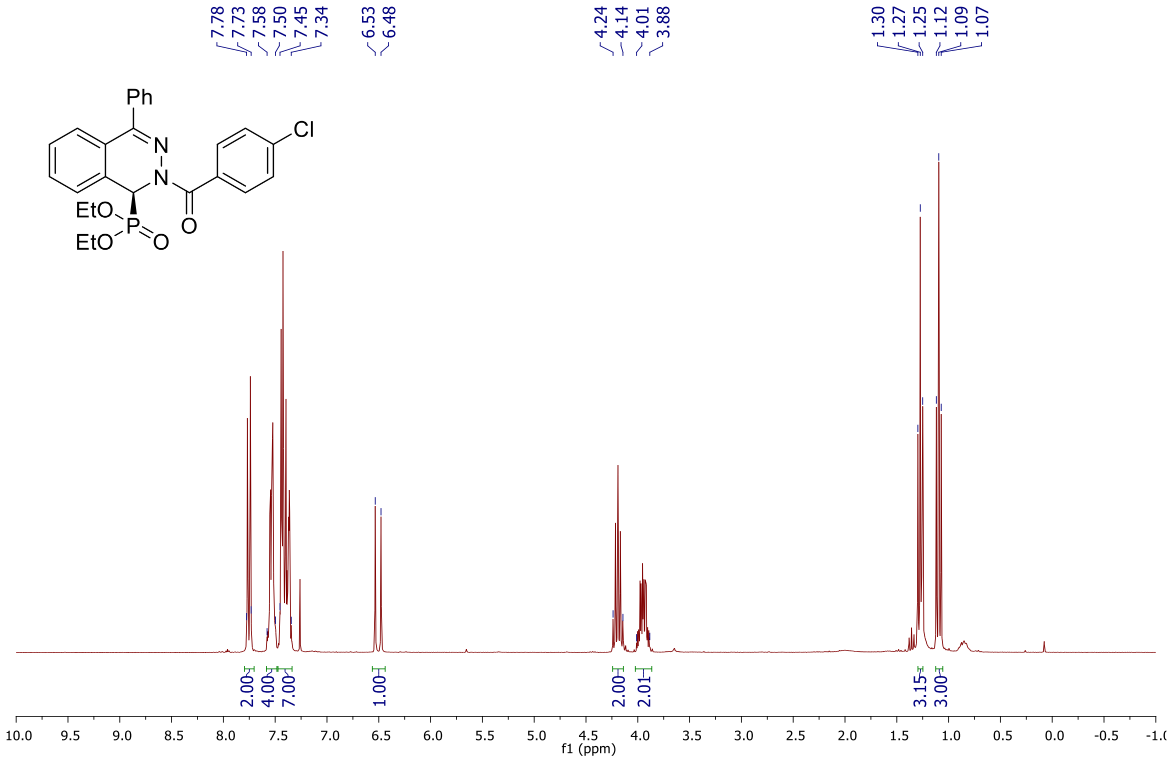

[13]**C-NMR** (CDCl$_3$, 75.5 MHz) of (*S*)-**11pA**

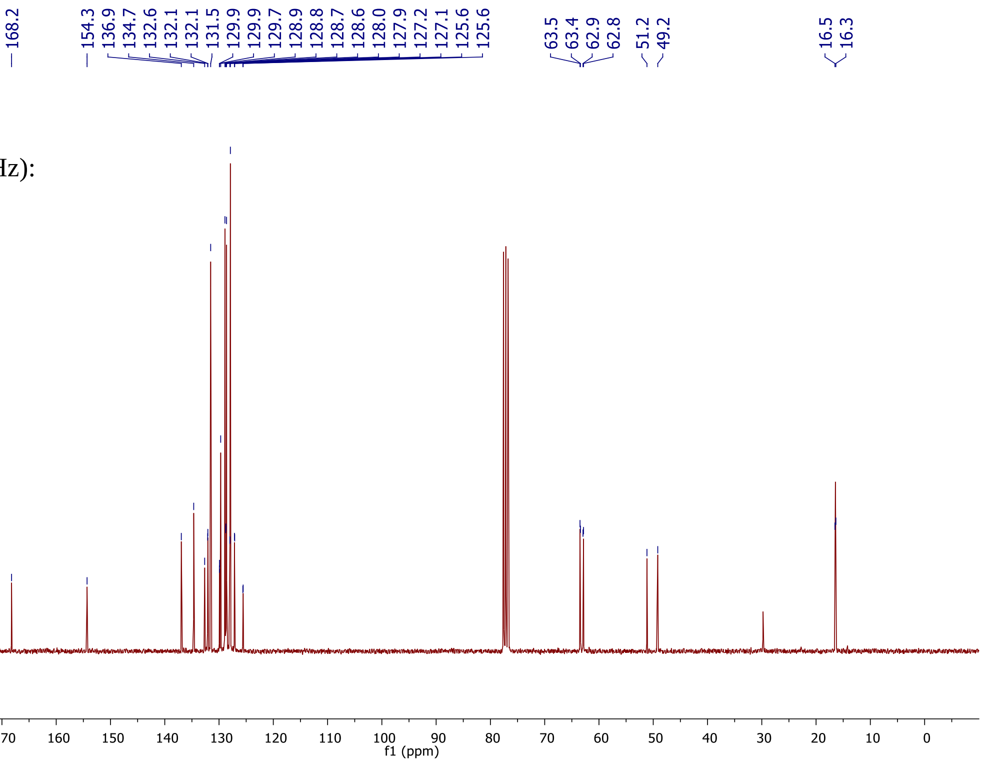

[31]**P-NMR (**122 MHz):

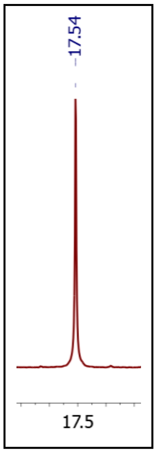

^{1}H-NMR ($CDCl_3$, 300 MHz) of (*S*)-**11qA**

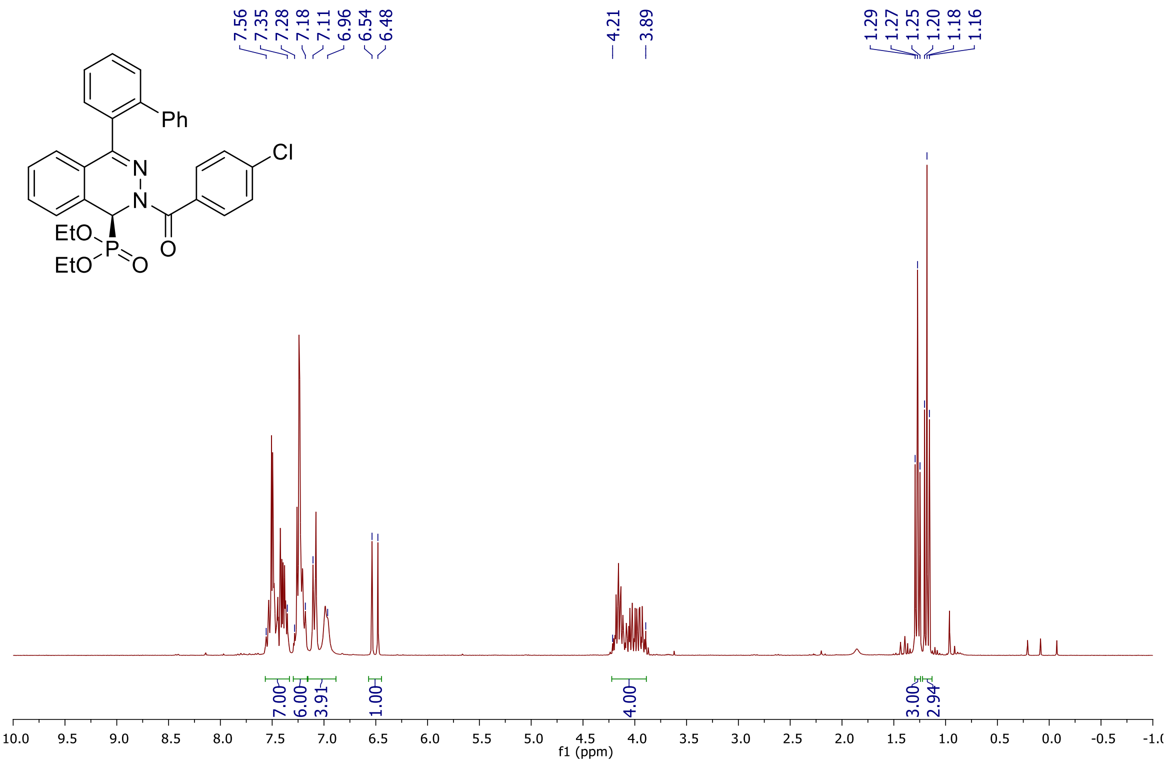

^{13}C-NMR ($CDCl_3$, 75.5 MHz) of (*S*)-**11qA**

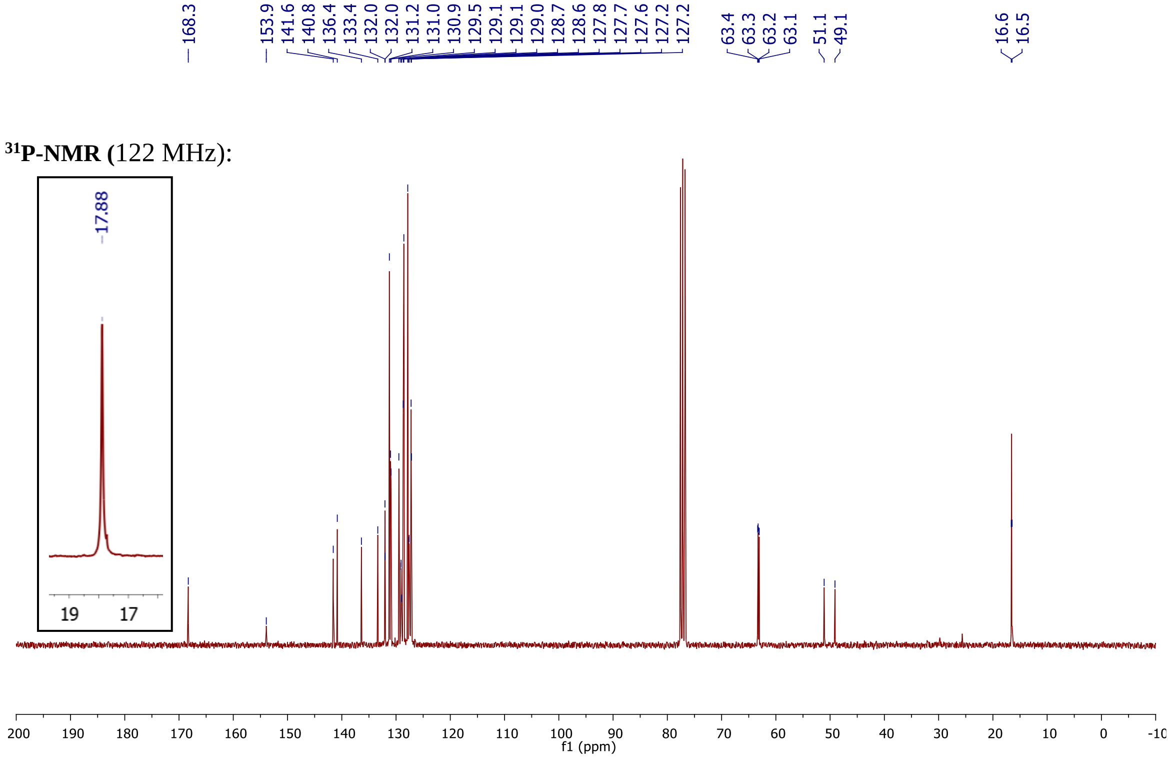

^{1}H NMR (CDCl$_3$, 300 MHz) of (*S*)-**11rA**

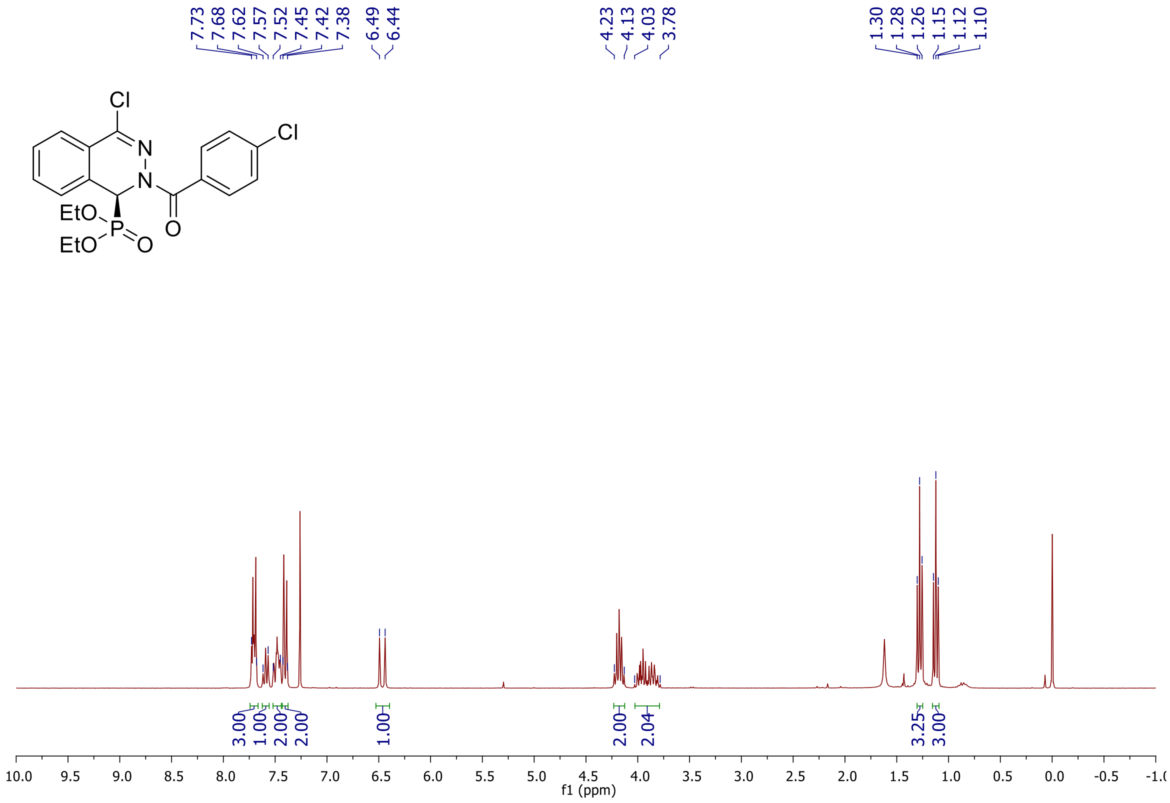

^{13}C NMR (CDCl$_3$, 126 MHz) of (*S*)-**11rA**

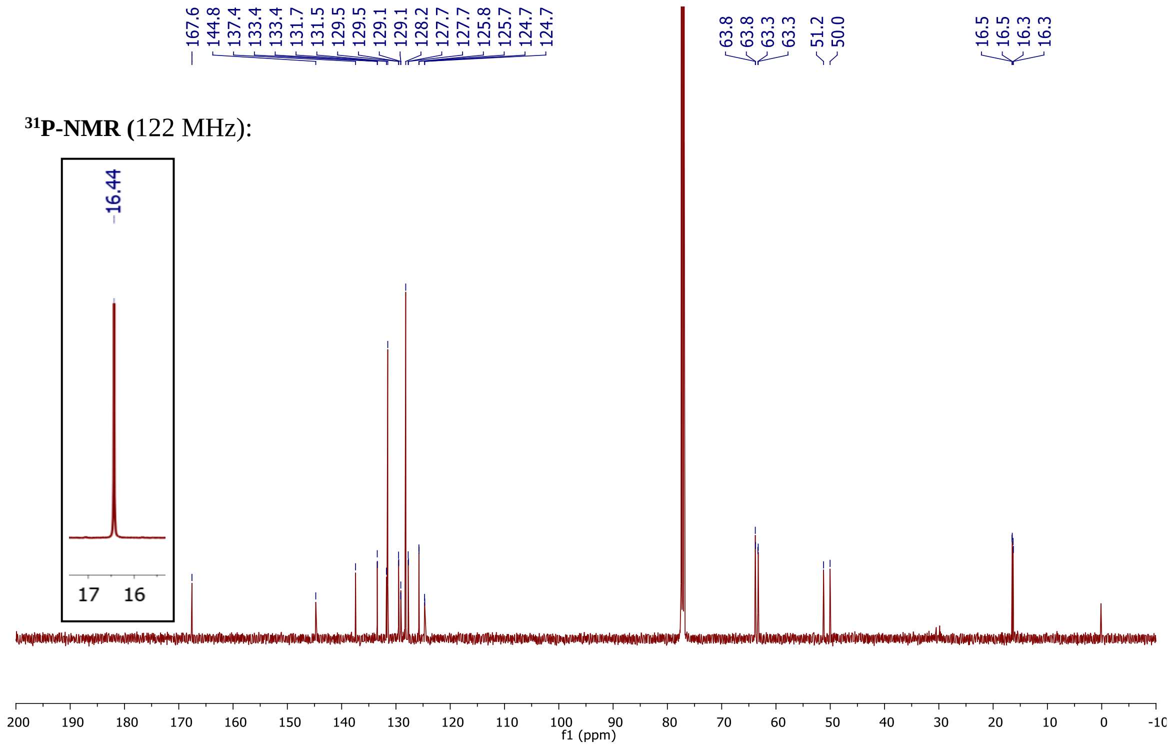

^{1}H NMR (CDCl$_3$, 300 MHz) of (*S*)-**11sA**

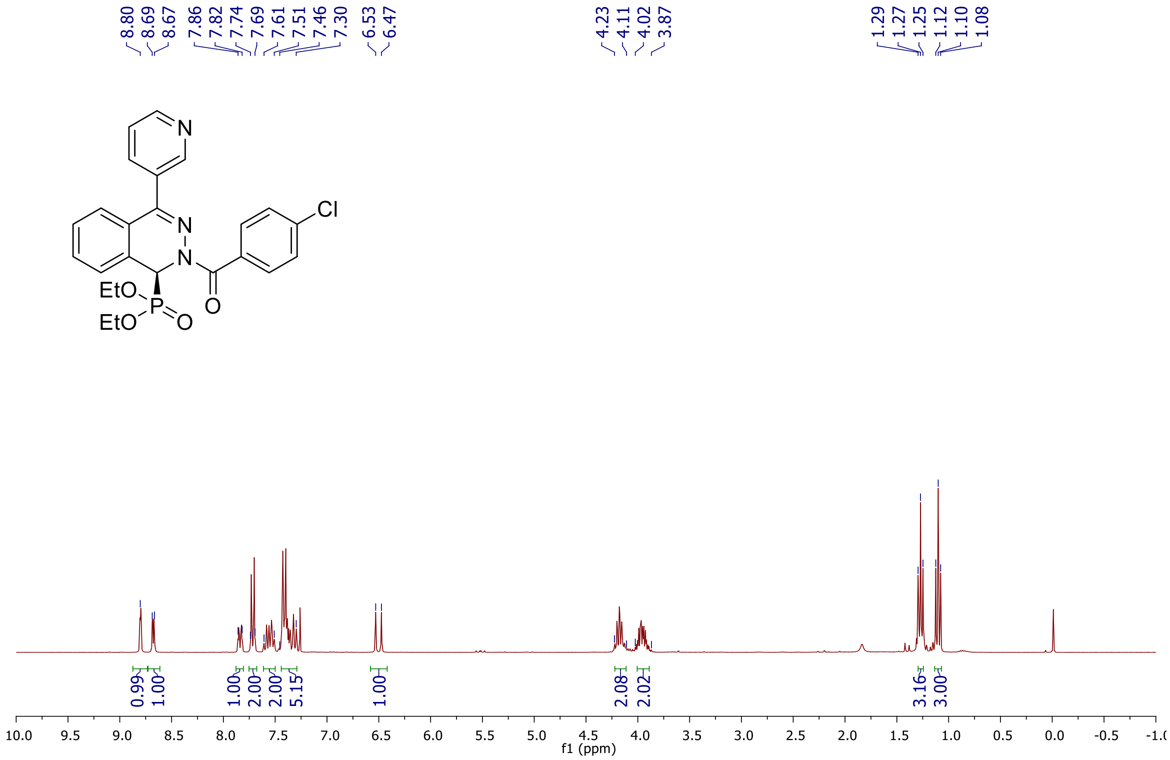

^{13}C NMR (CDCl$_3$, 75.5 MHz) of (*S*)-**11sA**

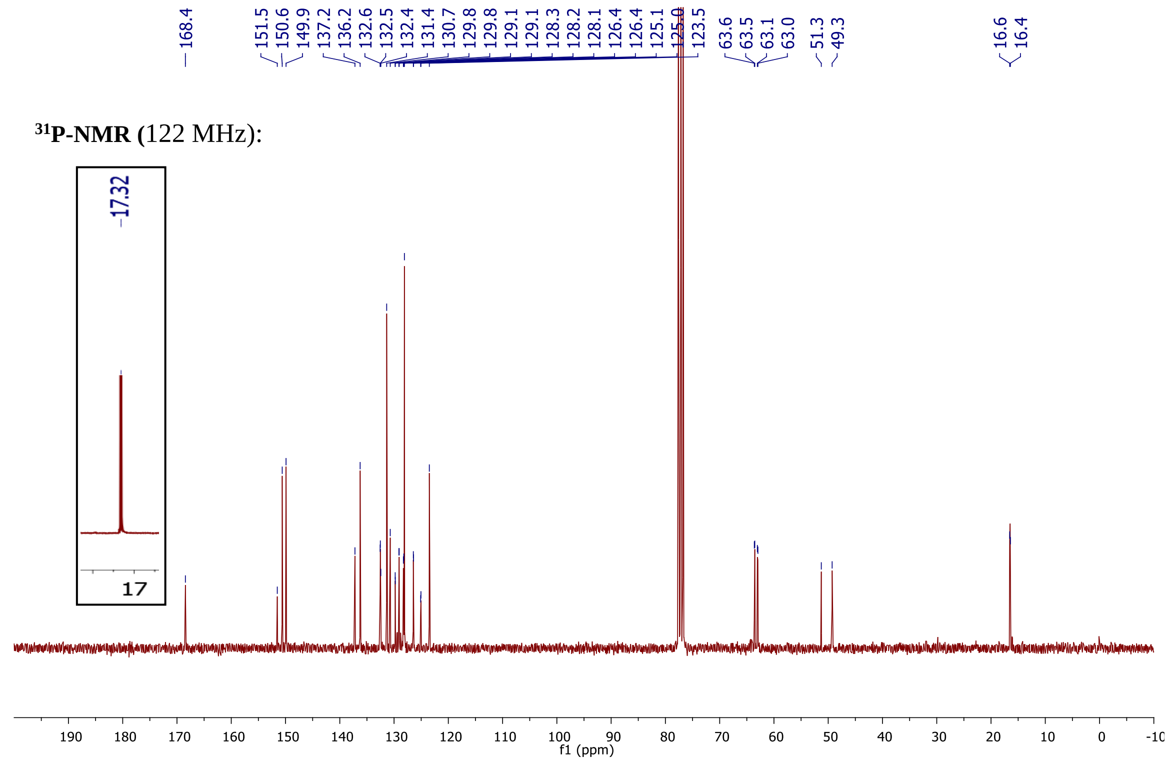

[1]**H-NMR** (CDCl$_3$, 300 MHz) of (*S*)-**11tA**

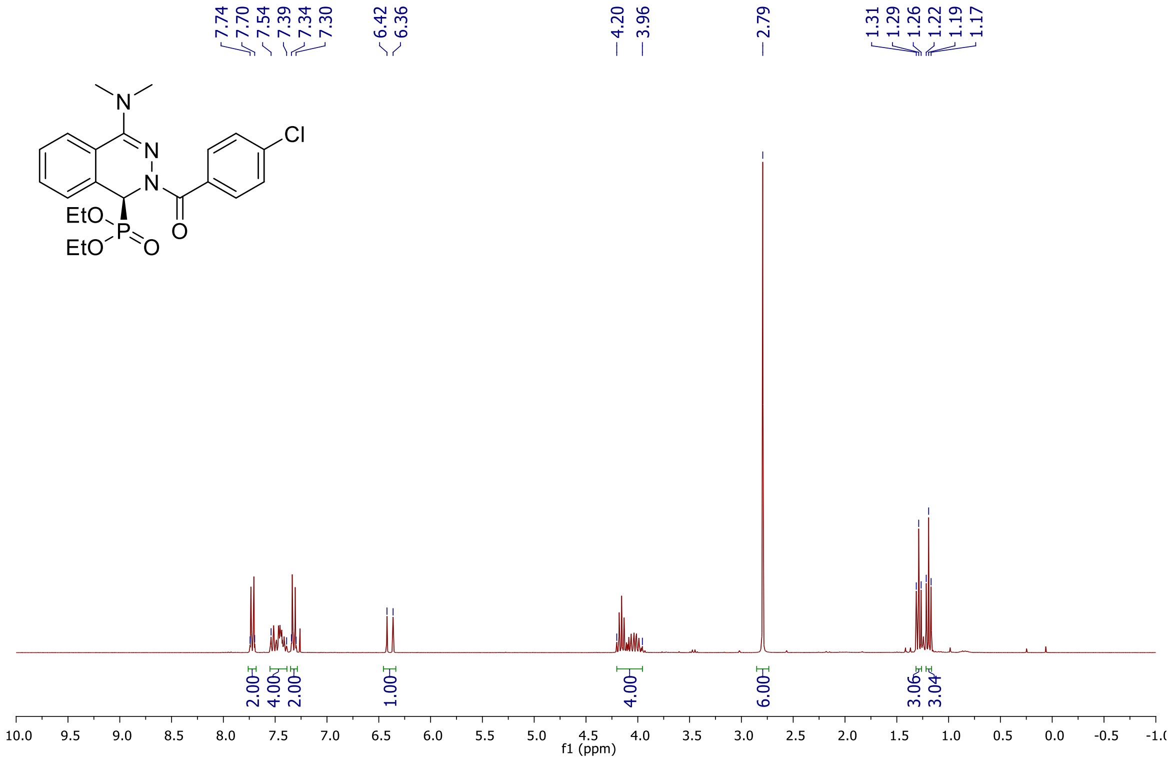

[13]**C-NMR** (CDCl$_3$, 75.5 MHz) of (*S*)-**11tA**

[31]**P-NMR (**122 MHz):

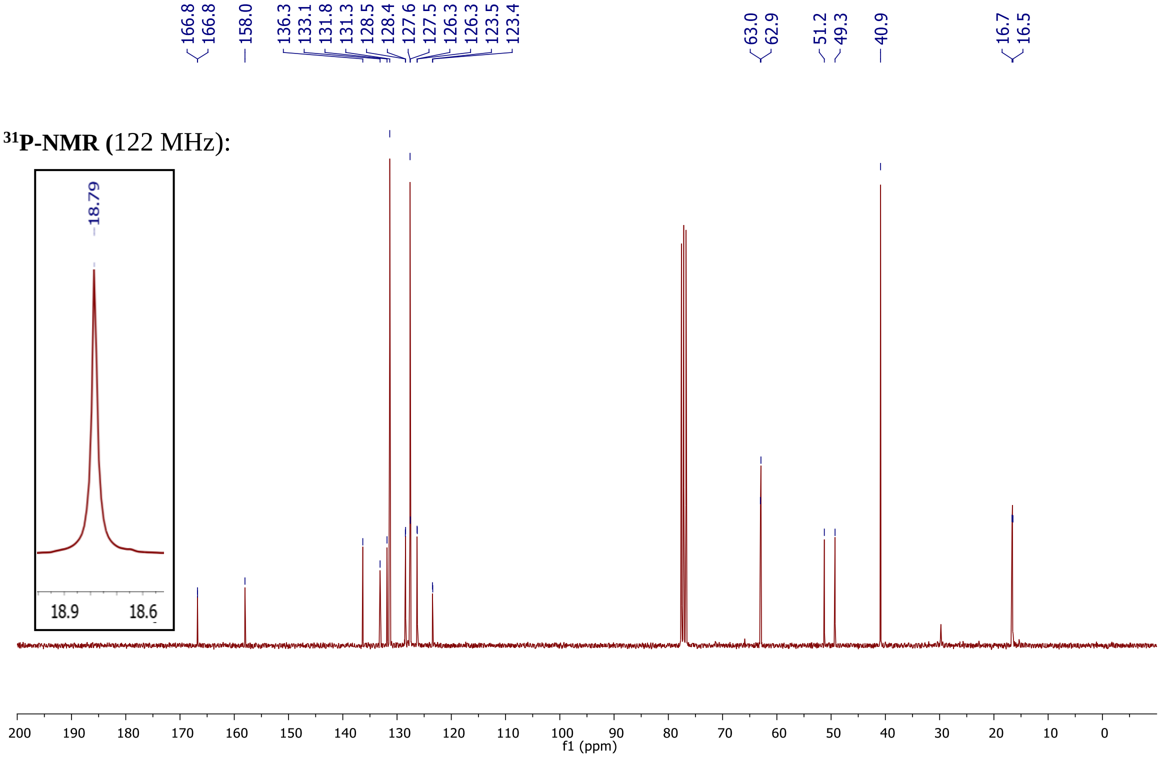

^{1}H NMR (CDCl$_3$, 300 MHz) of (*S*)-**11uA**

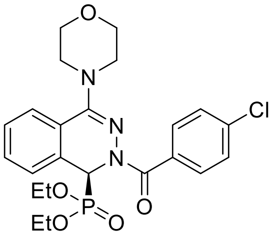

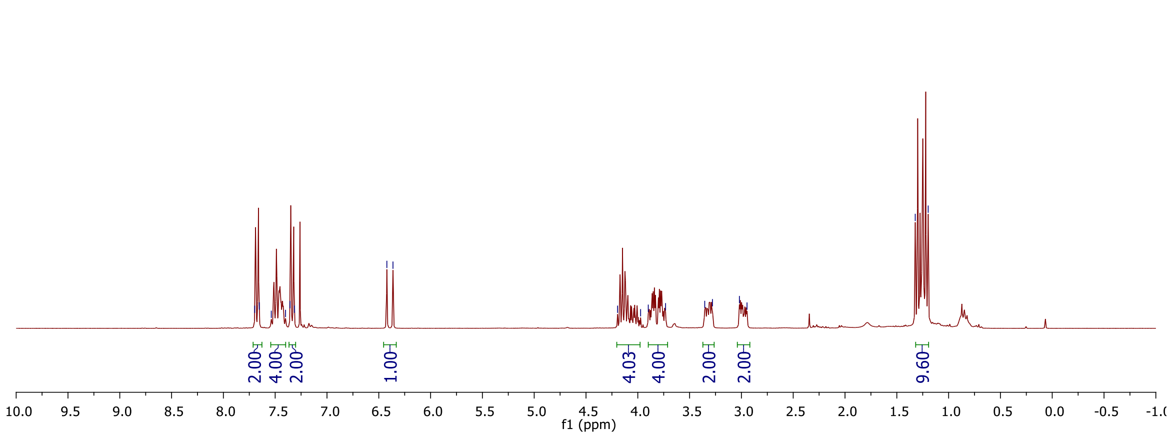

^{13}C NMR (CDCl$_3$, 75.5 MHz) of (*S*)-**11uA**

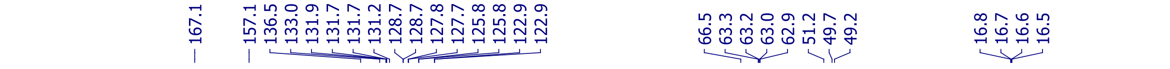

^{31}P-NMR (122 MHz):

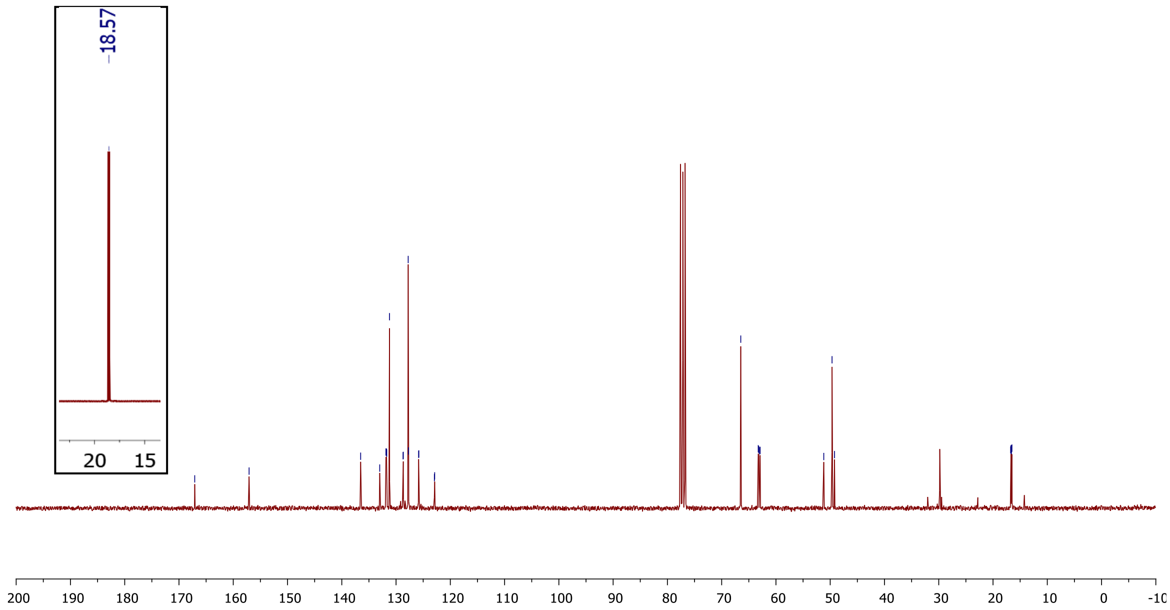

^{1}H-NMR (CDCl$_3$, 300 MHz) of (*S*)-**11vA**

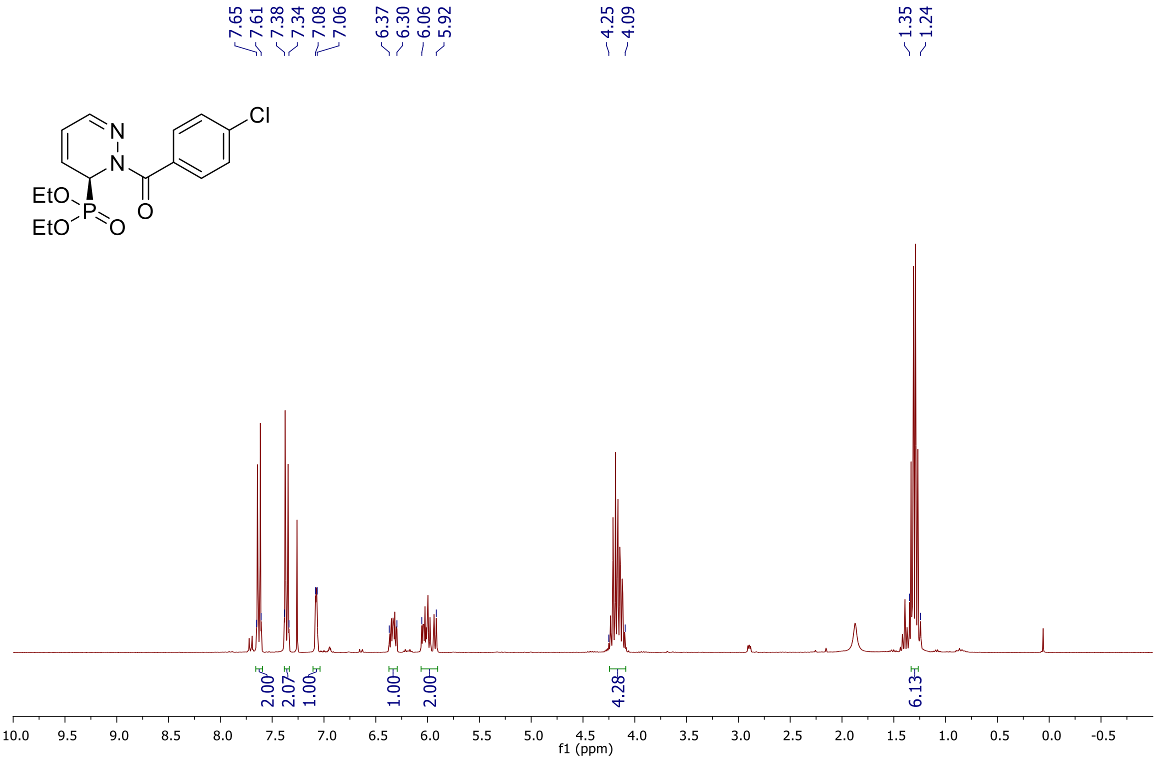

^{13}C-NMR (CDCl$_3$, 75.5 MHz) of (*S*)-**11vA**

^{31}P-NMR (122 MHz):

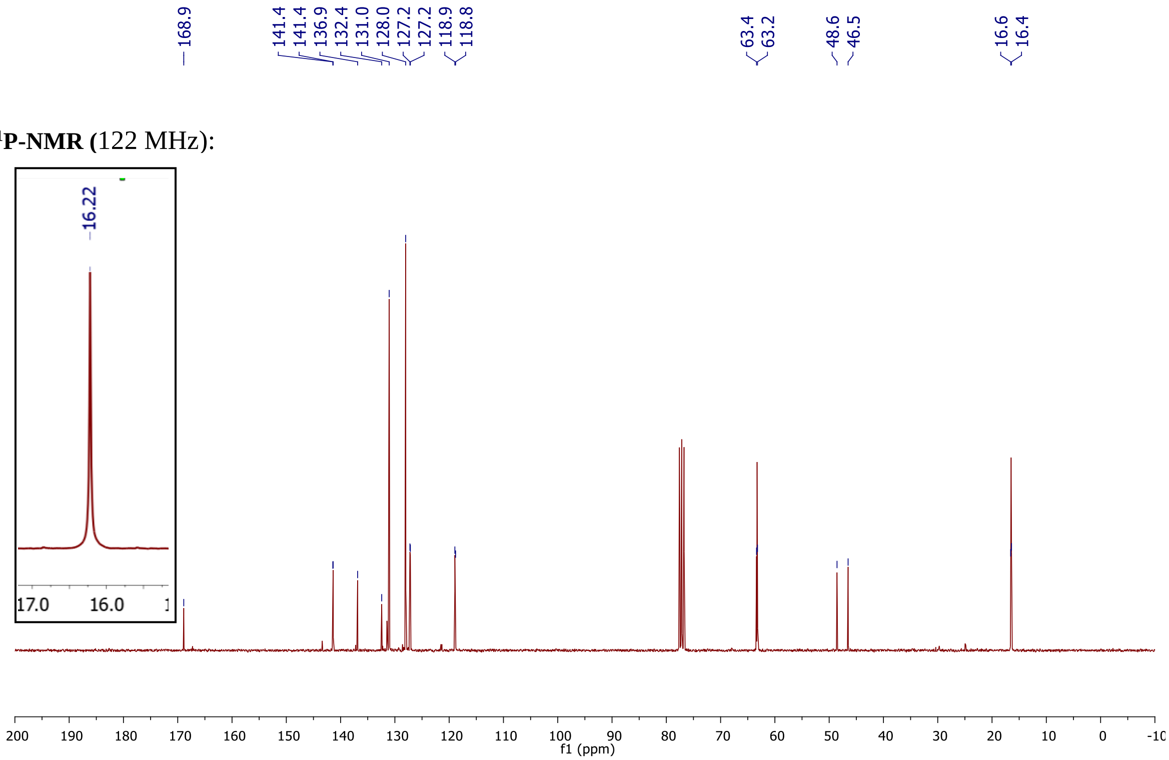

^{1}H-NMR (Acetone-d_6, 300 MHz) of (*S*)-**11wA**

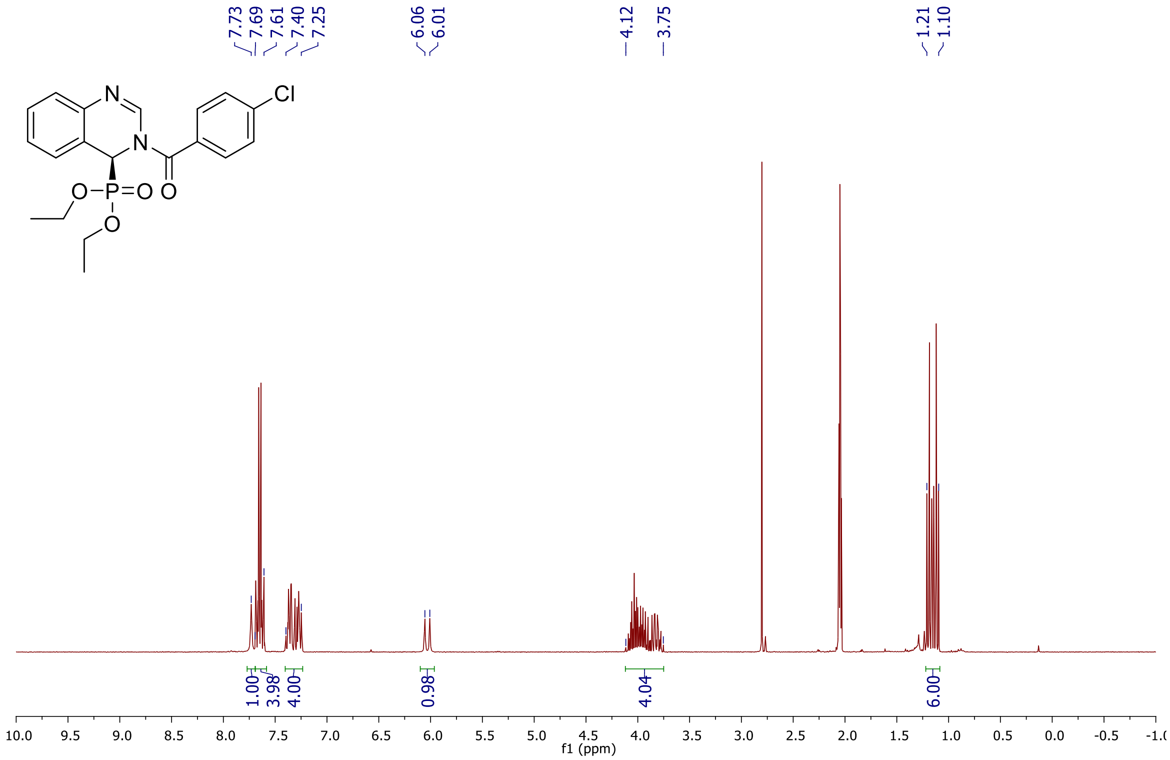

^{13}C-NMR (Acetone-d_6, 75.5 MHz) of (*S*)-**11wA**

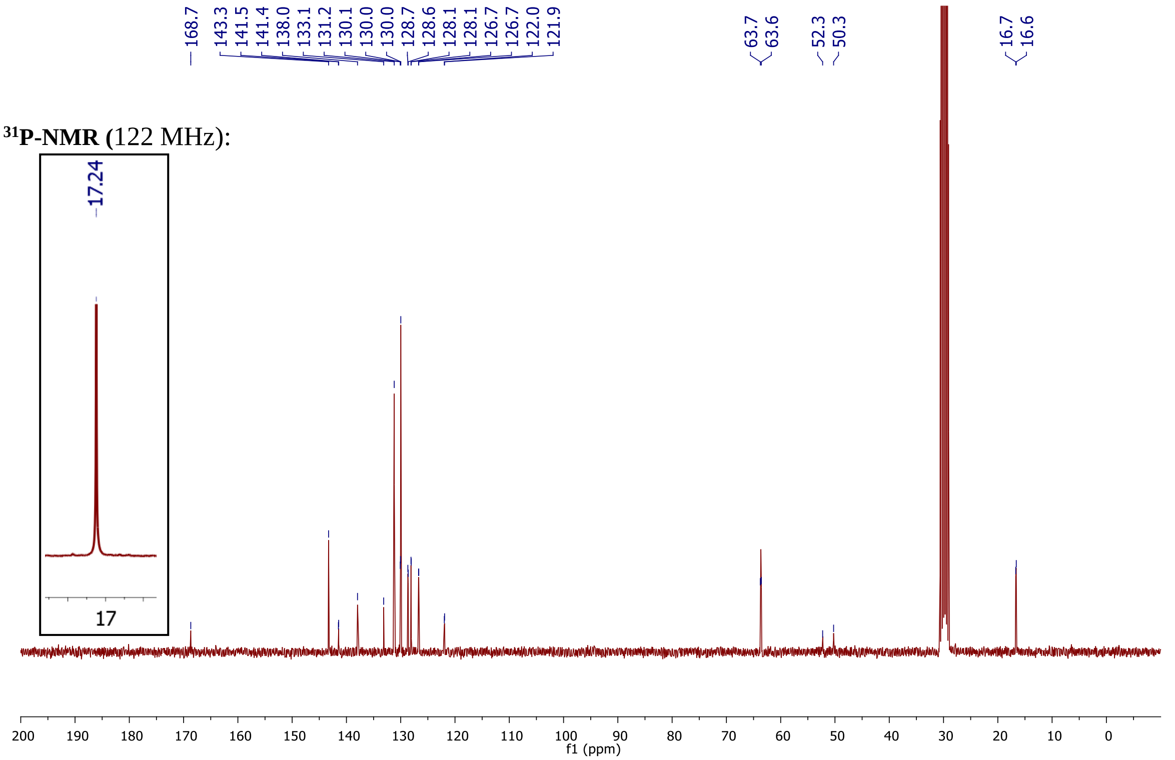

NOESY (CDCl$_3$, 500 MHz) of (*S*)-**11wA**

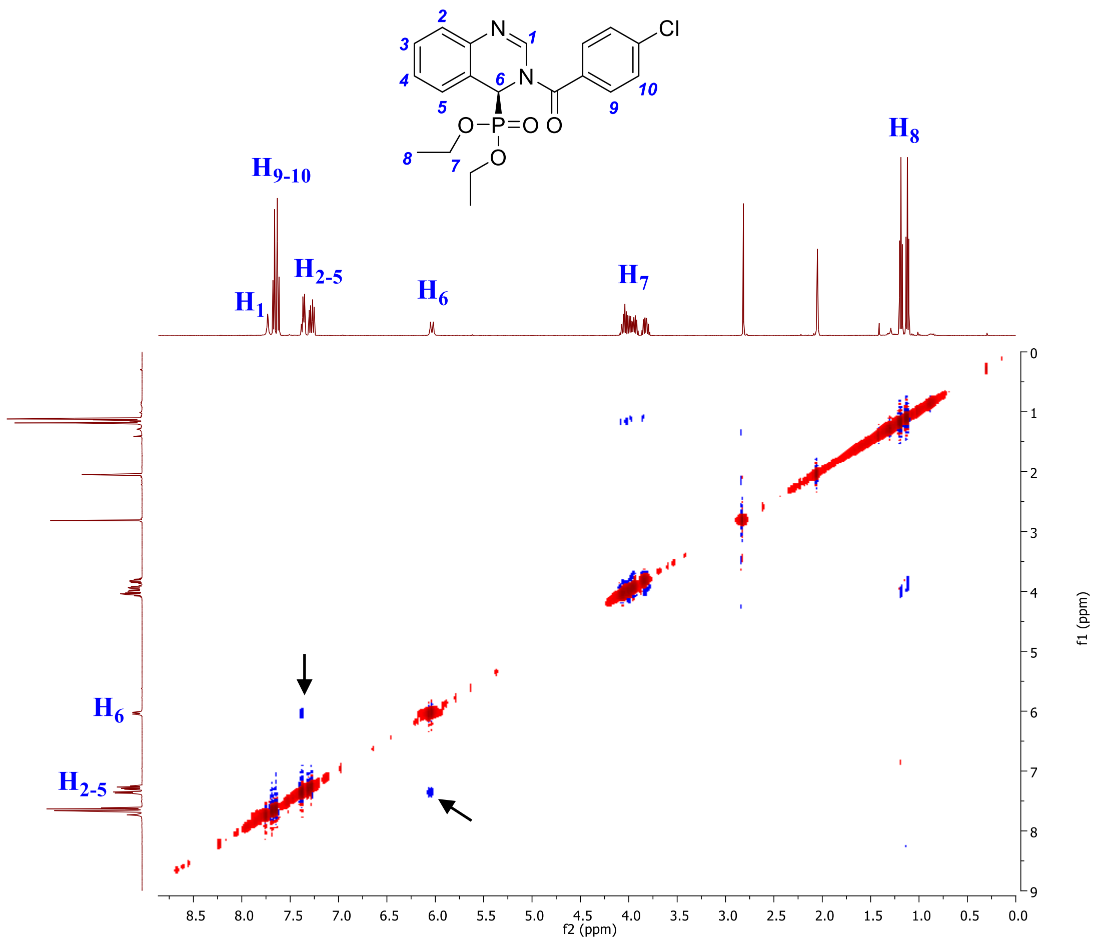

[1]**H-NMR** ($CDCl_3$, 300 MHz) of (*S*)-**11xA**

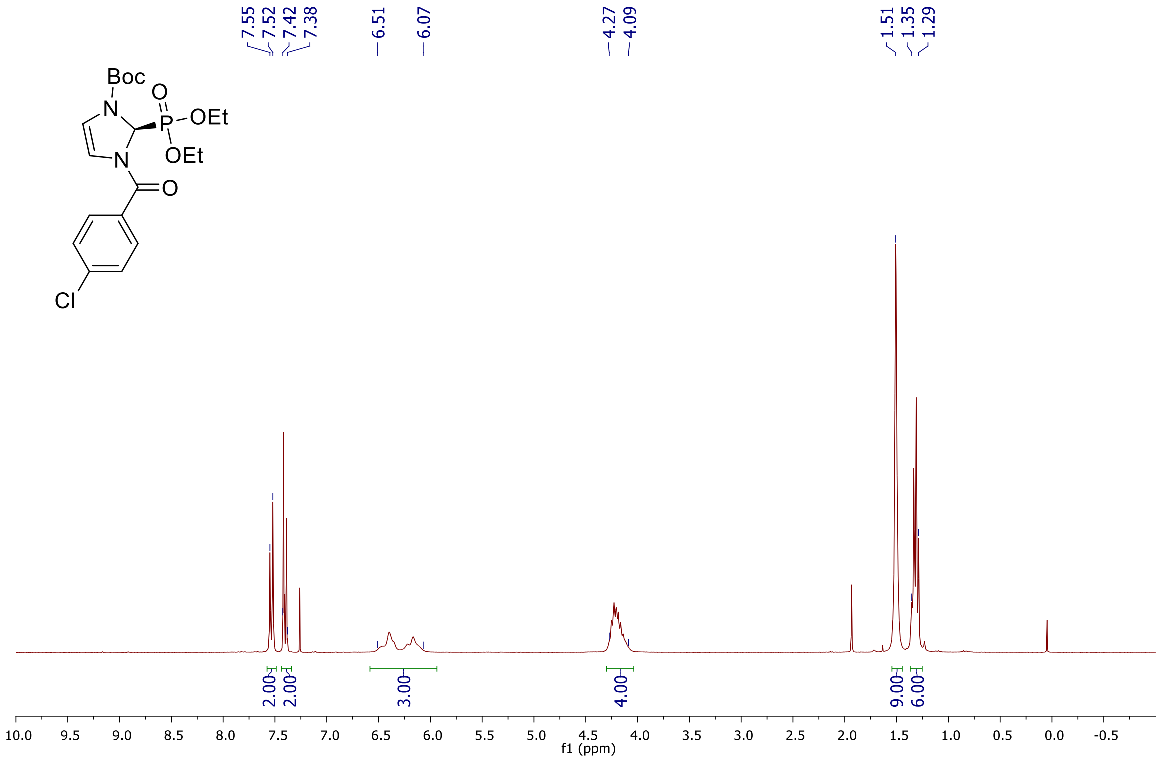

[13]**C-NMR** ($CDCl_3$, 75.5 MHz) of (*S*)-**11xA**

[31]**P-NMR** (122 MHz):

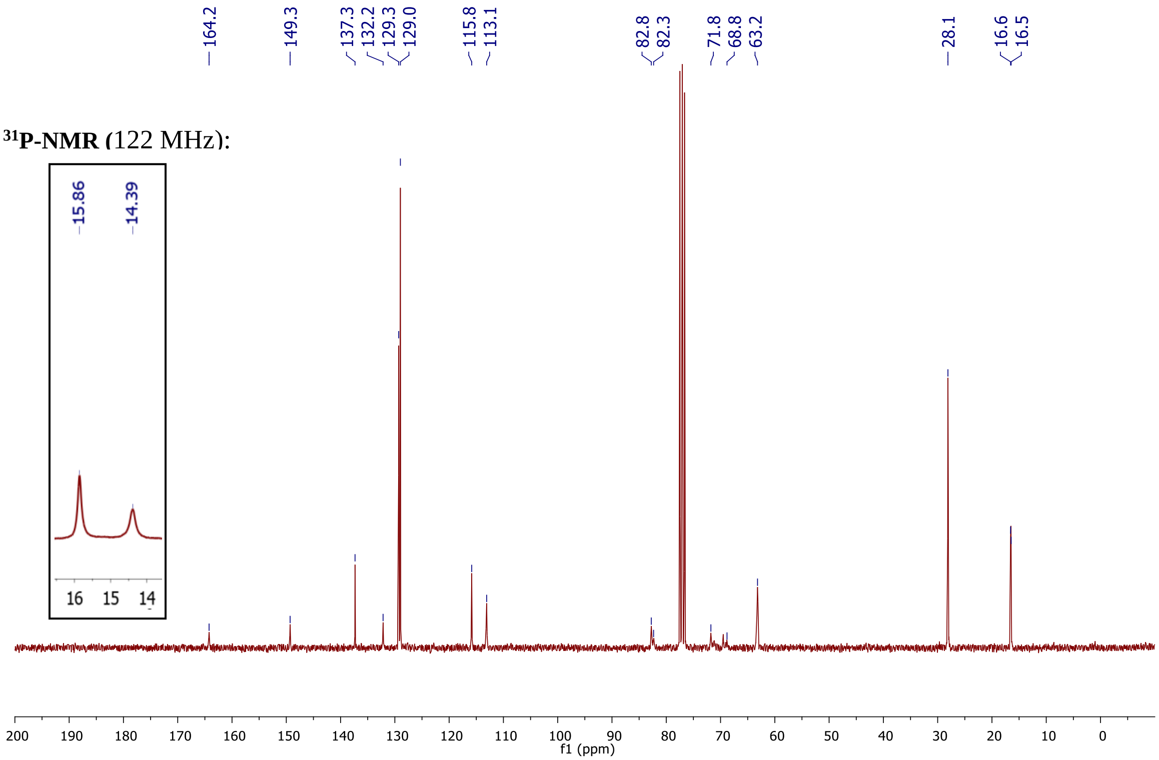

^{1}H NMR (CDCl$_3$, 300 MHz) of (*S*)-**11yA**

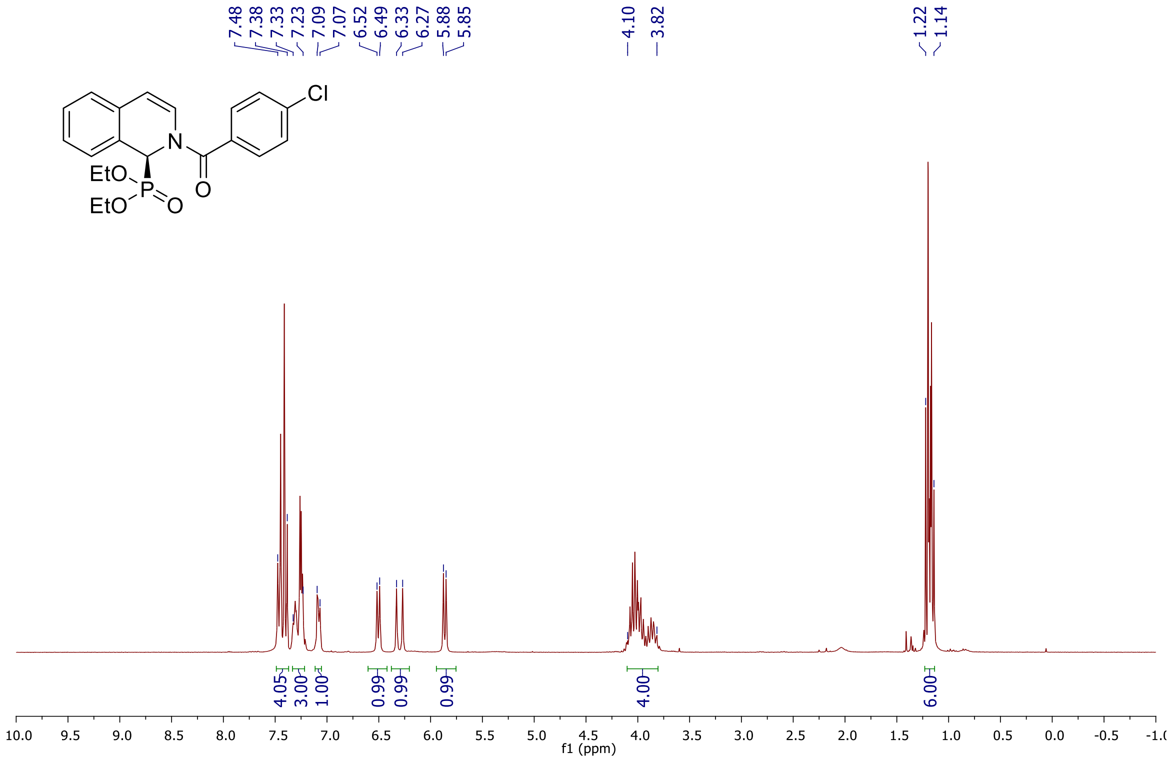

^{13}C NMR (CDCl$_3$, 75.5 MHz) of (*S*)-**11yA**

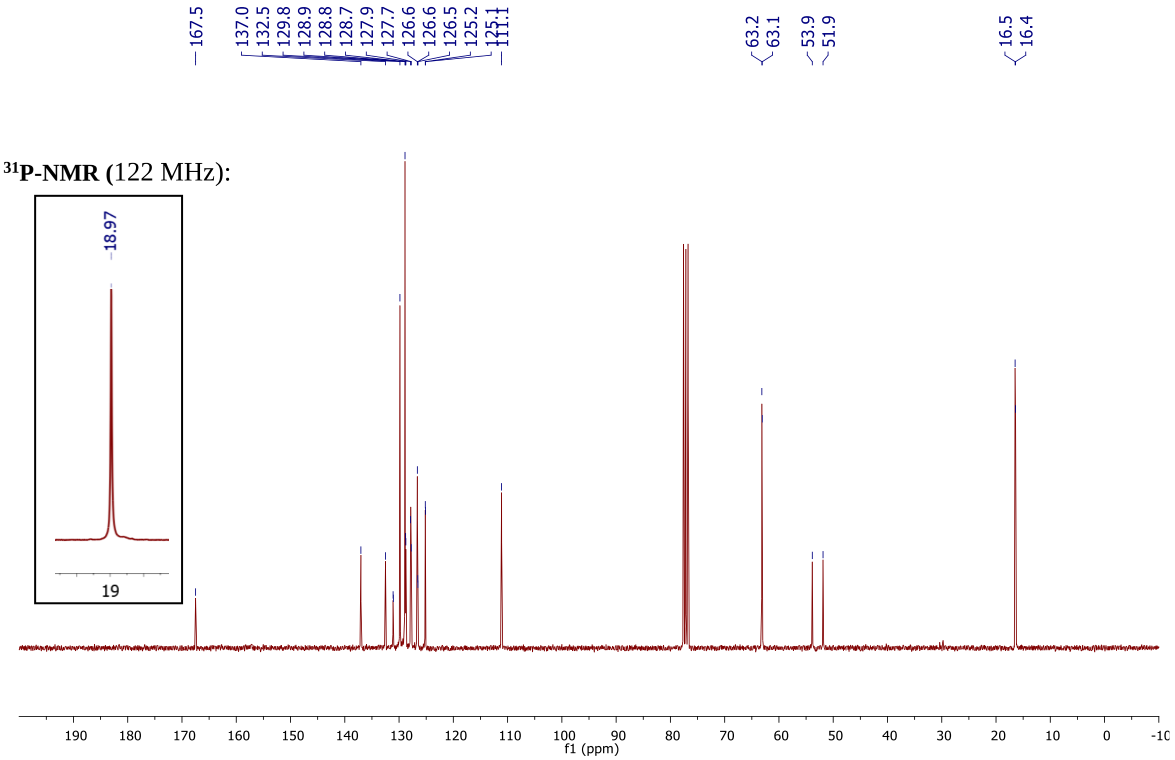

^{1}H-NMR (Acetone-d_6, 300 MHz) of (*S*)-**16**

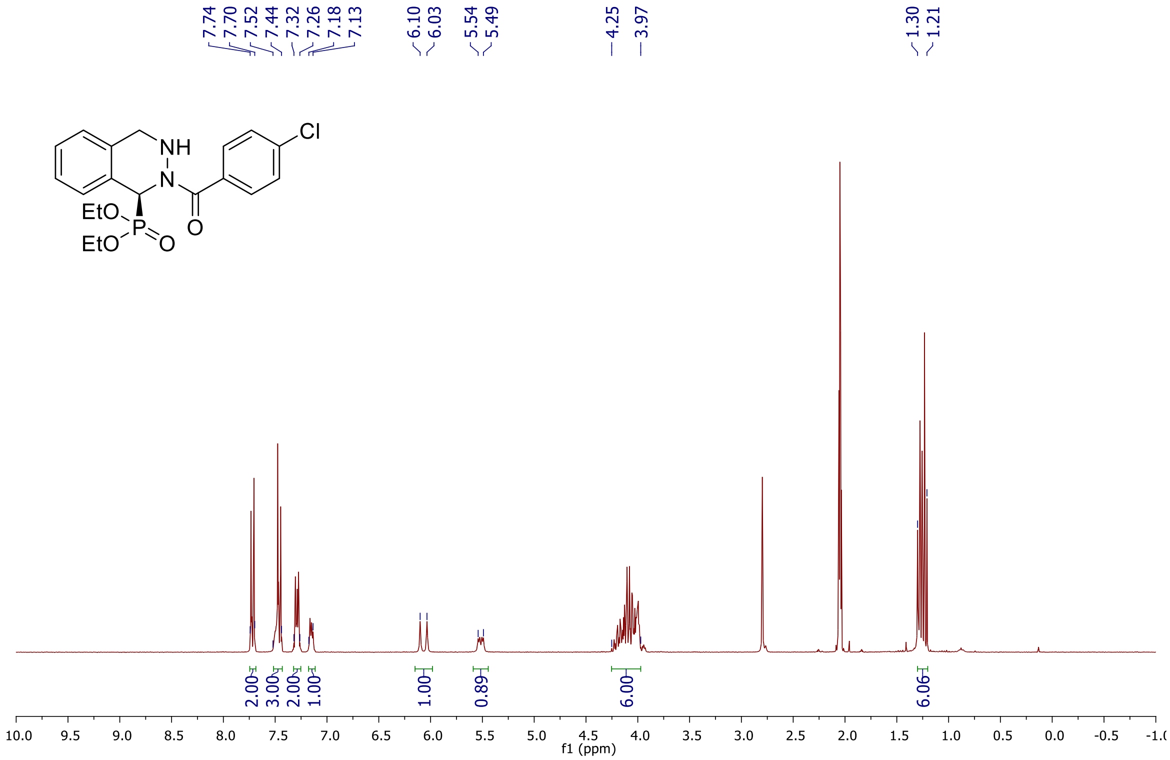

^{13}C-NMR (Acetone-d_6, 75.5 MHz) of (*S*)-**16**

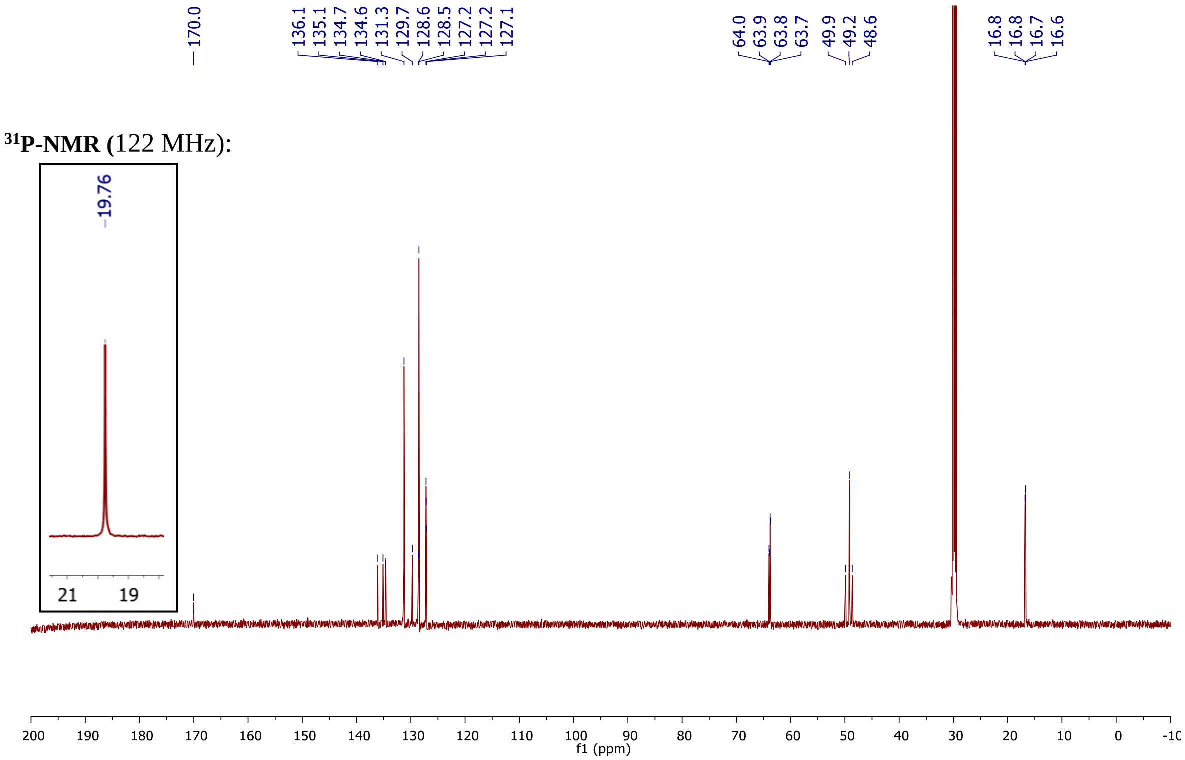

^{1}H-NMR (Acetone-d_6, 300 MHz) of (*S*)-**17**

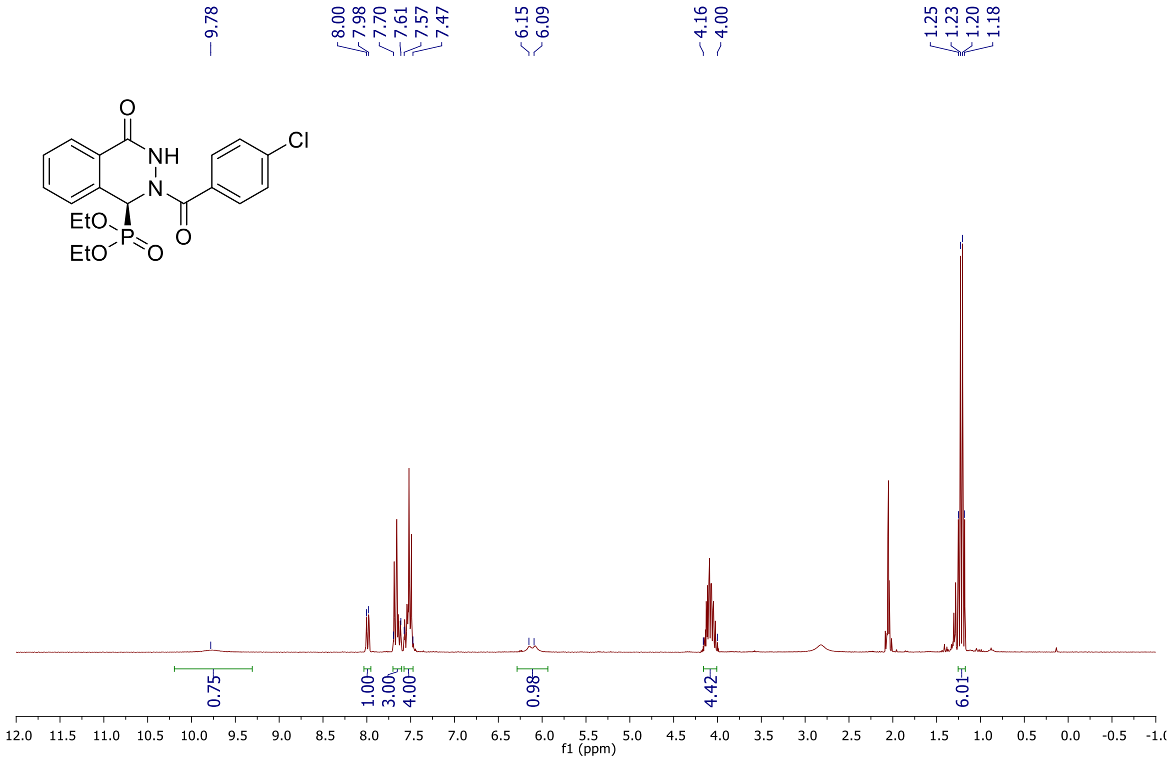

^{13}C-NMR (Acetone-d_6, 75.5 MHz) of (*S*)-**17**

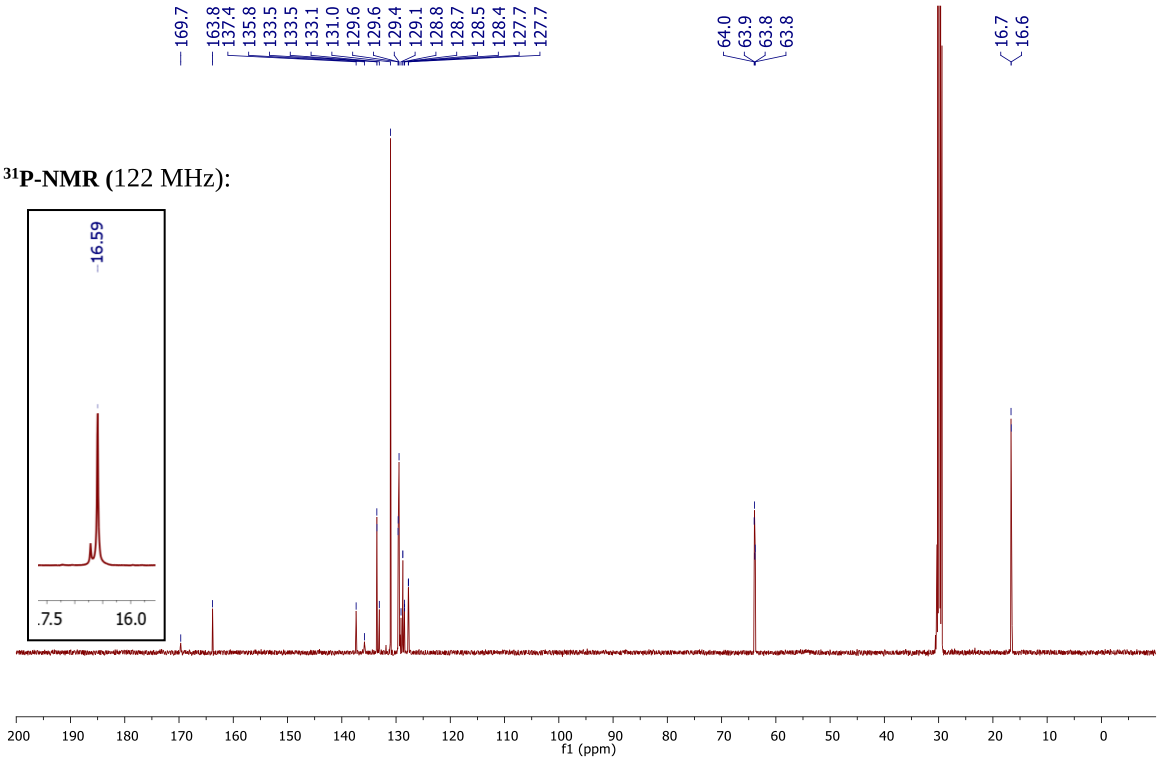

[1]**H-NMR** (DMSO-d_6, 300 MHz) of (*R*)-**18**

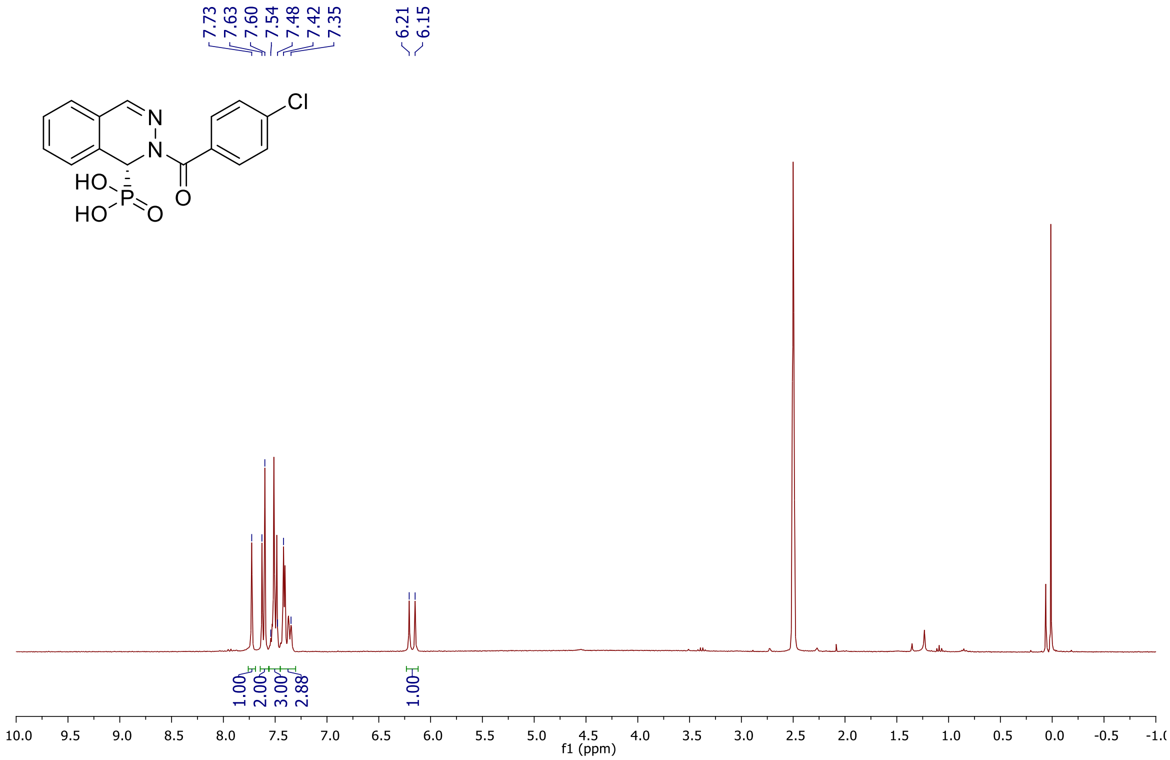

[13]**C-NMR** (DMSO- d_6, 126 MHz) of (*R*)-**18**

[31]**P-NMR (**122 MHz):

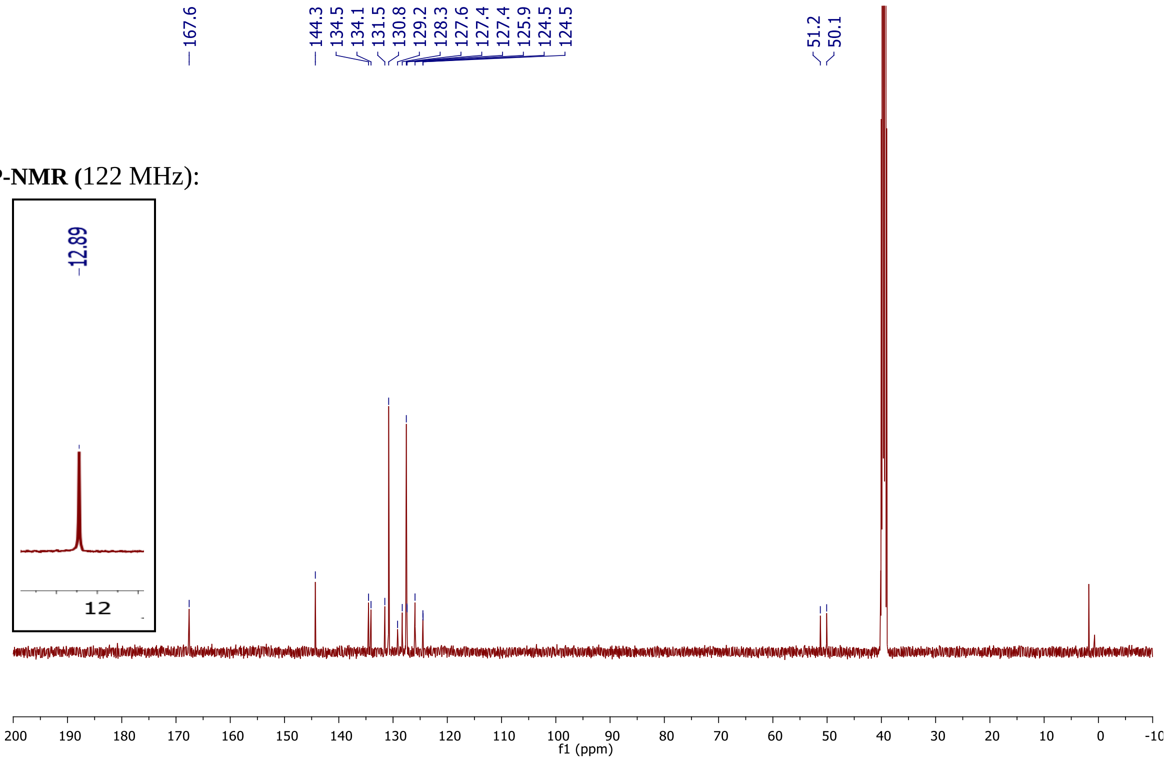

22. References.

[1]Montavon, T. J.; Türkmen Y. E.; Shamsi, N. A.; Miller C.; Sumaria C. S.; Rawal V. H.; Kozmin S. A. [2+2+2] Cycloadditions of siloxy alkynes with 1,2-diazines: from reaction discovery to identification of an antiglycolytic chemotype. *Angew. Chem. Int. Ed*. **2013**, *52*, 13576-13579.

[2]Kessler, S. N.; Wegner H. A. One-pot synthesis of phthalazines and pyridazino-aromatics: a novel strategy for substituted naphthalenes. *Org. Lett*. **2012**, *14*, 3268-3271.

[3]Hadsarung, R.; Thongnest, S.; Oekchuae, S.; Chaiyaveij, D.; Boonsombat, J.; Ruchirawat, S. Facile synthesis of 1-substituted 4-H phthalazine, a versatile scaffold for chemically diverse phthalazines. *Tetrahedron* **2022**, *121*, 132920.

[4]Velázquez, M.; Fernández, R.; Lassaletta, J. M.; Monge, D. Asymmetric dearomatization of phthalazines by anion-binding catalysis. *Org. Lett*. **2023**, *25*, 8797-8802.

[5]Lepri, S.; Ceccarelli, M.; Milani, N.; Tortorella, S.; Cucco, A.; Valeri, A.; Goracci, L.; Brink, A.; Cruciani, G. Structure-metabolism relationships in human-AOX: Chemical insights from a large database of aza-aromatic and amide compounds. PNAS, **2017**, *114*, E3177-E3187.

[6]Chang, S-M.; Jain, V.; Chen, T-L.; Patel, A. S.; Pidugu, H. B.; Lin, Y-W.; Wu, M-H.; Huang, J-R.; Wu, H-C.; Shah, A.; Su, T-L.; Lee, T-C. Design and synthesis of 1,2-bis(hydroxymethyl)pyrrolo[2,1-*a*]phthalazine hybrids as potent anticancer agents that inhibit angiogenesis and induce DNA interstrand cross-links. *J. Med. Chem.* **2019**, *62*, 2404-2418.

[7]Gurvey, A.; Schuster, F.; Herrmann, L.; Hahn, F.; Wangen, C.; Hodek, J.; Weber, J.; Marschall, M.; Tsogoeva, S. V. Light-driven catalyst-free access to phthalazines: entry to antiviral model drugs by merging domino reactions. *Eur. J. Org. Chem.* **2023**, *26*, e202200994.

[8]Kondoh, A.; Aoki, T.; Terada, M. Organocatalytic arylation of α-ketoesters based on umpolung strategy: phosphazene-catalyzed S_NAr reaction utilizing [1,2]-phospha-brook rearrangement. *Chem. Eur. J.* **2018**, *24*, 13110-13113.

[9]Pupo, G.; Ibba, F.; Ascough, D. M. H.; Vacini, A. C.; Ricci, P.; Christense, K. E.; Pfeifer, L.; Morphy, J. R.; Brown, J. M.; Paton, R. S.; Gouverneur, V. Asymmetric nucleophilic fluorination under hydrogen bonding phase-transfer catalysis. *Science*, **2018**, *360*, 638.

[10]Sibi, M. P.; Itoh, K. Organocatalysis in conjugate amine additions. synthesis of β-amino acid derivatives. *J. Am. Chem. Soc.* **2007**, *129*, 8064-8065.

[11]Brown, A. R.; Uyeda, C.; Brotherton, C. A.; Jacobsen, E. N. Enantioselective thiourea-catalyzed intramolecular Cope-type hydroamination. *J. Am. Chem. Soc.* **2013**, *135*, 6747-6749.

[12]Lehnherr, D.; Ford, D. D.; Bendelsmith, A. J.; Kennedy, C. R.; Jacobsen, E. N. Conformational control of chiral amido-thiourea catalysts enables improved activity and enantioselectivity. *Org. Lett.* **2016**, *18*, 3214-3217.

[13]E. Matador, E.; Iglesias-Sigüenza, J.; Monge, D.; Merino, P.; Fernández, R.; Lassaletta, J. M. Enantio- and diastereoselective nucleophilic addition of *N-tert*-butylhydrazones to isoquinolinium ions through anion-binding catalysis. *Angew. Chem. Int. Ed*. **2021**, *60*, 5096-5101.

[14]Reisman, S. E.; Doyle, A. G.; Jacobsen, E. N. Enantioselective thiourea-catalyzed additions to oxocarbenium ions. *J. Am. Chem. Soc.* **2008**, *130*, 7198-7199.

[15]Zuend, S. J.; Jacobsen, E. N. Mechanism of amido-thiourea catalyzed enantioselective imine hydrocyanation: transition state stabilization via multiple non-covalent interactions. *J. Am. Chem. Soc.* **2009**, *131*, 15358-15374.

[16]Matador, E.; Retamosa, M. G.; Monge, D.; Iglesias-Sigüenza, J.; Fernández, R.; Lassaletta, J. M. Bifunctional squaramide organocatalysts for the asymmetric addition of formaldehyde *tert*-butylhydrazone to simple aldehydes. *Chem. Eur. J.* **2018**, *24*, 6854-6860.

[17]Lalonde, M. P.; McGowan, M. A.; Rajapaksa, N. S.; Jacobsen, E. N. Enantioselective formal Aza-Diels–Alder reactions of enones with cyclic imines catalyzed by primary aminothioureas. *J. Am. Chem. Soc.* **2013**, *135*, 1891-1894.

[18]Zhao, C.; Seidel, D. Enantioselective A^3 reactions of secondary amines with a Cu(I)/acid–thiourea catalyst combination. *J. Am. Chem. Soc.* **2015**, *137*, 4650-4653.

[19]Bhushau-Tripathi, C.; Mukherjee, S. Catalytic enantioselective iodoaminocyclization of hydrazones. *Org. Lett.* **2014**, *16*, 3368-3371.

[20]Zhao, C.; Sojdak, C. A.; W. Myint, W.; Seidel, D. Reductive etherification via anion-binding catalysis. *J. Am. Chem. Soc.* **2017**, *139*, 10224-10227.

[21]Saito, Y.; Min-Cho, S.; Danieli, L. A.; Kobayashi, S. Zinc-catalyzed phosphonylation of alcohols with alkyl phosphites. *Org. Lett*. **2020**, *22*, 3171-3175.

[22]Wezenberg, S. J.; Feringa, B. Photocontrol of anion binding affinity to a bis-urea receptor derived from stiff-stilbene. *Org. Lett*. **2017**, *19*, 324-327.

[23]BindFit v0.5, available at http://app.supramolecular.org/bindfit/

[24]Howe, E. N. W.; Bhadbhade, M.; Thordarson, P. Cooperativity and complexity in the binding of anions and cations to tetratopic ion-pair host. *J. Am. Chem. Soc.* **2014**, *136*, 7505-7516.

[25]Case, D. A.; Aktulga, H. M.; Belfon, K.; Ben-Shalom, I. Y.; Berryman, J. T.; Brozell, S. R.; Cerutti, D. S.; Cheatham III, T. E.; Cisneros, G. A.; Cruzeiro, V. W. D.; Darden, T. A.; Forouzesh, N.; Ghazimirsaeed, M.; Giambaşu, G.; Giese, T.; Gilson, M. K.; Gohlke, H.; Goetz, A. W.; Harris, J.; Huang, Z.; Izadi, S.; Izmailov, S. A.; Kasavajhala, K.; Kaymak, M. C.; Kovalenko, A.; Kurtzman, T.; Lee, T. S.; Li, P.; Li, Z.; Lin, C.; Liu, J.; Luchko, T.; Luo, R.; Machado, M.; Manathunga, M.; Merz, K. M.; Miao, Y.; Mikhailovskii, O.; Monard, G.; Nguyen, H.; O'Hearn, K. A.; Onufriev, A.; Pan, F.; Pantano, S.; Rahnamoun, A.; Roe, D. R.; Roitberg, A.; Sagui, C.; Schott-Verdugo, S.; Shajan, A.; Shen, J.; Simmerling, C. L.; Skrynnikov, N. R.; Smith, J.; Swails, J.; Walker, R. C.; Wang, J.; Wang, J.; Wu, X.; Wu, Y.; Xiong, Y.; Xue, Y.; York, D. M.; Zhao, C.; Zhu, Q.; Kollman, P.A. (2024), Amber 2024, University of California, San Francisco.

[26]Wang, J.; Wang, W.; Kollman, P. A.; Case, D. A. Automatic atom type and bond type perception in molecular mechanical calculations. *J. Mol. Graph. Mod.* **2006**, *25*.2778

[27]Maier, J. A.; Martinez, C.; Kasavajhala, K.; Wickstrom, L.; Hauser, K. E.; Simmerling, C. ff14SB: Improving the Accuracy of Protein Side Chain and Backbone Parameters from ff99SB. *J. Chem. Theory Comput.* **2015**, *11*, 3696-3713.

[28]Strajbl, M.; Sham, Y. Y.; Villà, J.; Chu, Z. T.; Warshel, A. Calculations of Activation Entropies of Chemical Reactions in Solution. *J. Phys. Chem. B.* **2000**, *104*, 4578-4584.

[29]Darden, T.; York, D.; Pedersen, L.; Particle mesh Ewald: An N·log(N) method for Ewald sums in large systems. *J. Chem. Phys.***1993**, *98*, 10089-10092.

[230]The PyMOL Molecular Graphics System, Version 2.0 Schrödinger, LLC.

[31]Gaussian 16, Revision C.01, Frisch, M. J.; Trucks, G. W.; Schlegel, H. B.; Scuseria, G. E.; Robb, M. A.; Cheeseman, J. R.; Scalmani, G.; Barone, V.; Petersson, G. A.; Nakatsuji, H.; Li, X.; Caricato, M.; Marenich, A. V.; Bloino, J.; Janesko,

B. G.; Gomperts, R.; Mennucci, B.; Hratchian, H. P.; Ortiz, J. V.; Izmaylov, A. F.; Sonnenberg, J. L.; Williams-Young, D.; Ding, F.; Lipparini, F.; Egidi, F.; Goings, J.; Peng, B.; Petrone, A.; Henderson, T.; Ranasinghe, D.; Zakrzewski, V. G.; Gao, J.; Rega, N.; Zheng, G.; Liang, W.; Hada, M.; Ehara, M.; Toyota, K.; Fukuda, R.; Hasegawa, J.; Ishida, M.; Nakajima, T.; Honda, Y.; Kitao, O.; Nakai, H.; Vreven, T.; Throssell, K.; Montgomery Jr., J. A.; Peralta, J. E.; Ogliaro, F.; Bearpark, M. J.; Heyd, J. J.; Brothers, E. N.; Kudin, K. N.; Staroverov, V. N.; Keith, T. A.; Kobayashi, R.; Normand, J.; Raghavachari, K.; Rendell, A. P.; Burant, J. C.; Iyengar, S. S.; Tomasi, J.; Cossi, M.; Millam, J. M.; Klene, M.; Adamo, C.; Cammi, R.; Ochterski, J. W.; Martin, R. L.; Morokuma, K.; Farkas, O.; Foresman, J. B.; Fox, D. J. Gaussian, Inc., Wallingford CT, 2016.

[32]Chai, J.-D.; Head-Gordon, M. Long-range corrected hybrid density functionals with damped atom–atom dispersion corrections. *Phys. Chem. Chem. Phys.* **2008**, *10*, 6615-6620.

[33]Weigend, F.; Ahlrichs, R. Balanced basis sets of split valence, triple zeta valence and quadruple zeta valence quality for H to Rn: Design and assessment of accuracy. *Phys. Chem. Chem. Phys.* **2005**, *7*, 3297-3305.

[34]Marenich, A. V.; Cramer, C. J.; Truhlar, D. G. Universal Solvation Model Based on Solute Electron Density and on a Continuum Model of the Solvent Defined by the Bulk Dielectric Constant and Atomic Surface Tensions. *J. Phys. Chem. B.* **2009**, *113*, 6378-6396.

[35]Schlegel, H. B. in *Modern Electronic Structure Theory*, ed.: Yarkony, D. R. World Scientific Publishing, Singapore, **1994**.

[36]Fukui, K. The path of chemical reactions - the IRC approach. *Acc. Chem. Res.* **1981**, *14*, 363-368.

[37]Plata, R. E.; Singleton, D. A. A Case Study of the Mechanism of Alcohol-Mediated Morita Baylis-Hillman Reactions. The Importance of Experimental Observations. *J. Am. Chem. Soc.* **2015**, *137*, 3811-3826.

[38]Legault, C. Y. CYLview, 1.0b, Université de Sherbrooke 2009, http://www.cylview.org

[39]Johnson, E. R.; Keinan, S.; Mori-Sanchez, P.; Contreras-Garcia, J.; Cohen, A. J.; Yang, W. Revealing Noncovalent Interactions. *J. Am. Chem. Soc.* **2010**, *132*, 6498-6506.

[40]Boto, R. A.; Peccati, F.; Laplaza, R.; Quan, C.; Carbone, A.; Piquemal, J.-P.; Maday, Y.; Contreras-García, J.; NCIPLOT4.

[41]Schulten, M. VMD - Visual Molecular Dynamics, *J. Mol. Graph.* **1996**, *14*, 33-38.

[42]Williams, T.; Kelley, C. Gnuplot 4.5: an interactive plotting program., **2011**, http://gnuplot.info.
